# Synthesis of *S*-Glycoside Building Blocks as Mimetics of the Repeating d-GlcN-α-1,4-d-GlcA Heparan Sulfate Disaccharide

**DOI:** 10.3390/molecules29235809

**Published:** 2024-12-09

**Authors:** Conor O’Shea, Gavin J. Miller

**Affiliations:** School of Chemical and Physical Sciences and Centre for Glycoscience, Keele University, Keele, Staffordshire ST5 5BG, UK; c.oshea@keele.ac.uk

**Keywords:** heparan sulfate, *S*-glycoside, mimetic, glycosylation, substitution

## Abstract

Heparan sulfate (HS), a sulfated linear carbohydrate that decorates the cell surface and extracellular matrix, is a key regulator of biological processes. Owing to the inherent structural complexity of HS, structure-to-function studies with its ligands are required, and materials to improve the understanding of such interactions are therefore of high importance. Herein, the synthesis of novel *S*-linked GlcN-α(1→4)-GlcA disaccharide building blocks is detailed. Initial attempts at constructing the desired disaccharide using d-GlcN donors and d-Glc/GlcA acceptors via an *S*-glycosylation failed. Reversing the reactivity polarity of the monosaccharide building blocks enabled successful S_N_2 coupling using α-d-GlcN thiohemiacetals and d-galactosyl triflates. Subsequent C6-oxidation furnished the desired *S*-linked GlcN-α(1→4)-GlcA disaccharide building blocks on a gram scale. Such disaccharides offer potential for incorporation into wider synthetic HS sequences to provide glycomimetic tools.

## 1. Introduction

**Figure 1:**
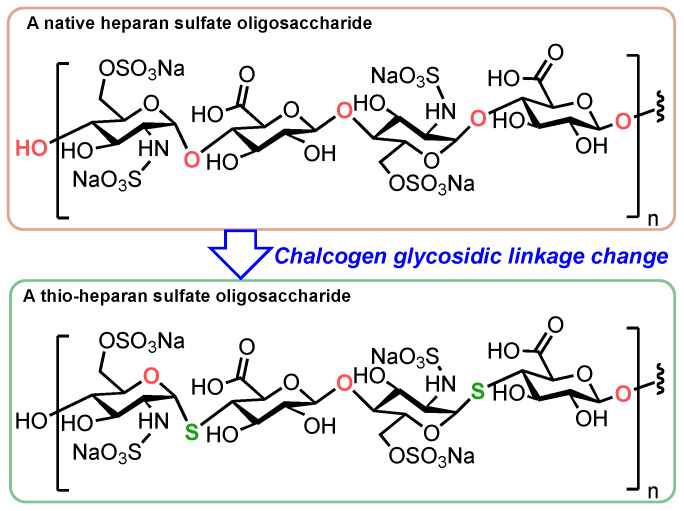
Chemical structure of HS and indicative *S*-linked HS consisting of repeating disaccharide units composed of glucosamine (d-GlcN) and a uronic acid. The amino sugar can be *N*-sulfated or *N*-acetylated, the uronic acid d-GlcA can be epimerised to L-IdoA, and saccharide units can be variably sulfated, most commonly at O6 of d-GlcN and O2 of L-IdoA.

Glycosaminoglycans (GAGs) are an omnipresent glycan in nature, existing on most animal cell surfaces and in the surrounding extracellular matrix. They are extremely diverse, containing a linear and structurally heterogeneous anionic glycan chain, and impart important biological functions by binding to different growth factors, enzymes, morphogens, cell adhesion molecules, and cytokines [1]. One GAG in particular, heparan sulfate (HS, Figure 1), is involved in mediating mammalian cell function, which is exemplified by its interaction with fibroblast growth factors (FGFs), a protein family involved in cell proliferation, differentiation, and angiogenesis [2]. HS also mediates many pathological conditions, including cancer [3], Alzheimer’s disease [4], and viral infections, such as SARS-CoV-2 [5,6], HIV [7], and HSV [8], alongside bacterial infectivity events [9].

Therefore, there is a requirement to interrogate and fundamentally understand the role that HS plays in such processes. This will allow the formulation of a precise picture of HS-mediated structure-to-function relationships and initiate the development of a new understanding of the pathophysiologies they control. However, any advance in HS structure-to-function knowledge is predicated on the ability to efficiently access a viable HS glycan toolkit. Within this requirement, chemically altered variants or mimetics of native HS are required to study the processes surrounding HS biological function. One such modification is the replacement of glycosidic linkage oxygen with sulfur (Figure 1). Introducing this chalcogen imparts improved hydrolytic stability to the linkage, and wider *S*-glycosidic forms have underpinned advances in glycoscience understanding over the past quarter of a century [10]. Choosing sulfur to replace oxygen gives a similar conformational preference about the (now longer) thioglycosidic and aglyconic bonds, both when in solution and when complexed with a protein [11]. This, combined with sulfur’s lower affinity for protons, confers the overall reduced susceptibility of the *S*-glycan to hydrolysis [12].

Notwithstanding, *S*-glycoside analogues of HS remain underexplored [13,14], mostly as effective synthetic methodologies for their synthesis have not been broadly developed. Building on our related work on creating a variety of building blocks to incorporate sulfur within native sugars [15,16,17,18,19], herein, we establish a methodology to access d-GlcN-α-1,4-*S*-d-GlcA disaccharide building blocks, as the entry point to explore oligosaccharide assembly and derived *S*-linked HS glycomimetics.

### Disaccharide Design

**Figure 2:**
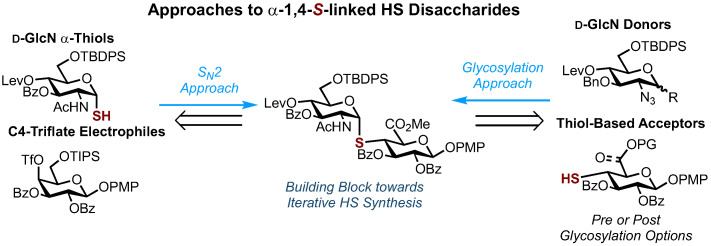
Retrosynthetic approaches to *S*-linked HS disaccharide building blocks using glycosylation or nucleophilic substitution. R = appropriate anomeric donor.

*S*-linked d-GlcN(α-1→4)-*S*-d-GlcA was chosen as the target disaccharide building block. The retrosynthetic design involved two separate approaches, the first seeking linkage formation using glycosylation with a thiol acceptor and the second using an S_N_2 reaction to complete chalcogen incorporation (Figure 2). For the glycosylation approach, the donor was masked with a non-participating azide at C2 alongside a bulky TBDPS group at C6 to promote α-glycosylation selectivity and act as a surrogate for future *O*-sulfation. Furthermore, C4 was masked with an orthogonal temporary protecting group (Lev) to facilitate chain elongation. Both glucuronate and glucose acceptors were proposed with the thiol at C4 and PMP at the reducing end, the latter to enable later conversion to an appropriate donor [20,21,22].

This approach complements related efforts to access *S*-glycosides, including (i) protecting group-free methods using the glycosylation of fluoride donors [23]; (ii) the displacement of anomeric glycosyl bromides with thiol nucleophiles to access *S*-linked chitin analogues [24,25]; (iii) β-thiomannoside synthesis using the displacement of an anomeric triflate by a thiol; and (iv) *S*-sialosylglycoside probe synthesis using thioacetate donors and Et_2_NH with triflate electrophiles [26]. In the case of GAGs, an *S*-linked β-(1-4) system between d-GlcA and d-GlcN was reported by Yu [27] as part of a trisaccharide mimetic and within a multivalent construct by Kovensky [28]. Relatedly, Withers reported the synthesis of a β-linked *S*-glycoside to explore chondroitin sulfate lyase [29]. To the best of our knowledge, however, there are limited reports accessing α-linked *S*-disaccharides [30,31,32] and none enabling building blocks for mimetic HS/GAG oligosaccharide synthesis.

## 2. Results and Discussion

### 2.1. Four-Exploring a Glycosylation Approach Towards α-1,4-S-Linked HS Disaccharides

#### 2.1.1. C4-Thio Acceptor Synthesis

**Scheme 1:**
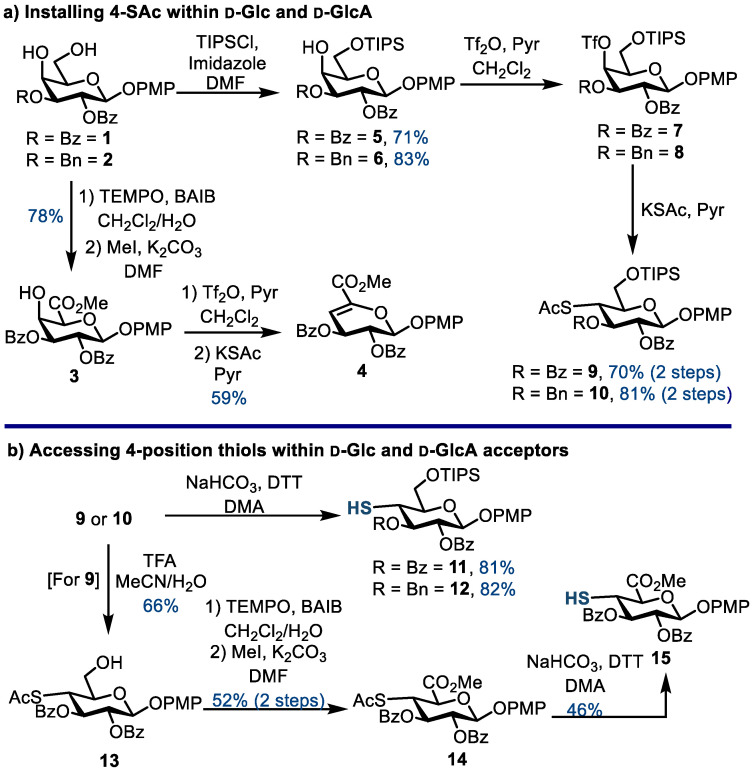
Synthesis of C4-thio d-glucose and d-glucuronate acceptors **11**, **12**, and **15**. (**a**) Unsuccessful approach to uronate **4** attempting to install thioacetate on d-galacturonate **3**, followed by synthesis of glucose 4-thioacetates **9** and **10**. (**b**) C4-Thiol acceptor synthesis for d-gluco and d-glucuronate systems, **11**, **12**, and **15**.

In order to access an appropriate C4-thio acceptor for glycosidation with a d-GlcN donor, we selected a d-Gal configured starting material, proposing that the axial *O*-4 position be inverted via an S_N_2 reaction, using an appropriate thiol nucleophile. Our first approach sought the inclusion of the chalcogen within a galacturonate building block (Scheme 1a). Accordingly, galactose diol **1**, which was accessed from galactose in six steps and 21% overall yield (see Supplementary Materials), was treated with TEMPO/BAIB, and the resultant carboxylate was methylated using MeI to afford uronate **3** in 78% yield. To convert *O*-4 to an appropriate leaving group for inversion, uronate **3** was treated with Tf_2_O in CH_2_Cl_2_/pyridine at 0 °C to furnish the *O*-4 triflate, and treatment with KSAc in CH_2_Cl_2_/pyridine for 2 h at room temperature was completed thereafter. ^1^H NMR analysis of this reaction indicated that none of the desired C4-thioacetate had formed. Instead, C4-C5 elimination product **4** was isolated in 59% yield.

To circumvent the formation of alkene **4**, C4 inversion prior to C6 oxidation was pursued, concomitantly enabling access to a d-Glc-based C4-thio acceptor for the exploration of a post-glycosylation oxidation to the uronate. As such, we returned to galactose diol **1** and completed *O*-6 TIPS protection using TIPSCl and imidazole to furnish silylated alcohol **5** in 71% yield (Scheme 1a), noting an amount of *O*-benzoyl migration (C3 to C4) side product (**S11**, see Supplementary Materials) forming after 2 h. C4 inversion of **5** to the glucose configuration proceeded smoothly via triflate **7,** and C4-thioacetate **9** was isolated in 70% yield over two steps. A ^4^*C*_1_ d-gluco configuration was supported by ^1^H NMR, revealing a doublet of doublets at δ_H_ = 5.86 ppm, corresponding to H-3 (*^3^J_H3-H4_* = 11.0 Hz). Furthermore, and with a view to exploring the effect of incorporating an activating C3 protecting group (Bn), we synthesised an analogous thioacetate, **10**, starting from known C3-OBn galactose diol **2** (see Supplementary Materials), noting comparable yields for this process versus the C3-OBz route (10% over nine steps for **9** versus 9% over eleven steps for **10**, starting from α/β-d-galactose). With C4-thioacetates to hand, two separate routes were pursued towards C4-thio d-gluco and d-glucuronate acceptors **11**, **12**, and **15** (Scheme 1b). Firstly, the thioesters within **9** and **10** were cleaved using DTT to generate acceptors **11** and **12** in 81% and 82% yields, respectively. Secondly, 6-*O*-TIPS deprotection of **9** using TFA furnished **13** in 66% yield alongside a 6-*O*Ac migration side product in 7% yield (**S12**, see Supplementary Materials). This was followed by C6 TEMPO/BAIB oxidation and methylation of **13** to give ester **14** in 52% yield over two steps. Similar *S*-deacetylation using DTT generated d-glucuronate acceptor **15** in 46% yield. With three appropriate thiol-based acceptors to hand, we pursued their glycosidation.

#### 2.1.2. Attempted Glycosidations of C4-Thioacceptors with d-GlcN Donors

**Scheme 2:**
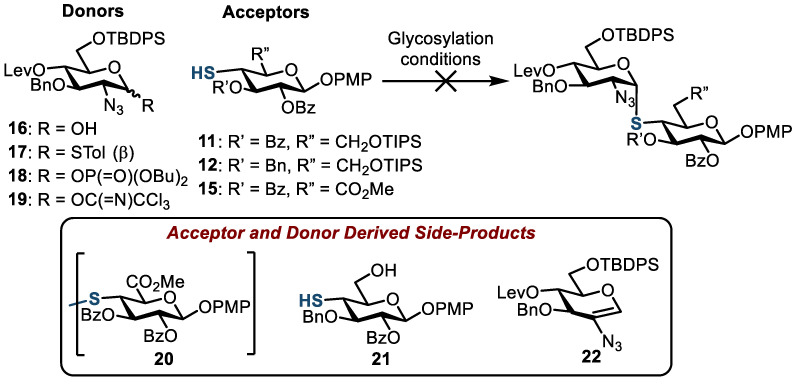
Attempted glycosylation using C4-thiol acceptors **11**, **12**, and **15**. Glycosylation activation conditions trialled: Ph_2_SO/Tf_2_O or TMSOTf, including the incorporation of DTT to prevent the formation of glucuronate acceptor-derived disulfide **20**. Side products **20** and **21** were isolated and fully characterised (see Supplementary Materials), and **22** matched data reported previously [20].

Acceptors **11**, **12**, and **15** were each screened in glycosylation reactions using hemiacetal donor **16** and a Ph_2_SO/Tf_2_O promoter system on a 100 mg scale (Scheme 2). These conditions were selected following previous use by us to synthesise the oxygenated analogue of the target *S*-disaccharide [20]. Unfortunately, no disaccharide formation was observed using any of the three acceptors; only acceptor (disulfide **20** and alcohol **21**) and donor (glycal **22**) derived materials could be isolated and were characterised. As such, we explored different monosaccharide donors, including known thioglycoside **17** and imidate **19** [20], alongside novel phosphate **18**. Unfortunately, the reaction of any of **17**, **18**, and **19** with acceptor **12** furnished no disaccharide product; only donor- and acceptor-derived side products were again evident in crude reaction mixtures. In light of this unsuccessful approach, we attempted to install the required α-*S*-linkage through the donor and thus sought the appropriate synthesis of α-thiols as the nucleophile source for reaction with an appropriate C4-triflate (e.g., **7**).

### 2.2. A Nucleophilic Substitution Approach Towards α-1,4-S-Linked HS Disaccharides 

**Scheme 3:**
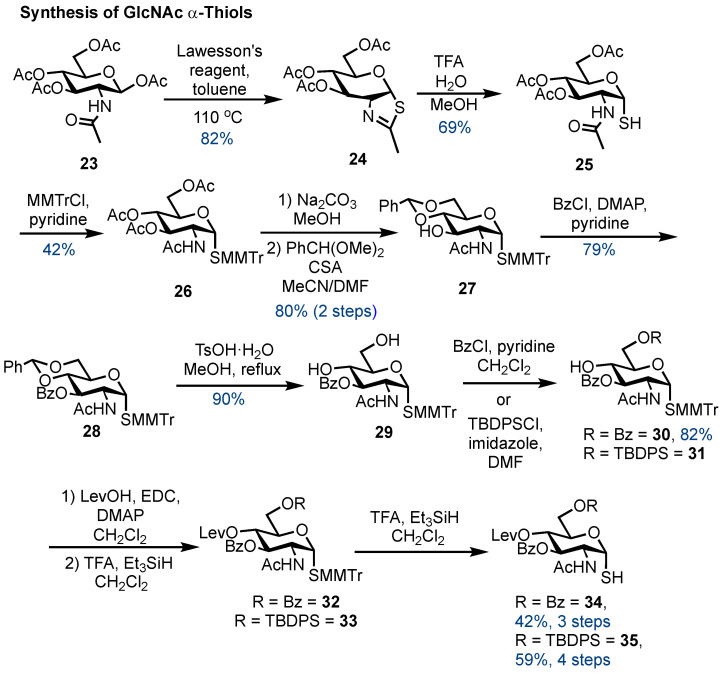
Synthesis of anomeric thiols **33** and **35**. Harnessing Lawesson’s reagent to access a thiooxazoline, followed by manipulation to orthogonally protected α-thiols.

Several methods have been reported to access α-thiohemiacetals, including the regioselective opening of 1,6-anhydrosugars using sulfur nucleophiles [33], the displacement of anomeric chlorides using thiourea [34], and the glycosylation of imidates using sulfur acceptors [35] from hemiacetals using Lawesson’s reagent [36] and via thiooxazolines in the case of amino sugars [37]. As our target disaccharides contained a non-reducing d-GlcNAc, we selected Lawesson’s reagent to target anomeric α-thiols **34** and **35** (Scheme 3). 

The treatment of commercial acetate **β-23** with Lawesson’s reagent yielded thiooxazoline **24** in 82% yield, and acid-mediated hydrolysis furnished the desired thiohemiacetal **α-25** in 69% yield. A protocol to install a monomethoxytrityl (MMTr) protecting group on the anomeric α-thiol was followed next [38], forming *S*-MMTr-protected **26** in 42% yield. Global deacetylation using Na_2_CO_3_ in MeOH and *O*-4,6-benzylidene protection yielded alcohol **27** in 80% yield over two steps. The C3-benzoylation of alcohol **27** using BzCl with catalytic DMAP, followed by benzylidene deprotection using TsOH·H_2_O, generated diol **29** in 71% yield over two steps as an appropriate material to install orthogonal protecting groups at C4 and C6. Accordingly, the regioselective benzoylation of **29** was completed to form *O*-6 benzoate **30** using BzCl and pyridine in CH_2_Cl_2_ at −30 °C in 82% yield [39]. Finally, the *O*-4 Lev protection of alcohol **30** using levulinic acid, EDC, and DMAP, followed by MMTr cleavage using TFA and triethylsilane, generated the desired α-thiohemiacetal **34** in 42% yield over three steps. Similar transformations were also completed from diol **29** to generate an *O*-6-TBDPS building block **35** (Scheme 3), incorporating the potential to engage orthogonal *O*-6 sulfation in downstream HS sequences. Finally, to evaluate an orthogonal form of d-GlcN, we synthesised C2-azido α-thiol **36** (see Supplementary Materials). 

**Scheme 4:**
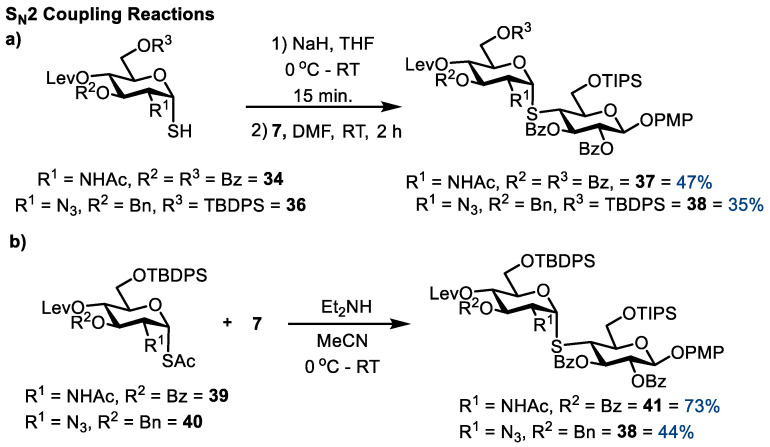
Synthesis of S-linked disaccharides **37**, **38**, and **41** using thioacetate-protected anomeric thiols. (**a**) Low-yielding approach using NaH to deprotonate the α-thiol. (**b**) Completing *S*-linked disaccharide synthesis using in situ thiol release from the thioacetate.

An S_N_2 coupling reported for the synthesis of a chondroitin sulfate *S*-linked disaccharide was selected [29], using NaH to deprotonate anomeric thiol **34**, prior to reaction with triflate **7** on a 100 mg scale (Scheme 4a). The desired *S*-disaccharide **37** was isolated in 47% yield, alongside amounts of an inseparable mixture of C4-C5 and C3-C4 elimination products (see Supplementary Materials), which were derived from **7** and accounted for the reduced yield of **37**. Thiohemiacetal **36** was also trialled, forming the required *S*-disaccharide in 35% yield. The low yield was again attributed to the observation of uronate-derived elimination, but an additional side product, a C4-OH d-glucoside, was also isolated and characterised (**S18**, see Supplementary Materials). 

Overall, given the low yields observed using NaH to deprotonate anomeric thiols as a means to access *S*-linked disaccharides, we explored an alternative approach using anomeric thioacetates, which was mediated by in situ thiol release using Et_2_NH [40]. Accordingly, thioacetates **39** and **40** (see Supplementary Materials for details of synthesis) were converted to *S*-disaccharides **41** and **38** in 73% and 44% yields, respectively (Scheme 4b), using MeCN as a solvent and conducting the reactions at −5 °C. This procedure proved scalable, affording >1.0 g quantities of **41**. Gratifyingly, only traces of the previously observed triflate-derived elimination side products were observed (<5% by crude ^1^H NMR), and ^1^H NMR analysis confirmed successful S_N_2 coupling to furnish the α-(1→4)-*S*-linked disaccharide, revealing a doublet of doublets at δ_H_ = 5.70 ppm, corresponding to H-3 (^3^*J*_H3-H2_ = 9.6 Hz, ^3^*J*_H3-H4_ = 10.8 Hz), anti-periplanar to H-4. The anomeric stereochemistry of the new glycosidic linkage was confirmed as 1,2-*cis*, with H-1 observed as a doublet at δ_H_ = 5.61 ppm 1 (^3^*J*_H1′-H2′_ = 5.3 Hz). Furthermore, the chemical shift for H-4 within **41** was upfield at δ_H_ = 3.25 ppm, supporting *S*-glycoside formation. 

**Scheme 5:**
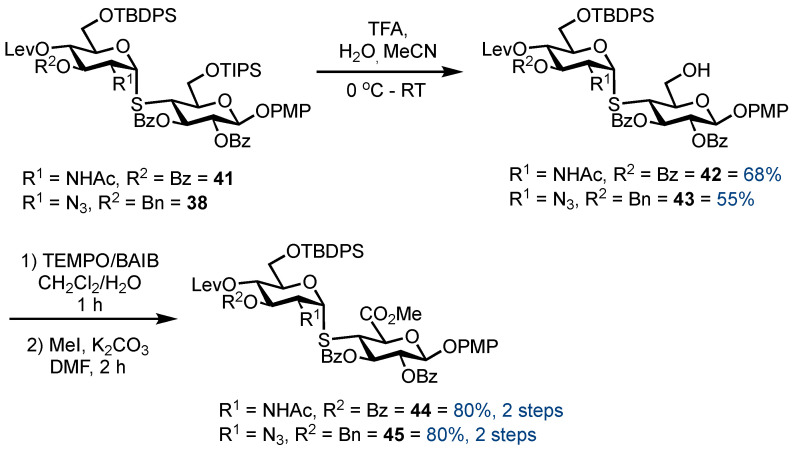
Oxidation of reducing end d-gluco component to the uronate and delivering access to *S*-linked HS disaccharide building blocks **44** and **45**.

Finally, each of *S*-linked disaccharides **41** and **38** was subjected to reducing end d-*gluco-O*-6 modification to form the desired d-GlcN-α-(1→4)-*S*-d-GlcA building blocks. Accordingly, *O*-6-TIPS cleavage was completed using TFA/H_2_O in MeCN to afford alcohols **42** and **43** in 68% and 55% yields (Scheme 5). To prevent unwanted ester migration, these reactions were quenched after 6 h when the starting material still remained, thus attributing to the moderate yields obtained. Subsequent TEMPO/BAIB-mediated oxidation, followed by treatment of the carboxylic acids with MeI and K_2_CO_3_, furnished the desired methyl esters **44** and **45**, both in 80% yields. HMRS analysis confirmed that no oxidation of the internal *S*-glycosidic linkage had occurred.

## 3. Experimental Section

### 3.1. General Experimental

All chemicals were purchased from Acros Organics (Waltham, MA, USA), Alfa Aesar (Waltham, MA, USA), Biosynth Carbosynth (Staad, Switzerland), Fisher Scientific (Waltham, MA, USA), Fluorochem (Derbyshire, UK), Sigma Aldrich (St. Louis, MO, USA) or TCI Chemicals (Tokyo, Japan) and were used without further purification unless otherwise stated. NMR spectra were recorded on a Bruker Avance 400 spectrometer (Billerica, MA, USA). The chemical shift data are given as δ in units of parts per million (ppm) relative to tetramethylsilane, where δ = 0.00. The number of protons (n) for a given resonance is indicated by nH. The multiplicity of each signal is indicated as follows: s (singlet), br s (broad singlet), d (doublet), t (triplet), q (quartet), dd (doublet of doublets), ddd (doublet of doublet of doublets), dddd (doublet of doublet of doublet of doublets), dt (doublet of triplets), tt (triplet of triplets), td (triplet of doublets), or m (multiplet). Coupling constants (*J*) are quoted in Hz and calculated to the nearest 0.1 Hz. Anhydrous DMF, MeOH, pyridine, and Et_3_N were obtained from Sure/SealTM bottles via chemical suppliers. Anhydrous THF, CH_2_Cl_2_, and toluene were obtained by passing a solvent through activated alumina columns, dispensed from a PureSolv MD ASNA solvent purification system (Jeddah, Saudi Arabia), and stored over 4 Å molecular sieves. Unless otherwise stated, all reactions were conducted using anhydrous solvents in an atmosphere of N_2_, which was passed through a Drierite^®^ drying column (Xenia, OH, USA). Brine and NaHCO_3_ refer to saturated aqueous solutions of NaCl and NaHCO_3_, respectively. High-resolution mass spectra were measured at the EPSRC National Mass Spectrometry Facility at Swansea University or Keele University Accurate Mass Service. Analytical thin layer chromatography (TLC) was carried out on pre-coated 0.25 mm Merck KGaA 60 F254 silica gel plates (Darmstadt, Germany). Visualisation was conducted using the adsorption of UV light or thermal development after dipping in a methanolic solution of sulfuric acid (5% *v*/*v*). Manual column chromatography was carried out on silica gel (VWR International 40–63 μm, Leicestershire, UK) under a positive pressure of compressed air. 

### 3.2. General Procedure A: Coupling Using NaH

A solution of the thiohemiacetal (1.00 equiv.) in DMF (0.12 M) was treated with NaH (60% dispersion in mineral oil, 1.00 equiv.) at 0 °C. The reaction mixture was stirred gradually to RT over 15 min. A solution of triflate 7 in DMF (1.50 equiv., 0.2 M) was added at 0 °C, and the reaction was left stirring at RT for 2 h or until a TLC analysis (95/5, CH_2_Cl_2_/Et_2_O) revealed an almost full conversion of 7 to a lower R_f_ spot. The reaction mixture was then concentrated *in vacuo* to give a yellow residue. 

### 3.3. General Procedure B: Coupling Using Et_2_NH

A solution of the anomeric *S*-acetate (1.00 equiv.) and triflate **7** (1.10 equiv.) in MeCN (0.1 M) was cooled to −5 °C and treated with the dropwise addition of Et_2_NH (13.0 equiv.). The reaction was stirred at this temperature for 1 h or until a TLC analysis (95/5, CH_2_Cl_2_/Et_2_O) revealed the conversion of the triflate to a lower R_f_ spot. The reaction mixture was then concentrated *in vacuo* to give a yellow residue. 

### 3.4. General Procedure C: O-6 TIPS Cleavage

A solution of *O*-6 TIPS-protected *S*-linked disaccharide (1.00 equiv.) in MeCN (0.1 M) was treated with TFA (8.00 equiv.) and H_2_O (10.0 equiv.). The reaction was left stirring at RT for 3 h. The further addition of TFA (4.00 equiv.) and H_2_O (10.0 equiv.) was made, and the reaction was left stirring for another 3 h. The reaction was diluted in CH_2_Cl_2_ and washed with NaHCO_3_. The aqueous layer was extracted with CH_2_Cl_2_ (×2), and the combined organic layers were washed with brine, dried over anhydrous MgSO_4_, filtered, and concentrated *in vacuo* to yield a white solid. 

### 3.5. General Procedure D: TEMPO/BAIB and MeI Oxidation/Methylation 

A solution of 6-OH *S*-linked disaccharide (1.00 equiv.) in CH_2_Cl_2_/H_2_O (2/1, 0.2 M) was treated with TEMPO (0.20 equiv.) and BAIB (2.50 equiv.) at 0 °C. The reaction was left stirring for 1 h at RT, whereupon a TLC analysis (14/7/1, EtOAc/MeOH/H_2_O) revealed the full conversion of the starting material to a lower R_f_ spot. The reaction was diluted in EtOAc and quenched with Na_2_S_2_O_3_. The aqueous layer was separated and acidified to pH = 2 using 1 M HCl and then extracted with EtOAc (×5). The combined organic layers were washed with brine, dried over anhydrous MgSO_4_, filtered, and concentrated *in vacuo* to give the crude acid. A solution of this material in DMF (0.3 M) was treated with K_2_CO_3_ (3.00 equiv.) and MeI (3.00 equiv.). The reaction was stirred at RT for 2 h, whereupon a TLC analysis (1/1, hexane/EtOAc) revealed the full conversion of the starting material to a higher R_f_ spot. The reaction mixture was diluted in CH_2_Cl_2_ and washed with H_2_O. The aqueous layer was extracted with CH_2_Cl_2_. The organic layers were combined and washed with brine, dried over anhydrous MgSO_4_, filtered, and concentrated *in vacuo* to generate an oil.

### 3.6. Experimetnal Procedures for Compounds ***3*** to ***45***

Methyl (*p*-methoxyphenyl 2,3-di-*O*-benzoyl-β-d-galactopyranosid) uronate (**3**)

A suspension of *p*-methoxyphenyl 2,3-di-*O*-benzoyl-β-d-galactopyranoside **1** (200 mg, 0.40 mmol, 1.00 equiv.) in CH_2_Cl_2_/H_2_O (2 mL, 2/1) was treated with TEMPO (13 mg, 0.08 mmol, 0.20 equiv.) and BAIB (322 mg, 1.00 mmol, 2.50 equiv.) at 0 °C. The reaction was stirred at RT for 1 h. A TLC analysis (14/7/1, EtOAc/MeOH/H_2_O) showed full conversion of the starting material to a lower R_f_ spot. The reaction was quenched with Na_2_S_2_O_3_ (2 mL) and diluted with EtOAc (5 mL). The aqueous layer was isolated and acidified to pH = 2 using 1 M HCl and extracted with EtOAc (5 × 5 mL). The combined organic layers were washed with brine (30 mL), dried over anhydrous MgSO_4_, filtered, and concentrated *in vacuo* to give a yellow solid. A solution of this crude material in DMF (1.5 mL) and K_2_CO_3_ (170 mg, 1.20 mmol, 3.00 equiv.) was treated with MeI (75 µL, 1.20 mmol, 3.00 equiv.) at 0 °C. The reaction was stirred at RT for 2 h, whereupon a TLC analysis (1/1, hexane/EtOAc) revealed the full conversion of the starting material to a higher R_f_ spot. The reaction mixture was diluted with CH_2_Cl_2_ (5 mL) and washed with H_2_O (10 mL). The aqueous layer was separated and extracted with CH_2_Cl_2_ (2 × 10 mL). The organic layers were combined and washed with brine (25 mL), dried over anhydrous MgSO_4_, filtered, and concentrated *in vacuo* to give a brown syrup. The purification of this crude material via column chromatography (1/0 → 6/4, hexane/EtOAc) furnished **3** as a tan solid (160 mg, 0.31 mmol, 78%). R_f_ = 0.36 (1/1, hexane/EtOAc); m.p. 107–109 °C; ${\left[\alpha \right]}_{D}^{23}$ +120.9 (c = 0.50, CHCl_3_); ^1^H NMR (400 MHz, CDCl_3_) δ 8.04–7.96 (m, 4H, Ph), 7.57–7.50 (m, 2H, Ph), 7.42–7.38 (m, 4H, Ph), 7.02–6.97 (m, 2H, Ph), 6.80–6.74 (m, 2H, Ph), 5.99 (dd, *J* = 10.1, 7.8 Hz, 1H, H_2_), 5.40 (dd, *J* = 10.2, 3.2 Hz, 1H, H_3_), 5.13 (d, *J* = 7.8 Hz, 1H, H_1_), 4.73 (ddd, *J* = 5.7, 3.2, 1.4 Hz, 1H, H_4_), 4.45 (d, *J* = 1.3 Hz, 1H, H_5_), 3.85 (s, 3H, OCH_3_), 3.75 (s, 3H, OCH_3_), 2.61 (d, *J* = 6.0 Hz, 1H, C_4_-OH); ^13^C NMR (100 MHz, CDCl_3_) δ 167.5 (C=O), 165.8 (C=O), 165.2 (C=O), 155.9 (C_q_), 151.2 (C_q_), 133.6 (CH), 133.3 (CH), 130.0 (CH), 129.8 (CH), 129.3 (C_q_), 128.8 (C_q_), 128.54 (CH), 128.45 (CH), 119.6 (CH), 114.5 (CH), 101.4 (C_1_), 73.9 (C_5_), 73.4 (C_3_), 69.0 (C_2_), 68.3 (C_4_), 55.6 (OCH_3_), 52.8 (OCH_3_). HRMS (ESI^+^) *m*/*z* found (M+NH_4_)^+^ 540.1865; C_28_H_30_NO_10_ requires 540.1864.

Methyl (*p*-methoxyphenyl 2,3-di-*O*-benzoyl-4-deoxy-α-L-*threo*-hex-4-enopyranosid) uronate (**4**)

A solution of methyl (*p*-methoxyphenyl 2,3-di-*O*-benzoyl-β-d-galactopyranosid)uronate **3** (100 mg, 0.19 mmol, 1.00 equiv.) in CH_2_Cl_2_/pyridine (2.4 mL, 8/2) was treated with Tf_2_O (70 mL, 0.40 mmol, 2.30 equiv.) at 0 °C and allowed to stir at RT for 1 h. TLC (1/1, hexane/EtOAc) revealed the full conversion of the starting material to two higher R_f_ spots. The reaction was diluted with CH_2_Cl_2_ (10 mL) and washed with 1 M HCl (8 mL), NaHCO_3_ (8 mL), and brine (8 mL), dried over anhydrous MgSO_4_, filtered, and concentrated *in vacuo* to furnish the crude triflate as a yellow syrup. ^1^H NMR (400 MHz, CDCl_3_) selected signals: δ 5.97 (dd, *J* = 10.6, 8.0 Hz, 1H, H_2_), 5.78 (d, *J* = 2.3 Hz, 1H, H_4_), 5.59 (dd, *J* = 10.5, 3.0 Hz, 1H, H_3_), 5.18 (d, *J* = 8.0 Hz, 1H, H_1_), 4.61 (d, *J* = 0.8 Hz, 1H, H_5_), 3.87 (s, 3H, COCH_3_), 3.75 (s, 3H, OCH_3_); ^19^F NMR (377 MHz, CDCl_3_) δ −74.2. A solution of this crude material in CH_2_Cl_2_ (1.4 mL) was treated with KSAc (65.0 mg, 0.57 mmol, 3.10 equiv.) and pyridine (0.50 mL, 0.60 mmol, 3.00 equiv.) and stirred for 4 h. A TLC analysis (1/1, hexane/EtOAc) showed conversion to a higher R_f_ spot. The reaction was diluted with CH_2_Cl_2_ (8 mL), washed with NaHCO_3_ (8 mL) and brine (8 mL), dried over anhydrous MgSO_4_, filtered, and concentrated *in vacuo* to furnish a yellow solid. The purification of the crude material via column chromatography (1/0 → 8/2, hexane/EtOAc) furnished **4** as an off-white solid (56 mg, 0.11 mmol, 59%). R_f_ = 0.52 (1/1, hexane/EtOAc); m.p. 90–92 °C; ${\left[\alpha \right]}_{D}^{23}$ +1.34 (c = 0.9, CHCl_3_); ^1^H NMR (400 MHz, CDCl_3_) δ 8.14–8.09 (m, 2H, Ph), 8.06–8.01 (m, 2H, Ph), 7.60–7.58 (m, 2H, Ph), 7.49–7.42 (m, 4H, Ph), 7.12–7.07 (m, 2H, Ph), 6.87–6.82 (m, 2H, Ph), 6.50 (dd, *J* = 4.6, 1.4 Hz, 1H, H_4_), 5.94 (dd, *J* = 2.7, 0.9 Hz, 1H, H_1_), 5.71–5.67 (m, 2H, H_2_, H_3_), 3.84 (s, 3H, CH_3_), 3.78 (s, 3H, OCH_3_); ^13^C NMR (100 MHz, CDCl_3_) δ 164.5 (C=O), 164.0 (C=O), 161.1 (C=O), 154.7 (C_q_), 149.3 (C_q_), 141.6 (C_5_), 132.8 (C_q_), 132.5 (CH), 129.0 (CH), 128.9 (CH), 128.5 (C_q_), 127.8 (CH), 127.52 (CH), 127.49 (CH), 117.5 (CH), 113.7 (CH), 106.4 (C_4_), 94.7 (C_1_), 67.4 (C_2/3_), 63.3 (C_2/3_), 54.6 (OCH_3_), 51.7 (CH_3_). HRMS (ESI^+^) *m*/*z* found (M+Na)^+^ 527.1307; C_28_H_24_O_9_Na requires 527.1312.

*p*-Methoxyphenyl 2,3-di-*O*-benzoyl-6-*O*-(triisopropylsilyl)-β-d-galactopyranoside (**5**)

A solution of *p*-methoxyphenyl 2,3-di-*O*-benzoyl-β-d-galactopyranoside **1** (2.09 g, 4.22 mmol, 1.00 equiv.) and imidazole (0.865 g, 12.7 mmol, 3.00 equiv.) in anhydrous DMF (21 mL) was treated with the dropwise addition of TIPSCl (1.15 mL, 4.64 mmol, 1.10 equiv.) at 0 °C. The reaction was stirred at RT for 4 h, whereupon a TLC analysis (2:1, hexane/EtOAc) revealed the full conversion of the starting material to a higher R_f_ spot. The reaction was quenched with water (10 mL) and diluted with CH_2_Cl_2_ (20 mL). The aqueous layer was separated and extracted with CH_2_Cl_2_ (3 × 15 mL), and the combined organic layers were washed with brine (50 mL), dried over anhydrous MgSO_4_, filtered, and concentrated *in vacuo* to furnish a colourless syrup. The purification of this crude material via column chromatography (1/0 → 85/15, hexane/EtOAc) generated title compound **5** (1.96 g, 3.01 mmol, 71%) as a white solid, along with ester migrated product **S11** (322 mg, 0.495 mmol, 12%). Characterisation data for **5**: R_f_ = 0.79 (2/1, hexane/EtOAc); m.p. 72–73 °C; ${\left[\alpha \right]}_{D}^{25}$ +71.3 (c = 0.54, CHCl_3_); ^1^H NMR (400 MHz, CDCl_3_) δ 7.97–7.89 (m, 4H, Ph), 7.48–7.40 (m, 2H, Ph), 7.35–7.27 (m, 4H, Ph), 6.92–6.87 (m, 2H, Ph), 6.72–6.66 (m, 2H, Ph), 5.94 (dd, *J* = 10.3, 7.9 Hz, 1H, H_2_), 5.29 (dd, *J* = 10.3, 3.1 Hz, 1H, H_3_), 5.06 (d, *J* = 8.0 Hz, 1H, H_1_), 4.41 (t, *J* = 3.4 Hz, 1H, H_4_), 4.08–4.04 (m, 1H, H_6a_), 3.99 (dd, *J* = 10.5, 4.8 Hz, 1H, H_6b_), 3.73 (t, *J* = 5.1 Hz, 1H, H_5_), 3.68 (s, 3H, OCH_3_), 2.96 (d, *J* = 4.1 Hz, 1H, 4-OH), 1.08–0.98 (m, 21H, Si(C_3_H_7_)_3_); ^13^C NMR (100 MHz, CDCl_3_) δ 166.0 (C=O), 165.4 (C=O), 155.6 (C_q_), 151.4 (C_q_), 133.4 (CH), 133.1 (CH), 129.9 (CH), 129.7 (CH), 129.6 (C_q_), 129.2 (C_q_), 128.42 (CH), 128.36 (CH), 119.2 (CH), 114.4 (CH), 101.5 (C_1_), 74.6 (C_5_), 74.5 (C_3_), 69.7 (C_2_), 68.2 (C_4_), 63.4 (C_6_), 55.6 (OCH_3_), 17.93 (Si(CH(*C*H_3_)_2_)_3_), 17.90 (Si(CH(*C*H_3_)_2_)_3_), 11.8 (Si(*C*H(CH_3_)_2_)_3_). HRMS (ESI^+^) *m*/*z* found (M+Na)^+^ 673.2795; C_36_H_46_O_9_SiNa requires 673.2803.

*p*-Methoxyphenyl 2-*O*-benzoyl-3-*O*-benzyl-6-*O*-(triisopropylsilyl)-β-d-galactopyranoside (**6**)

A solution of *p*-methoxyphenyl 2-*O*-benzoyl-3-*O*-benzyl-β-d-galactopyranoside **2** (3.56 g, 7.41 mmol, 1.00 equiv.) and imidazole (1.51 g, 22.2 mmol, 3.00 equiv.) in anhydrous DMF (38 mL) was treated with the dropwise addition of TIPSCl (2.03 mL, 8.15 mmol, 1.10 equiv.) at 0 °C. The reaction was stirred at RT for 2.5 h, whereupon a TLC analysis (2/1, hexane/EtOAc) revealed almost full conversion of the starting material to a higher R_f_ spot. The reaction was quenched with water (15 mL) and diluted with CH_2_Cl_2_ (25 mL). The aqueous layer was separated and extracted with CH_2_Cl_2_ (3 × 20 mL), and the combined organic layers were washed with brine (60 mL), dried over anhydrous MgSO_4_, filtered, and concentrated *in vacuo* to generate a yellow oil. The purification of this crude material via column chromatography (100/0 → 95/5, CH_2_Cl_2_/Et_2_O) generated title compound **6** as a colourless syrup (3.91 g, 6.14 mmol, 83%). R_f_ = 0.28 (95/5, CH_2_Cl_2_/Et_2_O); m.p. 147–149 °C; ${\left[\alpha \right]}_{D}^{25}$ +8.6 (c = 0.60, CHCl_3_); ^1^H NMR (400 MHz, CDCl_3_) δ 8.05–7.99 (m, 2H, Ph), 7.62–7.55 (m, 2H, Ph), 7.46–7.44 (m, 2H, Ph), 7.24–7.14 (m, 4H, Ph), 6.94–6.88 (m, 2H, Ph), 6.74–6.68 (m, 2H, Ph), 5.71 (dd, *J* = 9.7, 8.0 Hz, 1H, H_2_), 4.93 (d, *J* = 8.0 Hz, 1H, H_1_), 4.70 (d, *J* = 12.4 Hz, 1H, PhC*H*H), 4.58 (d, *J* = 12.4 Hz, 1H, PhCH*H*), 4.18 (br s, 1H H_4_), 4.07 (dd, *J* = 10.1, 6.1 Hz, 1H, H_6a_), 3.99 (dd, *J* = 10.1, 5.9 Hz, 1H, H_6b_), 3.72 (s, 3H, OCH_3_) 3.72–3.68 (m, 1H, H_3_), 3.60 (t, *J* = 5.9 Hz, 1H, H_5_), 2.69–2.67 (m, 1H, C_4_-OH), 1.10–1.05 (m, 21H, Si(C_3_H_7_)_3_); ^13^C NMR (100 MHz, CDCl_3_) δ 165.4 (C=O), 155.3 (C_q_), 151.6 (C_q_), 137.2 (C_q_), 133.1 (CH), 130.0 (C_q_), 129.8 (CH), 128.5 (CH), 128.4 (CH), 128.0 (CH), 127.9 (CH), 118.8 (CH), 114.3 (CH), 101.2 (C_1_), 78.4 (C_2_), 75.4 (C_5_), 71.44 (PhCH_2_), 71.35 (C_3_), 65.9 (C_4_), 62.5 (C_6_), 55.6 (OCH_3_), 17.96 (Si(CH(*C*H_3_)_2_)_3_), 17.95 (Si(CH(*C*H_3_)_2_)_3_), 11.9 (Si(*C*H(CH_3_)_2_)_3_). HRMS (ESI^+^) *m*/*z* found (M+Na)^+^ 659.3011; C_36_H_48_O_8_SiNa requires 659.3011.

*p*-Methoxyphenyl 4-*S*-acetyl-2,3-di-*O*-benzoyl-6-*O*-(triisopropylsilyl)-β-d-glucopyranoside (**9**)

A solution of *p*-methoxyphenyl 2,3-di-*O*-benzoyl-6-*O*-(triisopropylsilyl)-β-d-galactopyranoside **5** (1.90 g, 2.92 mmol, 1.00 equiv.) in CH_2_Cl_2_/pyridine (34 mL, 5/1) was treated with the dropwise addition of Tf_2_O (1.13 mL, 6.72 mmol, 2.30 equiv.) at 0 °C. The reaction was left stirring for 1 h at 0 °C. A TLC analysis (8/2, hexane/EtOAc) revealed the full conversion of the starting material to a higher R_f_ spot. The reaction was diluted with CH_2_Cl_2_ (30 mL) and washed with 1 M HCl (30 mL), NaHCO_3_ (30 mL), and brine (30 mL), dried over anhydrous MgSO_4_, filtered, and concentrated *in vacuo* to give a yellow foam. A solution of this crude material in pyridine (21 mL) was treated with potassium thioacetate (1.01 g, 8.76 mmol, 3.00 equiv.), and the reaction was left stirring overnight. A TLC analysis (CH_2_Cl_2_) revealed the full conversion of the starting material to a lower R_f_ spot. The mixture was diluted with hexane/EtOAc (20 mL, 1/1) and washed with H_2_O (5 × 30 mL). The organic layer was separated and washed with brine (30 mL), dried over anhydrous MgSO_4_, filtered, and concentrated *in vacuo* to give a yellow residue. The purification of this crude material via column chromatography (100/0 → 85/15, hexane/EtOAc) furnished title compound **9** as an off-white solid (1.44 g, 2.03 mmol, 70% (over two steps)). R_f_ = 0.60 (CH_2_Cl_2_); m.p. 89–90 °C; ${\left[\alpha \right]}_{D}^{25}$ +63.9 (c = 0.25, CHCl_3_); ^1^H NMR (400 MHz, DMSO-*d*^6^) δ 7.87–7.83 (m, 2H, Ph), 7.81–7.77 (m, 2H, Ph), 7.66–7.60 (m, 2H, Ph), 7.52–7.45 (m, 4H, Ph), 6.93–6.88 (m, 2H, Ph), 6.82–6.78 (m, 2H, Ph), 5.86 (dd, *J* = 11.0, 9.4 Hz, 1H, H_3_), 5.52 (d, *J* = 8.0 Hz, 1H, H_1_), 5.34 (dd, *J* = 9.3, 8.0 Hz, 1H, H_2_), 4.23–4.18 (m, 1H, H_5_), 3.99 (t, *J* = 11.0 Hz, 1H, H_4_), 3.94 (dd, *J* = 11.8, 1.9 Hz, 1H, H_6a_), 3.88 (dd, *J* = 11.4, 5.4 Hz, 1H, H_6b_), 3.68 (s, 3H, OCH_3_), 2.23 (s, 3H, SCOCH_3_), 1.09–1.03 (m, 21H, Si(C_3_H_7_)_3_); ^13^C NMR (100 MHz, DMSO- *d*^6^) δ 192.7 (SC=O), 165.0 (C=O), 164.7 (C=O), 155.0 (C_q_), 150.6 (C_q_), 133.70 (CH), 133.68 (CH), 129.13 (CH), 129.11 (CH), 128.80 (CH), 128.77 (CH), 128.6 (C_q_), 118.1 (CH), 114.4 (CH), 98.8 (C_1_), 74.9 (C_5_), 73.2 (C_2_), 72.3 (C_3_), 62.9 (C_6_), 55.3 (OCH_3_), 43.3 (C_4_), 30.5 (SCO*C*H_3_), 17.73 (Si(CH(*C*H_3_)_2_)_3_), 17.70 (Si(CH(*C*H_3_)_2_)_3_), 11.4 (Si(*C*H(CH_3_)_2_)_3_). HRMS (ESI^+^) *m*/*z* found (M+NH_4_)^+^ 726.3137; C_38_H_52_NO_9_SSi requires 726.3127.

*p*-Methoxyphenyl 4-*S*-acetyl-2-*O*-benzoyl-3-*O*-benzyl-6-*O*-(triisopropylsilyl)-β-d-glucopyranoside (**10**)

A solution of *p*-methoxyphenyl 2-*O*-benzoyl-3-*O*-benzyl-6-*O*-(triisopropylsilyl)-β-d-galactopyranoside **6** (2.91 g, 4.57 mmol, 1.00 equiv.) in CH_2_Cl_2_/pyridine (49 mL, 5/1) was treated with the dropwise addition of Tf_2_O (1.77 mL, 10.5 mmol, 2.30 equiv.) at 0 °C. The reaction was left stirring for 50 min at 0 °C. A TLC analysis (CH_2_Cl_2_) revealed the full conversion of the starting material to a higher R_f_ spot. The reaction was diluted with CH_2_Cl_2_ (30 mL) and washed with 1 M HCl (30 mL), NaHCO_3_ (30 mL), and brine (30 mL), dried over anhydrous MgSO_4_, filtered, and concentrated *in vacuo* to furnish triflate **8** as a yellowish foam. It was used in the next step without further purification. ^1^H NMR (400 MHz, CDCl_3_) δ 7.99–7.93 (m, 2H, Ph), 7.63–7.56 (m, 1H, Ph), 7.45–7.43 (m, 2H, Ph), 7.22–7.19 (m, 3H, Ph), 7.16–7.09 (m, 2H, Ph), 6.89–6.84 (m, 2H, Ph), 6.74–6.69 (m, 2H, Ph), 5.64 (dd, *J* = 10.1, 8.1 Hz, 1H, H_2_), 5.45 (d, *J* = 2.8 Hz, 1H, H_4_), 4.99 (d, *J* = 8.0 Hz, 1H, H_1_), 4.80 (d, *J* = 12.7 Hz, 1H, PhC*H*H), 4.52 (d, *J* = 12.7 Hz, 1H, PhCH*H*), 3.98 (dd, *J* = 10.3, 6.5 Hz, 1H, H_6a_), 3.92 (dd, *J* = 10.3, 6.9 Hz, 1H, H_6b_), 3.84 (dd, *J* = 10.1, 2.9 Hz, 1H, H_3_), 3.77 (t, *J* = 6.7 Hz, 1H, H_5_), 3.72 (s, 3H, OCH_3_), 1.16–1.02 (m, Si(C_3_H_7_)_3_); ^19^F NMR (377 MHz, CDCl_3_) δ −73.5. A solution of crude **8** in pyridine (32 mL) was treated with KSAc (1.56 g, 13.7 mmol, 3.00 equiv.) and left stirring overnight. A TLC analysis (CH_2_Cl_2_) revealed the full conversion to three lower R_f_ spots. The mixture was diluted with CH_2_Cl_2_ (20 mL) and washed with 1 M HCl (2 × 30 mL) and NaHCO_3_ (30 mL), dried over anhydrous MgSO_4_, filtered, and concentrated *in vacuo* to give a brown residue. The purification of this crude material via column chromatography (100/0 → 85/15, hexane/EtOAc) furnished title compound **10** as a yellowish/brown oil (2.58 g, 3.71 mmol, 81%). R_f_ = 0.62 (CH_2_Cl_2_); m.p. 210–213 °C; ${\left[\alpha \right]}_{D}^{25}$ +85.9 (c = 0.70, CHCl_3_); ^1^H NMR (400 MHz, CDCl_3_) δ 8.00–7.95 (m, 2H, Ph), 7.53–7.47 (m, 1H, Ph), 7.40–7.34 (m, 2H, Ph), 7.14–7.06 (m, 5H, Ph), 6.89–6.84 (m, 2H, Ph), 6.67–6.61 (m, 2H, Ph), 5.43 (dd, *J* = 9.0, 8.0 Hz, 1H, H_2_), 4.93 (d, *J* = 8.0 Hz, 1H, H_1_), 4.58 (d, *J* = 11.0 Hz, 1H, PhC*H*H), 4.53, (d, *J* = 10.9 Hz, 1H, PhCH*H*). 4.01 (dd, *J* = 10.4, 9.3 Hz, 1H, H_3_), 3.94–3.90 (m, 1H, H_6a_), 3.85–3.82 (m, 2H, H_5_, H_6b_), 3.65 (s, 3H, OCH_3_), 3.55 (t, *J* = 10.6 Hz, 1H, H_4_), 2.23 (s, 3H, SCOCH_3_), 1.08–0.91 (m, 21H, Si(C_3_H_7_)_3_); ^13^C NMR (100 MHz, CDCl_3_) δ 194.2 (SC=O), 165.1 (C=O), 155.4 (C_q_), 151.7 (C_q_), 137.5 (C_q_), 133.2 (CH), 129.9 (C_q_), 129.8 (CH), 128.4 (CH), 128.2 (CH), 128.0 (CH), 127.7 (CH), 118.9 (CH), 114.3 (CH), 100.8 (C_1_), 78.7 (C_3_), 76.2 (C_5_), 74.8 (C_2_), 74.4 (PhCH_2_), 63.6 (C_6_), 55.6 (OCH_3_), 45.9 (C_4_), 30.8 (CH_3_), 18.0 (Si(CH(*C*H_3_)_2_)_3_), 11.9 (Si(*C*H(CH_3_)_2_)_3_). HRMS (ESI^+^) *m*/*z* found (M+NH_4_)^+^ 712.3340; C_38_H_54_NO_8_SSi requires 712.3334.

*p*-Methoxyphenyl 2,3-di-*O*-benzoyl-4-thio-6-*O*-(triisopropylsilyl)-β-d-glucopyranoside (**11**)

A solution of *p*-methoxyphenyl 4-*S*-acetyl-2,3-di-*O*-benzoyl-6-*O*-(triisopropylsilyl)-β-d-glucopyranoside **9** (0.50 mg, 0.70 mmol, 1.00 equiv.) and NaHCO_3_ (6.0 mg, 0.70 mmol, 1.00 equiv.) in DMA (4.8 mL) was treated with DTT (0.22 g, 1.4 mmol, 2.00 equiv.) and left stirring at 33 °C for 1 h. A TLC analysis (CH_2_Cl_2_) revealed the full conversion of the starting material to a higher R_f_ spot. The mixture was dissolved in EtOAc (15 mL) and washed with brine (2 × 15 mL). The aqueous layer was separated and extracted with EtOAc (30 mL), and the combined organic layers were dried over anhydrous MgSO_4_, filtered, and concentrated *in vacuo* to produce a yellow syrup. The purification of this crude material via column chromatography (CH_2_Cl_2_) generated title compound **11** as a white solid (380 mg, 0.57 mmol, 81%). R_f_ = 0.70 (CH_2_Cl_2_); m.p. 129–131 °C; ${\left[\alpha \right]}_{D}^{25}$ +58.7 (c = 0.60, CHCl_3_); ^1^H NMR (400 MHz, DMSO- *d*^6^) δ 7.91–7.83 (m, 4H, Ph), 7.67–7.60 (m, 2H, Ph), 7.51–7.47 (m 4H, Ph), 6.93–6.88 (m, 2H, Ph), 6.82–6.77 (m, 2H, Ph), 5.68 (dd, *J* = 10.6, 9.5 Hz, 1H, H_3_), 5.45 (d, *J* = 8.0 Hz, 1H, H_1_), 5.30 (dd, *J* = 9.4, 8.0 Hz, 1H, H_2_), 4.23–4.21 (m, 1H, H_6a_), 4.04–3.95 (m, 2H, H_5,_ H_6b_), 3.68 (s, 3H, OCH_3_), 3.23 (br s, 1H, H_4_), 2.79 (br s, 1H, SH), 1.21–0.93 (m, 21H, Si(C_3_H_7_)_3_); ^13^C NMR (100 MHz, DMSO-*d*^6^) δ 165.6 (C=O), 165.2 (C=O), 155.4 (C_q_), 151.2 (C_q_), 134.2 (CH), 134.1 (CH), 129.7 (CH), 129.6 (CH), 129.5 (C_q_), 129.32 (CH), 129.30 (C_q_), 129.26 (CH), 118.6 (CH), 114.9 (CH), 99.4 (C_1_), 78.5 (C_5_), 76.1 (C_3_), 73.6 (C_2_), 55.8 (OCH_3_), 40.4 (C_4_), 18.30 (SiCH(*C*H_3_)_2_), 18.29 (SiCH(*C*H_3_)_2_), 11.9 (Si*C*H(CH_3_)_2_). HRMS (ESI^+^) *m*/*z* found (M+NH_4_)^+^ 684.3027; C_36_H_50_NO_8_SSi, requires 684.3021.

*p*-Methoxyphenyl 2-*O*-benzoyl-3-*O*-benzyl-4-thio-6-*O*-(triisopropylsilyl)-β-d-glucopyranoside (**12**)

A solution of *p*-methoxyphenyl 4-*S*-acetyl-2-*O*-benzoyl-3-*O*-benzyl-6-*O*-(triisopropylsilyl)-β-d-glucopyranoside **10** (2.58 g, 3.71 mmol, 1.00 equiv.) and NaHCO_3_ (312 mg, 3.71 mmol, 1.00 equiv.) in DMA (25 mL) was treated with DTT (1.14 g, 7.42 mmol, 2.00 equiv.) and left stirring at 33 °C for 1 h. A TLC analysis (CH_2_Cl_2_) revealed the full conversion of the starting material to a higher R_f_ spot. The mixture was diluted with EtOAc (25 mL) and washed with brine (2 × 20 mL). The aqueous layer was separated and extracted with EtOAc (30 mL), and the combined organic layers were dried over anhydrous MgSO_4_, filtered, and concentrated *in vacuo* to produce a yellow syrup. The purification of this crude material via column chromatography (CH_2_Cl_2_) generated title compound **12** as a colourless syrup (1.98 g, 3.03 mmol, 82%). R_f_ = 0.73 (CH_2_Cl_2_); ${\left[\alpha \right]}_{D}^{25}$ +12.3 (c = 2.30, CHCl_3_); ^1^H NMR (400 MHz, CDCl_3_) δ 8.08–8.03 (m, 2H, Ph), 7.61–7.55 (m, 1H, Ph), 7.48–7.42 (m, 2H, Ph), 7.25–7.17 (m, 5H, Ph), 6.95–6.90 (m, 2H, Ph), 6.75–6.69 (m, 2H, Ph), 5.46 (dd, *J* = 9.2, 8.0 Hz, 1H, H_2_), 4.97 (d, *J* = 8.0 Hz, 1H, H_1_), 4.77 (d, *J* = 10.5 Hz, 1H, PhCH*H*), 4.69 (d, *J* = 10.5 Hz, 1H, PhC*H*H), 4.14 (dd, *J* = 11.1, 2.0 Hz, 1H, H_6a_), 4.02 (dd, *J* = 11.1, 5.3 Hz, 1H, H_6b_), 3.73 (s, 3H, OCH_3_), 3.70 (dd, *J* = 10.3, 9.4 Hz, 1H, H_3_), 3.53 (ddd, *J* = 10.5, 5.3, 2.0 Hz, 1H, H_5_), 3.24 (td, *J* = 10.4, 5.9 Hz, 1H, H_4_), 1.91 (d, *J* = 5.9 Hz, 1H, SH), 1.18–1.04 (m, 21H, Si(C_3_H_7_)_3_); ^13^C NMR (100 MHz, CDCl_3_) δ 165.2 (C=O), 155.5 (C_q_), 151.6 (C_q_), 137.3 (C_q_), 133.2 (CH), 129.80 (CH), 129.78 (CH), 128.5 (CH), 128.4 (C_q_), 128.3 (CH), 127.9 (CH), 119.1 (CH), 114.3 (CH), 101.2 (C_1_), 83.6 (C_3_), 78.6 (C_5_), 74.6 (PhCH_2_), 74.4 (C_2_), 63.8 (C_6_), 55.6 (OCH_3_), 41.1 (C_4_), 17.99 (Si(CH(*C*H_3_)_2_)_3_), 17.98 (Si(CH(*C*H_3_)_2_)_3_), 11.9 (Si(*C*H(CH_3_)_2_)_3_). HRMS (ESI^+^) *m*/*z* found (M+Na)^+^ 675.2782; C_36_H_48_O_7_SSiNa requires 675.2793.

*p*-Methoxyphenyl 4-*S*-acetyl-2,3-di-*O*-benzoyl-β-d-glucopyranoside (**13**)

A solution of *p*-methoxyphenyl 4-*S*-acetyl-2,3-di-*O*-benzoyl-6-*O*-(triisopropylsilyl)-β-d-glucopyranoside **9** (1.80 g, 2.50 mmol, 1.00 equiv.) in MeCN/H_2_O (25 mL, 4/1) was treated with TFA (2.90 mL, 20.0 mmol, 8.00 equiv.). The reaction was stirred for 7 h at RT. A TLC analysis (8/2, hexane/EtOAc) revealed the full conversion of the starting material to two lower R_f_ spots. The reaction was quenched with NaHCO_3_ (30 mL) and extracted with CH_2_Cl_2_ (2 × 50 mL). The combined organic layers were washed with brine (50 mL), dried over anhydrous MgSO_4_, filtered, and concentrated *in vacuo* to produce a yellow syrup. The purification of this crude material via column chromatography (1/0 → 8/2, hexane/EtOAc) generated compound **13** as a white solid (0.93 g, 1.68 mmol, 66%), along with ester migration product **S12** (103 mg, 0.19 mmol, 7%). Characterisation data for **13**: R_f_ = 0.56 (7/3, hexane/EtOAc); m.p. 202–204 °C; ${\left[\alpha \right]}_{D}^{25}$ +86.5 (c = 0.60, CHCl_3_); ^1^H NMR (400 MHz, CDCl_3_) δ 7.96–7.90 (m, 4H, Ph), 7.54–7.46 (m, 2H, Ph), 7.40–7.33 (m, 4H, Ph), 6.93–6.88 (m, 2H, Ph), 6.81–6.74 (m, 2H, Ph), 5.77 (t, *J* = 10.0 Hz, 1H, H_3_), 5.63 (dd, *J* = 9.5, 7.9 Hz, 1H, H_2_), 5.22 (d, *J* = 7.9 Hz, 1H, H_1_), 3.97–3.93 (m, 3H, H_4_, H_5_, H_6a_), 3.88–3.79 (m, 1H, H_6b_), 3.74 (s, 1H, OCH_3_), 2.26 (s, 1H, SCOCH_3_); ^13^C NMR (100 MHz, CDCl_3_) δ 193.9 (C=O), 165.8 (C=O), 165.2 (C=O), 155.7 (C_q_), 151.0 (C_q_), 133.4 (CH), 133.2 (CH), 129.9 (CH), 129.8 (CH), 129.2 (C_q_), 128.9 (C_q_), 128.42 (CH), 128.38 (CH), 118.6 (CH), 114.6 (CH), 100.5 (C_1_), 75.5 (C_5_), 72.9 (C_2_), 71.4 (C_3_), 62.4 (C_6_), 55.6 (OCH_3_), 44.2 (C_4_), 30.8 (SCO*C*H_3_). HRMS (ESI^+^) *m*/*z* found (M+Na)^+^ 575.1353; C_29_H_28_O_9_SNa requires 575.1346.

Methyl (*p*-methoxyphenyl 4-*S*-acetyl-2,3-di-*O*-benzoyl-β-d-glucopyranosid)uronate (**14**)

A suspension of *p*-methoxyphenyl 4-*S*-acetyl-2,3-di-*O*-benzoyl-β-d-glucopyranoside **13** (602 mg, 1.09 mmol, 1.00 equiv.) in CH_2_Cl_2_/H_2_O (6 mL, 2/1) was treated with TEMPO (34.0 mg, 0.218 mmol, 0.20 equiv.) and BAIB (879 mg, 2.73 mmol, 2.50 equiv.) at 0 °C. The reaction was stirred at RT for 1 h. A TLC analysis (14/7/1, EtOAc/MeOH/H_2_O) of the resulting brown reaction mixture showed the complete conversion of the starting material to a lower R_f_ spot. The reaction was quenched with Na_2_S_2_O_3_ (5 mL) and diluted with EtOAc (10 mL). The aqueous layer was separated and acidified to pH = 2 using 1 M HCl and extracted with EtOAc (5 × 10 mL). The combined organic layers were washed with brine (50 mL), dried over anhydrous MgSO_4_, filtered, and concentrated *in vacuo* to give a yellow solid. A suspension of this crude material and K_2_CO_3_ (452 mg, 3.27 mmol, 3.00 equiv.) in DMF (3.7 mL) was treated with the dropwise addition of MeI (202 µL, 3.27 mmol, 3.00 equiv.) at 0 °C. The reaction was stirred at RT for 2 h, whereupon a TLC analysis (1/1, hexane/EtOAc) revealed the full conversion of the starting material to a higher R_f_ spot. The reaction mixture was diluted with CH_2_Cl_2_ (20 mL) and washed with H_2_O (30 mL). The aqueous layer was separated and extracted with CH_2_Cl_2_ (2 × 30 mL). The organic layers were combined and washed with brine (60 mL), dried over anhydrous MgSO_4_, filtered, and concentrated *in vacuo* to give a brown syrup. The purification of this crude material via column chromatography (100/0 → 98/2, CH_2_Cl_2_/Et_2_O) generated title compound **14** as a white solid (330 mg, 0.57 mmol, 52%). R_f_ = 0.14 (CH_2_Cl_2_); m.p. 155–157 °C; ${\left[\alpha \right]}_{D}^{25}$ +89.2 (c = 0.50, CHCl_3_); ^1^H NMR (400 MHz, CDCl_3_) δ 7.97–7.92 (m, 4H, Ph), 7.55–7.48 (m, 2H, Ph), 7.42–7.34 (m, 4H, Ph), 6.94–6.88 (m, 2H, Ph), 6.80–6.74 (m, 2H, Ph), 5.85 (dd, *J* = 10.9, 9.2 Hz, 1H, H_3_), 5.67 (dd, *J* = 9.2, 7.5 Hz, 1H, H_2_), 5.24 (d, *J* = 7.5 Hz, 1H, H_1_), 4.55 (d, *J* = 10.6 Hz, 1H, H_5_), 4.13 (t, *J* = 10.8 Hz, 1H, H_4_), 3.77 (s, 3H, OCH_3_), 3.74 (s, 3H, OCH_3_), 2.24 (s, 1H, SCOCH_3_); ^13^C NMR (100 MHz, CDCl_3_) δ 192.9 (SC=O), 167.2 (C=O), 165.6 (C=O), 165.1 (C=O), 155.8 (C_q_), 151.0 (C_q_), 133.5 (CH), 133.3 (CH), 130.0 (CH), 129.8 (CH), 129.1 (C_q_), 128.8 (C_q_), 128.44 (CH), 128.41 (CH), 118.9 (CH), 114.5 (CH) 100.9 (C_1_), 74.4 (C_5_), 72.6 (C_2_), 70.6 (C_3_), 55.6 (OCH_3_), 52.9 (SCO*C*H_3_), 44.9, (C_4_) 30.8 (OCH_3_). HRMS (ESI^+^) *m*/*z* found (M+NH_4_)^+^ 599.1776; C_30_H_32_NO_10_S requires 599.1774.

Methyl (*p*-methoxyphenyl 2,3-di-*O*-benzoyl-4-thio-β-d-glucopyranosid)uronate (**15**)

A solution of *p*-methoxyphenyl 2,3-di-*O*-benzoyl-4-*S*-acetyl-β-d-glucuronic methyl ester **14** (320 mg, 0.55 mmol, 1.00 equiv.) and NaHCO_3_ (46 mg, 0.55 mmol, 1.00 equiv.) in DMA (3.8 mL) was treated with DTT (170 mg, 1.1 mmol, 2.00 equiv.) and left stirring at 33 °C for 1.5 h. A TLC analysis (CH_2_Cl_2_) revealed almost a full conversion of the starting material to a higher R_f_ spot. The mixture was diluted with EtOAc (15 mL) and washed with brine (2 × 15 mL). The aqueous layer was separated and extracted with EtOAc (30 mL), and the combined organic layers were dried over anhydrous MgSO_4_ and concentrated *in vacuo* to produce a yellow syrup. The purification of this crude material via column chromatography (CH_2_Cl_2_) generated title compound **15** as a white solid (135 mg, 0.253 mmol, 46%). R_f_ = 0.67 (95/5, CH_2_Cl_2_/Et_2_O); m.p. 109–112 °C; ${\left[\alpha \right]}_{D}^{25}$ +39.6 (c = 0.81, CHCl_3_); ^1^H NMR (400 MHz, CDCl_3_) δ 8.02–7.97 (m, 2H, Ph), 7.95–7.91 (m, 2H, Ph), 7.56–7.48 (m, 2H, Ph), 7.43–7.34 (m, 4H, Ph), 6.93–6.88 (m, 2H, Ph), 6.79–6.74 (m, 2H, Ph), 5.63 (dd, *J* = 9.4, 7.6 Hz, 1H, H_2_), 5.53 (dd, *J* = 10.8, 9.5 Hz, 1H, H_3_), 5.20 (d, *J* = 7.6 Hz, 1H, H_1_), 4.18 (d, *J* = 10.6 Hz, 1H, H_5_), 3.85 (s, 3H, OCH_3_), 3.74 (s, 3H, OCH_3_), 3.54 (td, *J* = 10.7, 9.2 Hz, 1H, H_4_), 1.67 (d, *J* = 9.2 Hz, 1H, SH); ^13^C NMR (100 MHz, CDCl_3_) δ 167.3 (C=O), 165.9 (C=O), 165.1 (C=O), 155.9 (C_q_), 150.9 (C_q_), 133.5 (CH), 133.3 (CH), 133.0 (CH), 129.8 (CH), 129.1 (C_q_), 128.9 (C_q_), 128.5 (CH), 128.4 (CH), 118.9 (CH), 114.6 (CH), 101.4 (C_1_), 78.1 (C_5_), 74.2 (C_3_), 72.6 (C_2_), 55.6 (OCH_3_), 53.0 (OCH_3_), 40.9 (C_4_). HRMS (ESI^+^) *m*/*z* found (M+NH_4_)^+^ 556.1642; C_28_H_30_NO_9_S requires 556.1636.

Dibutyl-2-azido-3-*O*-benzyl-2-deoxy-4-*O*-levulinoyl-6-*O*-tert-butyldiphenylsilyl-α/β-d-glucopyranosyl phosphate (**18**)

A solution of *p*-methylphenyl 2-azido-3-*O*-benzyl-2-deoxy-4-*O*-levulinoyl-6-*O*-(tert-butyldiphenylsilyl)-1-thio-β-d-glucopyranoside **17** (502 mg, 0.680 mmol, 1.20 equiv.) in CH_2_Cl_2_ (16 mL) was stirred at RT for 1 h in the presence of 4 Å molecular sieves. The mixture was cooled to −45 °C (using MeCN and dry ice) and treated with NIS (184 mg, 0.820 mmol, 1.20 equiv.), TfOH (17.0 µL, 0.204 mmol, 0.30 equiv.), and P(O)(OBu)_2_OH (112 µL, 0.566 mmol, 1.0 equiv.) and allowed to warm to RT while stirring for 2.5 h. A TLC analysis (CH_2_Cl_2_) revealed the partial conversion of the starting material to a lower R_f_ spot. The reaction was brought to −10 °C (using NaCl in ice), and the reaction mixture was treated with supplementary additions of NIS (47.0 mg, 0.204 mmol, 0.300 equiv.) and TfOH (5.60 µL, 670 µmmol, 0.100 equiv.) to bring the reaction to completion. After stirring for 30 min, the reaction was quenched with NaHCO_3_ (10 mL), and the organic layer was separated and washed with Na_2_S_2_O_3_ (10 mL). The aqueous layer was extracted with CH_2_Cl_2_ (2 × 10 mL), and the combined organic layers were washed with brine (20 mL), dried over anhydrous MgSO_4_, filtered, and concentrated *in vacuo* to give a yellow oil. The purification of this crude material via column chromatography (9/1 → 8/2, hexane/EtOAc) yielded title compound **18** as a yellow syrup (298 mg, 0.362 mmol, 64%, α/β = 1.00:0.30); R_f_ = 0.70 (1/1, hexane/EtOAc).

α anomer

^1^H NMR (400 MHz, CDCl_3_) δ 7.68–7.60 (m, 4H, Ph), 7.44–7.28 (m, 11H, Ph), 5.77 (dd, *J* = 6.7, 3.3 Hz, 1H, H_1_), 5.43–5.37 (m, 1H, H_4_), 4.80 (d, *J* = 11.1 Hz, 1H, PhC*H*H), 4.68 (d, *J* = 11.1 Hz, 1H, PhCH*H*), 3.99–3.93 (m, 2H, H_3_, H_5_), 3.73–3.66 (m, 2H, H_6a_, H_6b_), 3.60 (dt, *J* = 10.1, 3.0 Hz, 1H, H_2_), 2.65–2.50 (m, 2H, Lev-CH_2_), 2.40–2.18 (m, 2H, Lev-CH_2_), 2.14 (s, 3H, Lev-CH_3_), 1.72–1.61 (m, 2H, Bu-CH_2_), 1.60–1.51 (m, 2H, Bu-CH_2_), 1.46–1.36 (m, 4H, 2 × Bu-CH_2_), 1.36–1.25 (m, 4H, 2 × Bu-CH_2_), 1.07–1.00 (m, 9H, SiC(CH_3_)_3_), 0.92 (td, *J* = 7.4, 3.2 Hz, 3H, Bu-CH_3_), 0.85 (td, *J* = 7.4, 5.4 Hz, 3H, Bu-CH_3_); ^13^C NMR (100 MHz, CDCl_3_) δ 206.0 (C=O), 171.0 (C=O), 137.5 (C_q_), 135.74 (CH), 135.70 (CH), 133.2 (C_q_), 133.1 (C_q_), 129.7 (CH), 129.6 (CH), 128.5 (CH), 128.2 (CH), 128.1 (CH), 128.0 (CH), 127.6 (CH), 95.6 (d, *J* = 5.9 Hz, C_1_), 77.8 (C_3_/C_5_), 75.0 (PhCH_2_), 72.4 (C_5_/C_3_), 69.5 (C_4_), 68.0 (d, *J* = 5.7 Hz, Bu-CH_2_), 67.8 (d, *J* = 5.8 Hz, Bu-CH_2_), 63.3 (d, *J* = 8.6 Hz, C_2_), 61.6 (C_6_), 37.8 (Lev-CH_2_), 32.23 (d, *J* = 3.5 Hz, Bu-CH_2_) 32.16 (d, *J* = 3.50, Bu-CH_2_), 29.8 (Lev-CH_3_), 27.8 (Lev-CH_2_), 26.7 (SiC(*C*H_3_)_3_), 19.3 Si*C*(CH_3_)_3_), 18.64 (Bu-CH_2_), 18.58 (Bu-CH_2_), 13.6 (Bu-CH_3_), 13.5 (Bu-CH_3_); ^31^P NMR (162 MHz, CDCl_3_) −2.28 (d, *J* = 1.5 Hz).

β anomer

^1^H NMR (400 MHz, CDCl_3_) selected signals δ 5.19–5.13 (m, 1H, H_4_), 5.08 (t, *J* = 7.7 Hz, 1H, H_1_), 4.79 (d, *J* = 11.2 Hz, 1H, PhC*H*H), 4.67 (d, *J* = 11.2 Hz, 1H, PhCH*H*), 4.20–3.99 (m, 2H, H_6a_, H_6b_), and 3.56–3.49 (m, 3H, H_2_, H_3_, H_5_); ^31^P NMR (162 MHz, CDCl_3_) δ −0.62. HRMS (ESI^+^) *m*/*z* found (M+NH_4_)^+^ 841.3973; C_42_H_62_N_4_O_10_PSi requires 841.3967.

3,4,6-Tri-*O*-acetyl-1,2-di-deoxy-2′-azidomethyl-α-d-glucopyrano-[2,1-d]-Δ2′-thiaoxazoline (**24**)

A stirred suspension of 2-acetamido-2-deoxy-1,3,4,6-tetra-*O*-acetyl-β-d-galactopyranose **23** (30.21 g, 77.59 mmol, 1.00 equiv.) and Lawesson’s reagent (26.67 g, 65.95 mmol, 0.85 equiv) in toluene (250 mL) was heated at 110 °C for 5 h. A TLC analysis (8/2, CH_2_Cl_2_/Et_2_O) revealed the full conversion of the starting material to a lower R_f_ spot. The red homogeneous reaction mixture was cooled to room temperature and diluted in CH_2_Cl_2_ (100 mL). The organic layer was washed with NaHCO_3_ (250 mL), and the aqueous layer was separated and extracted with CH_2_Cl_2_ (200 mL). The combined organic layers were washed with brine (300 mL), dried over anhydrous MgSO_4_, filtered, and concentrated *in vacuo* to yield a red/brownish syrup. The purification of this crude material via column chromatography (2/1 → 1/1, hexane/EtOAc) furnished title compound **24** as a red syrup (22.04 g, 63.70 mmol, 82%). R_f_ = 0.48 (8/2, CH_2_Cl_2_/Et_2_O); ^1^H NMR (400 MHz, CDCl_3_) δ 6.25 (d, *J* = 7.1 Hz, 1H, H_1_), 5.58 (dd, *J* = 3.3, 1.8 Hz, 1H, H_3_), 4.98–4.94 (m, 1H, H_4_), 4.48 (dddd, *J* = 7.0, 3.4, 2.3, 1.2 Hz, 1H, H_2_), 4.14–4.12 (m, 2H, H_6a_, H_6b_), 3.55 (dt, *J* = 9.1, 4.4 Hz, 1H, H_5_), 2.33 (s, 1H, CH_3_), 2.32 (s, 3H, CH_3_), 2.14 (s, 3H, CH_3_), 2.09 (d, *J* = 0.7 Hz, 3H, CH_3_); ^13^C NMR (100 MHz, CDCl_3_) δ 170.6 (N=C-S), 169.6 (C=O), 169.3 (C=O), 168.2 (C=O), 88.9 (C_1_), 76.7 (C_2_), 70.8 (C_3_), 69.3 (C_4_), 68.5 (C_5_), 63.3 (C_6_), 21.0 (CH_3_), 20.9 (CH_3_), 20.8 (CH_3_), 20.7 (CH_3_). HRMS (ESI^+^) *m*/*z* found (M+H)^+^ 346.0955; C_14_H_20_NO_7_S requires 346.0953. NMR data were consistent with those previously reported in the literature [41].

2-Acetamido-3,4,6-tri-*O*-acetyl-2-deoxy-1-thio-α-d-glucopyranose (**25**)

A solution 3,4,6-tri-*O*-acetyl-1,2-di-deoxy-2′-azidomethyl-α-d-glucopyrano-[2,1-d]-Δ2′-thiaoxazoline **24** (1.05 g, 2.47 mmol, 1.00 equiv.) was cooled to 0 °C and treated with TFA (1.02 mL, 12.3 mmol, 5.00 equiv.) and H_2_O (1.02 mL, 55.5 mmol, 22.5 equiv.). The reaction was stirred at RT for 1 h, and a ^1^H NMR spectroscopic analysis revealed the full conversion of **24** to title compound **25**. The reaction mixture was concentrated *in vacuo* and purified via column chromatography (1/0 → 8/2, CH_2_Cl_2_/Et_2_O) to furnish **25** as an off-white solid (620 mg, 1.71 mmol, 69%). R_f_ = 0.10 (8/2, CH_2_Cl_2_/Et_2_O); ^1^H NMR (400 MHz, CDCl_3_) δ 5.88 (d, *J* = 8.3 Hz, 1H, NH), 5.78 (dd, *J* = 7.1, 5.3 Hz, 1H, H_1_), 5.15 (t, *J* = 9.2 Hz, 1H, H_4_), 5.09 (t, *J* = 10.9 Hz, 1H, H_3_), 4.49 (ddd, *J* = 10.7, 8.5, 5.2 Hz, 1H, H_2_), 4.31 (ddd, *J* = 9.2, 4.2, 2.1 Hz, 1H, H_5_), 4.26 (dd, *J* = 12.2, 4.2 Hz, 1H, H_6a_), 4.12 (dd, *J* = 12.2, 2.1 Hz, 1H, H_6b_), 2.11 (s, 3H, CH_3_), 2.06 (s, 3H, CH_3_), 2.06 (s, 3H, CH_3_), 2.03 (d, *J* = 7.2 Hz, 1H, SH), 2.00 (s, 3H, CH_3_); ^13^C NMR (100 MHz, CDCl_3_) δ 171.9 (C=O), 170.8 (C=O), 170.6 (C=O), 169.3 (C=O), 78.8 (C_1_), 70.7 (C_3_), 69.0 (C_5_), 67.8 (C_4_), 61.8 (C_6_), 52.7 (C_2_), 23.1 (CH_3_), 20.75 (CH_3_), 20.73 (CH_3_), 20.6 (CH_3_). HRMS (ESI^+^) *m*/*z* found (M+Na)^+^ 386.0885; C_14_H_21_NO_8_SNa requires 386.0880. NMR data were consistent with those previously reported in the literature [41].

*p*-Monomethoxytrityl 2-acetamido-3,4,6-tetra-*O*-acetyl-2-deoxy-1-thio-α-d-glucopyranoside (**26**)

A solution of 2-acetamido-3,4,6-tri-*O*-acetyl-2-deoxy-1-thio-α-d-glucopyranose **25** (2.81 g, 7.73 mmol, 1.00 equiv.) in pyridine (35 mL) was treated with MMTrCl (2.62 g, 8.56 mmol, 1.10 equiv.) and left to stir overnight. A TLC analysis (7/3, CH_2_Cl_2_/Et_2_O) revealed the full conversion of the starting material to a higher R_f_ spot. The reaction mixture was concentrated *in vacuo* to give a brown syrup and purified via column chromatography (100/0 → 99/1, CH_2_Cl_2_/Et_2_O) to generate title compound **26** as a white solid (2.13 g, 3.35 mmol, 42%). R_f_ = 0.39 (7/3, CH_2_Cl_2_/Et_2_O); m.p. 230–222 °C (decomposed); ${\left[\alpha \right]}_{D}^{25}$ +219.0 (c = 1.0, CHCl_3_); ^1^H NMR (400 MHz, CDCl_3_) δ 7.34–7.24 (m, 10H, Ph), 7.24–7.19 (m, 2H, Ph), 6.82–6.77 (m, 2H, Ph), 5.60 (d, *J* = 9.7 Hz, 1H, NH), 5.16–5.08 (m, 1H, H_4_), 5.01 (dd, *J* = 11.2, 9.2 Hz, 1H, H_3_), 4.82 (d, *J* = 5.4 Hz, 1H, H_1_), 4.45 (ddd, *J* = 11.1, 9.8, 5.3 Hz, 1H, H_2_), 4.40–4.44 (m, 1H, H_5_), 4.27 (dd, *J* = 12.4, 3.3 Hz, 1H, H_6a_), 4.01 (dd, *J* = 12.4, 2.4 Hz, 1H, H_6b_), 3.80 (s, 3H, OCH_3_), 2.10 (s, 3H, CH_3_), 2.02 (s, 3H, CH_3_), 1.99 (s, 3H, CH_3_), 1.93 (s, 3H, CH_3_); ^13^C NMR (100 MHz, CDCl_3_) δ 171.2 (C=O), 170.8 (C=O), 169.2 (C=O), 169.1 (C=O), 158.6 (C_q_), 144.44 (C_q_), 144.35 (C_q_), 136.0 (C_q_), 131.1 (CH), 129.8 (CH), 128.0 (CH), 127.3 (CH), 113.3 (CH), 84.2 (C_1_), 71.9 (C_3_), 70.0 (C_5_), 69.9 (C_q_), 68.0 (C_4_), 61.9 (C_6_), 55.3 (OCH_3_), 51.9 (C_2_), 23.3 (CH_3_), 20.8 (CH_3_), 20.7 (CH_3_), 20.6 (CH_3_). HRMS (ESI^+^) *m*/*z* found (M+NH_4_)^+^ 635.2524; C_34_H_41_N_2_O_9_S requires 635.2527.

*p*-Monomethoxytrityl 2-acetamido-4,6-*O*-benzylidene-2-deoxy-1-thio-α-d-glucopyranoside (**27**)

A suspension of *p*-monomethoxytrityl 2-acetamido-3,4,6-tetra-*O*-acetyl-2-deoxy-1-thio-α-d-glucopyranoside **26** (3.13 g, 4.92 mmol, 1.00 equiv.) in MeOH (20 mL) was treated with Na_2_CO_3_ (114 mg, 1.08 mol, 0.22 equiv.) and left to stir at RT for 3 h. A TLC analysis (7/3, CH_2_Cl_2_/Et_2_O) revealed the full conversion of the starting material to a lower R_f_ spot. The now colourless reaction mixture was neutralised via the addition of Amberlite^TM^ IRC120 H resin, filtered, and concentrated *in vacuo* to generate the crude deacetylated product as a white solid (2.50 g, 4.91 mmol). It was used in the next step without further purification. HRMS (ESI^−^) *m*/*z* [found: (M-H)^−^ 508.1804, C_28_H_29_NO_6_S requires 508.1799]. A solution of the crude material (3.51 g, 6.89 mmol, 1.00 equiv.) in MeCN/DMF (23 mL, 3.5/1) was treated with benzaldehyde dimethyl acetal (1.48 mL, 9.84 mmol, 2.00 equiv.) and CSA (240 mg, 1.03 mmol, 0.15 equiv.) and left stirring at RT for 5 h. A TLC analysis (1/1, CH_2_Cl_2_/Et_2_O) revealed an almost full conversion of the starting material to a higher Rf spot. The reaction mixture was washed with NaHCO_3_ (15 mL) and diluted with CH_2_Cl_2_ (20 mL). The aqueous layer was separated and extracted with CH_2_Cl_2_ (2 × 20 mL), and the combined organic layers were washed with brine (50 mL), dried over anhydrous MgSO_4_, filtered, and concentrated *in vacuo* to generate a yellow syrup. The purification of this crude material via column chromatography (100/0 → 85/15, Et_2_O/MeOH) furnished title compound **27** as a white solid (2.35 g, 3.93 mmol, 80%). R_f_ = 0.28 (1/1, CH_2_Cl_2_/Et_2_O); m.p. 170–171 °C; ${\left[\alpha \right]}_{D}^{25}$ +183.3 (c = 2.25, CHCl_3_); ^1^H NMR (400 MHz, CDCl_3_) δ 7.53–7.50 (m, 2H, Ph), 7.40–7.35 (m, 7H, Ph), 7.33–7.22 (m, 8H, Ph), 6.84–6.78 (m, 2H, Ph), 5.72 (d, *J* = 9.0 Hz, 1H, NH), 5.55 (s, 1H, PhCH), 4.67 (d, *J* = 5.6 Hz, 1H, H_1_), 4.38 (dd, *J* = 9.8, 4.8 Hz, 1H, H_5_), 4.32 (ddd, *J* = 10.8, 9.0, 5.5 Hz, 1H, H_2_), 4.25 (dd, *J* = 10.2, 4.9 Hz, 1H, H_6a_), 3.80 (s, 3H, OCH_3_), 3.83–3.73 (m, 2H, H_3_, H_6b_), 3.52 (t, *J* = 9.2 Hz, 1H, H_4_), 1.83 (s, 3H, CH_3_); ^13^C NMR (100 MHz, CDCl_3_) δ 170.7 (C=O), 158.5 (C_q_), 144.5 (C_q_), 144.4 (C_q_), 137.0 (C_q_), 136.1 (C_q_), 131.1 (CH), 129.71 (CH), 129.68 (CH), 129.3 (CH), 128.3 (CH), 128.01 (CH), 127.99 (CH), 127.26 (CH), 127.25 (CH), 126.4 (CH), 113.3 (CH), 102.1 (PhCH), 84.9 (C_1_), 82.5 (C_4_), 71.4 (C_3_), 69.8 (C_q_), 68.7 (C_6_), 65.0 (C_5_), 55.3 (OCH_3_), 53.9 (C_2_), 23.4 (CH_3_). HRMS (ESI^+^) *m*/*z* found (M+Na)^+^ 620.2081; C_35_H_35_NO_6_SNa requires 620.2077.

*p*-Monomethoxytrityl-2-acetamido-3-*O*-benzoyl-4,6-*O*-benzylidene-2-deoxy-1-thio-α-d-glucopyranoside (**28**)

A solution of *p*-monomethoxytrityl 2-acetamido-4,6-*O*-benzylidene-2-deoxy-1-thio-α-d-glucopyranoside **27** (3.64 g, 6.09 mmol, 1.00 equiv.) in pyridine (40 mL) was treated with DMAP (75.0 mg, 0.61 mmol, 0.10 equiv.), followed by the addition of BzCl (1.41 mL, 12.2 mmol, 2.00 equiv.) at 0 °C. The reaction was left stirring at RT for 1 h, whereupon a TLC analysis (1/1, CH_2_Cl_2_/Et_2_O) revealed the full conversion of the starting material to a higher R_f_ spot. The reaction was diluted with CH_2_Cl_2_ (25 mL) and washed with NaHCO_3_ (30 mL). The aqueous layer was separated and extracted with CH_2_Cl_2_ (2 × 30 mL), and the combined organic layers were washed with brine (50 mL), dried over anhydrous MgSO_4_, filtered, and concentrated *in vacuo* to give a yellow syrup. This crude material was dissolved in hot MeOH (10 mL) and left to cool slowly to 0 °C, furnishing a white solid that was collected via filtration. The filtrate was concentrated *in vacuo* and purified via column chromatography (100/0 → 95/5, CH_2_Cl_2_/Et_2_O) to furnish another crop of material (390 mg). The overall yield of title compound **28** was (3.37 g, 4.80 mmol, 79%). R_f_ = 0.54 (9/1, CH_2_Cl_2_/Et_2_O); m.p. 209–211 °C; ${\left[\alpha \right]}_{D}^{25}$ +137.0 (c = 1.0, CHCl_3_); ^1^H NMR (400 MHz, CDCl_3_) δ 8.12–8.07 (m, 1H, Ph), 8.00–7.94 (m, 2H, Ph), 7.63–7.26 (m, 20H, Ph), 6.81–6.79 (m, 1H, Ph), 5.85 (d, *J* = 9.7 Hz, 1H, NH), 5.53 (s, 1H, PhCH), 5.41 (dd, *J* = 11.1, 9.4 Hz, 1H, H_3_), 4.83 (d, *J* = 5.5 Hz, 1H, H_1_), 4.61 (ddd, *J* = 11.0, 9.7, 5.5 Hz, 1H, H_2_), 4.47 (td, *J* = 9.9, 4.8 Hz, 1H, H_5_), 4.21 (dd, *J* = 10.5, 4.8 Hz, 1H, H_6a_), 3.80 (s, 1H, OCH_3_), 3.84–3.73 (m, 2H, H_4_, H_6b_), 1.83 (s, 3H, CH_3_); ^13^C NMR (100 MHz, CDCl_3_) δ 169.3 (C=O), 166.9 (C=O), 158.5 (C_q_), 144.49 (C_q_), 144.45 (C_q_), 136.9 (C_q_), 136.2 (C_q_), 133.6 (CH), 133.3 (CH), 131.0 (CH), 130.2 (CH), 129.9 (CH), 129.8 (CH), 129.3 (C_q_), 129.1 (CH), 128.5 (CH), 128.4 (CH), 128.2 (CH), 127.9 (CH), 127.2 (CH), 126.2 (CH), 113.2 (CH), 101.6 (PhCH), 85.3 (C_1_), 79.5 (C_4_), 71.2 (C_3_), 69.5 (C_q_), 68.8 (C_6_), 65.3 (C_5_), 55.3 (OCH_3_), 52.8 (C_2_), 23.2 (CH_3_). HRMS (ESI^+^) *m*/*z* found (M+NH_4_)^+^ 719.2790; C_42_H_43_N_2_O_7_S requires 719.2785.

*p*-Monomethoxytrityl 2-acetamido-3-*O*-benzoyl-2-deoxy-1-thio-α-d-glucopyranoside (**29**)

A suspension of *p*-monomethoxytrityl 2-acetamido-3-*O*-benzoyl-4,6-*O*-benzylidene-2-deoxy-1-thio-α-d-glucopyranoside **28** (3.20 g, 4.56 mmol, 1.00 equiv.) in MeOH (45 mL) was treated with TsOH·H_2_O (86.7 mg, 0.456 mmol, 0.10 equiv.) and stirred at reflux for 8 h. A TLC analysis (95/5, CH_2_Cl_2_/Et_2_O) revealed the full conversion of the starting material to a lower R_f_ spot. The reaction was diluted with CH_2_Cl_2_ (30 mL) and washed with NaHCO_3_ (25 mL). The aqueous layer was separated and extracted with CH_2_Cl_2_ (2 × 30 mL), and the combined organic layers were washed with brine (40 mL), dried over anhydrous MgSO_4_, filtered, and concentrated *in vacuo* to generate a white solid. The purification of this crude material via a short silica plug (1/0 → 7/3, CH_2_Cl_2_/MeOH) furnished title compound **29** as a white solid (2.53 g, 4.12 mmol, 90%). R_f_ = 0.10 (9/1, CH_2_Cl_2_/Et_2_O); m.p. 151–153 °C; ${\left[\alpha \right]}_{D}^{25}$ +187.6 (c = 1.0, CHCl_3_); ^1^H NMR (400 MHz, CDCl_3_) δ 8.02–7.97 (m, 2H, Ph), 7.64–7.59 (m, 1H, Ph), 7.41–7.44 (m, 2H, Ph), 7.33–7.22 (m, 12H, Ph), 6.84–6.78 (m, 2H, Ph), 5.75 (d, *J* = 9.7 Hz, 1H, NH), 5.13 (dd, *J* = 11.3, 9.0 Hz, 1H, H_3_), 4.82 (d, *J* = 5.3 Hz, 1H, H_1_), 4.51 (ddd, *J* = 11.3, 9.8, 5.4 Hz, 1H, H_2_), 4.25 (dt, *J* = 9.5, 3.7 Hz, 1H, H_5_), 3.89–3.81 (m, 3H, H_4_, H_6a,_ H_6b_), 3.80 (s, 3H, OCH_3_), 1.82 (s, 3H, CH_3_). ^13^C NMR (100 MHz, CDCl_3_) δ 169.3 (C=O), 167.8 (C=O), 158.5 (C_q_), 144.6 (C_q_), 144.5 (C_q_), 136.2 (C_q_), 133.7 (CH), 131.1 (CH), 130.0 (CH), 129.8 (CH), 129.0 (C_q_), 128.6 (CH), 128.0 (CH), 127.2 (CH), 113.2 (CH), 84.3 (C_1_), 75.6 (C_3_), 73.9 (C_5_), 69.6 (C_4_, C_q_), 62.3 (C_6_), 55.3 (OCH_3_), 51.9 (C_2_), 23.3 (CH_3_). HRMS (ESI^−^) *m*/*z* found (M-H)^−^ 612.2048; C_35_H_34_NO_7_S requires 612.2061.

*p*-Monomethoxytrityl 2-acetamido-3,6-di-*O*-benzoyl-2-deoxy-1-thio-α-d-glucopyranoside (**30**)

A solution of *p*-monomethoxytrityl 2-acetamido-3-*O*-benzoyl-2-deoxy-1-thio-α-d-glucopyranoside **29** (0.990 g, 1.61 mmol, 1.00 equiv.) in CH_2_Cl_2_ (12 mL) was cooled to −30 °C, treated with the dropwise addition of BzCl (0.220 mL, 1.93 mmol, 1.20 equiv.), and left to stir at −30 °C for 1 h. A TLC analysis (9/1, CH_2_Cl_2_/Et_2_O) revealed the full conversion of the starting material to a higher R_f_ spot. The reaction was diluted with CH_2_Cl_2_ (15 mL) and washed with NaHCO_3_ (15 mL). The aqueous layer was separated and extracted with CH_2_Cl_2_ (2 × 15 mL), and the combined organic layers were washed with brine (25 mL), dried over anhydrous MgSO_4_, filtered, and concentrated *in vacuo* to yield an off-white solid. The purification of this crude material via column chromatography (100/0 → 95/15, CH_2_Cl_2_/Et_2_O) yielded title compound **30** as a white solid (0.952 g, 1.32 mmol, 82%). R_f_ = 0.43 (9/1, CH_2_Cl_2_/Et_2_O); ${\left[\alpha \right]}_{D}^{25}$ +179.0 (c = 1.0, CHCl_3_); ^1^H NMR (400 MHz, CDCl_3_) δ 8.08–8.05 (m, 2H, Ph), 8.00–7.96 (m, 2H, Ph), 7.61–7.51 (m, 3H, Ph), 7.48–7.34 (m, 10H, Ph), 7.31–7.21 (m, 5H, Ph), 6.83–6.75 (m, 2H, Ph), 5.76 (d, *J* = 9.8 Hz, 1H, NH), 5.19 (dd, *J* = 11.3, 9.1 Hz, 1H, H_3_), 4.89 (dd, *J* = 12.0, 4.0 Hz, 1H, H_6a_), 4.87 (d, *J* = 5.1 Hz, 1H, H_1_), 4.55 (ddd, *J* = 11.2, 9.9, 5.4 Hz, 1H, H_2_), 4.49–4.48 (m, 1H, H_5_), 4.37 (dd, *J* = 12.3, 2.1 Hz, 1H, H_6b_), 3.82 (t, *J* = 9.5 Hz, 1H, H_4_), 3.77 (s, 3H, OCH_3_), 1.81 (s, 3H, CH_3_). ^13^C NMR (100 MHz, CDCl_3_) δ 169.5 (C=O), 167.6 (C=O), 167.2 (C=O), 158.5 (C_q_), 144.60 (C_q_), 14455 (C_q_), 136.2 (C_q_), 133.5 (CH), 133.4 (CH), 131.1 (CH), 130.0 (CH), 129.9 (CH), 129.83 (CH), 129.81 (CH), 129.5 (CH), 129.1 (C_q_), 128.47 (C_q_), 128.45 (CH), 128.3 (CH), 128.0 (CH), 127.23 (CH), 127.22 (CH), 113.2 (CH), 84.5 (C_1_), 75.0 (C_3_), 72.8 (C_5_), 69.7 (C_q_), 68.8 (C_4_), 63.4 (C_6_), 55.2 (OCH_3_), 52.0 (C_2_), 23.3 (CH_3_). HRMS (ESI^+^) *m*/*z* found (M+Na)^+^ 740.2294; C_42_H_39_NO_8_SNa requires 720.2289.

*p*-Monomethoxytrityl 2-acetamido-3,6-di-*O*-benzoyl-2-deoxy-4-*O*-levulinoyl-1-thio-α-d-glucopyranoside (**32**)

A solution of *p*-monomethoxytrityl 2-acetamido-3,6-*O*-dibenzoyl-2-deoxy-1-thio-α-d-glucopyranoside **30** (855 mg, 1.00 mmol, 1.00 equiv.) in CH_2_Cl_2_ (2 mL) was treated with levulinic acid (163 mL, 1.60 mmol, 1.60 equiv.), EDCI·HCl (248 mg, 1.60 mmol, 1.60 equiv.), DMAP (195 mg, 1.60 mmol, 1.60 equiv.), and DIPEA (279 µL, 1.60 mmol, 1.60 equiv.). The reaction was stirred at RT for 2 h, whereupon a TLC analysis (9/1, CH_2_Cl_2_/Et_2_O) revealed the full conversion of the starting material to a higher R_f_ spot. The reaction was diluted in CH_2_Cl_2_ (20 mL) and washed with NaHCO_3_ (15 mL). The aqueous layer was separated and extracted with CH_2_Cl_2_ (2 × 15 mL). The combined organic layers were washed with brine (20 mL), dried over anhydrous MgSO_4_, filtered, and concentrated *in vacuo* to yield a yellow syrup. The purification of this crude material via column chromatography (1/0 → 8/2, CH_2_Cl_2_/Et_2_O) generated title compound **32** as a white solid (649 mg, 0.684 mmol, 68%). R_f_ = 0.33 (9:1, CH_2_Cl_2_/Et_2_O); m.p. 81–82 °C; ${\left[\alpha \right]}_{D}^{23}$ +35.0 (c = 0.80, CHCl_3_); ^1^H NMR (400 MHz, CDCl_3_) δ 8.11–8.04 (m, 2H, Ph), 7.98–7.91 (m, 2H, Ph), 7.59–7.52 (m, 2H, Ph), 7.45–7.42 (m, 4H, Ph), 7.38–7.32 (m, 4H, Ph), 7.31–7.21 (m, 9H, Ph), 6.83–6.75 (m, 1H, Ph), 5.82 (d, *J* = 9.6 Hz, 1H, NH), 5.45 (t, *J* = 9.7 Hz, 1H, H_3_), 5.33 (dd, *J* = 11.4, 9.5 Hz, 1H, H_4_), 4.95 (d, *J* = 5.3 Hz, 1H, H_1_), 4.65–4.58 (m, 2H, H_2_, H_5_), 4.46 (dd, *J* = 12.5, 2.9 Hz, 1H, H_6a_), 4.41 (dd, *J* = 12.5, 2.5 Hz, 1H, H_6b_), 3.77 (s, 1H, OCH_3_), 2.57–2.51 (m, 2H, Lev-CH_2_), 2.44–2.33 (m, 2H, Lev-CH_2_), 1.93 (s, 3H, Lev-CH_3_), 1.83 (s, 3H, CH_3_); ^13^C NMR (100 MHz, CDCl_3_) δ 205.7 (C=O), 171.3 (C=O), 169.2 (C=O), 167.0 (C=O), 166.2 (C=O), 158.6 (C_q_), 144.49 (C_q_), 144.47 (C_q_), 136.1 (C_q_), 133.5 (CH), 133.10 (CH), 131.13 (CH), 130.0 (CH), 129.84 (CH), 129.83 (CH), 129.80 (CH), 128.8 (C_q_), 128.5 (CH), 128.4 (CH), 128.0 (CH), 127.30 (CH), 127.29 (CH), 113.3 (CH), 84.4 (C_1_), 72.4 (C_4_), 70.1 (C_q_), 69.8 (C_5_), 68.3 (C_3_), 62.4 (C_6_), 55.3 (OCH_3_), 52.5 (C_2_), 37.9 (Lev-CH_2_), 29.4 (Lev-CH_3_), 27.8 (Lev-CH_2_), 23.3 (CH_3_). HRMS (ESI^+^) *m*/*z* found (M+Na)^+^ 838.2665; C_47_H_45_NO_10_SNa requires 838.2656.

*p*-Monomethoxytrityl 2-acetamido-3-*O*-benzoyl-2-deoxy-4-*O*-levulinoyl-6-*O*-(*tert*-butyldiphenylsilyl)-1-thio-α-d-glucopyranoside (**33**)

A solution of *p*-monomethoxytrityl 2-acetamido-3-*O*-benzoyl-2-deoxy-1-thio-α-d-glucopyranoside **29** (4.02 g, 6.55 mmol, 1.00 equiv.) and imidazole (1.34 g, 19.7 mmol, 3.00 equiv.) in DMF (15 mL) was treated with TBDPSCl (2.55 mL, 9.83 mmol, 3.00 equiv.), and the reaction was left stirring at RT for 1 h. A TLC analysis (9/1, CH_2_Cl_2_/Et_2_O) revealed the full conversion of the starting material to a higher R_f_ spot. The reaction was diluted in CH_2_Cl_2_ (20 mL) and washed with H_2_O (25 mL). The aqueous layer was separated and extracted with CH_2_Cl_2_ (3 × 20 mL), and the combined organic layers were washed with brine (30 mL), dried over anhydrous MgSO_4_, filtered, and concentrated *in vacuo* to yield a colourless syrup. A solution of this crude material, DMAP (1.28 g, 10.5 mmol, 1.60 equiv.), and EDCI·HCl (1.63 g, 10.5 mmol, 1.60 equiv.) in CH_2_Cl_2_ (32 mL) was treated with DIPEA (1.92 mL, 10.5 mmol, 1.60 equiv.) and levulinic acid (4.53 mL, 10.5 mmol, 1.60 equiv.). The reaction was stirred for 4 h, whereupon a TLC analysis (95/5, CH_2_Cl_2_/Et_2_O) revealed an almost full conversion of the starting material to a higher R_f_ spot. The reaction was quenched with H_2_O (20 mL), and the aqueous layer was separated and extracted with CH_2_Cl_2_ (3 × 20 mL). The combined organic layers were washed with brine (30 mL), dried over anhydrous MgSO_4_, and concentrated *in vacuo* to give a yellow syrup. This crude material was dissolved in hot MeOH (10 mL) and solidified slowly at 0 °C. The solid was collected via filtration to generate title compound **33** as a white solid (4.06 g, 4.27 mmol, 65%). R_f_ = 0.62 (95/5, CH_2_Cl_2_/Et_2_O); m.p. 117–119 °C; ${\left[\alpha \right]}_{D}^{23}$ +90.8 (c = 1.35, CHCl_3_); ^1^H NMR (400 MHz, CDCl_3_) δ 8.00–7.96 (m, 2H, Ph), 7.73–7.64 (m, 4H, Ph), 7.57–7.52 (m, 1H, Ph), 7.44–7.32 (m, 8H, Ph), 7.31–7.27 (m, 4H, Ph), 7.22–7.15 (m, 8H, Ph), 6.73–6.67 (m, 2H, Ph), 5.81 (d, *J* = 9.6 Hz, 1H, NH), 5.65 (t, *J* = 9.7 Hz, 1H, H_4_), 5.28 (dd, *J* = 11.4, 9.4 Hz, 1H, H_3_), 4.98 (d, *J* = 5.3 Hz, 1H, H_1_), 4.60 (ddd, *J* = 11.4, 9.6, 5.3 Hz, 1H, H_2_), 4.32 (dt, *J* = 10.0, 2.0 Hz, 1H, H_5_), 3.77 (m, 2H, H_6a_, H_6b_), 3.71 (s, 3H, OCH_3_), 2.54–2.48 (m, 2H, Lev-CH_2_), 2.42–2.28 (m, 2H, Lev-CH_2_), 1.96 (Lev-CH_3_), 1.06 (s, 9H, SiC(CH_3_)_3_). ^13^C NMR (100 MHz, CDCl_3_) δ 205.7 (C=O), 170.9 (C=O), 169.15 (C=O), 167.16 (C=O), 158.4 (C_q_), 144.64 (C_q_), 144.63 (C_q_), 136.4 (C_q_), 135.8 (CH), 135.7 (CH), 133.42 (CH), 133.39 (CH), 133.05 (CH), 131.13 (CH), 130.1 (CH), 129.89 (CH), 129.85 (CH), 129.7 (CH), 129.6 (CH), 129.0 (C_q_), 128.5 (CH), 127.9 (CH), 127.68 (CH), 127.67 (CH), 127.17 (CH), 127.15 (C_q_), 113.2 (CH), 84.4 (C_1_), 73.1 (C_3_), 72.4 (C_5_), 69.6 (C_q_), 67.9 (C_4_), 61.9 (C_6_), 55.2 (OCH_3_), 52.6 (C_2_), 38.0 (Lev-CH_2_), 29.5 (Lev-CH_3_), 27.9 (Lev-CH_2_), 26.8 (SiC(*C*H_3_)_3_), 23.4 (CH_3_), 19.3 (Si*C*(CH_3_)_3_). HRMS (ESI^+^) *m*/*z* found: (M+Na)^+^ 972.3583; C_56_H_59_NO_9_SSiNa requires 972.3572.

2-Acetamido-3,6-di-*O*-benzoyl-2-deoxy-4-*O*-levulinoyl-1-thio-α-d-glucopyranose (**34**)

A solution of *p*-monomethoxytrityl 2-acetamido-3,6-*O*-dibenzoyl-4-*O*-levulinoyl-2-deoxy-1-thio-α-d-glucopyranoside **32** (234 mg, 0.29 mmol, 1.00 equiv.) in CH_2_Cl_2_ (29 mL) was cooled to 0 °C and treated with the dropwise addition of TFA (219 µL, 2.87 mmol, 10.0 equiv.), followed by the addition of Et_3_SiH (311 µL, 1.95 mmol, 6.80 equiv.). The reaction was stirred at 0 °C for 1 h and a further 3 h at RT. A TLC analysis (9/1, CH_2_Cl_2_/Et_2_O) revealed the full conversion of the starting material to a lower R_f_ spot. The reaction mixture was concentrated *in vacuo* to generate a yellow residue. The purification of this crude material via column chromatography (1/0 → 8/2, CH_2_Cl_2_/Et_2_O) furnished the title compound **34** as a white solid (98.0 mg, 0.18 mmol, 62%). R_f_ = 0.51 (CH_2_Cl_2_/Et_2_O); m.p. 67–70 °C; ${\left[\alpha \right]}_{D}^{23}$ +22.9 (c = 0.56, CHCl_3_); ^1^H NMR (400 MHz, CDCl_3_) δ 8.09–8.07 (m, 2H, Ph), 8.01–7.97 (m, 2H, Ph), 7.58 (m, 2H, Ph), 7.51–7.42 (m, 4H, Ph), 6.04 (d, *J* = 8.1 Hz, 1H, NH), 5.89 (dd, *J* = 6.8, 5.3 Hz, 1H, H_1_), 5.46 (t, *J* = 9.6 Hz, 1H, H_4_), 5.43–5.36 (m, 1H, H_3_), 4.64–4.59 (m, 1H, H_2_), 4.58–4.53 (m, 2H, H_5_, H_6a_), 4.47 (dd, *J* = 12.5, 4.5 Hz, 1H, H_6b_), 2.60 (dd, *J* = 9.4, 4.7 Hz, 2H, Lev-CH_2_), 2.52–2.36 (m, 2H, Lev-CH_2_), 2.05 (d, *J* = 6.9 Hz, 1H, SH), 1.95 (s, 3H, Lev-CH_3_), 1.90 (s, 3H, CH_3_); ^13^C NMR (100 MHz, CDCl_3_) δ 205.8 (C=O), 171.6 (C=O), 170.3 (C=O), 167.6 (C=O), 166.3 (C=O), 133.9 (CH), 133.3 (CH), 130.2 (CH), 129.9 (C_q_), 128.8 (C_q_), 128.7 (CH), 128.6 (CH), 123.0 (CH), 79.2 (C_1_), 71.5 (C_3_), 69.4 (C_5_), 68.3 (C_4_), 62.4 (C_6_), 53.3 (C_2_), 38.0 (Lev-CH_2_), 29.5 (Lev-CH_3_), 28.0 (Lev-CH_2_), 23.3 (CH_3_). HRMS (ESI^+^) *m*/*z* found (M+H)^+^ 544.1632; C_27_H_30_NO_9_S requires 544.1636.

2-Acetamido-3-*O*-benzoyl-2-deoxy-4-*O*-levulinoyl-6-*O*-(*tert*-butyldiphenylsilyl)-1-thio-α-d-glucopyranose (**35**)

A solution of *p*-monomethoxytrityl 2-acetamido-3-*O*-benzoyl-2-deoxy-4-*O*-levulinoyl-6-*O*-(*tert*-butyldiphenylsilyl)-1-thio-α-d-glucopyranoside **33** (3.87 g, 4.07 mmol, 1.00 equiv.) in CH_2_Cl_2_ (180 mL) was cooled to 0 °C and treated via the dropwise addition of TFA (3.11 mL, 40.7 mmol, 10.00 equiv.), followed by the addition of Et_3_SiH (4.12 mL, 27.7 mmol, 6.80 equiv.). The reaction was stirred at 0 °C for 1 h and a further 3 h at RT. A TLC analysis (9/1, CH_2_Cl_2_/Et_2_O) revealed the full conversion of the starting material to a lower R_f_ spot. The reaction mixture was concentrated *in vacuo* to generate a yellow residue. The purification of this crude material via column chromatography (1/0 → 8/2, CH_2_Cl_2_/Et_2_O) furnished title compound **35** as a white solid (2.51 g, 3.70 mmol, 91%). R_f_ = 0.56 (95/5, CH_2_Cl_2_/Et_2_O); m.p. 77–79 °C; ${\left[\alpha \right]}_{D}^{23}$ +75.0 (c = 0.50, CHCl_3_); ^1^H NMR (400 MHz, CDCl_3_) δ 8.02–7.96 (m, 2H, Ph), 7.70–7.66 (m, 4H, Ph), 7.63–7.54 (m, 1H, Ph), 7.48–7.35 (m, 8H, Ph), 6.09 (d, *J* = 8.0 Hz, 1H, NH), 5.89 (dd, *J* = 6.6, 5.4 Hz, 1H, H_1_), 5.47 (t, *J* = 9.7 Hz, 1H, H_4_), 5.35 (dd, *J* = 10.9, 9.6 Hz, 1H, H_3_), 4.56 (ddd, *J* = 11.1, 8.0, 5.2 Hz, 1H, H_2_), 4.24 (ddd, *J* = 10.0, 3.7, 2.2 Hz, 1H, H_5_), 3.81 (dd, *J* = 11.7, 4.1 Hz, 1H, H_6a_), 3.76 (dd, *J* = 11.7, 2.2 Hz, 1H, H_6b_), 2.51 (t, *J* = 6.7 Hz, 2H, Lev-CH_2_), 2.32 (t, *J* = 6.7 Hz, 2H, Lev-CH_2_), 1.97–1.95 (m, 4H, Lev-CH_3_, SH), 1.90 (s, 3H, CH_3_), 1.07–1.04 (m, 9H, SiC(CH_3_)_3_); ^13^C NMR (100 MHz, CDCl_3_) δ 205.5 (C=O), 171.2 (C=O), 170.1 (C=O), 167.6 (C=O), 135.79 (CH), 135.75 (CH), 133.70 (CH), 133.3 (C_q_), 133.2 (C_q_), 130.1 (CH), 129.70 (CH), 129.67 (CH), 128.8 (CH), 128.6 (CH), 127.7 (CH), 79.0 (C_1_), 71.84 (C_3_), 71.75 (C_5_), 68.1 (C_4_), 62.4 (C_6_), 53.4 (C_2_), 37.9 (Lev-CH_2_), 29.4 (Lev-CH_3_), 27.8 (Lev-CH_2_), 26.8 (SiC(*C*H_3_)_3_), 23.2 (CH_3_), 17.7 (Si*C*(CH_3_)_3_). HRMS (ESI^+^) *m*/*z* found (M+H)^+^ 678.2537; C_36_H_44_NO_8_SSi requires 678.2552.

*S*-(2-Acetamido-3,6-di-*O*-benzoyl-2-deoxy-4-*O*-levulinoyl-α-d-glucopyranosyl)-(1→4)-*p*-methoxyphenyl 2,3-di-*O*-benzoyl-4-thio-6-*O*-(triisopropylsilyl)-β-d-glucopyranoside (**37**)

*S*-linked disaccharide **37** was prepared following general procedure A using thiohemiacetal **34** (98.0 mg, 0.270 mmol), NaH (11 mg of a 60% dispersion in mineral oil, 0.270 mmol), and triflate **7** (318 mg, 0.405 mmol) in THF (5.8 mL). Purification via column chromatography (1/0 → 9:1, CH_2_Cl_2_/Et_2_O) furnished title compound **37** as a white solid (145 mg, 0.128 mmol, 47%). R_f_ = 0.41 (95/5, CH_2_Cl_2_/Et_2_O); m.p. 107–109 °C; ${\left[\alpha \right]}_{D}^{25}$ +113.3 (c = 2.0, CHCl_3_); ^1^H NMR (400 MHz, CDCl_3_) δ 8.11–8.06 (m, 2H, Ph), 7.98–7.85 (m, 6H, Ph), 7.62–7.55 (m, 1H, Ph), 7.55–7.45 (m, 5H, Ph), 7.42–7.32 (m, 6H, Ph), 6.98–6.91 (m, 2H, Ph), 6.77–6.71 (m, 2H, Ph), 5.75 (dd, *J* = 10.9, 9.6 Hz, 1H, H_3_), 5.66 (d, *J* = 5.3 Hz, 1H, H_1′_), 5.55–5.49 (m, 1H, H_2_), 5.49 (d, *J* = 9.5 Hz, 1H, NH), 5.41 (t, *J* = 9.7 Hz, 1H, H_4′_), 5.22 (dd, *J* = 11.2, 9.5 Hz, 1H, H_3′_), 5.13 (d, *J* = 7.9 Hz, 1H, H_1_), 4.60 (ddd, *J* = 11.2, 9.2, 5.2 Hz, 1H, H_2′_), 4.54 (dd, *J* = 12.4, 2.2 Hz, 1H, H_6a′_), 4.46 (dd, *J* = 12.4, 3.7 Hz, 1H, H_6b′_), 4.40 (ddd, *J* = 10.1, 3.3, 2.5 Hz, 1H, H_5′_), 4.25 (dd, *J* = 10.9, 1.5 Hz, 1H, H_6a_), 4.14 (dd, *J* = 11.5, 4.6 Hz, 1H, H_6b_), 3.78–3.72 (m, 4H, OCH_3_, H_5_), 3.44 (t, *J* = 10.7, 1H, H_4_), 2.57–2.53 (m, 2H, Lev-CH_2_), 2.47–2.31 (m, 2H, Lev-CH_2_), 1.92 (s, 3H, Lev-CH_3_), 1.35 (s, 3H, CH_3_), 1.09 (m, 21H, Si(C_3_H_7_)_3_). ^13^C NMR (100 MHz, CDCl_3_) δ 205.7 (C=O), 171.4 (C=O), 169.5 (C=O), 167.0 (C=O), 166.2 (C=O), 165.9 (C=O), 165.4 (C=O), 155.7 (C_q_), 151.5 (C_q_), 133.73 (CH), 133.71 (CH), 133.3 (CH), 130.1 (CH), 130.0 (CH), 129.93 (CH), 129.90 (CH), 129.8 (C_q_), 129.4 (C_q_), 128.94 (C_q_), 128.83 (C_q_), 128.78 (CH), 128.64 (CH), 128.61 (CH), 128.5 (CH), 119.2 (CH), 114.5 (CH), 100.8 (C_1_), 85.7 (C_1′_), 77.3 (C_5_), 74.7 (C_3_), 73.2 (C_2_), 71.9 (C_3′_), 70.0 (C_5′_), 68.1 (C_4′_), 63.6 (C_6_), 62.3 (C_6′_), 55.8 (OCH_3_), 52.4 (C_2′_), 46.6 (C_4_), 38.0 (Lev-CH_2_), 29.5 (Lev-CH_3_), 27.9 (Lev-CH_2_), 22.6 (CH_3_), 18.1 (Si(CH(*C*H_3_)_2_)_3_), 12.1 (Si(*C*H(CH_3_)_2_)_3_). HRMS (ESI^+^) *m*/*z* found (M+NH_4_)^+^ 1193.4718; C_63_H_77_N_2_O_17_SSi requires (M+NH_4_)^+^ 1193.4707.

*S*-(2-Azido-3-*O*-benzyl-2-deoxy-4-*O*-levulinoyl-6-*O*-(*tert*-butyldiphenylsilyl)-α-d-glucopyranosyl)-(1→4)-*p*-methoxyphenyl 2,3-di-*O*-benzoyl-4-thio-6-*O*-(triisopropylsilyl)-β-d-glucopyranoside (**38**)

Disaccharide **38** was prepared following general procedure B using thioacetate **40** (100 mg, 0.15 mmol), triflate **7** (122 mg, 0.16 mmol), and Et_2_NH (205 µL, 1.90 mmol) in MeCN (1.2 mL). Purification via column chromatography (1/0 → 9:1, CH_2_Cl_2_/Et_2_O) furnished title compound **38** as a white solid (82.0 mg, 64.0 µmol, 44%). R_f_ = 0.59 (CH_2_Cl_2_); m.p. 60–63 °C; ${\left[\alpha \right]}_{D}^{25}$ +27.4 (c = 0.50, CHCl_3_); ^1^H NMR (400 MHz, CDCl_3_) δ 8.07–8.03 (m, 2H, Ph), 7.97–7.92 (m, 2H, Ph), 7.67–7.63 (m 4H, Ph), 7.53–7.48 (m, 2H, Ph), 7.45–7.33 (m, 11H, Ph), 7.30–7.22 (m, 2H, Ph), 7.18–7.13 (m, 2H, Ph), 6.98–6.92 (m, 2H, Ph), 6.77–6.71 (m, 2H, Ph), 5.69 (dd, *J* = 10.8, 9.7 Hz, 1H, H_3_), 5.56 (dd, *J* = 9.6, 7.9 Hz, 1H, H_2_), 5.49 (d, *J* = 5.0 Hz, 1H, H_1′_), 5.19–5.12 (m, 1H, H_4′_), 5.09 (d, *J* = 7.9 Hz, 1H, H_1_), 4.44 (d, *J* = 11.0 Hz, 1H, PhC*H*H), 4.35 (d, *J* = 11.0 Hz, 1H, PhCH*H*), 4.19 (dd, *J* = 11.0, 1.6 Hz, 1H, H_6a′_), 4.01 (dd, *J* = 11.0, 5.3 Hz, 1H, H_6b′_), 3.82 (dt, *J* = 10.0, 2.7 Hz, 1H, H_5′_), 3.74 (s, 3H, OCH_3_), 3.69–3.65 (m, 3H, H_5_, H_6a_, H_6b_), 3.63 (dd, *J* = 10.2, 5.1 Hz, 1H, H_2′_), 3.56–3.51 (m, 1H, H_3′_), 3.28 (t, *J* = 10.8 Hz, 1H, H_4_), 2.53 (t, *J* = 7.2 Hz, 2H, Lev-CH_2_), 2.26–2.24 (m, 2H, Lev-CH_2_), 2.10 (s, 3H, Lev-CH_3_), 1.07–0.99 (m, 30H, Si(C_3_H_7_)_3_, SiC(CH_3_)_3_); ^13^C NMR (100 MHz, CDCl_3_) δ 206.0 (C=O), 171.1 (C=O), 165.8 (C=O), 165.5 (C=O), 155.7 (C_q_), 151.6 (C_q_), 137.5 (C_q_), 135.88 (CH), 135.86 (CH), 133.4 (CH), 133.3 (CH), 130.04 (C_q_), 129.94 (C_q_), 129.85 (CH), 129.5 (CH), 128.6 (CH), 128.51 (CH), 128.47 (CH), 119.2 (CH), 114.5 (CH), 101.0 (C_1_), 84.4 (C_1′_), 79.5 (C_3′_), 75.1 (PhCH_2_), 74.6 (C_3_), 73.2 (C_2_), 73.0 (C_5′_), 69.8 (C_4′_), 64.1 (C_6′_), 64.0 (C_5_), 63.7 (C_2′_), 62.0 (C_6_) 55.8 (OCH_3_), 46.4 (C_4_), 37.9 (Lev-CH_2_), 29.9 (Lev-CH_3_), 27.9 (Lev-CH_2_), 26.9 (SiC(*C*H_3_)_3_), 19.4 (Si*C*(CH_3_)_3_), 18.1 Si(CH(*C*H_3_)_2_)_3_, 12.1 (Si(*C*H(CH_3_)_2_)_3_). HRMS (ESI^+^) *m*/*z* found (M+Na)^+^ 1303.5196; C_70_H_85_N_3_O_14_SSi_2_Na requires 1303.5211.

*S*-(2-Acetamido-2-deoxy-3-*O*-benzoyl-4-*O*-levulinoyl-6-*O*-(*tert*-butyldiphenylsilyl)-α-d-glucopyranosyl)-(1→4)-*p*-methoxyphenyl 2,3-di-*O*-benzoyl-4-thio-6-*O*-(triisopropylsilyl)-β-d-glucopyranoside (**41**)

*S*-linked **41** was prepared following general procedure B using thioacetate **39** (0.974 g, 1.35 mmol), triflate **7** (1.17 g, 1.49 mmol), and Et_2_NH (1.80 mL, 17.6 mmol) in MeCN (13 mL). Purification via column chromatography (1/0 → 9:1, CH_2_Cl_2_/Et_2_O) generated title compound **41** as a white solid (1.29 g, 0.984 mmol, 73%). R_f_ = 0.44 (99/1, CH_2_Cl_2_/Et_2_O); m.p. 102–105 °C; ${\left[\alpha \right]}_{D}^{23}$ +50.9 (c = 0.50, CHCl_3_); ^1^H NMR (400 MHz, CDCl_3_) δ 7.95–7.87 (m, 6H, Ph), 7.74–7.64 (m, 4H, Ph), 7.56–7.46 (m, 3H, Ph), 7.46–7.32 (m, 12H, Ph), 6.97–6.92 (m, 2H, Ph), 6.76–6.72 (m, 2H, Ph), 5.70 (dd, *J* = 10.8, 9.6 Hz, 1H, H_3_), 5.61 (d, *J* = 5.3 Hz, 1H, H_1′_), 5.51 (dd, *J* = 9.5, 7.9 Hz, 1H, H_4′_), 5.51 (d, *J* = 9.8 Hz, 1H, NH), 5.49 (dd, *J* = 9.7, 7.6 Hz, 1H, H_2_), 5.16 (dd, *J* = 11.2, 9.5 Hz, 1H, H_3′_), 5.09 (d, *J* = 7.9 Hz, 1H, H_1_), 4.56 (ddd, *J* = 11.3, 9.2, 5.2 Hz, 1H, H_2′_), 4.17 (dd, *J* = 10.9, 1.6 Hz, 1H, H_6a′_), 4.09 (dt, *J* = 10.3, 2.3 Hz, 1H, H_5′_), 3.99 (dd, *J* = 11.0, 5.7 Hz, 1H, H_6b′_), 3.78 (t, *J* = 7.1 Hz, 2H, H_6a_, H_6b_), 3.74 (s, 3H, OCH_3_), 3.73–3.69 (m, 1H, H_5_), 3.25 (t, *J* = 10.8 Hz, 1H, H_4_), 2.49–2.47 (m, 2H, Lev-CH_2_), 2.27 (t, *J* = 6.7 Hz, 2H, Lev-CH_2_), 1.93 (s, 3H, Lev-CH_3_), 1.33 (s, 3H, CH_3_), 1.09 (s, 9H, SiC(CH_3_)_3_), 1.01 (s, 21H, (Si(C_3_H_7_)_3_); ^13^C NMR (100 MHz, CDCl_3_) δ 205.5 (C=O), 170.9 (C=O), 169.2 (C=O), 167.0 (C=O), 165.7 (C=O), 165.3 (C=O), 155.6 (C_q_), 151.4 (C_q_), 135.79 (CH), 135.76 (CH), 133.6 (CH), 133.5 (CH), 133.2 (CH), 133.1 (C_q_), 133.03 (C_q_), 129.99 (C_q_), 129.8 (CH), 129.3 (C_q_), 128.9 (C_q_), 128.8 (CH), 128.6 (CH), 128.43 (CH), 128.35 (CH), 127.7 (CH), 119.0 (CH), 114.4 (CH), 100.7 (C_1_), 85.7 (C_1′_), 77.1 (C_5_), 74.9 (C_2_), 73.1 (C_3′_), 72.4 (C_5′_), 72.3 (C_3_), 67.7 (C_4′_), 63.6 (C_6′_), 61.8 (C_6_), 55.6 (OCH_3_), 52.3 (C_2′_), 46.3 (C_4_), 37.9 (Lev-CH_2_), 29.4 (Lev-CH_3_), 27.7 (Lev-CH_2_), 26.8 (SiC(*C*H_3_)_3_), 22.5 (CH_3_), 19.3 (Si*C*(CH_3_)_3_), 17.9 (Si(CH(*C*H_3_)_2_)_3_), 11.9 (Si(*C*H(CH_3_)_2_)_3_). HRMS (ESI^+^) *m*/*z* found (M+H)^+^ 1313.5403; C_72_H_88_NO_16_SSi_2_ requires 1313.5395.

*S*-(2-Acetamido-3-*O*-benzoyl-2-deoxy-4-*O*-levulinoyl-6-O-(tert-butyldiphenylsilyl)-α-d-glucopyranosyl)-(1→4)-p-methoxyphenyl 2,3-di-O-benzoyl-4-thio-β-d-glucopyranoside (**42**)

Disaccharide **42** was prepared following general procedure C using *S*-(2-azido-3-*O*-benzyl-2-deoxy-4-*O*-levulinoyl-6-*O*-(*tert*-butyldiphenylsilyl)-α-d-glucopyranosyl)-(1→4)-*p*-methoxyphenyl 2,3-di-*O*-benzoyl-4-thio-6-*O*-(triisopropylsilyl)-β-d-glucopyranoside **41** (822 mg, 0.70 mmol), TFA (644 µL, 8.40 mmol), and H_2_O (252 µL, 14.0 mmol) in MeCN (7 mL). Purification via column chromatography (1/0 → 8:2, CH_2_Cl_2_/Et_2_O) generated compound **42** as a white solid (0.55 g, 0.476 mmol, 68%). R_f_ = 0.23 (95/5, CH_2_Cl_2_/Et_2_O); m.p. 108–110 °C; ${\left[\alpha \right]}_{D}^{25}$ +43.8 (c = 1.1, CHCl_3_); ^1^H NMR (400 MHz, CDCl_3_) δ 7.96–7.87 (m, 6H, Ph), 7.75–7.71 (m, 2H, Ph), 7.70–7.66 (m, 2H, Ph), 7.55–7.47 (m, 3H, Ph), 7.46–7.31 (m, 12H, Ph), 6.92–6.86 (m, 2H), 6.80–6.74 (m, 2H, Ph), 5.72 (dd, *J* = 11.0, 9.6 Hz, 1H, H_3_), 5.60 (d, *J* = 5.3 Hz, 1H, H_1′_), 5.58–5.51 (m, 2H, NH, H_4′_), 5.46 (t, *J* = 9.8 Hz, 1H, H_2_), 5.16 (d, *J* = 7.9 Hz, 1H, H_1_), 5.15 (t, *J* = 10.4 Hz, 1H, H_3′_), 4.55 (ddd, *J* = 11.3, 9.1, 5.2 Hz, 1H, H_2′_), 4.16 (dt, *J* = 10.2, 2.6 Hz, 1H, H_5′_), 3.96–3.91 (m, 2H, H_6a′_, H_6b′_), 3.85–3.77 (m, 2H, H_6a_, H_6b_), 3.75 (s, 3H, OCH_3_), 3.70 (dt, *J* = 10.8, 3.3 Hz, 1H, H_5_), 3.41 (t, *J* = 10.9 Hz, 1H, H_4_), 2.48 (t, *J* = 6.8 Hz, 2H, Lev-CH_2_), 2.29 (t, *J* = 6.7 Hz, 2H, Lev-CH_2_), 2.07 (t, *J* = 6.9 Hz, 1H, 6-OH), 1.94 (s, 3H, Lev-CH_3_), 1.20 (s, 3H, CH_3_), 1.10 (s, 9H, SiC(CH_3_)_3_); ^13^C NMR (100 MHz, CDCl_3_) δ 205.5 (C=O), 171.0 (C=O), 169.7 (C=O), 167.1 (C=O), 165.7 (C=O), 165.2 (C=O), 155.7 (C_q_), 151.0 (C_q_), 135.8 (CH), 135.7 (CH), 133.6 (CH), 133.23 (C_q_), 133.16 (C_q_), 133.02 (C_q_), 130.00 (CH), 129.79 (CH), 129.75 (CH), 129.2 (C_q_), 128.78 (C_q_), 128.68 (CH), 128.6 (CH), 128.5 (CH), 128.4 (CH), 127.7 (CH), 118.7 (CH), 114.6 (CH), 100.5 (C_1_), 86.7 (C_1′_), 76.4 (C_5′_), 74.5 (C_3_), 72.9 (C_5_), 72.3 (C_4′_), 72.2 (C_3′_), 67.8 (C_2_), 62.3 (C_6′_), 62.0 (C_6_), 55.6 (OCH_3_), 53.4 (C_2′_), 46.6 (C_4_), 37.9 (Lev-CH_2_), 29.4 (Lev-CH_3_), 27.7 (Lev-CH_2_), 26.8 (SiC(*C*H_3_)_3_), 22.2 (CH_3_), 19.3 (Si*C*(CH_3_)_3_). HRMS (ESI^+^) *m*/*z* found: (M+Na)^+^ 1176.3821; C_63_H_67_NO_16_SSiNa requires 1176.3829.

*S*-(2-Azido-3-*O*-benzyl-2-deoxy-4-*O*-levulinoyl-6-*O*-(*tert*-butyldiphenylsilyl)-α-d-glucopyranosyl)-(1→4)-*p*-methoxyphenyl 2,3-di-*O*-benzoyl-4-thio-β-d-glucopyranoside (**43**)

*S*-linked **43** was prepared following general procedure C using *S*-(2-azido-3-*O*-benzyl-2-deoxy-4-*O*-levulinoyl-6-*O*-(*tert*-butyldiphenylsilyl)-α-d-glucopyranosyl)-(1→4)-*p*-methoxyphenyl 2,3-di-*O*-benzoyl-4-thio-6-*O*-(triisopropylsilyl)-β-d-glucopyranoside **38** (822 mg, 0.699 mmol), TFA (0.641 mL, 8.38 mmol), and H_2_O (256 µL, 14.00 mmol) in MeCN (7 mL). Purification via column chromatography (1/0 → 8:2, CH_2_Cl_2_/Et_2_O) generated title compound **43** as a white solid (433 mg, 0.385 mmol, 55%). R_f_ = 0.21 (99/1, CH_2_Cl_2_/Et_2_O); m.p. 128–129 °C; ${\left[\alpha \right]}_{D}^{25}$ +46.3 (c = 1.0, CHCl_3_); ^1^H NMR (400 MHz, CDCl_3_) δ 8.05–8.01 (m, 2H, Ph), 7.98–7.92 (m, 2H, Ph), 7.71–7.64 (m, 4H, Ph), 7.54–7.50 (m, 2H, Ph), 7.46–7.35 (m, 9H, Ph), 7.31–7.23 (m, 4H, Ph), 7.22–7.17 (m, 2H, Ph), 6.92–6.86 (m, 2H, Ph), 6.80–6.74 (m, 2H, Ph), 5.75 (dd, *J* = 10.9, 9.7 Hz, 1H, H_3_), 5.58 (dd, *J* = 9.7, 7.9 Hz, 1H, H_2_), 5.40 (d, *J* = 5.1 Hz, 1H, H_1′_), 5.17 (d, *J* = 7.8 Hz, 1H, H_1_), 5.13 (dd, *J* = 9.7, 8.6 Hz, 1H, H_4′_), 4.58 (d, *J* = 11.0 Hz, 1H, PhC*H*H), 4.47 (d, *J* = 11.1 Hz, 1H, PhCH*H*), 4.00–3.88 (m, 3H, H_6a_, H_6b_, H_5′_), 3.74 (s, 3H, OCH_3_), 3.73–3.71 (m, 2H, H_6a′_, H_6b′_), 3.70–3.64 (m, 1H, H_5_), 3.59 (dd, *J* = 10.1, 5.1 Hz, 1H, H_2′_), 3.53 (dd, *J* = 10.0, 8.8 Hz, 1H, H_3′_), 3.36 (t, *J* = 10.9 Hz, 1H, H_4_), 2.55 (t, *J* = 7.0 Hz, 2H, Lev-CH_2_), 2.35–2.26 (m, 2H, Lev-CH_2_), 2.11 (s, 3H, Lev-CH_3_), 2.00 (t, *J* = 6.9 Hz, 1H, 6-OH), 1.06 (s, 9H, SiC(CH_3_)_3_); ^13^C NMR (100 MHz, CDCl_3_) δ 205.9 (C=O), 171.0 (C=O), 165.6 (C=O), 165.3 (C=O), 155.7 (C_q_), 151.0 (C_q_), 137.4 (C_q_), 135.8 (CH), 135.7 (CH), 133.4 (C_q_), 133.27 (C_q_), 133.25 (CH), 129.83 (CH), 129.77 (CH), 129.75 (CH), 129.7 (CH), 129.3 (C_q_), 128.6 (CH), 128.40 (CH), 128.37 (CH), 128.1 (CH), 127.9 (C_q_), 127.7 (CH), 118.7 (CH), 114.6 (CH), 100.7 (C_1_), 85.6 (C_1′_), 79.2 (C_3′_), 76.1 (C_5′_), 75.0 (PhCH_2_), 74.7 (C_3_), 72.8 (C_5_), 72.7 (C_2_), 69.8 (C_4′_), 63.6 (C_2′_), 62.4 (C_6_), 62.1 (C_6′_), 55.6 (OCH_3_), 46.1 (H_4_), 37.7 (Lev-CH_2_), 29.8 (Lev-CH_3_), 27.8 (Lev-CH_2_), 26.8 (SiC(*C*H_3_)_3_), 19.3 (Si*C*(CH_3_)_3_). HRMS (ESI^+^) *m*/*z* found (M+Na)^+^ 1146.3832; C_61_H_65_N_3_O_14_SSiNa requires 1146.3830.

Methyl (*S*-(2-acetamido-3-*O*-benzoyl-2-deoxy-4-*O*-levulinoyl-6-*O*-(*tert*-butyldiphenylsilyl)-α-d-glucopyranosyl)-(1→4)-(*p*-methoxyphenyl 2,3-di-*O*-benzoyl-4-thio-β-d-glucopyranosid)uronate (**44**)

*S*-linked **44** was prepared following general procedure D using *S*-(2-Acetamido-3-*O*-benzoyl-2-deoxy-4-*O*-levulinoyl-6-*O*-(*tert*-butyldiphenylsilyl)-α-d-glucopyranosyl)-(1→4)-*p*-methoxyphenyl 2,3-di-*O*-benzoyl-4-thio-β-d-glucopyranoside **42** (402 mg, 0.346 mmol), TEMPO (10.9 mg, 69.2 µmol), and BAIB (283 mg, 0.87 mmol). The crude acid was treated with K_2_CO_3_ (145 mg, 1.05 mmol) and MeI (70.0 µL, 1.05 mmol) in DMF (1.2 mL). Purification via column chromatography (1/0 → 9:1, CH_2_Cl_2_/Et_2_O) generated title compound **44** as a white solid (326 mg, 0.28 mmol, 80%). R_f_ = 0.61 (9/1, CH_2_Cl_2_/Et_2_O); m.p. 109–111 °C; ${\left[\alpha \right]}_{D}^{23}$ +195.3 (c = 0.50, CHCl_3_); ^1^H NMR (400 MHz, CDCl_3_) δ 7.96–7.88 (m, 6H, Ph), 7.77–7.73 (m, 2H, Ph), 7.67–7.65 (m, 2H, Ph), 7.57–7.48 (m, 3H, Ph), 7.46–7.33 (m, 12H, Ph), 6.92–6.85 (m, 2H, Ph), 6.81–6.73 (m, 2H, Ph), 5.68 (dd, *J* = 10.6, 9.2 Hz, 1H, H_3_), 5.64 (d, *J* = 5.2 Hz, 1H, H_1′_), 5.65–5.55 (m, 3H, H_2_, H_4′_, NH), 5.17 (d, *J* = 7.2 Hz, 1H, H_1_), 5.15 (dd, *J* = 11.3, 9.5 Hz, 1H, H_3′_), 4.53 (ddd, *J* = 11.4, 8.8, 5.2 Hz, 1H, H_2′_), 4.24 (d, *J* = 10.6 Hz, 1H, H_5_), 4.04–3.99 (m, 1H, H_5′_), 3.93 (dd, *J* = 11.8, 1.7 Hz, 1H, H_6b′_), 3.77 (dd, *J* = 11.8, 2.3 Hz, 1H, H_6b′_), 3.74 (s, 3H, OCH_3_), 3.68 (t, *J* = 10.6 Hz, 1H, H_4_), 3.55 (s, 3H, OCH_3_), 2.49 (t, *J* = 6.9 Hz, 2H, Lev-CH_2_), 2.31 (t, *J* = 6.6 Hz, 2H, Lev-CH_2_), 1.93 (s, 3H, Lev-CH_3_), 1.38 (s, 3H, CH_3_), 1.10 (s, 9H, SiC(CH_3_)_3_); ^13^C NMR (100 MHz, CDCl_3_) δ 205.6 (C=O), 170.9 (C=O), 169.5 (C=O), 167.2 (C=O), 166.9 (C=O), 165.4 (C=O), 165.1 (C=O), 155.8 (C_q_), 150.9 (C_q_), 135.9 (CH), 135.8 (CH), 133.7 (C_q_), 133.5 (C_q_), 133.34 (CH), 133.31 (CH), 133.2 (CH), 130.0 (CH), 129.82 (CH), 129.78 (CH), 129.73 (CH), 129.67 (CH), 129.0 (CH), 128.8 (CH), 128.6 (C_q_), 128.44 (C_q_), 128.40 (CH), 127.7 (CH), 127.6 (CH), 118.8 (CH), 114.5 (CH), 100.9 (C_1_), 85.7 (C_1′_), 75.9 (C_5_), 73.56 (C_3_), 72.7 (C_2_), 72.3 (C_3′_), 71.7 (C_5′_), 67.5 (C_4′_), 61.3 (C_6′_), 55.6 (OCH_3_), 52.9 (OCH_3_), 52.6 (C_2′_), 45.7 (C_4_), 37.9 (Lev-CH_2_), 29.4 (Lev-CH_3_), 27.8 (Lev-CH_2_), 26.8 (SiC(*C*H_3_)_3_), 22.5 (CH_3_), 19.3 (Si*C*(CH_3_)_3_). HRMS (ESI^+^) *m*/*z* found (M+Na)^+^ 1204.3798; C_64_H_67_NO_17_SSiNa requires 1204.3791.

Methyl *S*-(2-azido-3-*O*-benzyl-2-deoxy-4-*O*-levulinoyl-6-*O*-(*tert*-butyldiphenylsilyl)-α-d-glucopyranosyl)-(1→4)-(*p*-methoxyphenyl 2,3-*O*-dibenzoyl-4-thio-β-d-glucopyranosid)uronate (**45**)

*S*-linked **45** was prepared following general procedure D using *S*-(2-azido-3-*O*-benzyl-2-deoxy-4-*O*-levulinoyl-6-*O*-(*tert*-butyldiphenylsilyl)-α-d-glucopyranosyl)-(1→4)-*p*-methoxyphenyl 2,3-di-*O*-benzoyl-4-thio-β-d-glucopyranoside **43** (248 mg, 0.222 mmol), TEMPO (6.00 mg, 398 µmol), and BAIB (177 mg, 0.550 mmol). The crude acid was treated with K_2_CO_3_ (91.0 mg, 0.660 mmol) and MeI (40.0 µL, 0.666 mmol) in DMF (1.5 mL). Purification via column chromatography (1/0 → 9:1, CH_2_Cl_2_/Et_2_O) generated title compound **45** as a white solid (205 mg, 0.177 mmol, 80%). R_f_ = 0.82 (9/1, CH_2_Cl_2_/Et_2_O); m.p. 88–90 °C; ${\left[\alpha \right]}_{D}^{23}$ +95.0 (c = 0.50, CHCl_3_); ^1^H NMR (400 MHz, CDCl_3_) δ 8.06–8.00 (m, 2H, Ph), 7.99–7.90 (m, 2H, Ph), 7.76–7.69 (m, 2H, Ph), 7.70–7.63 (m, 2H, Ph), 7.57–7.48 (m, 2H, Ph), 7.48–7.33 (m, 10H, Ph), 7.32–7.18 (m, 5H, Ph), 6.94–6.85 (m, 2H, Ph), 6.80–6.73 (m, 2H, Ph), 5.74 (dd, *J* = 10.6, 9.3 Hz, 1H, H_3_), 5.61 (dd, *J* = 9.2, 7.2 Hz, 1H, H_2_), 5.44 (d, *J* = 5.1 Hz, 1H, H_1′_), 5.31 (dd, *J* = 9.8, 9.0 Hz, 1H, H_4′_), 5.19 (d, *J* = 7.2 Hz, 1H, H_1_), 4.58 (d, *J* = 11.0 Hz, 1H, PhC*H*H), 4.46 (d, *J* = 11.1 Hz, 1H, PhCH*H*), 4.21 (d, *J* = 10.7 Hz, 1H, H_5_), 3.86–3.78 (m, 2H, H_5′_, H_6a′_), 3.74–3.69 (m, 4H, H_6b′_, OCH_3_), 3.65–3.58 (m, 2H, H_2_, H_4_), 3.58–3.51 (m, 4H, H_3′_, OCH_3_), 2.66–2.50 (m, 2H, Lev-CH_2_), 2.42–2.24 (m, 2H, Lev-CH_2_), 2.12 (s, 3H, Lev-CH_3_), 1.06 (s, 9H, SiC(CH_3_)_3_); ^13^C NMR (100 MHz, CDCl_3_) δ 206.0 (C=O), 170.9 (C=O), 167.28 (C=O), 165.34 (C=O), 165.2 (C=O), 155.8 (C_q_), 150.9 (C_q_), 137.4 (C_q_), 135.83 (CH), 135.77 (CH), 133.48 (CH), 133.45 (C_q_), 133.40 (C_q_), 133.31 (CH), 129.85 (CH), 129.8 (CH), 129.74 (CH), 129.67 (CH), 129.12 (C_q_), 129.08 (C_q_), 128.6 (CH), 128.42 (CH), 128.36 (CH), 128.1 (CH), 127.9 (CH), 127.64 (CH), 127.63 (CH), 118.8 (CH), 114.5 (CH), 101.1 (C_1_), 84.6 (C_1′_), 79.3 (C_3_), 75.5 (C_5_), 75.0 (PhCH_2_), 74.1 (C_3′_), 72.7 (C_2_), 72.1 (C_5′_), 69.5 (C_4′_), 63.6 (C_2′_), 61.4 (C_6′_), 55.6 (OCH_3_), 52.8 (OCH_3_), 45.1 (C_4_), 37.8 (Lev-CH_2_), 29.8 (Lev-CH_3_), 27.8 (Lev-CH_2_), 26.8 (SiC(*C*H_3_)_3_), 19.4 (Si*C*(CH_3_)_3_). HRMS (ESI^+^) *m*/*z* found (M+NH_4_)^+^ 1169.4240; C_62_H_69_N_4_O_15_SSi requires 1169.4244.

## 4. Conclusions

The synthesis of *S*-linked d-GlcN-α(1→4)-d-GlcA disaccharide building blocks has been completed. Using an S_N_2 reaction to combine an anomeric α-thiol with a galactosyl triflate electrophile, a robust, gram-scale glycosylation protocol is established. Initial NaH-mediated coupling, in which the anomeric thiol was converted to the thiolate, gave disaccharides at 35% to 40% yields. Using Et_2_NH, where anomeric thioacetates were unmasked in situ, facilitated entry to improved yields of up to 73%. *S*-Linked disaccharides were thereafter subjected to *O*-6 modification to generate *S*-linked d-GlcN-α(1→4)-d-GlcA systems. The manipulation of these materials into appropriate d-GlcN-α(1→4)-S-d-GlcA donor and acceptor building blocks presents an opportunity to pursue *S*-linked HS oligosaccharide synthesis.

## Data Availability

Data are contained within the article and Supplementary Materials.

## Supplementary Materials

The following supporting information can be downloaded at https://www.mdpi.com/article/10.3390/molecules29235809/s1: Relevant NMR spectra for compounds **3**–**45**. References [20,42,43,44,45,46,47,48] are cited in the Supplementary Materials.
