# Supplementary material for: Synthesis of S-Glycoside Building Blocks as Mimetics of the Repeating d-GlcN-α-1,4-d-GlcA Heparan Sulfate Disaccharide
Source: Molecules. 2024 Dec 9;29(23):5809. doi: 10.3390/molecules29235809 (PMC11643514; doi:10.3390/molecules29235809)
Supplement: Supplementary file 1 [file molecules-29-05809-s001.zip › molecules-3307243-supplementary.pdf]

Synthesis of *S*-Glycoside Building Blocks as Mimetics of the Repeating D-GlcN-*α*-1,4-D-GlcA Heparan Sulfate Disaccharide

Conor O'Shea and Gavin J. Miller *

School of Chemical and Physical Sciences and Centre for Glycoscience, Keele University, Keele, Staffordshire ST5 5BG, UK
Email: g.j.miller@keele.ac.uk

Supplementary Information

Contents

Chemical Synthesis 2
- Synthesis of C4-Thio Glucuronic acid and Glucose Acceptors 2
- Synthesis of Glucosamine Donors 7
- Ester Migrated Side Products 10
- *S*-Glycosylation Side Products 12
- Synthesis of Gluco-azide α-thiol 36 and Thioacetates 39 and 40 13
- Side products formed using NaH mediated S_N2 Coupling 15

NMR Spectra 18
- Synthesis of C4-Glucuronic and Glucose Acceptors 18
- Synthesis of Glucosamine Donors 88
- Side Products Obtained From Attempted *S*-glycosylations 113
- Synthesis of Anomeric Thiols and Thioacetates 123
- *S*-linked Disaccharides 178
- Side Products Obtained During NaH Mediated S_N2 Coupling 205

References 229

Chemical Synthesis

Synthesis of C4-Thio Glucuronic acid and Glucose Acceptors

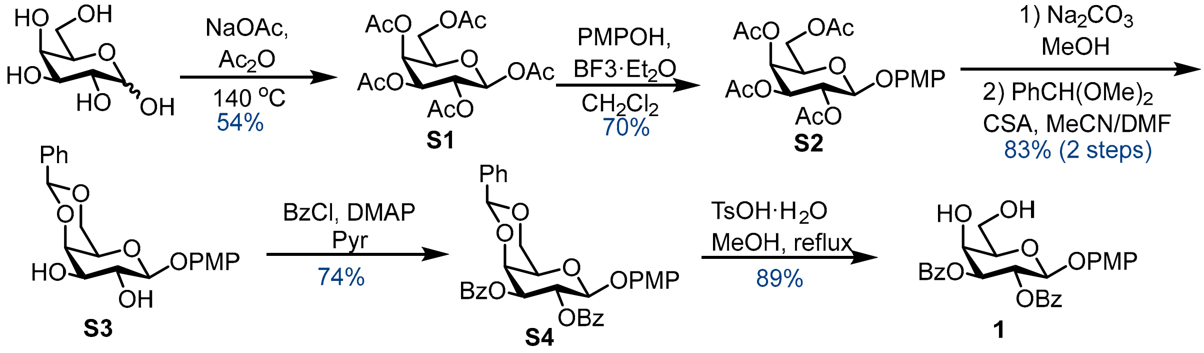

Scheme S1. *Synthesis of galactosyl diol **1**.*

1,2,3,4,6-Penta-*O*-acetyl-β-D-galactopyranose S1

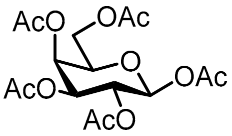

A suspension of NaOAc (5.10 g, 62.6 mmol, 1.11 equiv.) in acetic anhydride (104 mL, 1.06 mol, 17.0 equiv.) was refluxed for 30 min at 140 °C before D-galactose (10.0 g, 56.0 mmol, 1.00 equiv.) was added slowly at RT. The reaction was left at reflux for 1 h with stirring whereupon TLC analysis (2/1, cyclohexane/EtOAc) revealed full conversion of the starting material to a higher R_f spot. The reaction mixture was poured onto ice/water (600 mL) and left to stir overnight. The cloudy precipitate was filtered and recrystallised from hot MeOH (20 mL) to furnish title compound **S1** as a white solid (11.8 g, 30.2 mmol, 54%). R_f = 0.51 (2/1, cyclohexane/EtOAc); ^{1}H NMR (400 MHz, $CDCl_3$) δ 5.70 (d, J = 8.3 Hz, 1H, H_1), 5.43 (d, J = 2.7 Hz, 1H, H_4), 5.34 (dd, J = 10.4, 8.3 Hz, 1H, H_2), 5.08 (dd, J = 10.4, 3.4 Hz, 1H, H_3), 4.12 (m, 3H, H_5, H_{6a}, H_{6b}), 2.17 (s, 3H, CH_3), 2.13 (s, 3H, CH_3), 2.05 (s, 6H, 2 x CH_3), 2.00 (s, 3H, CH_3); ^{13}C NMR (100 MHz, $CDCl_3$) δ 170.4 (C=O), 170.1 (C=O), 170.0 (C=O), 169.4 (C=O), 169.0 (C=O), 92.2 (C_1), 71.7 (C_5), 70.8 (C_3), 67.8 (C_2), 66.8 (C_4), 61.0 (C_6), 20.7 (5 x CH_3). NMR data were consistent with literature reported previously.[1] HRMS (ESI$^+$) *m/z* found: $(M+Na)^+$ 413.1052, $C_{16}H_{22}O_{11}Na$ requires 413.1054.

***p*-Methoxyphenyl 2,3,4,6-tetra-*O*-acetyl-β-D-galactopyranoside S2**

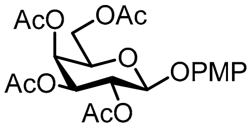

A solution of 1,2,3,4,6-penta-*O*-acetyl-β-D-galactopyranoside **S1** (31.80 g, 81.47 mmol, 1.00 equiv.) and *p*-methoxyphenol (11.63 g, 93.69 mmol, 1.15 equiv.) in CH_2Cl_2 (235 mL) was treated with the dropwise addition of $BF_3·Et_2O$ (25.14 mL, 20.37 mmol, 2.50 equiv.) at 0 ºC. The reaction was left stirring for 3.5 h at RT whereupon TLC analysis (9/1, CH_2Cl_2/Et_2O) revealed full conversion of the starting material to a higher R_f spot. The reaction was quenched with $NaHCO_3$ (100 mL) and the biphasic layers were separated. The aqueous layer was extracted with CH_2Cl_2 (100 mL) and the organic layers were combined, dried over anhydrous $MgSO_4$, filtered and concentrated *in vacuo* to give a yellow oil, which solidified overnight upon standing to afford a whitish/yellow solid. Purification of this crude material *via* recrystallisation in hot MeOH (30 mL) generated title compound **S2** as a white solid (26.00 g, 57.21 mmol, 70%). R_f = 0.30 (2/1, hexane/EtOAc); ^{1}H NMR (400 MHz, $CDCl_3$) δ 6.98 – 6.93 (m,

2H, Ph), 6.84 – 6.80 (m, 2H, Ph), 5.48 – 5.43 (m, 2H, H_2, H_4), 5.09 (dd, J = 10.5, 3.4 Hz, 1H, H_3), 4.92 (d, J = 8.0 Hz, 1H, H_1), 4.24 (dd, J = 11.3, 6.9 Hz, 1H, H_{6a}), 4.16 (dd, J = 11.3, 6.5 Hz, 1H, H_{6b}), 4.01 (td, J = 6.7, 1.0 Hz, 1H, H_5), 3.78 (s, 1H, OCH_3), 2.18 (s, 3H, CH_3), 2.09 (s, 3H, CH_3), 2.06 (s, 3H, CH_3), 2.01 (s, 3H, CH_3); ^{13}C NMR (100 MHz, $CDCl_3$) δ 170.4 (C=O), 170.3 (C=O), 170.2 (C=O), 169.4 (C=O), 155.8 (C_q), 151.1 (C_q), 118.7 (CH), 114.6 (CH), 100.9 (C_1), 70.9 (C_3), 70.9 (C_5), 68.8 (C_2), 66.9 (C_4), 61.3 (C_6), 55.7 (OCH_3), 20.8 (CH_3), 20.69 (CH_3), 20.68 (CH_3), 20.6 (CH_3). NMR data was consistent with literature reported previously.[2] HRMS (ESI^+) *m/z* found: $(M+NH_4)^+$ 472.1809, $C_{21}H_{30}NO_{11}$ requires 472.1813.

***p*-Methoxyphenyl 4,6-*O*-benzylidene-β-D-galactopyranoside S3**

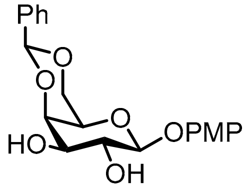

A suspension of *p*-methoxyphenyl 2,3,4,6-tetra-*O*-acetyl-β-D-galactopyranoside **S2** (7.46 g, 26.2 mmol, 1.00 equiv.) and TsOH·H_2O (219 mg, 1.31 mmol, 0.05 equiv.) in MeCN (130 mL) was treated with $PhCH(OMe)_2$ (7.85 mL, 52.4 mmol, 2.00 equiv.) and left stirring at RT for 1 day. TLC analysis (9/1, CH_2Cl_2/Et_2O) revealed complete conversion of the starting material to a higher R_f spot. The reaction mixture was cooled to 0 ºC to furnish **S3** as a white solid which was isolated *via* filtration. The resultant filtrate was concentrated *in vacuo* and filtered to generate another batch of white crystals which were washed with PE/Et_2O (15 mL, 2/1) to remove unreacted benzaldehyde dimethyl acetal. Overall yield of title compound **S3** was 8.11 g, 21.7 mmol, 83%. R_f = 0.13 (9/1, CH_2Cl_2/Et_2O): ^1H NMR (400 MHz, $CDCl_3$) δ 7.53 – 7.51 (m, 2H, Ph), 7.40 – 7.35 (m, 3H, Ph), 7.08 – 7.06 (m, 2H, Ph), 6.84 – 6.82 (m, 2H, Ph), 5.58 (s, 1H, PhCH), 4.79 (d, J = 7.7 Hz, 1H, H_1), 4.37 (d, J = 12.5, 1.0 Hz, 1H, H_{6a}), 4.27 (d, J = 3.4 Hz, 1H, H_4), 4.11 (dd, J = 12.5, 1.7 Hz, 1H, H_{6b}), 4.01 (t, J = 8.7 Hz, 1H, H_2), 3.78 (m, 4H, H_3, OCH_3), 3.58 (br s, 1H, H_5), 2.57 (m, 2H, OH x 2); ^{13}C NMR (100 MHz, $CDCl_3$) δ 154.6 (C_q), 150.1 (C_q), 136.4 (C_q), 128.3 (CH), 127.3 (CH), 125.4 (CH), 118.0 (CH), 113.5 (CH), 101.4 (C_1), 100.5 (PhCH), 74.1 (C_4), 71.7 (C_3), 70.5 (C_2), 68.1 (C_6), 65.8 (C_5), 54.6 (OCH_3). NMR data were consistent with literature reported previously.[2] HRMS (ESI^-) *m/z* found: $(M-H)^-$ 373.1293, $C_{20}H_{21}O_7$ requires 373.1293.

***p*-Methoxyphenyl 2,3-di-*O*-benzoyl-4,6-*O*-benzylidene-β-D-galactopyranoside S4**

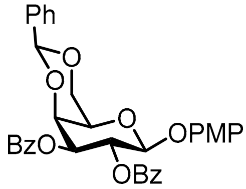

A solution of *p*-methoxyphenyl 4,6-*O*-benzylidene-β-D-galactopyranoside **S3** (2.98 g, 8.03 mmol, 1.00 equv.) and DMAP (100 mg, 0.8 mmol, 0.10 equiv.) in pyridine (25 mL) was treated with BzCl (2.80 mL, 24.1 mmol, 3.00 equiv.) at 0 ºC and the reaction was stirred at RT for 1 day. TLC (9/1, CH_2Cl_2/Et_2O) revealed full conversion of the starting material to a higher R_f spot. The reaction was diluted with CH_2Cl_2 (25 mL) and washed with $NaHCO_3$ (2 x 30 mL). The aqueous layer was extracted with CH_2Cl_2 (30 mL), and the combined organic layers were washed with brine (50 mL), dried over anhydrous $MgSO_4$, filtered and concentrated *in vacuo* to give a yellow solid. This crude material was washed with ice-cold MeOH (5 mL) to furnish **S4** as a white solid. The resultant filtrate was concentrated *in vacuo* to give a yellow oil which formed a precipitate following the addition of ice-cold hexane (5 mL). The precipitate was filtered to furnish another batch of white solid. Overall yield of title compound **S4** was 3.47 g, 5.96 mmol, 74%. R_f = 0.59 (CH_2Cl_2); ^1H NMR (400 MHz, $CDCl_3$) δ 8.02 – 7.97 (m, 4H, Ph), 7.55 – 7.50 (m, 4H, Ph), 7.40 – 7.36 (m, 7H, Ph), 7.02 – 6.97 (m, 2H, Ph), 6.78 – 6.74 (m, 2H, Ph), 6.09 (dd, J = 10.5, 8.0 Hz, 1H, H_2), 5.58 (s, 1H, PhCH), 5.41 (dd, J = 10.4, 3.6 Hz, 1H,

H_3), 5.19 (d, J = 8.0 Hz, 1H, H_1), 4.64 (d, J = 2.9 Hz, 1H, H_4), 4.45 (dd, J = 12.4, 1.5 Hz, 1H, H_{6a}), 4.17 (dd, J = 12.4, 1.7 Hz, 1H, H_{6b}), 3.77 (br s, 1H, H_5), 3.75 (s, 3H, OCH_3); ^{13}C NMR (100 MHz, $CDCl_3$) δ 166.3 (C=O), 165.2 (C=O), 155.7 (C_q), 151.3 (C_q), 137.4 (C_q), 133.4 (CH), 133.2 (CH), 130.0 (CH), 129.7 (CH), 129.6 (C_q), 129.1 (C_q), 129.0 (CH), 128.35 (CH), 128.34 (CH), 128.2 (CH), 126.3 (CH), 119.5 (CH), 114.4 (CH), 101.5 (C_1), 101.0 (PhCH), 73.4 (C_4), 72.8 (C_3), 69.0 (C_6), 68.9 (C_2), 66.7 (C_5), 55.6 (OCH_3). HRMS (ESI$^+$) *m/z* found: $(M+NH_4)^+$ 600.2225, $C_{34}H_{34}NO_9$ requires $(M+NH_4)^+$ 600.2228. NMR data were consistent with literature reported previously.[3]

***p*-Methoxyphenyl 2,3-di-*O*-benzoyl-β-D-galactopyranoside 1**

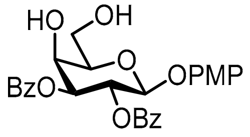

A suspension of *p*-methoxyphenyl 2,3-di-*O*-benzoyl-4,6-*O*-benzylidene-β-D-galactopyranoside **S4** (3.41 g, 5.84 mmol, 1.00 equiv.) and $TsOH·H_2O$ (102 mg, 0.584 mmol, 0.10 equiv.) in MeOH was stirred at reflux at 60 ºC overnight. TLC analysis (CH_2Cl_2) revealed full conversion of the starting material to a lower R_f spot. The reaction was quenched with $NaHCO_3$ (10 mL) and extracted with CH_2Cl_2 (2 x 30 mL). The organic layer was washed with brine (20 mL), dried over anhydrous $MgSO_4$, filtered and concentrated *in vacuo* to furnish a white solid. This crude material was washed with PE/Et_2O (2 x 10 mL, 2/1) to remove residual benzaldehyde dimethyl acetal and to generate title compound **1** as a white solid (2.60 g, 5.26 mmol, 89%). R_f = 0.20 (9/1, CH_2Cl_2/Et_2O); ^1H NMR (400 MHz, $CDCl_3$) δ 8.03 – 7.95 (m, 4H, Ph), 7.56 – 7.48 (m, 2H, Ph), 7.41 – 7.37 (m, 4H, Ph), 6.97 – 6.92 (m, 2H, Ph), 6.80 – 6.75 (m, 2H, Ph), 6.01 (dd, J = 10.3, 8.0 Hz, 1H, H_2), 5.36 (dd, J = 10.3, 3.1 Hz, 1H, H_3), 5.19 (d, J = 8.0 Hz, 1H, H_1), 4.45 (t, J = 3.4 Hz, 1H, H_4), 4.08 (dt, J = 11.7, 5.8 Hz, 1H, H_{6a}), 3.99 (ddd, J = 11.9, 7.6, 4.3 Hz, 1H, H_{6b}), 3.90 – 3.87 (m, 1H, H_5), 3.75 (s, 3H, OCH_3), 2.80 (d, J = 4.2 Hz, 1H, C_4-OH), 2.15 (dd, J = 7.5, 5.6 Hz, 1H, C_6-OH); ^{13}C NMR (100 MHz, $CDCl_3$) δ 165.9 (C=O), 165.4 (C=O), 155.7 (C_q), 151.1 (C_q), 133.6 (CH), 133.3 (CH), 129.9 (CH), 129.8 (CH), 129.4 (C_q), 129.0 (C_q), 128.5 (CH), 128.4 (CH), 118.7 (CH), 114.6 (CH), 101.2 (C_1), 74.5 (C_5), 74.3 (C_3), 69.4 (C_2), 68.4 (C_4), 62.7 (C_6), 55.6 (OCH_3). NMR data were consistent with literature reported previously.[4] HRMS (ESI$^-$) *m/z* found: $(M-H)^-$ 493.1506, $C_{27}H_{25}O_9$ requires 493.1504.

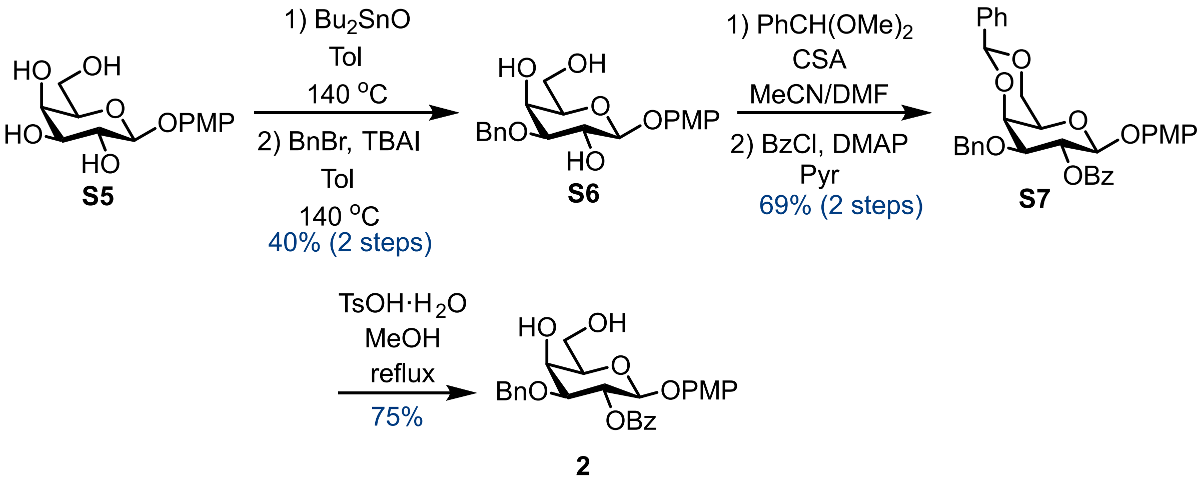

Scheme S2. *Synthesis of galactosyl diol* **2**.

p-Methoxyphenyl 3-*O*-benzyl-D-galactopyranoside S6

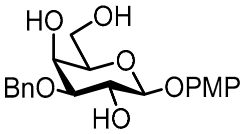

A solution of *p*-methylphenyl-β-D-galactopyranoside **S5** (4.9 g, 17 mmol, 1.00 equiv.) in toluene was treated with dibutyltin oxide (4.4 g, 17 mmol, 1.00 equiv.). The mixture was refluxed with azeotropic removal of the generated water for 14 h. TBAI (6.5 g, 17 mmol, 1.00 equiv.) and BnBr (3.1 mL, 26 mmol, 1.50 equiv.) were then added, and the mixture was stirred at reflux temperature for an additional 2 h. TLC analysis (4/1, CH_2Cl_2/acetone) revealed complete conversion of starting material to three higher R_f spots. The mixture was concentrated *in vacuo* to give a brown syrup which was purified via column chromatography (1/0 → 8:2, CH_2Cl_2/acetone) to generate title compound **S6** as a white solid (2.56 g, 6.80 mol, 40%), contaminated with an unknown co-eluting compound. A portion of **S6** was recrystallised from (1/1, MeOH/Et_2O) to yield a pure sample. R_f = 0.6 (4/1, CH_2Cl_2/acetone); ^{1}H NMR (400 MHz, CD_3OD) δ 7.49 – 7.46 (m, 2H, Ph), 7.37 – 7.32 (m, 2H, Ph), 7.31 – 7.25 (m, 1H, Ph), 7.09 – 7.04 (m, 2H, Ph), 6.87 – 6.81 (m, 2H, Ph), 4.81 (d, *J* = 11.7 Hz, 1H, PhCH*H*), 4.76 (d, *J* = 7.8 Hz, 1H, H_1), 4.72 (d, *J* = 11.8 Hz, 1H, PhC*H*H), 4.11 (dd, *J* = 3.2, 0.6 Hz, 1H, H_4), 3.93 (dd, *J* = 9.7, 7.8 Hz, 1H, H_2), 3.80 – 3.76 (m, 2H, H_{6a}, H_{6b}), 3.76 (s, 3H, OCH_3), 3.59 (ddd, *J* = 6.6, 5.5, 1.0 Hz, 1H, H_5), 3.48 (dd, *J* = 9.7, 3.3 Hz, 1H, H_3); ^{13}C NMR (100 MHz, CD_3OD) δ 156.6 (C_q), 153.2 (C_q), 139.8 (C_q), 129.3 (CH), 129.1 (CH), 128.6 (CH), 119.3 (CH), 115.4 (CH), 104.0 (C_1), 82.3 (C_3), 76.7 (C_5), 72.6 ($PhCH_2$), 71.6 (C_2), 67.1 (C_4), 62.4 (C_6), 56.0 (OCH_3); HRMS (ESI$^+$) *m/z* found: $(M+Na)^+$ 399.1408, $C_{20}H_{24}O_7Na$ requires 399.1414. NMR data was consistent with literature data.[5]

p-Methoxylphenyl 2-*O*-benzoyl-3-*O*-benzyl-4,6-*O*-benzylidene-β-D-galactopyranoside S7

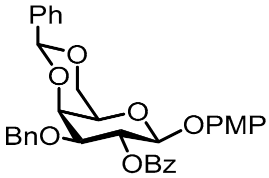

A solution of *p*-methoxyphenyl 3-*O*-benzyl-D-galactopyranoside **S6** (1.1 g, 2.9 mmol, 1.00 equiv.) in MeCN/DMF (40 mL, 3.5/1) was treated with $PhCH(OMe)_2$ (0.88 mL, 5.8 mmol, 2.00 equiv.) and CSA (10 mg, 0.41 mmol, 0.14 equiv.). The reaction was left to stir for 1 h at 60 °C, slowly forming a precipitate. TLC (9/1, CH_2Cl_2/Et_2O) revealed complete conversion of the starting material to a higher R_f spot. The reaction mixture was filtered, and the residue was dried under high vacuum to yield a white

solid (1.20 g, 2.58 mmol, 89%). R_f = 0.84 (9:1, CH_2Cl_2/Et_2O). A solution of this solid and DMAP (35 mg, 0.29 mmol, 0.10 equiv.) in pyridine (15 mL) was treated with BzCl (0.68 mL, 5.8 mmol, 2.00 equiv.) at 0 ºC. The reaction was stirred at RT for 2 h where it was diluted with CH_2Cl_2 (15 mL) and the washed with $NaHCO_3$ (20 mL). The aqueous layer was separated and extracted with CH_2Cl_2 (2 x 30 mL). The organic layers were combined, washed with brine (50 mL), dried over anhydrous $MgSO_4$, filtered and concentrated *in vacuo* to yield a yellow solid. This crude material was washed with cold MeOH (2 x 10 mL) and dried under high vacuum to generate title compound **S7** as a white solid (1.15 g, 2.02 mmol, 69% (over two steps)). R_f = 0.31 (CH_2Cl_2); m.p. 141 – 143 °C; $[\alpha]^{25}_D$ +23.3 (c = 0.67, $CHCl_3$); ^{1}H NMR (400 MHz, $CDCl_3$) δ 8.07 – 8.02 (m, 2H, Ph), 7.59 – 7.57 (m, 3H, Ph), 7.50 – 7.43 (m, 2H, Ph), 7.42 – 7.34 (m, 3H, Ph), 7.26 – 7.15 (m, 5H, Ph), 6.95 – 6.90 (m, 2H, Ph), 6.75 – 6.70 (m, 2H, Ph), 5.85 (dd, *J* = 10.1, 8.0 Hz, 1H, H_2), 5.01 (d, *J* = 8.0 Hz, 1H, H_1), 4.71 (d, *J* = 12.8 Hz, 1H, PhC*H*H), 4.63 (d, *J* = 12.8 Hz, 1H, PhCH*H*), 4.39 (dd, *J* = 12.3, 1.5 Hz, 1H, H_{6a}), 4.28 (d, *J* = 2.9 Hz, 1H, H_4), 4.09 (dd, *J* = 12.4, 1.7 Hz, 1H, H_{6b}), 3.80 (dd, *J* = 10.1, 3.5 Hz, 1H, H_3), 3.72 (s, OCH_3), 3.51 (d, *J* = 1.0 Hz, 1H, H_5); ^{13}C NMR (100 MHz, $CDCl_3$) δ 165.2 (C=O), 155.5 (C_q), 151.5 (C_q), 137.8 (C_q), 137.6 (C_q), 133.0 (CH), 130.1 (C_q), 129.8 (CH), 129.0 (CH), 128.4 (CH), 128.3 (CH), 128.2 (CH), 127.8 (CH), 127.7 (CH), 126.5 (CH), 119.3 (CH), 114.3 (CH), 101.5 (C_1), 101.3 (PhCH), 76.9 (C_3), 73.0 (C_4), 71.0 ($PhCH_2$), 70.7 (C_2), 69.1 (C_6), 66.9 (C_5), 55.6 (OCH_3); HRMS (ESI$^+$) *m/z* found: $(M+Na)^+$ 591.1986, $C_{34}H_{32}O_8Na$ requires 591.1989.

***p*-Methoxylphenyl 2-*O*-benzoyl-3-*O*-benzyl-β-D-galactopyranoside 2**

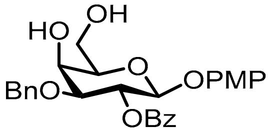

A suspension of *p*-methoxyphenyl 2-*O*-benzoyl-3-*O*-benzyl-4,6-*O*-benzylidene-β-D-galactopyranoside **7** (5.63 g, 9.90 mmol, 1.00 equiv.) and $TsOH{\cdot}H_2O$ (170 mg, 0.99 mmol, 0.10 equiv.) in MeOH was stirred at reflux for 3 h. TLC analysis (CH_2Cl_2) revealed almost full conversion of the starting material to a lower spot. The reaction was quenched with $NaHCO_3$ (15 mL) and cooled to 0 ºC to form white crystals which were collected *via* filtration. The filtrate was reduced in volume *in vacuo* and cooled to 0 ºC to furnish another crop of white crystals. Both batches of crystals were dissolved in toluene and concentrated *in vacuo* to facilitate the azeotropic removal of residual water. Product di-ol **2** was obtained as a white foam (3.56 g, 7.41 mmol, 75%). R_f = 0.15 (9/1, CH_2Cl_2/Et_2O); ^{1}H NMR (400 MHz, $CDCl_3$) δ 8.04 – 7.99 (m, 2H, Ph), 7.63 – 7.56 (m, 2H, Ph), 7.46 – 7.44 (m, 2H, Ph), 7.22 – 7.14 (m, 4H, Ph), 6.91 – 6.85 (m, 2H, Ph), 6.76 – 6.71 (m, 2H, Ph), 5.70 (dd, *J* = 9.7, 8.1 Hz, 1H, H_2), 4.97 (d, *J* = 8.0 Hz, 1H, H_1), 4.70 (d, *J* = 12.3 Hz, 1H, PhC*H*H), 4.56 (d, *J* = 12.3 Hz, 1H, PhCH*H*), 4.16 – 4.13 (m, 1H, H_4), 4.07 (ddd, *J* = 11.5, 7.0, 4.2 Hz, 1H, H_{6a}), 3.89 (ddd, *J* = 11.8, 8.8, 4.6 Hz, 1H, H_{6b}), 3.73 (dd, *J* = 9.7, 3.4 Hz, 1H, H_3), 3.72 (s, 3H, OCH_3), 3.69 – 3.65 (m, 1H, H_5), 2.76 (t, *J* = 1.4 Hz, 1H, 4-OH), 2.12 (dd, *J* = 8.8, 4.2 Hz, 1H, 6-OH); ^{13}C NMR (100 MHz, $CDCl_3$) δ 165.4 (C=O), 155.5 (C_q), 151.2 (C_q), 136.9 (C_q), 133.2 (CH), 129.8 (CH), 128.5 (CH), 128.4 (CH), 128.1 (C_q), 127.9 (CH), 118.6 (CH), 114.5 (CH), 100.8 (C_1), 78.1 (C_3), 74.8 (C_5), 71.6 ($PhCH_2$), 71.0 (C_2), 66.6 (C_4), 62.3 (C_6), 55.6 (OCH_3).

Synthesis of Glucosamine Donors

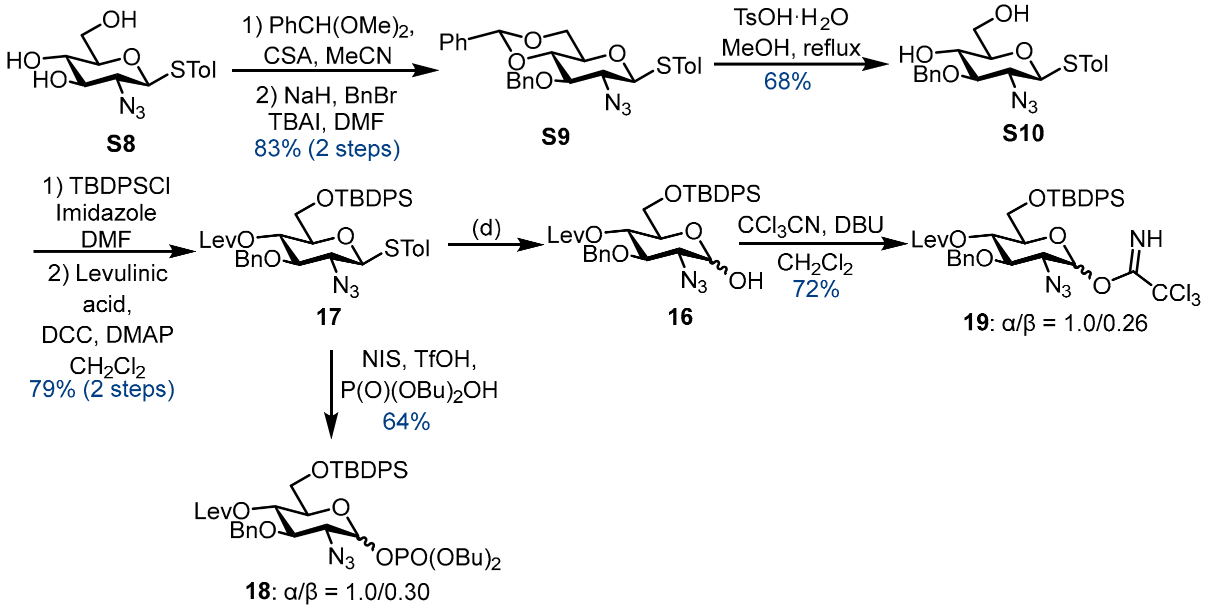

Scheme S3. *Synthesis of glucosamine donors **16**, **17**, **18** and **19**.*

p-Methylphenyl 2-azido-3-*O*-benzyl-4,6-*O*-benzylidene-2-deoxy-1-thio-β-D-glucopyranoside S9

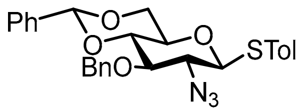

A solution of *p*-methylphenyl 2-azido-2-deoxy-1-thio-β-D-glucopyranoside **S8** (7.04 g, 22.5 mmol, 1.00 equiv.) in MeCN (110 mL) was treated with $PhCH(OMe)_2$ (6.77 mL, 45.0 mmol, 2.00 equiv.) and CSA (0.732 g, 3.15 mmol, 0.14 equiv.). The reaction was stirred at RT for 6 h whereupon TLC analysis (95/5, CH_2Cl_2/Et_2O) revealed full conversion of the starting material to a higher R_f spot. The reaction mixture was quenched with $NaHCO_3$ (40 mL) and diluted with CH_2Cl_2 (60 mL). The layers were separated, and the aqueous layer was extracted with CH_2Cl_2 (2 x 50 mL). The organic layers were combined, washed with brine (80 mL), dried over anhydrous $MgSO_4$, filtered and concentrated *in vacuo* to furnish an off-white solid, which was not purified before moving on to the next step. A solution of the crude benzylidene product (5.45 g, 13.6 mmol, 1.00 equiv.) and TBAI (0.500 g, 1.36 mmol, 0.10 equiv.) in anhydrous DMF (60 mL) was treated with NaH (60%, 0.650 g, 16.4 mmol, 1.20 equiv.) at 0 ºC. The reaction was stirred at RT for 30 min, followed by the dropwise addition of BnBr (3.24 mL, 27.3 mmol, 2.00 equiv.) at 0 ºC, where the reaction was stirred at RT for an additional 2 h. TLC analysis (CH_2Cl_2) revealed almost full conversion of the starting material to a higher R_f spot. The reaction was quenched with MeOH (15 mL) and diluted with CH_2Cl_2 (60 mL). The organic layer was washed with brine (50 mL), dried over anhydrous $MgSO_4$, filtered and concentrated *in vacuo* to furnish a yellow solid. This crude material was recrystalised from hot MeOH (25 ml) to generate title compound **S9** as a white solid (7.16 g, 14.6 mmol, 65% (over two steps)); R_f = 0.95 (CH_2Cl_2); $[\alpha]^{23}_D$ −8.4 (c = 0.55, $CHCl_3$); ^1H NMR (400 MHz, $CDCl_3$) δ 7.47 – 7.44 (m, 4H, Ph), 7.41 – 7.25 (m, 8H, Ph), 7.16 – 7.15 (m, 2H, Ph), 5.55 (s, 1H, PhCH), 4.90 (d, J = 10.9 Hz, 1H, PhC*H*H), 4.77 (d, J = 10.9 Hz, 1H, PhCH*H*), 4.42 (d, J = 10.2 Hz, 1H, H_1), 4.38 (dd, J = 10.6, 5.0 Hz, 1H, H_{6a}), 3.76 (t, J = 10.7 Hz, 1H, H_{6b}), 3.67 – 3.62 (m, 1H, H_4), 3.60 (t, J = 9.1 Hz, 1H, H_2), 3.43 (td, J = 9.7, 5.0 Hz, 1H, H_5), 3.32 (dd, J = 10.1, 8.7 Hz, 1H, H_3), 2.35 (s, 3H, CH_3); ^{13}C NMR (100 MHz, $CDCl_3$) δ 139.2 (C_q), 137.6 (C_q), 137.1 (C_q), 134.6 (CH), 129.9 (CH), 129.1 (CH), 128.5 (CH), 128.4 (CH), 128.3 (CH), 128.0 (CH), 126.5 (C_q), 126.0 (CH), 101.2 (PhCH), 86.6 (C_1), 81.3 (C_2), 81.0 (C_4), 75.2 ($PhCH_2$), 70.5 (C_5), 68.5 (C_6), 64.5 (C_3), 21.2 (CH_3). NMR data were consistent with literature reported previously.[6] HRMS (ESI^+) *m/z* found: $(M+H)^+$ 490.1800, $C_{27}H_{28}N_3O_4S$ requires 490.1801.

***p*-Methylphenyl 2-azido-3-*O*-benzyl-2-deoxy-1-thio-β-D-glucopyranoside S10**

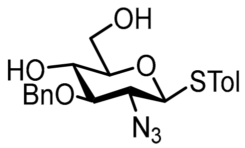

A solution of *p*-methylphenyl 2-azido-3-*O*-benzyl-4,6-*O*-benzylidene-2-deoxy-1-thio-β-D-glucopyranoside **S9** (6.70 g, 13.7 mmol, 1.00 equiv.) in MeOH (120 mL) was treated with TsOH·H_2O (228 mg, 1.36 mmol, 0.10 equiv.) and stirred at reflux at 80 ºC for 2 h. TLC analysis (CH_2Cl_2) revealed full conversion of the starting material to a lower R_f spot. The reaction was quenched with $NaHCO_3$ (20 mL) and diluted with CH_2Cl_2 (90 mL). The aqueous layer was separated and extracted with CH_2Cl_2 (2 x 50 mL). The organic layers were combined, washed with brine (150 mL), dried over anhydrous $MgSO_4$, filtered and concentrated *in vacuo* to give a yellow oil. Purification of this crude material *via* column chromatography (1/0 → 9:1, CH_2Cl_2/Et_2O) generated title compound **S10** as a white solid (3.75 g, 9.34 mmol, 68%). R_f = 0.22 (95:5 CH_2Cl_2/Et_2O); ^{1}H NMR (400 MHz, $CDCl_3$) δ 7.44 (d, *J* = 8.1 Hz, 2H, Ph), 7.39 – 7.30 (m, 5H, Ph), 7.14 (d, *J* = 7.9 Hz, 2H, Ph), 4.94 (d, *J* = 11.3 Hz, 1H, PhC*H*H), 4.76 (d, *J* = 11.3 Hz, 1H, PhCH*H*), 4.41 (d, *J* = 9.9 Hz, 1H, H_1), 3.87 (ddd, *J* = 11.5, 5.8, 3.6 Hz, 1H, H_{6a}), 3.81 – 3.74 (m, 1H, H_{6b}), 3.54 (td, *J* = 9.3, 3.1 Hz, 1H, H_4), 3.34 – 3.29 (m, 3H, H_2, H_3 and H_5), 2.55 – 2.53 (m 1H, 4-OH), 2.35 (s, 3H, CH_3), 2.18 (t, *J* = 6.5 Hz, 1H, 6-OH); ^{13}C NMR (100 MHz, $CDCl_3$) δ 138.9 (C_q), 137.8 (C_q), 133.9 (CH), 130.0 (CH), 128.74 (CH), 128.3 (CH), 128.2 (CH), 127.2 (C_q), 86.5 (C_1), 84.7 (C_4), 79.4 (C_2), 75.5 ($PhCH_2$), 70.1 (C_3), 64.8 (C_5), 62.3 (C_6), 21.2 (CH_3). NMR data was consistent with literature reported previously.[7] HRMS (ESI^+) *m/z* found: $(M+Na)^+$ 424.1297, $C_{20}H_{23}N_3O_4SNa$ requires 424.1301.

***p*-Methylphenyl 2-azido-3-*O*-benzyl-2-deoxy-4-*O*-levulinoyl-6-*O*-(*tert*-butyldiphenylsilyl)-1-thio-β-D-glucopyranoside 17**

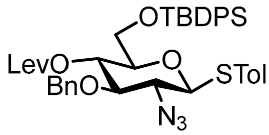

A solution of *p*-methylphenyl 2-azido-3-*O*-benzyl-2-deoxy-1-thio-β-D-glucopyranoside **S10** (495 mg, 1.25 mmol, 1.00 equiv.) and imidazole (255 mg, 3.75 mmol, 3.00 equiv.) in DMF (2.5 mL) was treated with TBDPSCl (0.490 mL, 1.87 mmol, 1.50 equiv.) and left to stir at room temperature for 1 h. The reaction mixture was diluted with Et_2O (150 mL) and washed with water (100 mL) and brine (50 mL), dried over anhydrous $MgSO_4$, filtered and concentrated *in vacuo* to give a yellowish syrup. A solution of this crude material in CH_2Cl_2 (7.5 mL) was treated with LevOH (290 mg, 2.5 mmol, 2.00 equiv.), DCC (0.770 g, 3.8 mmol, 3.00 equiv.) and DMAP (19 mg, 0.13 mmol, 0.10 equiv.) and the resulting suspension was sonicated for 15 min. TLC analysis (2/1, hexane/EtOAc) revealed full conversion of the starting material to a lower R_f spot. The reaction was diluted with Et_2O (150 mL) and filtered to remove the thiourea formed *in-situ*. The filtrate was washed with $NaHCO_3$ (100 mL) and brine (50 mL), dried over anhydrous $MgSO_4$, filtered and concentrated *in vacuo* to generate a brown syrup. Purification of this crude material *via* column chromatography (95/5 → 8:2, hexane/EtOAc) generated title compound **17** as a white solid (0.73 g, 0.99 mmol, 79% (over 2 steps)). R_f = 0.20 (2/1, hexane/EtOAc); m.p. 108 – 111 °C; $[\alpha]^{25}_D$ +67.3 (c = 0.50, $CHCl_3$); ^{1}H NMR (400 MHz, $CDCl_3$) δ 7.76 – 7.65 (m, 4H, Ph), 7.50 – 7.48 (m, 2H, Ph), 7.44 – 7.26 (m, 11H, Ph), 7.00 – 6.99 (m, 2H, Ph), 5.06 (t, *J* = 9.7 Hz, 1H, H_4), 4.77 (d, *J* = 11.1 Hz, 1H, PhCH*H*), 4.64 (d, *J* = 11.1 Hz, 1H, PhC*H*H), 4.37 (d, *J* = 10.1 Hz, 1H,

H_1), 3.76 (dd, *J* = 11.6, 1.9 Hz, 1H, H_{6a}), 3.68 (dd, *J* = 11.6, 4.7 Hz, 1H, H_{6b}), 3.49 (t, *J* = 9.3 Hz, 1H, H_3), 3.44 (ddd, *J* = 10.0, 4.6, 1.9 Hz, 1H, H_5), 3.39 – 3.33 (m, 1H, H_2), 2.56 (t, *J* = 6.7 Hz, 2H, Lev-CH_2), 2.33 – 2.28 (m, 5H, Lev-CH_2, Lev-CH_3), 2.11 (s, 3H, CH_3), 1.05 (s, 9H, $SiC(CH_3)_3$). ^{13}C NMR (100 MHz, $CDCl_3$) δ 205.9 (C=O), 171.1 (C=O), 138.7 (C_q), 137.5 (C_q), 135.7 (CH), 134.2 (CH), 133.23 (C_q), 133.15 (C_q), 129.8 (CH), 129.7 (CH), 129.6 (CH), 128.4 (CH), 128.2 (CH), 127.9 (CH), 127.7 (CH), 126.9 (C_q), 86.0 (C_1), 82.8 (C_3), 79.0 (C_5), 75.3 ($PhCH_2$), 69.7 (C_4), 64.5 (C_2), 62.5 (C_6), 37.7 (Lev-CH_2), 29.8 (Lev-CH_3), 27.8 (Lev-CH_2), 26.7 ($SiC(\mathit{C}H_3)_3$), 21.2 (CH_3), 19.2 ($Si\mathit{C}(CH_3)_3$); (ESI$^+$) *m/z* found: $(M+NH_4)^+$ 755.3296, $C_{41}H_{51}N_4O_6SSi$ requires 755.3293.

2-Azido-3-*O*-benzyl-2-deoxy-4-*O*-levulinoyl-6-*O*-(*tert*-butyldiphenylsilyl)-β-D-glucopyranose 16

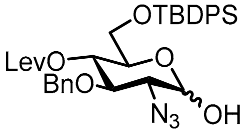

A solution of *p*-methylphenyl 2-azido-3-*O*-benzyl-2-deoxy-4-*O*-levulinoyl-6-*O*-(*tert*-butyldiphenylsilyl)-1-thio-β-D-glucopyranoside **17** (2.02 g, 2.71 mmol, 1.00 equiv.) and NIS (608 mg, 2.71 mmol, 1.00 equiv.) in CH_2Cl_2/water (27 mL, 10/1) was cooled to 0 °C and treated with TFA (210 µL, 2.7 mmol, 1.00 equiv.) and left stirring at the same temperature for 4 h. TLC analysis (99/1, CH_2Cl_2/Et_2O) revealed almost full conversion of the starting material to a lower R_f spot. The reaction was quenched with $Na_2S_2O_3$ (10 mL) and diluted with CH_2Cl_2 (10 mL). The organic layer was washed with $NaHCO_3$ (10 mL) and brine (10 mL), dried over anhydrous $MgSO_4$, filtered and concentrated *in vacuo* to generate a yellow syrup. Purification of this crude material *via* column chromatography (1/0 → 95:5, CH_2Cl_2/Et_2O) afforded the title compound **16** as a white solid (1.03 g, 1.63 mmol, 60%). R_f = 0.23 (99/1, CH_2Cl_2/Et_2O).

The following were observed for α and β anomers: ^{1}H NMR (400 MHz, $CDCl_3$) δ 7.61 – 7.58 (m, 4H, Ph), 7.38 – 7.20 (m, 11H, Ph), 3.67 – 3.59 (m, 2H, H_{6a}, H_{6b}), 2.51 (t, *J* = 6.8 Hz, 2H, Lev-CH_2), 2.30 – 2.22 (m, 2H, Lev-CH_2); ^{13}C NMR (100 MHz, $CDCl_3$) δ 134.9 (CH), 134.8 (CH), 134.66 (CH), 134.65 (CH), 133.0 (C_q), 132.6 (C_q), 132.4 (C_q), 132.2 (C_q), 128.7 (CH), 128.63 (CH), 128.61 (CH), 128.58 (CH), 127.40 (CH), 127.38 (CH), 127.1 (CH), 127.0 (CH), 126.8 (CH), 126.6 (CH), 126.54 (CH), 126.50 (CH), 74.1 ($PhCH_2$).

α-anomer

^{1}H NMR (400 MHz, $CDCl_3$) δ 5.21 (d, *J* = 3.4 Hz, 1H, H_1), 5.08 (dd, *J* = 10.1, 9.3 Hz, 1H, H_4), 4.72 (d, *J* = 11.1 Hz, 1H, PhC*H*H), 4.56 (d, *J* = 11.1 Hz, 1H, PhC*H*H), 3.95 – 3.88 (m, 1H, H_5), 3.91 (t, *J* = 9.7 Hz, 1H, H_3), 3.65 – 3.61 (m, 1H, H_2), 2.65 (dd, *J* = 3.3, 1.3 Hz, 1H, OH), 2.06 (s, 3H, Lev-CH_3), 0.97 (s, 9H, $SiC(CH_3)_3$); ^{13}C NMR (100 MHz, $CDCl_3$) δ 205.22 (C=O), 170.24 (C=O), 136.64 (C_q), 90.9 (C_1), 76.7 (C_3), 69.8 (C_5), 69.1 (C_4), 62.6 (C_2), 61.8 (C_6), 36.65 (Lev-CH_2), 28.82 (Lev-CH_3), 26.7 (Lev-CH_2), 25.80 ($SiC(\mathit{C}H_3)_3$), 18.2 ($Si\mathit{C}(CH_3)_3$).

β-anomer

^{1}H NMR (400 MHz, $CDCl_3$) δ 4.93 (dd, *J* = 10.0, 8.9 Hz, 1H, H_4), 4.72 (d, *J* = 11.3 Hz, 1H, PhC*H*H), 4.56 (d, *J* = 11.1 Hz, 1H, PhCH*H*), 4.39 (dd, *J* = 7.7, 5.2 Hz, 1H, H_1), 3.38 – 3.28 (m, 3H, H_5, H_3, H_2), 2.84 (d, *J* = 5.2 Hz, 1H, OH), 2.05 (s, 3H, Lev-CH_3), 0.96 (s, 9H, $SiC(CH_3)_3$); ^{13}C NMR (100 MHz, $CDCl_3$) δ 205.18 (C=O), 170.29 (C=O), 136.67 (C_q), 95.0 (C_1), 79.5 (C_3), 73.8 (C_5), 69.5 (C_4), 66.21 (C_2), 62.18 (C_6), 36.72 (Lev-$\mathit{C}H_2$), 27.9 (Lev-CH_3), 26.80 (Lev-$\mathit{C}H_2$), 25.78 ($SiC(\mathit{C}H_3)_3$), 18.3 ($Si\mathit{C}(CH_3)_3$),

HRMS (ESI$^+$) *m/z* found: $(M+NH_4)^+$ 649.3043, $C_{34}H_{45}N_4O_7Si$, requires 649.3052.

2-Azido-3-*O*-benzyl-2-deoxy-4-*O*-levulinoyl-6-*O*-(*tert*-butyldiphenylsilyl)-β-D-glucopyranosyl trichloroacetamidate 19

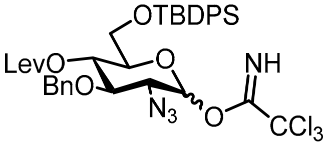

A solution of 2-azido-3-*O*-benzyl-2-deoxy-4-*O*-levulinoyl-6-*O*-(*tert*-butyldiphenylsilyl)-α/β-D-glucopyranose **16** (0.85 g, 1.3 mmol, 1.00 equiv.) in CH_2Cl_2 was treated with CCl_3CN (0.55 mL, 5.5 mmol, 4.00 equiv.) and DBU (60 µL, 0.4 mmol, 0.30 equiv.) and stirred for 1 h at RT. TLC analysis (CH_2Cl_2) revealed full conversion of starting material to a higher R_f spot. The reaction mixture was concentrated *in-vacuo* and purified *via* column chromatography (CH_2Cl_2, 0.5% Et_3N) to generate title compound **19** as a colourless syrup (0.72 g, 0.93 mmol, 72%). R_f = 0.55 (CH_2Cl_2). α/β = 1.00:0.26.

The following were observed for α and β anomers: ^{1}H NMR (400 MHz, $CDCl_3$) δ 7.70 – 7.60 (m, 5H, Ph), 7.44 – 7.27 (m, 10H, Ph), 2.62 – 2.56 (m, 2H, Lev-CH_2), 2.36 (t, *J* = 6.5 Hz, 2H, Lev-CH_2), 1.05 – 0.99 (m, 9H, $SiC(CH_3)_3$). ^{13}C NMR (100 MHz, $CDCl_3$) δ 135.78 (CH), 135.76 (CH), 135.72 (CH), 135.68 (CH), 133.33 (C_q), 133.31 (C_q), 133.2 (CH), 129.7 (CH), 129.62 (CH), 129.60 (CH), 129.56 (CH), 128.51 (CH), 128.46 (CH), 128.2 (CH), 128.1 (C_q), 127.96 (C_q), 127.95 (CH), 127.62 (CH), 127.61 (CH), 127.57 (CH), 37.7 (Lev-CH_2), 29.8 (Lev-CH_3), 27.8 (Lev-CH_2), 26.7 ($SiC(\mathit{C}H_3)_3$), 19.3 ($Si\mathit{C}(CH_3)_3$).
α anomer
^{1}H NMR (400 MHz, $CDCl_3$) δ 8.74 (s, 1H, NH), 6.47 (d, *J* = 3.5 Hz, 1H, H_1), 5.28 – 5.24 (m, 1H, H_4), 4.82 (d, *J* = 11.0 Hz, 1H, PhCH*H*), 4.72 (d, *J* = 11.0 Hz, 1H, PhC*H*H), 4.07 – 4.01 (m, 1H, H_3), 4.00 (dt, *J* = 10.3, 3.3 Hz, 1H, H_5), 3.76 (m, 1H, H_2), 3.74 – 3.67 (m, 2H, H_{6a}, H_{6b}), 2.12 (s, 3H, Lev-CH_3); ^{13}C NMR (100 MHz, $CDCl_3$) δ 205.89 (C=NH), 171.3 (C=O), 160.5 (C=O), 137.5 (C_q), 94.5 (C_1), 91.0 (CCl_3), 77.8 (C_3), 74.9 ($PhCH_2$), 73.6 (C_5), 70.0 (C_4), 62.8 (C_2), 62.4 (C_6).

β anomer
^{1}H NMR (400 MHz, $CDCl_3$) δ 8.76 (s, 1H, NH), 5.71 (d, *J* = 8.4 Hz, 1H, H_1), 5.25 (dd, *J* = 10.2, 9.3 Hz, 1H, H_4), 4.83 (d, *J* = 11.4 Hz, 1H, PhCH*H*), 4.71 (d, *J* = 11.4 Hz, 1H, PhC*H*H), 3.76 – 3.66 (m, 3H, H_2, H_{6a}, H_{6b}), 3.59 (ddd, *J* = 6.5, 4.3, 1.7 Hz, 1H, H_5), 3.58 – 3.53 (m, 1H, H_3), 2.13 (s, 3H, Lev-CH_3). ^{13}C NMR (100 MHz, $CDCl_3$) δ 205.93 (C=NH), 171.2 (C=O), 160.9 (C=O), 137.6 (C_q), 96.5 (C_1), 90.6 (CCl_3), 80.6 (C_3), 75.6 (C_5), 75.0 ($PhCH_2$), 69.5 (C_4), 65.5 (C_2), 62.1 (C_6).

HRMS (ESI$^+$) *m/z* found: $(M+Na)^+$ 799.1679, $C_{36}H_{41}Cl_3N_4O_7SiNa$ requires $(M+Na)^+$ 799.1679.

Ester Migrated Side Products

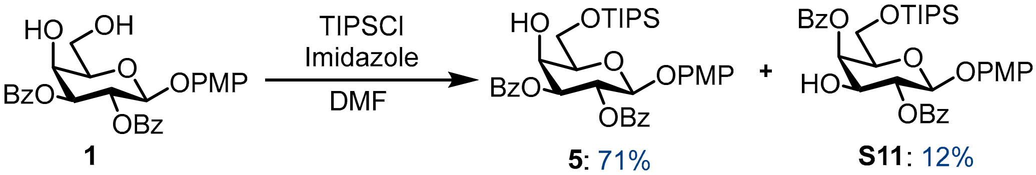

***p*-Methoxyphenyl 2,4-di-*O*-benzoyl-6-*O*-(triisopropylsilyl)-β-D-galactopyranoside S11**

S11 was obtained as a white solid (322 mg, 0.495 mmol, 12%). R_f = 0.75 (2/1, hexane/EtOAc); m.p. 137 – 140 °C; $[\alpha]^{25}_D$ +8.3 (c = 0.50, $CHCl_3$); ^{1}H NMR (400 MHz, $CDCl_3$) δ 8.18 – 8.13 (m, 2H, Ph), 8.07 – 8.03 (m, 2H, Ph), 7.64 – 7.55 (m, 2H, Ph), 7.51 – 7.42 (m, 4H, Ph), 7.03 – 6.98 (m, 2H, Ph), 6.81 – 6.76 (m, 2H, Ph), 5.75 (d, *J* = 3.1 Hz, 1H, H_4), 5.56 (dd, *J* = 10.0, 7.9 Hz, 1H, H_2), 5.14 (d, *J* = 7.9 Hz, 1H, H_1), 4.20 (ddd, *J* = 9.8, 5.5, 3.6 Hz, 1H, H_3), 3.99 – 3.91 (m, 3H, H_5, H_{6a}, H_{6b}), 3.76 (s, 3H, OCH_3), 2.80 (d, *J* = 5.5 Hz, 1H, 3-OH), 1.11 – 0.97 (m, 21H, $Si(C_3H_7)_3$); ^{13}C NMR (100 MHz, $CDCl_3$) δ 166.8 (C=O), 166.7 (C=O), 155.7 (C_q), 151.6 (C_q), 133.6 (CH), 133.5 (CH), 130.2 (CH), 130.0 (CH), 129.7 (C_q), 129.5 (C_q), 128.64 (CH), 128.57 (CH), 118.8 (CH), 114.6 (CH), 101.1 (C_1), 75.1 (C_3), 73.8

(C_2), 72.3 (C_5), 70.6 (C_4), 62.0 (C_6), 55.7 (OCH_3), 18.0 ($Si(CH(\mathit{C}H_3)_2)_3$), 12.0 ($Si(\mathit{C}H(CH_3)_2)_3$); HRMS ($ESI^+$) *m/z* found: $(M+H)^+$ 651.2982, $C_{36}H_{47}O_9Si$ requires 651.2989.

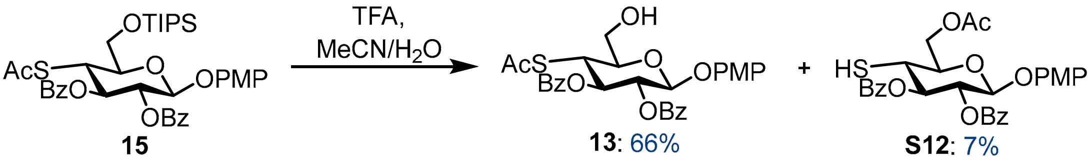

***p*-Methoxyphenyl 6-*O*-acetyl-2,3-di-*O*-benzoyl-4-thio-β-D-glucopyranoside S12**

S12 was obtained as a white solid (103 mg, 0.19 mmol, 7%). R_f = 0.61 (7/3 hexane/EtOAc); m.p. 196 – 198 °C; $[\alpha]^{25}_{D}$ +125.6 (c = 0.50, $CHCl_3$); ^1H NMR (400 MHz, $CDCl_3$) δ 8.02 – 7.99 (m, 2H, Ph), 7.94 – 7.91 (m, 2H, Ph), 7.57 – 7.48 (m, 2H, Ph), 7.43 – 7.33 (m, 4H, Ph), 6.95 – 6.89 (m, 2H, Ph), 6.79 – 6.73 (m, 2H, Ph), 5.61 – 5.48 (m, 2H, H_2, H_3), 5.12 (d, J = 7.6 Hz, 1H, H_1), 4.64 (dd, J = 12.0, 2.1 Hz, 1H, H_{6a}), 4.51 (dd, J = 12.1, 5.3 Hz, 1H, H_{6b}), 3.82 (ddd, J = 10.6, 5.3, 2.2 Hz, 1H, H_5), 3.75 (s, 3H, OCH_3), 3.24 (t, J = 10.4 Hz, 1H, H_4), 2.14 (s, 3H, CH_3), 1.63 (d, J = 9.3 Hz, 1H, SH); ^{13}C NMR (100 MHz, $CDCl_3$) δ 170.7 (C=O), 166.0 (C=O), 165.2 (C=O), 155.8 (C_q), 151.1 (C_q), 133.4 (CH), 133.3 (CH), 129.9 (CH), 129.8 (CH), 129.2 (C_q), 129.0 (C_q), 128.44 (CH), 128.41 (CH), 119.1 (CH), 114.5 (CH), 100.9 (C_1), 76.1 (C_5), 75.4 (C_3), 72.6 (C_2), 63.6 (C_6), 55.6 (OCH_3), 40.8 (C_4), 20.9 (CH_3); HRMS (ESI^+) *m/z* found: $(M+NH_4)^+$ 570.1874, $C_{29}H_{32}NO_9S$ requires 570.1792.

S-Glycosylation Side Products

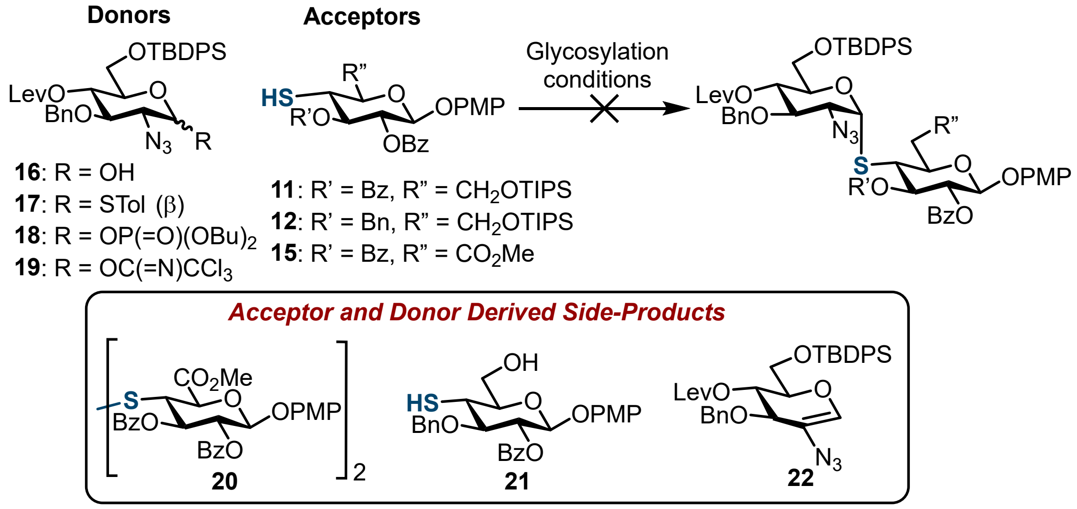

General Procedure SA: Ph_2SO/Tf_2O Promoter System using Glucosamine Donor

In a multi-neck RBF, freshly activated 4Å molecular sieves, diphenyl sulfoxide (2.80 equiv.) and TTBP (3.00 equiv.) were placed under 3 cycles of vacuum and N_2. A stock solution of the glucosamine donor (0.05 M, 1.00 equiv.) in anhydrous CH_2Cl_2 was then added to the flask. The reagents were pre-dried under a N_2 atmosphere for 30 min. The solution was cooled down to −65 °C (using $CHCl_3$ and dry ice) and then treated with triflic anhydride (1.40 equiv.). The solution was allowed to gradually warm up to −48 °C over a period of 1 h. A stock solution of the glucuronic acceptor in anhydrous CH_2Cl_2 (0.3 M, 2.00 equiv.) was then added to the flask at −65 °C. Once the $CHCl_3$/dry ice cooling bath warms up to -48 °C, the cooling bath was replaced with a MeCN/dry ice cooling bath. The reaction mixture was gradually warmed up to −10 °C. After 8 h (i.e. after addition of the acceptor), the reaction mixture was filtered and washed with H_2O (5 mL) and $NaHCO_3$ (5 mL). The organic layer was dried over anhydrous $MgSO_4$, filtered and concentrated in vacuo to give a yellowish paste.

Bis (Methyl (*p*-methoxyphenyl 2,3-di-*O*-benzoyl-4-thio-β-D-glucopyranosid)uronate)-4,4'-disulfide 20

Following general procedure SA, 2-azido-3-*O*-benzyl-2-deoxy-4-*O*-levulinoyl-6-*O*-(*tert*-butyldiphenylsilyl)-α/β-D-glucopyranose **16** (100 mg, 0.160 mmol), diphenyl sulfoxide (91.0 mg, 0.448 mmol), TTBP (119 mg, 0.480 mmol) and Tf_2O (37.6 µL, 0.224 mmol) were used with methyl (*p*-methoxyphenyl 2-*O*-benzoyl-3-*O*-benzyl-β-D-glucopyranosid)uronate **15** (163 mg, 0.320 mmol). Purification *via* column chromatography (100/0 → 95/5, CH_2Cl_2/Et_2O) generated title compound **20** as a colourless syrup (69 mg, 64 µmol, 20% (wrt acceptor **15** used)). R_f = 0.49 (2/1, hexane/EtOAc); ^{1}H NMR (400 MHz, $CDCl_3$) δ 8.01 – 7.89 (m, 4H, Ph), 7.54 – 7.48 (m, 1H, Ph), 7.45 – 7.33 (m, 3H, Ph), 7.30 – 7.23 (m, 2H, Ph), 6.92 – 6.86 (m, 2H, Ph), 6.81 – 6.75 (m, 2H, Ph), 5.89 (br s, 1H, H_3), 5.62 (dd, *J* = 9.1, 7.6 Hz, 1H, H_2), 5.08 (br s, 1H, H_1), 4.38 (d, *J* = 10.5 Hz, 1H, H_5), 3.81 (s, 3H, OCH_3), 3.76 (s, 3H, CO_2CH_3), 3.56 (t, *J* = 10.5 Hz, 1H, H_4); ^{13}C NMR (100 MHz, $CDCl_3$) δ 167.3 (C=O), 165.5 (C=O), 165.1 (C=O), 155.8 (C_q), 151.0 (C_q), 133.5 (CH), 133.3 (CH), 129.9 (CH), 129.8 (CH), 129.2 (C_q), 128.8 (C_q), 128.5 (CH), 128.4 (CH), 118.9 (CH), 114.5 (CH), 100.6 (C_1), 74.8 (C_5), 72.6 (C_2), 71.2 (C_3), 55.6 (OCH_3), 53.0 (CH_3), 51.3 (C_4). HRMS (ESI^+) *m/z* found: $(M+NH_4)^+$ 1092.2775, $C_{56}H_{54}NO_{18}S_2$ requires 1092.2777.

***p*-Methoxyphenyl 2-*O*-benzoyl-3-*O*-benzyl-4-thio-β-D-glucopyranoside 21**

Following an adaption of general procedure SA, methylphenyl 2-azido-3-*O*-benzyl-2-deoxy-4-*O*-levulinoyl-6-*O*-(*tert*-butyldiphenylsilyl)-1-thio-β-D-glucopyranose **17** (101 mg, 0.160 mmol), diphenyl sulfoxide (42.2 mg, 0.208 mmol), TTBP (119 mg, 0.480 mmol) and Tf_2O (37.6 µL, 0.224 mmol) were used with *p*-methoxyphenyl 2-*O*-benzoyl-3-*O*-benzyl-6-*O*-(triisopropylsilyl)-4-thio-β-D-glucopyranoside **12** (172 mg, 0.320 mmol). Purification *via* column chromatography (100/0 → 95/5,

CH_2Cl_2/Et_2O)) generated title compound **21** as a colourless syrup (22 mg, 44 µmol, 14% (wrt to acceptor **12** used)). R_f = 0.23 (1/1, hexane/EtOAc); ^{1}H NMR (400 MHz, $CDCl_3$) selected signals; δ 8.06 – 8.03 (m, 2H, Ph), 7.61 – 7.56 (m, 1H, Ph), 7.47 – 7.42 (m, 2H, Ph), 7.24 – 7.12 (m, 5H, Ph), 6.90 – 6.85 (m, 2H, Ph), 6.78 – 6.73 (m, 2H, Ph), 5.48 (dd, *J* = 9.1, 8.1 Hz, 1H, H_2), 5.08 (d, *J* = 8.0 Hz, 1H, H_1), 4.78 (d, *J* = 10.5 Hz, 1H, PhCH*H*), 4.70 (d, *J* = 10.5 Hz, 1H, PhC*H*H), 4.10 – 4.04 (m, 1H, H_{6a}), 3.91 – 3.81 (m, 1H, H_{6b}), 3.75 – 3.71 (m, 4H, H_3, OCH_3), 3.59 (ddd, *J* = 10.5, 5.5, 2.6 Hz, 1H, H_5), 3.20 (td, *J* = 10.4, 6.7 Hz, 1H, H_4), 2.05 (t, *J* = 7.1 Hz, 6-OH), 1.85 (d, *J* = 6.7 Hz, 1H, SH); ^{13}C NMR (100 MHz, $CDCl_3$) selected signals δ 165.2 (C=O), 155.6 (C_q), 151.1 (C_q), 137.2 (C_q), 133.3 (CH), 129.8 (CH), 129.6 (C_q), 128.5 (CH), 128.4 (CH), 128.2 (CH), 127.9 (CH), 118.4 (CH), 114.6 (CH), 100.7 (C_1), 83.4 (C_3), 77.7 (C_5), 74.9 ($PhCH_2$), 74.3 (C_2), 62.9 (C_6), 55.6 (OCH_3), 41.1 (C_4); HRMS (ESI$^+$) *m/z* found: $(M+Na)^+$ 520.1479, $C_{27}H_{28}O_7SNa$ requires 520.1481.

Compound **22** was characterised as previously.**[8]**

Synthesis of Gluco-azide α-thiol 36 and Thioacetates 39 and 40

1-*S*-Acetyl-2-azido-3-*O*-benzyl-2-deoxy-4-*O*-levulinoyl-6-*O*-(*tert*-butyldiphenylsilyl)-1-thio-α-D-glucopyranose 40

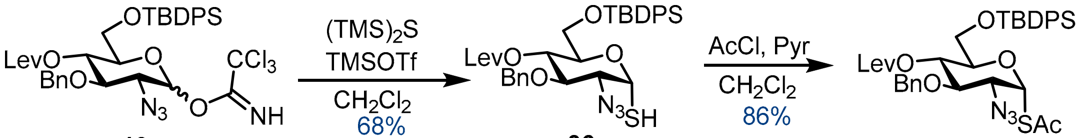

19 **36** **40**

A solution of 2-azido-3-*O*-benzyl-2-deoxy-4-*O*-levulinoyl-6-*O*-(*tert*-butyldiphenylsilyl)-α-D-glucopyranosyl trichloroacetamidate **19** (144 mg, 0.188, 1.00 equiv.) in CH_2Cl_2 (6 mL) was treated with $(TMS)_2S$ (39.0 µL, 0.188 mmol, 1.00 equiv.). TMSOTf (4.80 µL, 26.3 µmol, 0.14 equiv.) was added dropwise at 0 °C, and the reaction was left stirring at this temperature for 1 h. TLC analysis (9/1, CH_2Cl_2/Et_2O) revealed full conversion of the starting material to a higher R_f spot. The reaction was quenched with $NaHCO_3$ (10 mL), and the aqueous phase was separated and extracted with CH_2Cl_2 (2 x 10 mL). The combined organic layers were washed with brine (20 mL), dried over anhydrous $MgSO_4$, filtered and concentrated *in vacuo* to give a yellow syrup. Purification of this crude material *via* column chromatography (1/0 → 9:1, hexane/EtOAc) yielded α-thiol **36** as a colourless syrup (85.0 mg, 0.130 mmol, 68%). R_f = 0.30 (CH_2Cl_2); $[\alpha]^{25}_D$ +124.5 (c = 1.0, $CHCl_3$); ^{1}H NMR (400 MHz, $CDCl_3$) δ 7.70 – 7.61 (m, 4H, Ph), 7.45 – 7.28 (m, 11H, Ph), 5.70 (t, *J* = 5.0 Hz, 1H, H_1), 5.15 (dd, *J* = 10.0, 8.7 Hz, 1H, H_4), 4.79 (d, *J* = 11.1 Hz, 1H, PhC*H*H), 4.66 (d, *J* = 11.1 Hz, 1H, PhCH*H*), 4.17 (ddd, *J* = 10.0, 4.4, 2.5 Hz, 1H, H_5), 3.86 (dd, *J* = 9.9, 5.0 Hz, 1H, H_2), 3.80 (dd, *J* = 9.9, 8.7 Hz, 1H, H_3), 3.71 – 3.67 (m, 2H, H_{6a}, H_{6b}), 2.58 (t, *J* = 6.7 Hz, 2H, Lev-CH_2), 2.33 (t, *J* = 6.7 Hz, 2H, Lev-CH_2), 2.13 (s, 3H, Lev-CH_3), 1.91 (d, *J* = 4.9 Hz, 1H, SH), 1.03 (s, 9H, $SiC(CH_3)_3$); ^{13}C NMR (100 MHz, $CDCl_3$) δ 205.9 (C=O), 171.2 (C=O), 137.5 (C_q), 135.8 (CH), 135.7 (C_q), 133.34 (CH), 133.32 (CH), 129.7 (CH), 129.6 (CH), 128.5 (CH), 128.2 (CH), 128.0 (C_q), 127.62 (CH), 127.61 (CH), 78.8 (C_3), 78.3 (C_1), 75.2 ($PhCH_2$), 72.1 (C_5), 70.4 (C_4), 63.8 (C_2), 62.4 (C_6), 53.4 (Lev-CH_2), 37.7 (Lev-CH_3), 29.8 (Lev-CH_2), 27.8 ($SiC(CH_3)_3$), 19.3 ($Si\mathit{C}(CH_3)_3$); HRMS (ESI$^+$) *m/z* [Found: $(M+NH_4)^+$ 665.2819 $C_{34}H_{45}N_4O_6SSi$, requires 665.2829].
A solution of 2-azido-3-*O*-benzyl-2-deoxy-4-*O*-levulinoyl-6-*O*-(*tert*-butyldiphenylsilyl)-1-thio-α-D-glucopyranoside **36** (101 mg, 0.154 mmol, 1.00 equiv.) in CH_2Cl_2 (0.5 mL) was cooled to 0 °C and treated with pyridine (37.0 µL, 0.462 mmol, 3.00 equiv.) followed by the dropwise addition of AcCl (33.0 µL, 0.462 mmol, 3.00 equiv.). The reaction was stirred for 30 min at RT. TLC analysis (CH_2Cl_2) revealed full conversion of the starting material to a lower R_f spot. The reaction was diluted in CH_2Cl_2 (5 mL) and washed with $NaHCO_3$ (5 mL). The aqueous layer was separated and extracted with CH_2Cl_2 (2 x 5 mL), and the combined organic layers were washed with brine (15 mL), dried over anhydrous $MgSO_4$, filtered and concentrated in *vacuo* to give a yellow syrup. This crude material was dissolved in toluene (5 mL) and concentrated *in* vacuo to facilitate the azeotropic removal of residual pyridine. The process was repeated twice to generate title compound **40** as a colourless syrup (89 mg, 0.13 mmol,

86%). R_f = 0.33 (CH_2Cl_2); $[\alpha]^{25}_D$ +86.4 (c = 0.5, $CHCl_3$); ^1H NMR (400 MHz, $CDCl_3$) δ 7.63 – 7-67 (m, 4H, Ph), 7.45 – 7.27 (m, 11H, Ph), 6.14 (d, *J* = 5.4 Hz, 1H, H_1), 5.16 (dd, *J* = 9.8, 9.3 Hz, 1H, H_4), 4.79 (d, *J* = 11.1 Hz, 1H, PhC*H*H), 4.65 (d, *J* = 11.1 Hz, 1H, PhCH*H*), 3.97 (dd, *J* = 10.1, 5.4 Hz, 1H, H_2), 3.74 – 3.65 (m, 3H, H_5, H_{6a}, H_{6b}), 3.52 – 3.47 (m, 1H, H_3), 2.58 (t, *J* = 7.0 Hz, 2H, Lev-CH_2), 2.43 (s, 3H, Lev-CH_3), 2.34 – 2.30 (m, 2H, Lev-CH_2), 2.12 (s, 3H, CH_3), 1.05 – 0.99 (m, 9H, $SiC(CH_3)_3$); ^{13}C NMR (100 MHz, $CDCl_3$) δ 205.9 (C=O), 191.4 (C=O), 171.2 (C=O), 137.4 (C_q), 135.7 (CH), 133.3 (C_q), 133.2 (CH), 129.64 (CH), 129.60 (CH), 128.5 (CH), 128.1 (CH), 128.0 (C_q), 127.6 (CH), 81.7 (C_1), 80.5 (C_3), 75.1 (C_5), 75.0 ($PhCH_2$), 70.0 (C_4), 63.2 (C_2), 62.3 (C_6), 37.7 (Lev-CH_2), 31.5 (Lev-CH_3), 29.8 (CH_3), 27.8 (Lev-CH_2), 26.7 ($SiC(CH_3)_3$), 19.3 ($SiC(CH_3)_3$); HRMS (ESI$^+$) *m/z* found: $(M+Na)^+$ 712.2488, $C_{36}H_{43}N_3O_7SSNa$ requires $(M+Na)^+$ 712.2492.

2-Acetamido-1-*S*-acetyl-3-*O*-benzoyl-2-deoxy-4-*O*-levulinoyl-6-*O*-(*tert*-butyldiphenylsilyl)-1-thio-α-D-glucopyranose 39

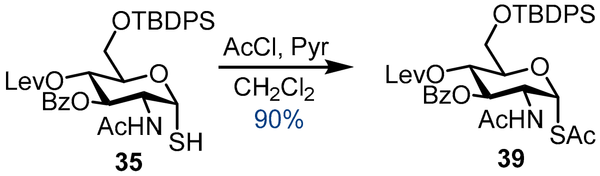

A solution of 2-acetamido-3-*O*-benzoyl-2-deoxy-4-*O*-levulinoyl-6-*O*-(*tert*-butyldiphenylsilyl)-1-thio-α-D-glucopyranose **35** (1.03 g, 1.48 mmol, 1.00 equiv.) in CH_2Cl_2 (9 mL) was cooled to 0 °C and treated with pyridine (358 µL, 4.44 mmol, 3.00 equiv.) along with the dropwise addition of AcCl (317 µL, 4.44 mmol, 3.00 equiv.). The reaction was stirred for 30 min at RT. TLC analysis (CH_2Cl_2) revealed full conversion of the starting material to a lower R_f spot. The reaction was diluted in CH_2Cl_2 (5 mL) and washed with $NaHCO_3$ (5 mL). The aqueous layer was separated and extracted with CH_2Cl_2 (2 x 5 mL), and the combined organic layers were washed with brine (15 mL), dried over anhydrous $MgSO_4$, filtered and concentrated in *vacuo* to give a yellow syrup. This crude material was dissolved in toluene (10 mL) and concentrated *in* vacuo to facilitate the azeotropic removal of residual pyridine. The process was repeated twice to furnish title compound **39** as a white solid (947 mg, 1.32 mmol, 90%). R_f = 0.38 (9/1, CH_2Cl_2/Et_2O); m.p. 160 – 163 °C; $[\alpha]^{23}_D$ +45.4 (c = 0.50, $CHCl_3$); ^1H NMR (400 MHz, $CDCl_3$) δ 8.01 – 7.95 (m, 2H, Ph), 7.72 – 7.64 (m, 4H, Ph), 7.62 – 7.55 (m, 1H, Ph), 7.48 – 7.34 (m, 8H, Ph), 6.26 (d, *J* = 5.1 Hz, 1H, H_1), 5.77 (d, *J* = 8.5 Hz, 1H, NH), 5.47 (t, *J* = 9.7 Hz, 1H, H_4), 5.13 (dd, *J* = 11.3, 9.4 Hz, 1H, H_3), 4.74 (ddd, *J* = 11.3, 8.5, 5.1 Hz, 1H, H_2), 3.86 (dt, *J* = 10.1, 3.1 Hz, 1H, H_5), 3.76 (d, *J* = 3.2 Hz, 2H, $H_{6a,}$ H_{6b}), 2.51 (t, *J* = 6.7 Hz, 2H, Lev-CH_2), 2.44 (s, 3H, CH_3), 2.31 (t, *J* = 6.5 Hz, 2H, Lev-CH_2), 1.94 (s, 3H, Lev-CH_3), 1.84 (s, 3H, CH_3), 1.05 (s, 9H, $SiC(CH_3)_3$); ^{13}C NMR (100 MHz, $CDCl_3$) δ 205.5 (C=O), 190.9 (C=O), 171.1 (C=O), 169.8 (C=O), 167.5 (C=O), 135.78 (CH), 135.77 (CH), 133.70 (CH), 133.2 (C_q), 133.1 (C_q), 130.1 (CH), 129.69 (CH), 129.66 (CH), 128.70 (CH), 128.6 (C_q), 127.67 (CH), 127.64 (CH), 82.6 (C_1), 74.8 (C_5), 73.2 (C_3), 67.8 (C_4), 62.3 (C_6), 52.4 (C_2), 37.9 (Lev-CH_2), 31.6 (CH_3), 29.4 (Lev-CH_3), 27.8 (Lev-CH_2), 26.7 ($SiC(CH_3)_3$), 23.1 (CH_3), 19.3 ($SiC(CH_3)_3$); HRMS (ESI$^+$) *m/z* found: $(M+H)^+$ 720.2650, $C_{38}H_{46}NO_9SSi$ requires 720.2668.

Side products formed using NaH mediated S_N2 Coupling

From Thiohemiacetal 33

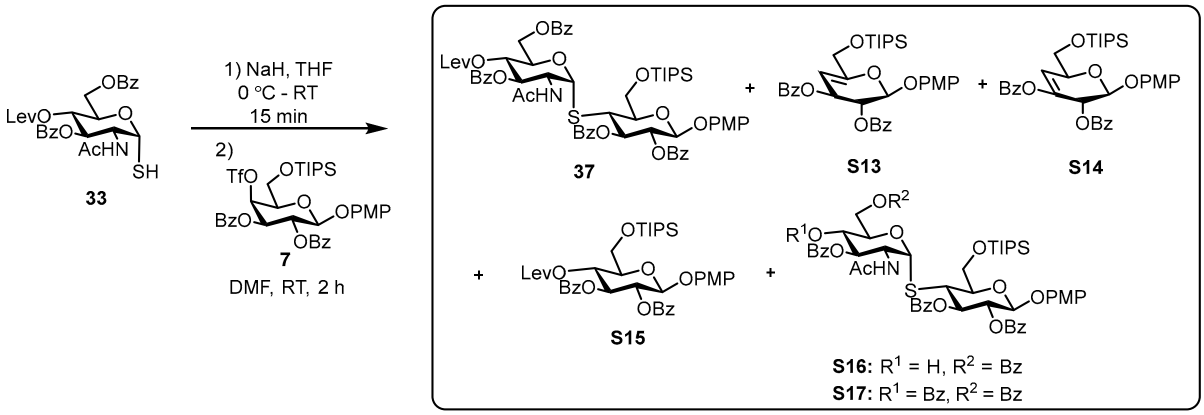

***p*-Methoxyphenyl 2,3-di-*O*-benzyl-4-deoxy-6-*O*-(triisopropylsilyl)-α-L-*threo*-hex-4-enopyranosid S13** and ***p*-methoxyphenyl 2,3-di-*O*-benzyl-4-deoxy-6-*O*-(triisopropylsilyl)-β-D-*erythro*-hex-4-enopyranosid S14**

Alkene side products **S13** and **S14** were obtained as an inseparable mixture appearing as a colourless syrup (1.0/0.4, **S13**/**S14**); the ratio was determined from ^{1}H NMR integration values. R_f = 0.50 (8/2, hexane/EtOAc).

S13: ^{1}H NMR (400 MHz, $CDCl_3$) selected signals δ 5.80 (dd, *J* = 3.4, 1.0 Hz, 1H, H_4), 5.70 (td, *J* = 3.1, 1.2 Hz, 1H, H_3), 5.70 – 5.63 (m, 1H, H_2), 5.45 (dd, *J* = 4.5, 1.2 Hz, 1H, H_1), 4.25 – 4.14 (m, 2H, H_{6a}, H_{6b}), 3.77 (s, 3H, OCH_3), 1.08 – 1.04 (m, 21H, $Si(C_3H_7)_3$); ^{13}C NMR (100 MHz, $CDCl_3$) selected signals δ 165.88 (C=O), 165.2 (C=O), 153.9 (C_5), 118.4 (CH), 114.60 (CH), 95.6 (C_4), 93.9 (C_1), 68.9 (C_3), 65.5 (C_2), 62.2 (C_6), 55.70 (OCH_3), 11.94 ($Si(CH(CH_3)_2)_3$).

S14: ^{1}H NMR (400 MHz, $CDCl_3$) selected signals δ 6.30 (d, *J* = 2.5 Hz, 1H, H_4), 5.90 (dd, *J* = 3.5, 1.6 Hz, 1H, H_2), 5.62 (d, *J* = 3.5 Hz, 1H, H_1), 4.73 (ddt, *J* = 8.0, 6.0, 1.9 Hz, 1H, H_5), 3.86 (dd, *J* = 9.5, 5.9 Hz, 1H, H_{6a}), 3.83 – 3.74 (m, 4H, H_{6b}, OCH_3), 1.04 – 1.00 (m, 21H, $Si(C_3H_7)_3$); ^{13}C NMR selected signals (100 MHz, $CDCl_3$) δ 165.84 (C=O), 164.3 (C=O), 141.1 (C_3), 118.7 (C_4) 117.9 (CH), 114.58 (CH), 98.3 (C_1), 73.5 (C_5), 67.8 (C_2), 66.2 (C_6), 55.71 (OCH_3), 11.86 ($Si(CH(CH_3)_2)_3$); HRMS (ESI$^+$) *m/z* found $(M+NH_4)^+$ 651.3172, $C_{36}H_{48}N_2O_8S$ requires 651.3173.

***p*-Methoxyphenyl 2,3-di-*O*-benzoyl-4-*O*-levulinoyl-6-*O*-(triisopropylsilyl)-β-D-glucopyranoside S15**

O-4 Levulinate glucoside **S15** was obtained as a white solid (115 mg, 0.154 mmol, 6%). R_f = 0.74 (2/1, hexane/EtOAc); $[\alpha]^{23}_D$ +84.7 (c = 0.85, $CHCl_3$); m.p. 80 – 82 °C; ^{1}H NMR (400 MHz, $CDCl_3$) δ 8.00 – 7.89 (m, 4H, Ph), 7.56 – 7.47 (m, 2H, Ph), 7.42 – 7.33 (m, 4H, Ph), 7.00 – 6.94 (m, 2H, Ph), 6.77 – 6.72 (m, 2H, Ph), 5.72 (t, *J* = 9.5 Hz, 1H, H_3), 5.63 (dd, *J* = 9.8, 7.8 Hz, 1H, H_2), 5.32 (t, *J* = 9.4 Hz, 1H, H_4), 5.14 (d, *J* = 7.8 Hz, 1H, H_1), 3.95 – 3.81 (m, 3H, H_5, H_{6a}, H_{6b}), 3.74 (s, 3H, OCH_3), 2.66 – 2.45 (m, 3H, LevC*H*H, Lev-CH_2), 2.42 – 2.33 (m, 1H, Lev-CH*H*), 2.03 (s, 3H, Lev-CH_3), 1.14 – 1.05 (m, 21H, $Si(C_3H_7)_3$); ^{13}C NMR (100 MHz, $CDCl_3$) δ 205.6 (C=O), 171.4 (C=O), 165.9 (C=O), 165.1 (C=O), 155.6 (C_q), 151.4 (C_q), 133.3 (CH), 133.2 (CH), 129.9 (CH), 129.8 (CH), 129.3 (C_q), 129.0 (C_q), 128.40 (CH), 128.38 (CH), 119.0 (CH), 114.4 (CH), 100.9 (C_1), 75.7 (C_5), 73.4 (C_3), 71.9 (C_2), 69.0 (C_4), 62.5 (C_6), 55.6 (OCH_3), 37.8 (Lev-CH_2), 29.5 (Lev-CH_3), 27.9 (Lev-CH_2), 17.9 ($Si(CH(CH_3)_2)_3$), 11.9 ($Si(CH(CH_3)_2)_3$); HRMS (ESI$^+$) *m/z* found $(M+NH_4)^+$ 766.3616, $C_{41}H_{56}NO_{11}S$ requires 766.3617.

S-(2-Acetamido-3,4,6-tri-O-benzoyl-2-deoxy-α-D-glucopyranosyl)-(1→4)-p-methoxyphenyl 2,3-di-O-benzoyl-4-thio-β-D-glucopyranoside S17

O-3 benzoate *S*-linked disaccharide **S17** was obtained as a colourless syrup (113 mg, 95.6 μmmol, 6%). R_f = 0.42 (2/1, hexane/EtOAc); $[\alpha]^{23}_D$ +133.5 (c = 1.40, $CHCl_3$); ^{1}H NMR (400 MHz, $CDCl_3$) δ 8.12 – 8.08 (m, 1H, Ph), 8.08 – 8.05 (m, 2H, Ph), 7.98 – 7.90 (m, 4H, Ph), 7.87 – 7.80 (m, 4H, Ph), 7.65 – 7.28 (m, 14H, Ph), 6.98 – 6.92 (m, 2H, Ph), 6.77 – 6.72 (m, 2H, Ph), 5.77 (dd, *J* = 10.8, 9.6 Hz, 1H, H_3), 5.71 (d, *J* = 5.1 Hz, 1H, $H_{1'}$), 5.70 – 5.65 (m, 1H, $H_{4'}$), 5.56 (d, *J* = 9.2 Hz, 1H, NH), 5.52 (dd, *J* = 9.5, 7.9 Hz, 1H, H_2), 5.39 (dd, *J* = 11.2, 9.4 Hz, 1H, $H_{3'}$), 5.15 (d, *J* = 7.9 Hz, 1H, H_1), 4.69 (ddd, *J* = 11.3, 9.3, 5.2 Hz, 1H, $H_{2'}$), 4.61 – 4.51 (m, 2H, $H_{5'}$, $H_{6a'}$), 4.49 – 4.40 (m, 1H, $H_{6b'}$), 4.29 (dd, *J* = 10.9, 1.6 Hz, 1H, H_{6a}), 4.16 (dd, *J* = 11.0, 5.1 Hz, 1H, H_{6b}), 3.81 – 3.73 (m, 4H, H_5, OCH_3), 3.47 (t, *J* = 10.8 Hz, 1H, H_4), 1.36 (s, 3H, CH_3), 1.12 – 1.03 (m, 21H, $Si(C_3H_7)_3$); ^{13}C NMR (100 MHz, $CDCl_3$) δ 169.4 (C=O), 166.9 (C=O), 166.1 (C=O), 165.9 (C=O), 165.3 (C=O), 164.8 (C=O), 155.6 (C_q), 151.4 (C_q), 133.6 (CH), 133.54 (CH), 133.46 (CH), 133.2 (CH), 130.0 (CH), 129.92 (CH), 129.85 (CH), 129.8 (CH), 129.8 (CH), 129.5 (C_q), 129.3 (C_q), 128.81 (C_q), 128.79 (C_q), 128.7 (CH), 128.51 (CH), 128.48 (CH), 128.43 (CH), 128.41 (CH), 128.38 (CH), 119.1 (CH), 114.4 (CH), 100.7 (C_1), 85.6 ($C_{1'}$), 77.4 (C_5), 74.7 (C_3), 73.0 (C_2), 71.8 ($C_{3'}$), 70.0 ($C_{5'}$), 68.7 ($C_{4'}$), 63.5 (C_6), 62.6 ($C_{6'}$), 55.6 (OCH_3), 52.3 ($C_{2'}$), 46.5 (C_4), 22.5 (CH_3), 20.8 ($Si(CH(CH_3)_2)_3$), 17.9 ($Si(CH(CH_3)_2)_3$), 12.0 ($Si(CH(CH_3)_2)_3$); HRMS (ESI^+) *m/z* found: $(M+Na)^+$ 1204.4141, $C_{65}H_{71}NO_{16}SSiNa$ requires 1204.4160.

S-(2-Acetamido-3,6-di-O-benzoyl-2-deoxy-α-D-glucopyranosyl)-(1→4)-p-methoxyphenyl 2,3-di-O-benzoyl-4-thio-6-O-(triisopropylsilyl)-β-D-glucopyranoside S16

C4-OH *S*-linked disaccharide **S16** was obtained as a colourless syrup (107 mg, 99.3 μmol, 6%). R_f = 0.37 (2/1, hexane/EtOAc); $[\alpha]^{25}_D$ +86.4 (c = 0.5, CH_3Cl); ^{1}H NMR (400 MHz, $CDCl_3$) δ 8.13 – 8.07 (m, 2H, Ph), 7.96 – 7.88 (m, 6H, Ph), 7.66 – 7.57 (m, 1H, Ph), 7.57 – 7.46 (m, 5H, Ph), 7.42 – 7.32 (m, 6H, Ph), 6.97 – 6.92 (m, 2H, Ph), 6.78 – 6.71 (m, 2H, Ph), 5.76 (dd, *J* = 10.8, 9.6 Hz, 1H, H_3), 5.60 (d, *J* = 5.3 Hz, 1H, $H_{1'}$), 5.50 (dd, *J* = 9.5, 7.9 Hz, 1H, H_2), 5.45 (d, *J* = 9.7 Hz, 1H, NH), 5.14 (d, *J* = 7.9 Hz, 1H, H_1), 5.05 (dd, *J* = 11.2, 9.1 Hz, 1H, $H_{3'}$), 4.90 (dd, *J* = 12.3, 3.0 Hz, 1H, $H_{6a'}$), 4.56 (ddd, *J* = 11.1, 9.8, 5.3 Hz, 1H, $H_{2'}$), 4.46 (dd, *J* = 12.3, 2.1 Hz, 1H, $H_{6b'}$), 4.28 (dd, *J* = 10.9, 1.8 Hz, 1H, H_{6a}), 4.21 (dt, *J* = 9.7, 2.4 Hz, 1H, $H_{5'}$), 4.13 (dd, *J* = 10.6, 6.2 Hz, 1H, H_{6b}), 3.81 – 3.73 (m, 5H, $H_{4'}$, H_5, CH_3), 3.43 (t, *J* = 10.8 Hz, 1H, H_4), 3.17 (d, *J* = 4.6 Hz, 1H, 4’-OH), 1.33 (s, 3H, CH_3), 1.08 (m, 21H, $SiCH(CH_3)_3$); ^{13}C NMR (100 MHz, $CDCl_3$) δ 169.5 (C=O), 167.6 (C=O), 167.2 (C=O), 165.8 (C=O), 165.3 (C=O), 155.6 (C_q), 151.4 (C_q), 133.64 (CH), 133.59 (CH), 133.5 (CH), 133.2 (CH), 130.0 (CH), 129.9 (CH), 129.7 (CH), 129.34 (C_q), 129.27 (C_q), 128.9 (C_q), 128.73 (C_q), 128.67 (CH), 128.60 (CH), 128.5 (CH), 128.4 (CH), 119.0 (CH), 114.4 (CH), 100.7 (C_1), 86.1 ($C_{1'}$), 77.0 ($C_{4'}/C_5$), 74.9 (C_3), 74.3 ($C_{3'}$), 73.1 (C_2), 72.5 ($C_{5'}$), 68.7 ($C_{4'}/C_5$), 63.5 (C_6), 63.0 ($C_{6'}$), 55.6 (OCH_3), 51.5 ($C_{2'}$), 46.6 (C_4), 22.5 (CH_3), 18.0 ($SiCH(CH_3)_2$), 12.00 ($SiCH(CH_3)_2$).

From Thiohemiacetal 36

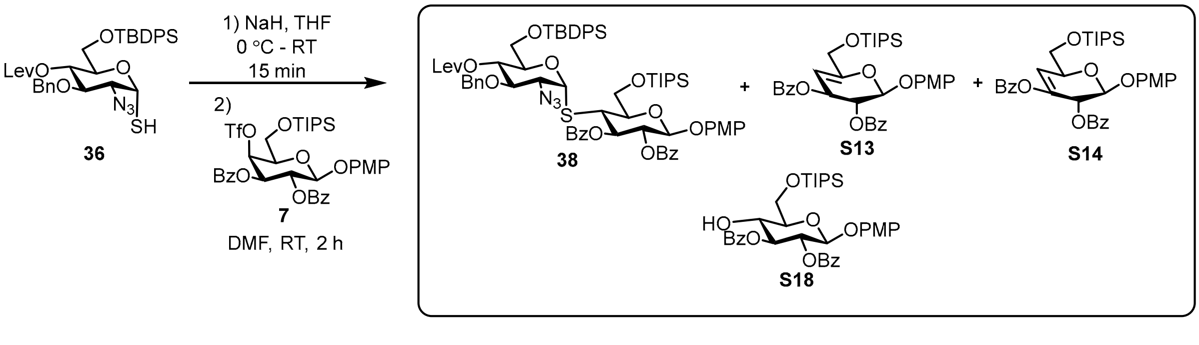

_p_-Methoxyphenyl 2,3-di-_O_-benzoyl-6-_O_-(triisopropylsilyl)-β-D-glucopyranoside S18

Glucoside **S18** was obtained as a white foam (54 mg, 83 μmmol, 35%). R_f = 0.73 (8/2, CH_2Cl_2/Et_2O); $[\alpha]^{23}_D$ −87.8 (c = 0.50, $CHCl_3$); ^1H NMR (400 MHz, $CDCl_3$) δ 8.02 – 7.91 (m, 4H, Ph), 7.55 – 7.47 (m, 2H, Ph), 7.43 – 7.34 (m, 4H, Ph), 6.97 – 6.92 (m, 2H, Ph), 6.78 – 6.70 (m, 2H, Ph), 5.77 (t, J = 10.0 Hz, 1H, H_3), 5.55 (dd, J = 9.7, 8.0 Hz, 1H, H_2), 5.14 (d, J = 8.0 Hz, 1H, H_1), 4.23 (t, J = 10.2 Hz, 1H, H_4), 4.17 (dd, J = 11.1, 1.6 Hz, 1H, H_{6a}), 4.08 (dd, J = 11.1, 4.7 Hz, 1H, H_{6b}), 3.78 (ddd, J = 10.1, 4.6, 1.5 Hz, 1H, H_5), 3.74 (s, 3H, OCH_3), 1.15 – 1.06 (m, 21H, $Si(C_3H_7)_3$); ^{13}C NMR (100 MHz, $CDCl_3$) δ 165.6 (C=O), 165.2 (C=O), 155.8 (C_q), 151.2 (C_q), 133.4 (CH), 133.3 (CH), 129.9 (C_q), 129.8 (C_q), 129.2 (CH), 129.1 (CH), 128.4 (CH), 119.4 (CH), 114.4 (CH), 101.1 (C_1), 77.6 (C_5), 75.0 (C_3), 72.5 (C_2), 62.5 (C_6), 55.6 (OCH_3), 54.9 (C_4), 17.97 ($Si(CH(\mathit{C}H_3)_2)_3$), 17.95 ($Si(CH(\mathit{C}H_3)_2)_3$), 12.0 ($Si(\mathit{C}H(CH_3)_2)_3$); HRMS ($ESI^+$) m/z found: $(M+Na)^+[-H_2O]$ 655.2720, $C_{36}H_{44}O_8SiNa$ requires 655.2708.

NMR Spectra

Synthesis of C4-Glucuronic and Glucose Acceptors

1,2,3,4,6-Penta-*O*-acetyl-β-D-galactopyranose S1

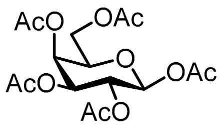

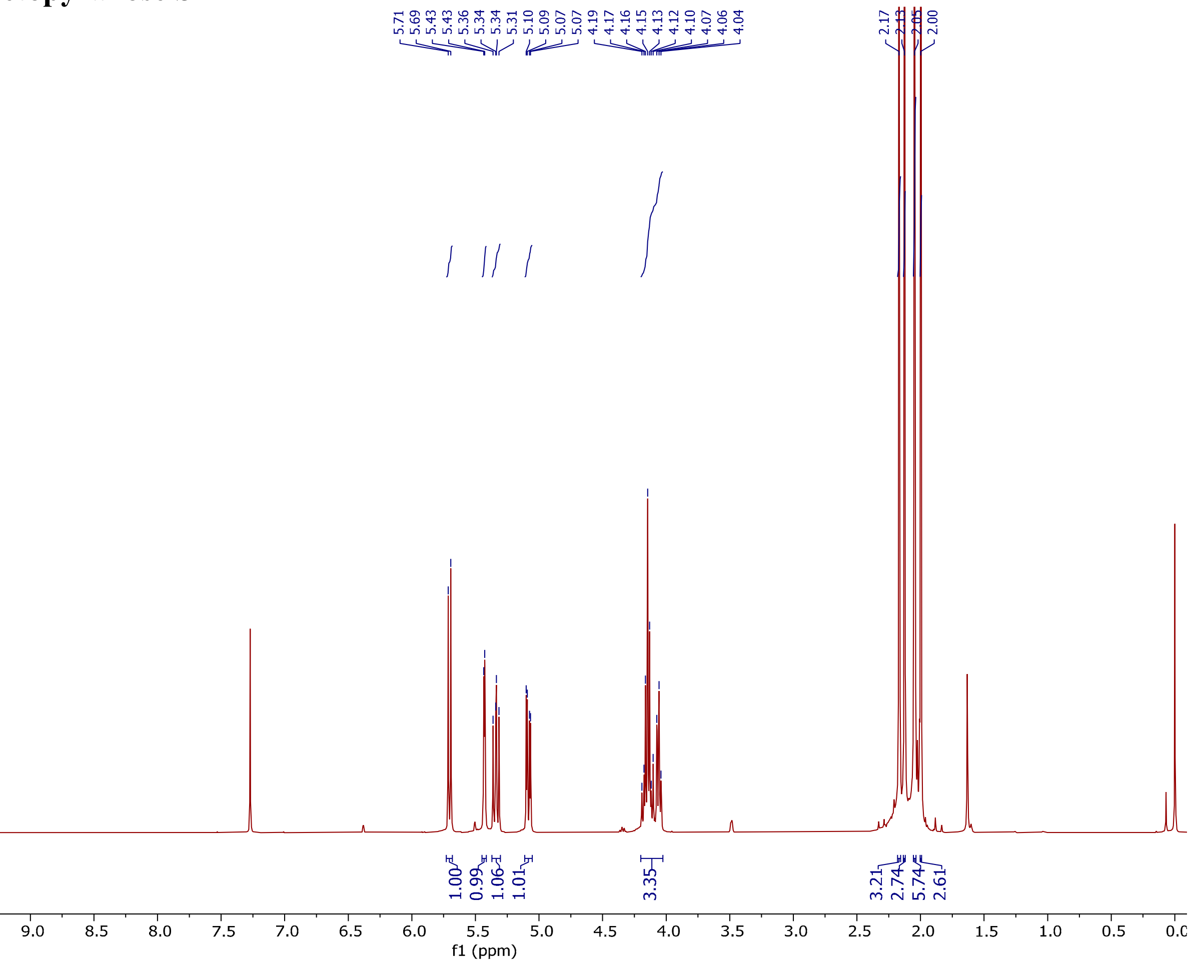

^{1}H NMR (400 MHz, Chloroform-*d*)

1,2,3,4,6-Penta-*O*-acetyl-β-D-galactopyranose S1

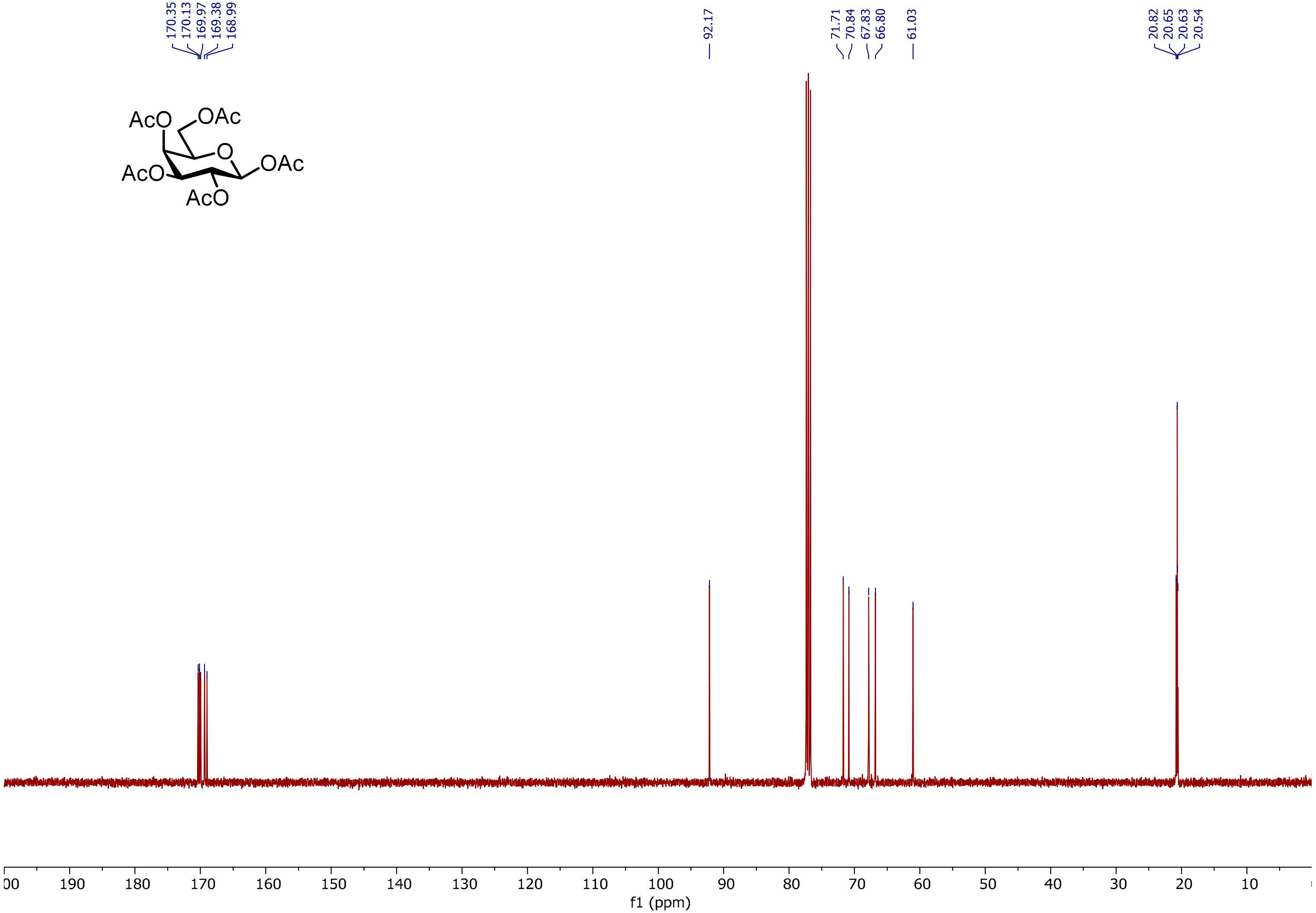

^{13}C NMR (100 MHz, Chloroform-*d*)

_p_-Methoxyphenyl 2,3,4,6-tetra-_O_-acetyl-β-D-galactopyranoside S2

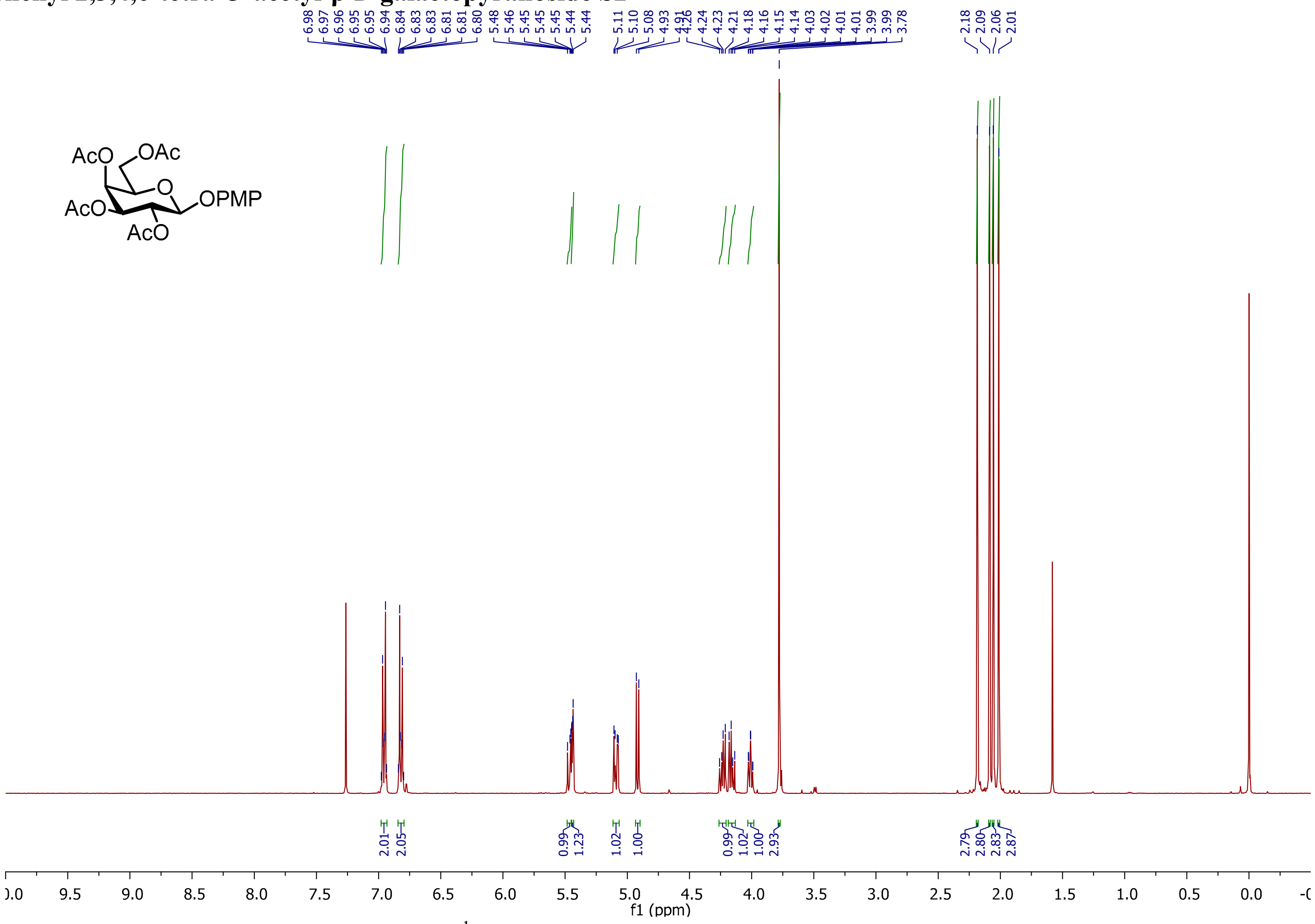

^{1}H NMR (400 MHz, Chloroform-_d_)

p-Methoxyphenyl 2,3,4,6-tetra-O-acetyl-β-D-galactopyranoside S2

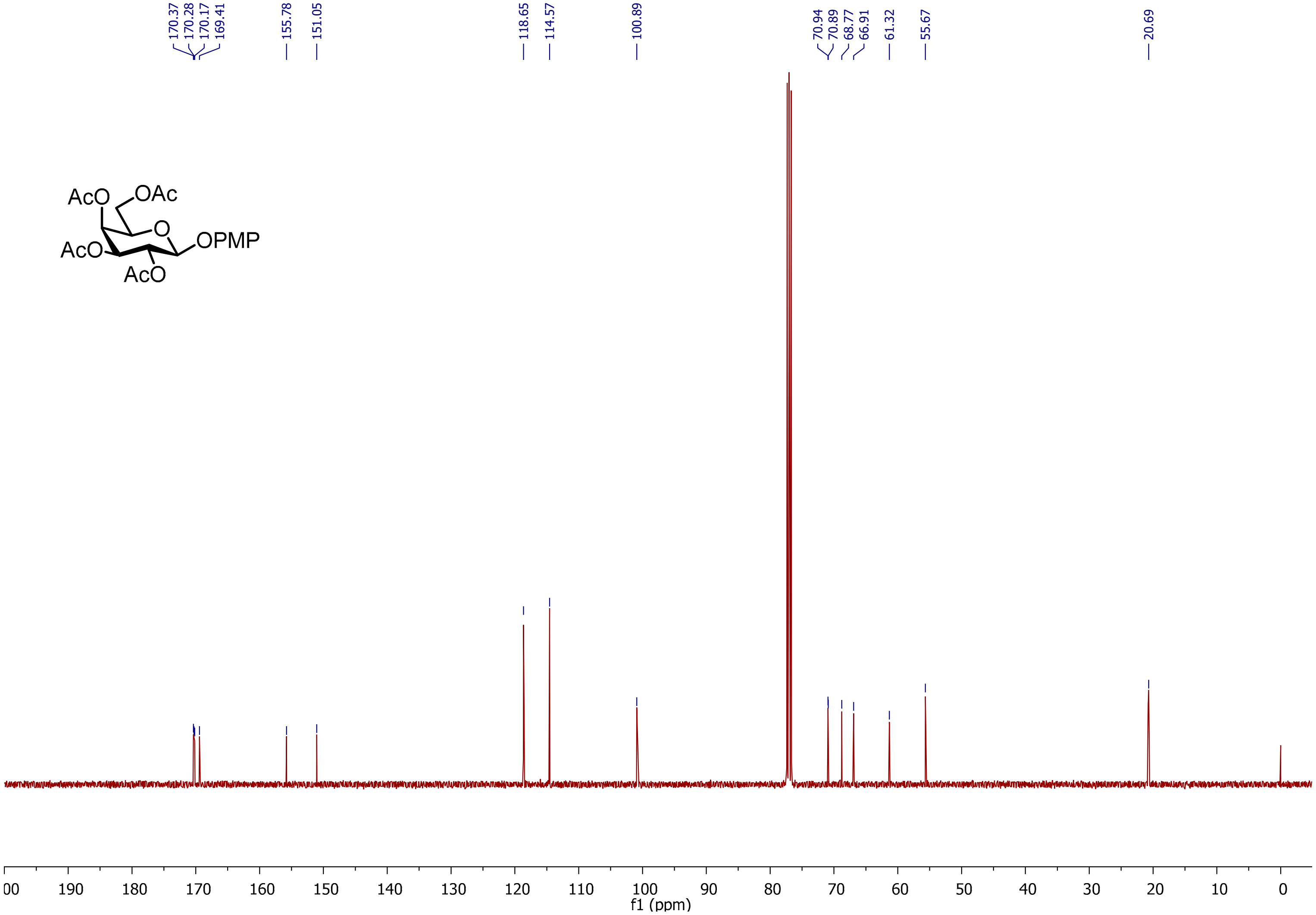

^{13}C NMR (100 MHz, Chloroform-*d*)

_p_-Methoxyphenyl 4,6-_O_-benzylidene-β-D-galactopyranoside S3

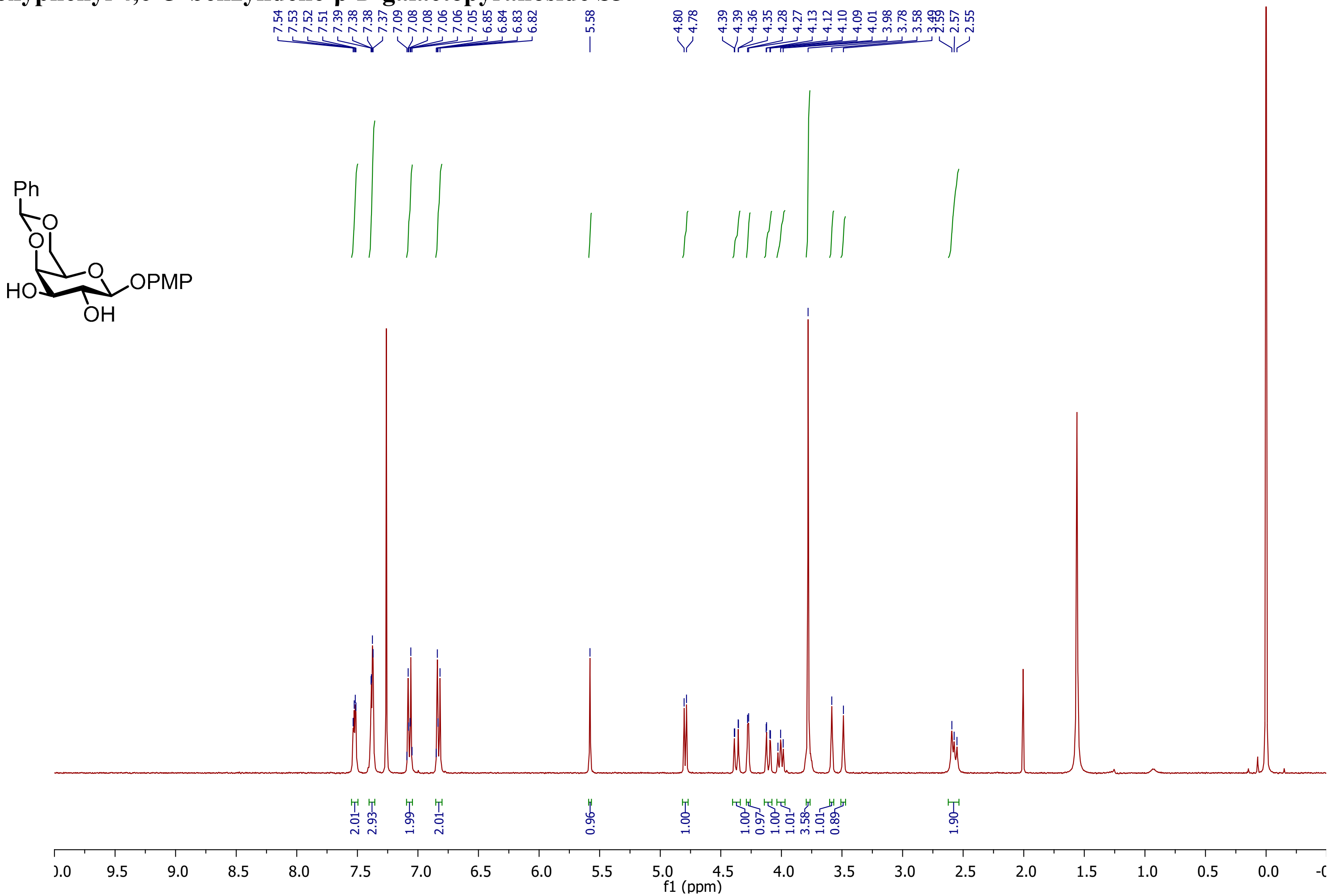

^{1}H NMR (400 MHz, Chloroform-*d*)

_p_-Methoxyphenyl 4,6-_O_-benzylidene-β-D-galactopyranoside S3

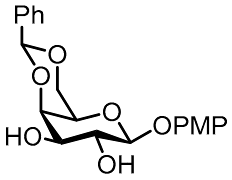

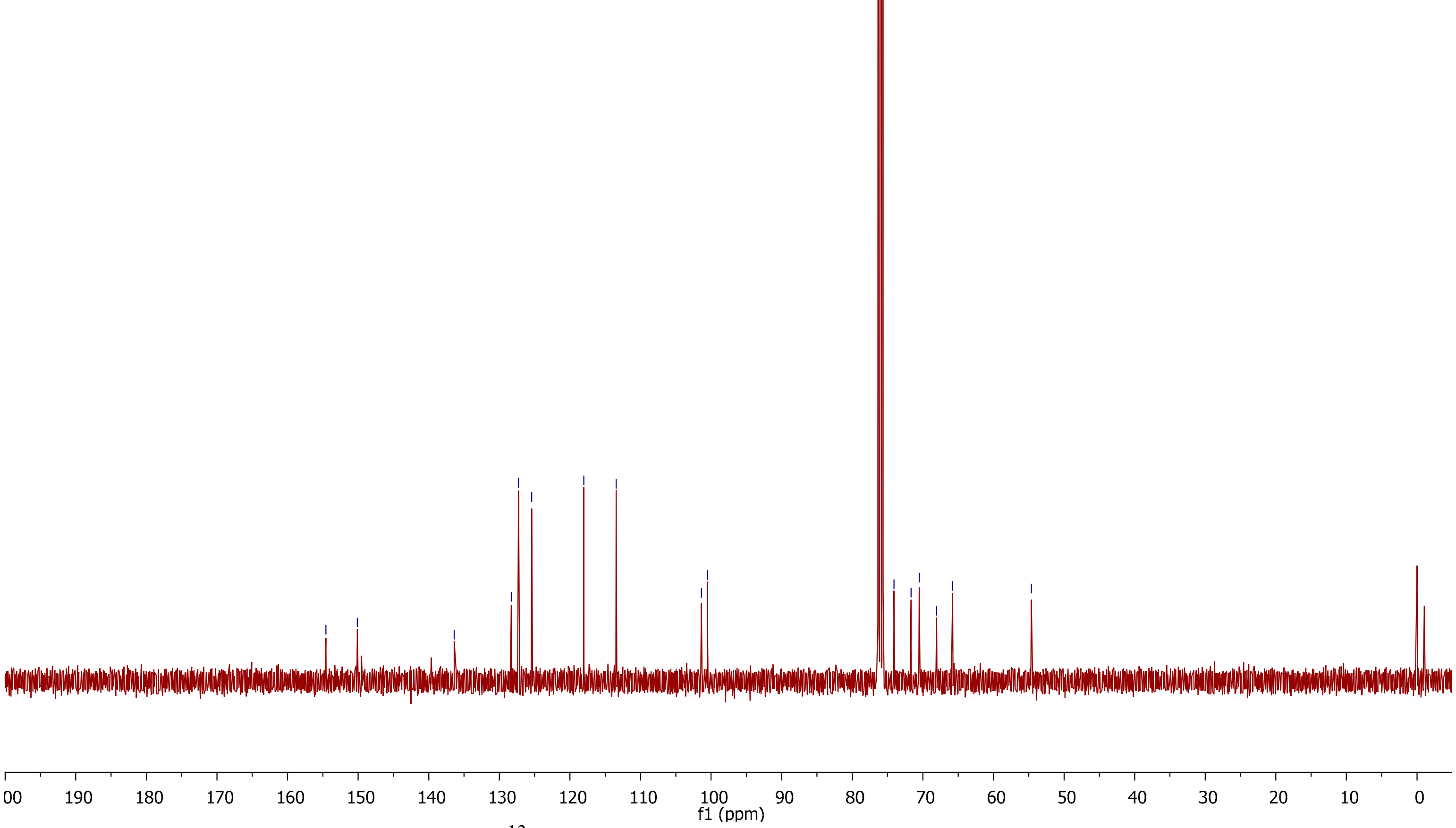

^{13}C NMR (100 MHz, Chloroform-_d_)

***p*-Methoxyphenyl 2,3-di-*O*-benzoyl-4,6-*O*-benzylidene-β-D-galactopyranoside S4**

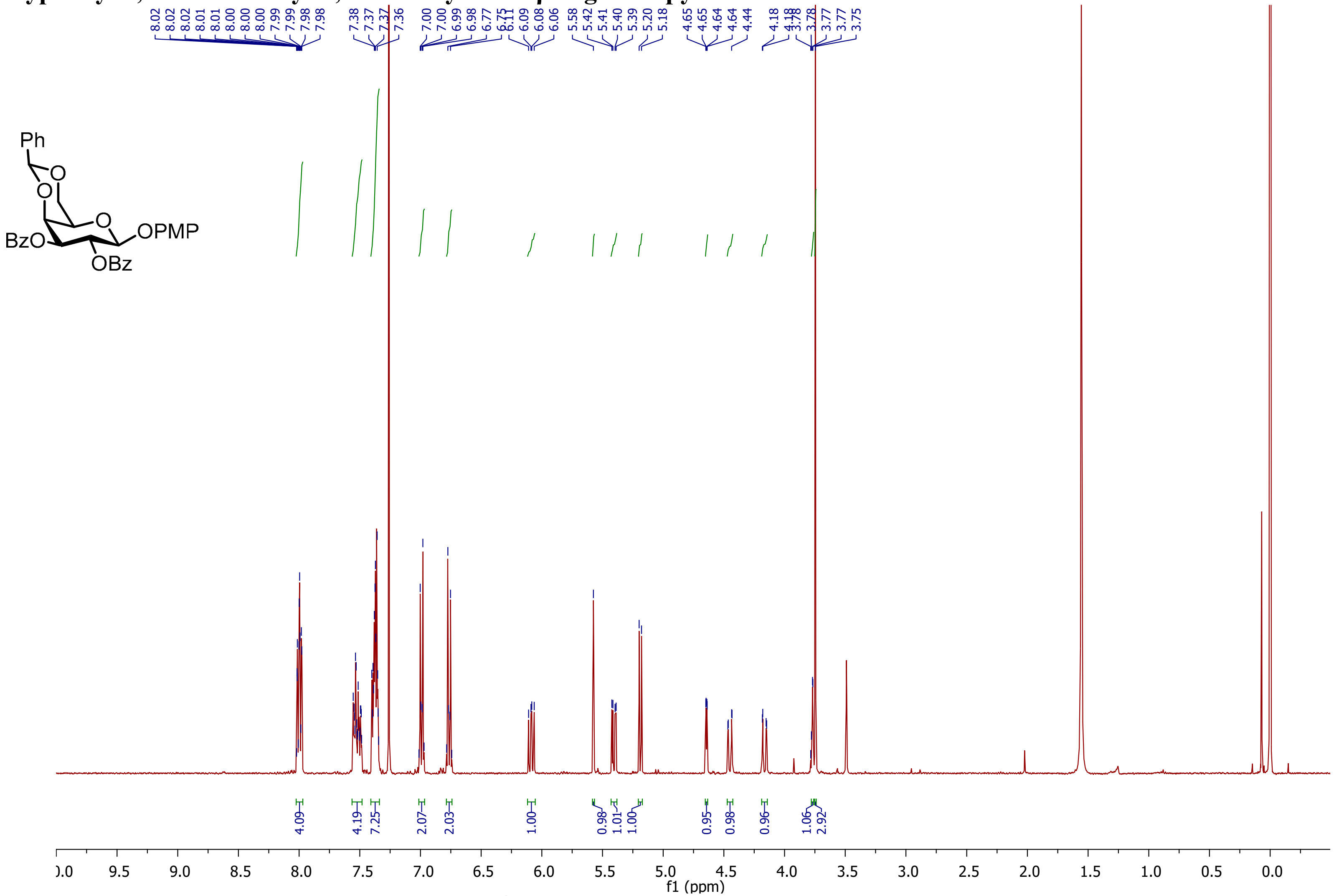

^{1}H NMR (400 MHz, Chloroform-*d*)

p-Methoxyphenyl 2,3-di-O-benzoyl-4,6-O-benzylidene-β-D-galactopyranoside S4

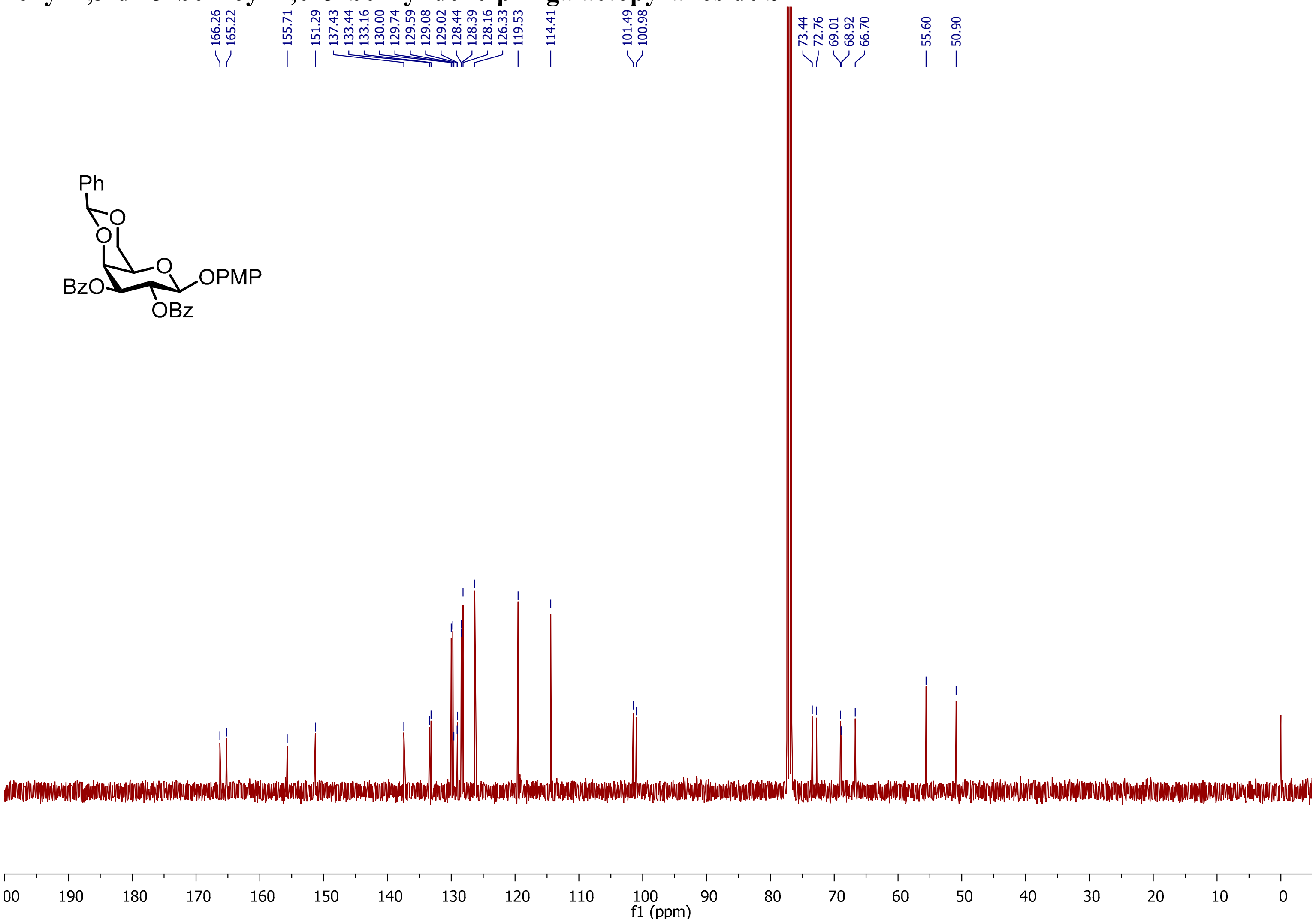

^{13}C NMR (100 MHz, Chloroform-*d*)

_p_-Methoxyphenyl 2,3-di-_O_-benzoyl-β-D-galactopyranoside 1

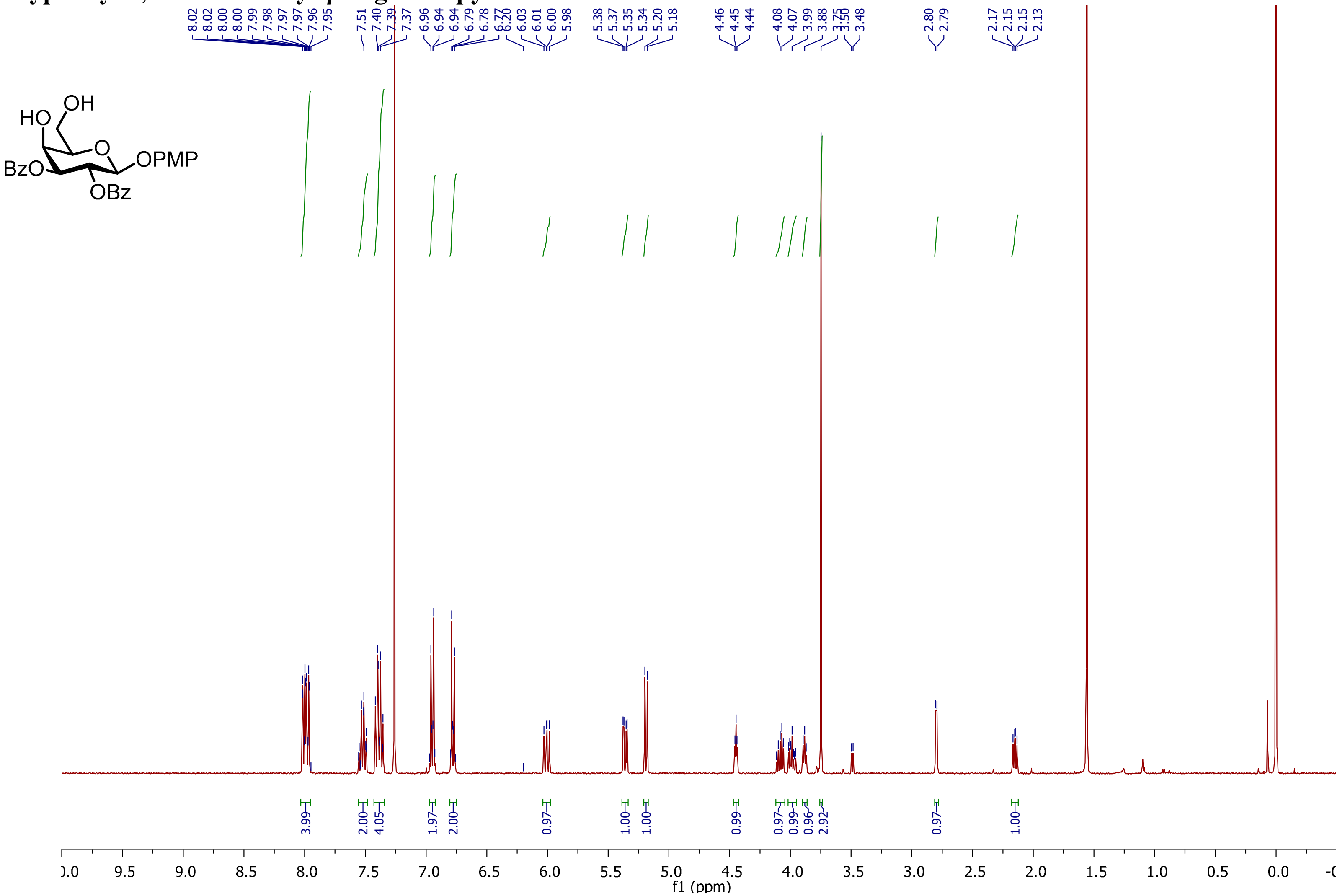

^{1}H NMR (400 MHz, Chloroform-*d*)

p-Methoxyphenyl 2,3-di-O-benzoyl-β-D-galactopyranoside 1

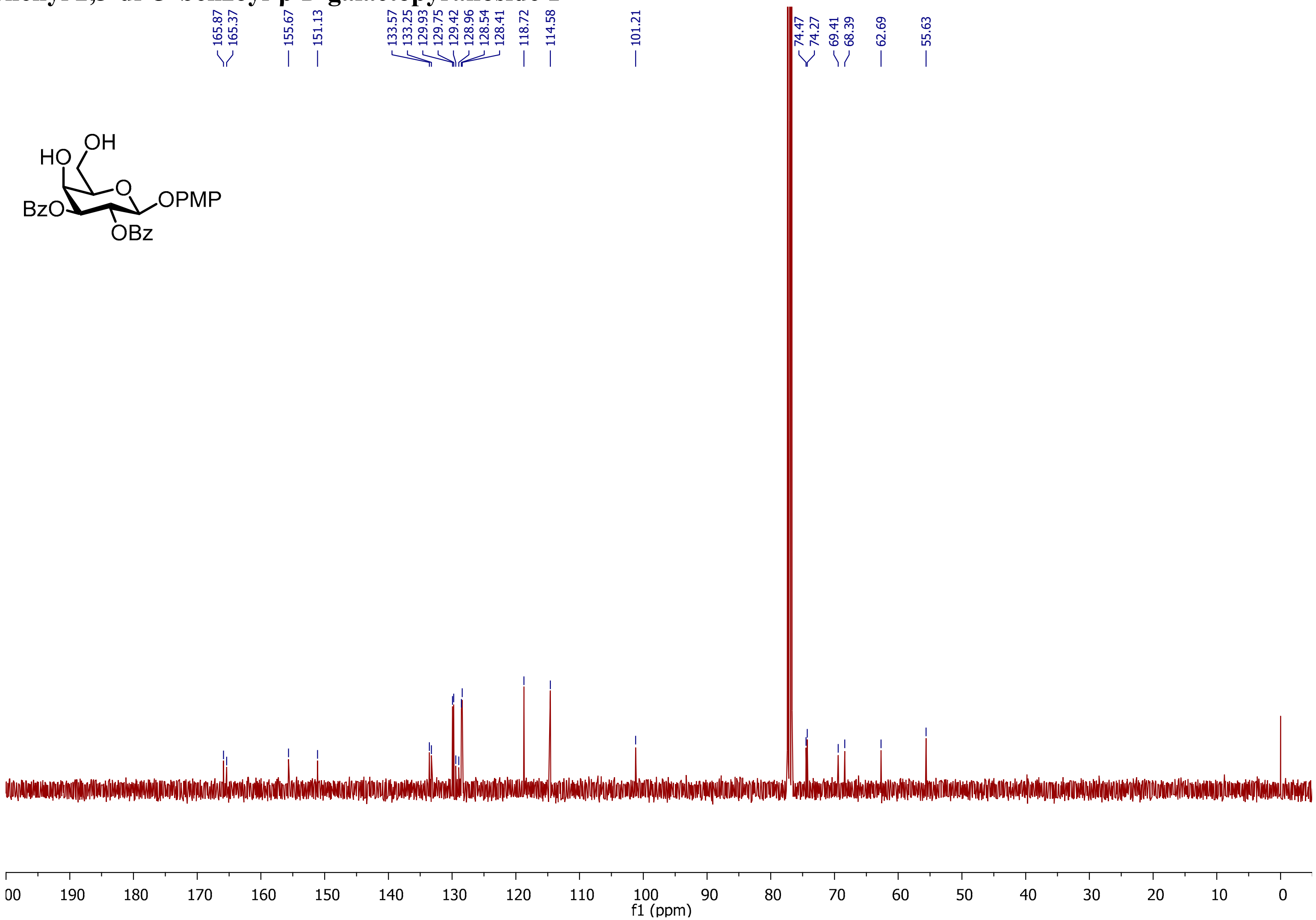

^{13}C NMR (100 MHz, Chloroform-*d*)

Methyl (*p*-methoxyphenyl 2,3-di-*O*-benzoyl-β-D-galactopyranosid)uronate 3

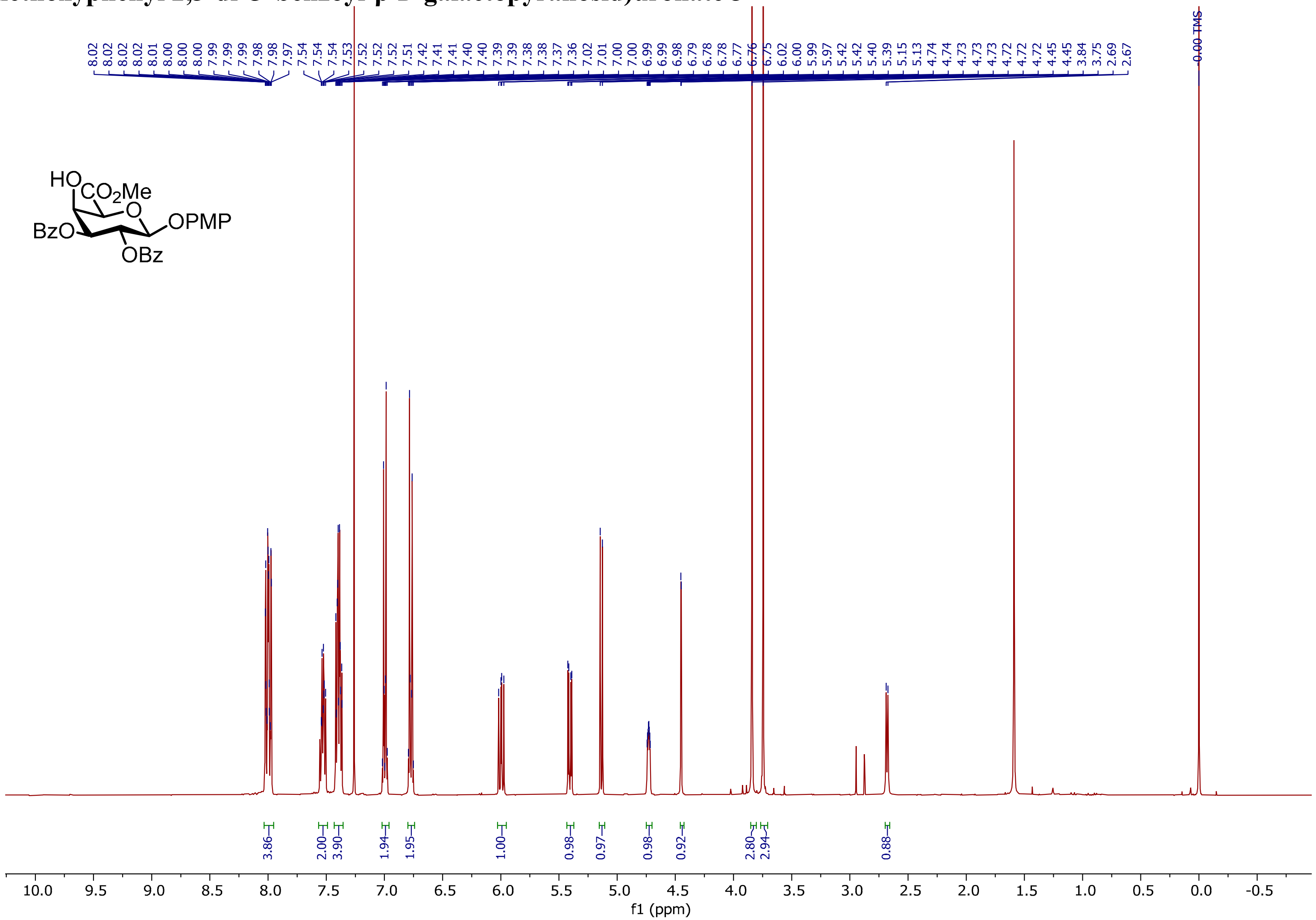

^1H NMR (400 MHz, Chloroform-*d*)

Methyl (*p*-methoxyphenyl 2,3-di-*O*-benzoyl-β-D-galactopyranosid)uronate 3

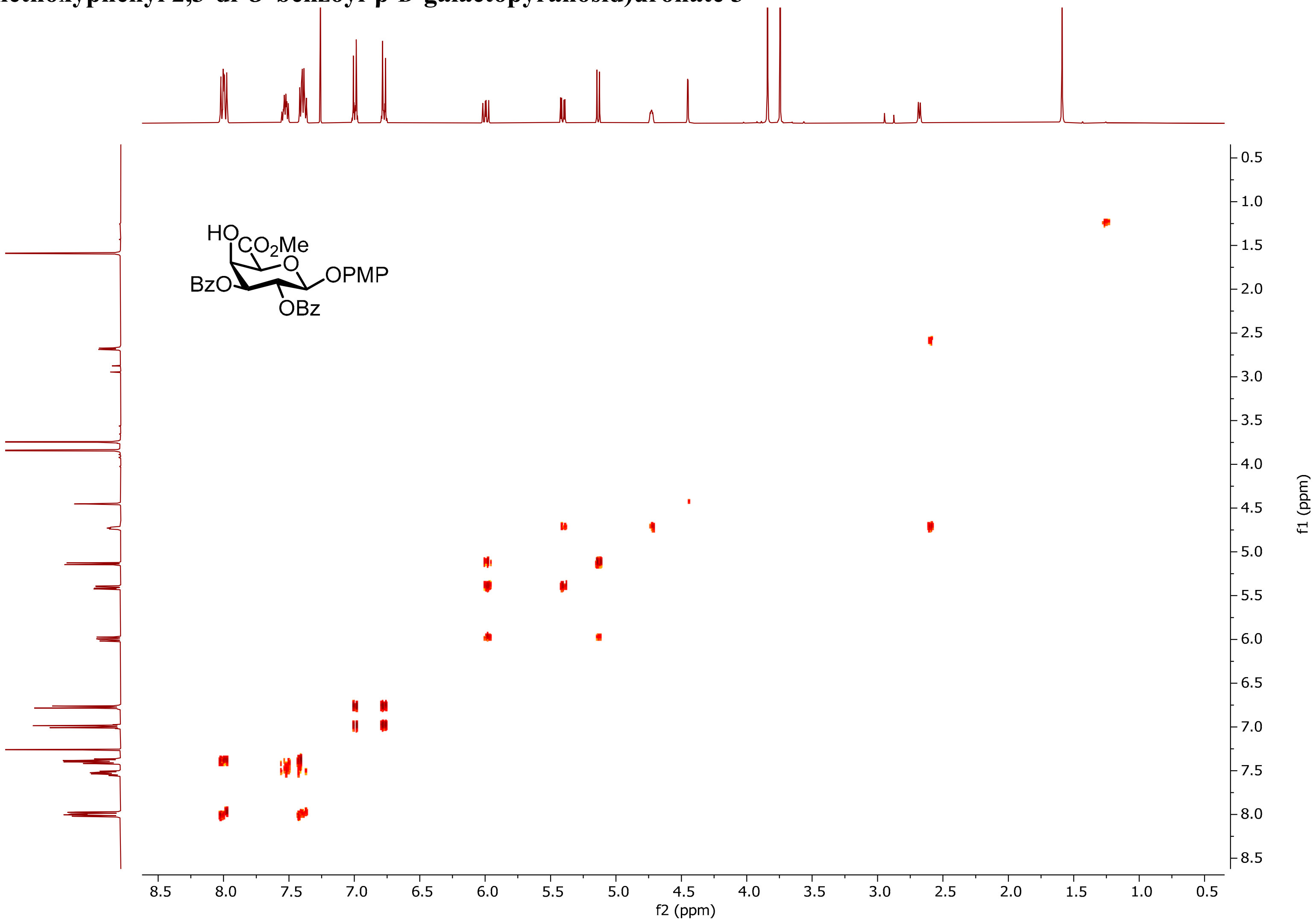

COSY NMR (400 MHz, Chloroform-*d*)

Methyl (*p*-methoxyphenyl 2,3-di-*O*-benzoyl-β-D-galactopyranosid)uronate 3

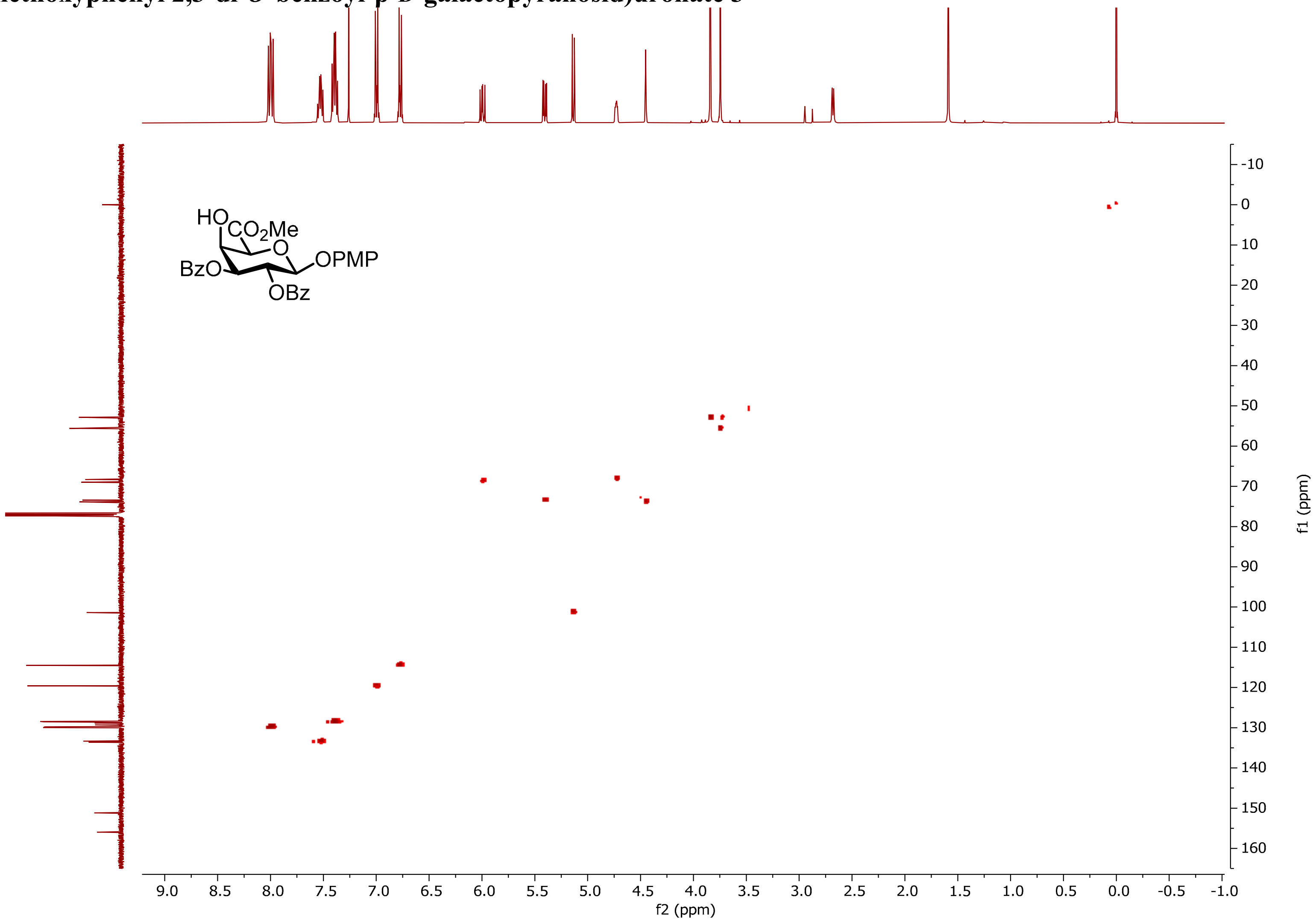

HSQC NMR (400 x 100 MHz, Chloroform-*d*)

Methyl (*p*-methoxyphenyl 2,3-di-*O*-benzoyl-β-D-galactopyranosid)uronate 3

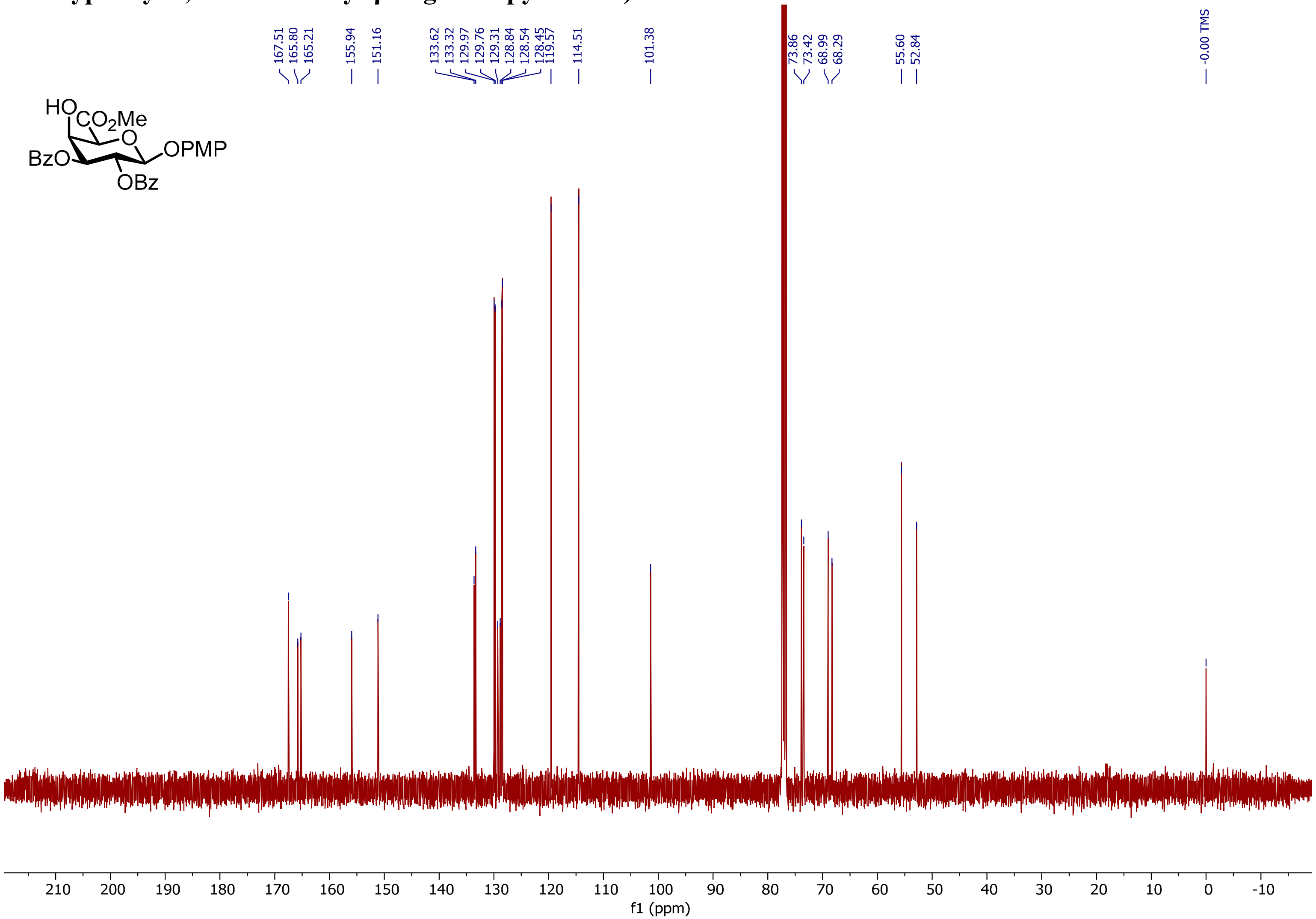

^{13}C NMR (100 MHz, Chloroform-*d*)

Methyl (*p*-methoxyphenyl 2,3-di-*O*-benzyl-4-deoxy-α-L-*threo*-hex-4-enopyranosid) uronate 4

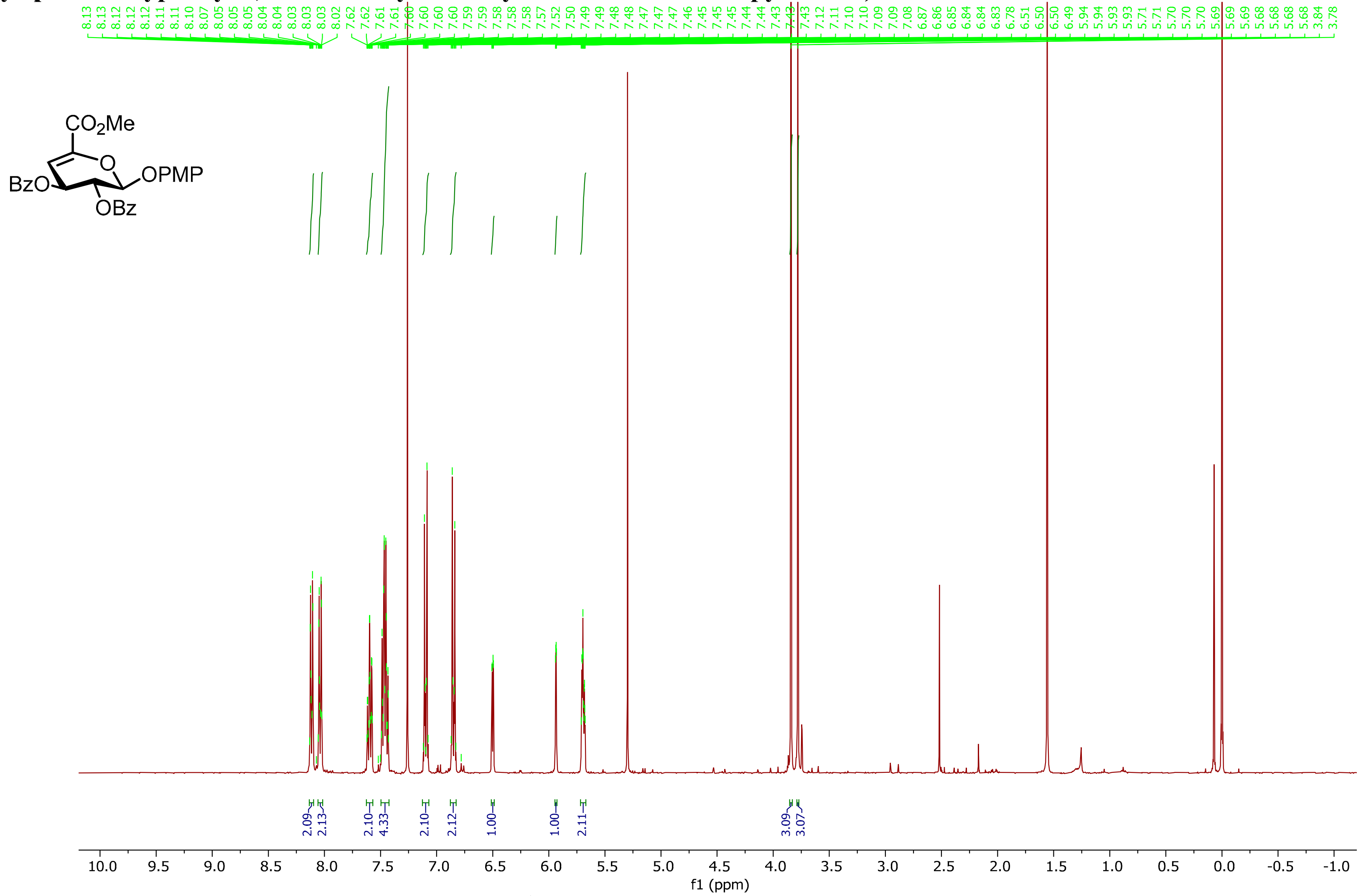

^{1}H NMR (400 MHz, Chloroform-*d*)

Methyl (*p*-methoxyphenyl 2,3-di-*O*-benzyl-4-deoxy-α-L-*threo*-hex-4-enopyranosid) uronate 4

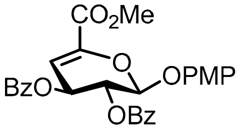

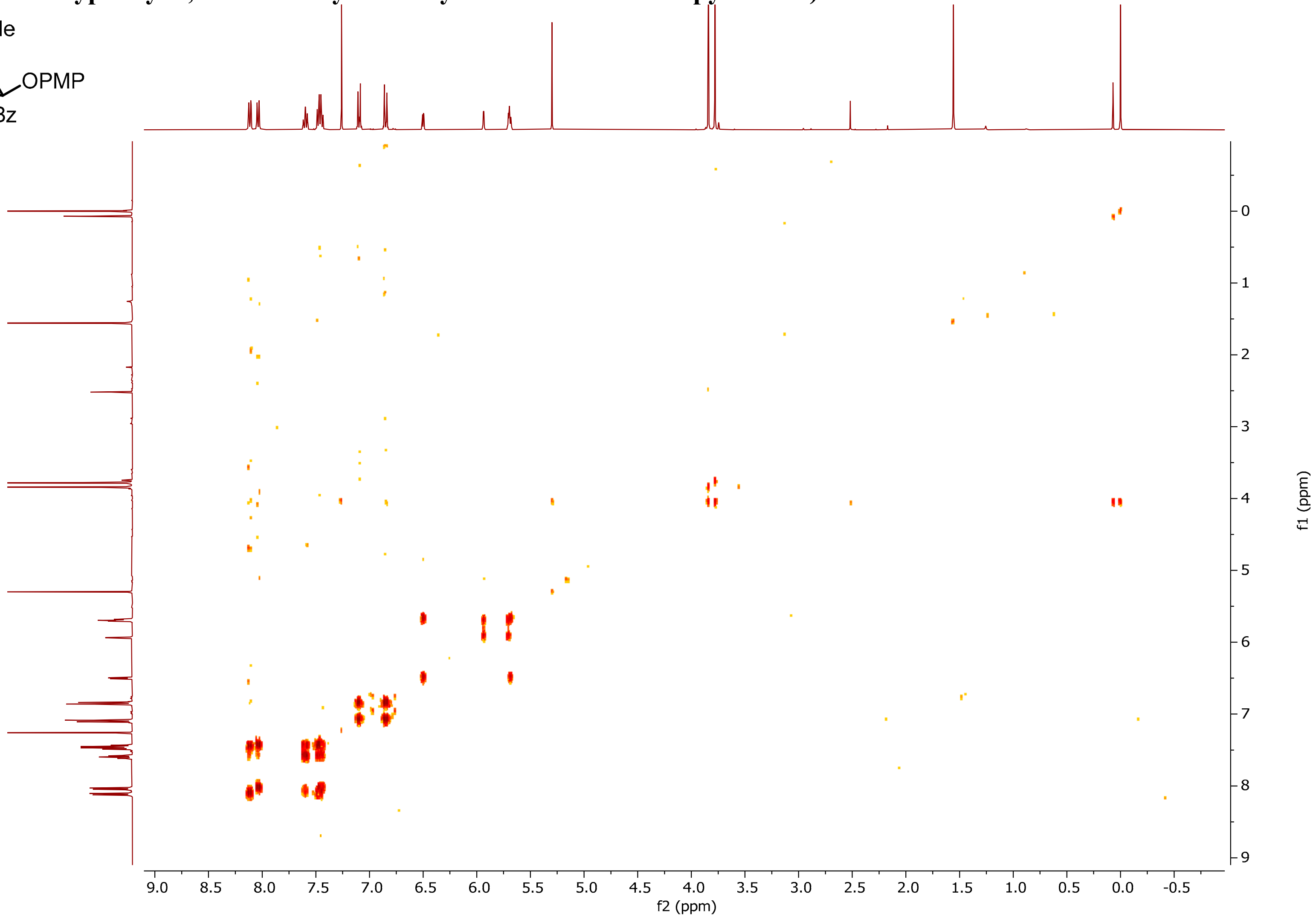

COSY NMR (400 MHz, Chloroform-*d*)

Methyl (*p*-methoxyphenyl 2,3-di-*O*-benzyl-4-deoxy-α-L-*threo*-hex-4-enopyranosid) uronate 4

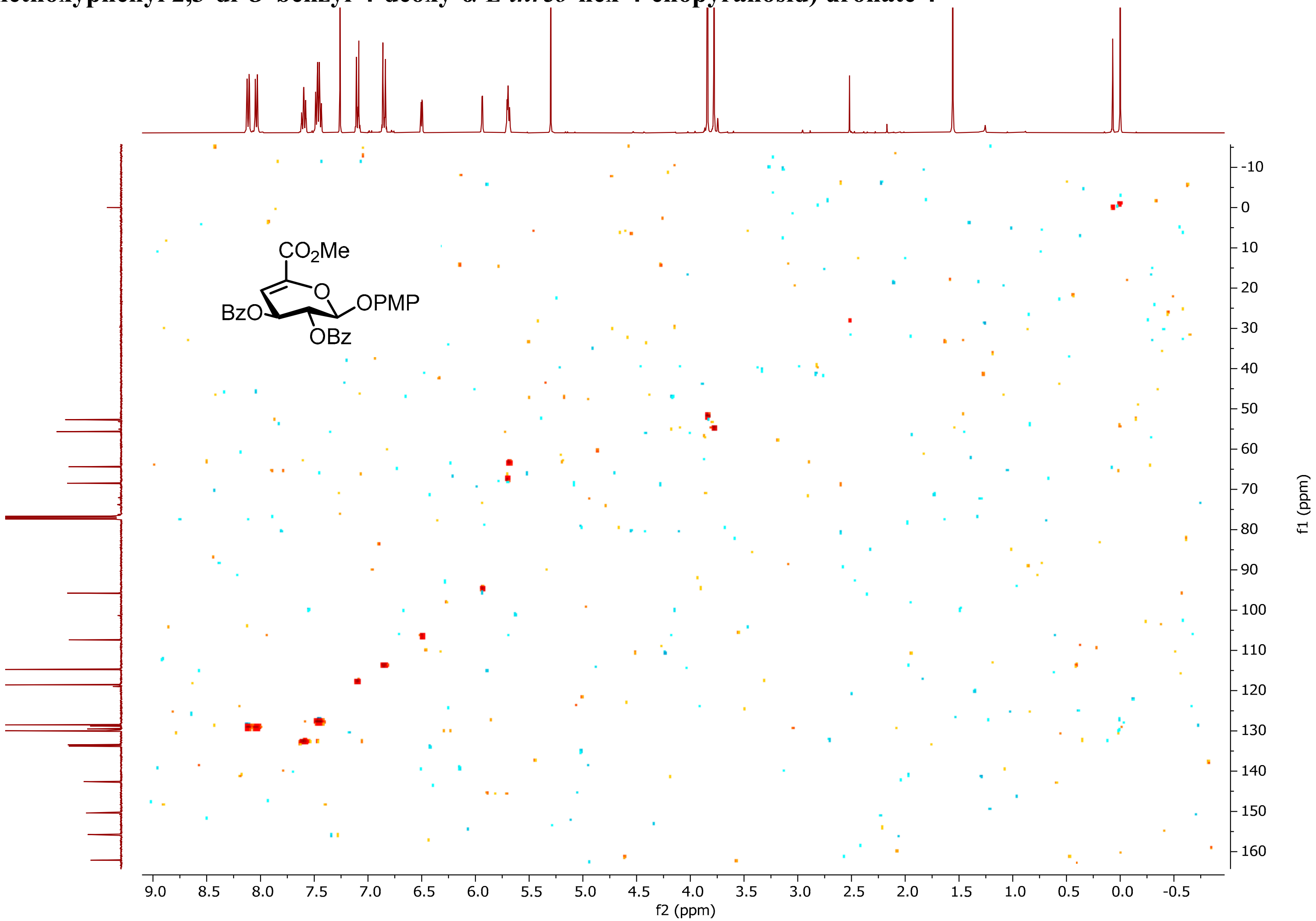

HSQC NMR (400 x 100 MHz, Chloroform-*d*)

Methyl (*p*-methoxyphenyl 2,3-di-*O*-benzyl-4-deoxy-α-L-*threo*-hex-4-enopyranosid) uronate 4

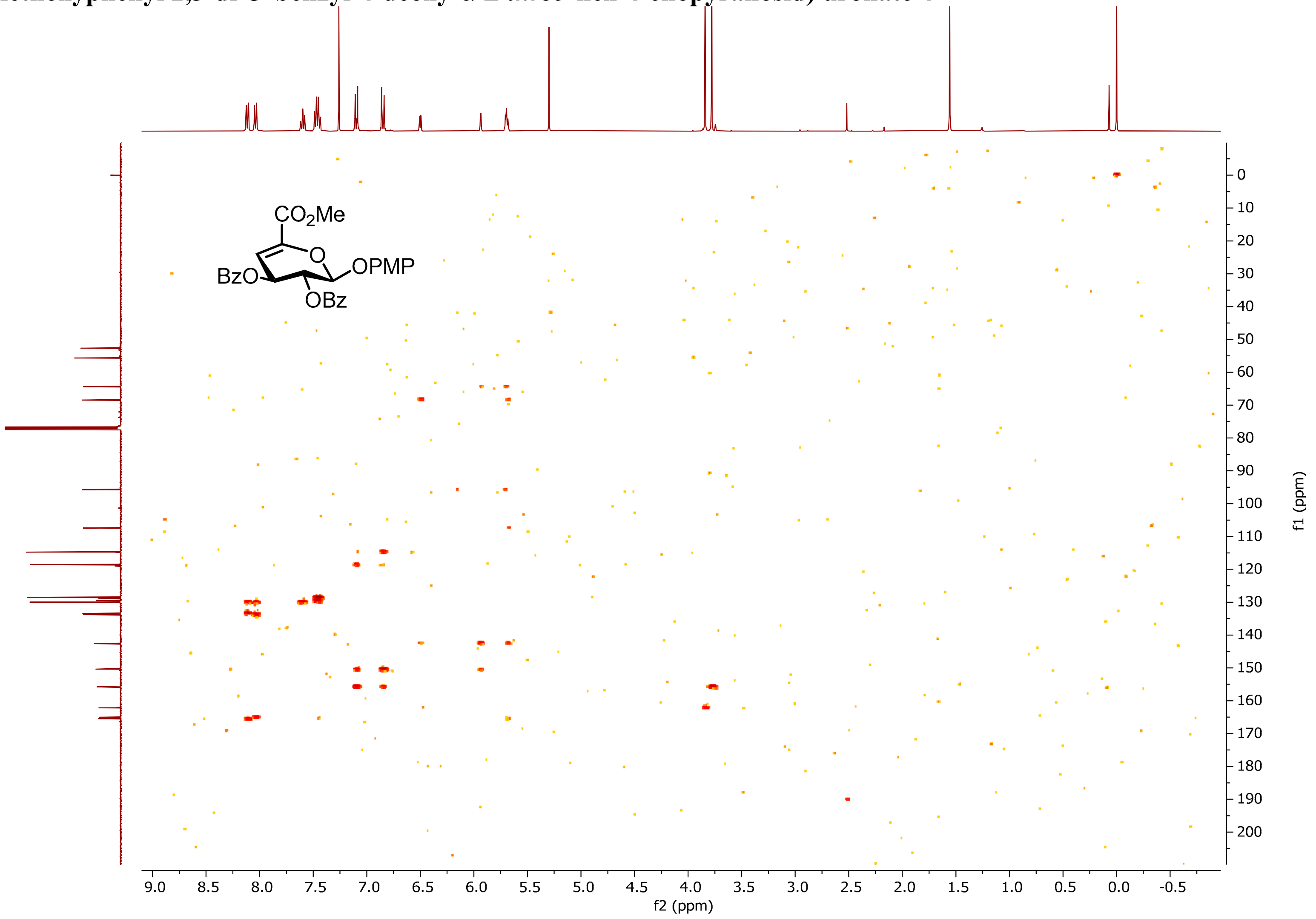

HMBC NMR (400 x 100 MHz, Chloroform-*d*)

Methyl (*p*-methoxyphenyl 2,3-di-*O*-benzyl-4-deoxy-α-L-*threo*-hex-4-enopyranosid) uronate 4

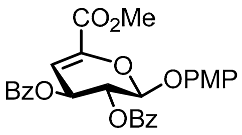

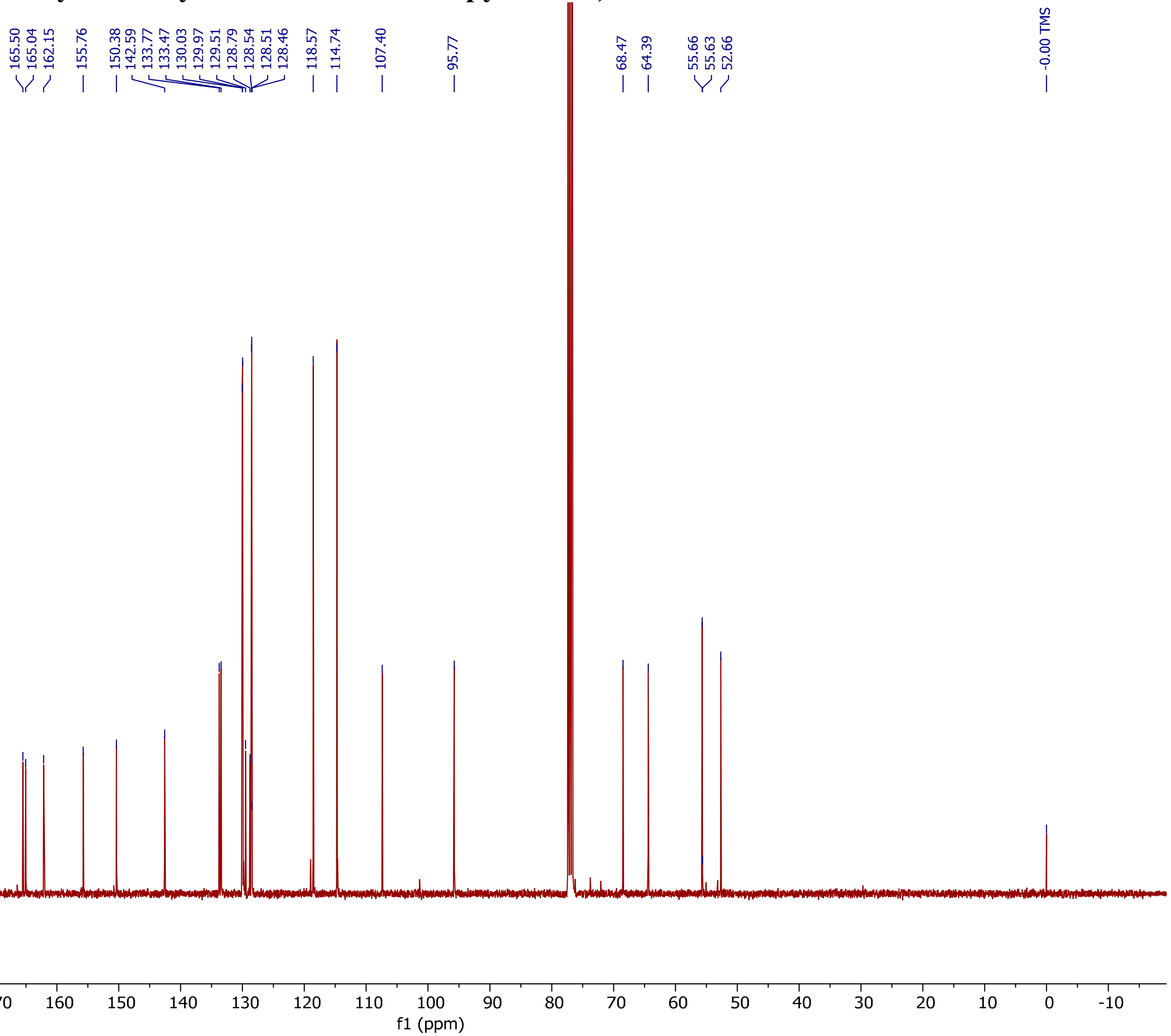

^{13}C NMR (100 MHz, Chloroform-*d*)

p-Methoxyphenyl 2,3-di-O-benzoyl-6-O-(triisopropylsilyl)-β-D-galactopyranoside 5

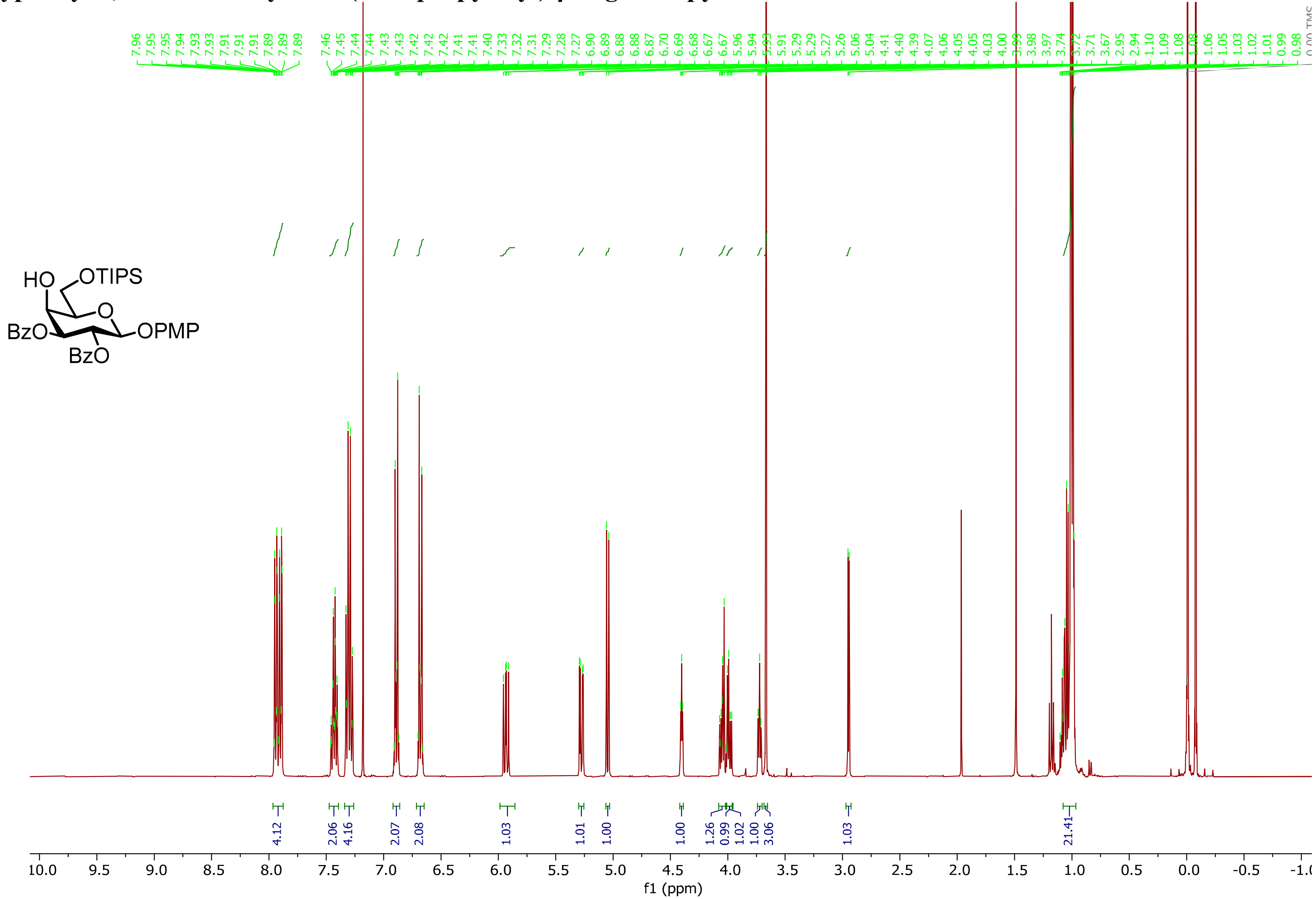

^{1}H NMR (400 MHz, Chloroform-*d*)

_p_-Methoxyphenyl 2,3-di-_O_-benzoyl-6-_O_-(triisopropylsilyl)-β-D-galactopyranoside 5

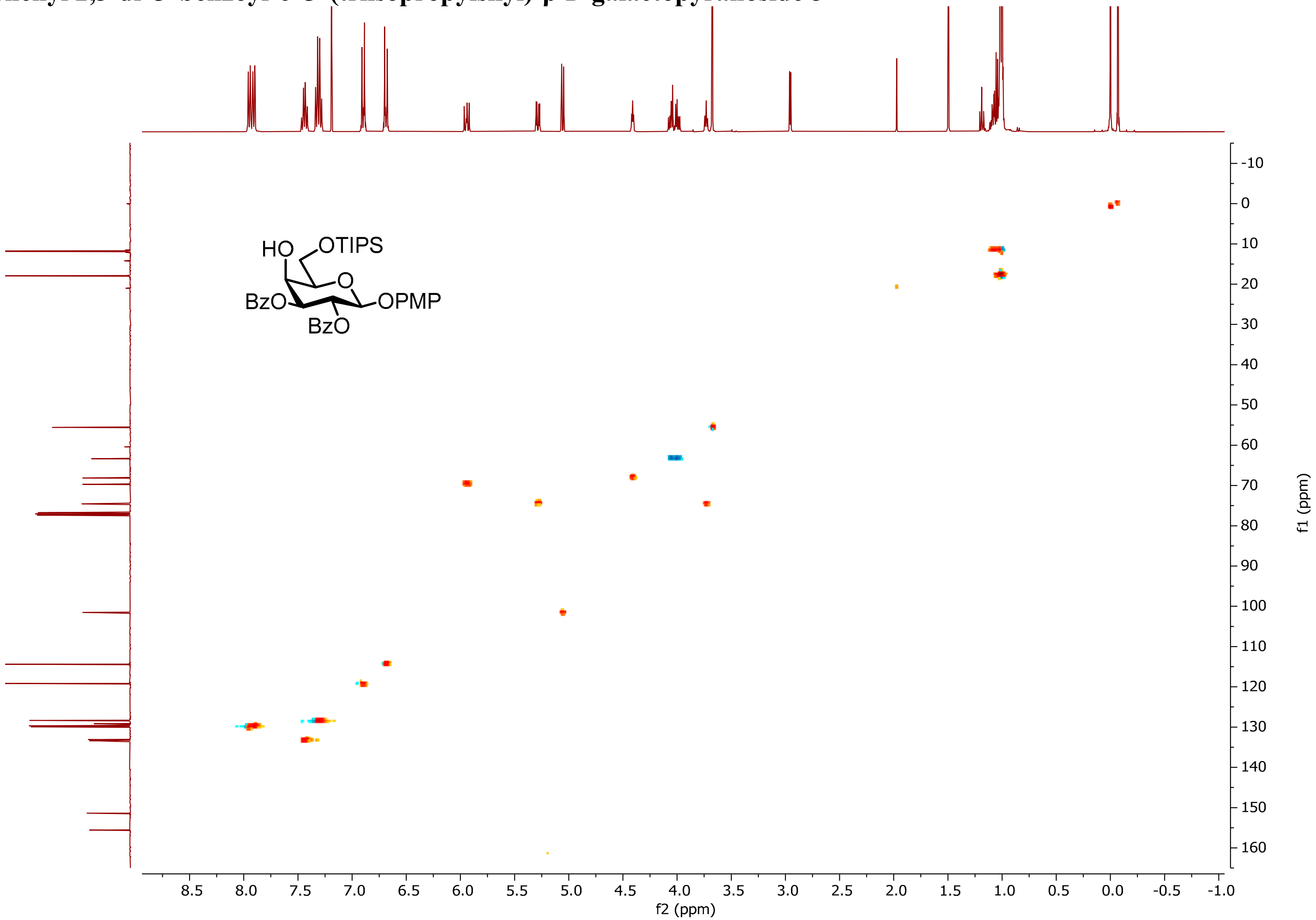

HSQC NMR (400 x 100 MHz, Chloroform-_d_)

p-Methoxyphenyl 2,3-di-O-benzoyl-6-O-(triisopropylsilyl)-β-D-galactopyranoside 5

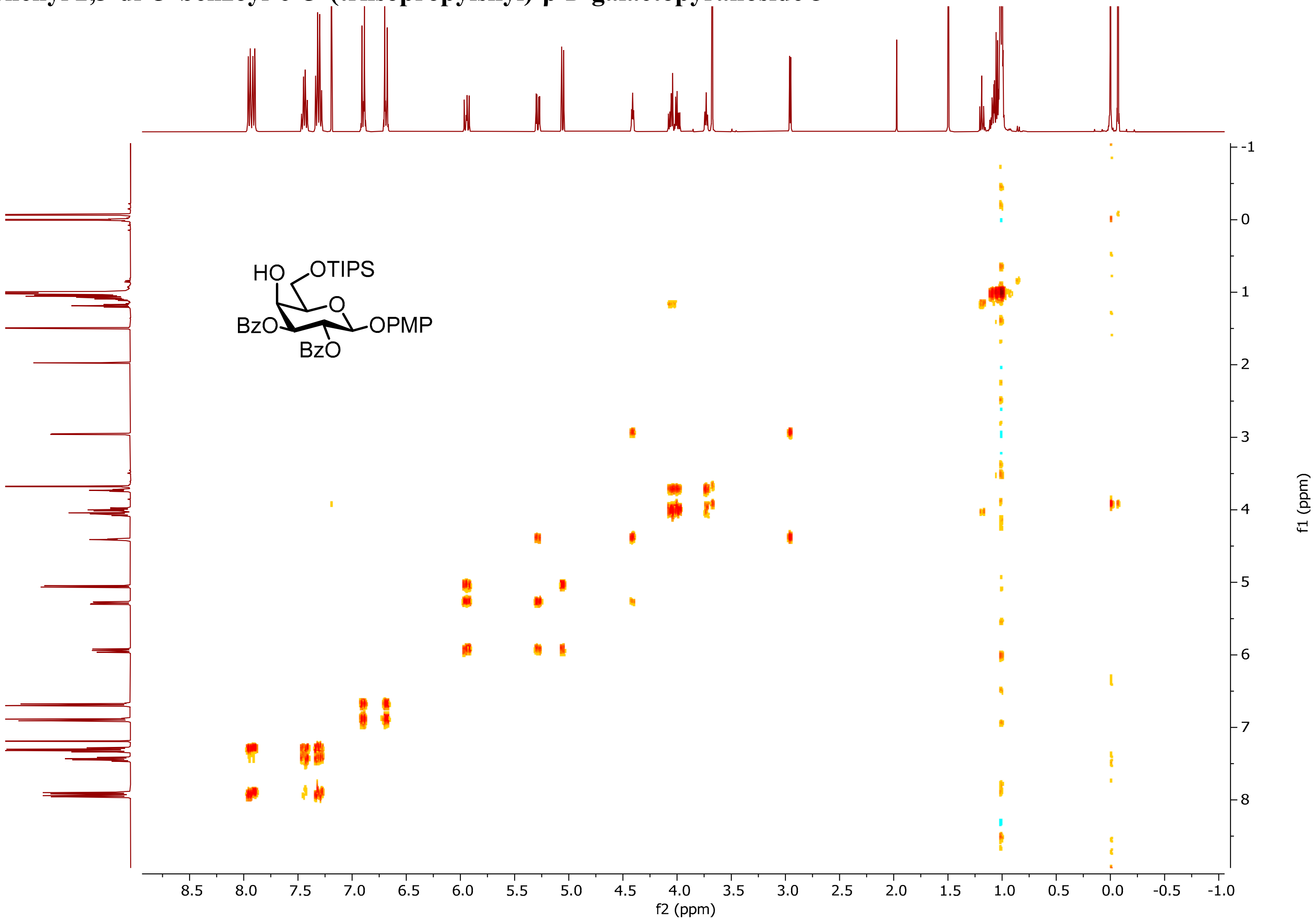

COSY NMR (400 MHz, Chloroform-*d*)

_p_-Methoxyphenyl 2,3-di-_O_-benzoyl-6-_O_-(triisopropylsilyl)-β-D-galactopyranoside 5

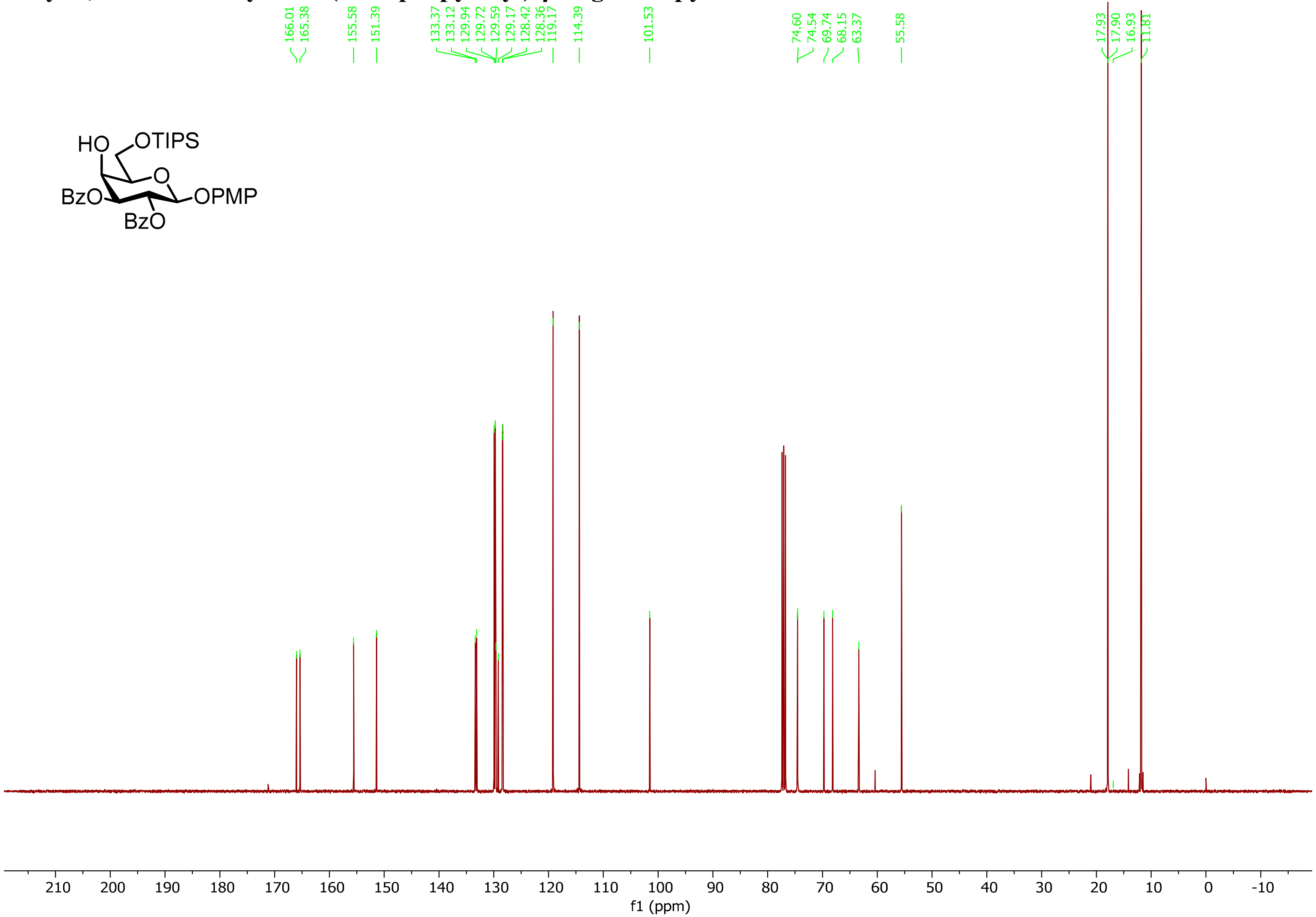

^{13}C NMR (100 MHz, Chloroform-*d*)

_p_-Methoxyphenyl 2,4-di-_O_-benzoyl-6-_O_-(triisopropylsilyl)-β-D-galactopyranoside S11

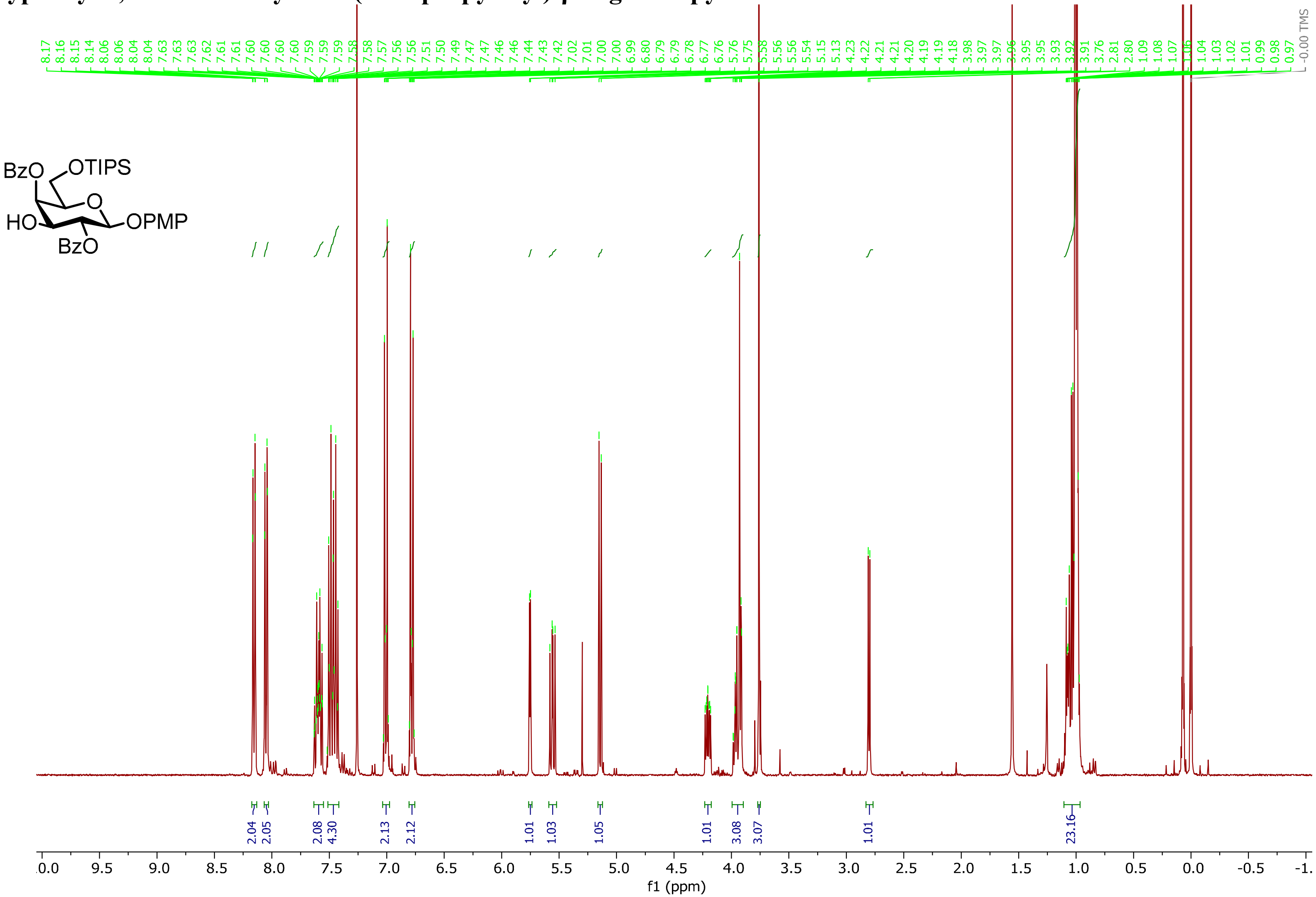

^{1}H NMR (400 MHz, Chloroform-_d_)

***p*-Methoxyphenyl 2,4-di-*O*-benzoyl-6-*O*-(triisopropylsilyl)-β-D-galactopyranoside S11**

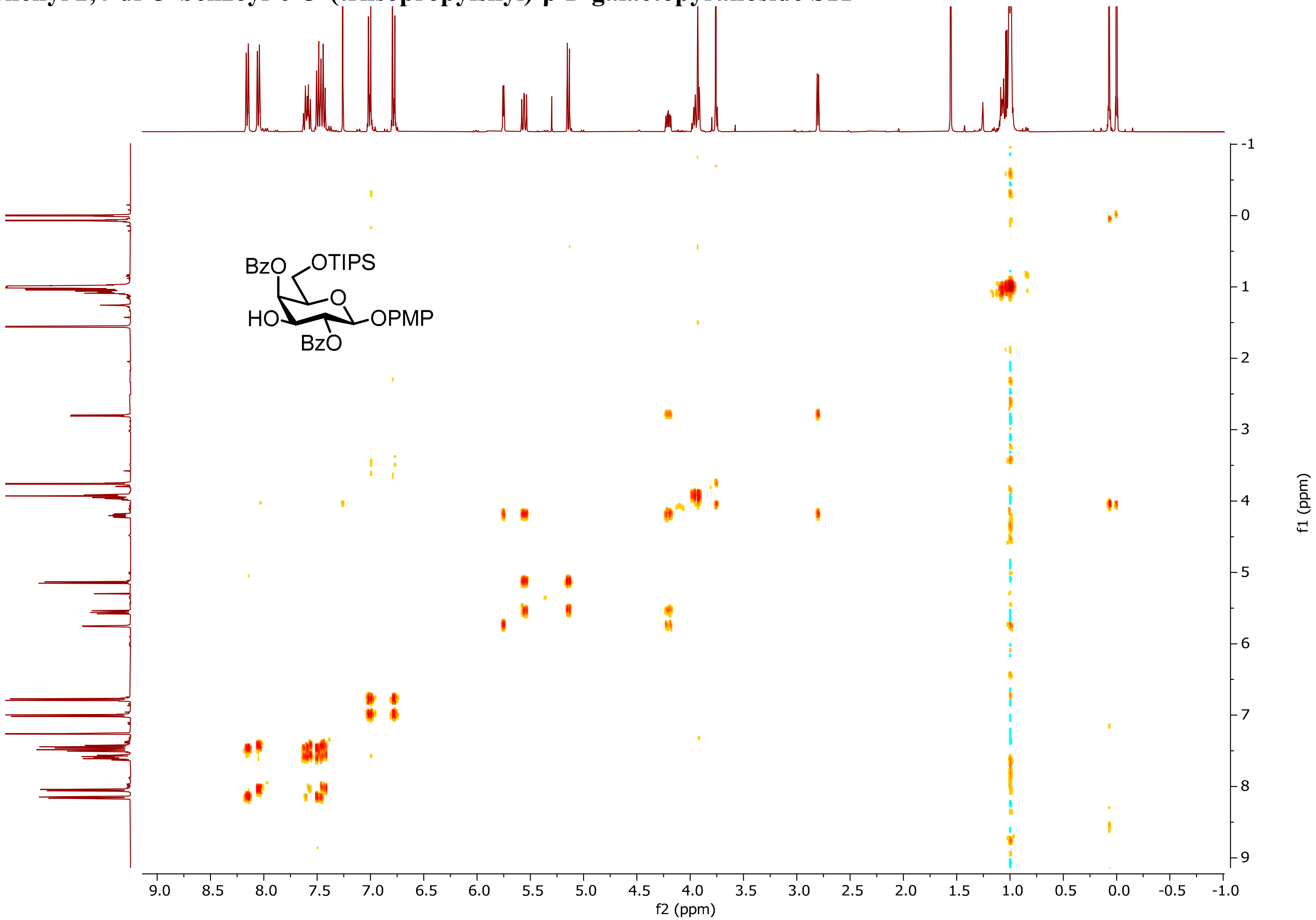

COSY NMR (400 MHz, Chloroform-*d*)

p-Methoxyphenyl 2,4-di-O-benzoyl-6-O-(triisopropylsilyl)-β-D-galactopyranoside S11

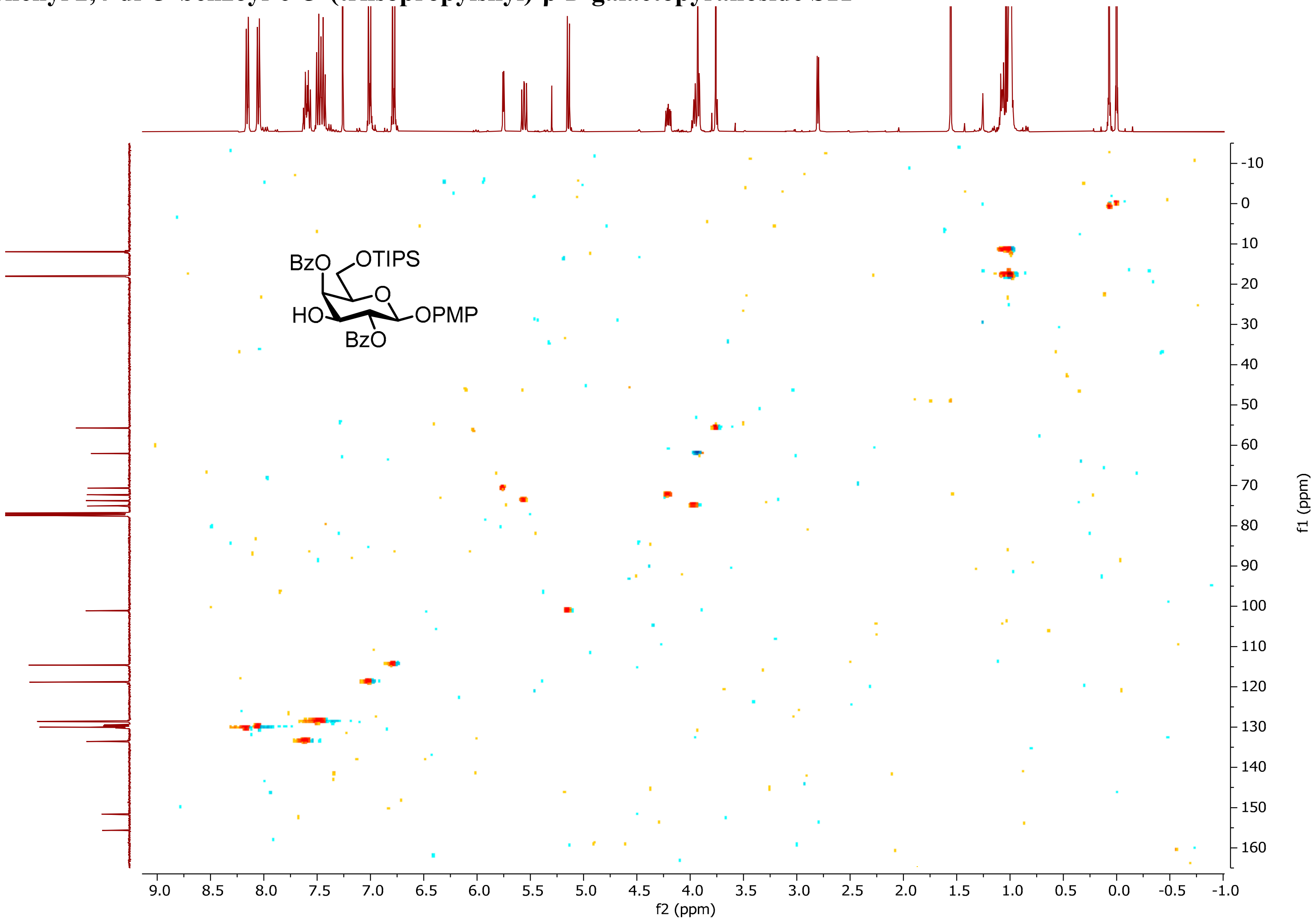

HSQC NMR (400 x 100 MHz, Chloroform-*d*)

***p*-Methoxyphenyl 2,4-di-*O*-benzoyl-6-*O*-(triisopropylsilyl)-β-D-galactopyranoside S11**

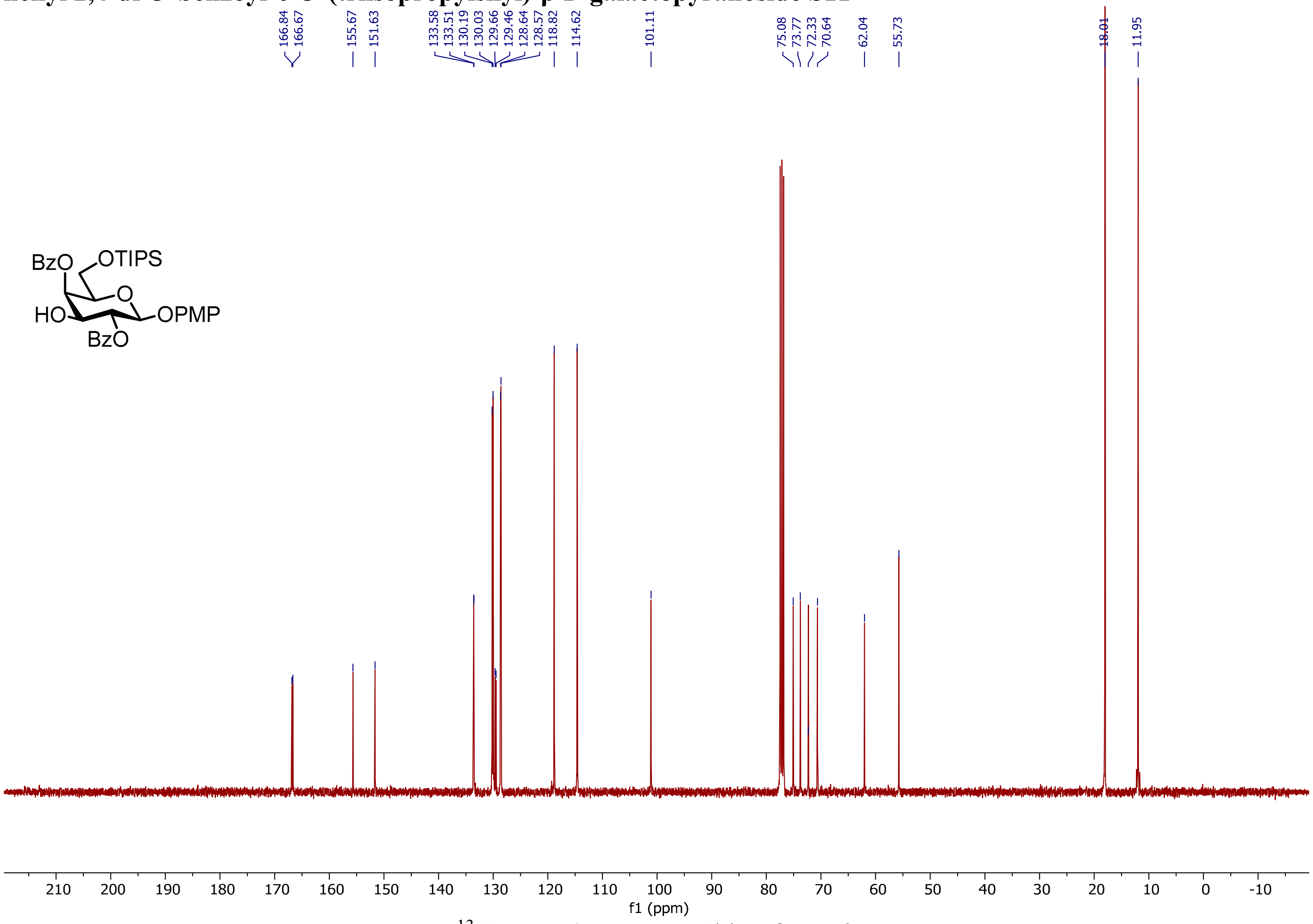

^{13}C NMR (100 MHz, Chloroform-*d*)

_p_-Methoxyphenyl 4-_S_-acetyl-2,3-di-_O_-benzoyl-6-_O_-(triisopropylsilyl)-β-D-glucopyranoside 9

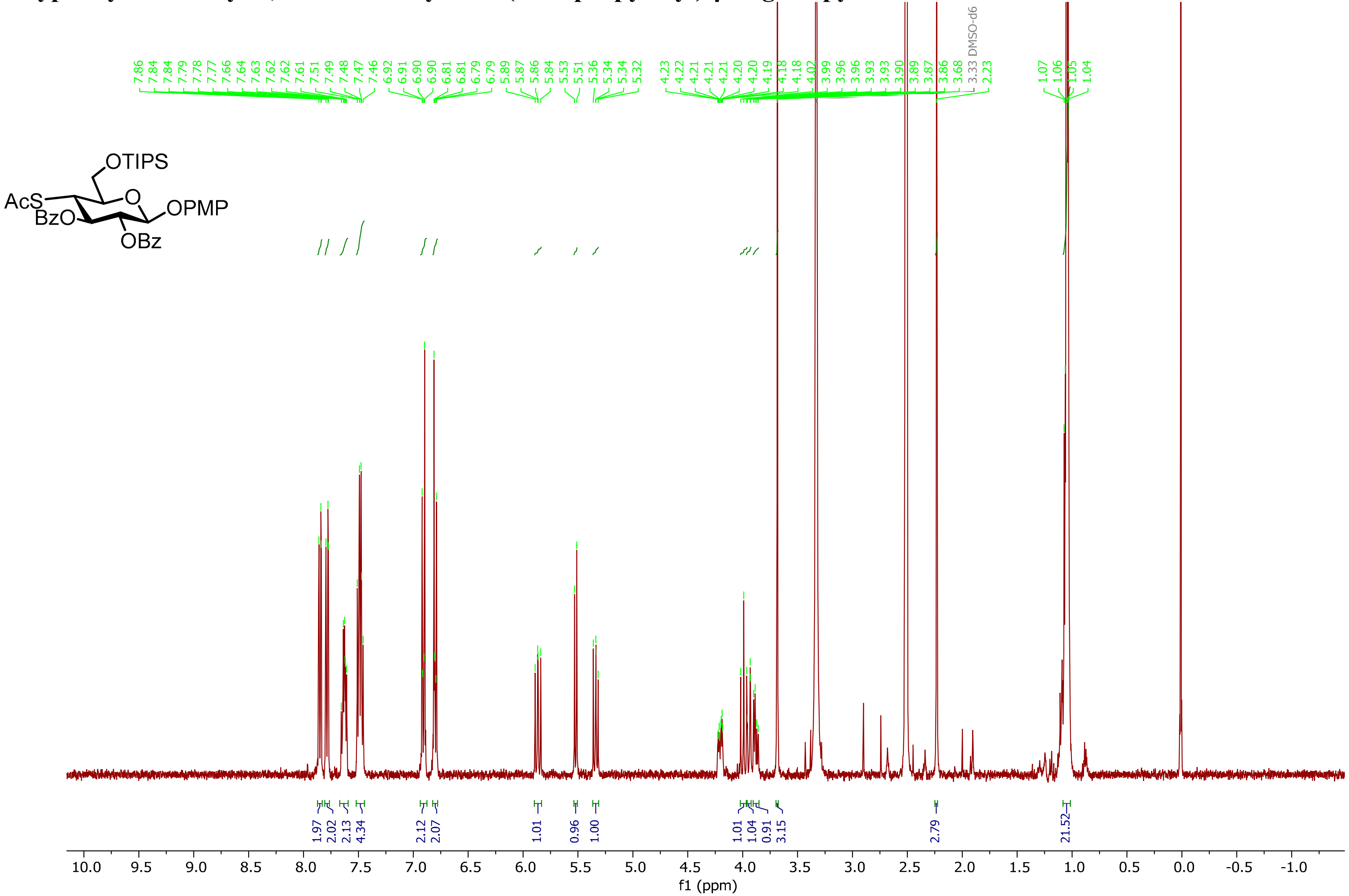

^{1}H NMR (400 MHz, DMSO-d^{6})

p-Methoxyphenyl 4-S-acetyl-2,3-di-O-benzoyl-6-O-(triisopropylsilyl)-β-D-glucopyranoside 9

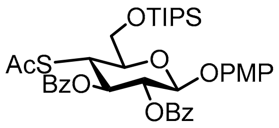

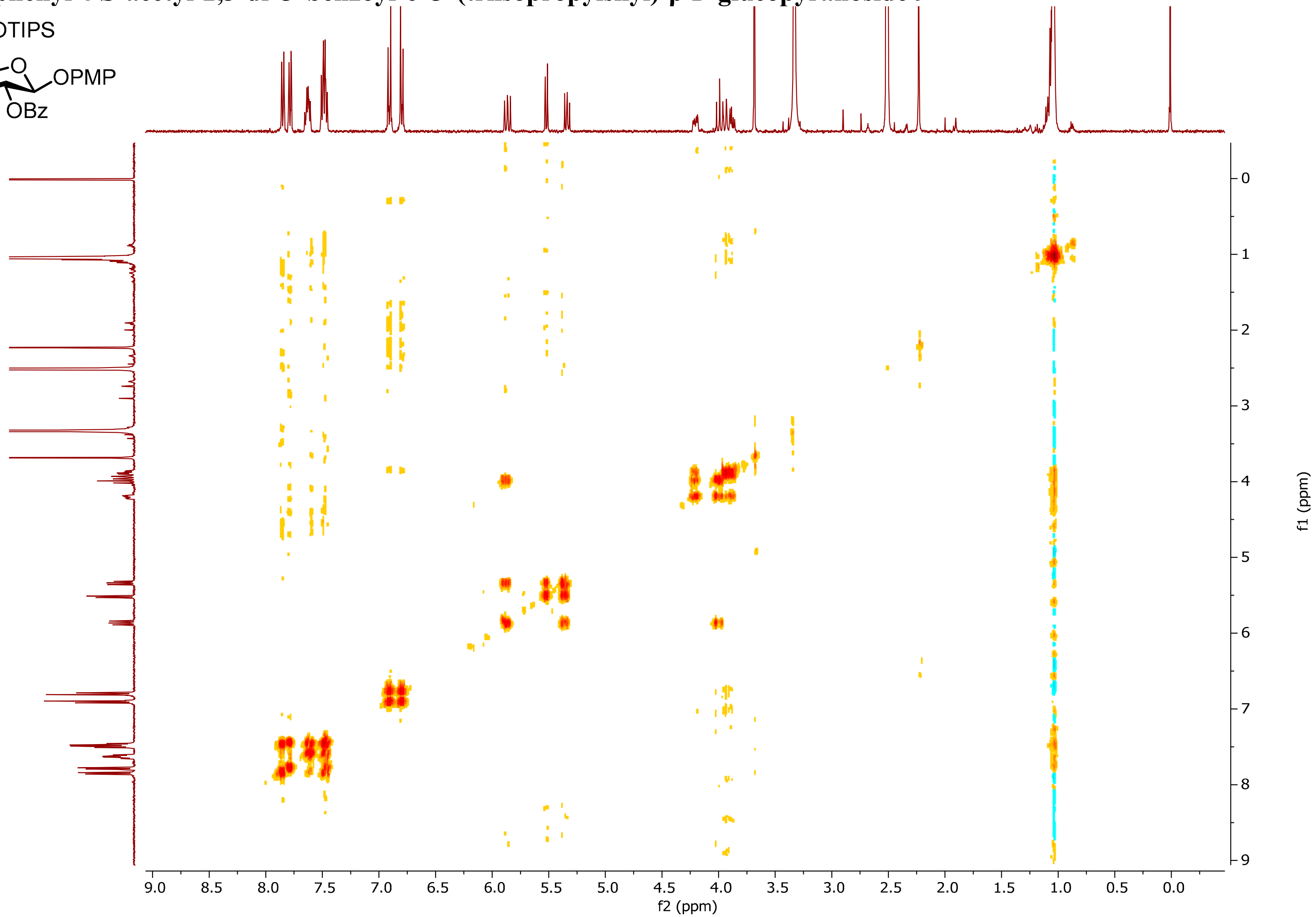

COSY NMR (400 MHz, DMSO-d^6)

p-Methoxyphenyl 4-S-acetyl-2,3-di-O-benzoyl-6-O-(triisopropylsilyl)-β-D-glucopyranoside 9

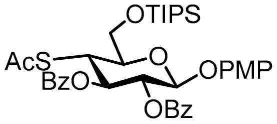

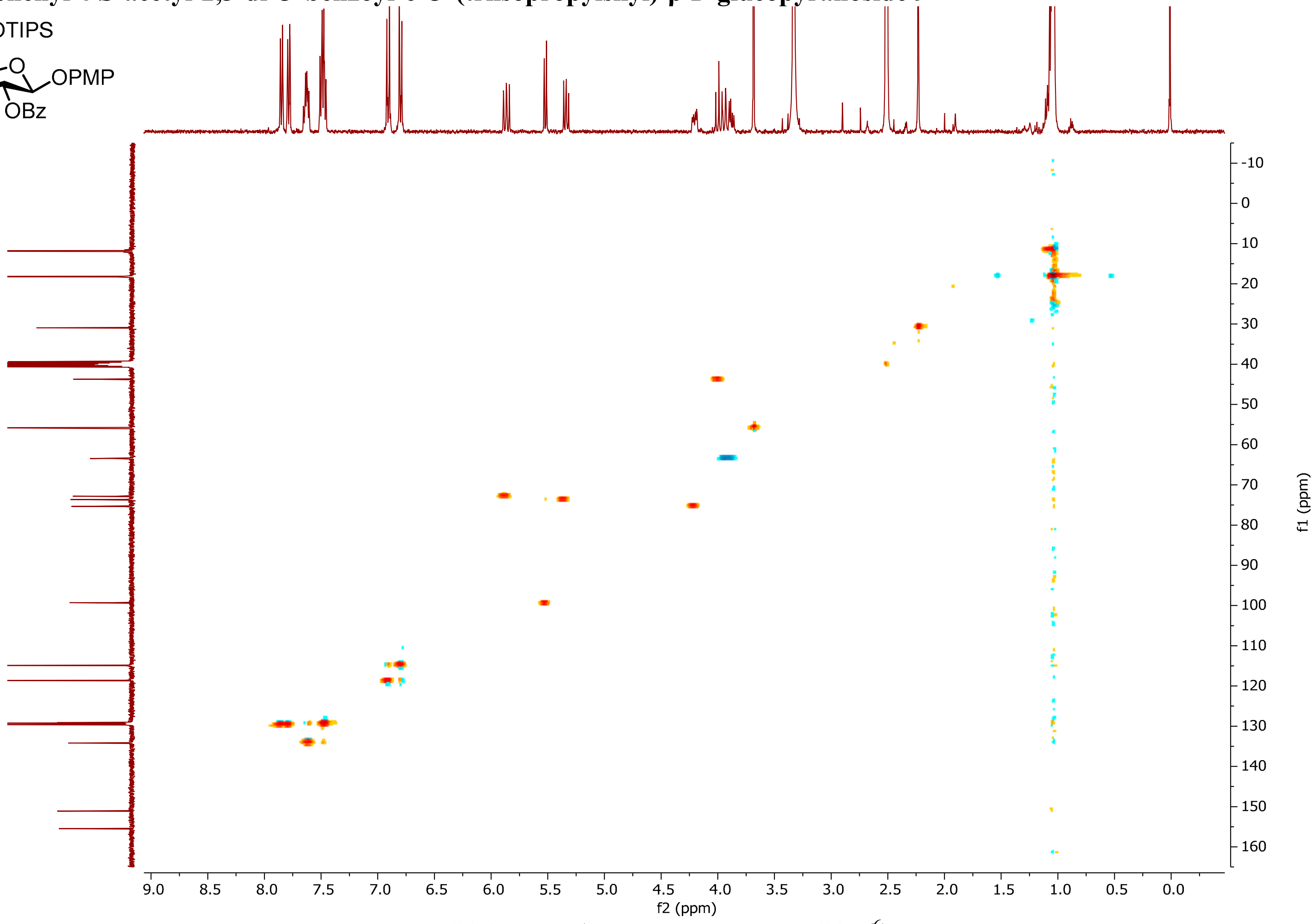

HSQC NMR (400 x 100 MHz, DMSO-d^6)

p-Methoxyphenyl 4-S-acetyl-2,3-di-O-benzoyl-6-O-(triisopropylsilyl)-β-D-glucopyranoside 9

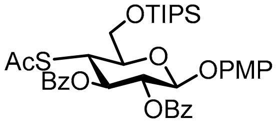

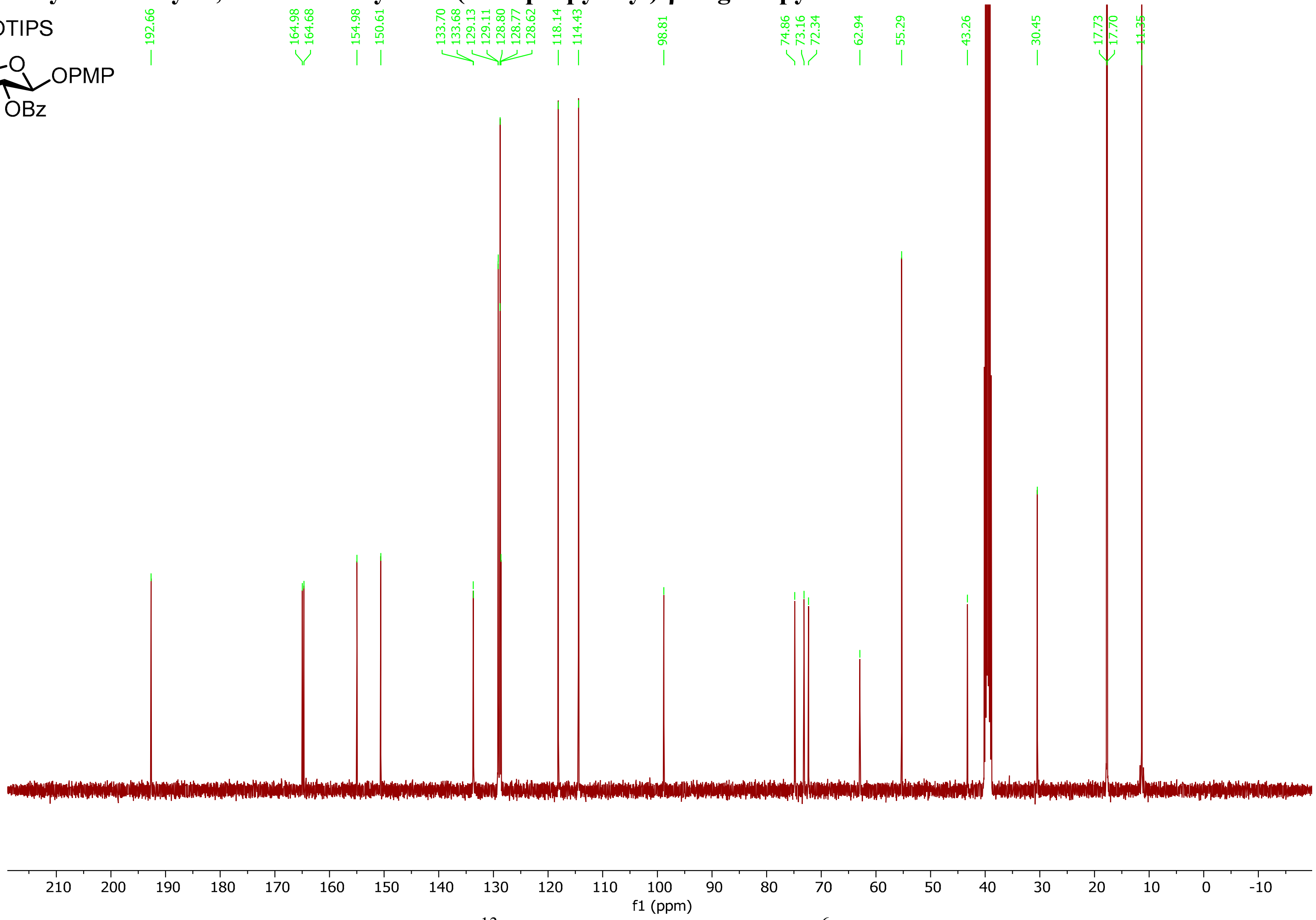

^{13}C NMR (100 MHz, DMSO-d^6)

_p_-Methoxyphenyl 2,3-di-_O_-benzoyl-4-thio-6-_O_-(triisopropylsilyl)-β-D-glucopyranoside 11

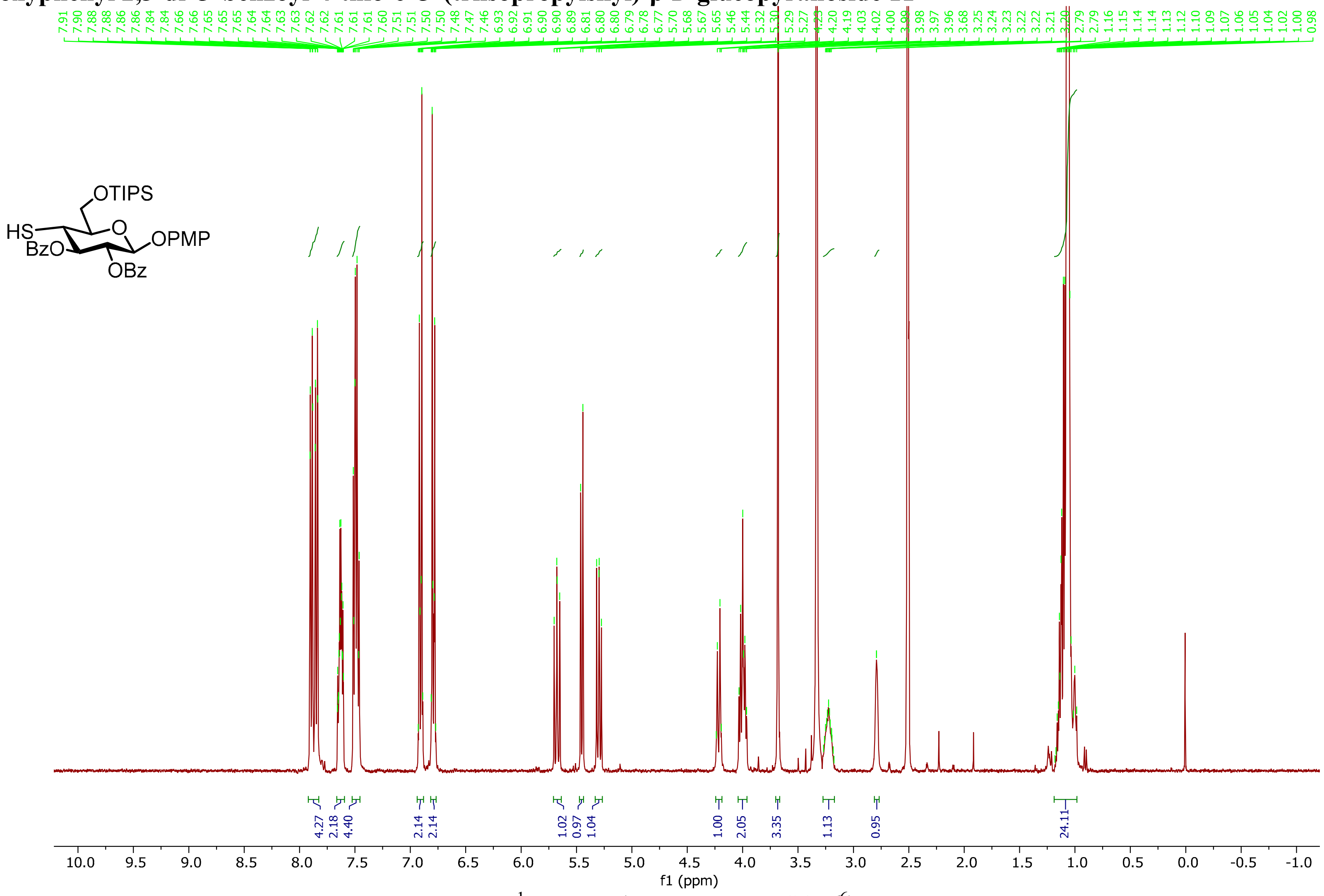

^{1}H NMR (400 MHz, DMSO-d^6)

_p_-Methoxyphenyl 2,3-di-_O_-benzoyl-4-thio-6-_O_-(triisopropylsilyl)-β-D-glucopyranoside 11

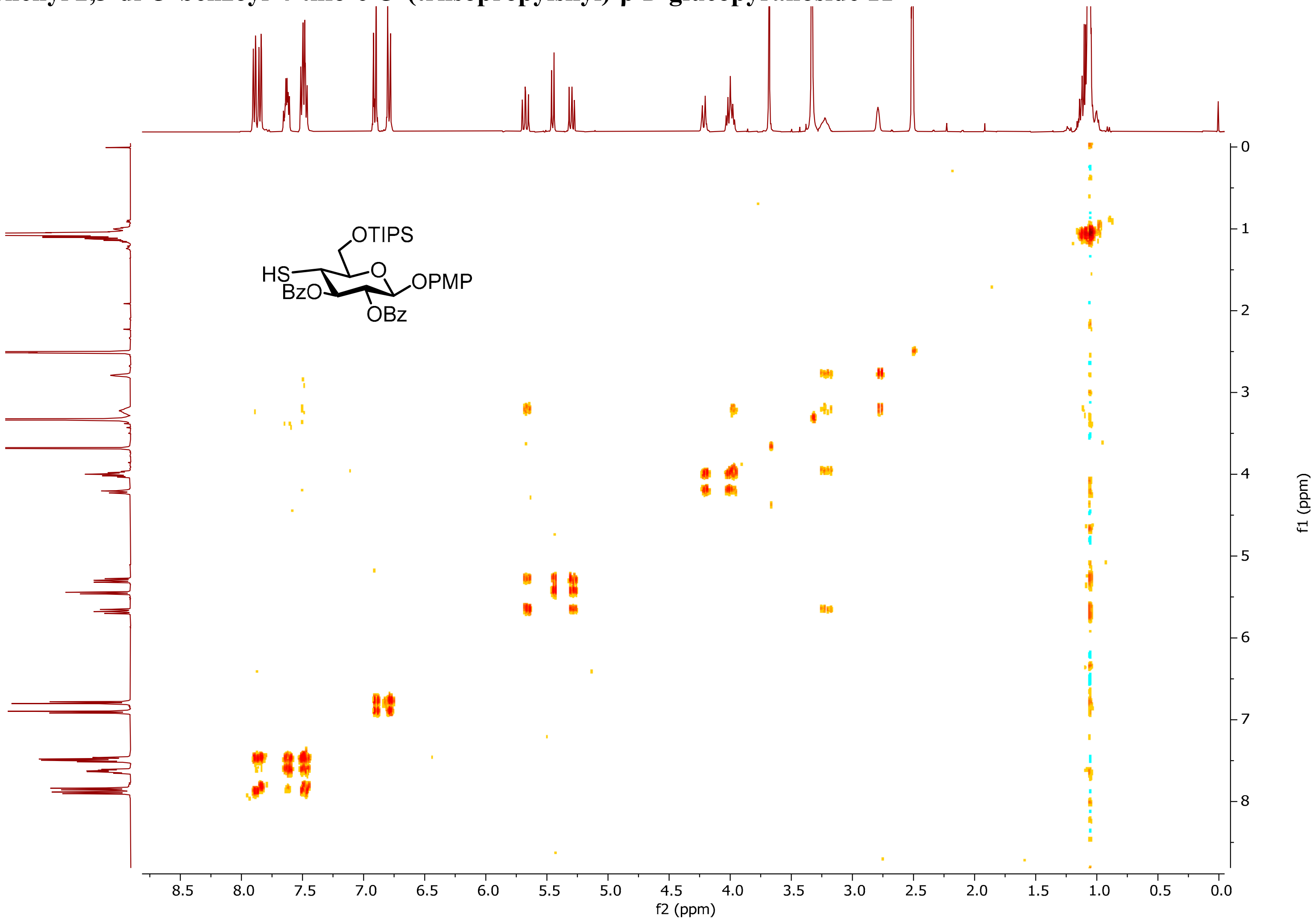

COSY NMR (400 MHz, DMSO-d^6)

_p_-Methoxyphenyl 2,3-di-_O_-benzoyl-4-thio-6-_O_-(triisopropylsilyl)-β-D-glucopyranoside 11

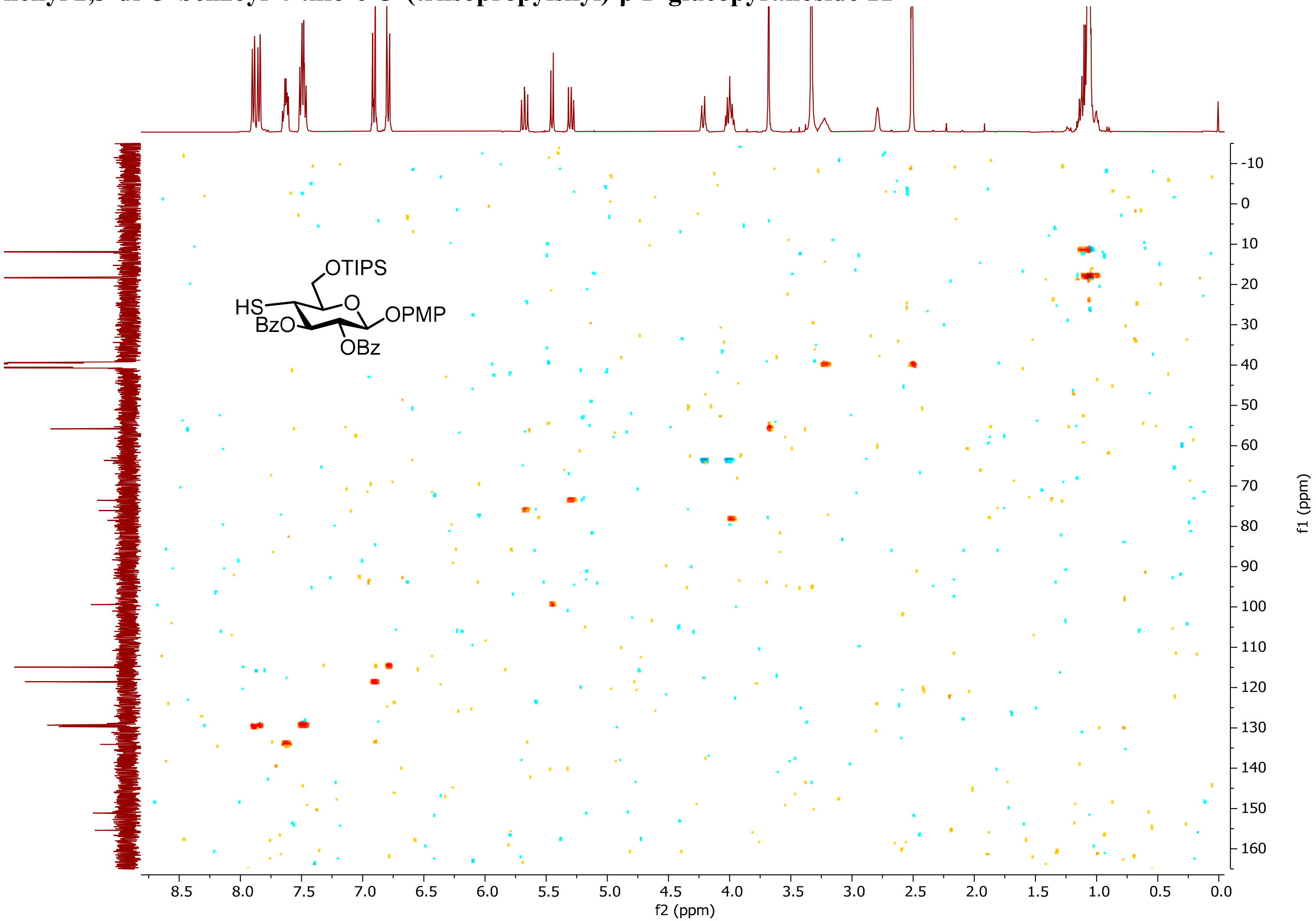

HSQC NMR (400 x 100 MHz, DMSO-d^6)

_p_-Methoxyphenyl 2,3-di-_O_-benzoyl-4-thio-6-_O_-(triisopropylsilyl)-β-D-glucopyranoside 11

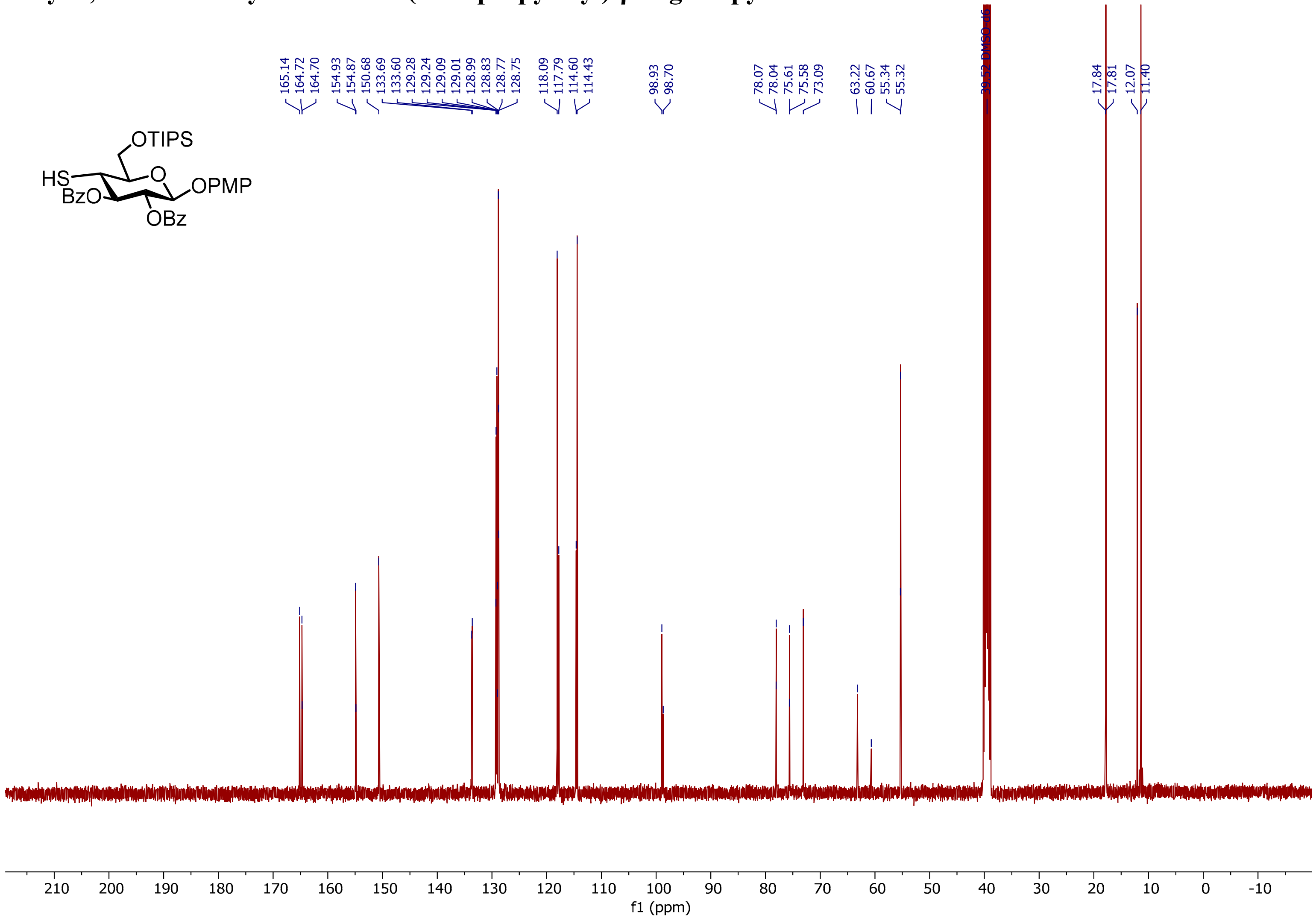

^{13}C NMR (100 MHz, DMSO-d^6)

p-Methoxyphenyl 4-S-acetyl-2,3-di-O-benzoyl-β-D-glucopyranoside 13

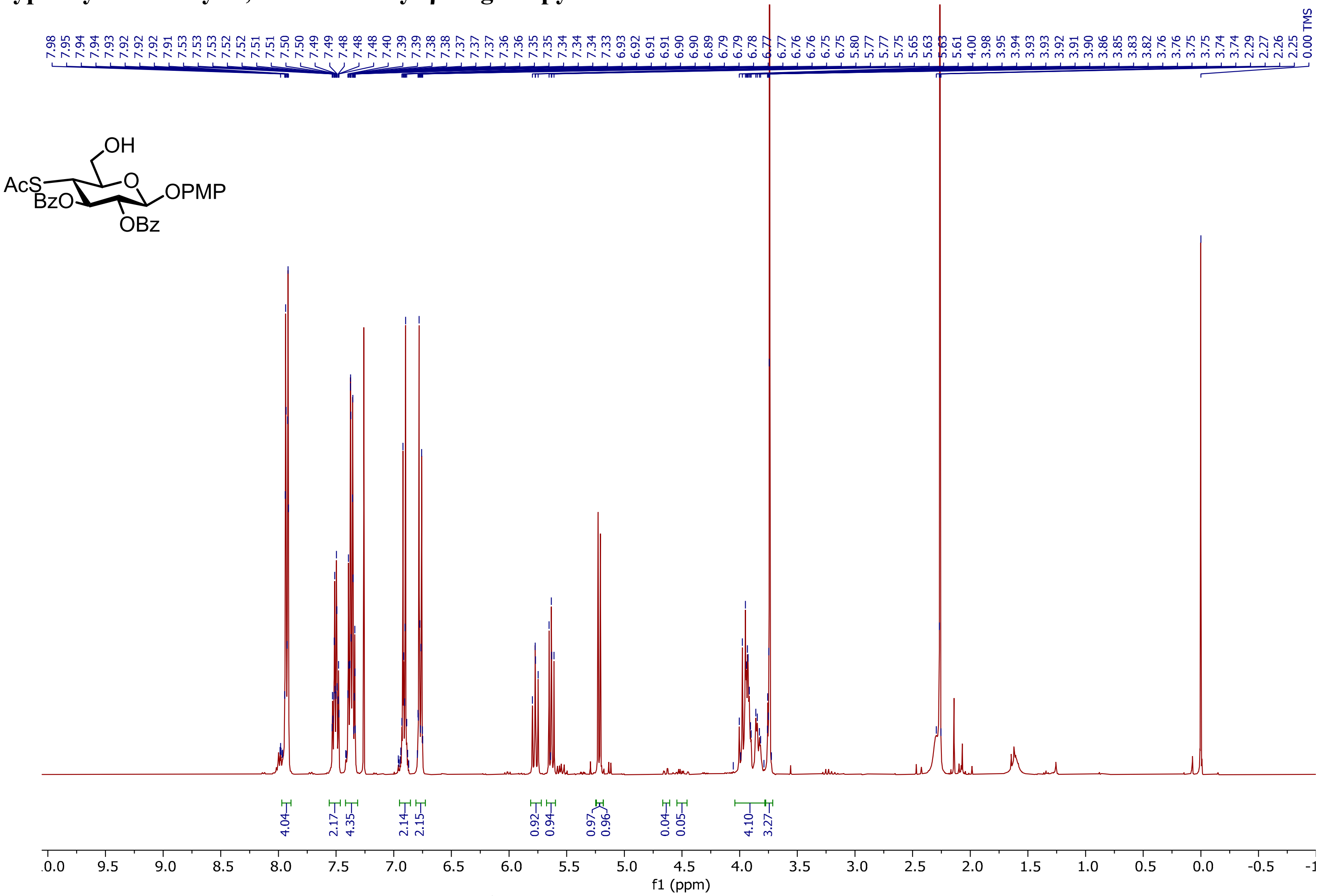

^{1}H NMR (400 MHz, Chloroform-*d*)

***p*-Methoxyphenyl 4-*S*-acetyl-2,3-di-*O*-benzoyl-β-D-glucopyranoside 13**

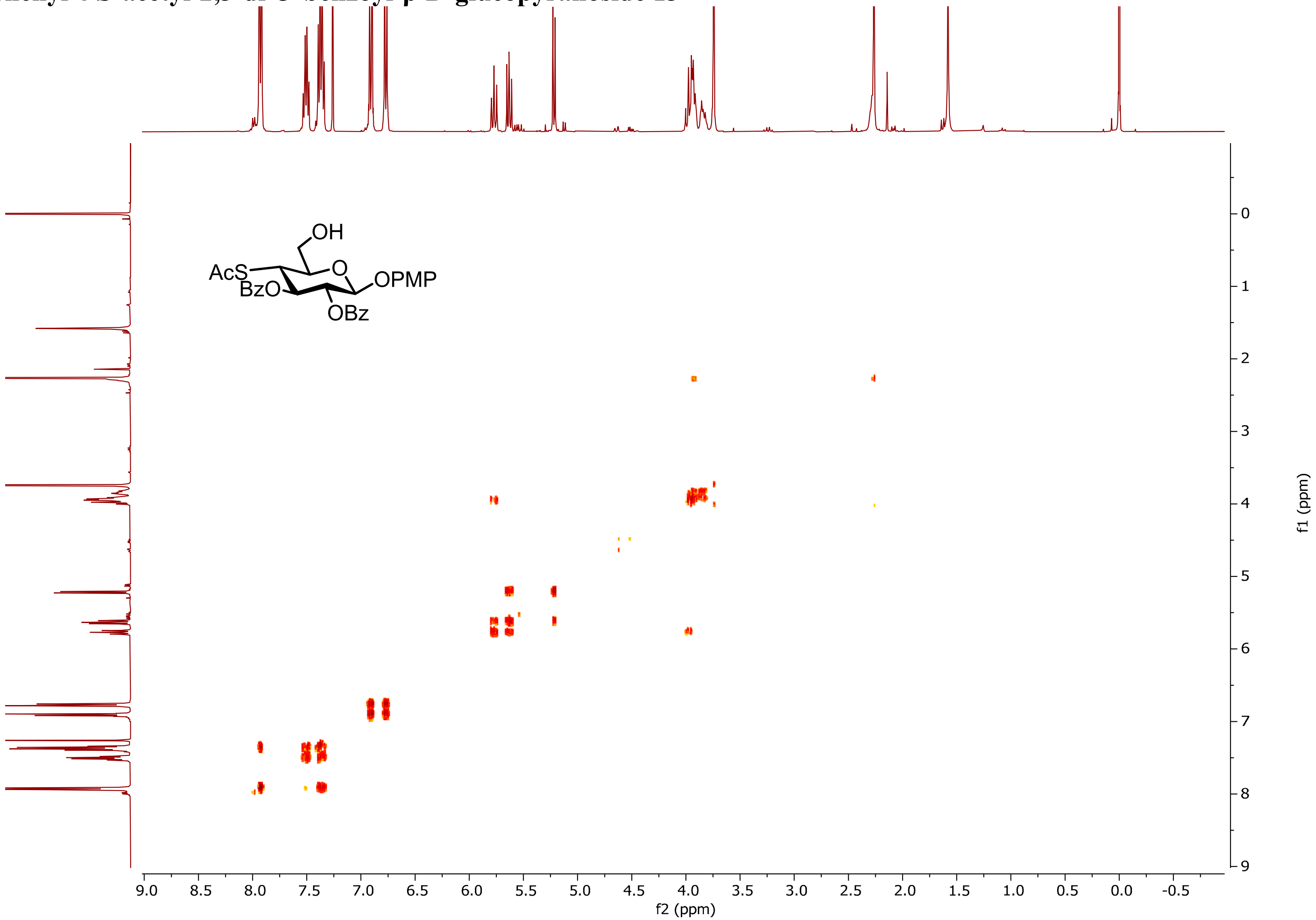

COSY NMR (400 MHz, Chloroform-*d*)

p-Methoxyphenyl 4-S-acetyl-2,3-di-O-benzoyl-β-D-glucopyranoside 13

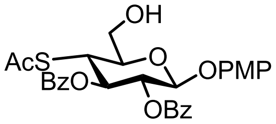

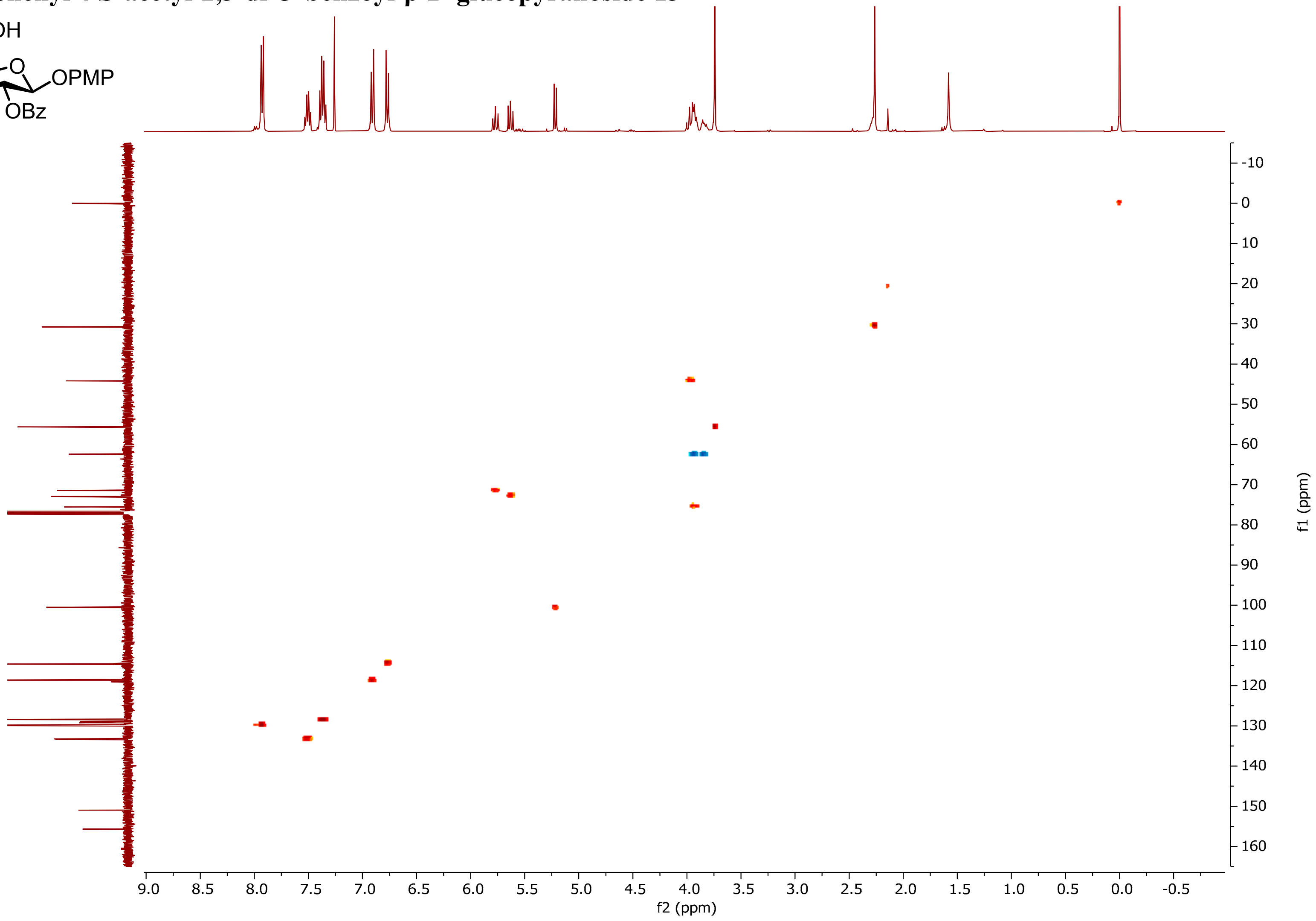

HSQC NMR (400 x 100 MHz, Chloroform-*d*)

p-Methoxyphenyl 4-S-acetyl-2,3-di-O-benzoyl-β-D-glucopyranoside 13

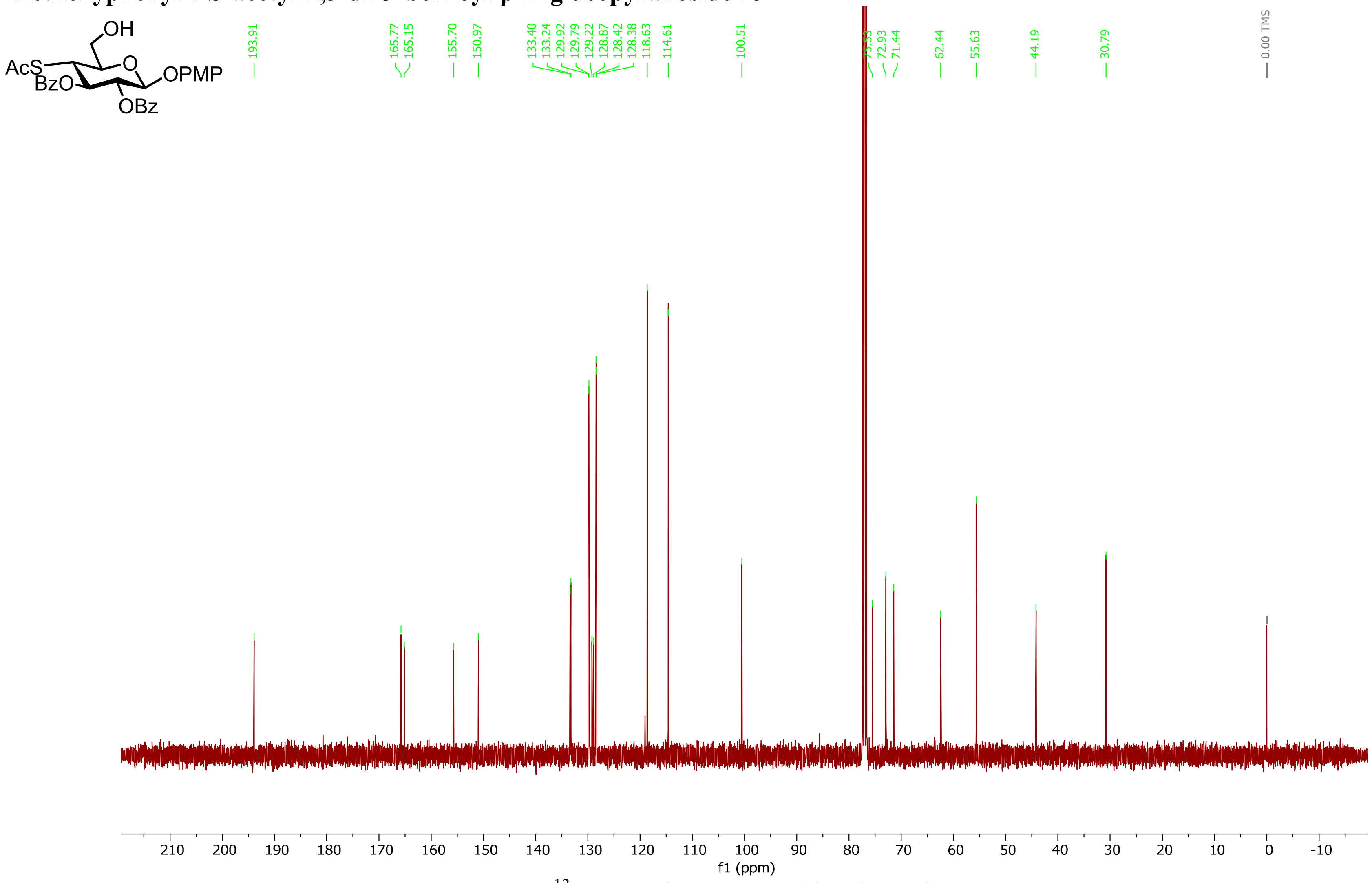

^{13}C NMR (100 MHz, Chloroform-*d*)

***p*-Methoxyphenyl 6-*O*-acetyl-2,3-di-*O*-benzoyl-4-thio-β-D-glucopyranoside S12**

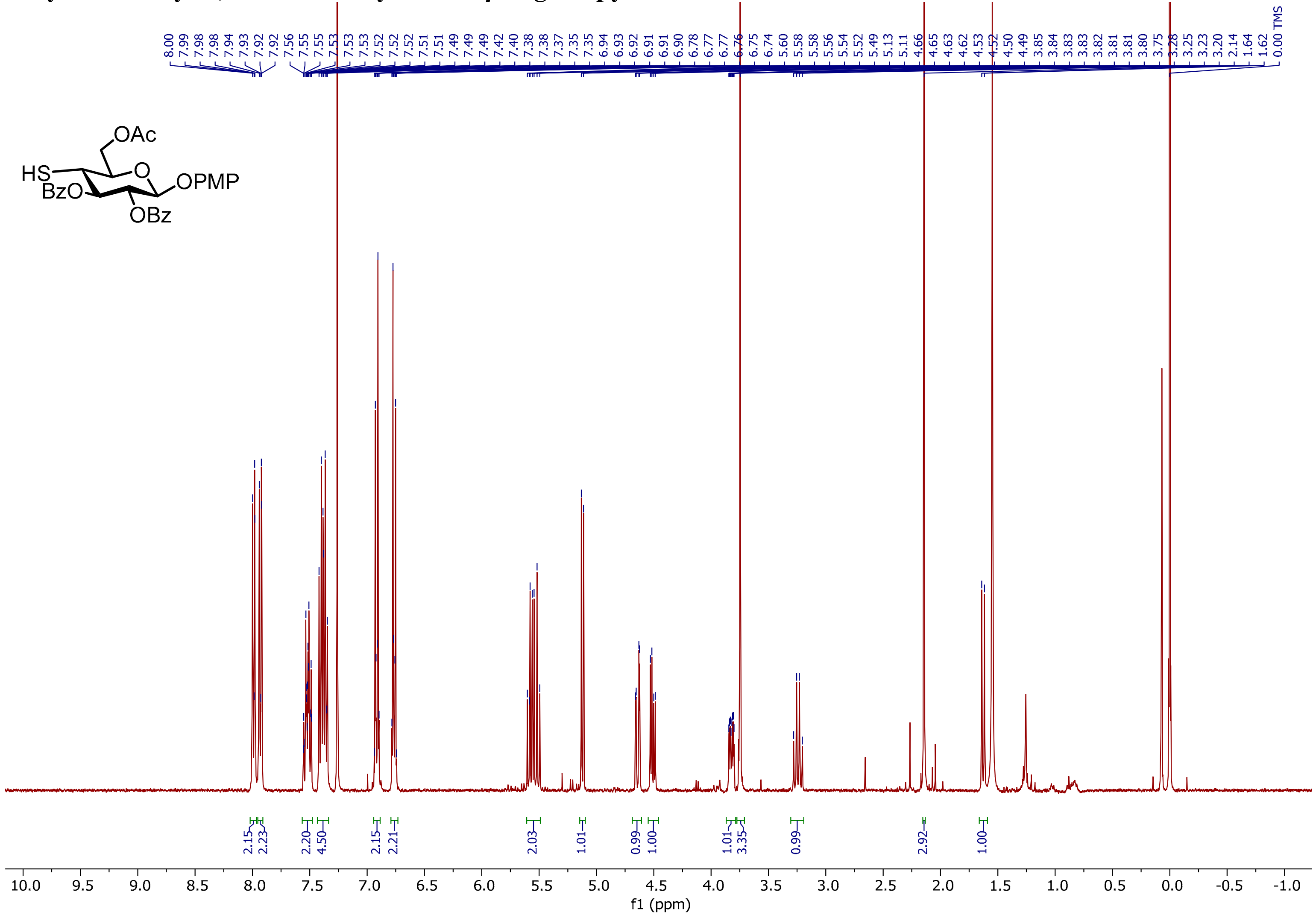

^{1}H NMR (400 MHz, Chloroform-*d*)

p-Methoxyphenyl 6-O-acetyl-2,3-di-O-benzoyl-4-thio-β-D-glucopyranoside S12

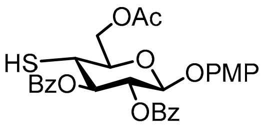

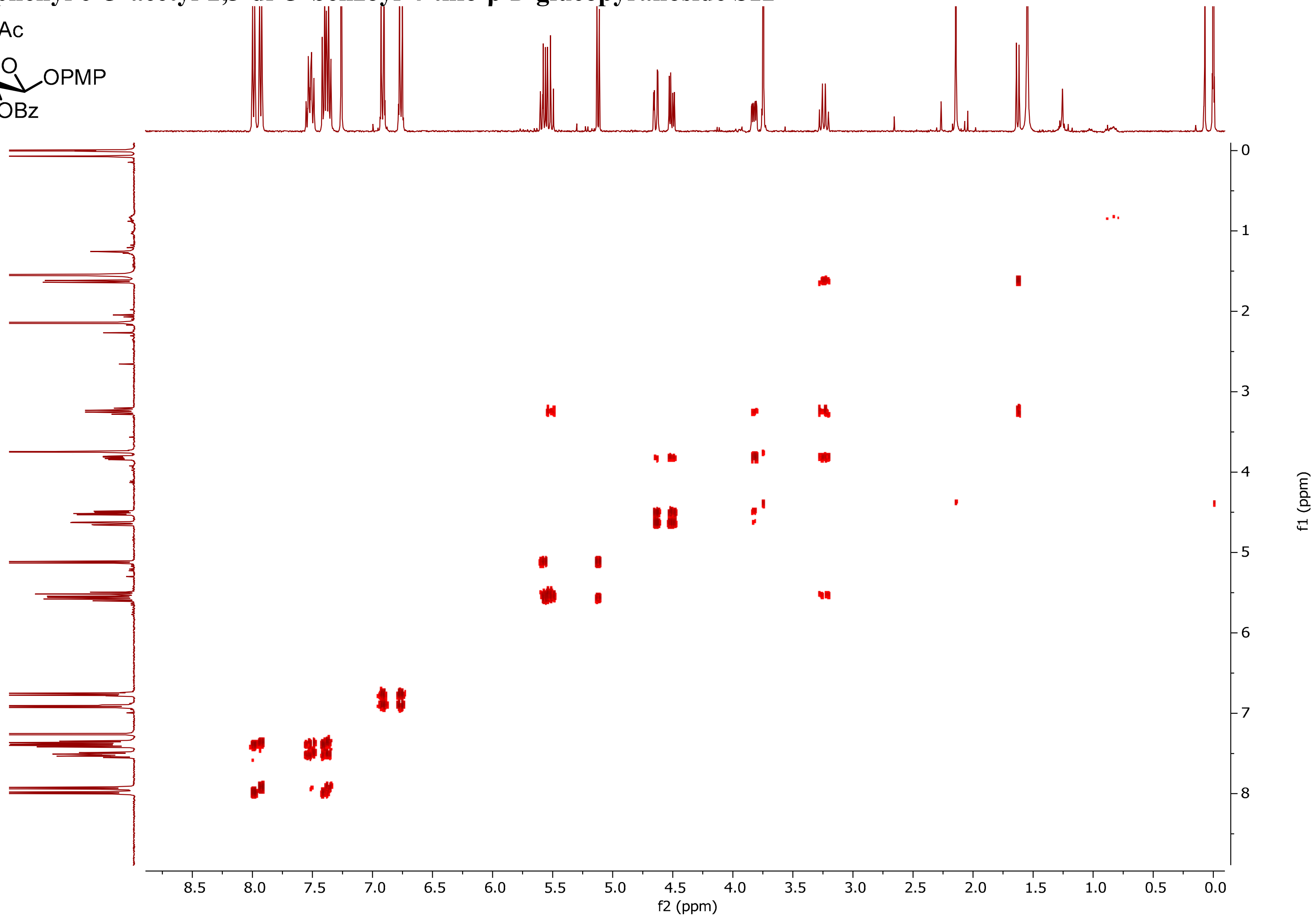

COSY NMR (400 MHz, Chloroform-*d*)

_p_-Methoxyphenyl 6-_O_-acetyl-2,3-di-_O_-benzoyl-4-thio-β-D-glucopyranoside S12

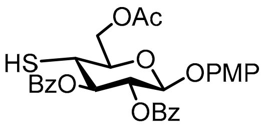

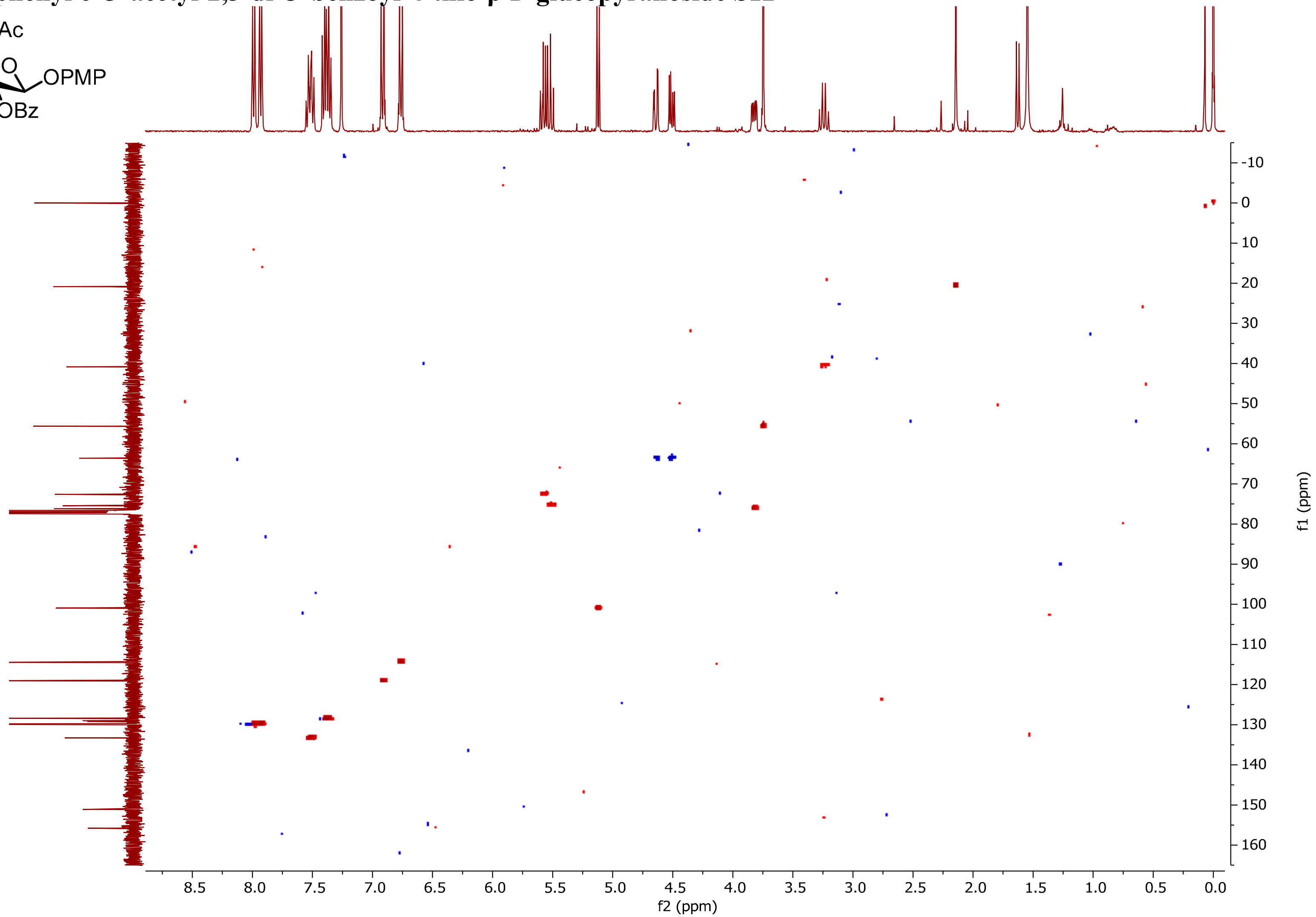

HSQC NMR (400 x 100 MHz, Chloroform-_d_)

p-Methoxyphenyl 6-*O*-acetyl-2,3-di-*O*-benzoyl-4-thio-β-D-glucopyranoside S12

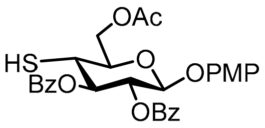

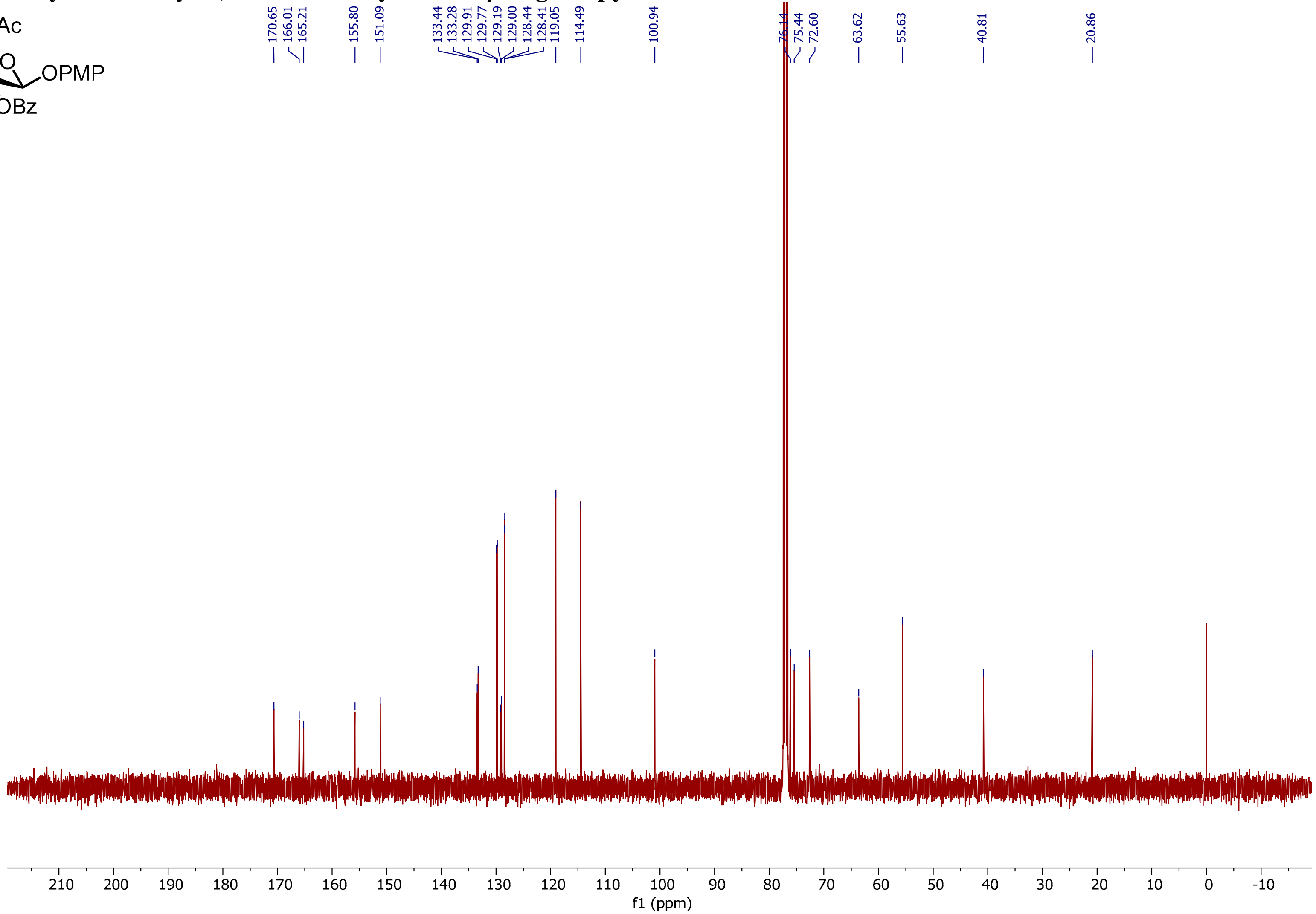

^{13}C NMR (100 MHz, Chloroform-*d*)

Methyl (*p*-methoxyphenyl 4-*S*-acetyl-2,3-di-*O*-benzoyl-β-D-glucopyranosid)uronate 14

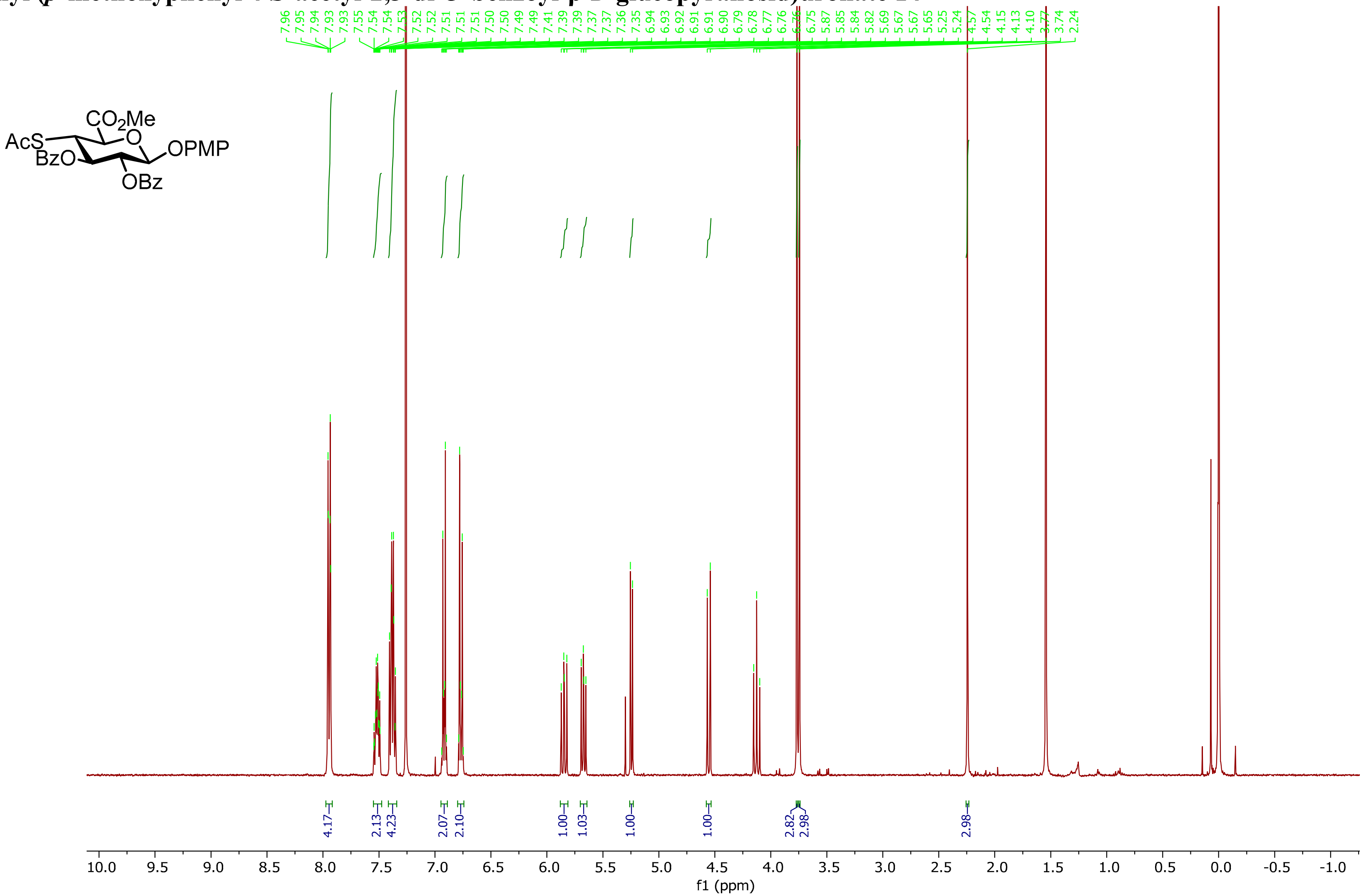

^{1}H NMR (400 MHz, Chloroform-*d*)

Methyl (*p*-methoxyphenyl 4-*S*-acetyl-2,3-di-*O*-benzoyl-β-D-glucopyranosid)uronate 14

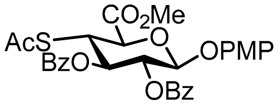

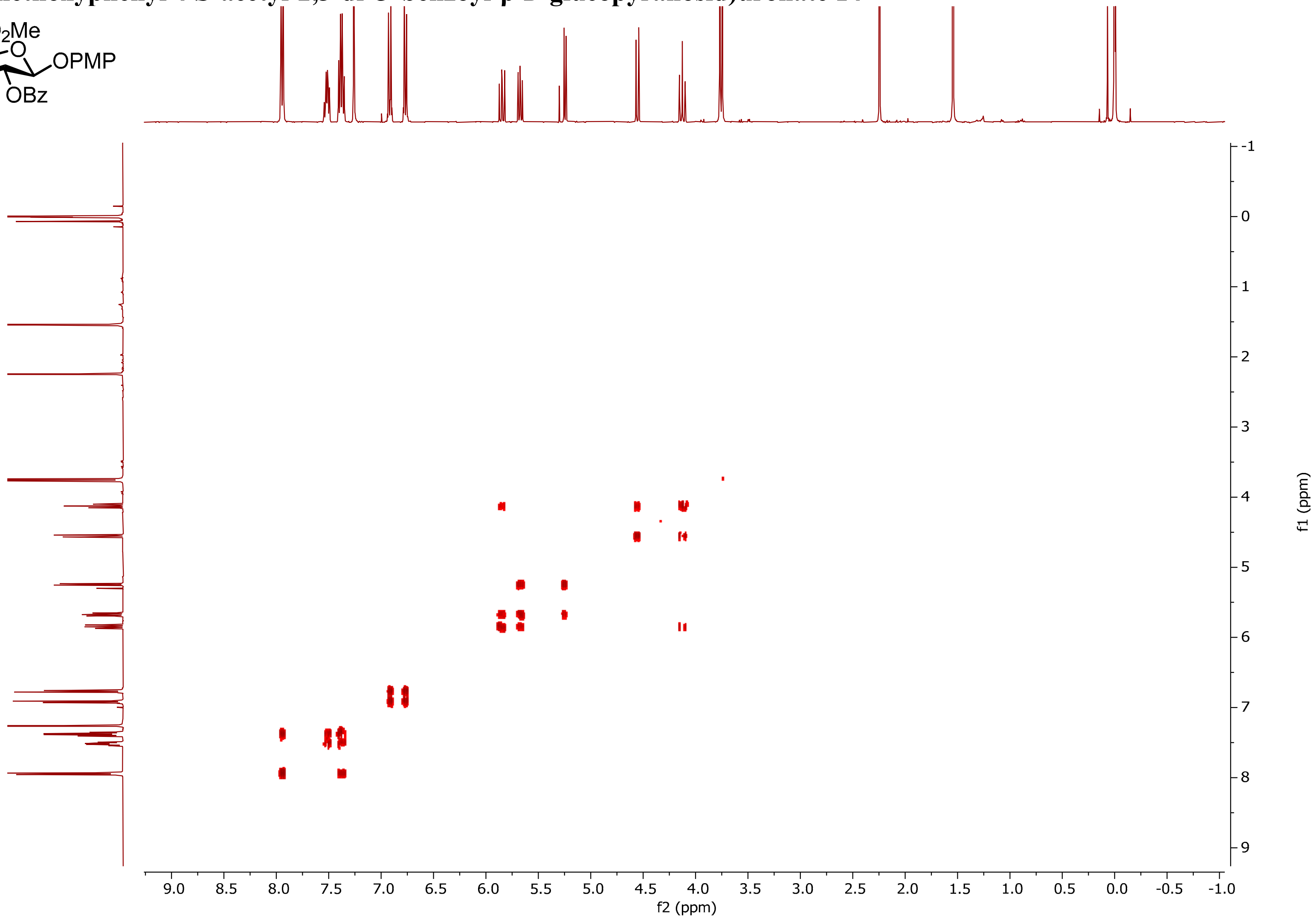

COSY NMR (400 MHz, Chloroform-*d*)

Methyl (*p*-methoxyphenyl 4-*S*-acetyl-2,3-di-*O*-benzoyl-β-D-glucopyranosid)uronate 14

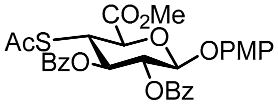

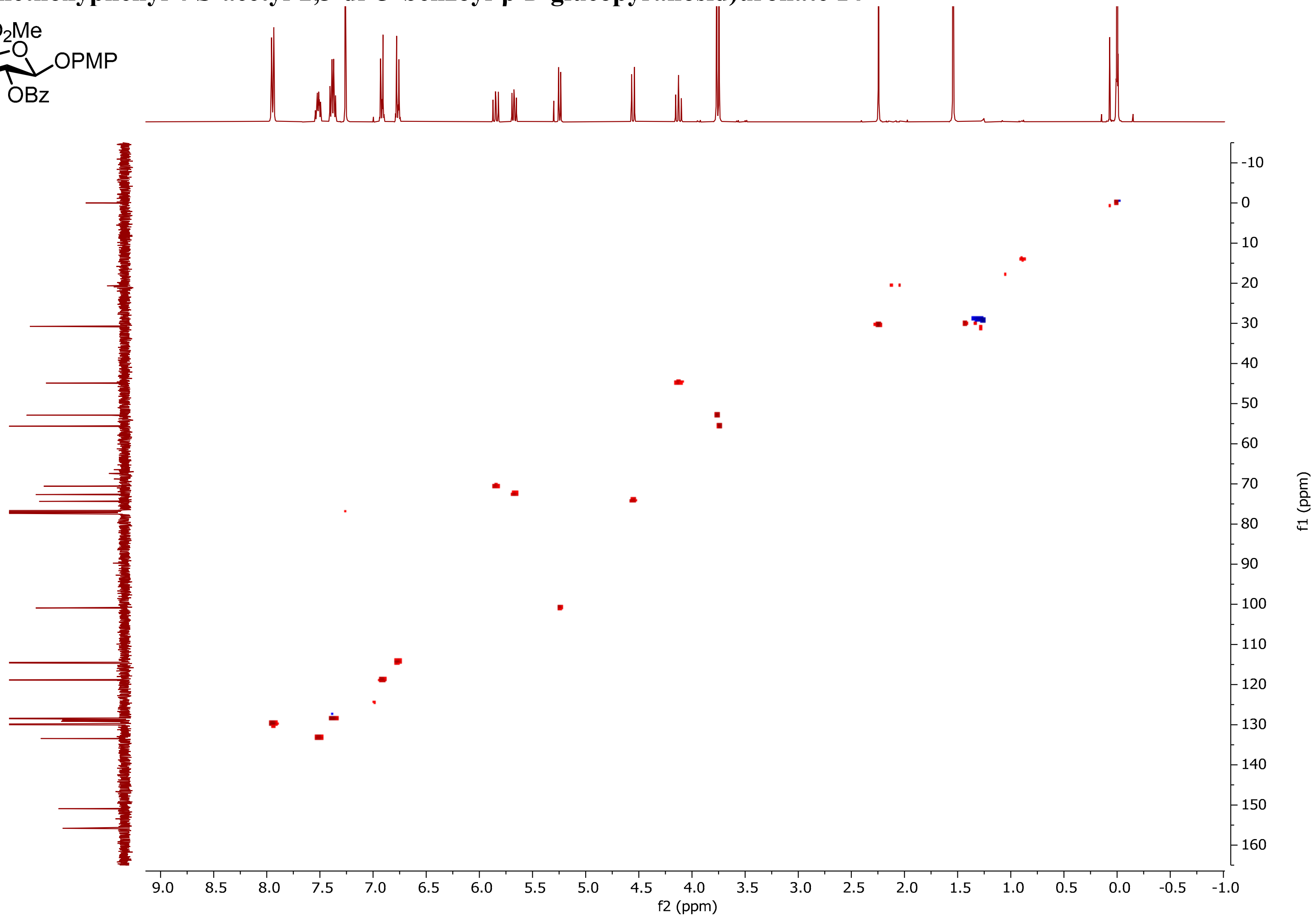

HSQC NMR (400 x 100 MHz, Chloroform-*d*)

Methyl (*p*-methoxyphenyl 4-*S*-acetyl-2,3-di-*O*-benzoyl-β-D-glucopyranosid)uronate 14

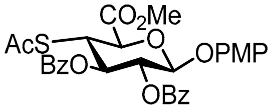

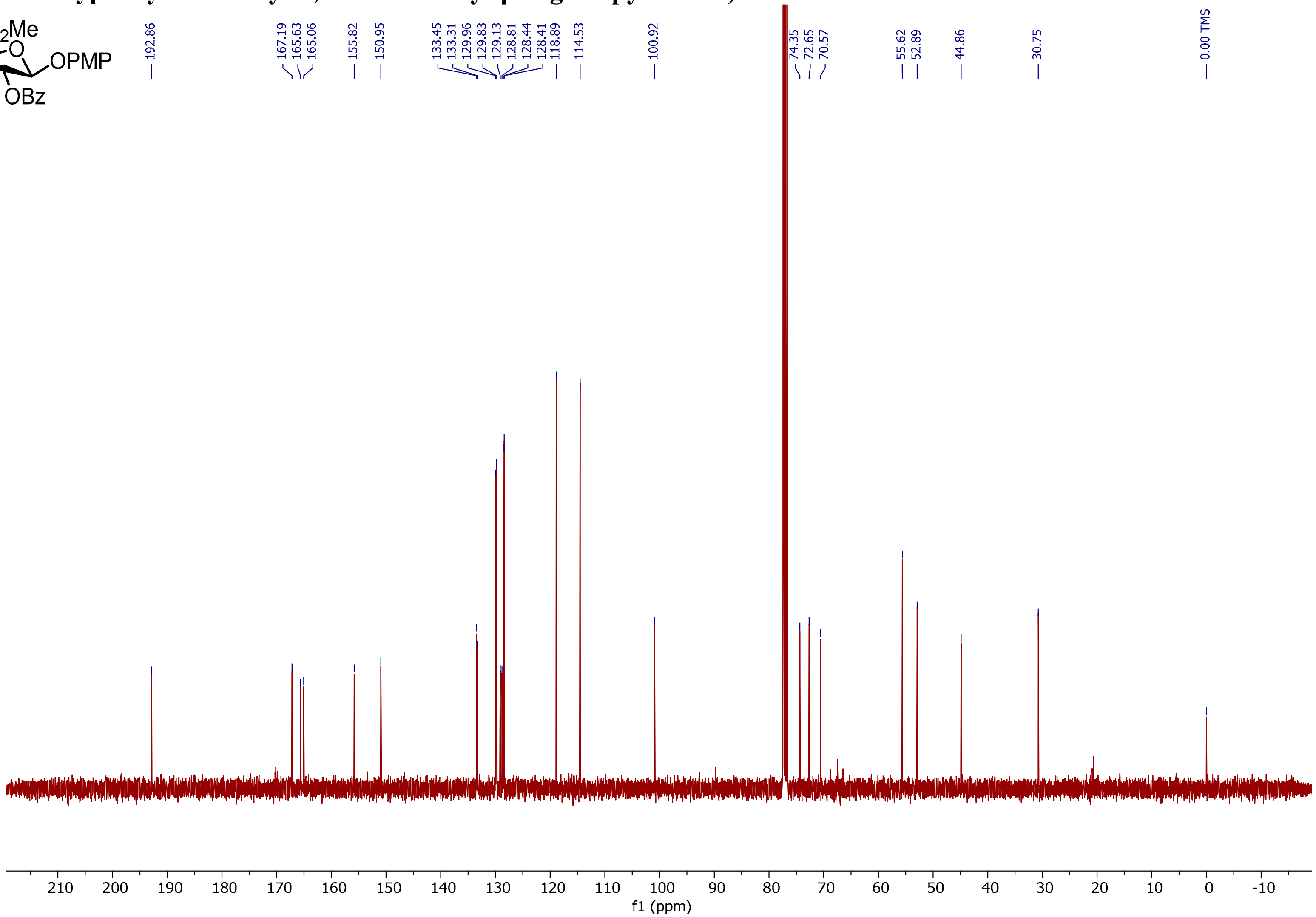

^{13}C NMR (100 MHz, Chloroform-*d*)

Methyl (*p*-methoxyphenyl 2,3-di-*O*-benzoyl-4-thio-β-D-glucopyranosid)uronate 15

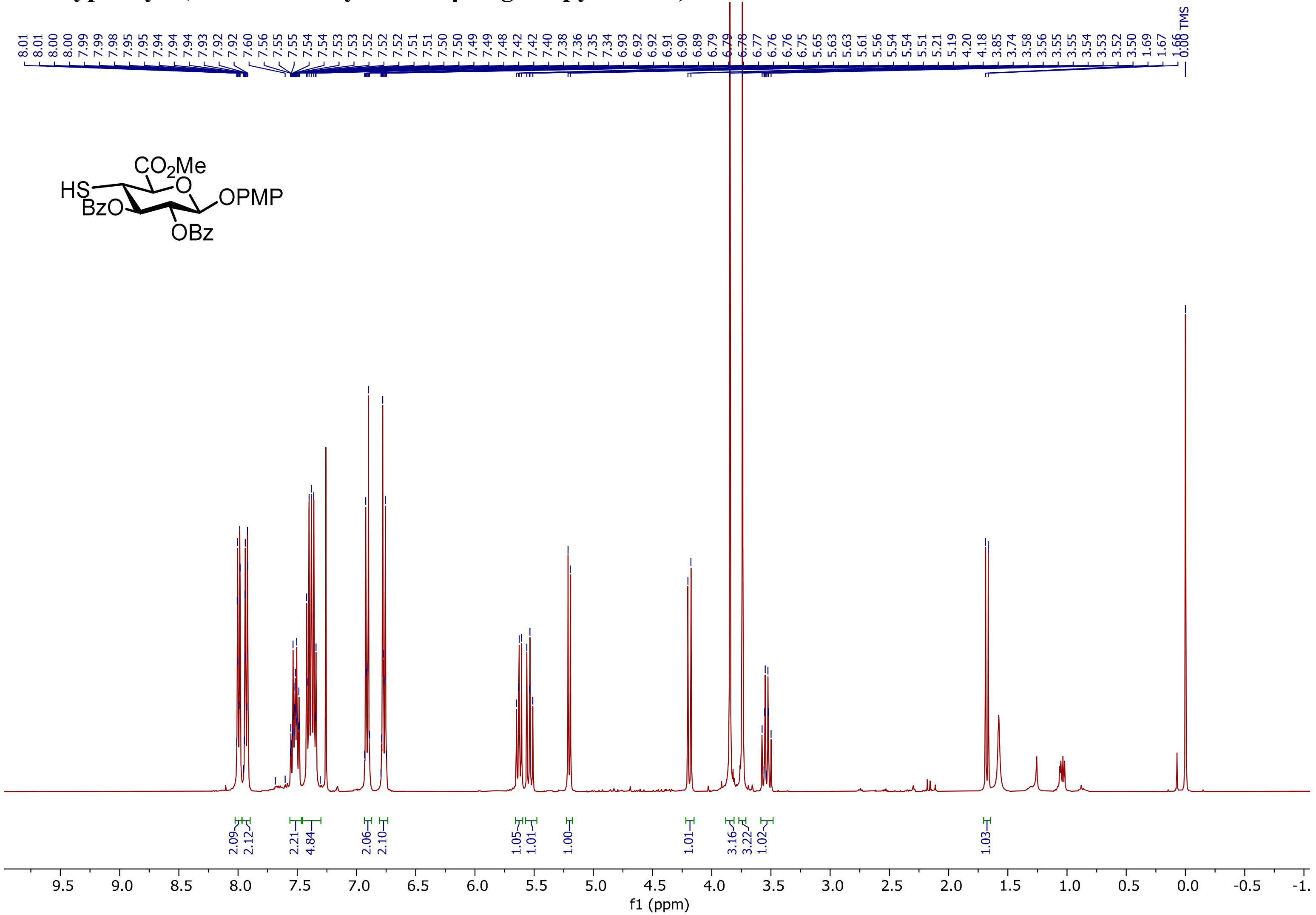

^{1}H NMR (400 MHz, Chloroform-*d*)

Methyl (*p*-methoxyphenyl 2,3-di-*O*-benzoyl-4-thio-β-D-glucopyranosid)uronate 15

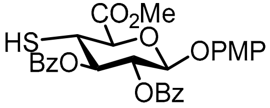

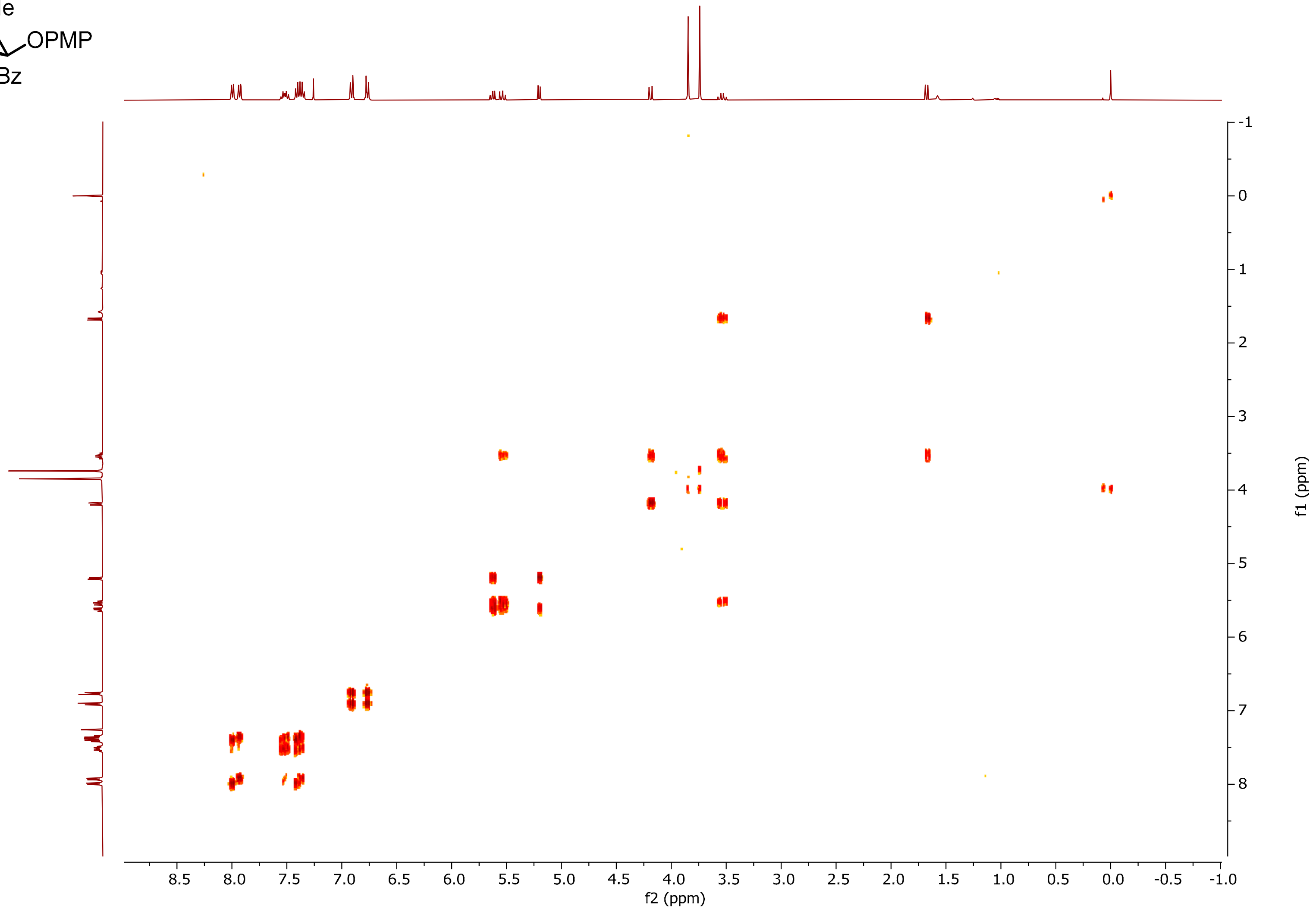

COSY NMR (400 MHz, Chloroform-*d*)

Methyl (*p*-methoxyphenyl 2,3-di-*O*-benzoyl-4-thio-β-D-glucopyranosid)uronate 15

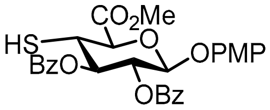

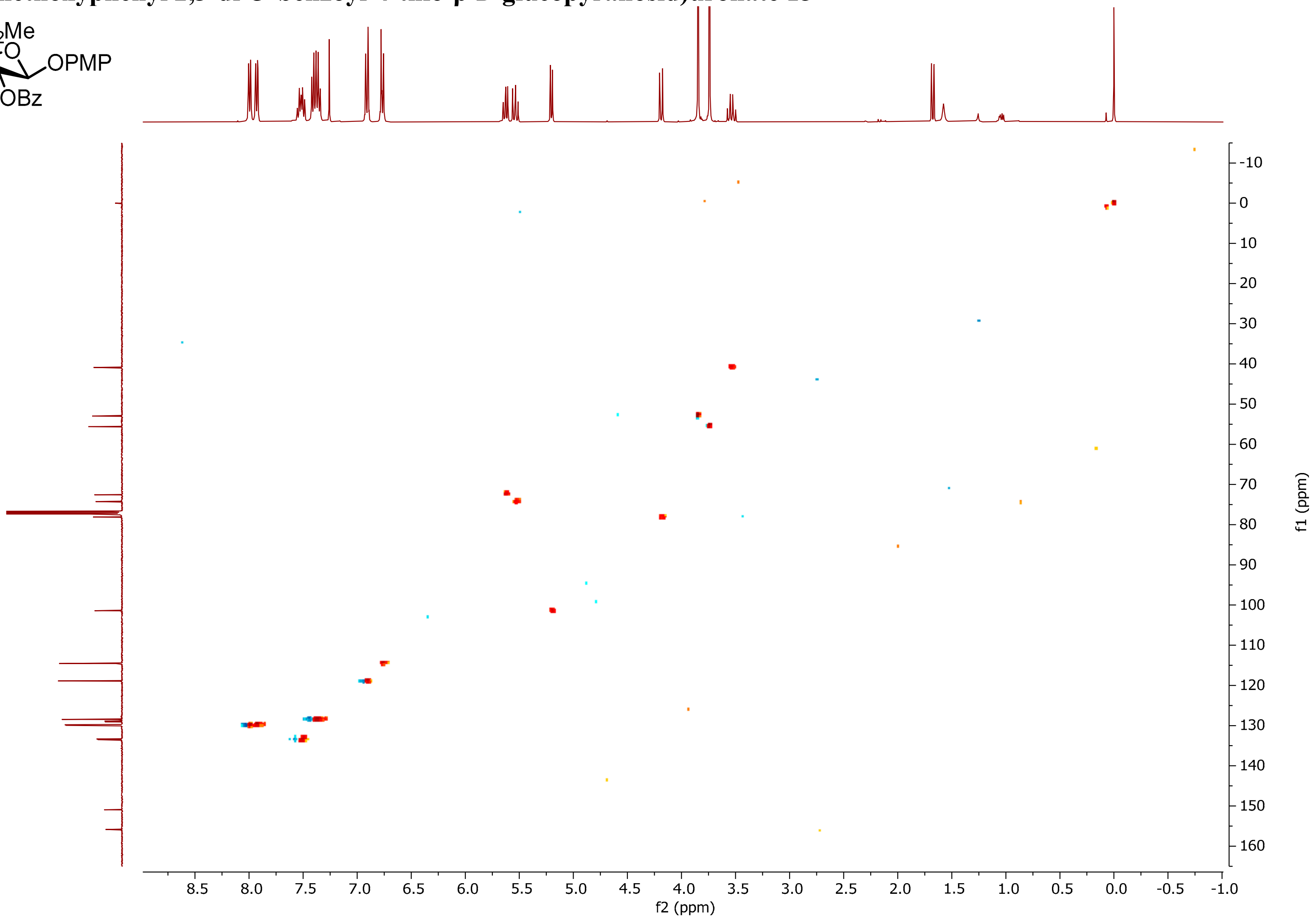

HSQC NMR (400 x 100 MHz, Chloroform-*d*)

Methyl (*p*-methoxyphenyl 2,3-di-*O*-benzoyl-4-thio-β-D-glucopyranosid)uronate 15

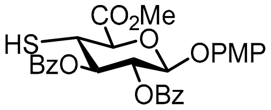

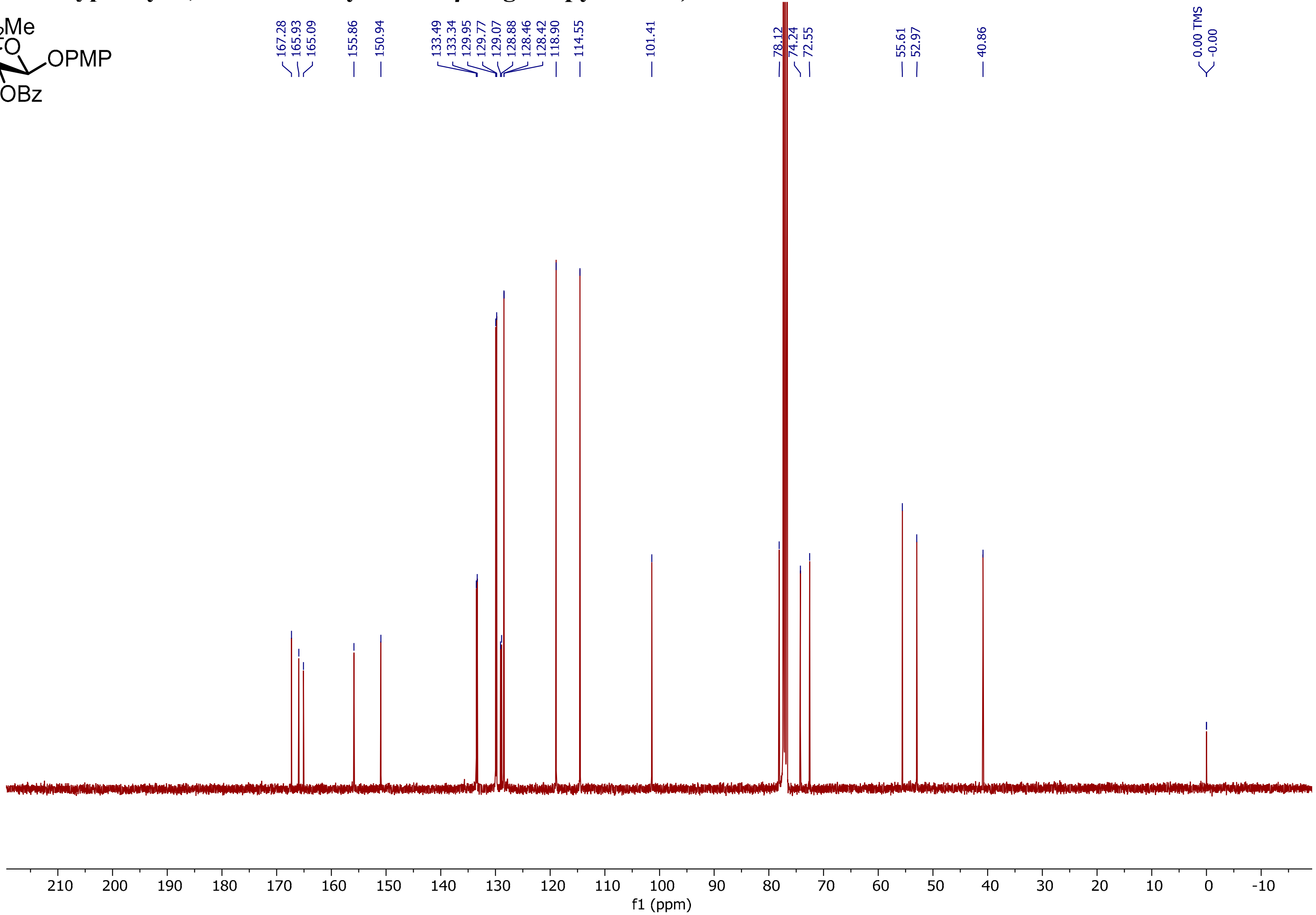

^{13}C NMR (100 MHz, Chloroform-*d*)

p-Methoxyphenyl 3-O-benzyl-D-galactopyranoside S6

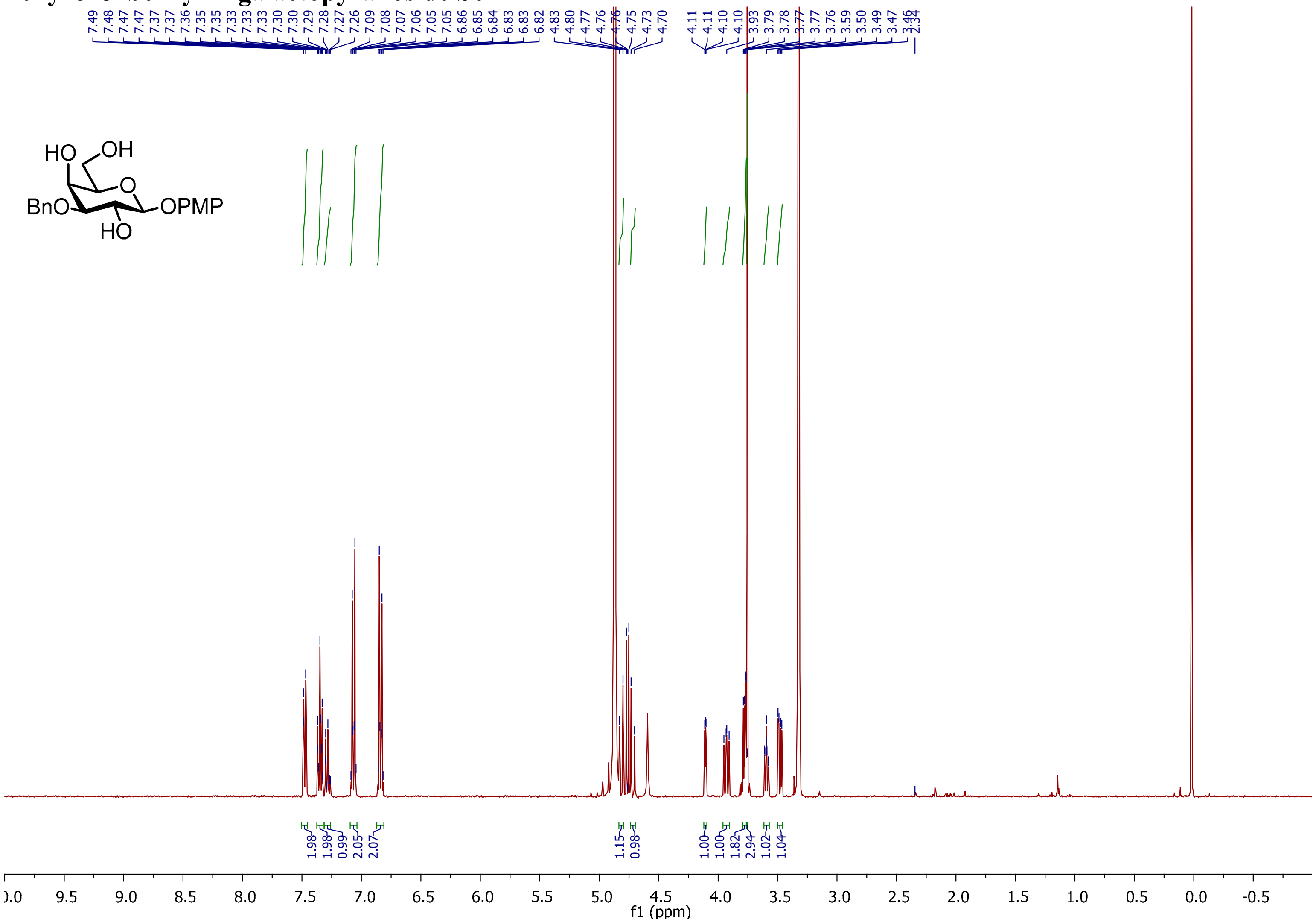

^{1}H NMR (400 MHz, Methanol-*d*)

p-Methoxyphenyl 3-O-benzyl-D-galactopyranoside S6

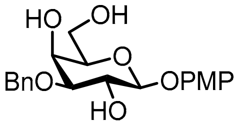

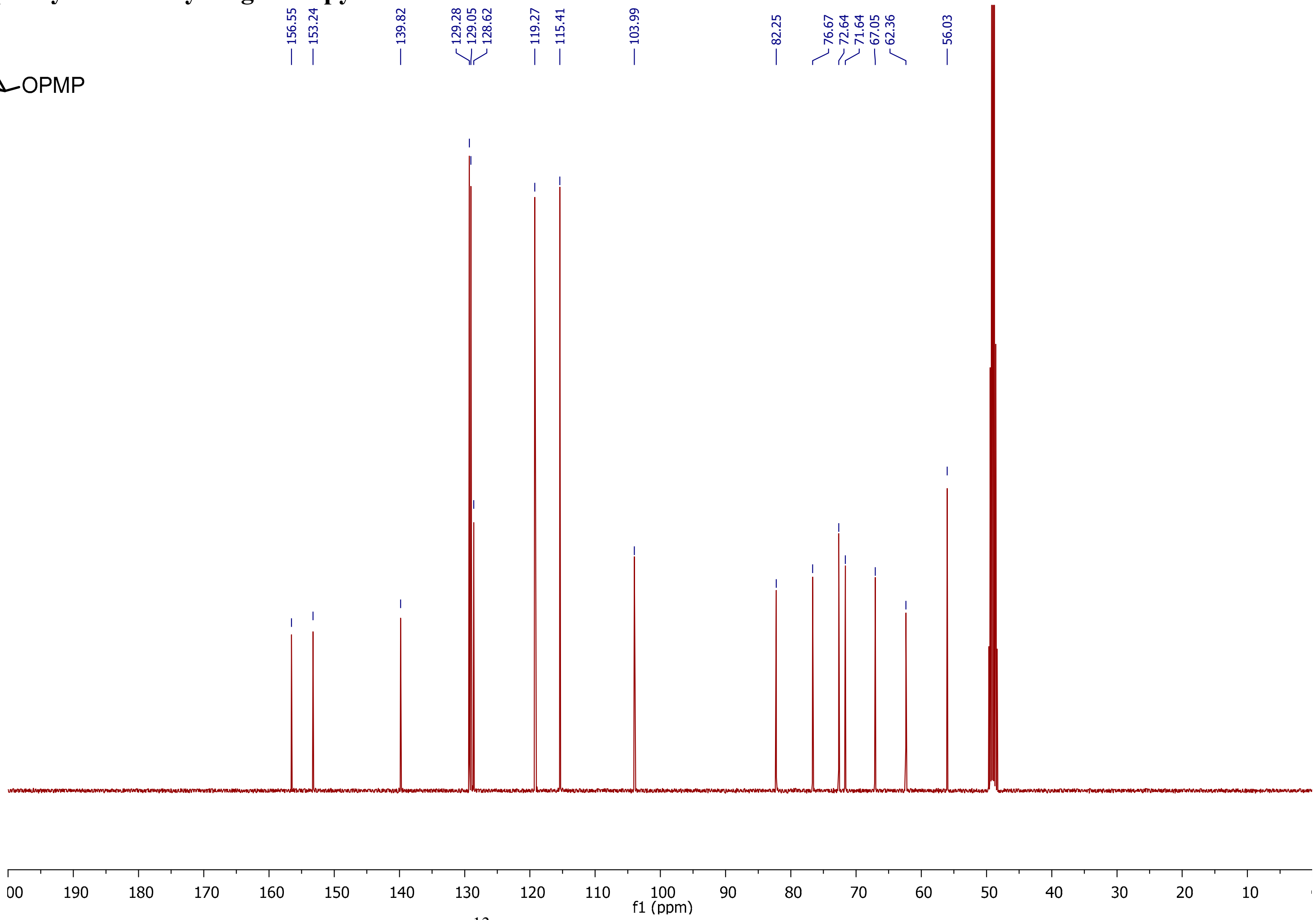

^{13}C NMR (100, MHz Methanol-*d*)

_p_-Methoxylphenyl 2-_O_-benzoyl-3-_O_-benzyl-4,6-_O_-benzylidene-β-D-galactopyranoside S7

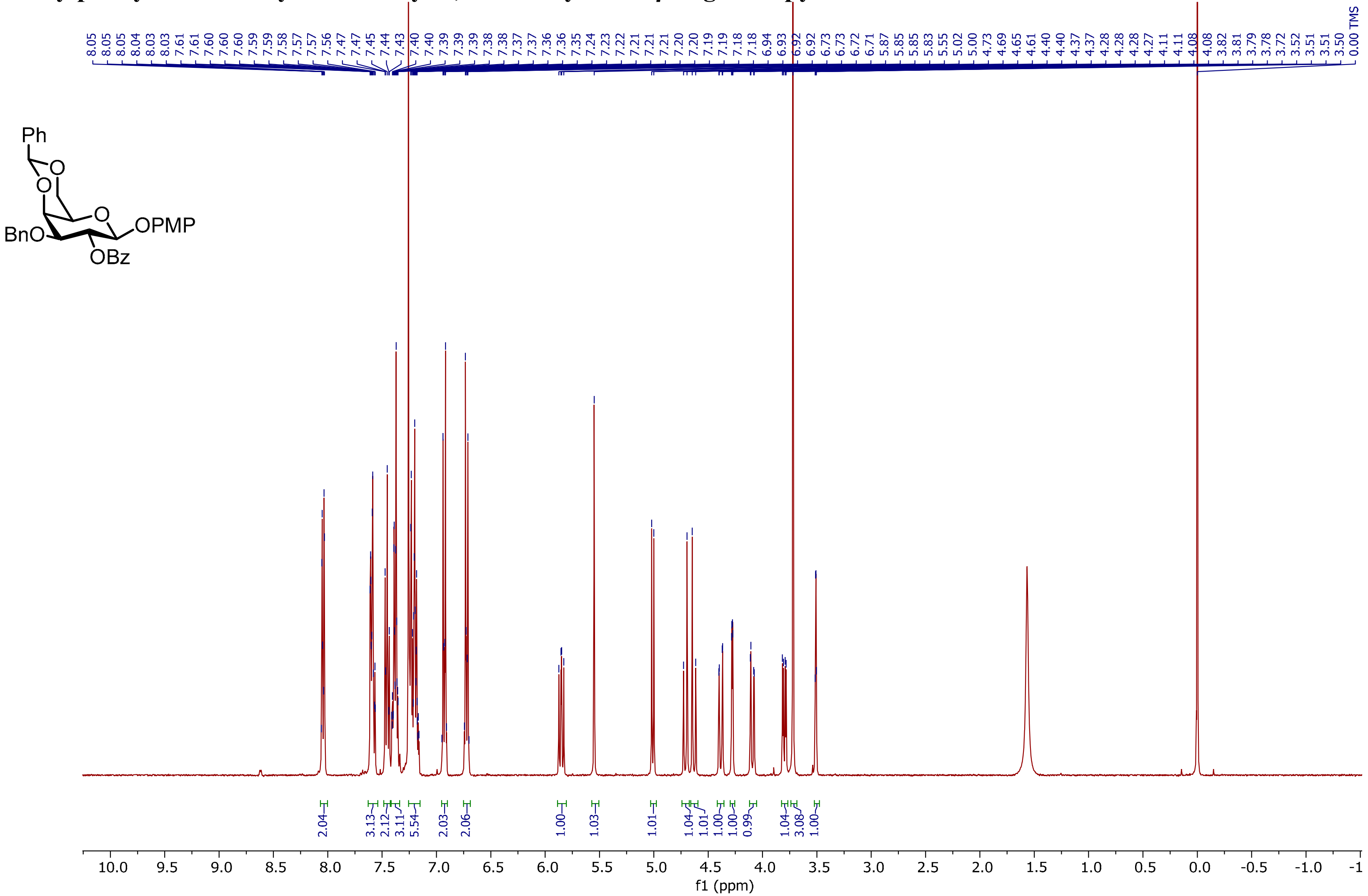

^{1}H NMR (400 MHz, Chloroform-_d_)

_p_-Methoxylphenyl 2-_O_-benzoyl-3-_O_-benzyl-4,6-_O_-benzylidene-β-D-galactopyranoside S7

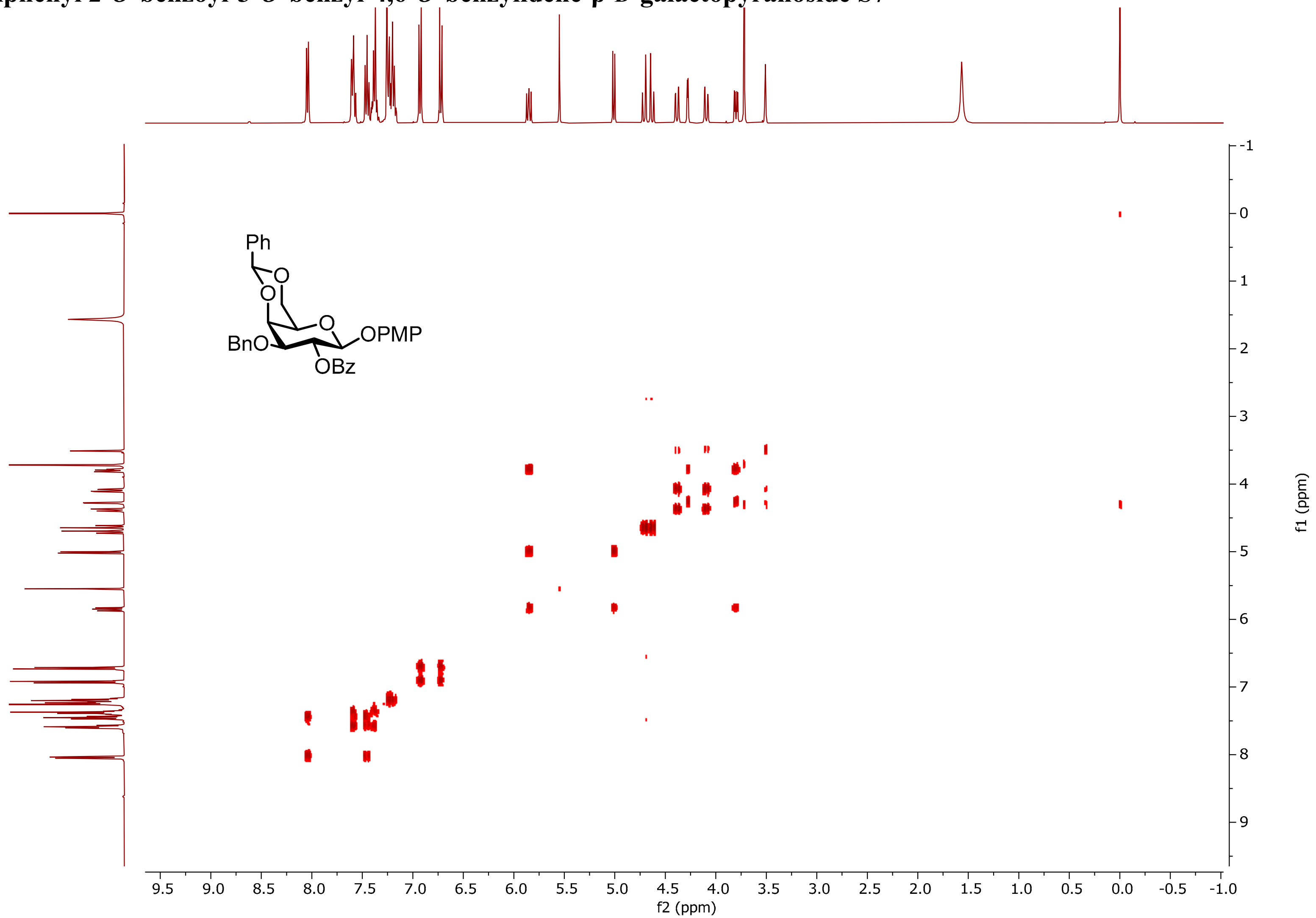

COSY NMR (400 MHz, Chloroform-_d_)

_p_-Methoxylphenyl 2-_O_-benzoyl-3-_O_-benzyl-4,6-_O_-benzylidene-β-D-galactopyranoside S7

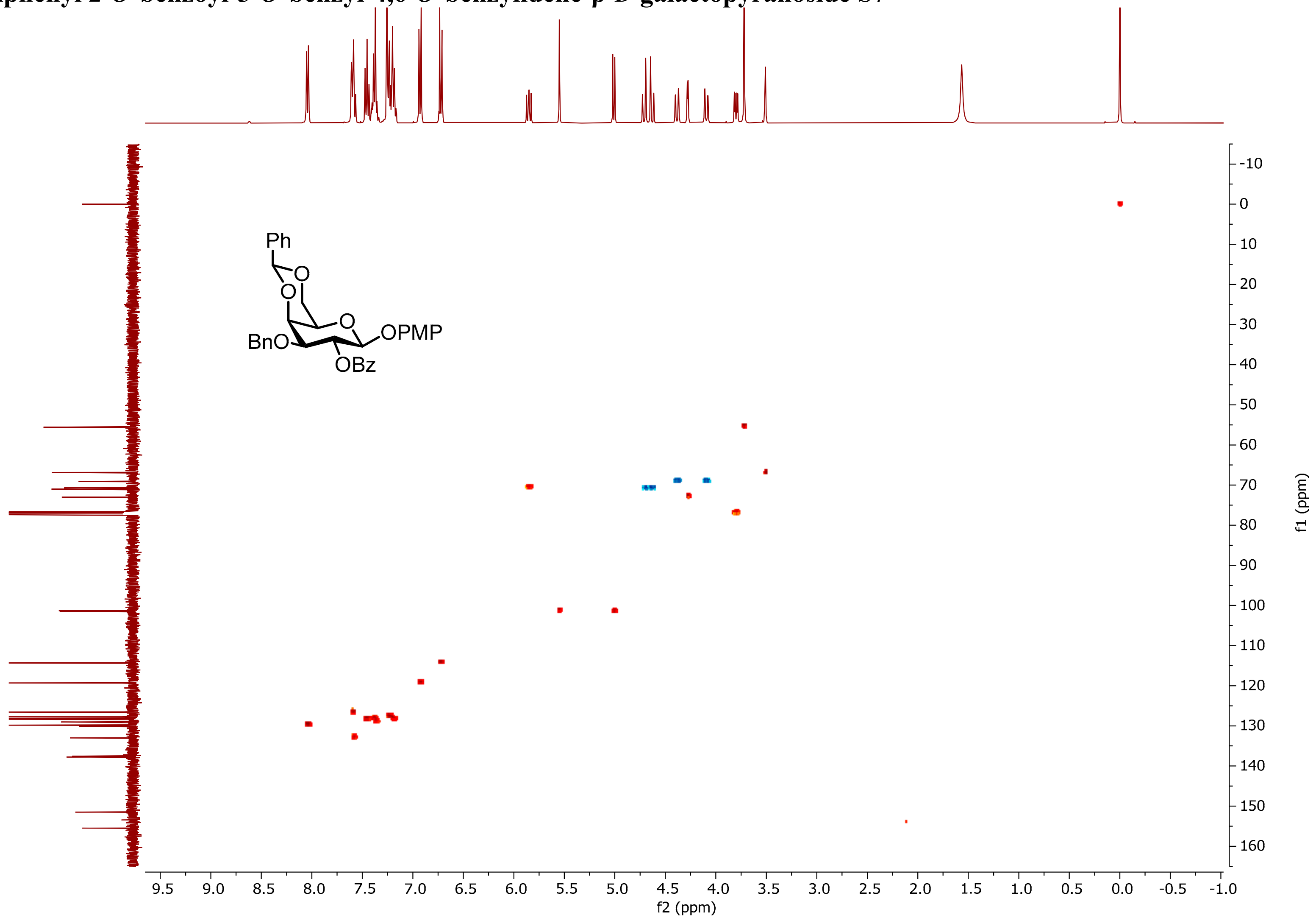

HSQC NMR (400 x 100 MHz, Chloroform-_d_)

_p_-Methoxylphenyl 2-_O_-benzoyl-3-_O_-benzyl-4,6-_O_-benzylidene-β-D-galactopyranoside S7

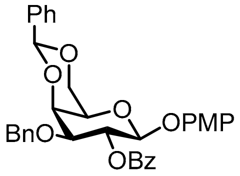

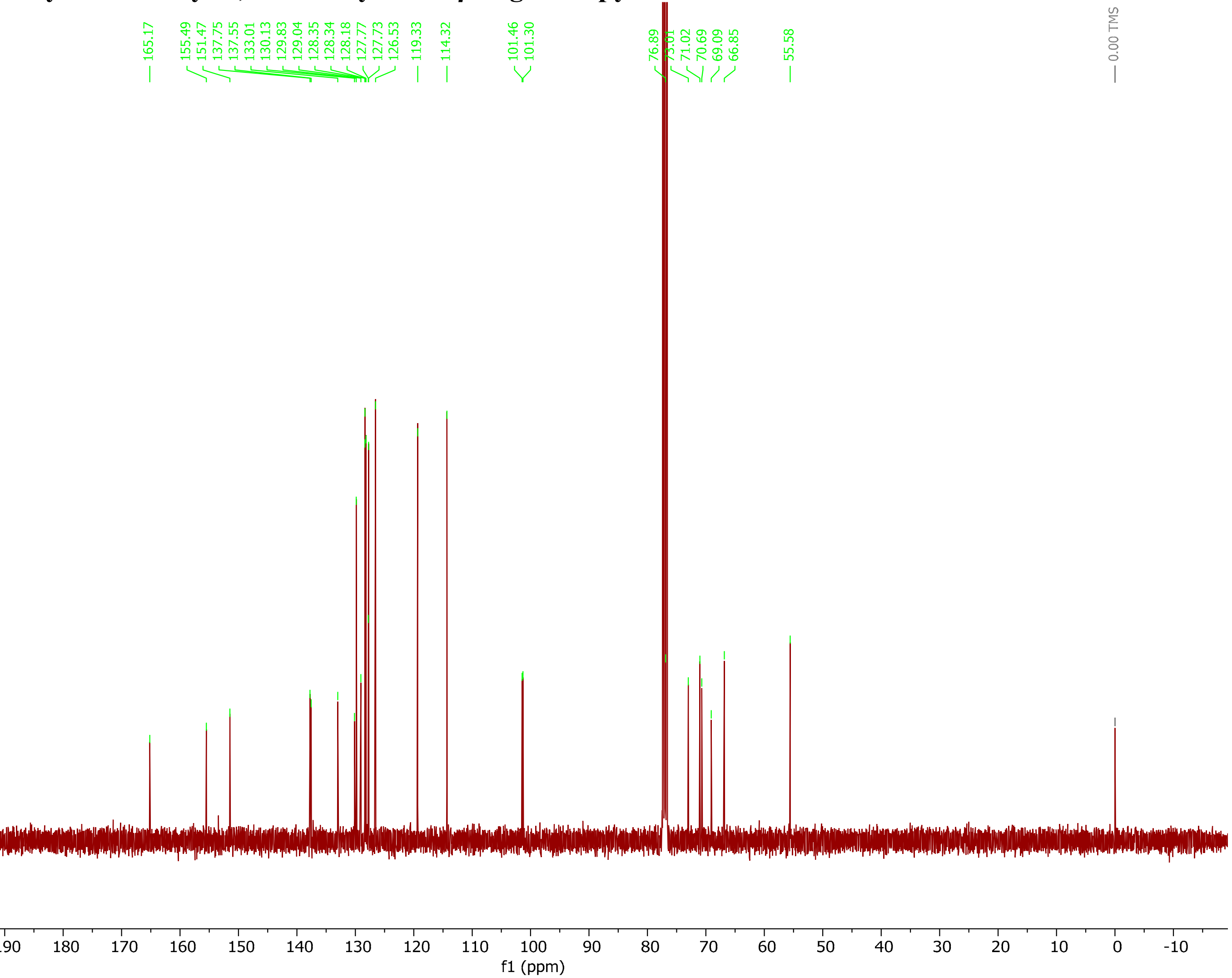

^{13}C NMR (100 MHz, Chloroform-*d*)

p-Methoxyphenyl 2-O-benzoyl-3-O-benzyl-6-O-β-D-galactopyranoside 2

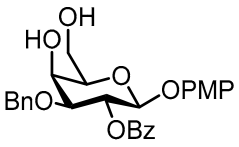

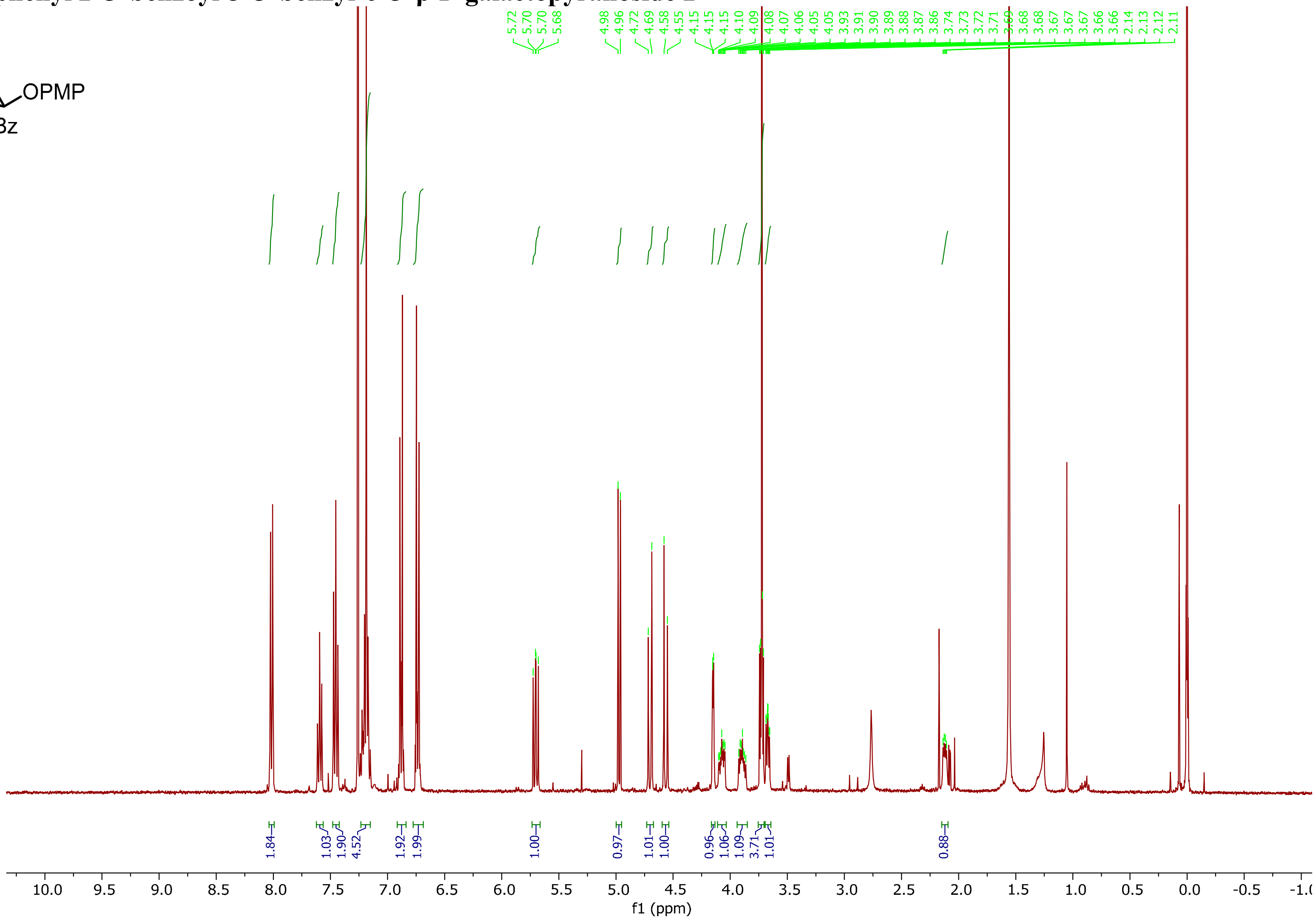

^{1}H NMR (400 MHz, Chloroform-*d*)

p-Methoxyphenyl 2-O-benzoyl-3-O-benzyl-6-O-β-D-galactopyranoside 2

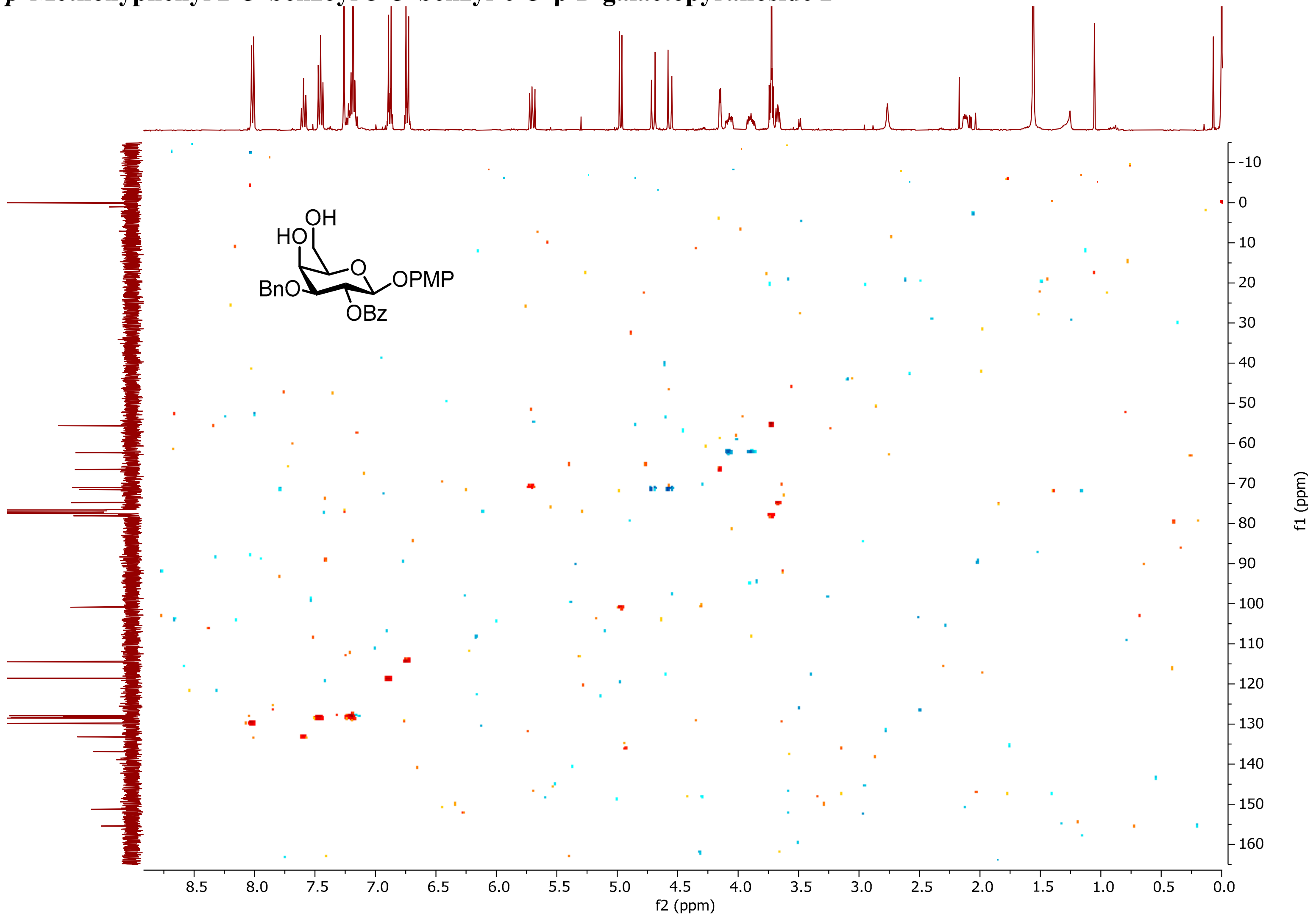

HSQC NMR (400 x 100 MHz, Chloroform-*d*)

p-Methoxyphenyl 2-O-benzoyl-3-O-benzyl-6-O-β-D-galactopyranoside 2

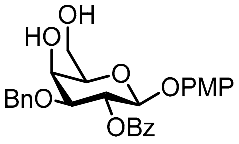

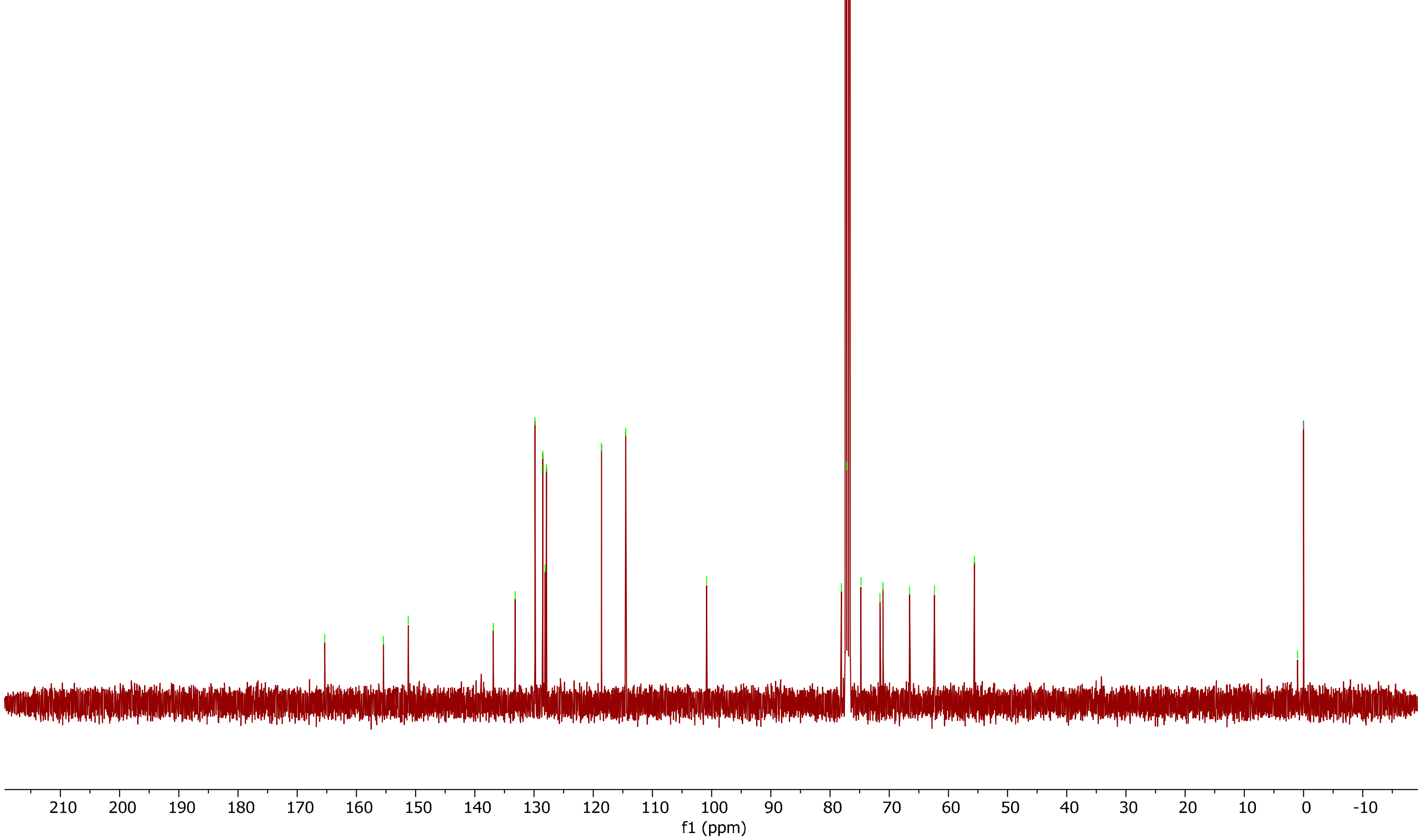

^{13}C NMR (100 MHz, Chloroform-*d*)

***p*-Methoxyphenyl 2-*O*-benzoyl-3-*O*-benzyl-6-*O*-(triisopropylsilyl)-β-D-galactopyranoside 6**

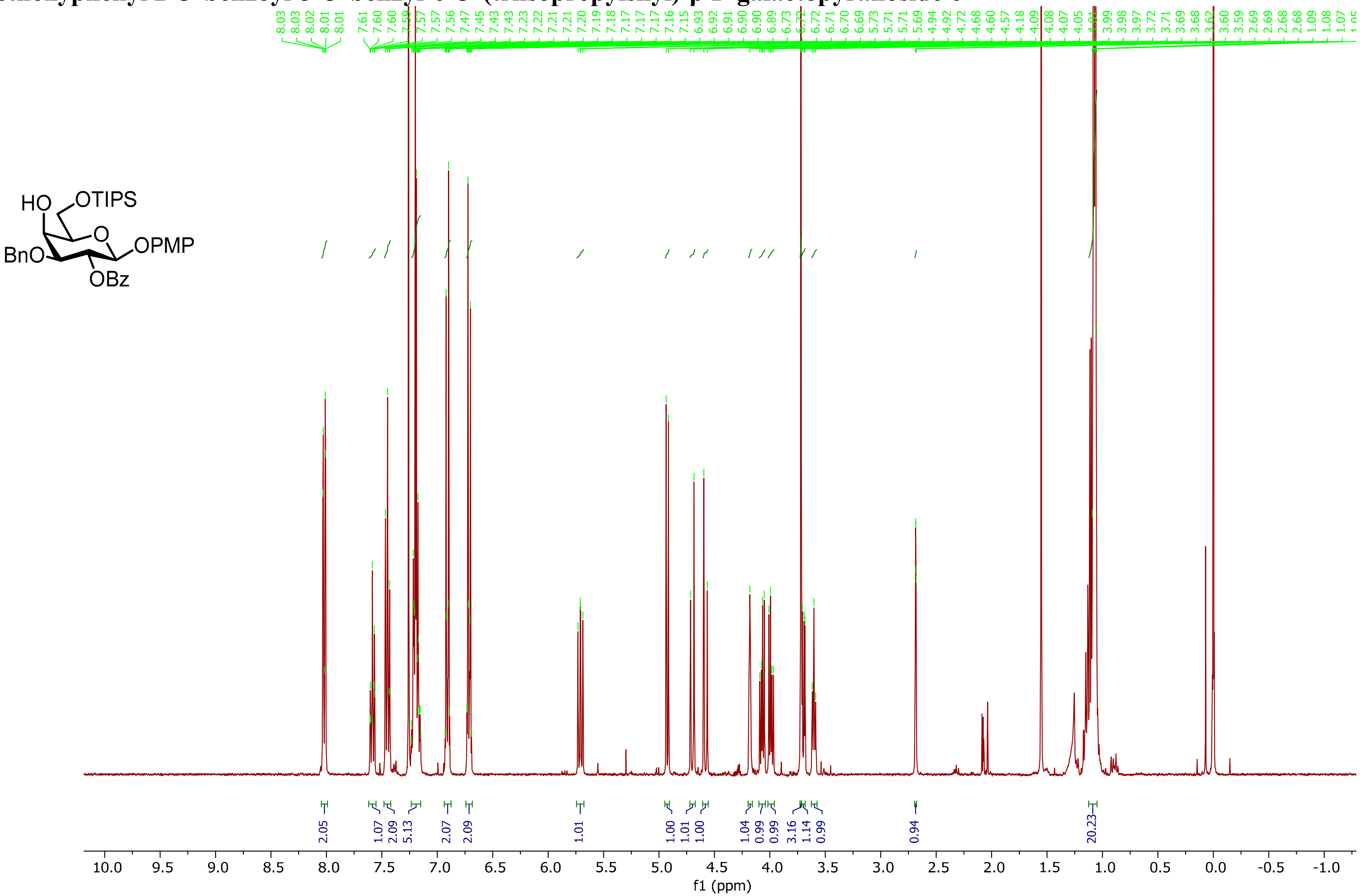

^{1}H NMR (400 MHz, Chloroform-*d*)

_p_-Methoxyphenyl 2-_O_-benzoyl-3-_O_-benzyl-6-_O_-(triisopropylsilyl)-β-D-galactopyranoside 6

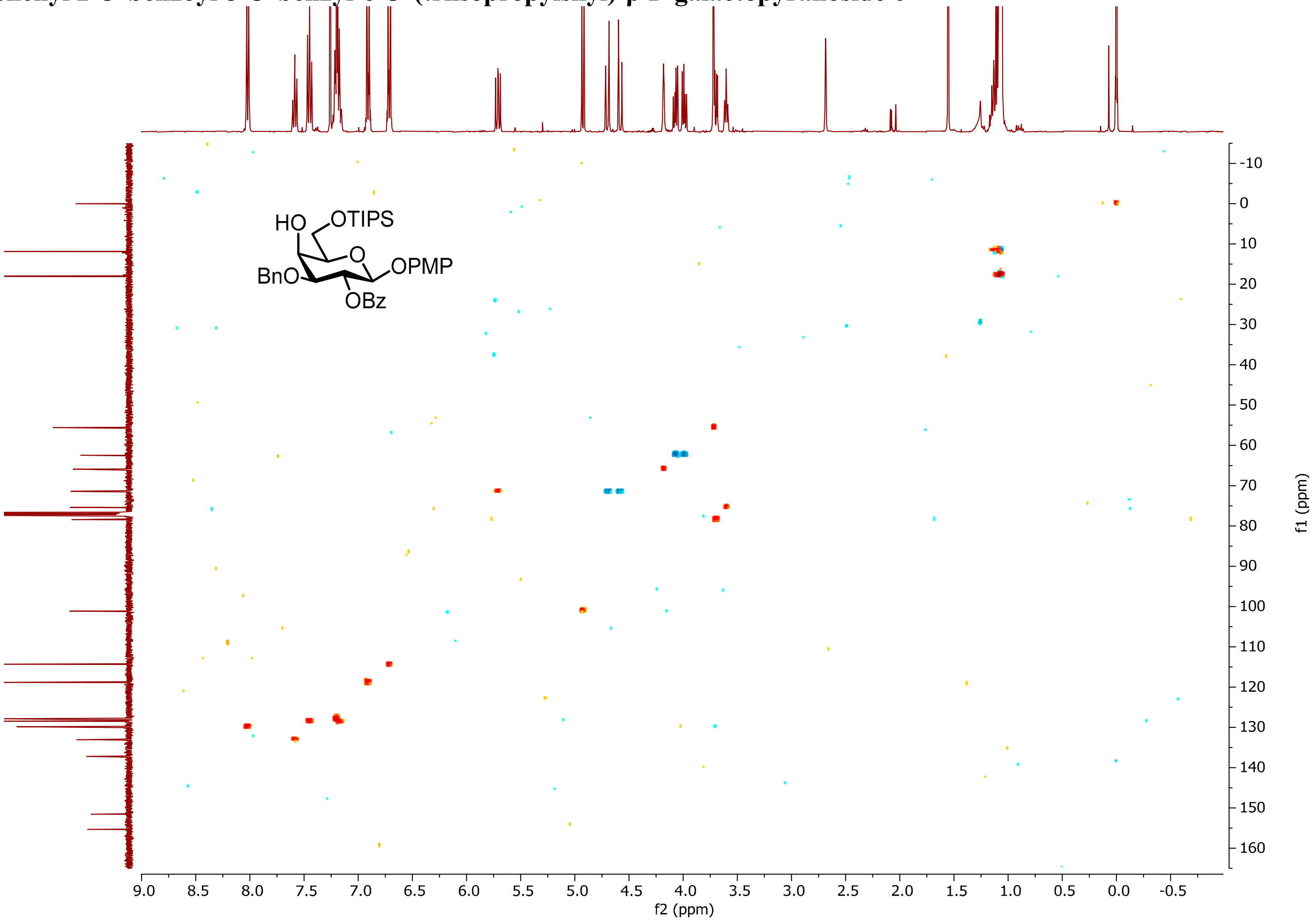

HSQC NMR (400 x 100 MHz, Chloroform-_d_)

_p_-Methoxyphenyl 2-_O_-benzoyl-3-_O_-benzyl-6-_O_-(triisopropylsilyl)-β-D-galactopyranoside 6

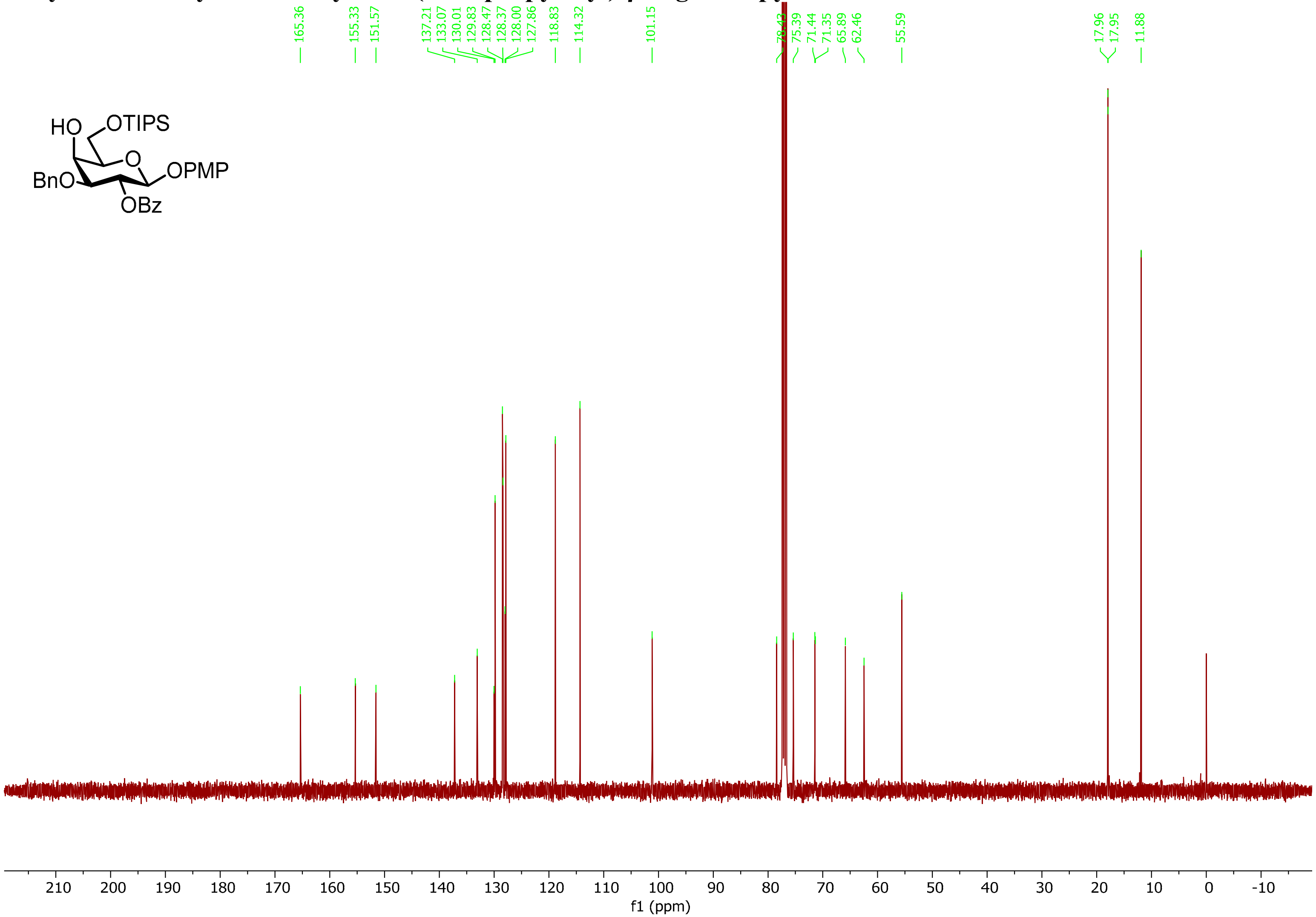

^{13}C NMR (100 MHz, Chloroform-_d_)

***p*-Methoxyphenyl 4-*S*-acetyl-2-*O*-benzoyl-3-*O*-benzyl-6-*O*-(triisopropylsilyl)- β-D-glucopyranoside 10**

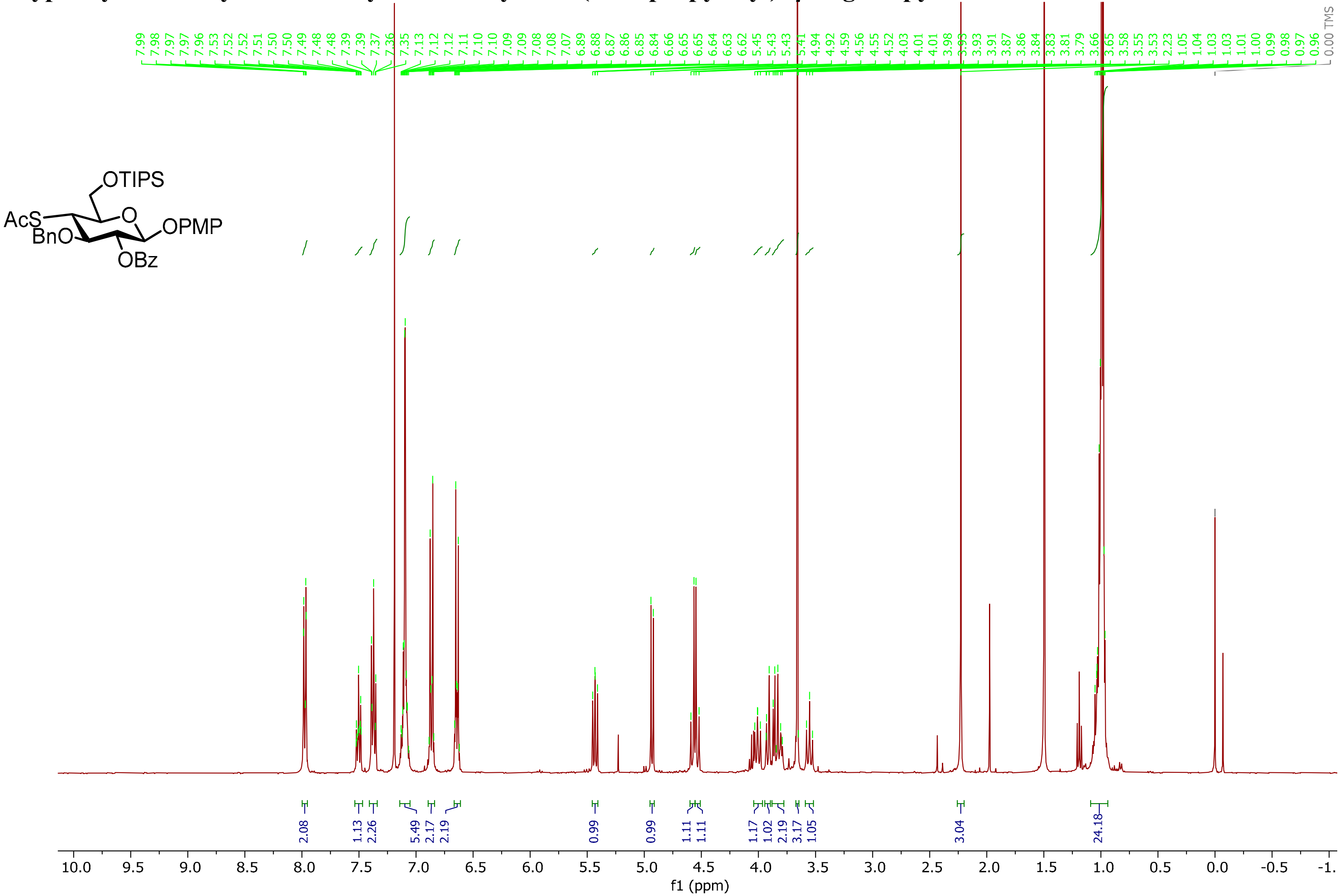

^{1}H NMR (400 MHz, Chloroform-*d*)

p-Methoxyphenyl 4-*S*-acetyl-2-*O*-benzoyl-3-*O*-benzyl-6-*O*-(triisopropylsilyl)- β-D-glucopyranoside 10

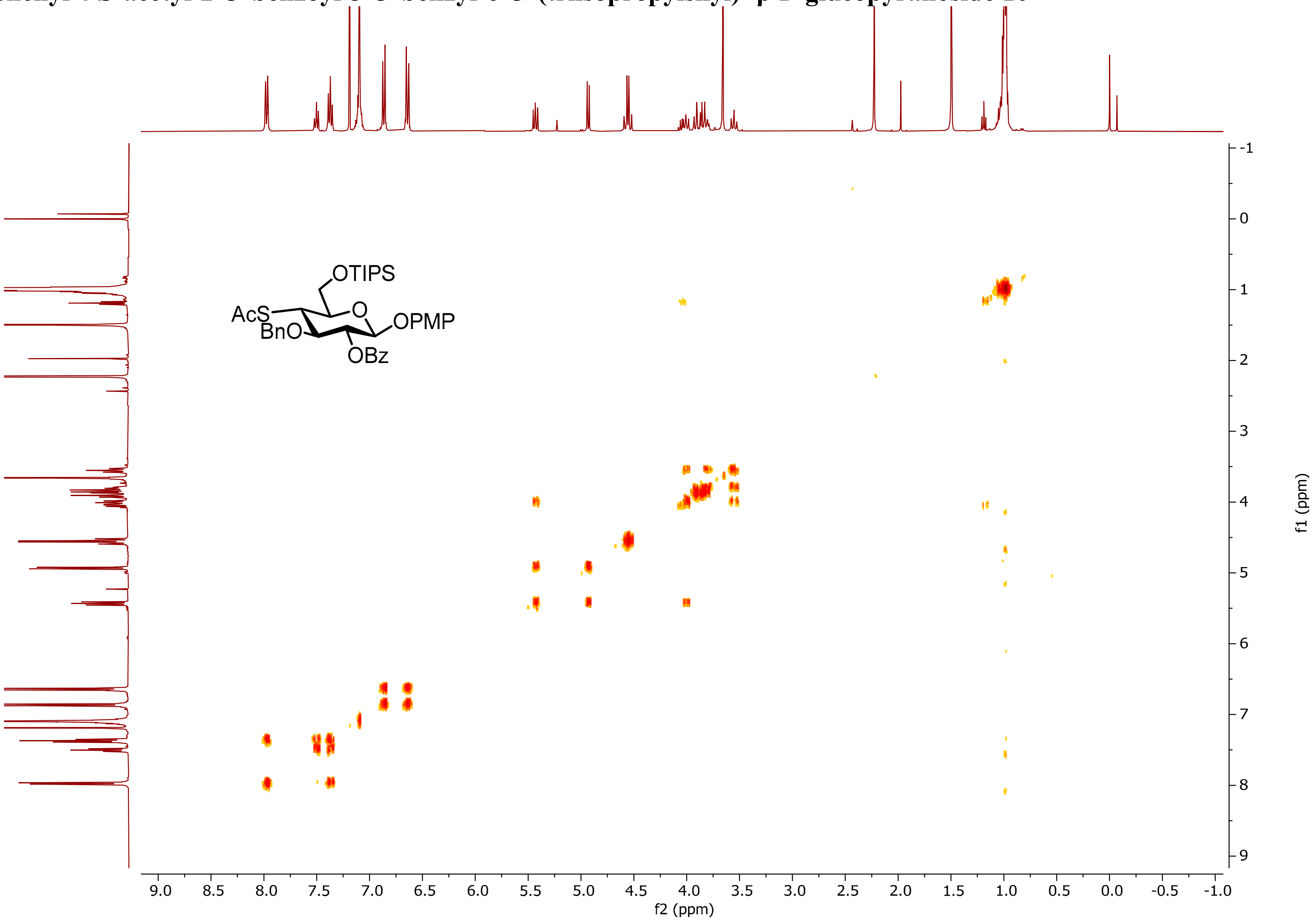

COSY NMR (400 MHz, Chloroform-d)

_p_-Methoxyphenyl 4-_S_-acetyl-2-_O_-benzoyl-3-_O_-benzyl-6-_O_-(triisopropylsilyl)- β-D-glucopyranoside 10

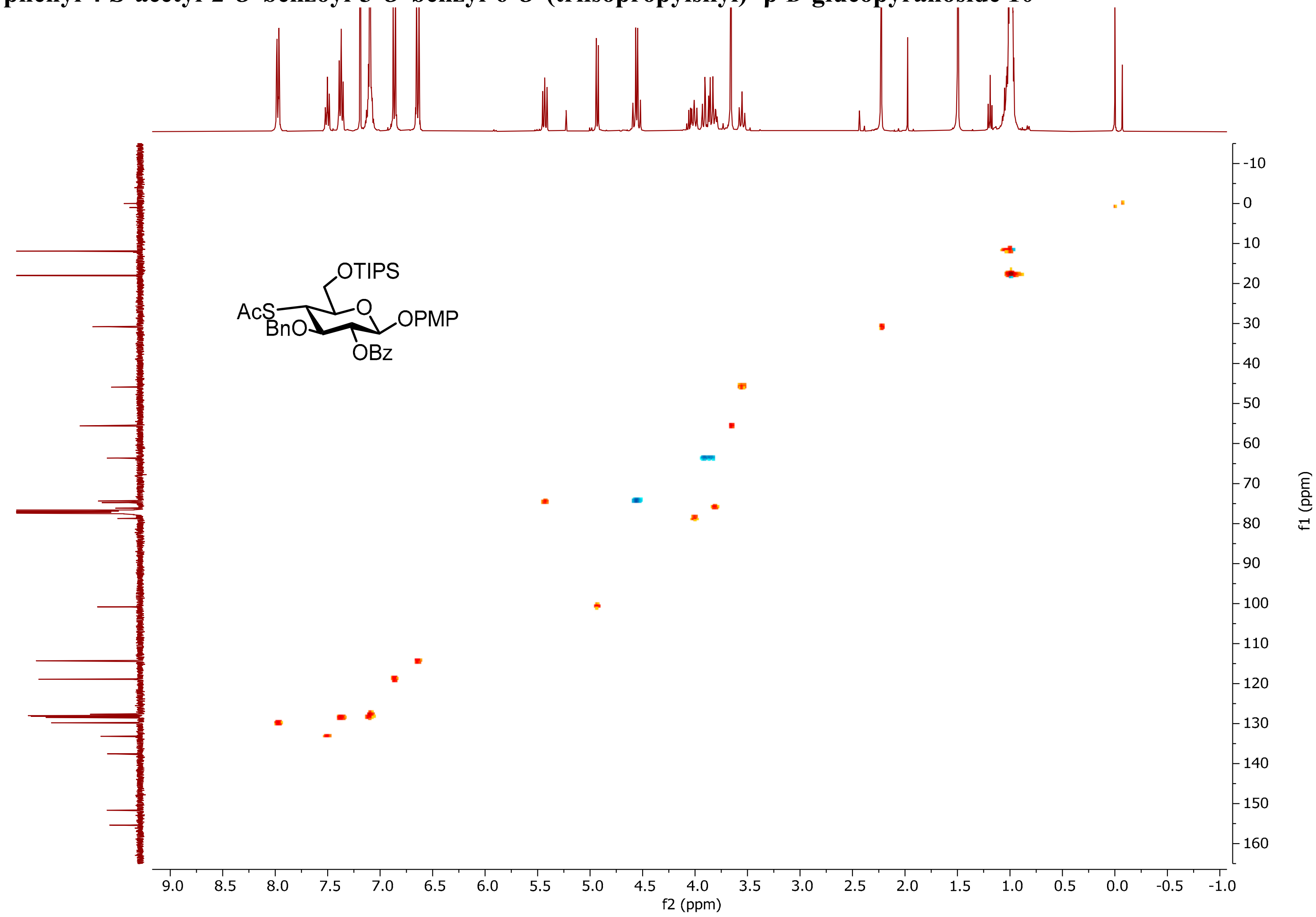

HSQC NMR (400 x 100 MHz, Chloroform-_d_)

p-Methoxyphenyl 4-S-acetyl-2-O-benzoyl-3-O-benzyl-6-O-(triisopropylsilyl)- β-D-glucopyranoside 10

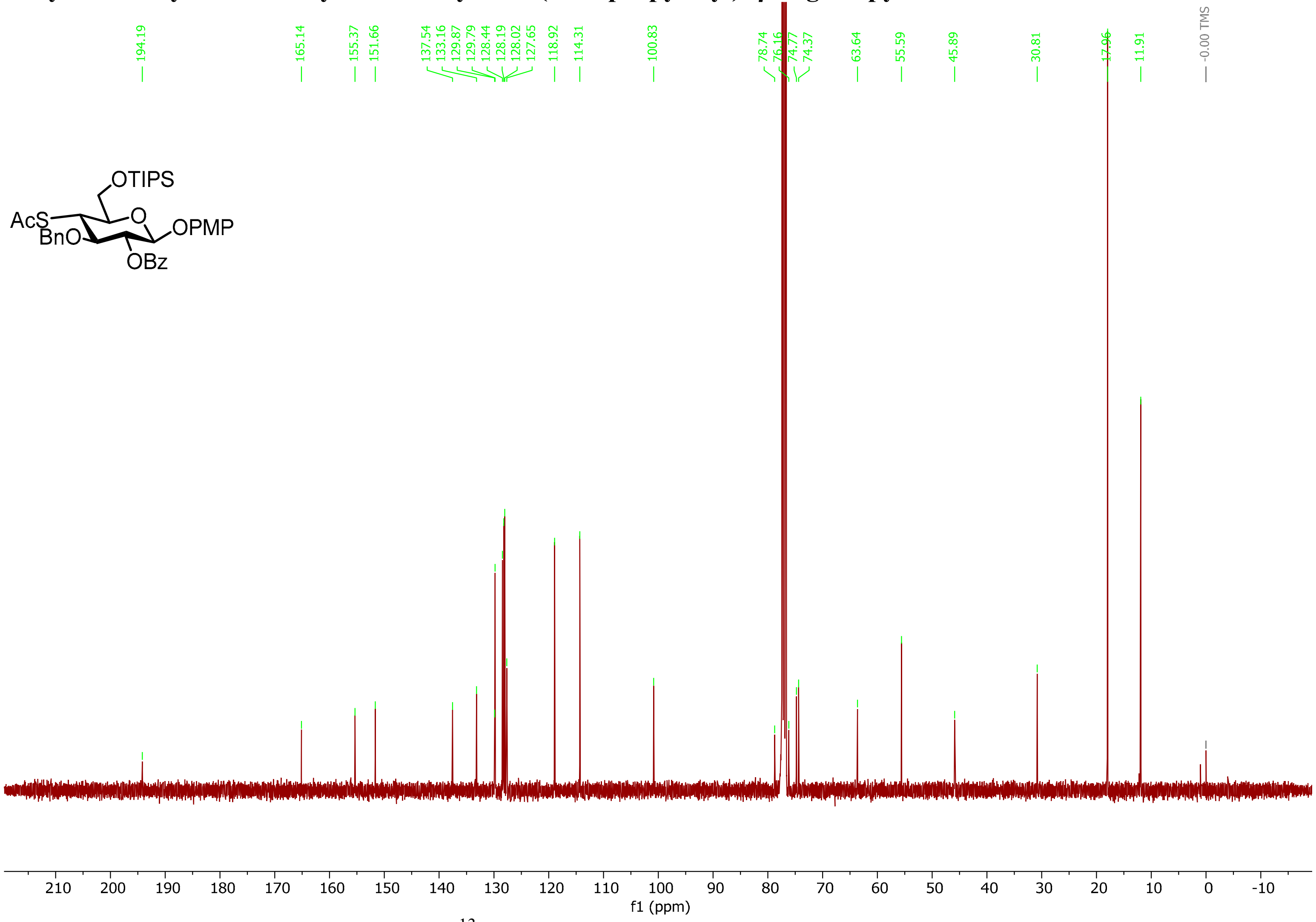

^{13}C NMR (100 MHz, Chloroform-*d*)

_p_-Methoxyphenyl 2,3-di-_O_-benzoyl-4-thio-6-_O_-(triisopropylsilyl)- β-D-glucopyranoside 12

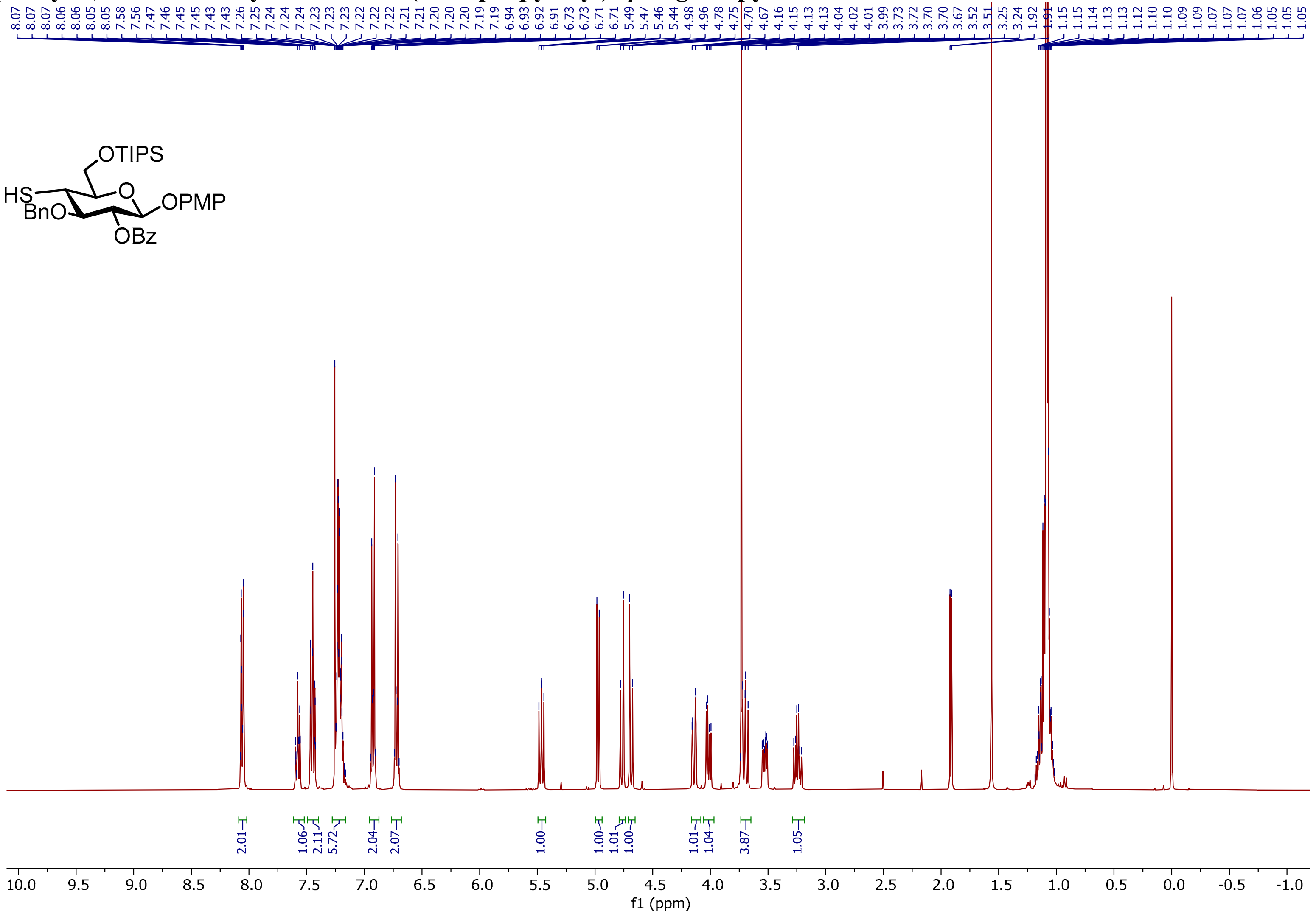

^{1}H NMR (400 MHz, Chloroform-*d*)

p-Methoxyphenyl 2,3-di-O-benzoyl-4-thio-6-O-(triisopropylsilyl)- β-D-glucopyranoside 12

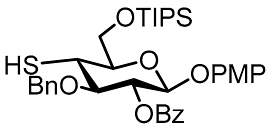

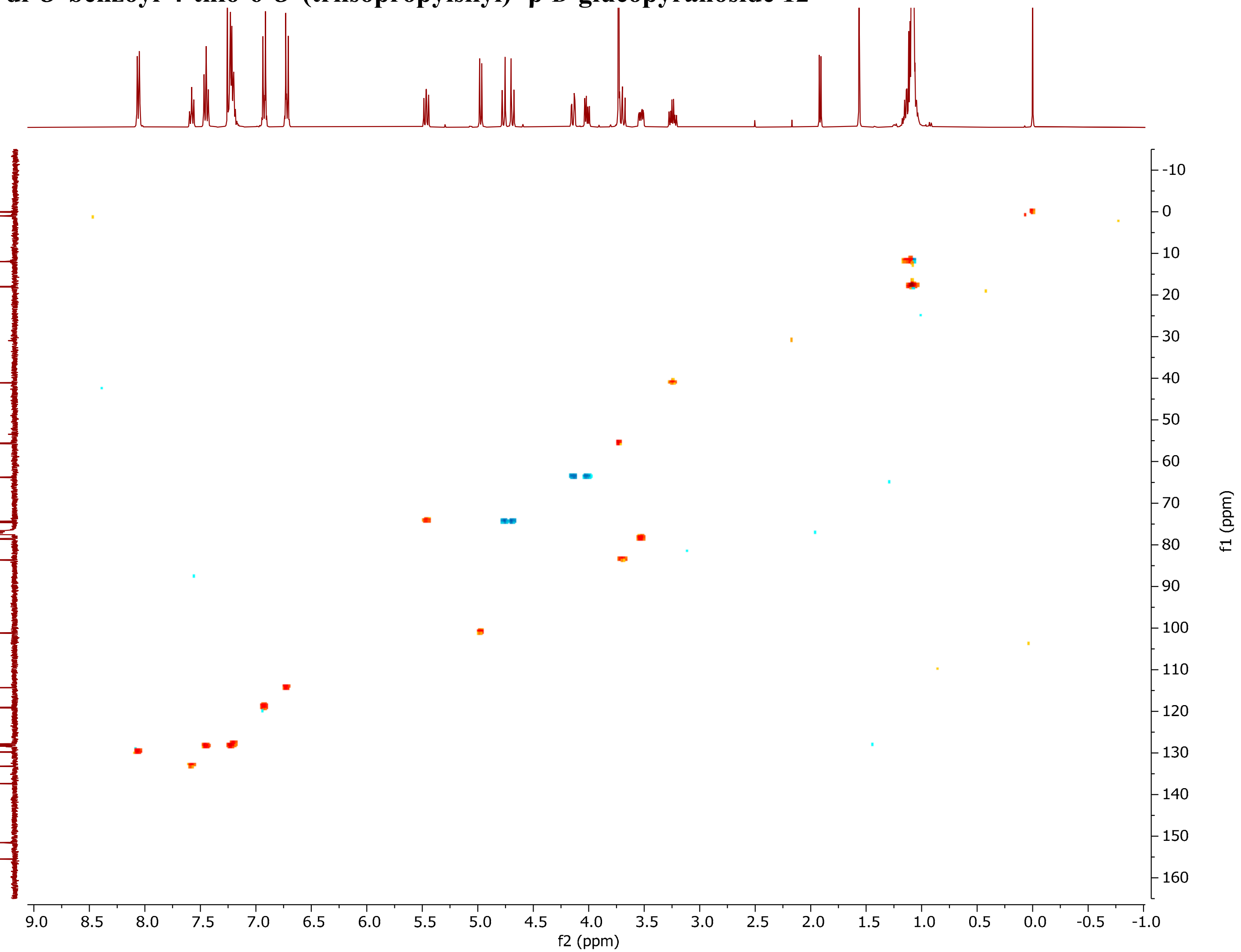

HSQC NMR (400 x 100 MHz, Chloroform-d)

_p_-Methoxyphenyl 2,3-di-_O_-benzoyl-4-thio-6-_O_-(triisopropylsilyl)- β-D-glucopyranoside 12

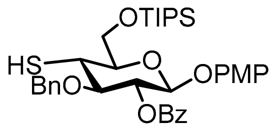

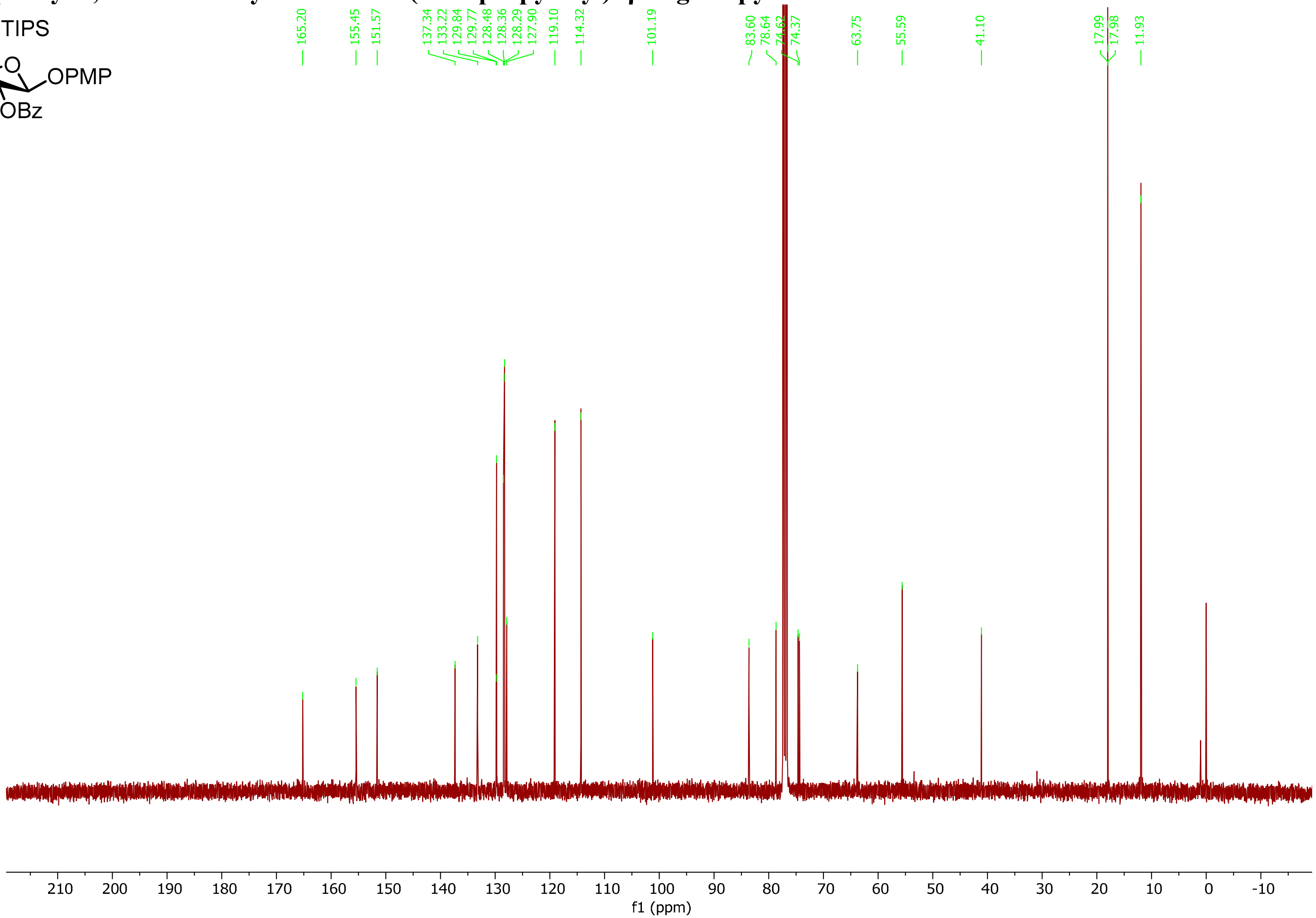

^{13}C NMR (100 MHz, Chloroform-*d*)

Synthesis of Glucosamine Donors

***p*-Methylphenyl 2-azido-3-*O*-benzyl -4,6-*O*-benzylidene-2-deoxy -1-thio-β-D-glucopyranoside S9**

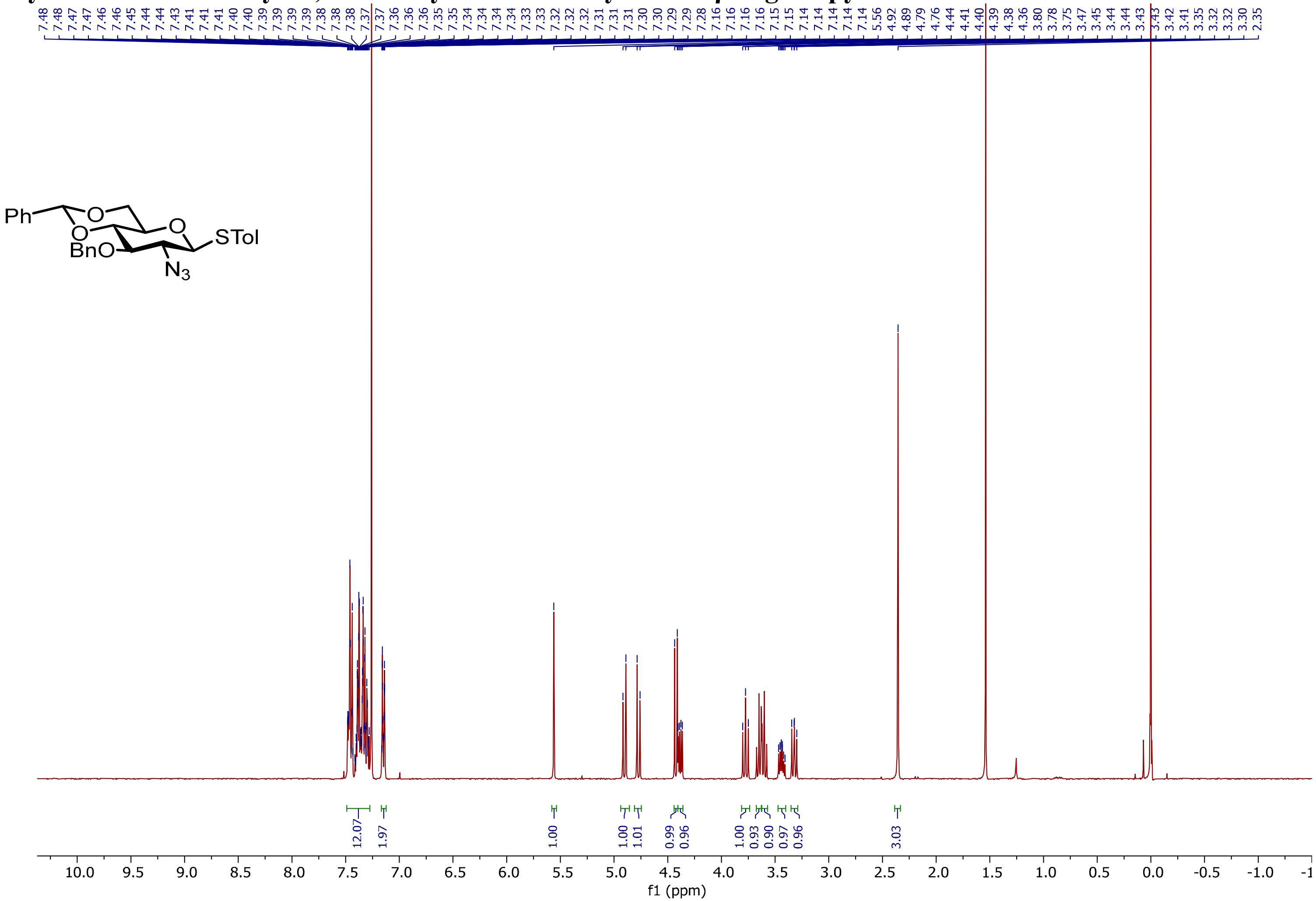

^{1}H NMR (400 MHz, Chloroform-*d*)

p-Methylphenyl 2-azido-3-*O*-benzyl -4,6-*O*-benzylidene-2-deoxy -1-thio-β-D-glucopyranoside S9

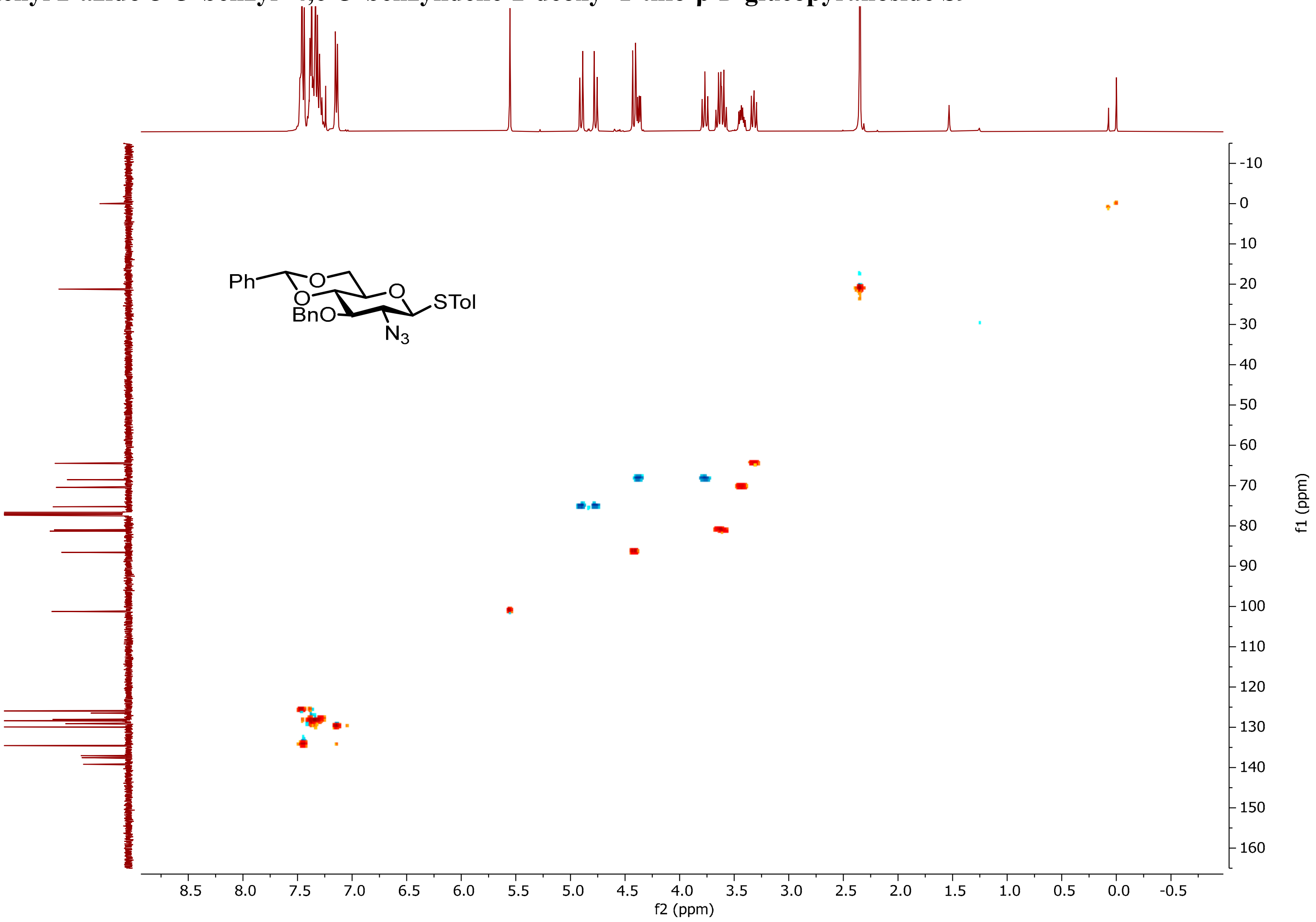

HSQC NMR (400 x 100 MHz, Chloroform-*d*)

Methylphenyl 2-azido-3-*O*-benzyl -4,6-*O*-benzylidene-2-deoxy -1-thio-β-D-glucopyranoside S9

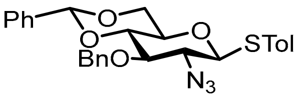

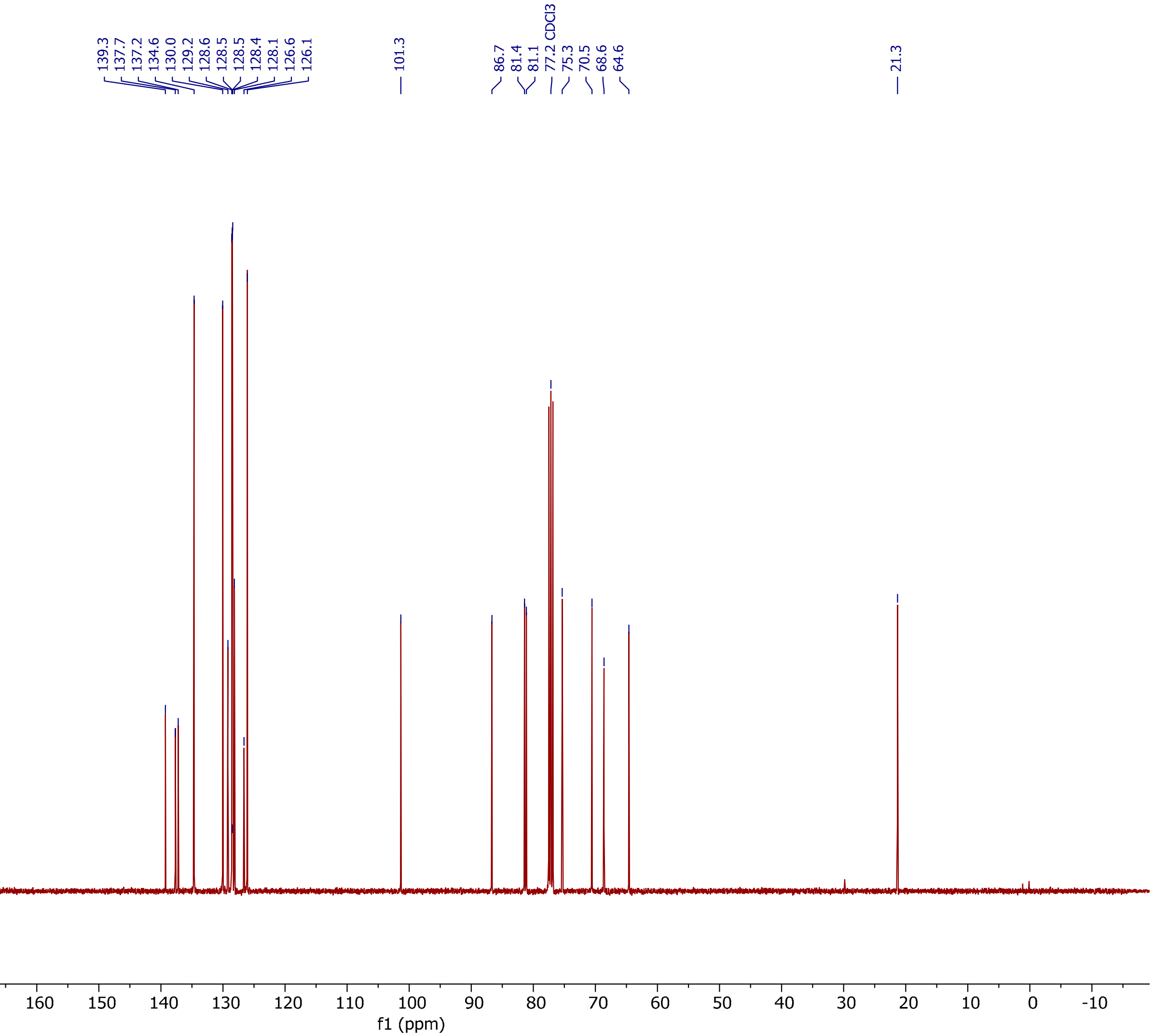

^{13}C NMR (100 MHz, Chloroform-*d*)

***p*-Methylphenyl 2-azido-3-*O*-benzyl-2-deoxy-1-thio-β-D-glucopyranoside S10**

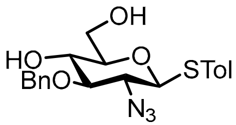

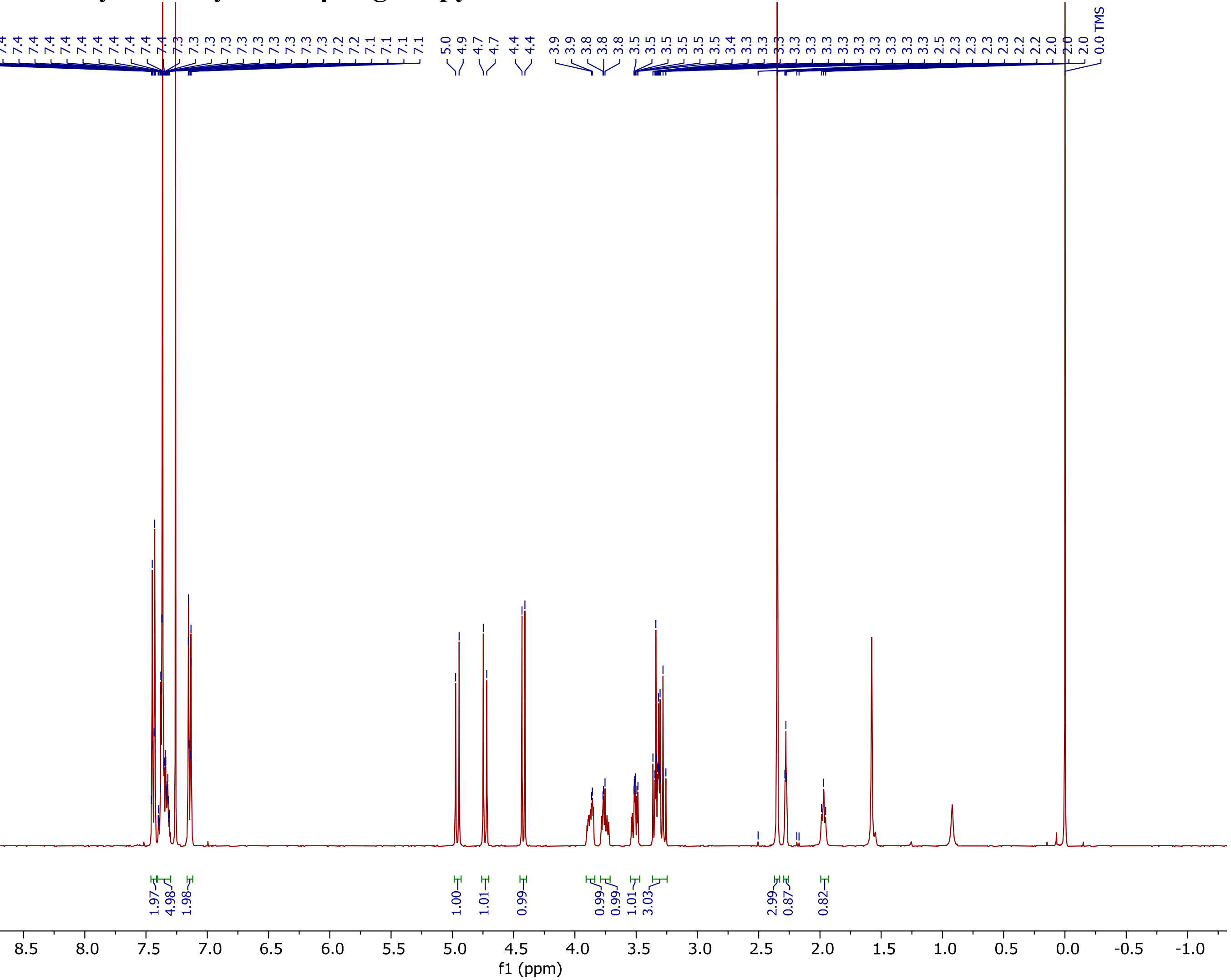

^{1}H NMR (400 MHz, Chloroform-*d*)

p-Methylphenyl 2-azido-3-O-benzyl-2-deoxy-1-thio-β-D-glucopyranoside S10

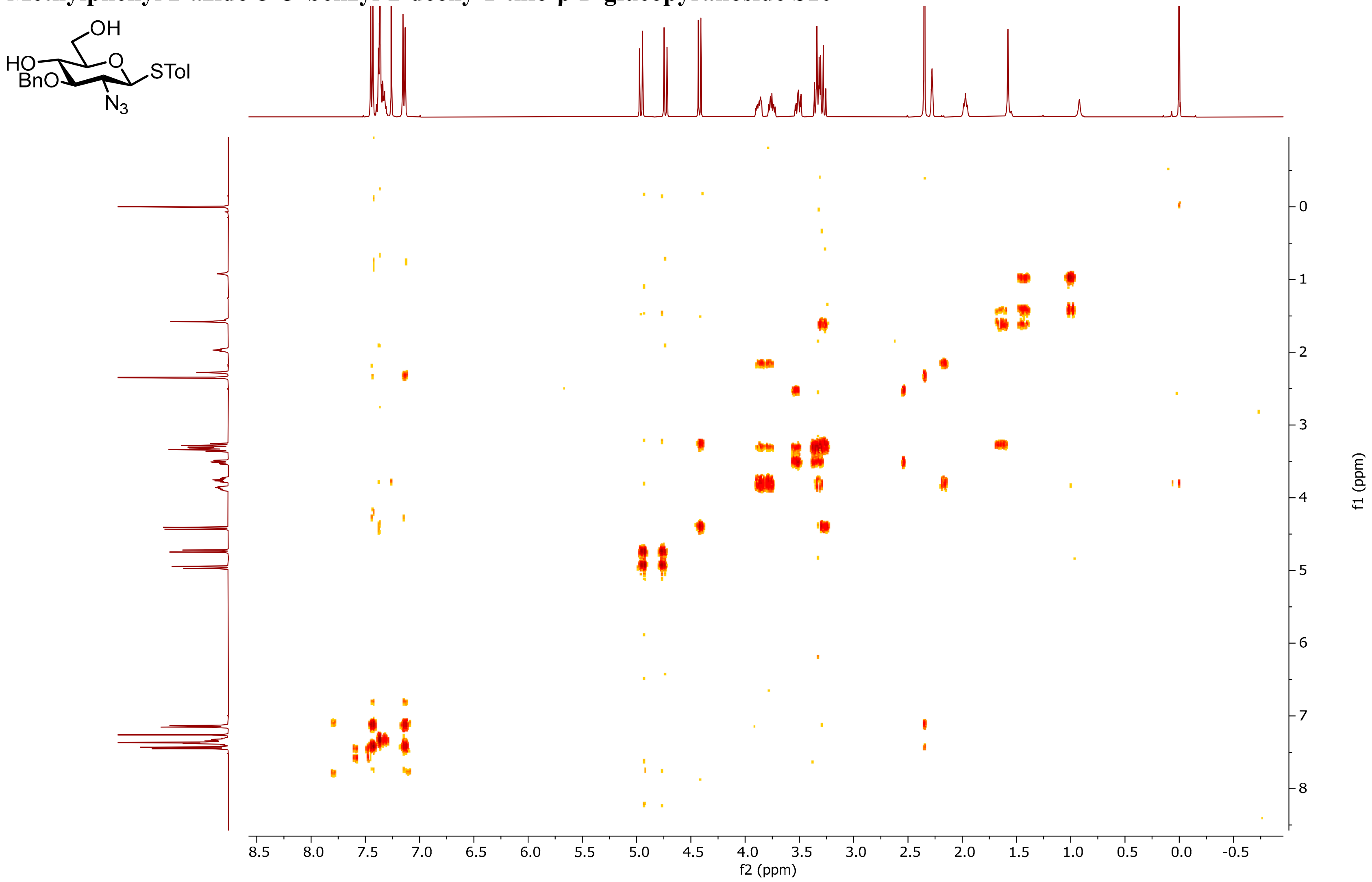

COSY NMR (400 MHz, Chloroform-d)

_p_-Methylphenyl 2-azido-3-_O_-benzyl-2-deoxy-1-thio-β-D-glucopyranoside S10

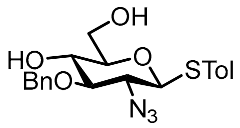

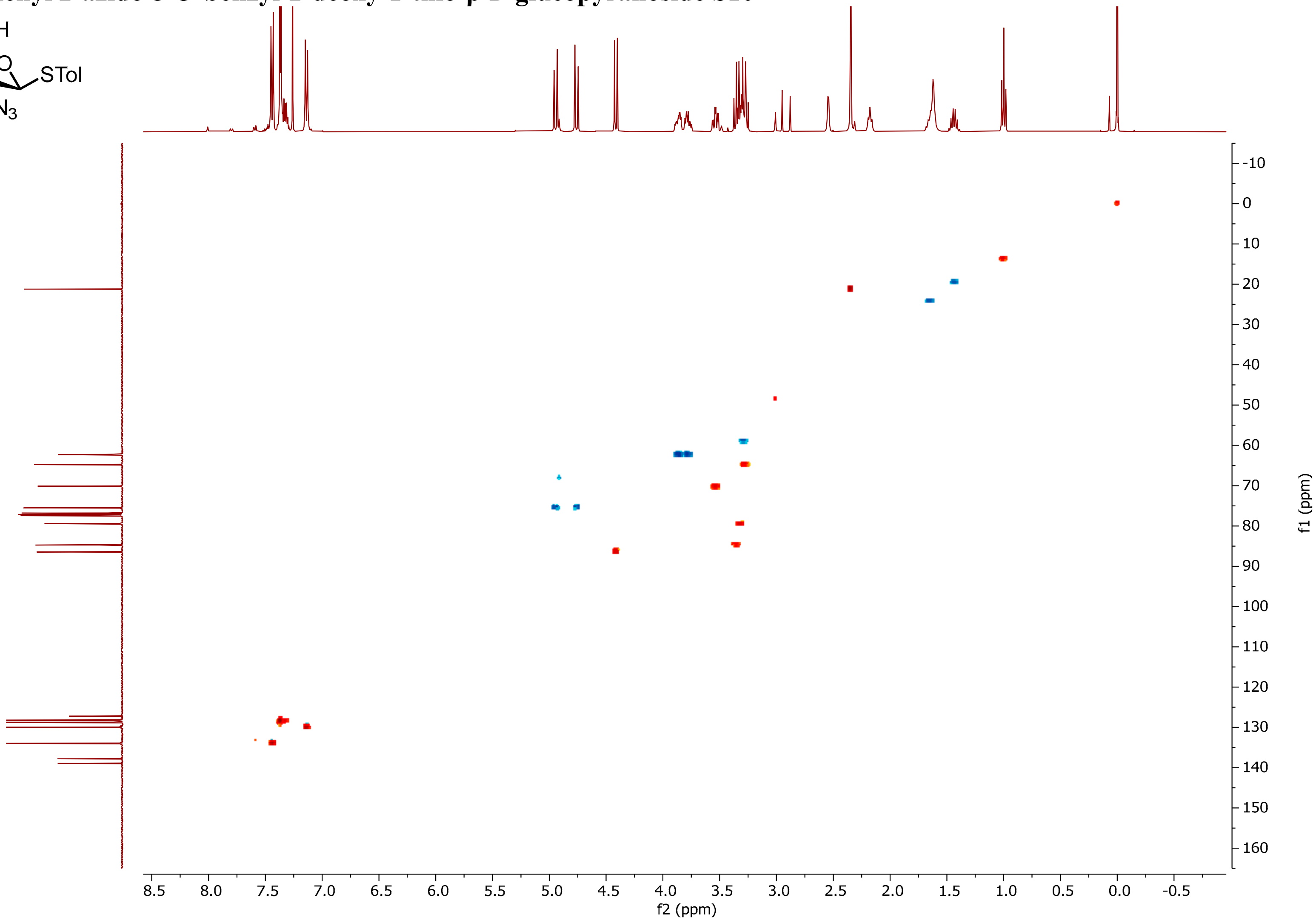

HSQC NMR (400 x 100 MHz, Chloroform-_d_)

_p_-Methylphenyl 2-azido-3-_O_-benzyl-2-deoxy-1-thio-β-D-glucopyranoside S10

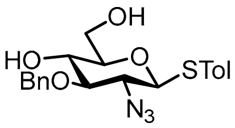

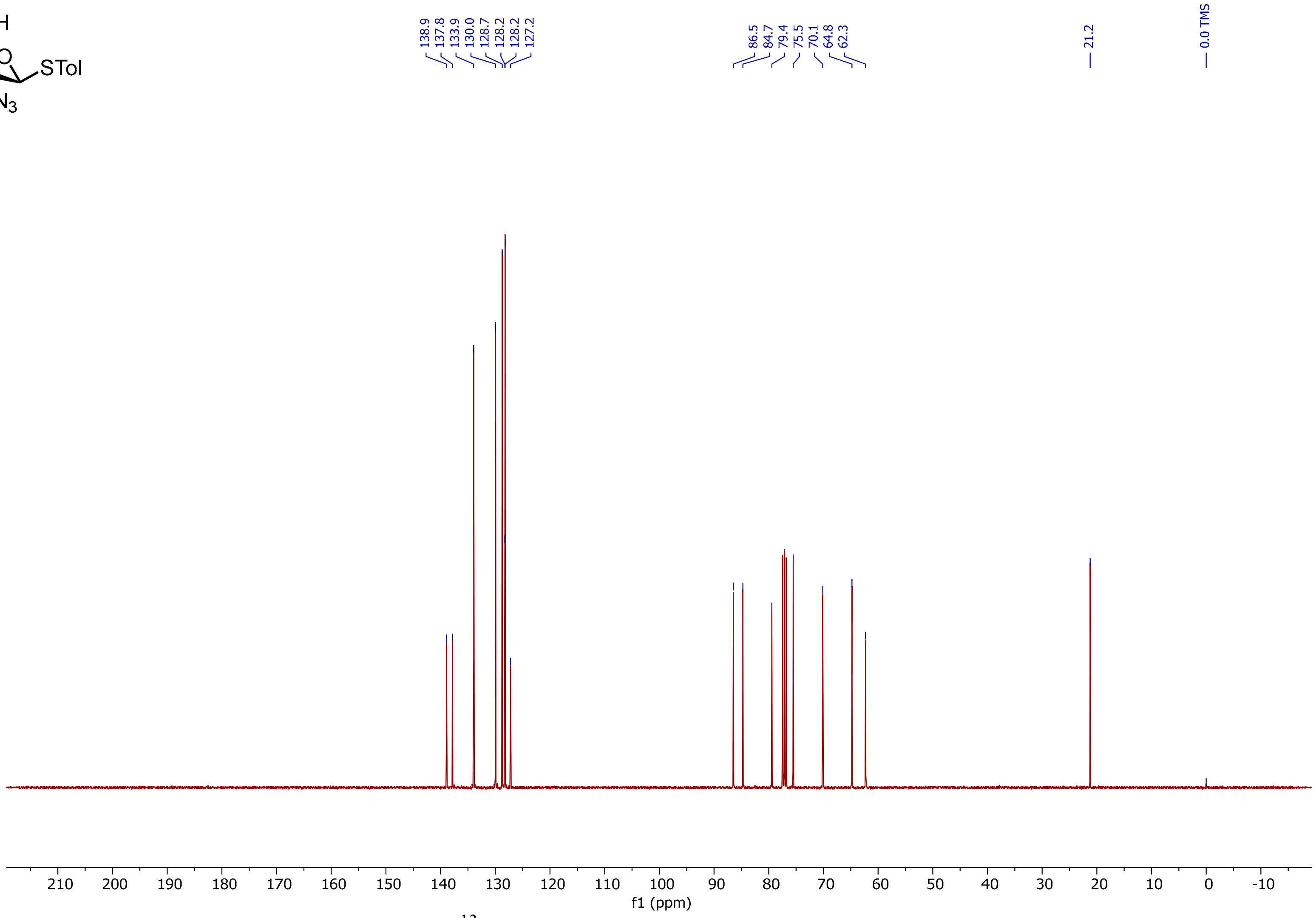

^{13}C NMR (100 MHz, Chloroform-*d*)

p-Methylphenyl 2-azido-3-O-benzyl -2-deoxy-4-O-levulinoyl-6-O-(tert-butyldiphenylsilyl)-1-thio-β-D-glucopyranoside 17

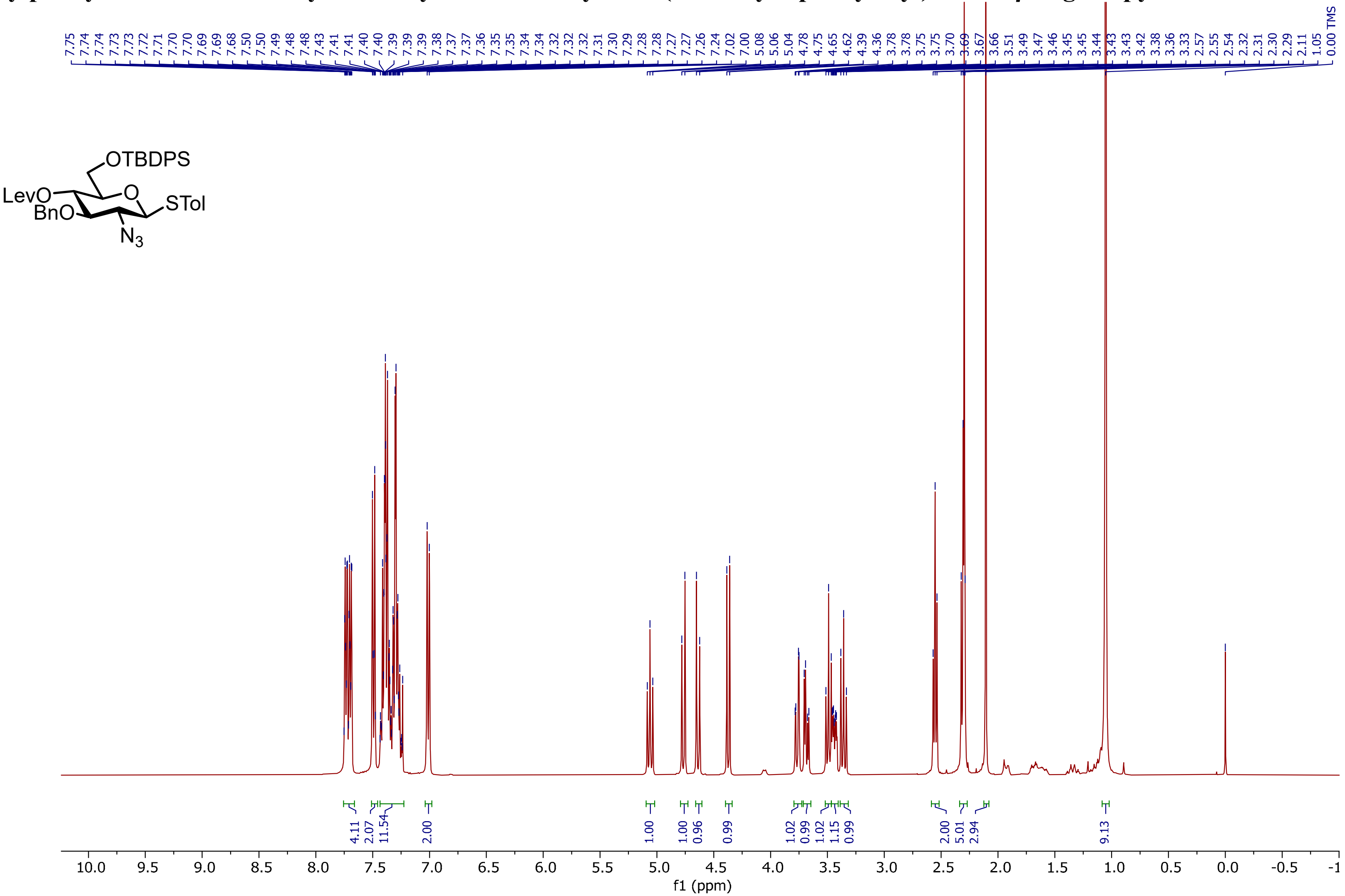

^{1}H NMR (400 MHz, Chloroform-*d*)

p-Methylphenyl 2-azido-3-O-benzyl -2-deoxy-4-O-levulinoyl-6-O-(tert-butyldiphenylsilyl)-1-thio-β-D-glucopyranoside 17

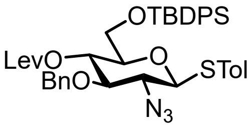

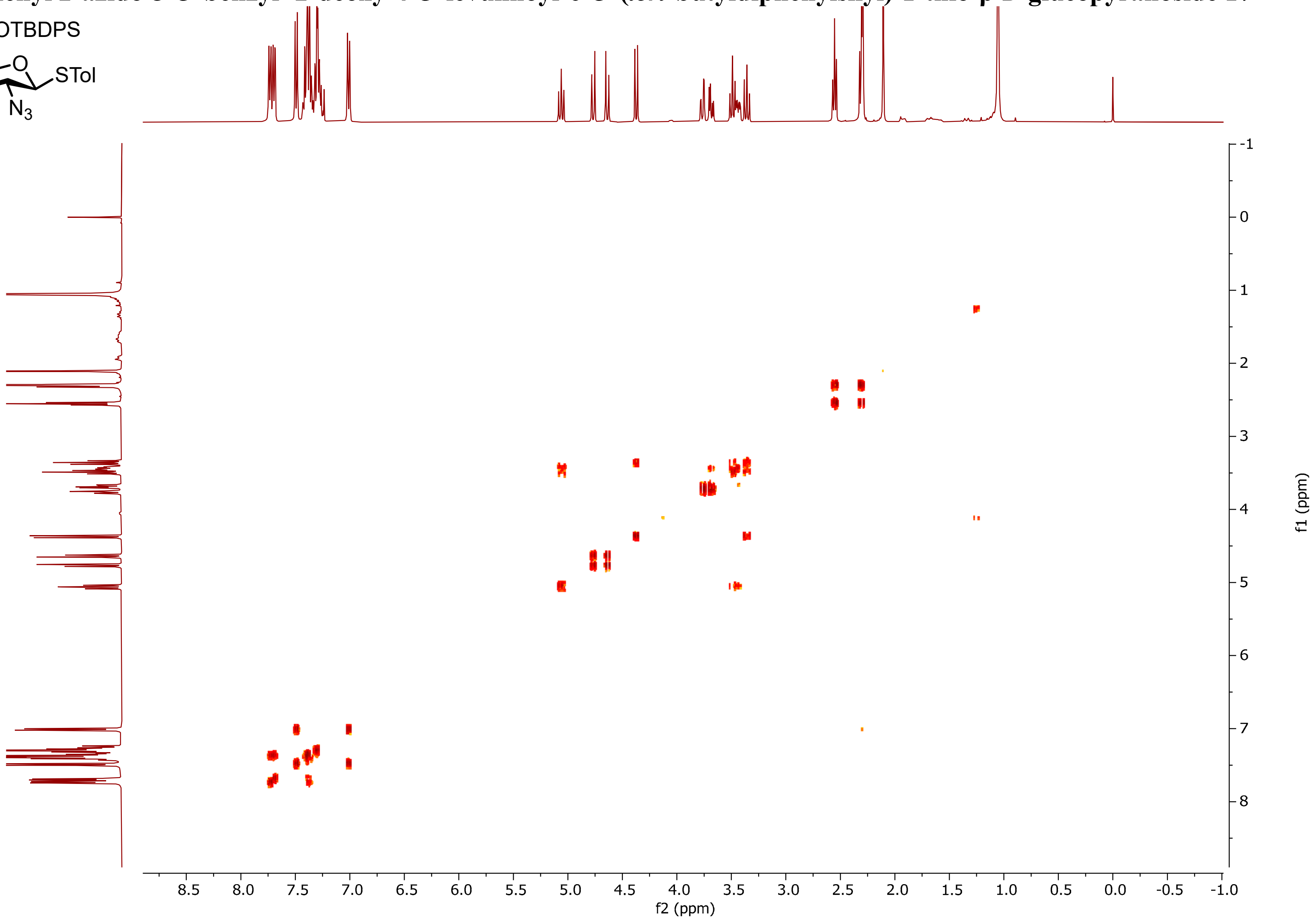

COSY NMR (400 MHz, Chloroform-*d*)

_p_-Methylphenyl 2-azido-3-_O_-benzyl -2-deoxy-4-_O_-levulinoyl-6-_O_-(_tert_-butyldiphenylsilyl)-1-thio-β-D-glucopyranoside 17

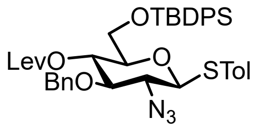

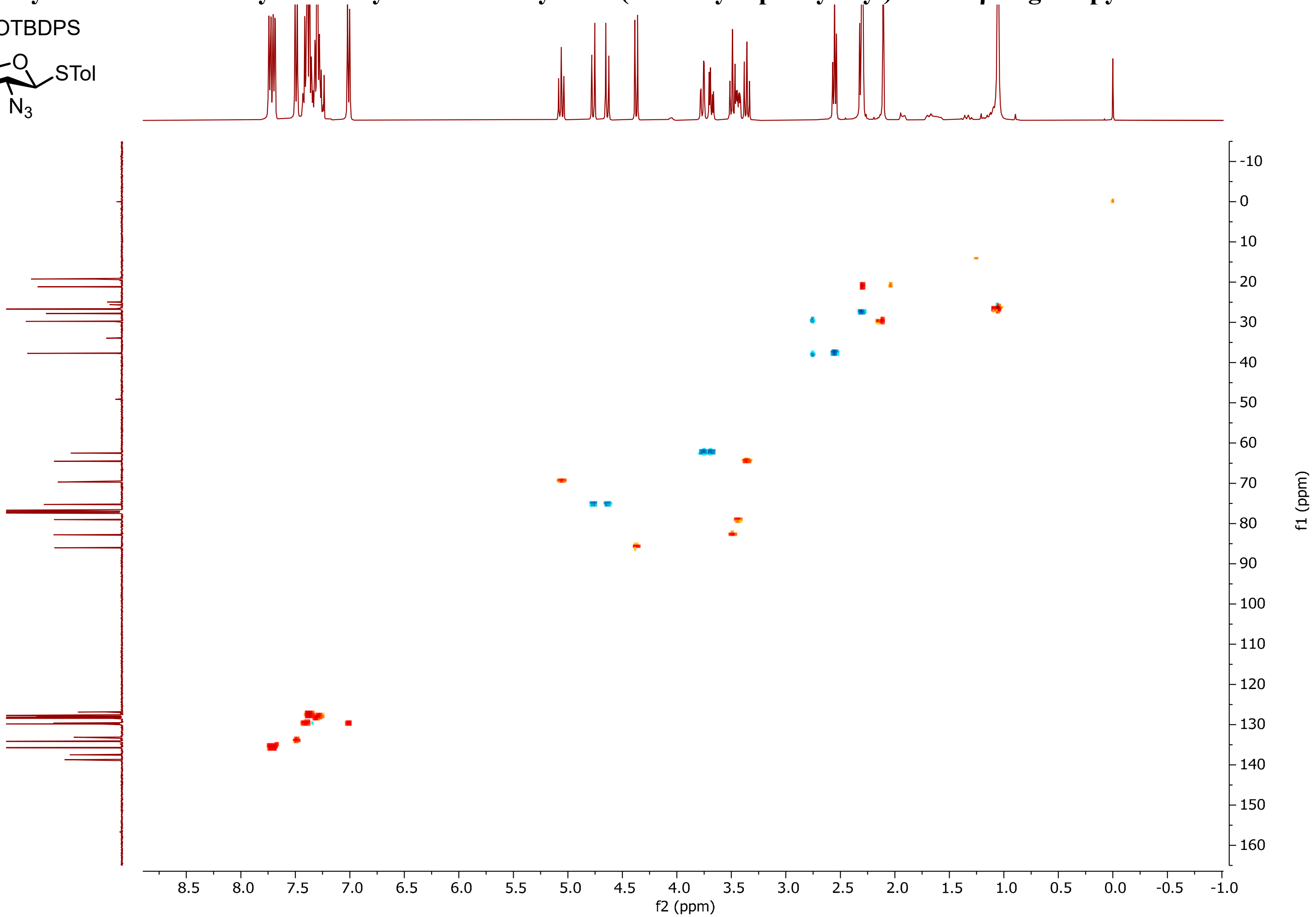

HSQC NMR (400 x 100 MHz, Chloroform-_d_)

p-Methylphenyl 2-azido-3-O-benzyl -2-deoxy-4-O-levulinoyl-6-O-(tert-butyldiphenylsilyl)-1-thio-β-D-glucopyranoside 17

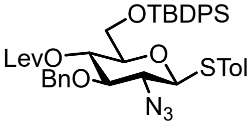

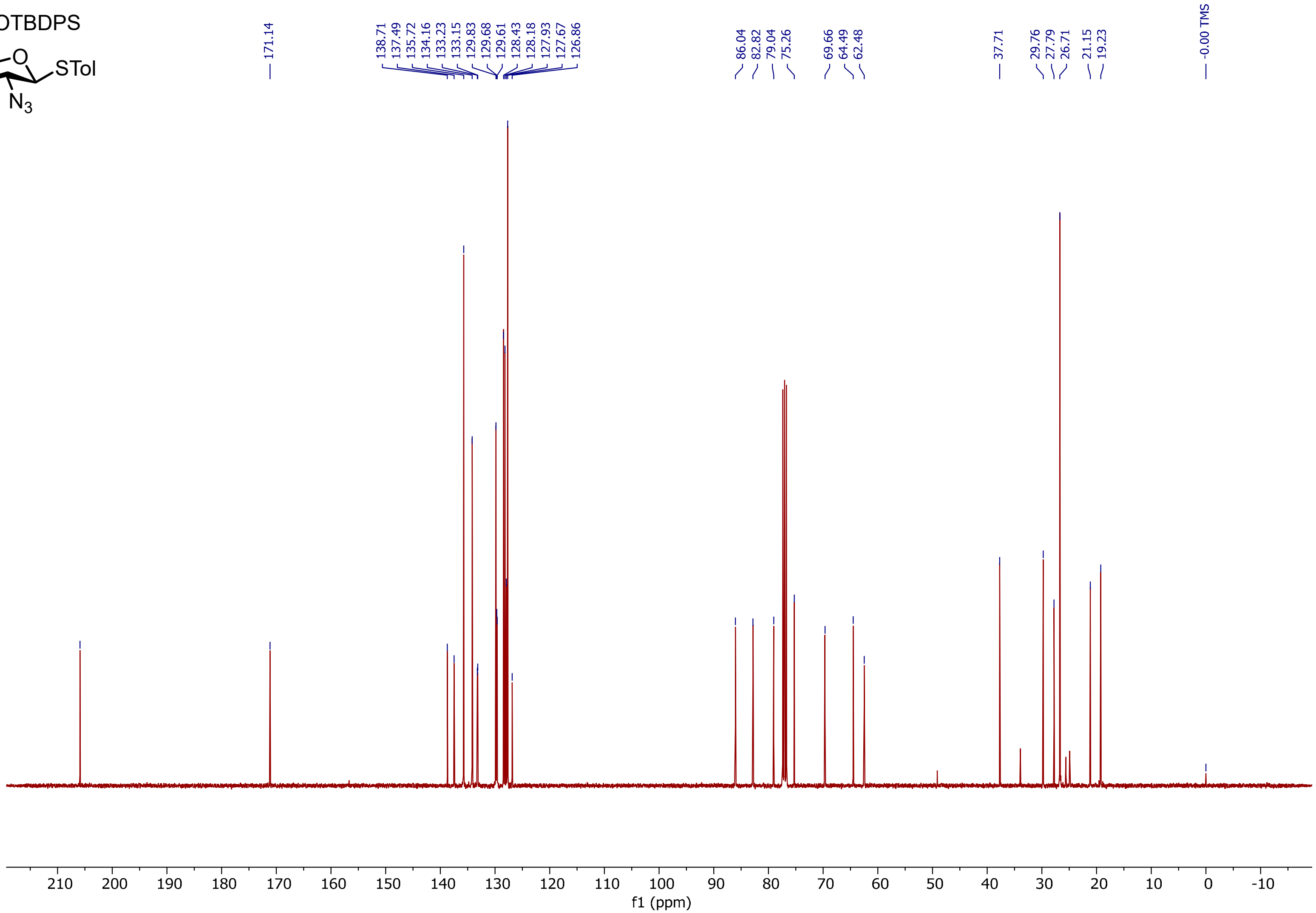

^{13}C NMR (100 MHz, Chloroform-*d*)

2-Azido-3-*O*-benzyl-2-deoxy-4-*O*-levulinoyl-6-*O*-(*tert*-butyldiphenylsilyl)-β-D-glucopyranose 16

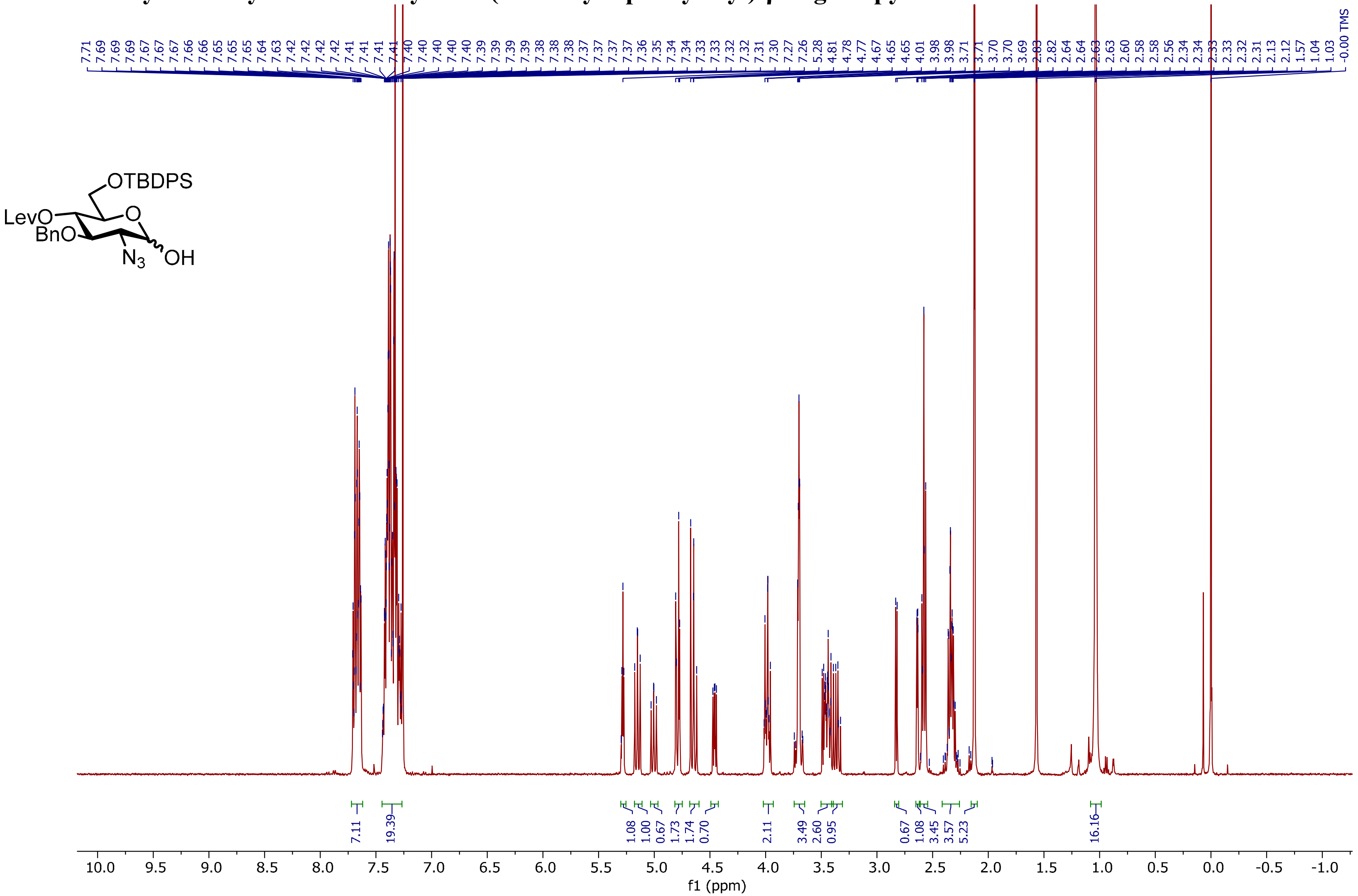

^{1}H NMR (400 MHz, Chloroform-*d*)

2-Azido-3-*O*-benzyl-2-deoxy-4-*O*-levulinoyl-6-*O*-(*tert*-butyldiphenylsilyl)-β-D-glucopyranose 16

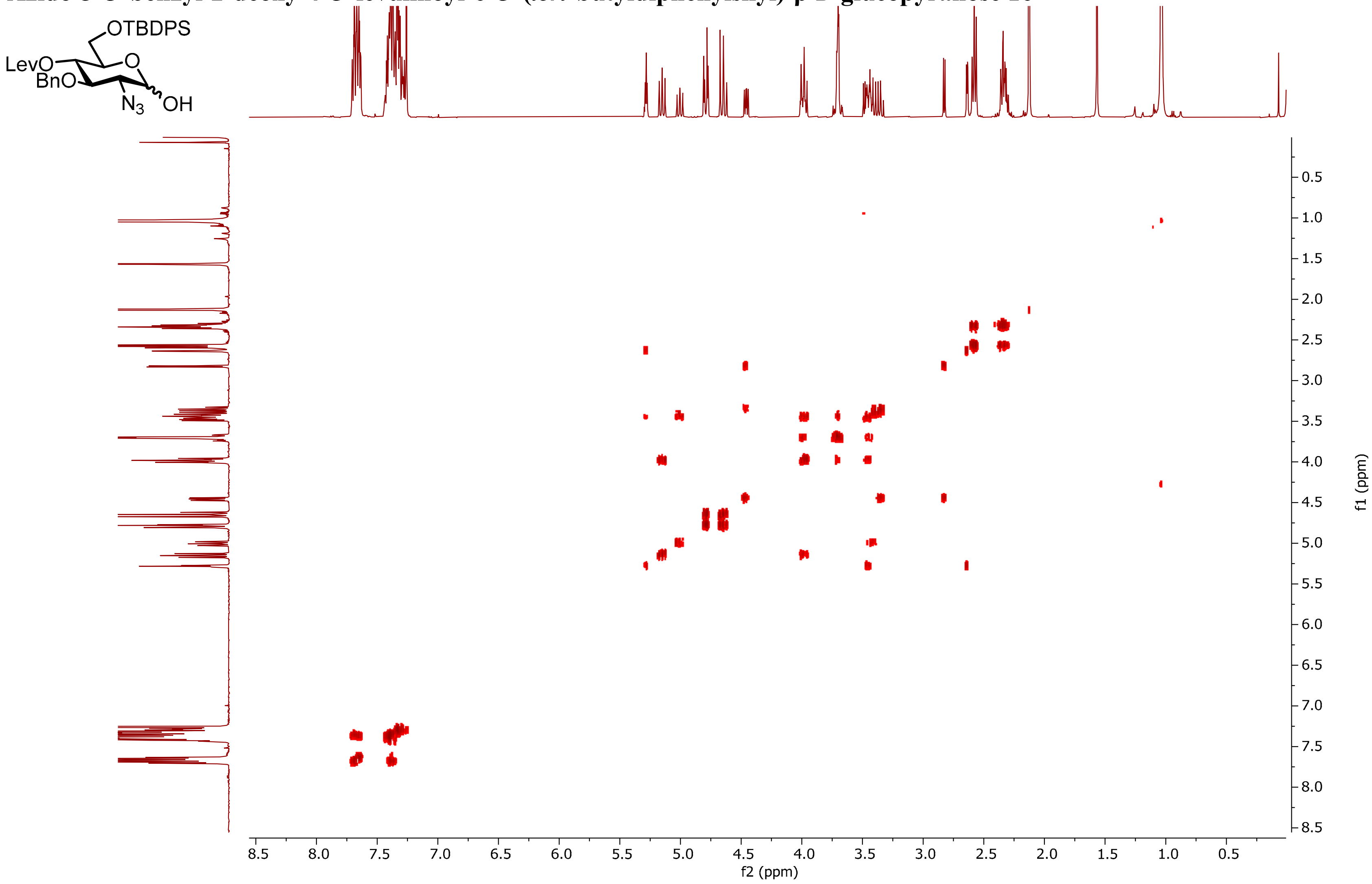

COSY NMR (400 MHz, Chloroform-*d*)

2-Azido-3-*O*-benzyl-2-deoxy-4-*O*-levulinoyl-6-*O*-(*tert*-butyldiphenylsilyl)-β-D-glucopyranose 16

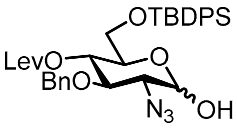

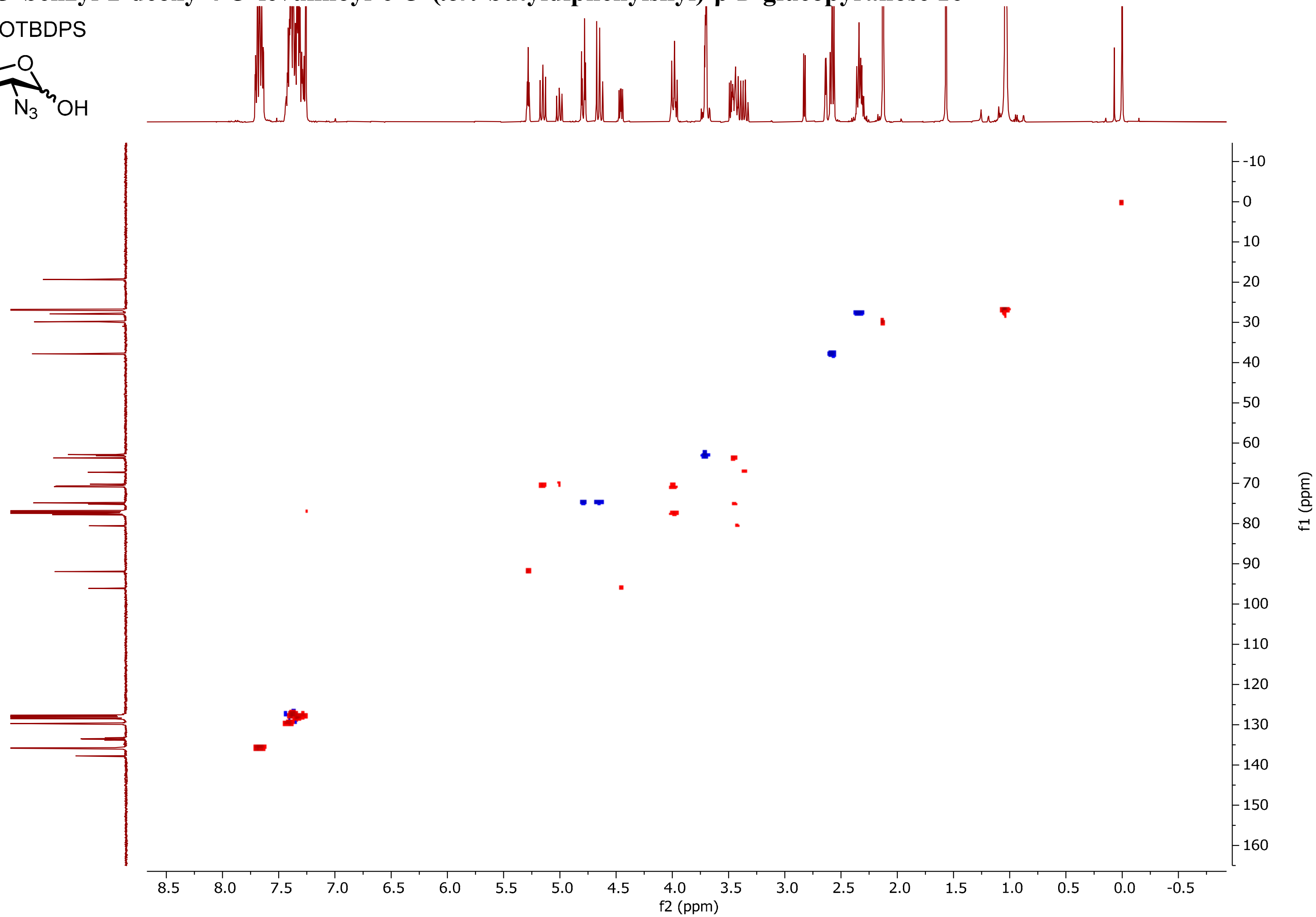

HSQC NMR (400 x 100 MHz, Chloroform-*d*)

2-Azido-3-*O*-benzyl-2-deoxy-4-*O*-levulinoyl-6-*O*-(*tert*-butyldiphenylsilyl)-β-D-glucopyranose 16

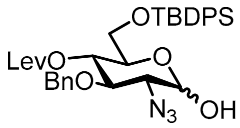

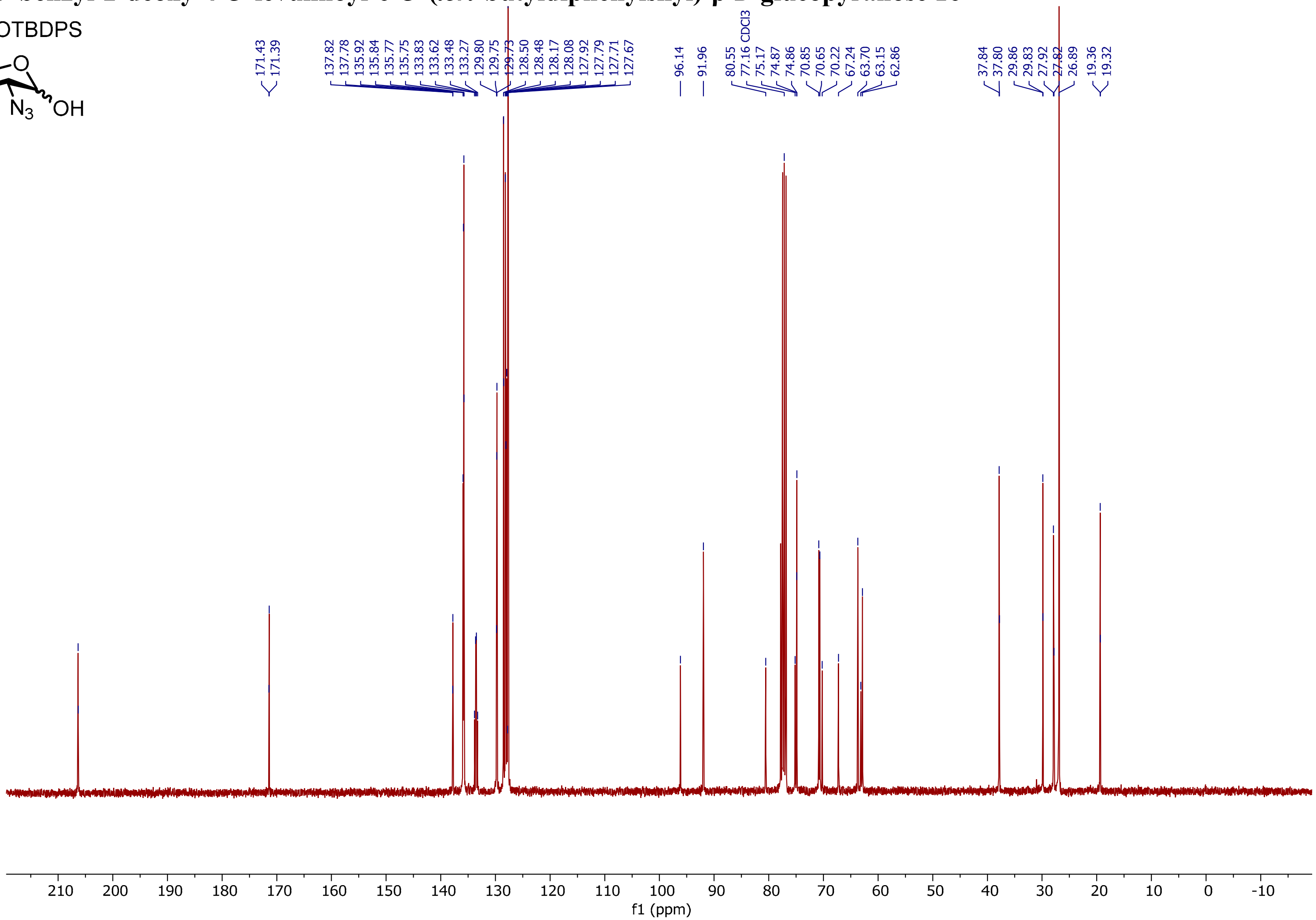

^{13}C NMR (100 MHz, Chloroform-*d*)

Dibutyl-2-Azido-3-*O*-benzyl -2-deoxy-4-*O*-levulinoyl-6-*O*-tert-butyldiphenylsilyl-α/β-D-glucopyranosyl phosphate 18

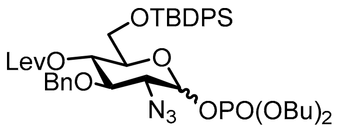

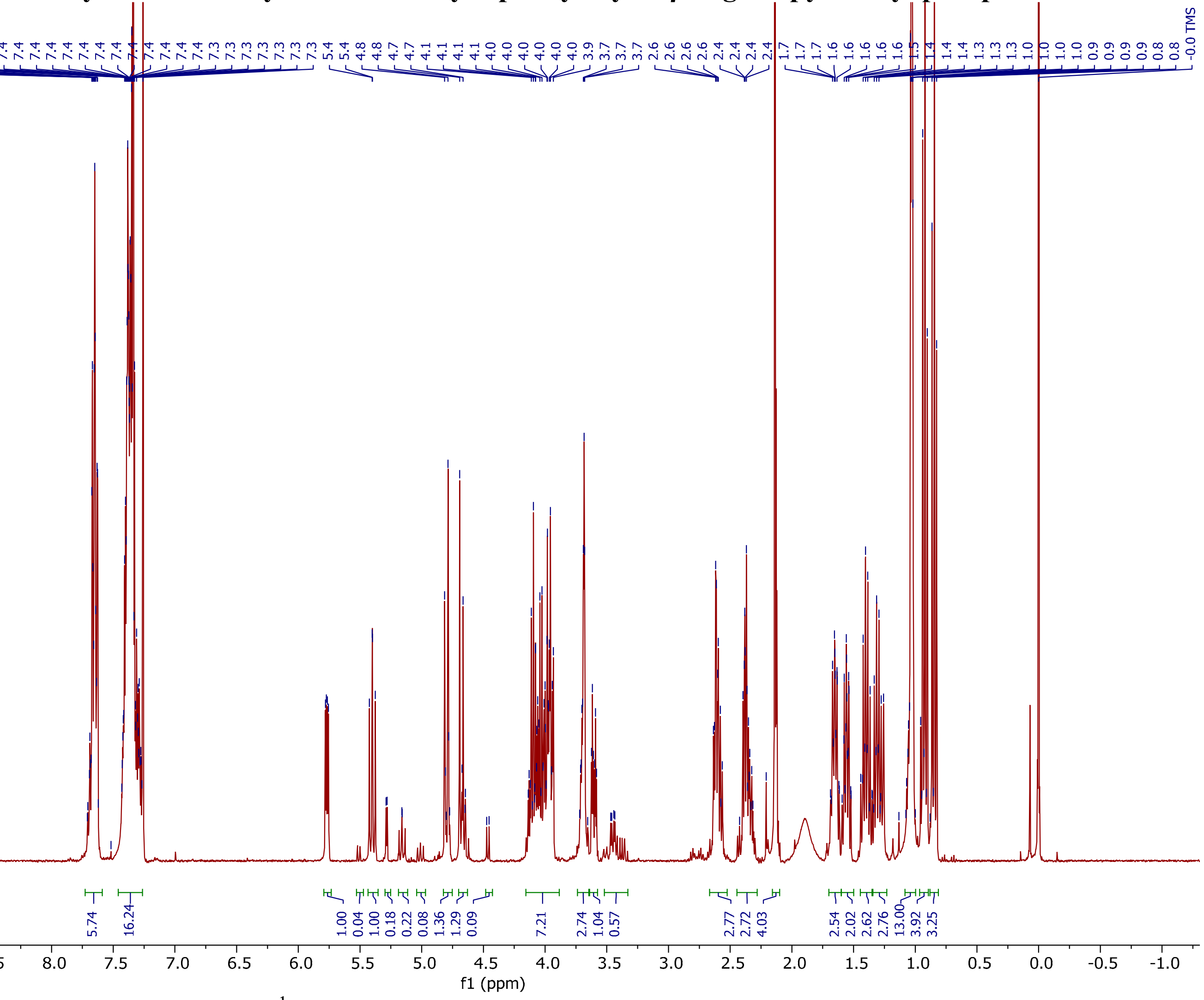

^{1}H NMR (400 MHz, Chloroform-*d*)

Dibutyl-2-Azido-3-*O*-benzyl -2-deoxy-4-*O*-levulinoyl-6-*O*-tert-butyldiphenylsilyl-α/β-D-glucopyranosyl phosphate 18

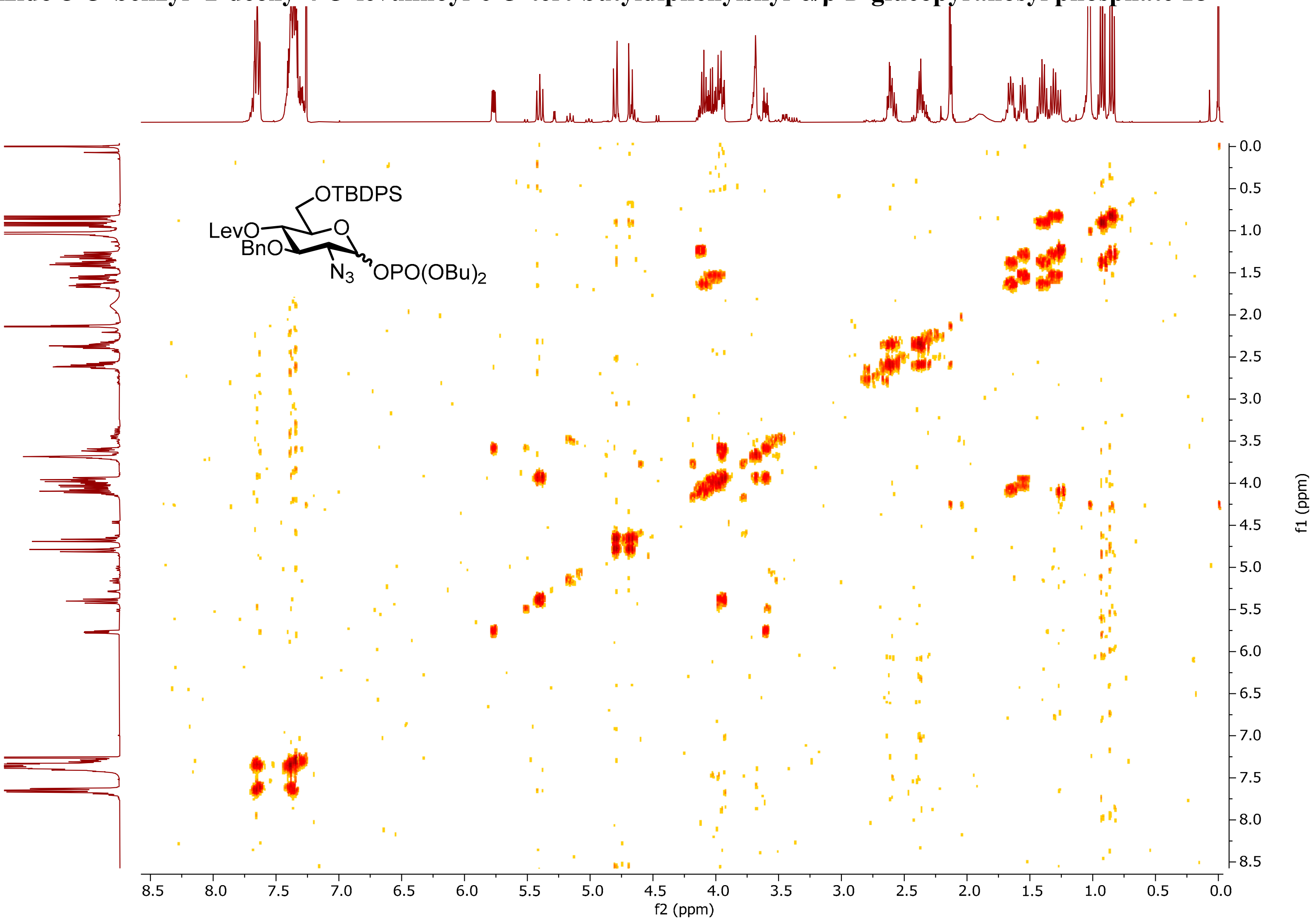

COSY NMR (400 MHz, Chloroform-*d*)

Dibutyl-2-Azido-3-*O*-benzyl -2-deoxy-4-*O*-levulinoyl-6-*O*-tert-butyldiphenylsilyl-α/β-D-glucopyranosyl phosphate 18

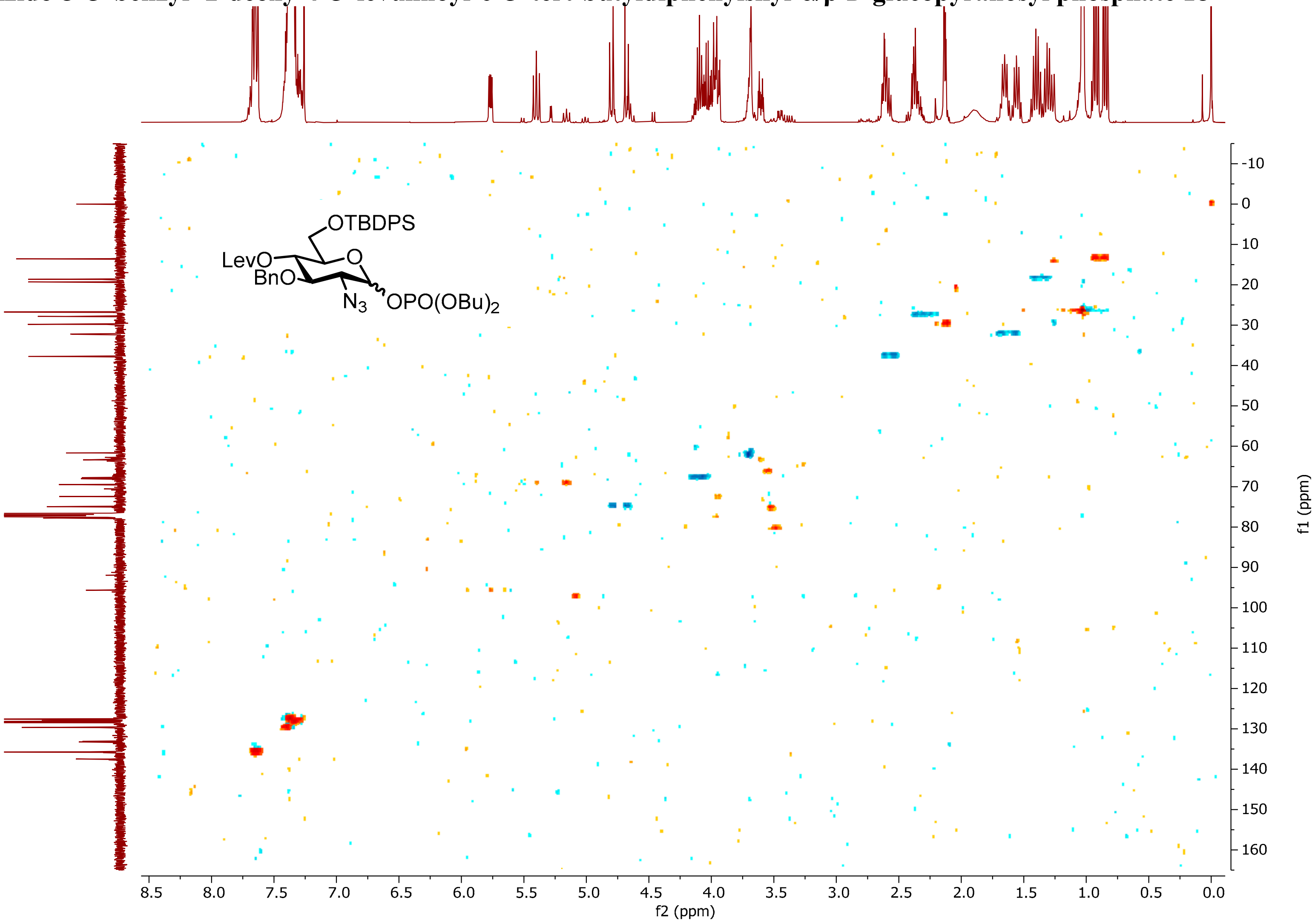

HSQC NMR (400 x 100 MHz, Chloroform-*d*)

Dibutyl-2-Azido-3-*O*-benzyl -2-deoxy-4-*O*-levulinoyl-6-*O*-tert-butyldiphenylsilyl-α/β-D-glucopyranosyl phosphate 18

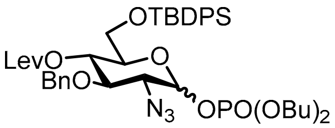

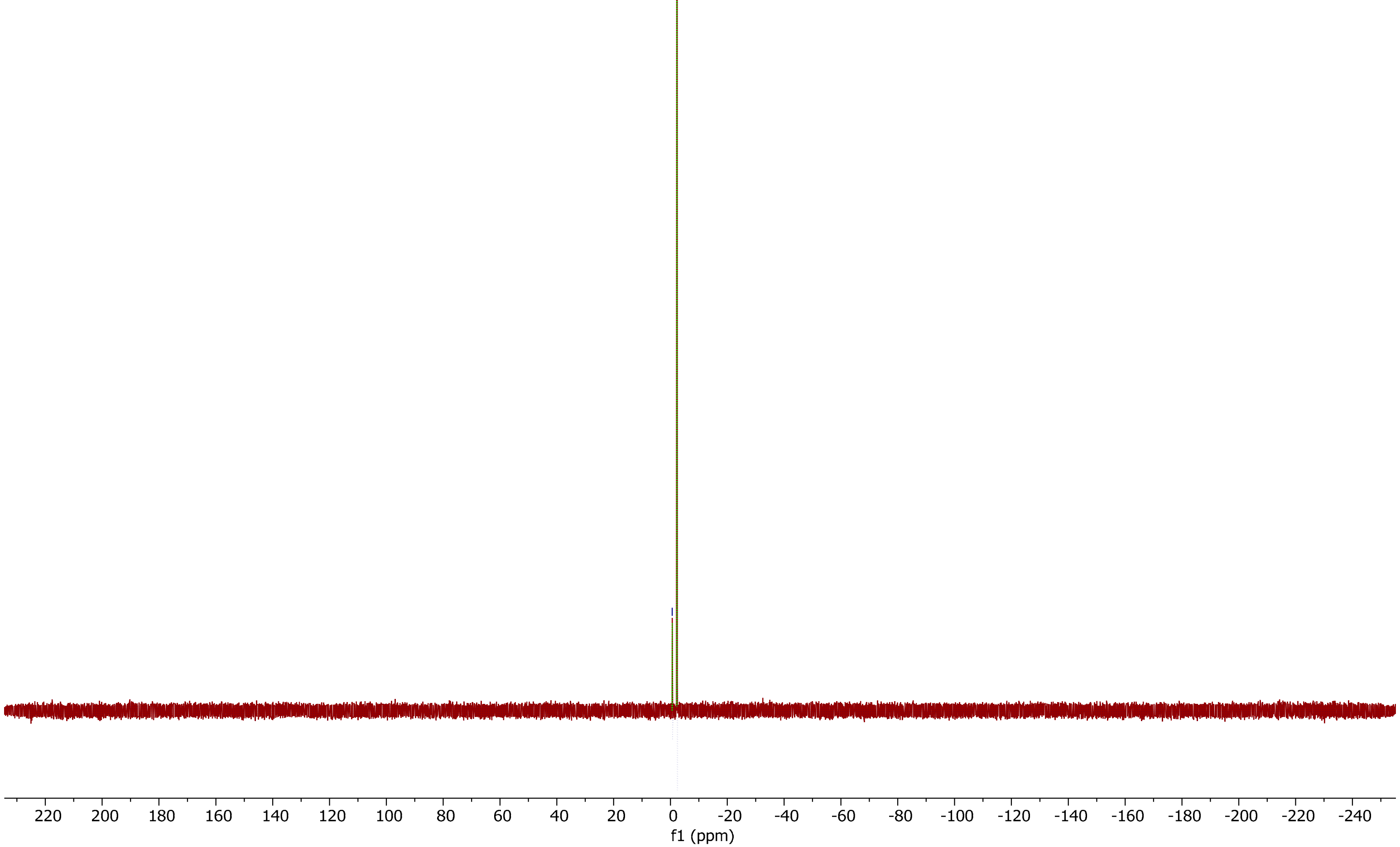

^{31}P NMR (162 MHz, Chloroform-*d*)

Dibutyl-2-Azido-3-*O*-benzyl -2-deoxy-4-*O*-levulinoyl-6-*O*-tert-butyldiphenylsilyl-α/β-D-glucopyranosyl phosphate 18

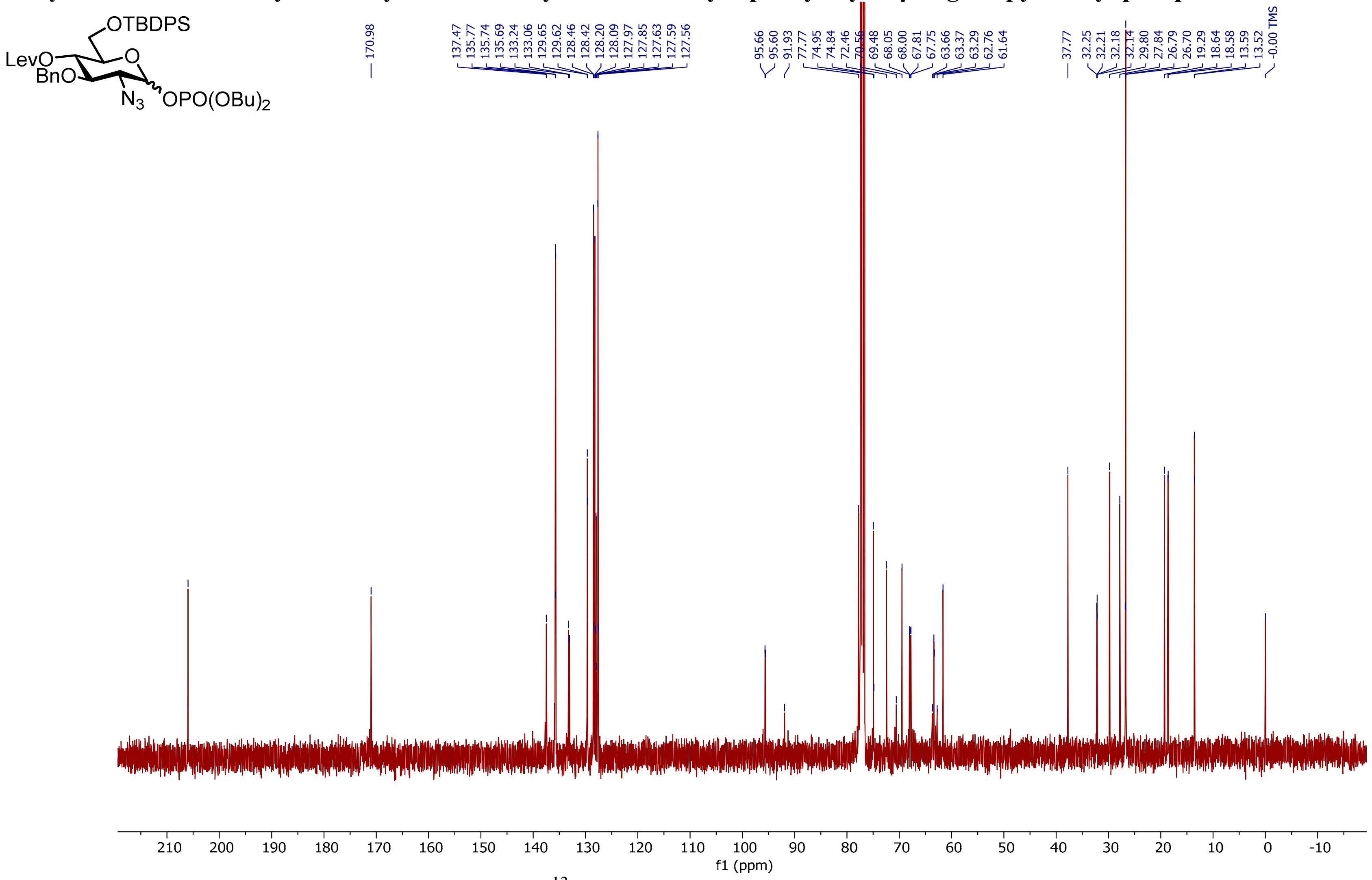

^{13}C NMR (100 MHz, Chloroform-*d*)

2-azido-3-*O*-benzyl-2-deoxy-4-*O*-levulinoyl-6-*O*-(*tert*-butyldiphenylsilyl)-β-D-glucopyranosyl trichloroacetamidate 19

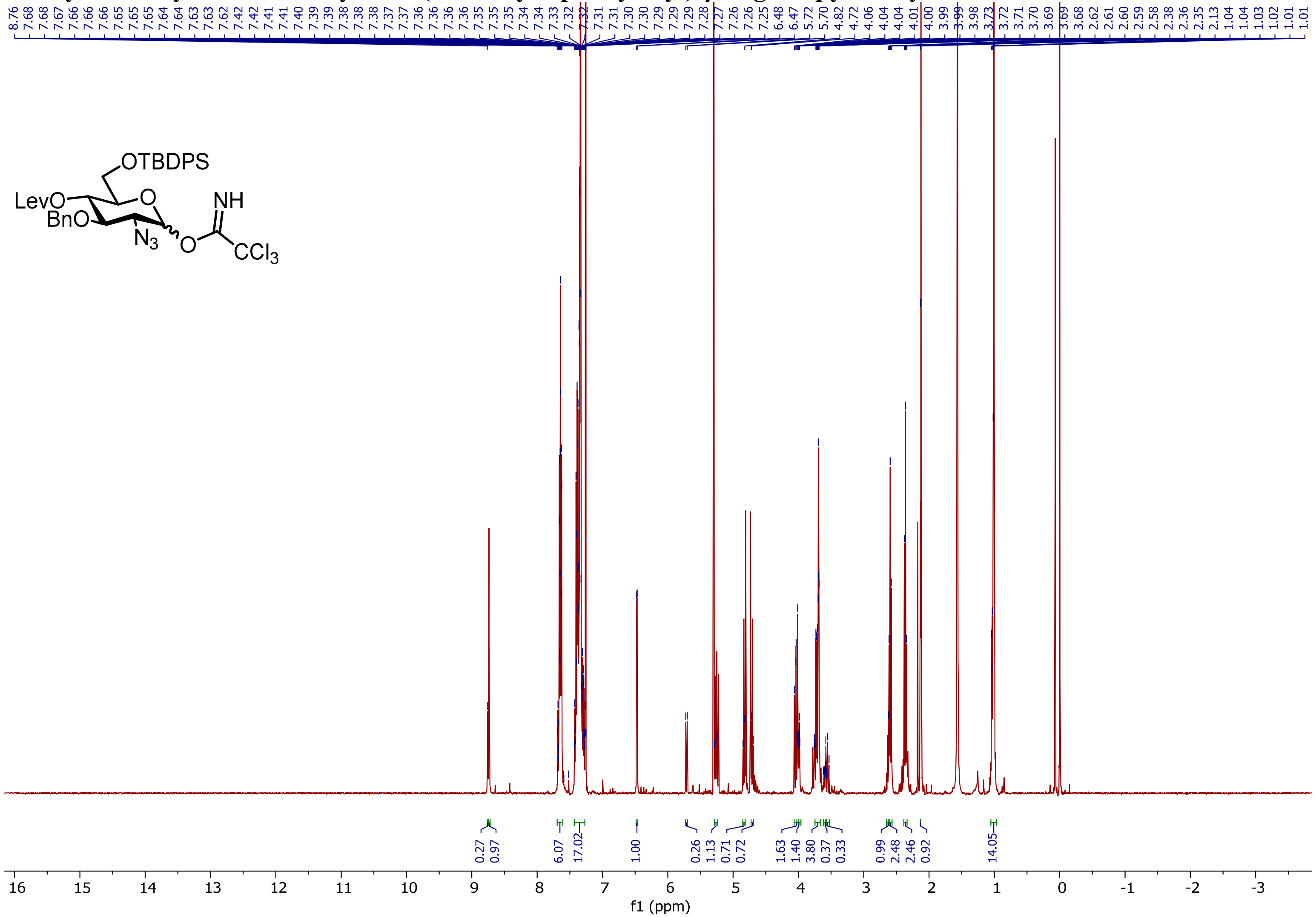

^{1}H NMR (400 MHz, Chloroform-*d*)

2-azido-3-*O*-benzyl-2-deoxy-4-*O*-levulinoyl-6-*O*-(*tert*-butyldiphenylsilyl)-β-D-glucopyranosyl trichloroacetamidate 19

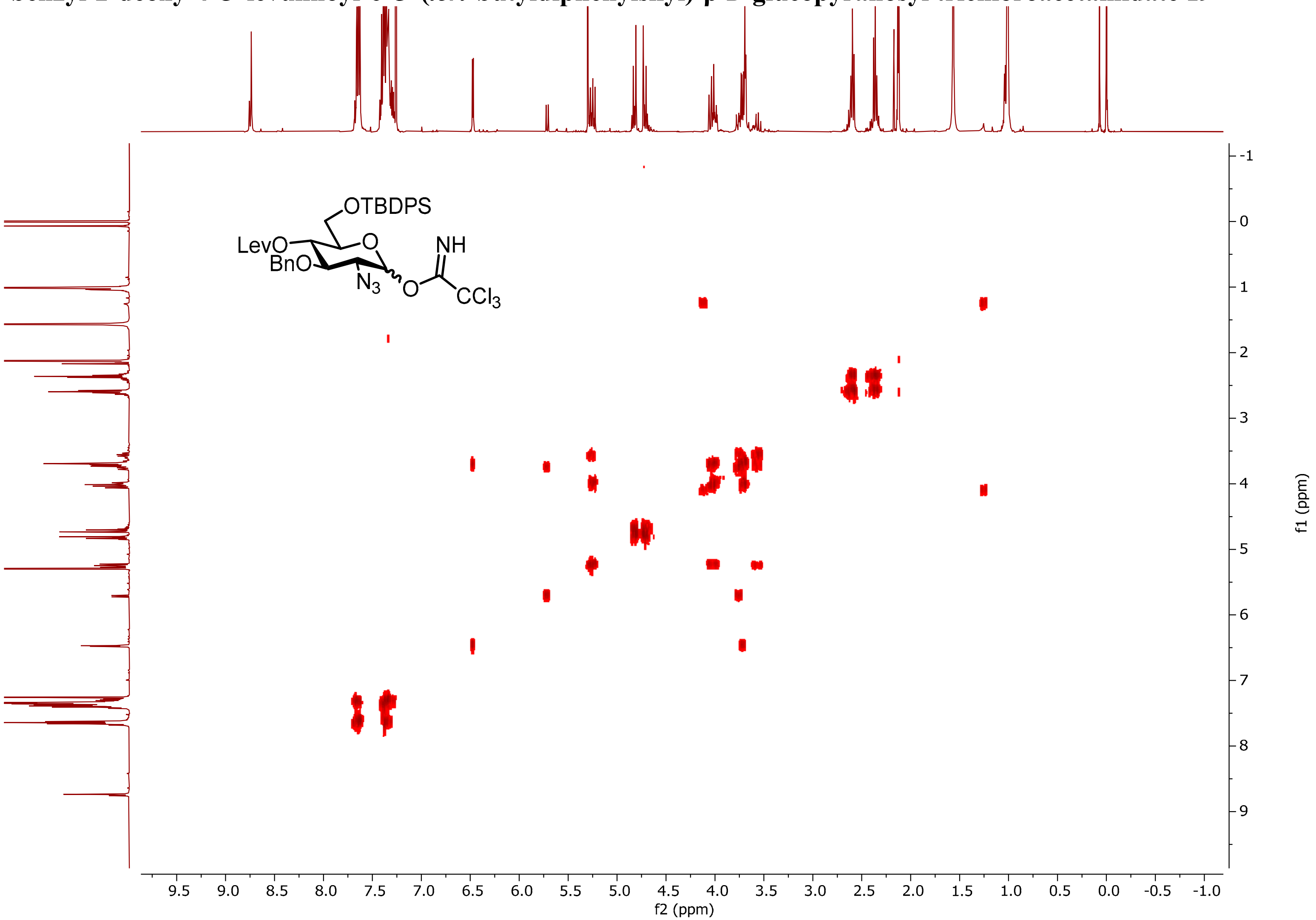

COSY NMR (400 MHz, Chloroform-*d*)

2-azido-3-*O*-benzyl-2-deoxy-4-*O*-levulinoyl-6-*O*-(*tert*-butyldiphenylsilyl)-β-D-glucopyranosyl trichloroacetamidate 19

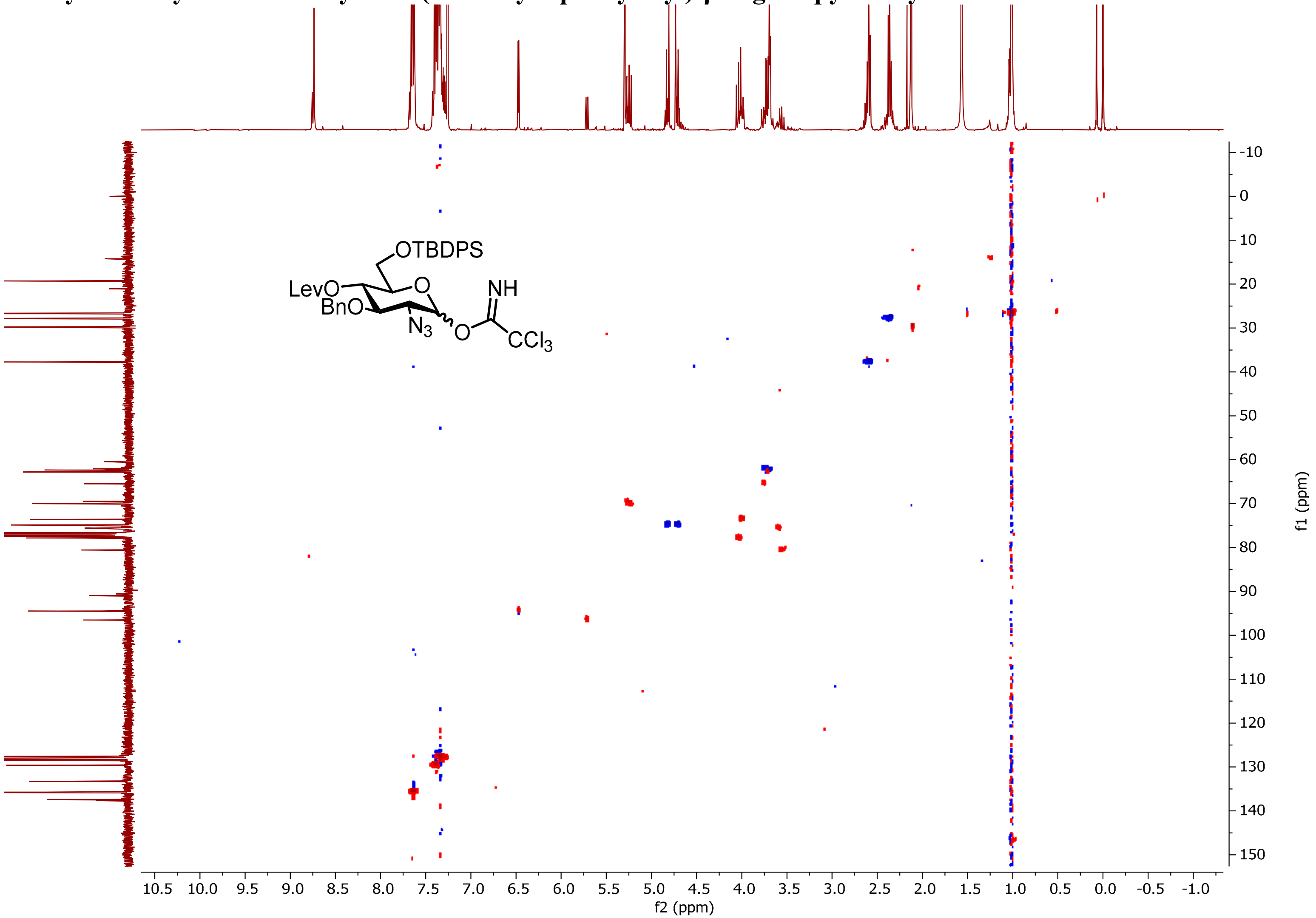

HSQC NMR (400 x 100 MHz, Chloroform-*d*)

2-azido-3-*O*-benzyl-2-deoxy-4-*O*-levulinoyl-6-*O*-(*tert*-butyldiphenylsilyl)-β-D-glucopyranosyl trichloroacetamidate 19

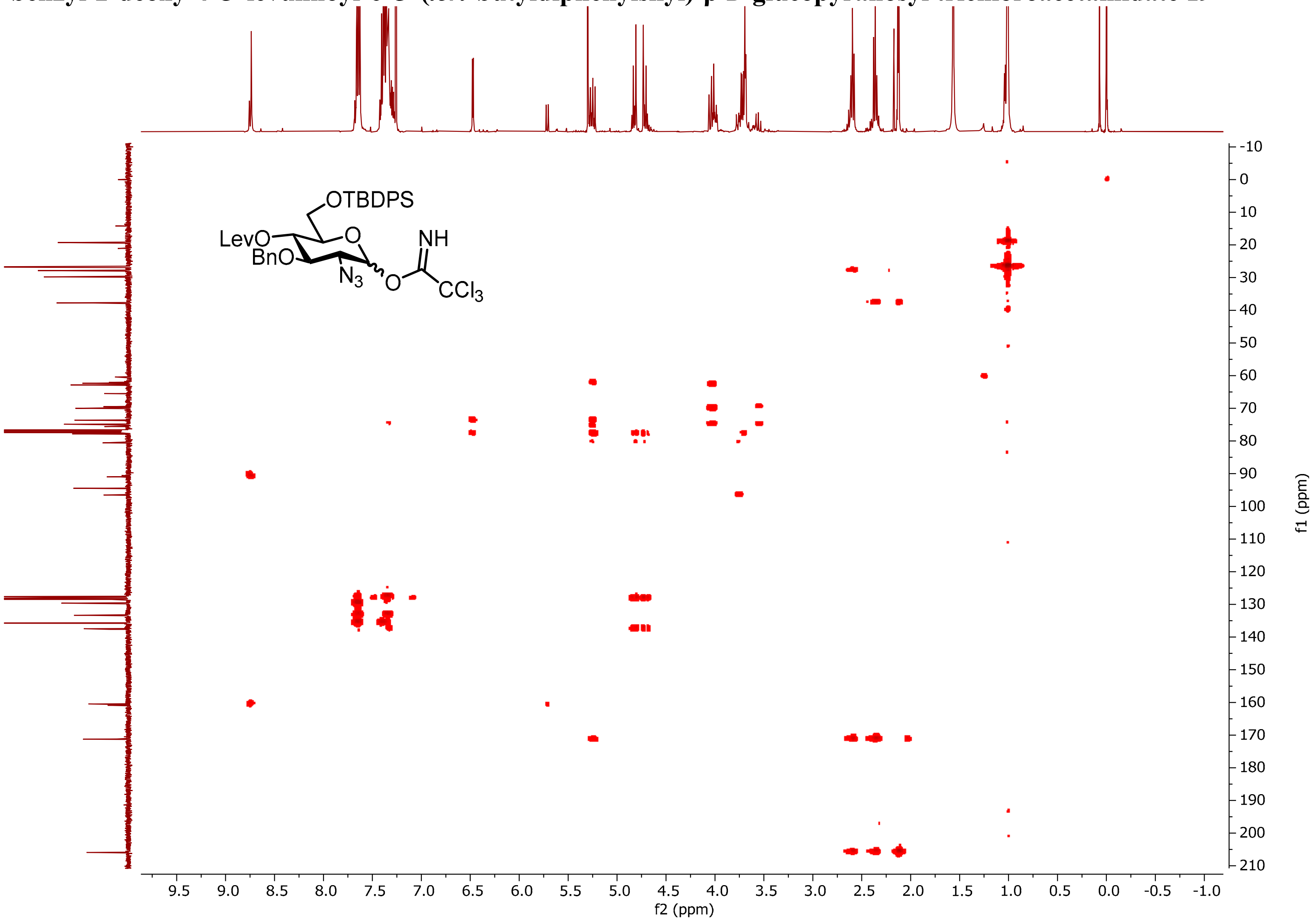

HMBC NMR (400 x 100 MHz, Chloroform-*d*)

2-azido-3-*O*-benzyl-2-deoxy-4-*O*-levulinoyl-6-*O*-(*tert*-butyldiphenylsilyl)-β-D-glucopyranosyl trichloroacetamidate 19

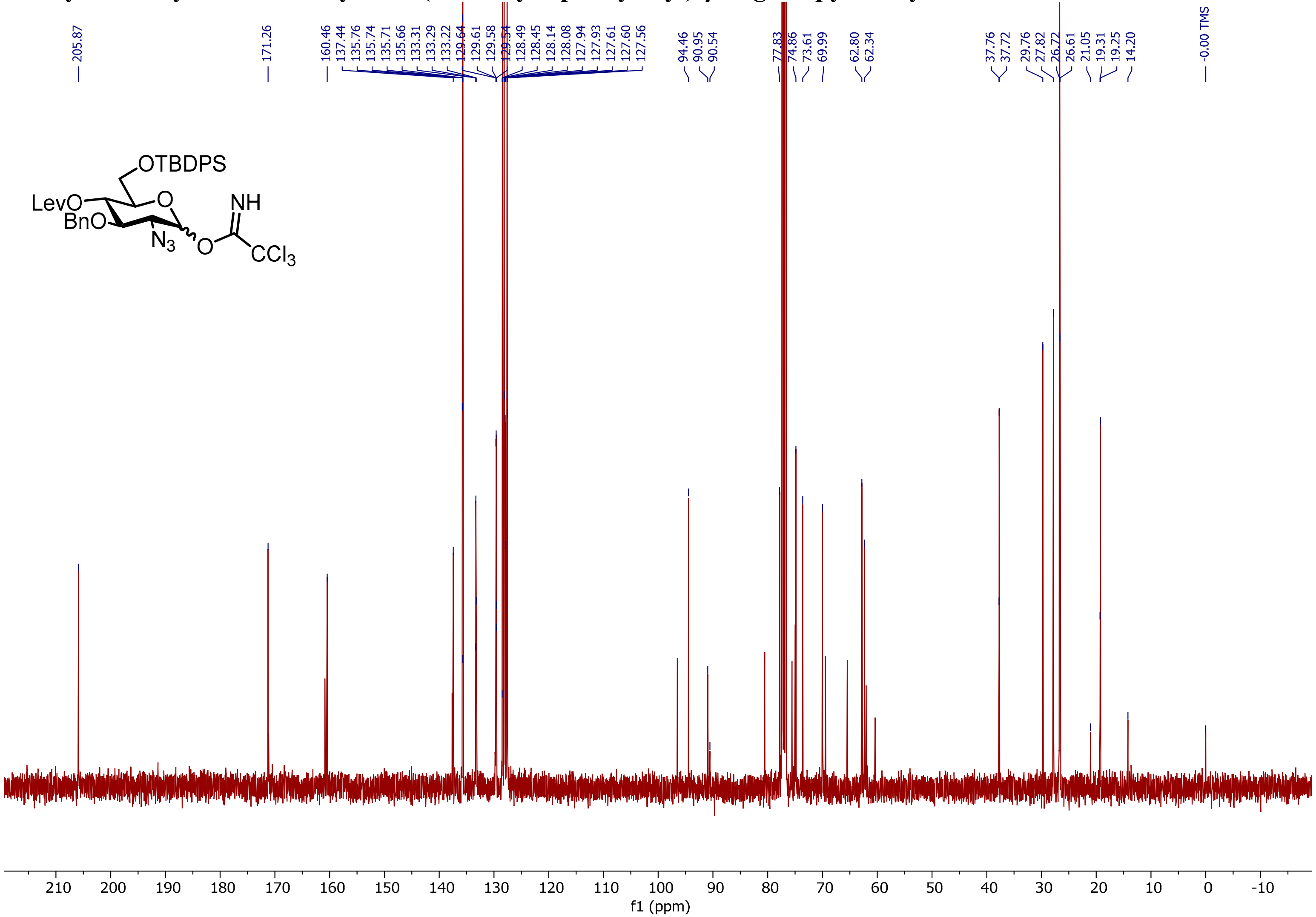

^{13}C NMR (100 MHz, Chloroform-*d*)

Side Products Obtained From Attempted *S*-glycosylations

Bis (Methyl (*p*-methoxyphenyl 2,3-di-*O*-benzoyl-4-thio-β-D-glucopyranosid)uronate)-4,4'-disulfide 20

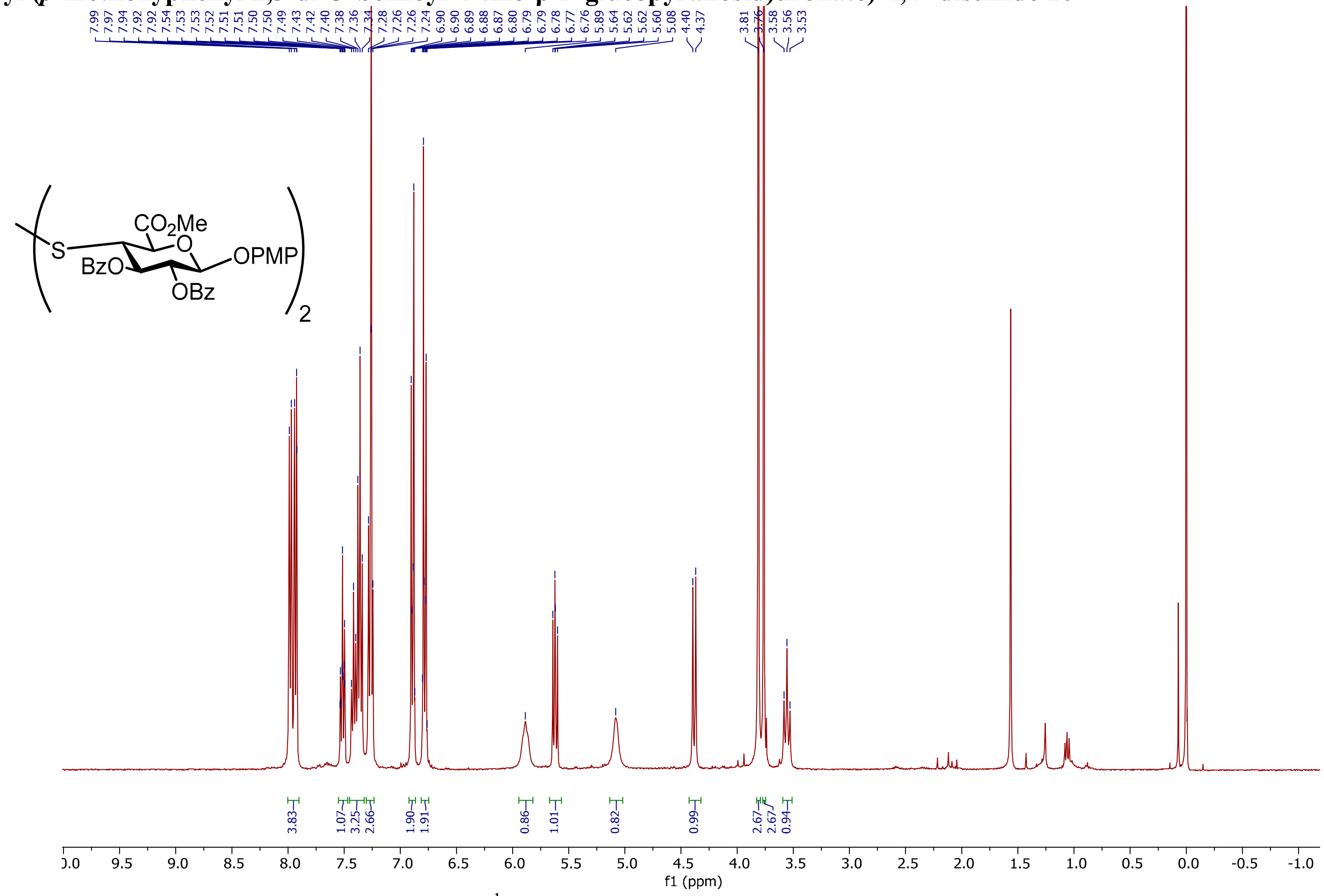

^{1}H NMR (400 MHz, Chloroform-*d*)

Bis (Methyl (*p*-methoxyphenyl 2,3-di-*O*-benzoyl-4-thio-β-D-glucopyranosid)uronate)-4,4'-disulfide 20

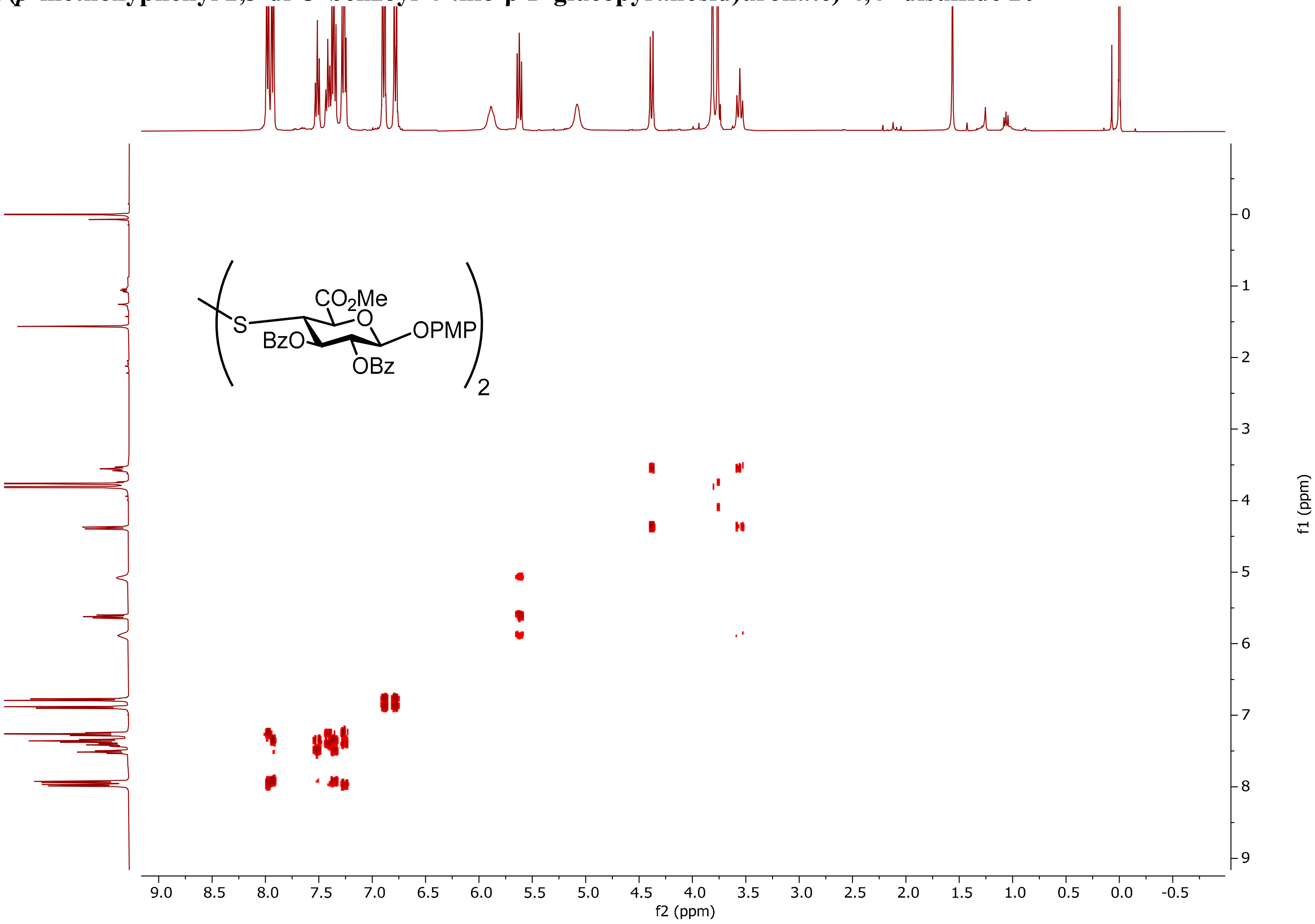

COSY NMR (400 MHz, Chloroform-*d*)

Bis (Methyl (*p*-methoxyphenyl 2,3-di-*O*-benzoyl-4-thio-β-D-glucopyranosid)uronate)-4,4'-disulfide 20

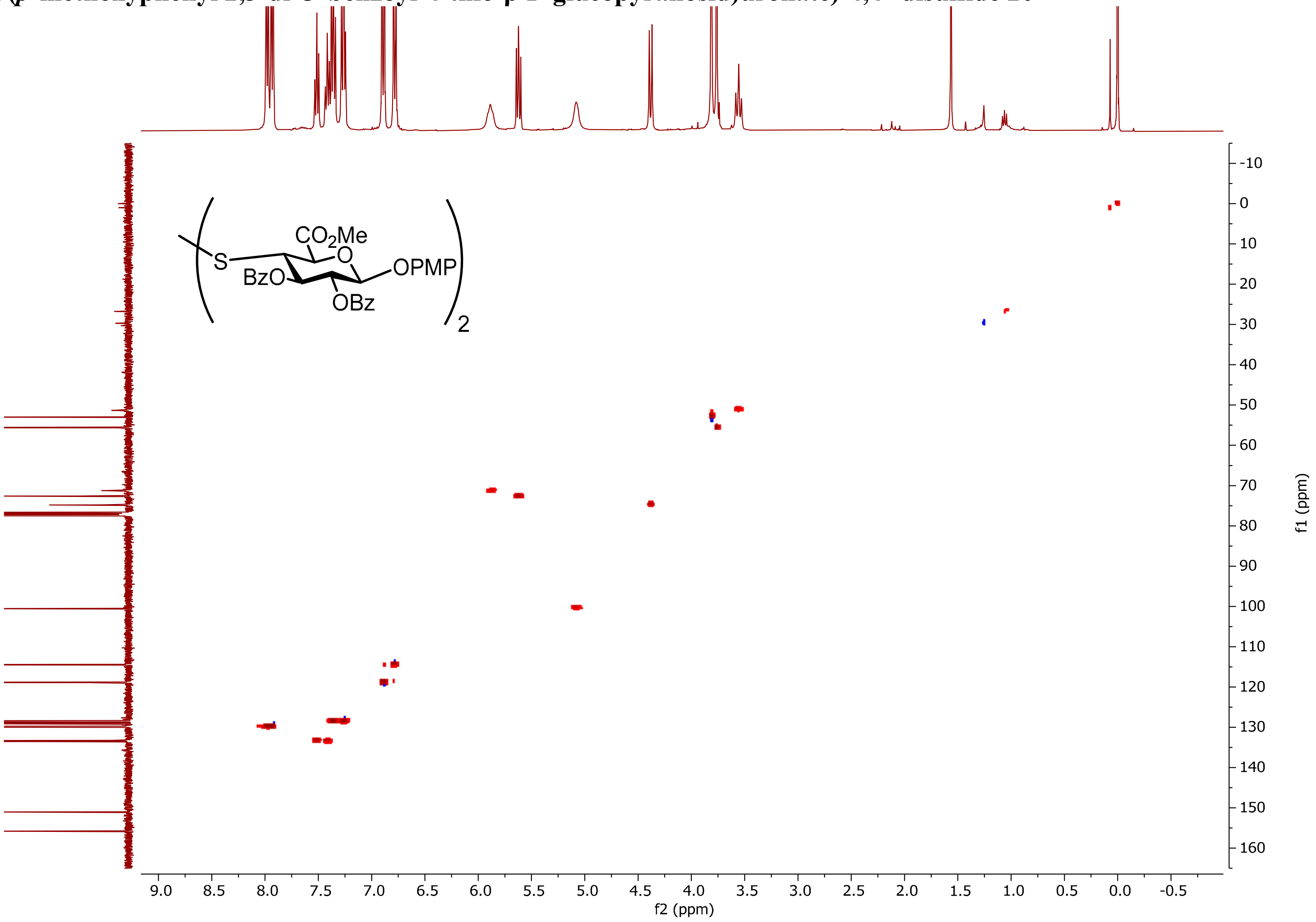

HSQC NMR (400 x 100 MHz, Chloroform-*d*)

Bis (Methyl (*p*-methoxyphenyl 2,3-di-*O*-benzoyl-4-thio-β-D-glucopyranosid)uronate)-4,4'-disulfide 20

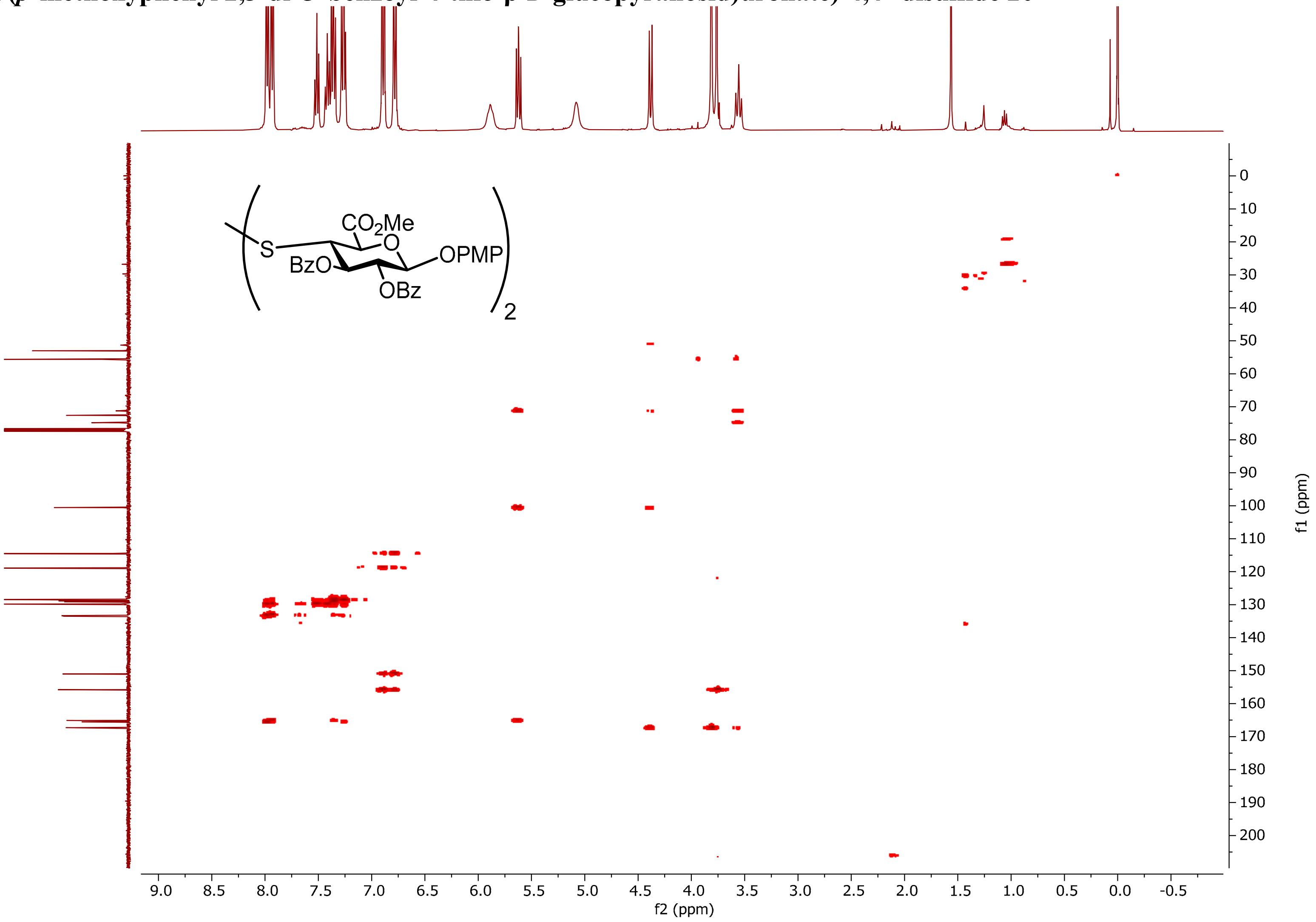

HMBC NMR (400 x 100 MHz, Chloroform-*d*)

Bis (Methyl (*p*-methoxyphenyl 2,3-di-*O*-benzoyl-4-thio-β-D-glucopyranosid)uronate)-4,4'-disulfide 20

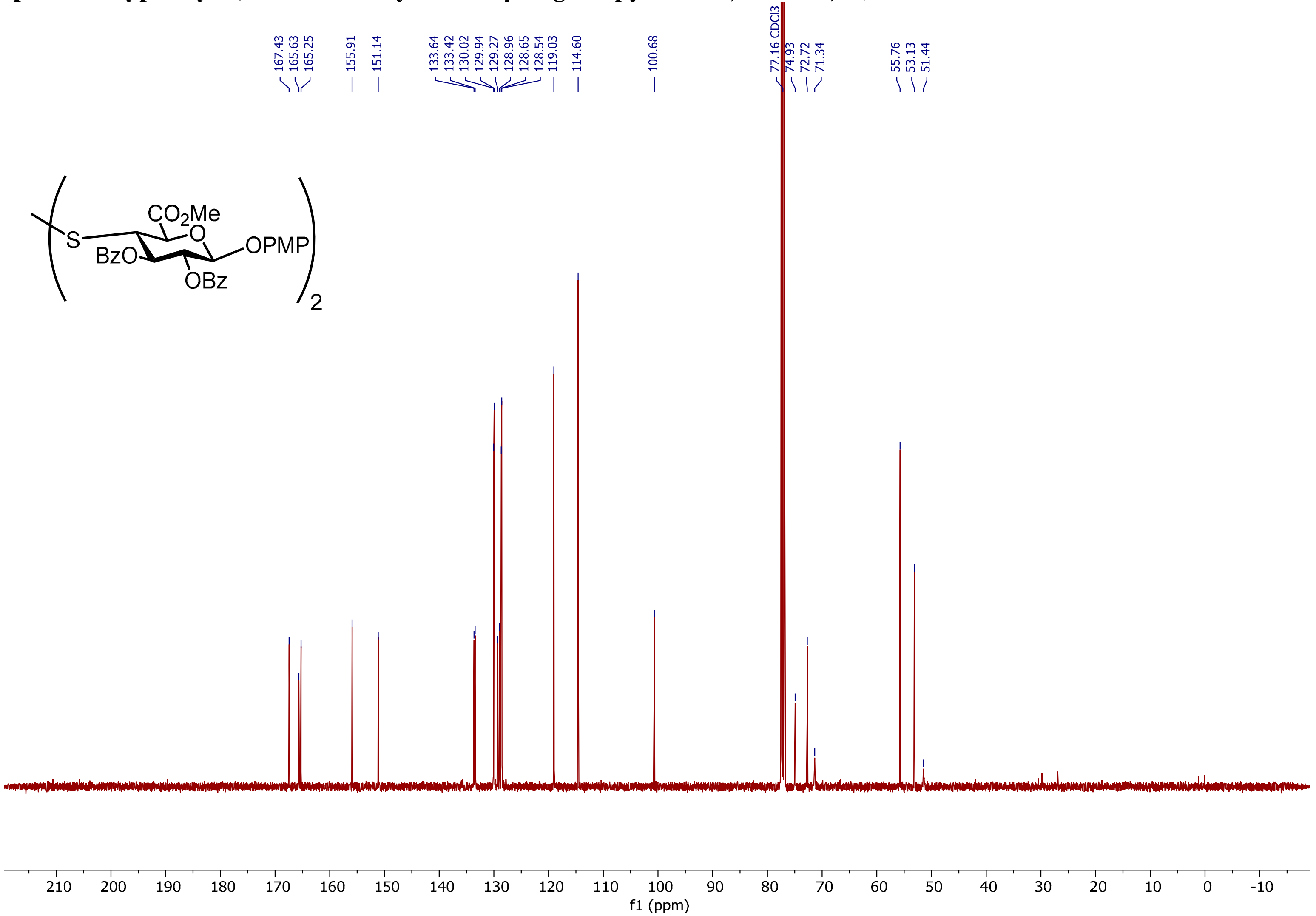

^{13}C NMR (100 MHz, Chloroform-*d*)

_p_-Methoxyphenyl 2,6-_O_-dibenzoyl-3-_O_-benzyl-4-thio-β-D-glucopyranoside 21

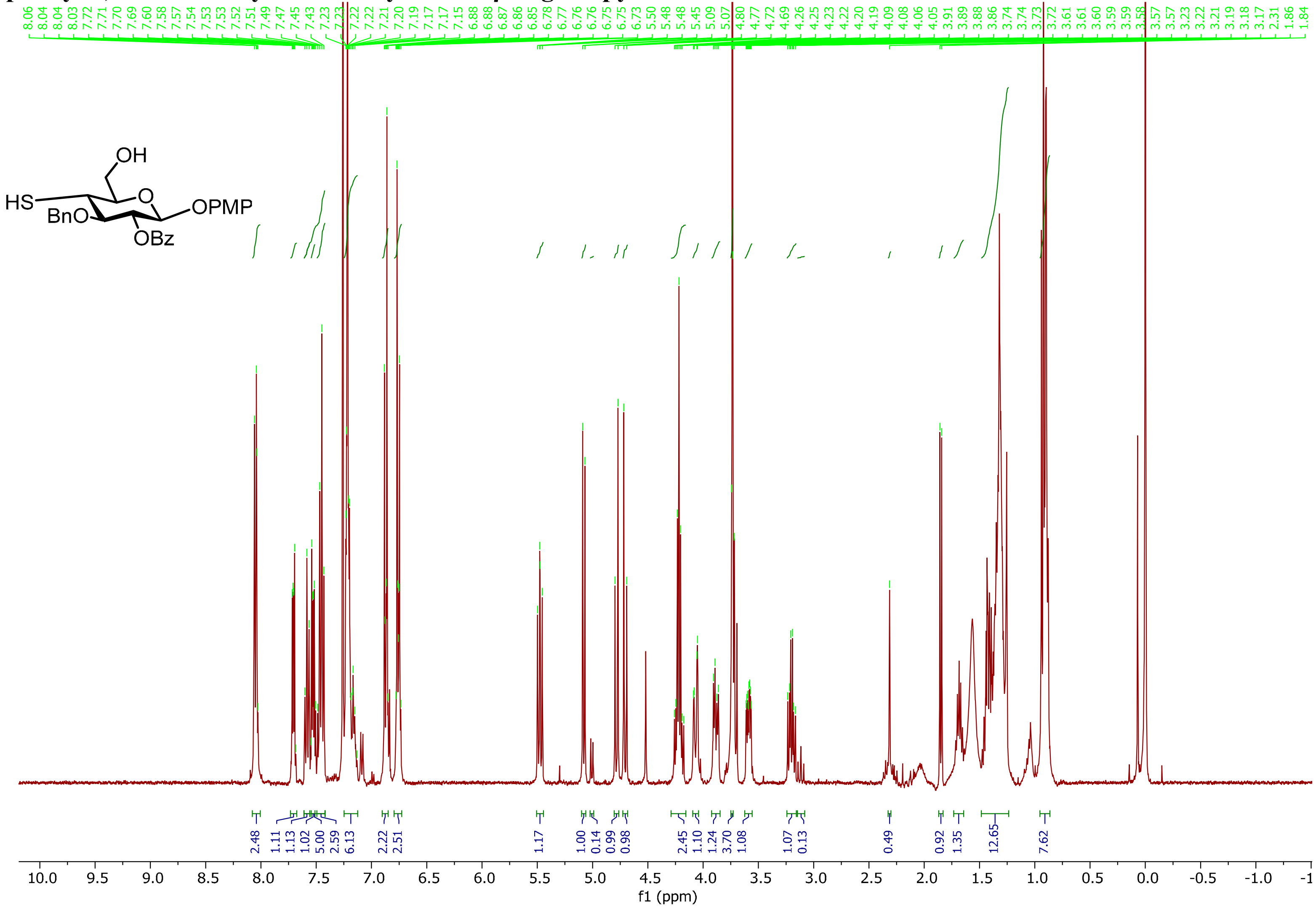

^{1}H NMR (400 MHz, Chloroform-_d_)

_p_-Methoxyphenyl 2,6-_O_-dibenzoyl-3-_O_-benzyl-4-thio-β-D-glucopyranoside 21

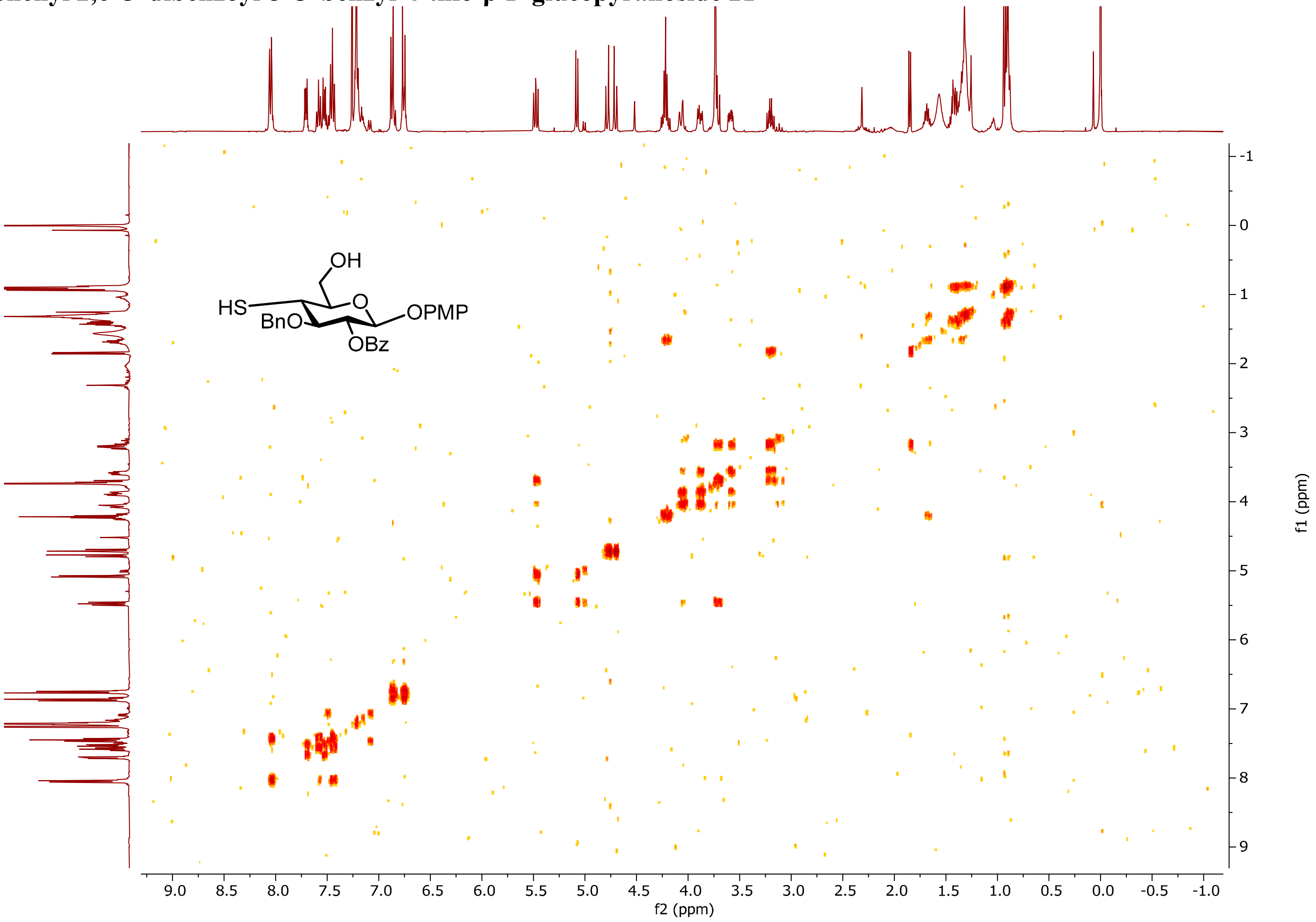

COSY NMR (400 MHz, Chloroform-_d_)

p-Methoxyphenyl 2,6-O-dibenzoyl-3-O-benzyl-4-thio-β-D-glucopyranoside 21

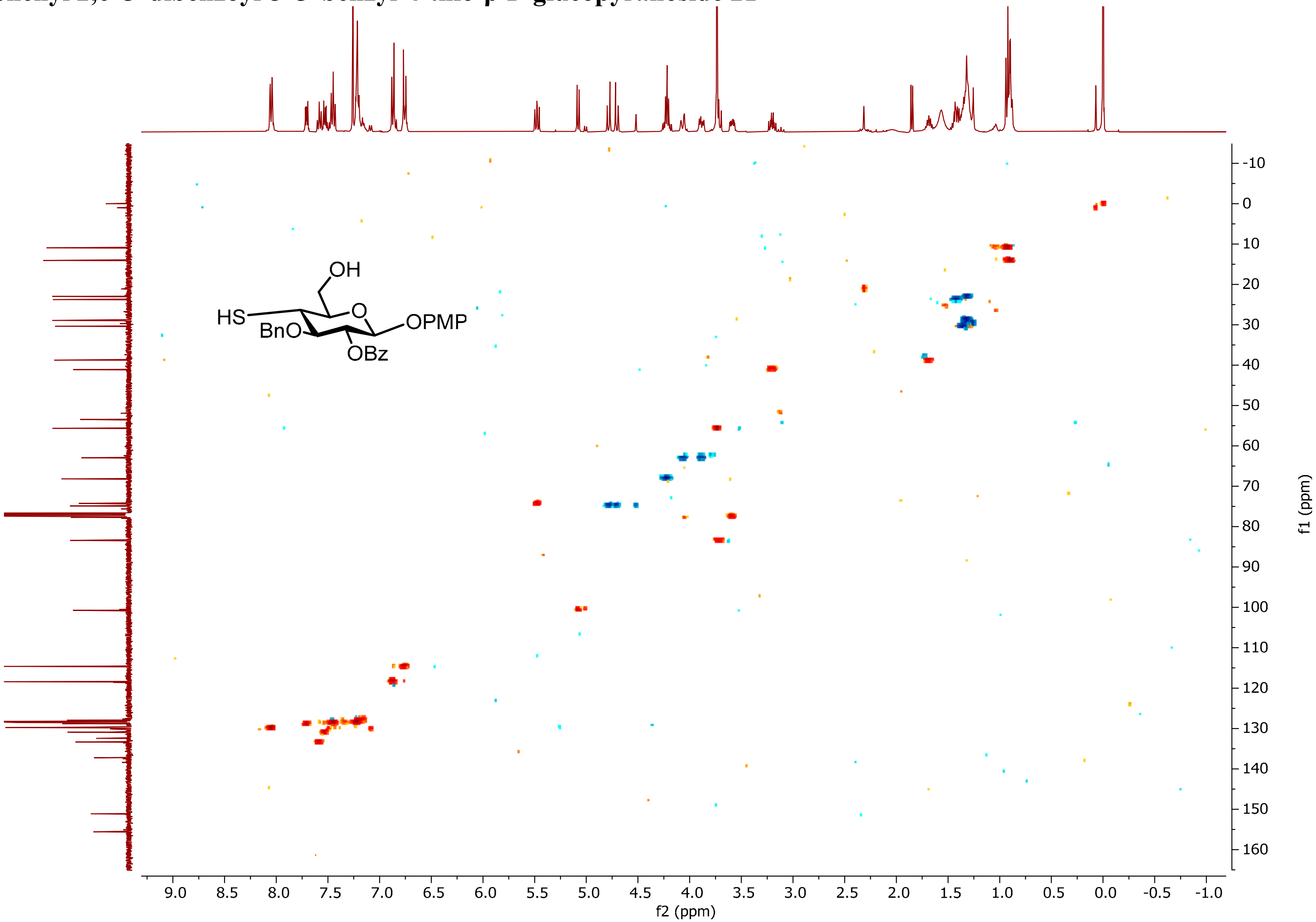

HSQC NMR (400 x 100 MHz, Chloroform-*d*)

p-Methoxyphenyl 2,6-O-dibenzoyl-3-O-benzyl-4-thio-β-D-glucopyranoside 21

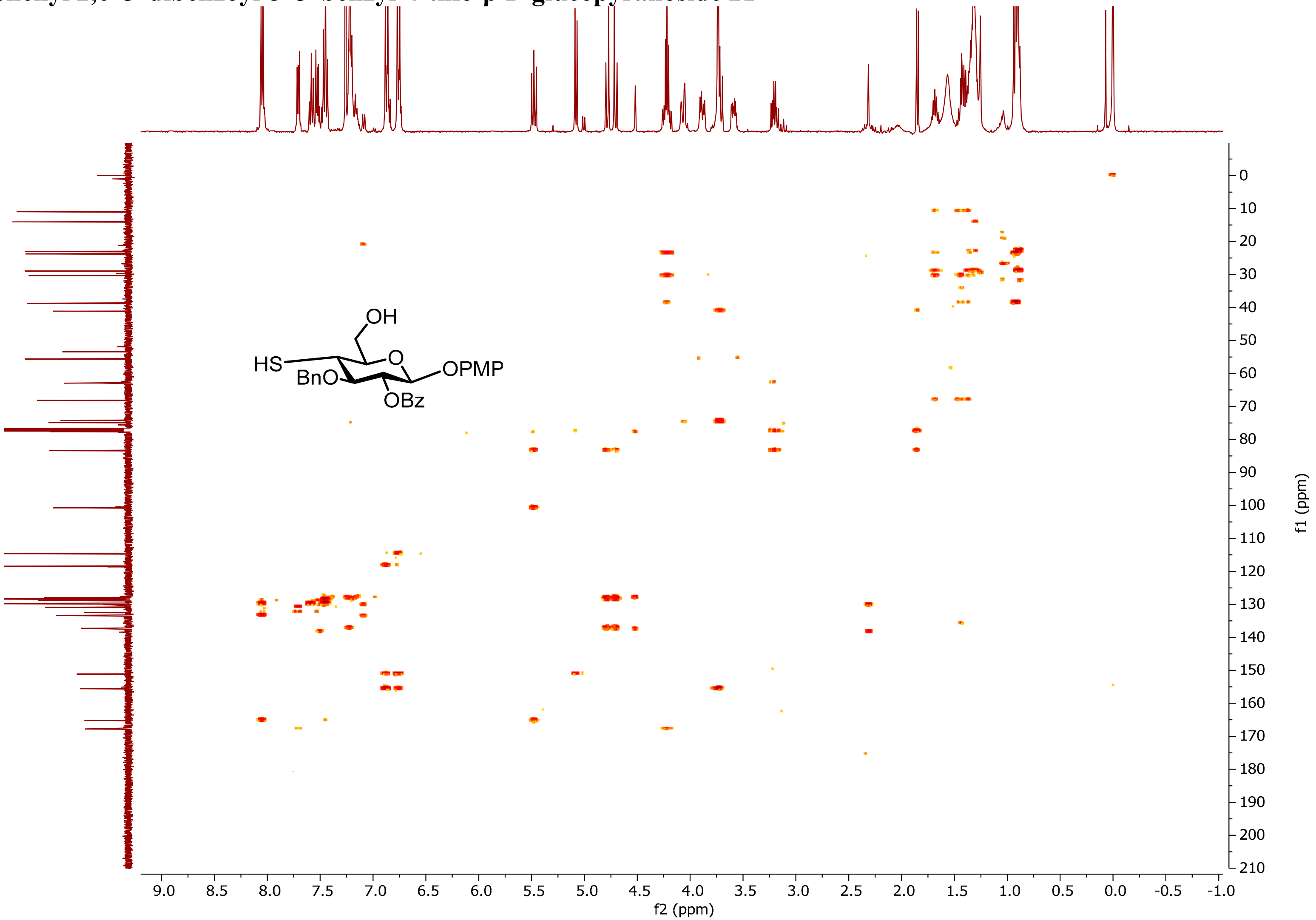

HMBC NMR (400 x 100 MHz, Chloroform-*d*)

***p*-Methoxyphenyl 2,6-*O*-dibenzoyl-3-*O*-benzyl-4-thio-β-D-glucopyranoside 21**

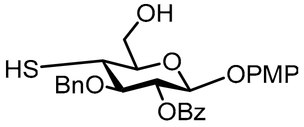

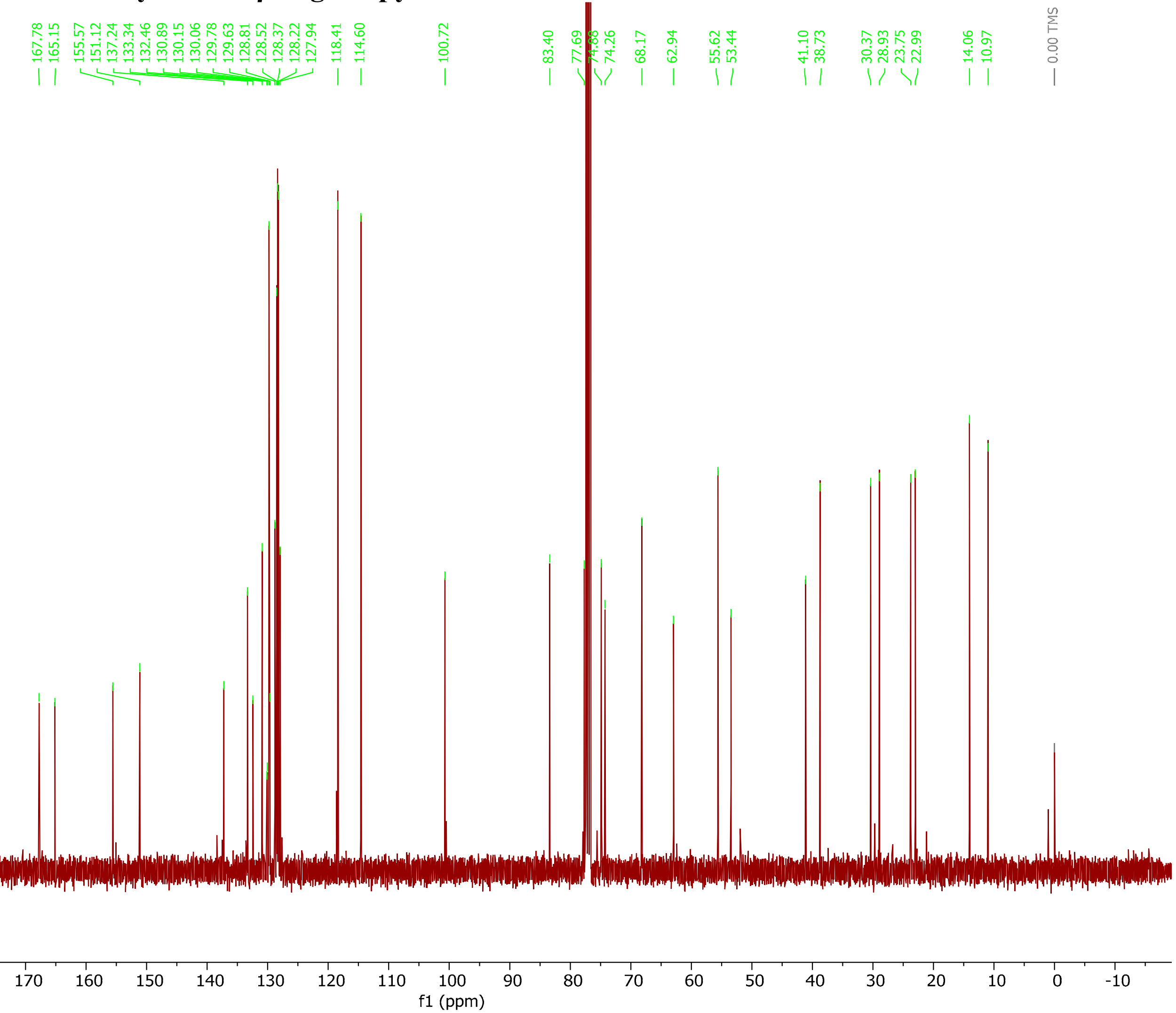

^{13}C NMR (100 MHz, Chloroform-*d*)

Synthesis of Anomeric Thiols and Thioacetates

3,4,6-Tri-*O*-acetyl-1,2-di-deoxy-2'-azidomethyl-α-D-glucopyrano-[2,1-d]-Δ2'-thiaoxazoline 24

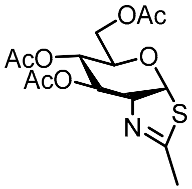

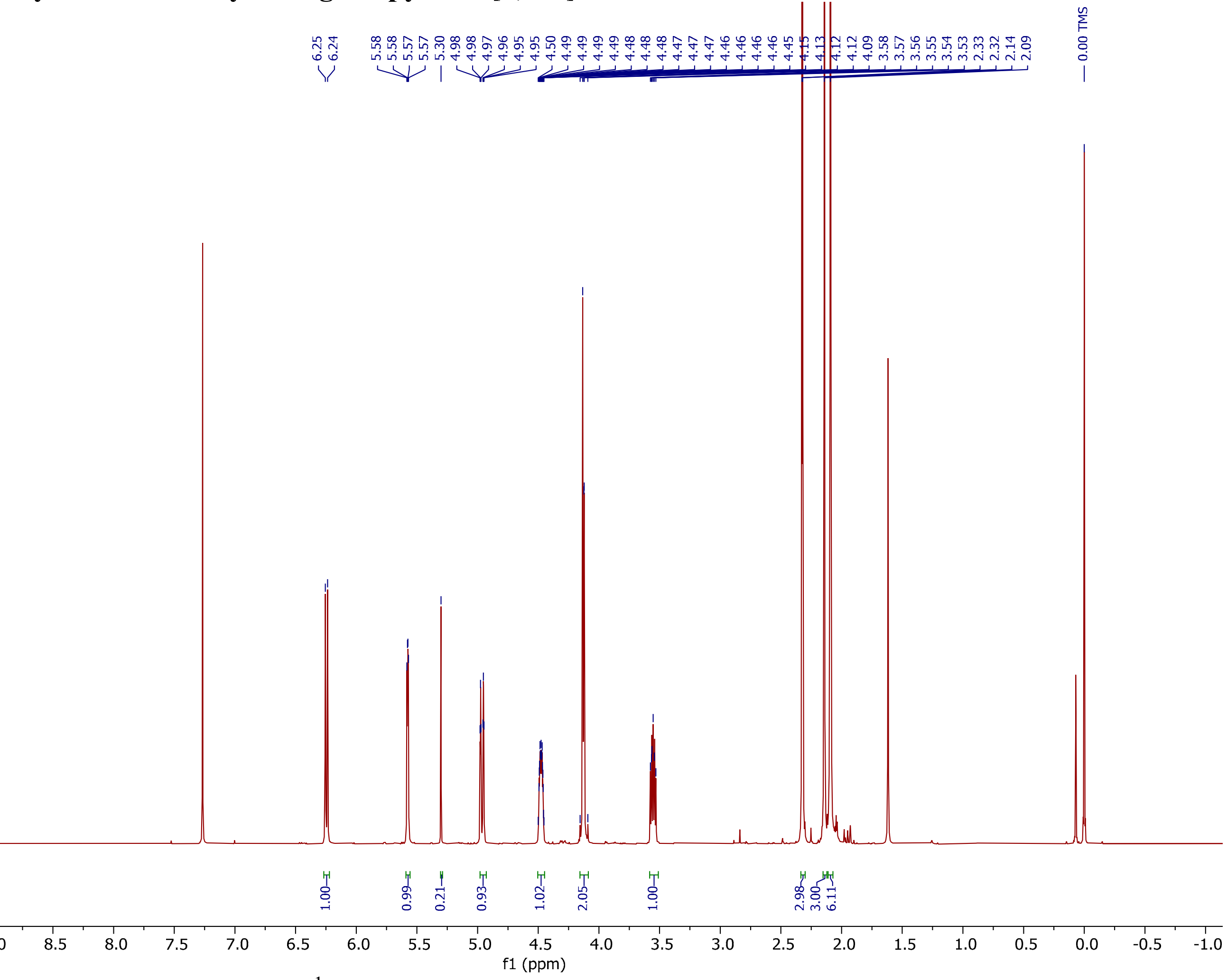

^{1}H NMR (400 MHz, Chloroform-*d*)

3,4,6-Tri-*O*-acetyl-1,2-di-deoxy-2'-azidomethyl-α-D-glucopyrano-[2,1-d]-Δ2'-thiaoxazoline 24

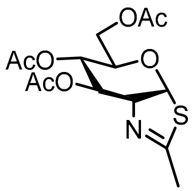

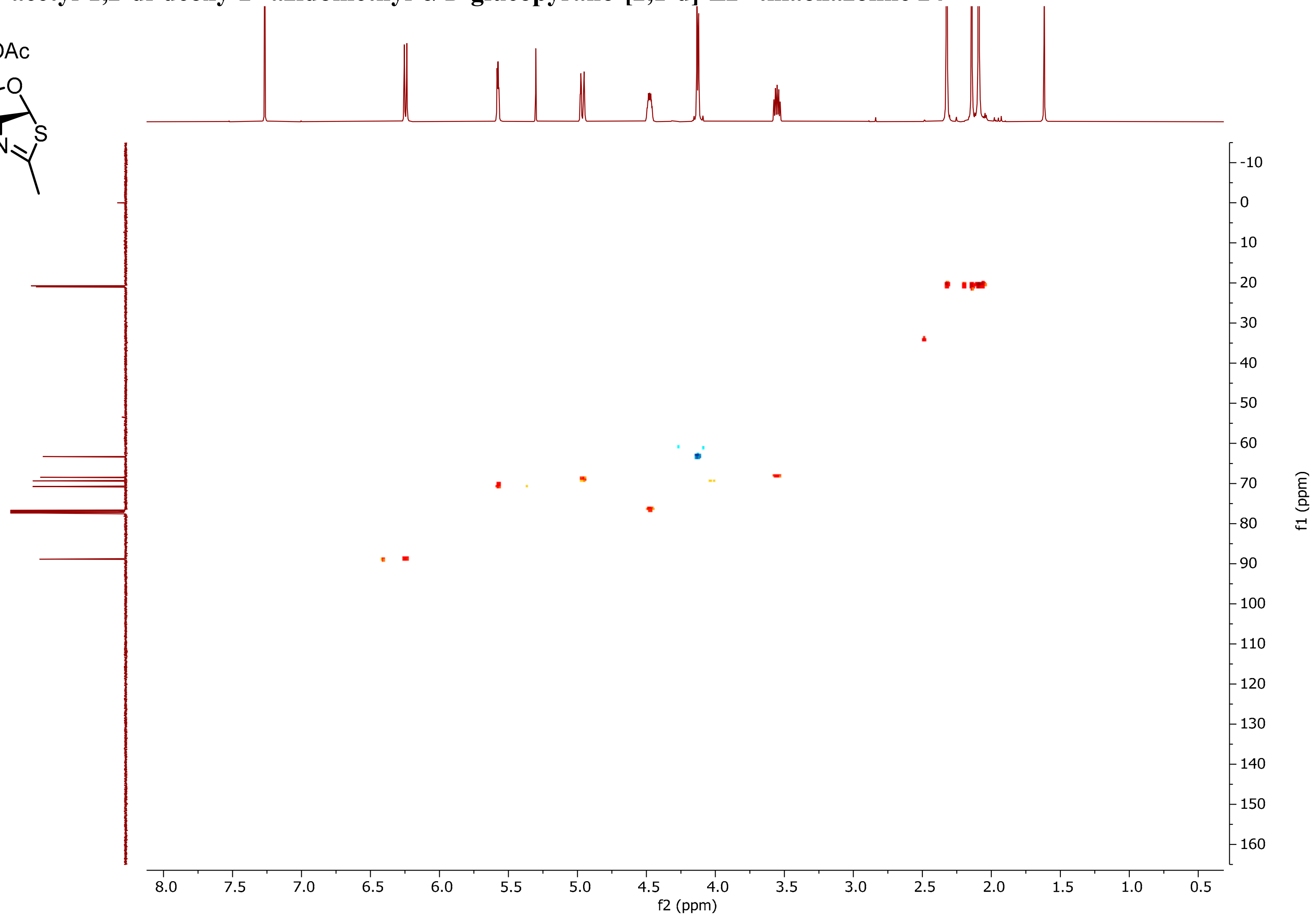

HSQC (400 × 100 MHz, Chloroform-*d*)

3,4,6-Tri-*O*-acetyl-1,2-di-deoxy-2'-azidomethyl-α-D-glucopyrano-[2,1-d]-Δ2'-thiaoxazoline 24

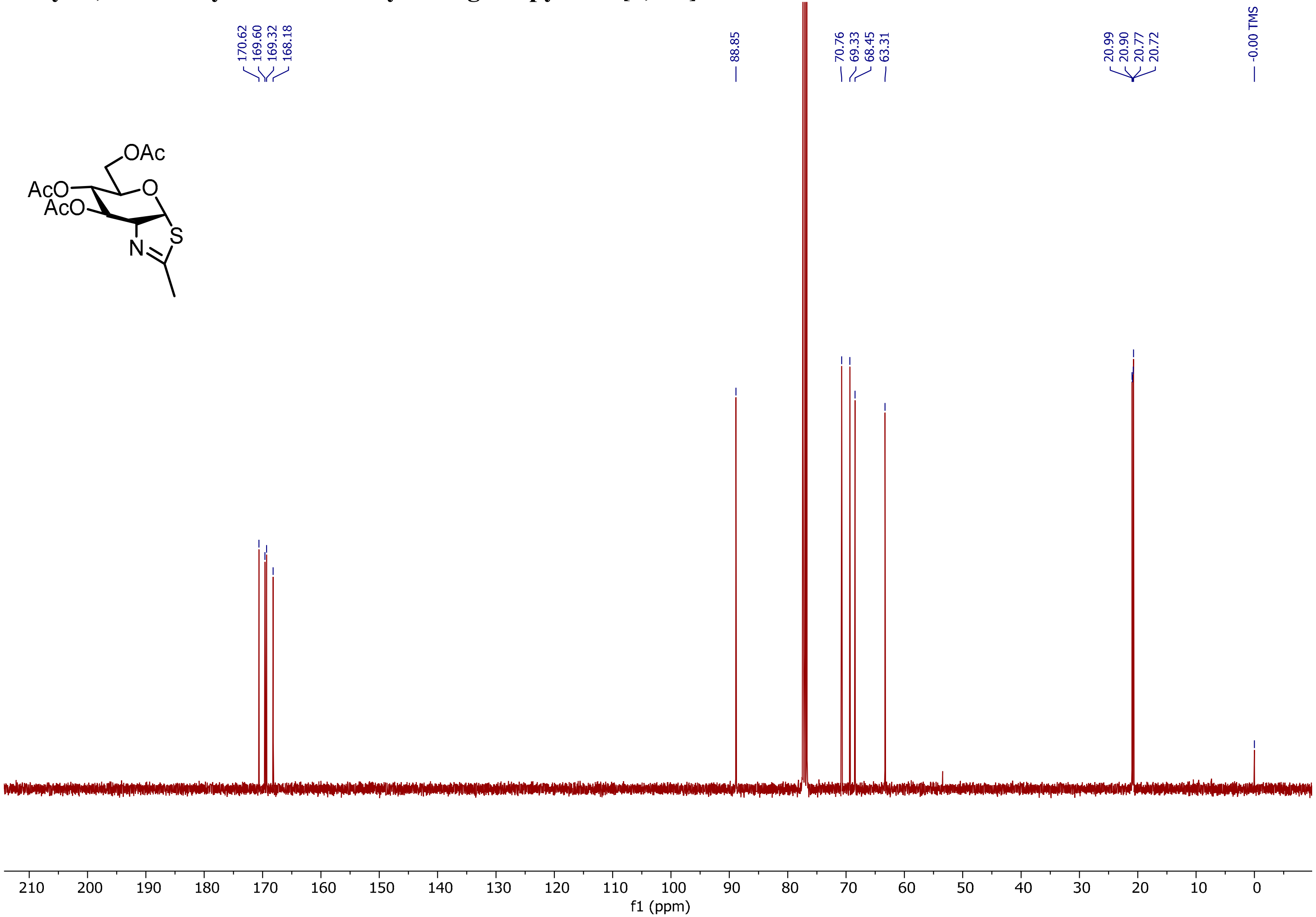

^{13}C NMR (100 MHz, Chloroform-*d*)

2-Acetamido-3,4,6-tri-*O*-acetyl-2-deoxy-1-thio-α-D-glucopyranose 25

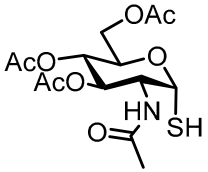

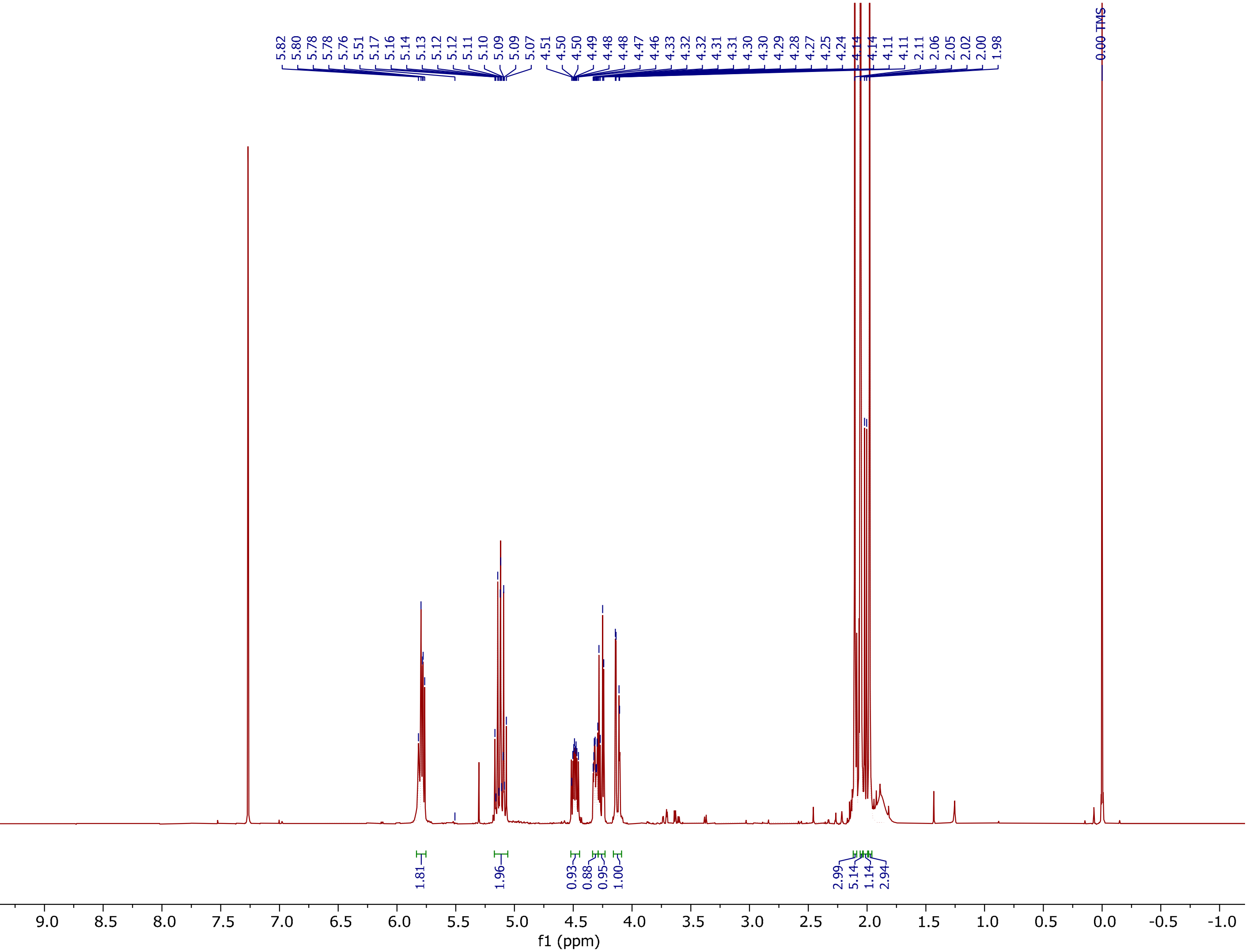

^{1}H NMR (400 MHz, Chloroform-*d*)

2-Acetamido-3,4,6-tri-*O*-acetyl-2-deoxy-1-thio-α-D-glucopyranose 25

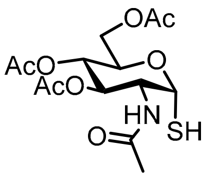

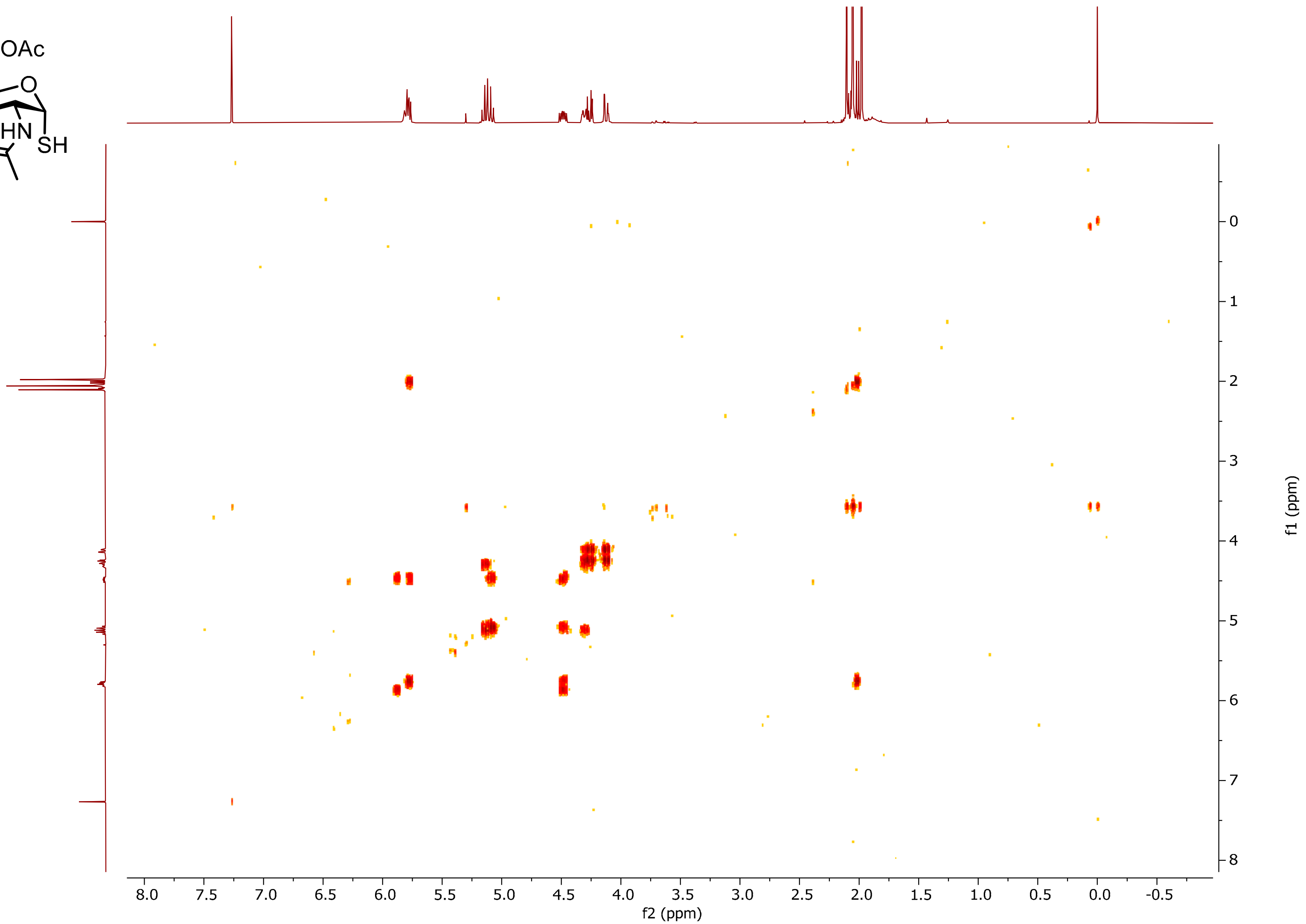

COSY (400 × 400 MHz, Chloroform-*d*)

2-Acetamido-3,4,6-tri-*O*-acetyl-2-deoxy-1-thio-α-D-glucopyranose 25

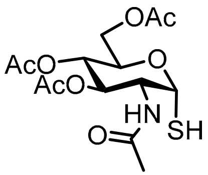

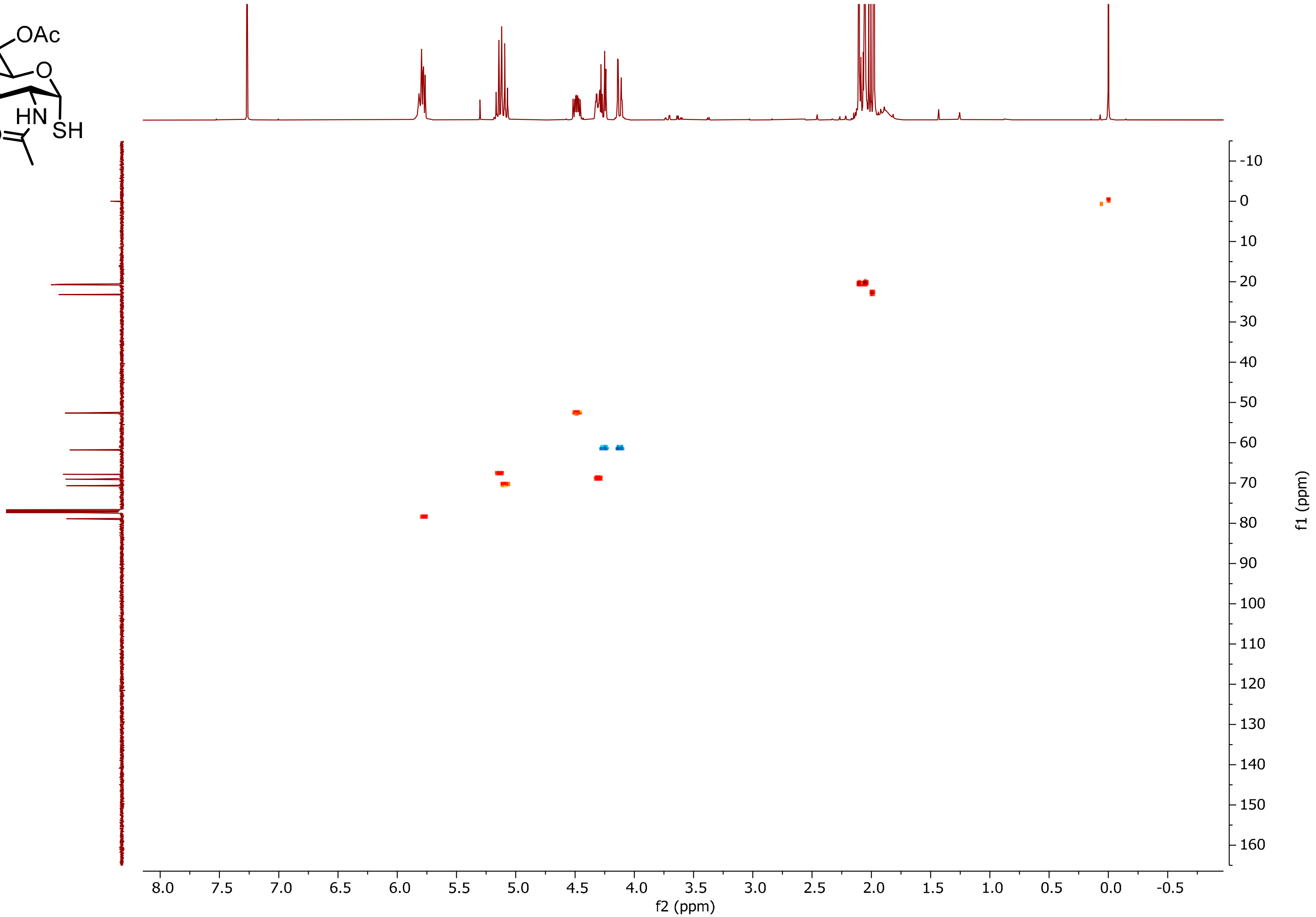

HSQC (400 × 100 MHz, Chloroform-*d*)

2-Acetamido-3,4,6-tri-*O*-acetyl-2-deoxy-1-thio-α-D-glucopyranose 25

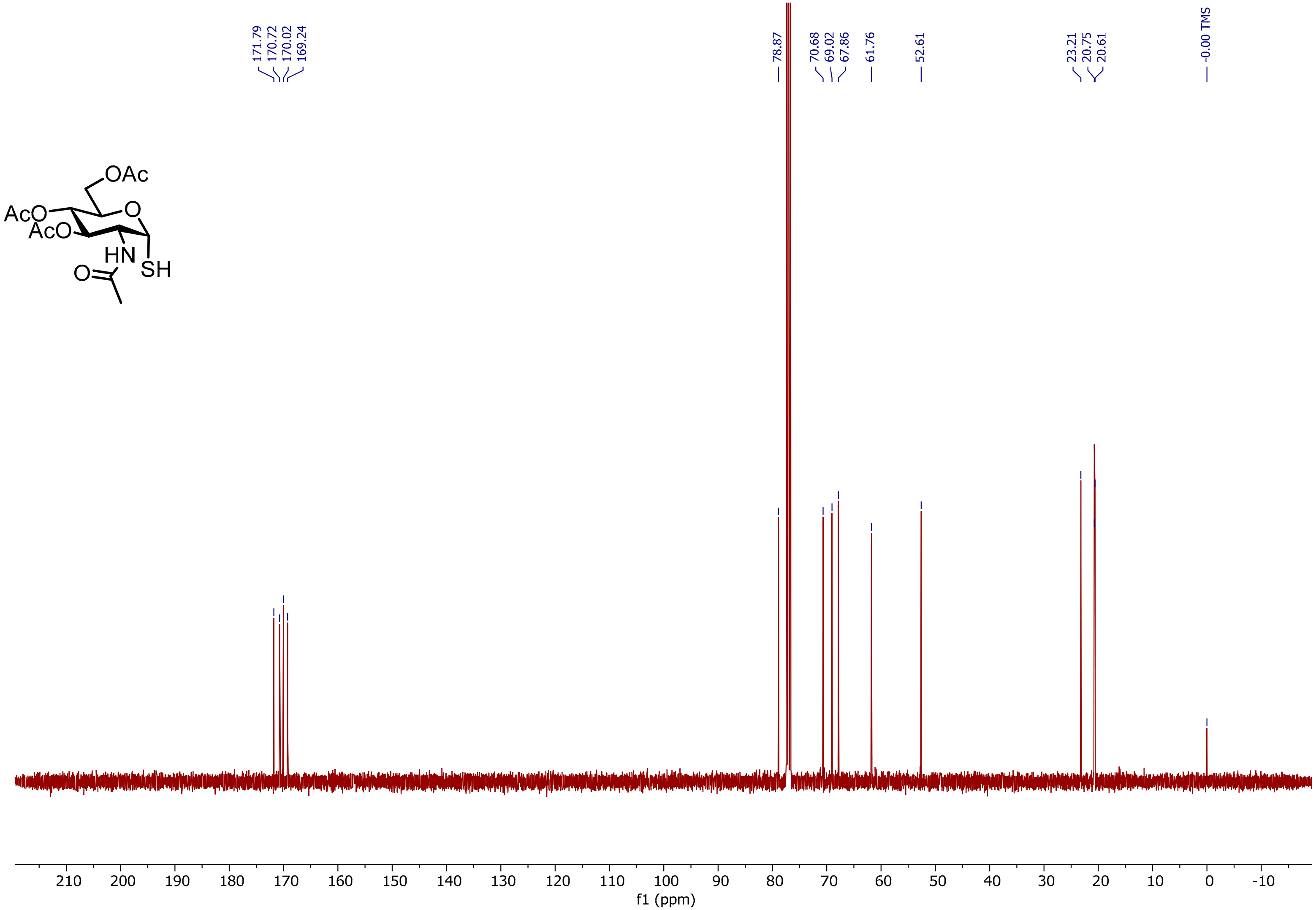

^{13}C NMR (100 MHz, Chloroform-*d*)

p-Monomethoxytrityl 2-acetamido-3,4,6-tetra-O-acetyl-2-deoxy-1-thio-α-D-glucopyranoside 26

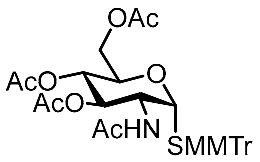

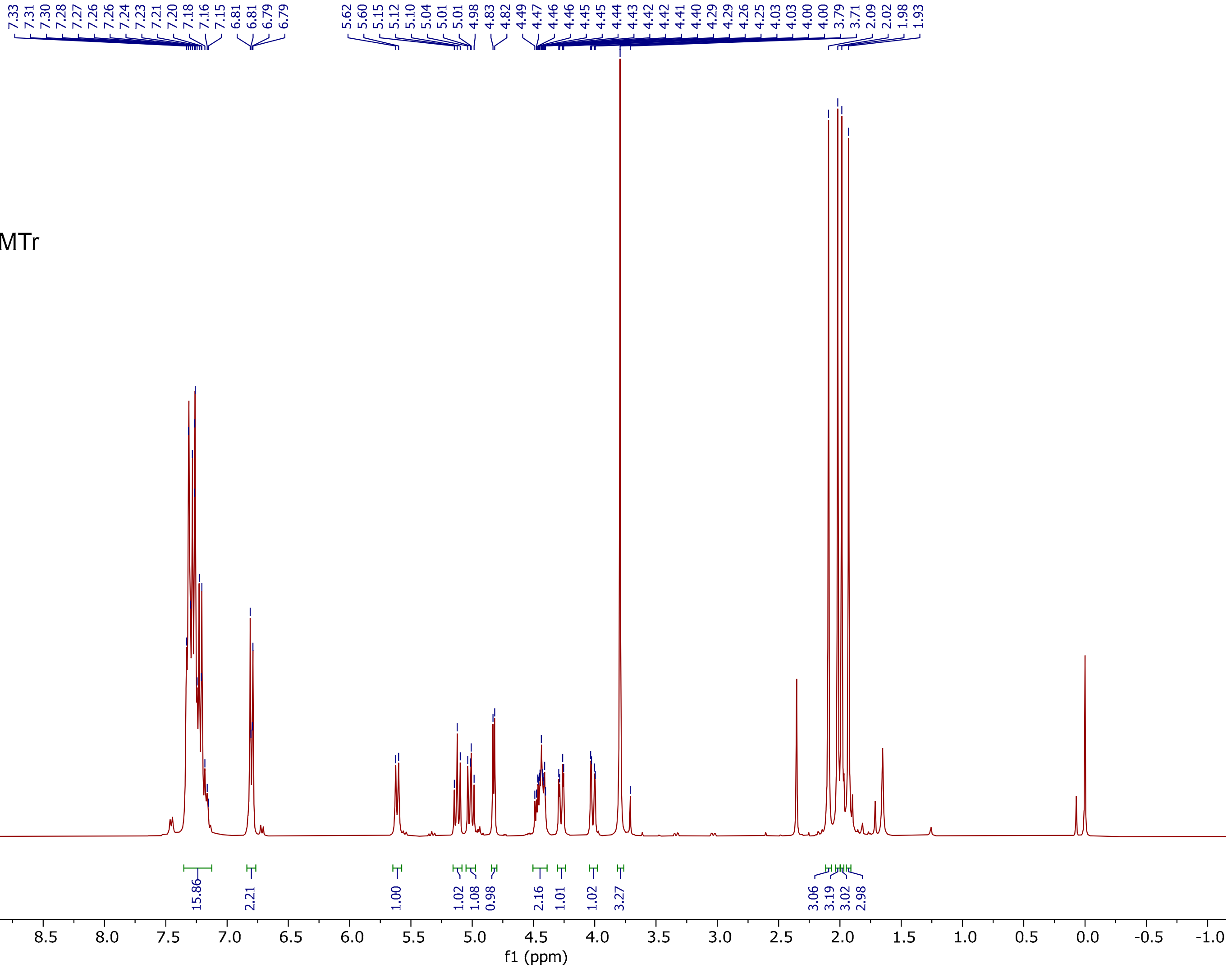

^{1}H NMR (400 MHz, Chloroform-*d*)

_p_-Monomethoxytrityl 2-acetamido-3,4,6-tetra-_O_-acetyl-2-deoxy-1-thio-α-D-glucopyranoside 26

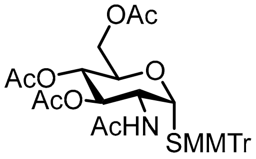

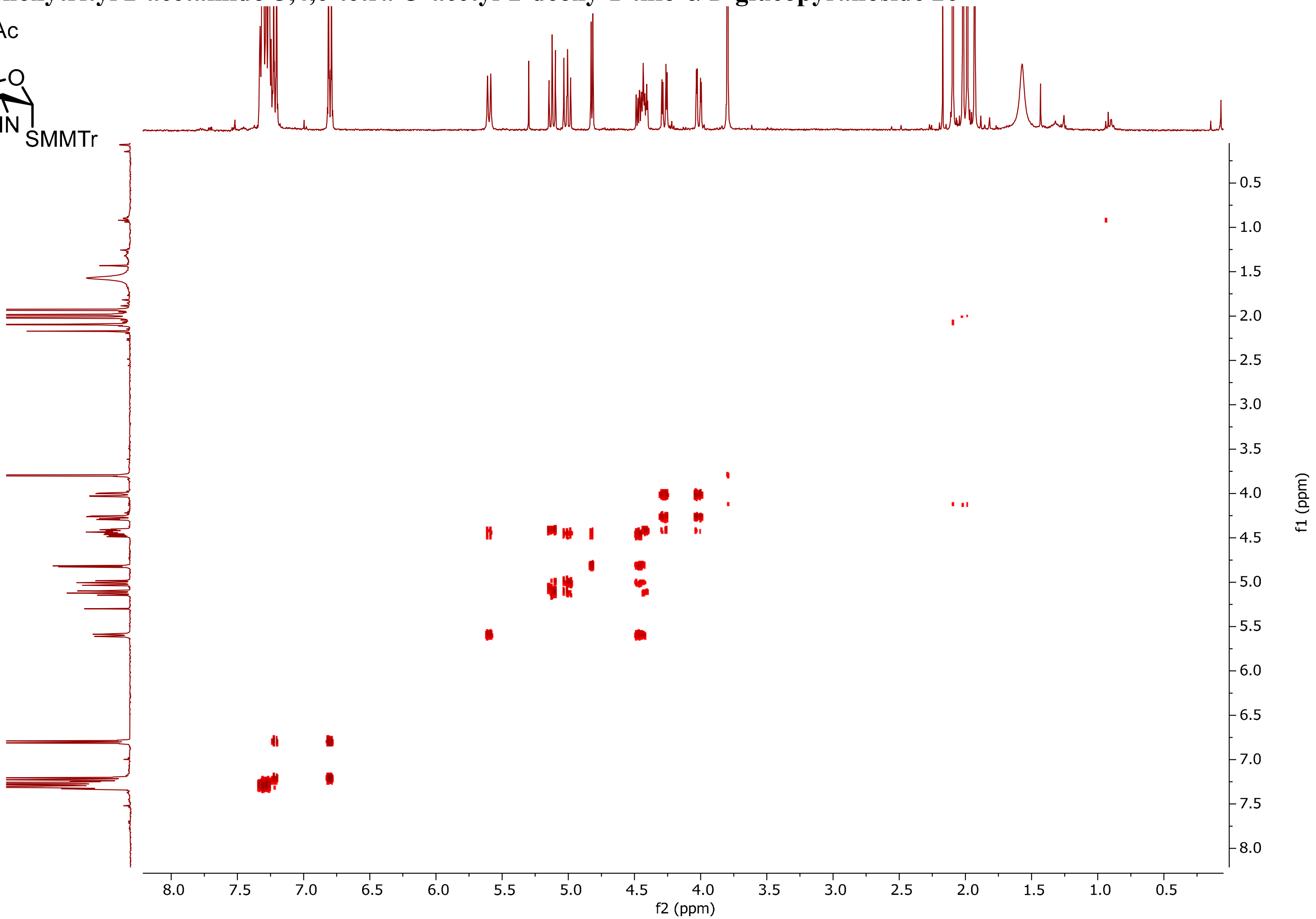

COSY (400 × 400 MHz, Chloroform-_d_)

p-Monomethoxytrityl 2-acetamido-3,4,6-tetra-*O*-acetyl-2-deoxy-1-thio-α-D-glucopyranoside 26

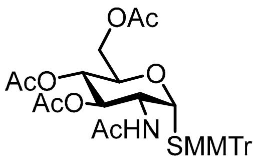

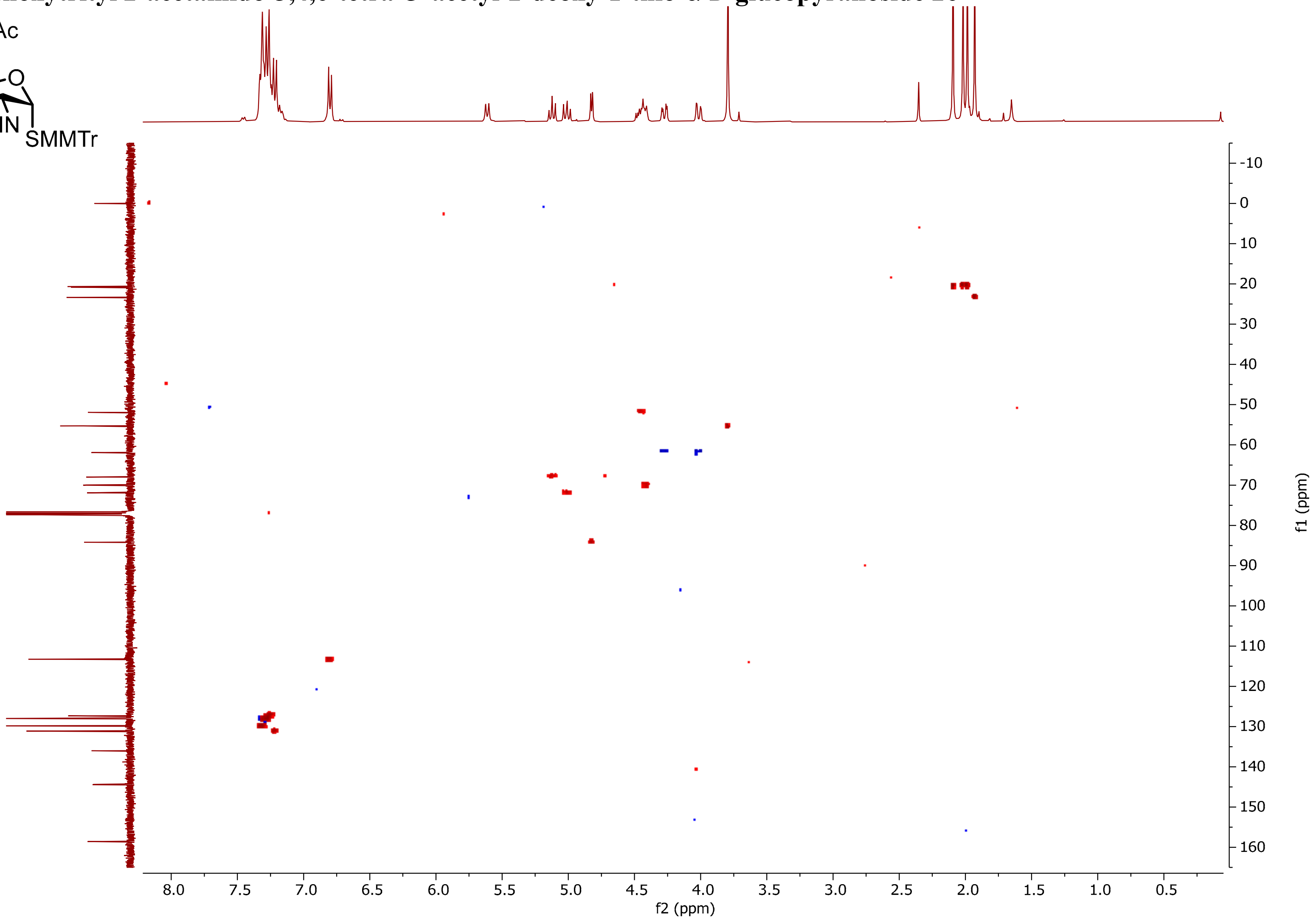

HSQC (400 × 100 MHz, Chloroform-*d*)

_p_-Monomethoxytrityl 2-acetamido-3,4,6-tetra-_O_-acetyl-2-deoxy-1-thio-α-D-glucopyranoside 26

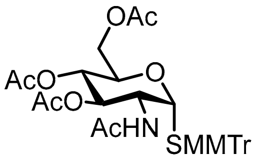

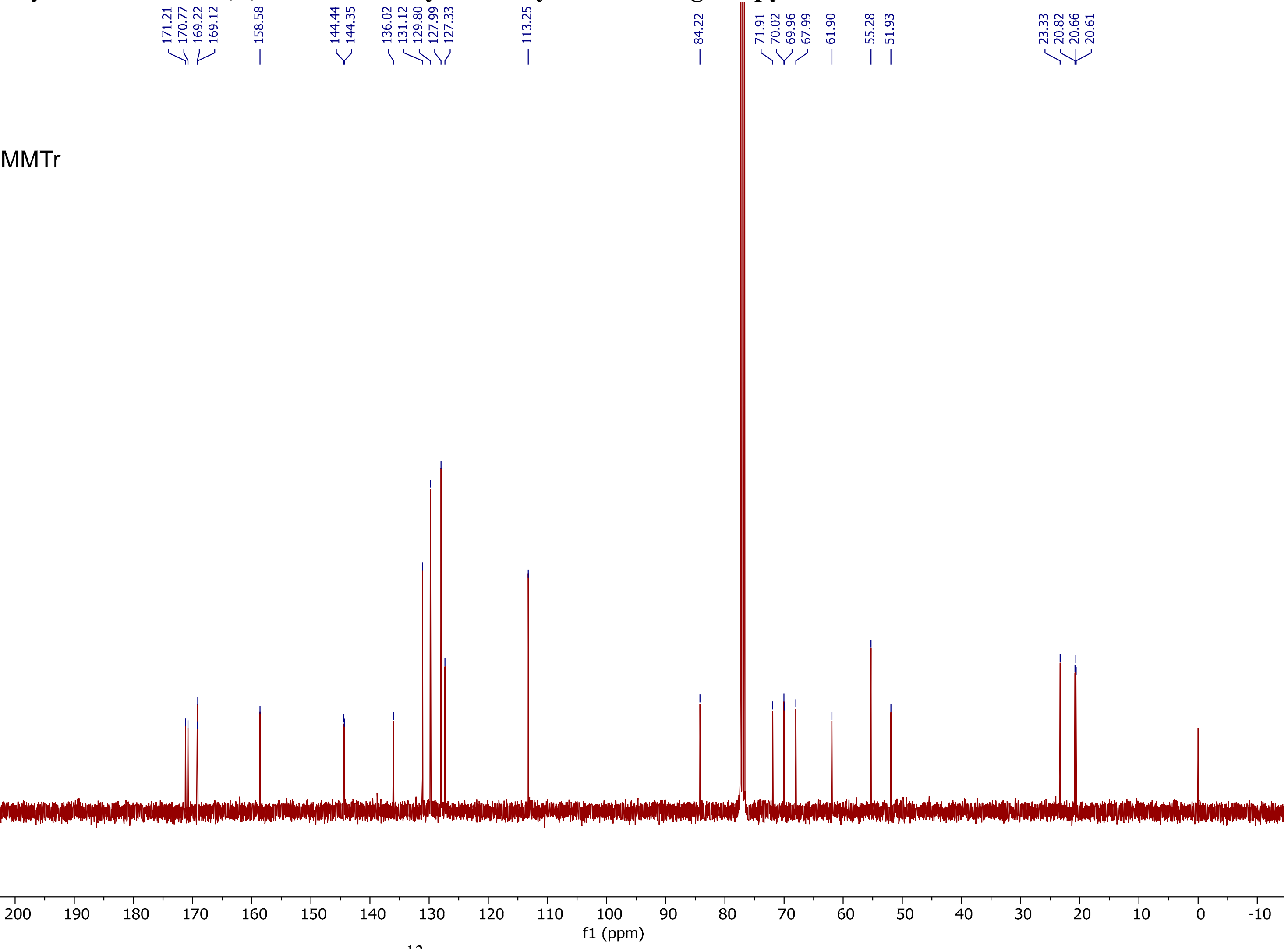

^{13}C NMR (100 MHz, Chloroform-_d_)

_p_-Monomethoxytrityl 2-acetamido-4,6-_O_-benzylidene-2-deoxy-1-thio-α-D-glucopyranoside 27

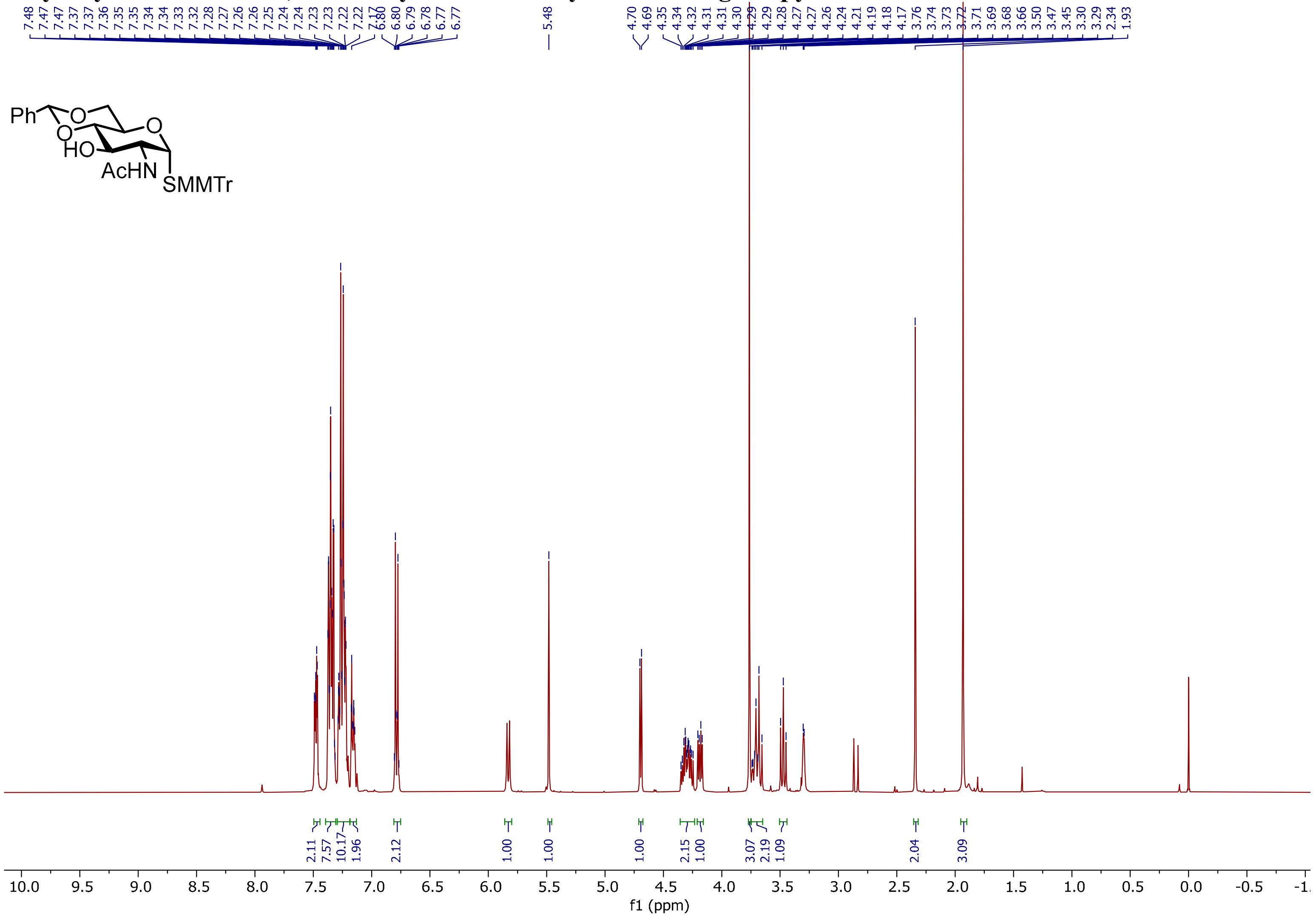

^{1}H NMR (400 MHz, Chloroform-*d*)

p-Monomethoxytrityl 2-acetamido-4,6-O-benzylidene-2-deoxy-1-thio-α-D-glucopyranoside 27

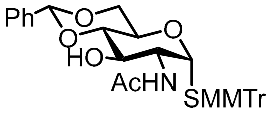

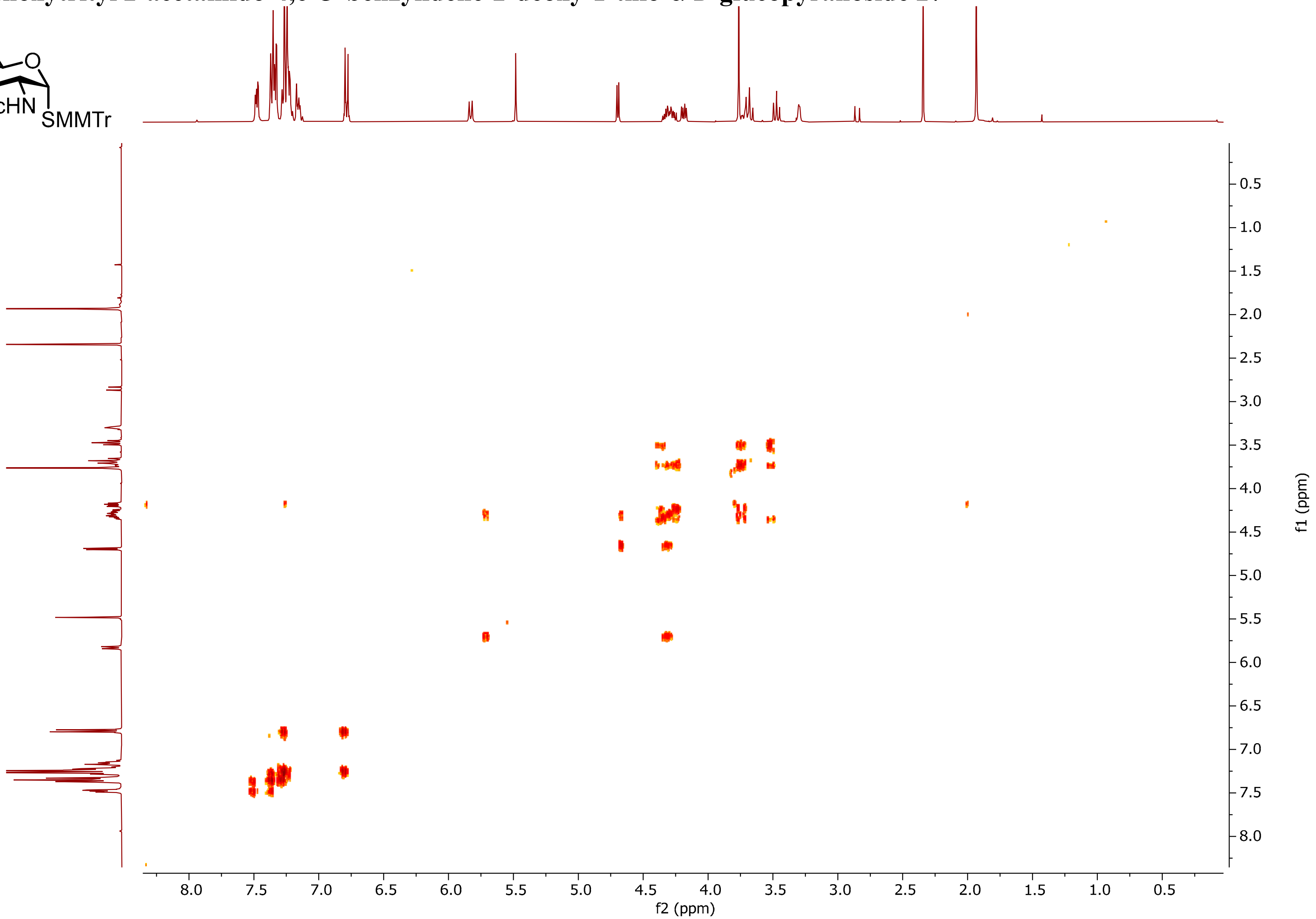

COSY (400 × 400 MHz, Chloroform-*d*)

_p_-Monomethoxytrityl 2-acetamido-4,6-_O_-benzylidene-2-deoxy-1-thio-α-D-glucopyranoside 27

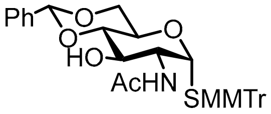

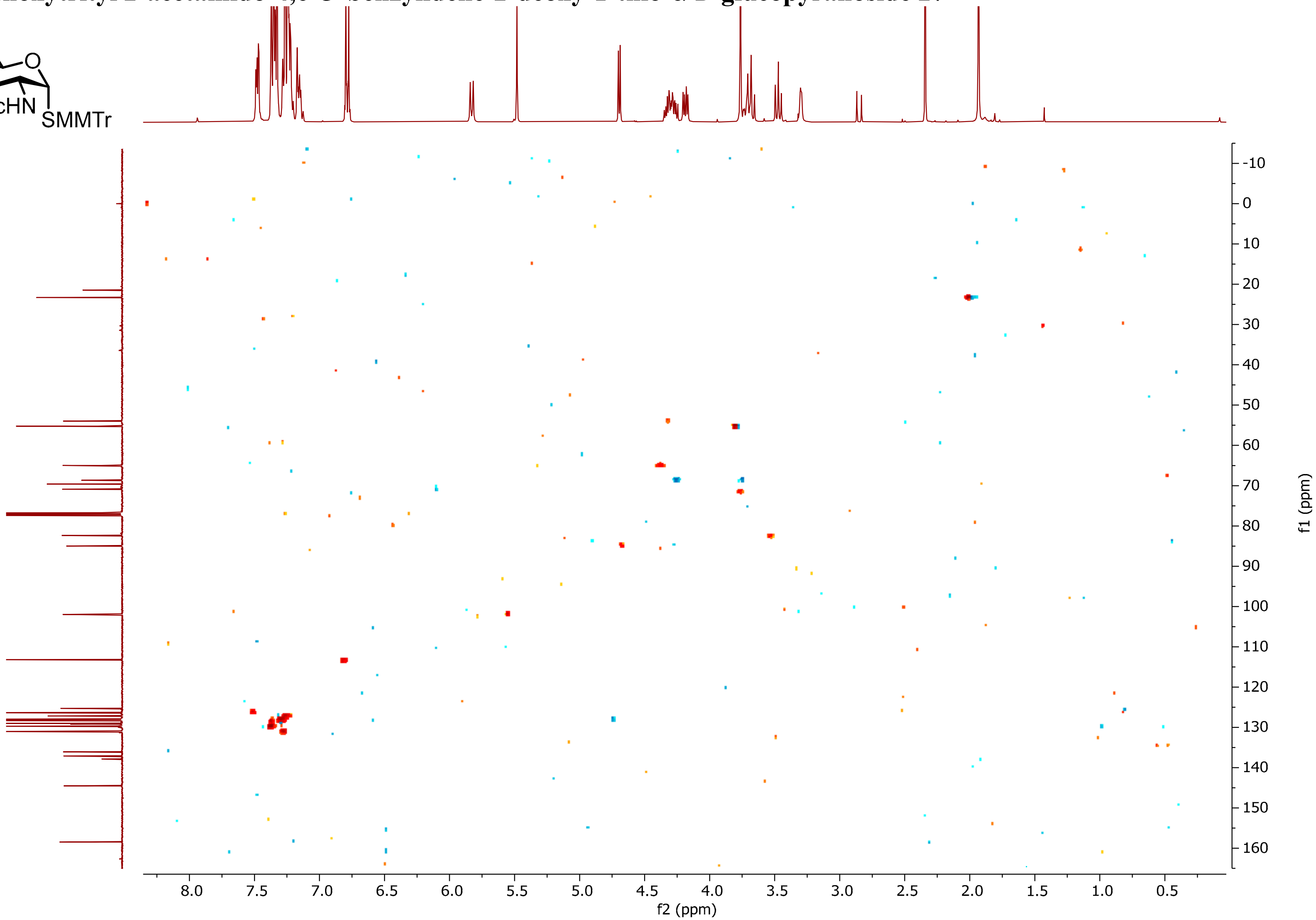

HSQC (400 × 100 MHz, Chloroform-_d_)

p-Monomethoxytrityl 2-acetamido-4,6-*O*-benzylidene-2-deoxy-1-thio-α-D-glucopyranoside 27

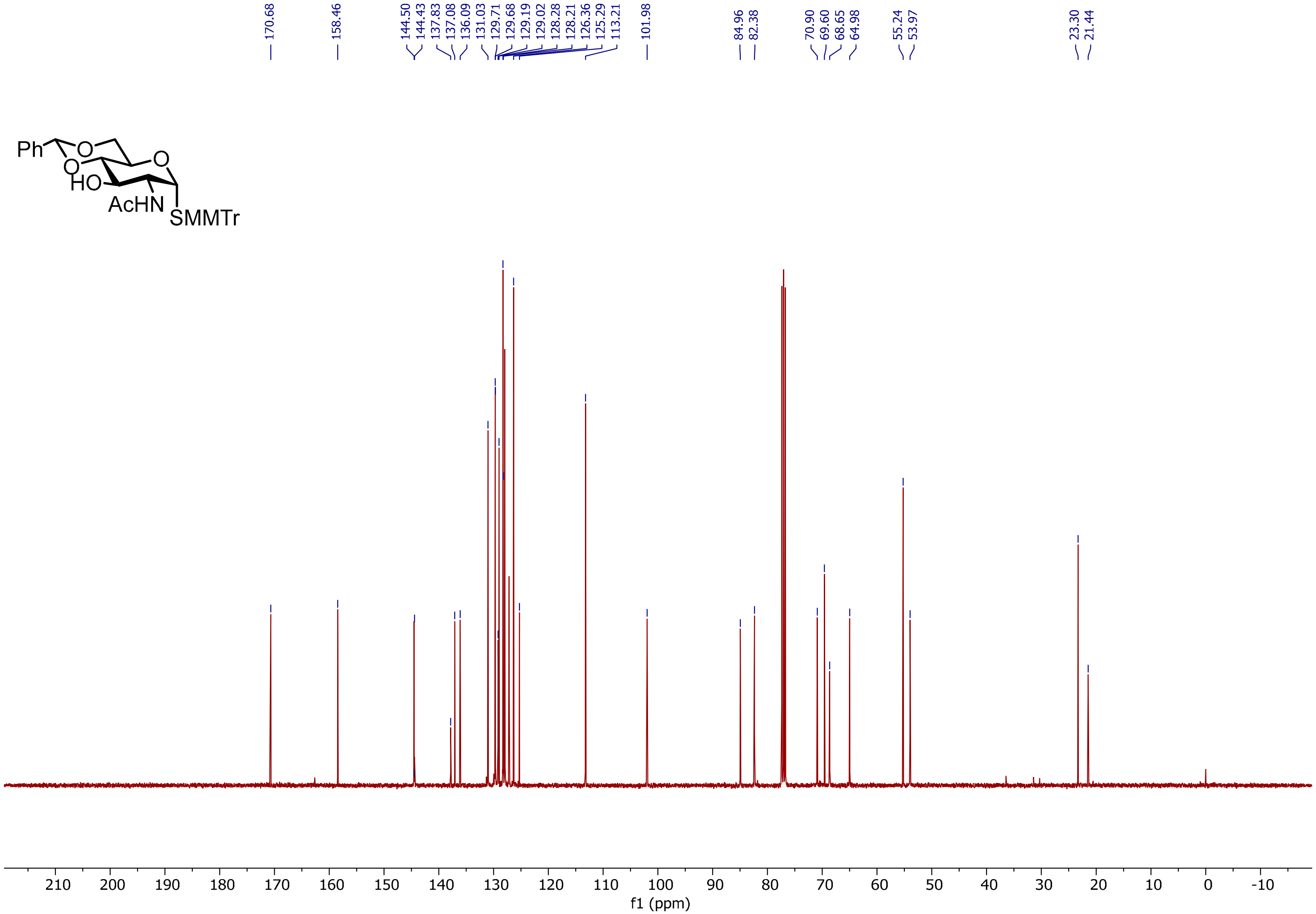

^{13}C NMR (100 MHz, Chloroform-*d*)

***p*-Monomethoxytrityl 2-acetamido-3-*O*-benzoyl-4,6-*O*-benzylidene-2-deoxy-1-thio-α-D-glucopyranoside 28**

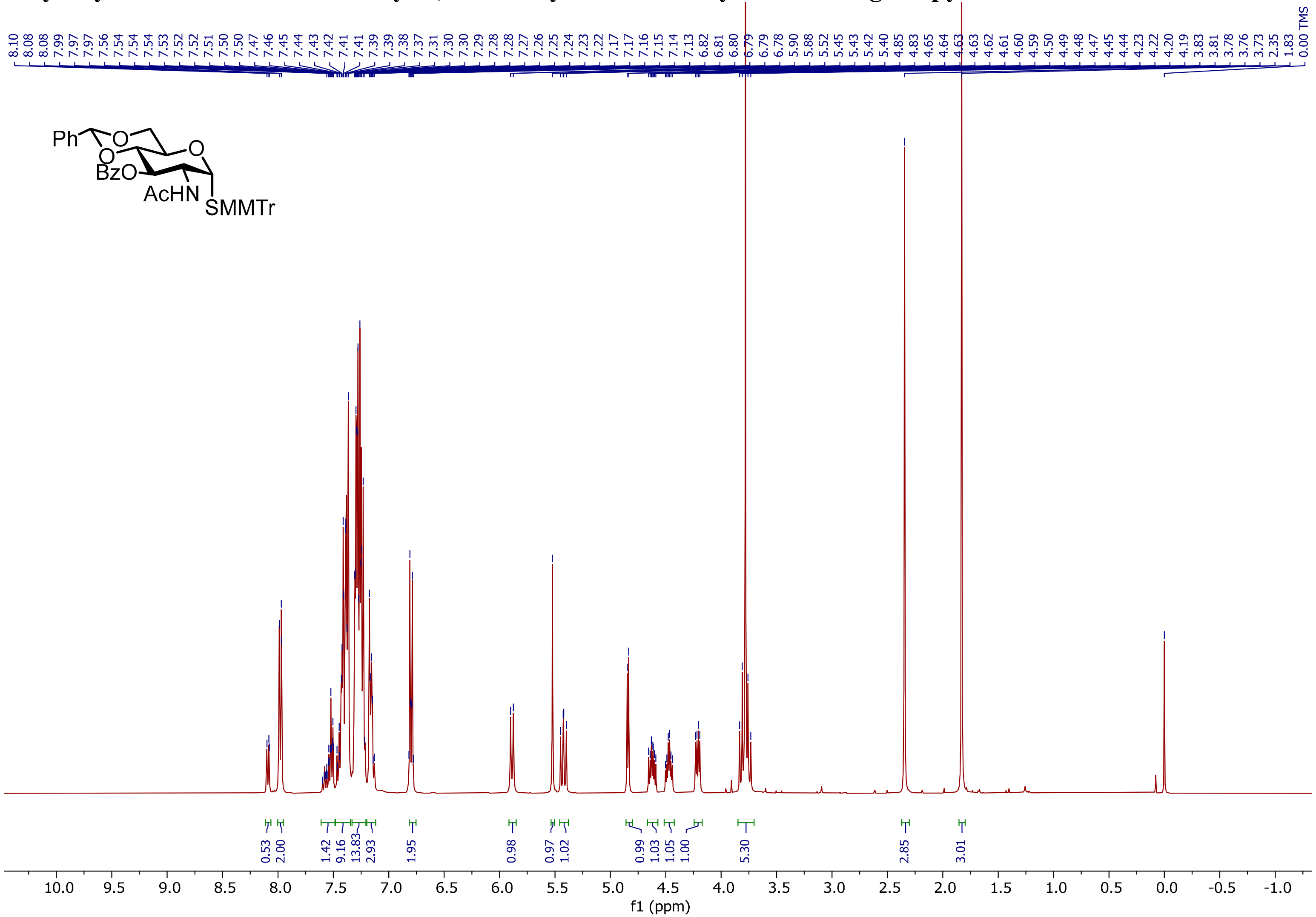

^{1}H NMR (400 MHz, Chloroform-*d*)

***p*-Monomethoxytrityl 2-acetamido-3-*O*-benzoyl-4,6-*O*-benzylidene-2-deoxy-1-thio-α-D-glucopyranoside 28**

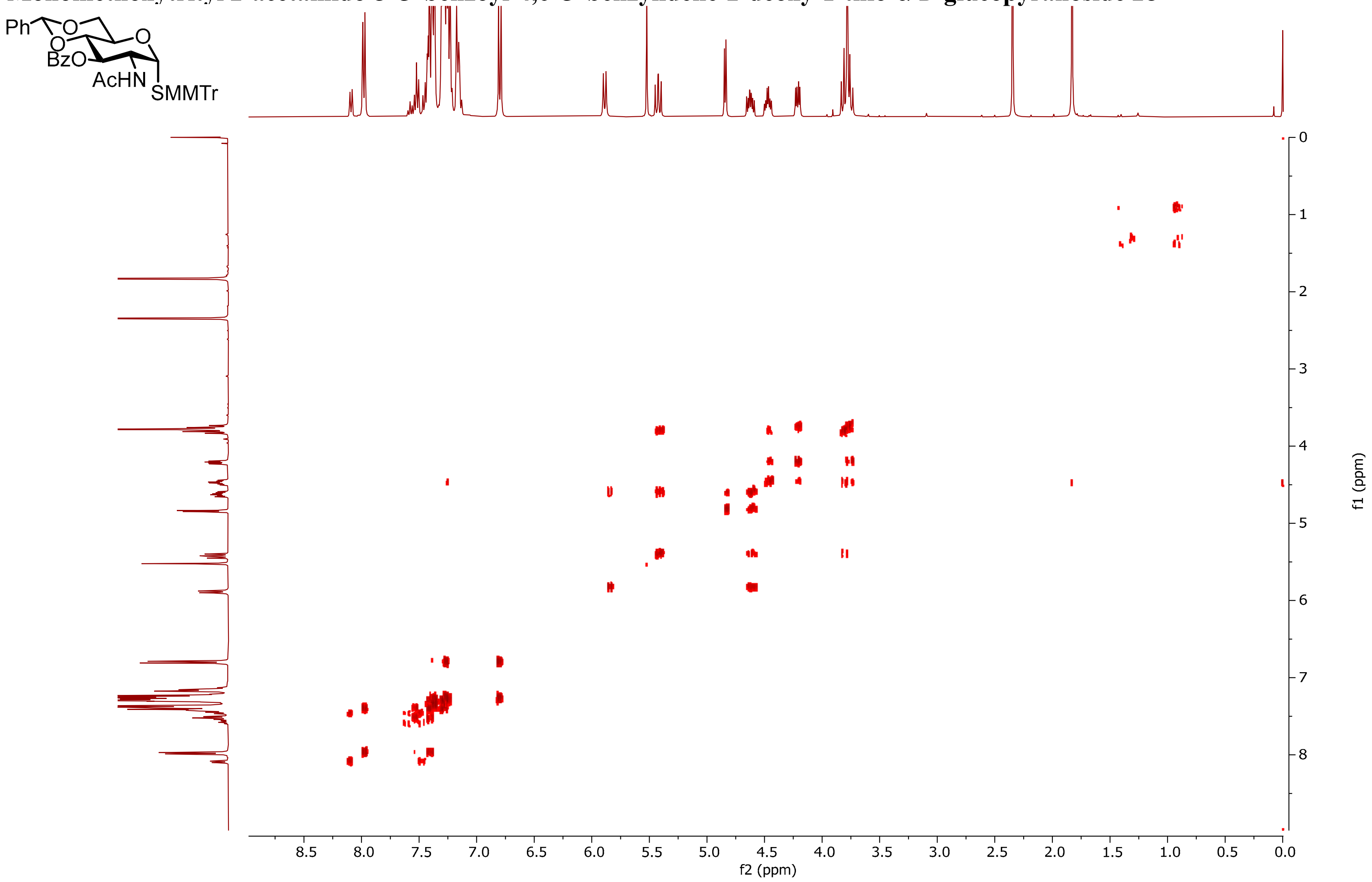

COSY (400 × 400 MHz, Chloroform-*d*)

_p_-Monomethoxytrityl 2-acetamido-3-_O_-benzoyl-4,6-_O_-benzylidene-2-deoxy-1-thio-α-D-glucopyranoside 28

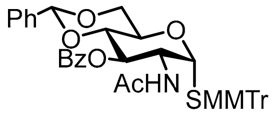

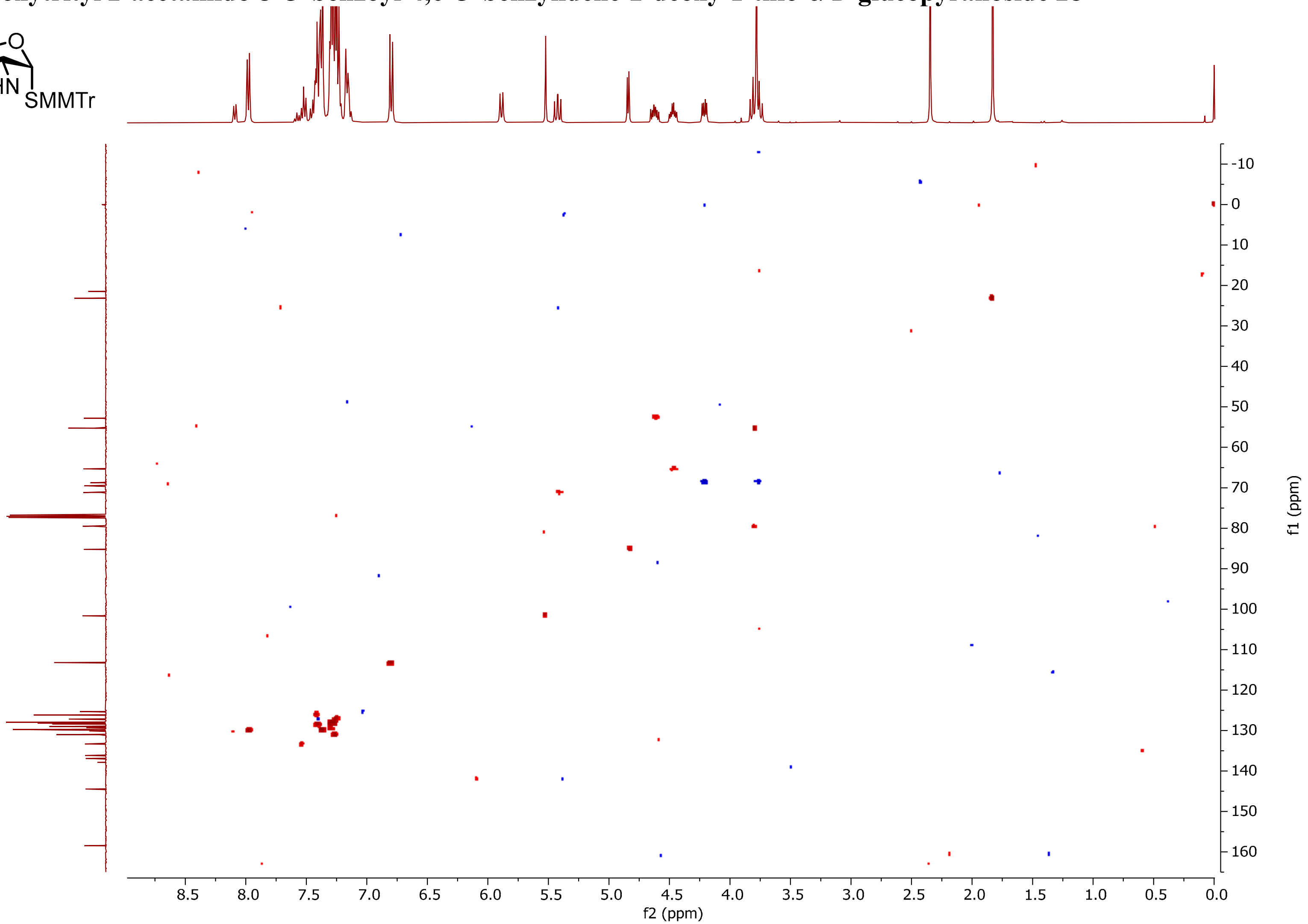

HSQC (400 × 100 MHz, Chloroform-_d_)

_p_-Monomethoxytrityl 2-acetamido-3-_O_-benzoyl-4,6-_O_-benzylidene-2-deoxy-1-thio-α-D-glucopyranoside 28

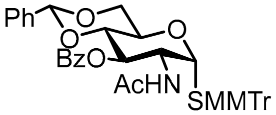

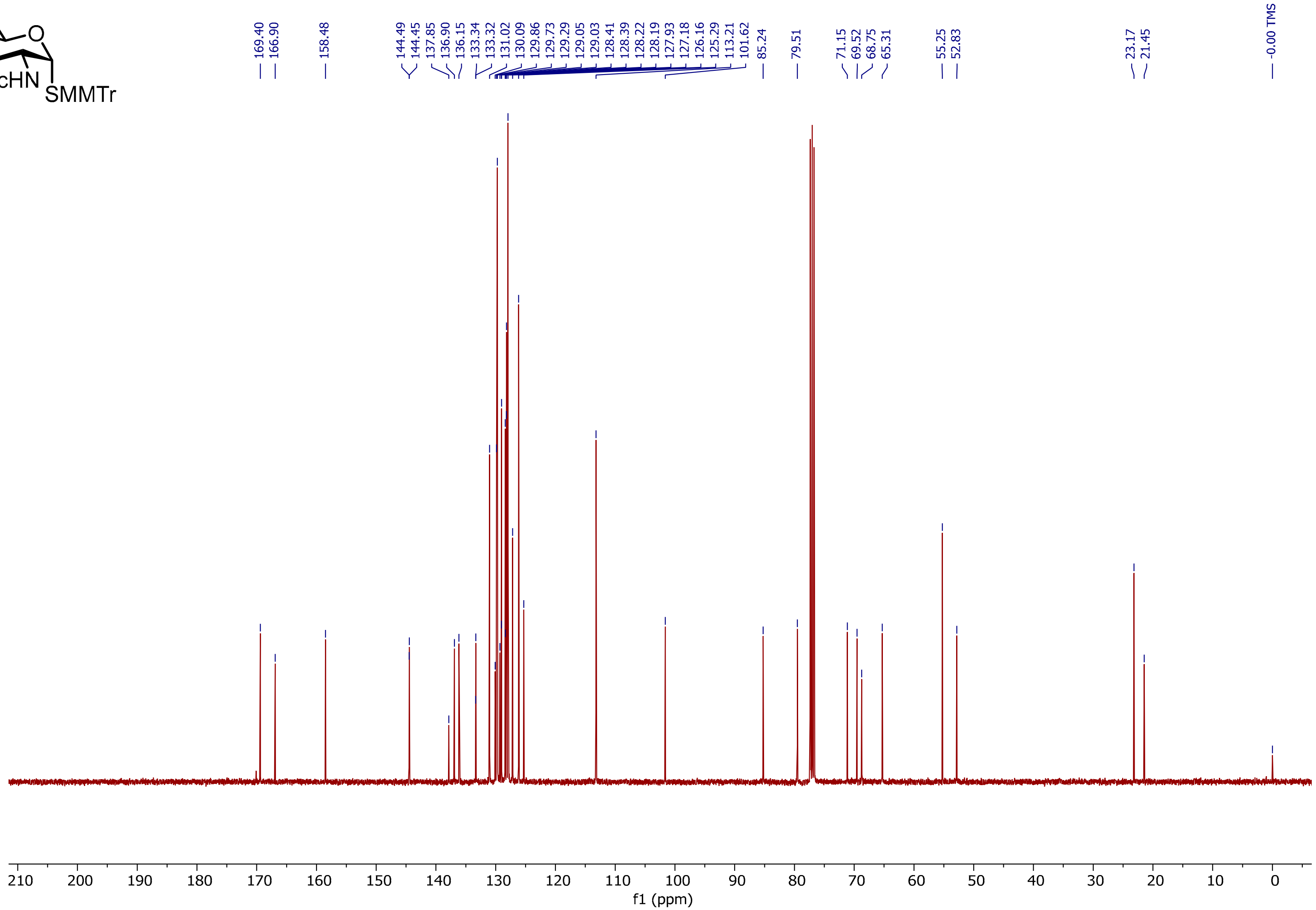

^{13}C NMR (100 MHz, Chloroform-*d*)

***p*-Monomethoxytrityl 2-acetamido-3-*O*-benzoyl-2-deoxy-1-thio-α-D-glucopyranoside 29**

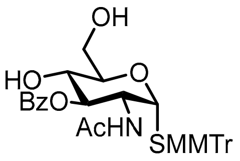

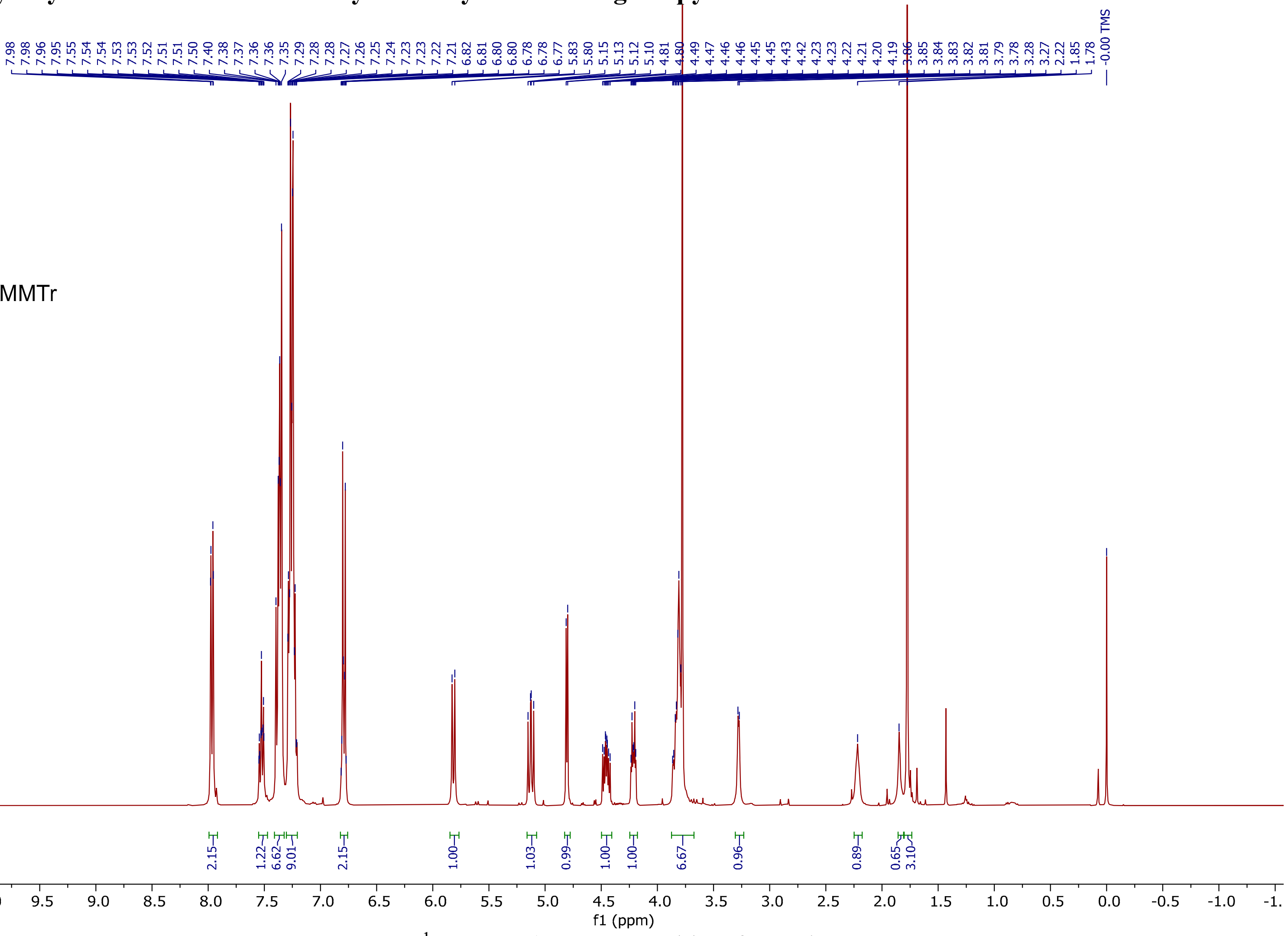

^{1}H NMR (400 MHz, Chloroform-*d*)

_p_-Monomethoxytrityl 2-acetamido-3-_O_-benzoyl-2-deoxy-1-thio-α-D-glucopyranoside 29

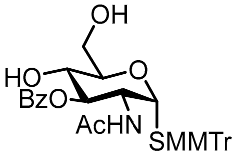

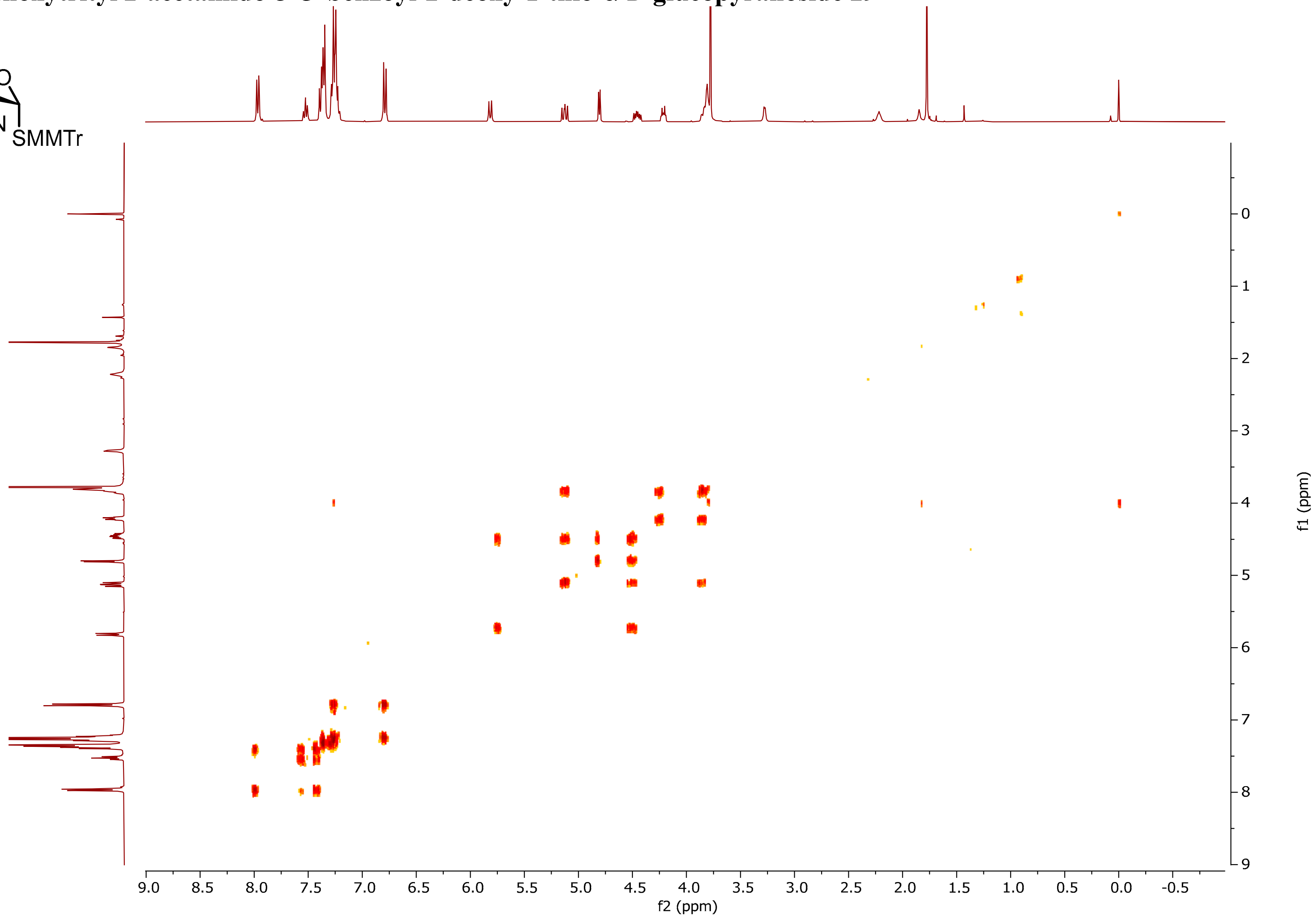

COSY (400 × 400 MHz, Chloroform-_d_)

***p*-Monomethoxytrityl 2-acetamido-3-*O*-benzoyl-2-deoxy-1-thio-α-D-glucopyranoside 29**

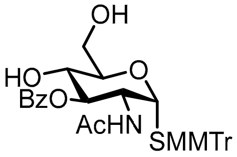

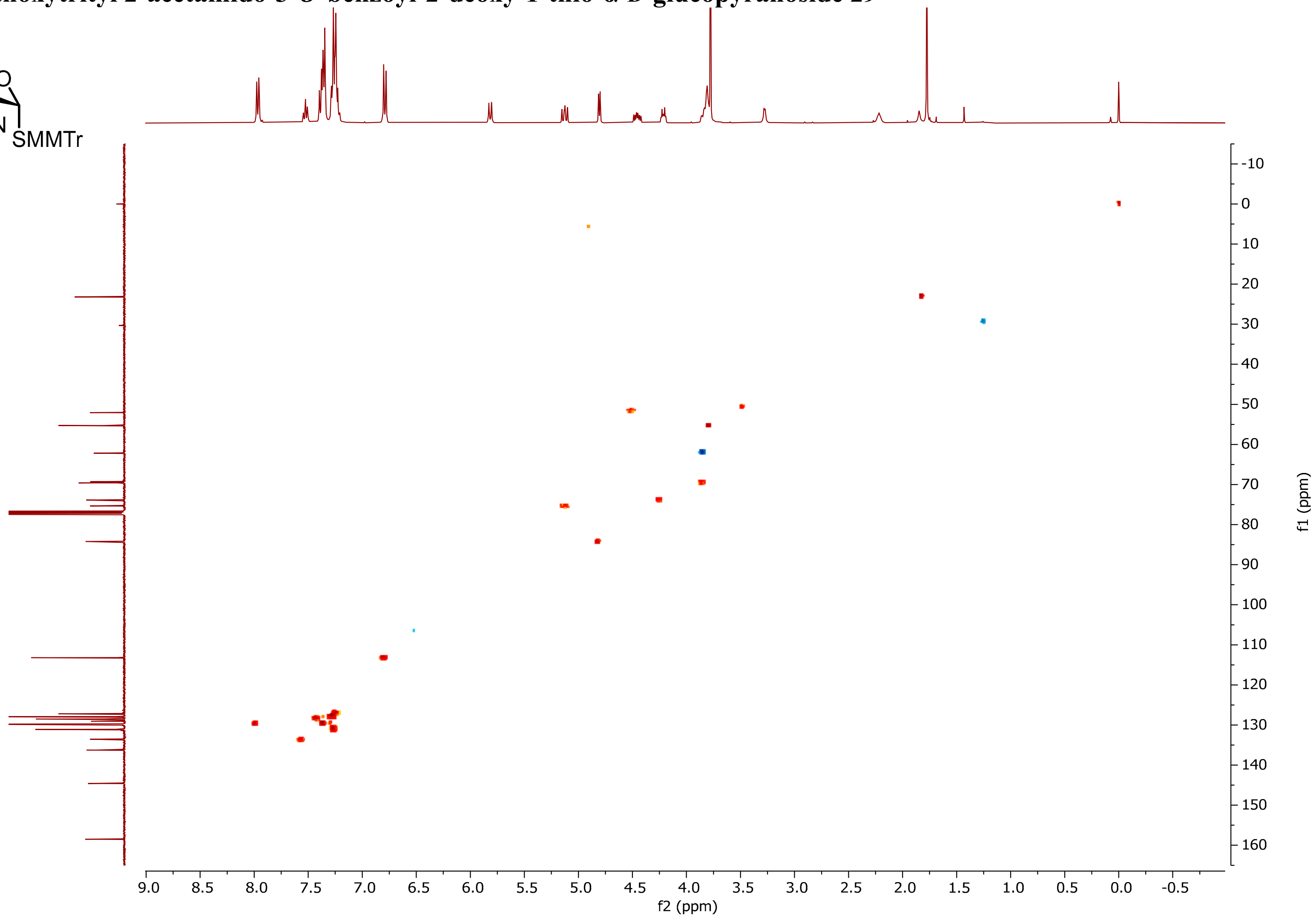

HSQC (400 × 100 MHz, Chloroform-*d*)

p-Monomethoxytrityl 2-acetamido-3-O-benzoyl-2-deoxy-1-thio-α-D-glucopyranoside 29

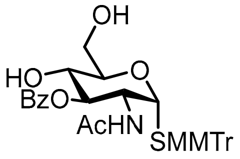

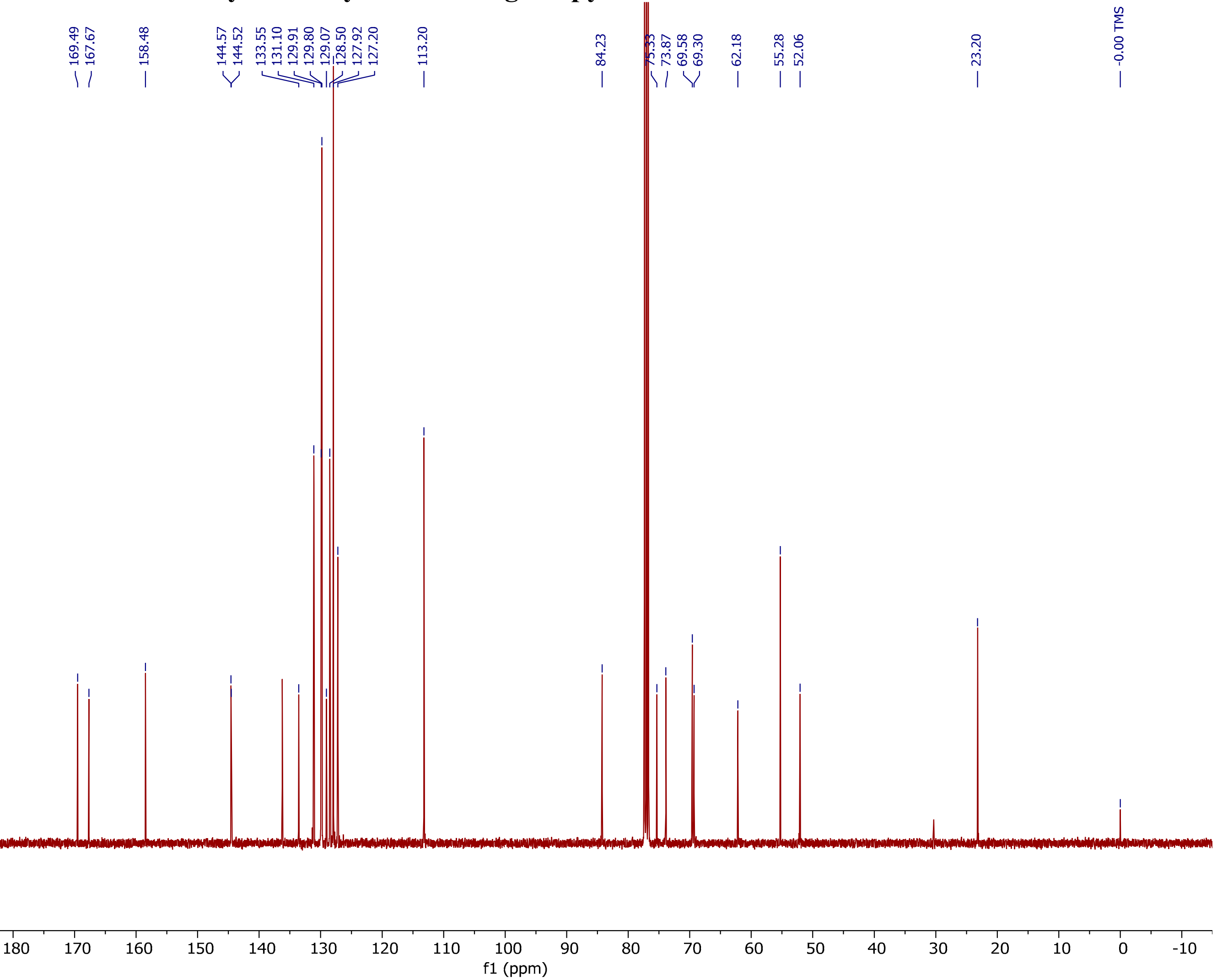

^{13}C NMR (100 MHz, Chloroform-*d*)

p-Monomethoxytrityl 2-acetamido-3,6-di-O-benzoyl-2-deoxy-1-thio-α-D-glucopyranoside 30

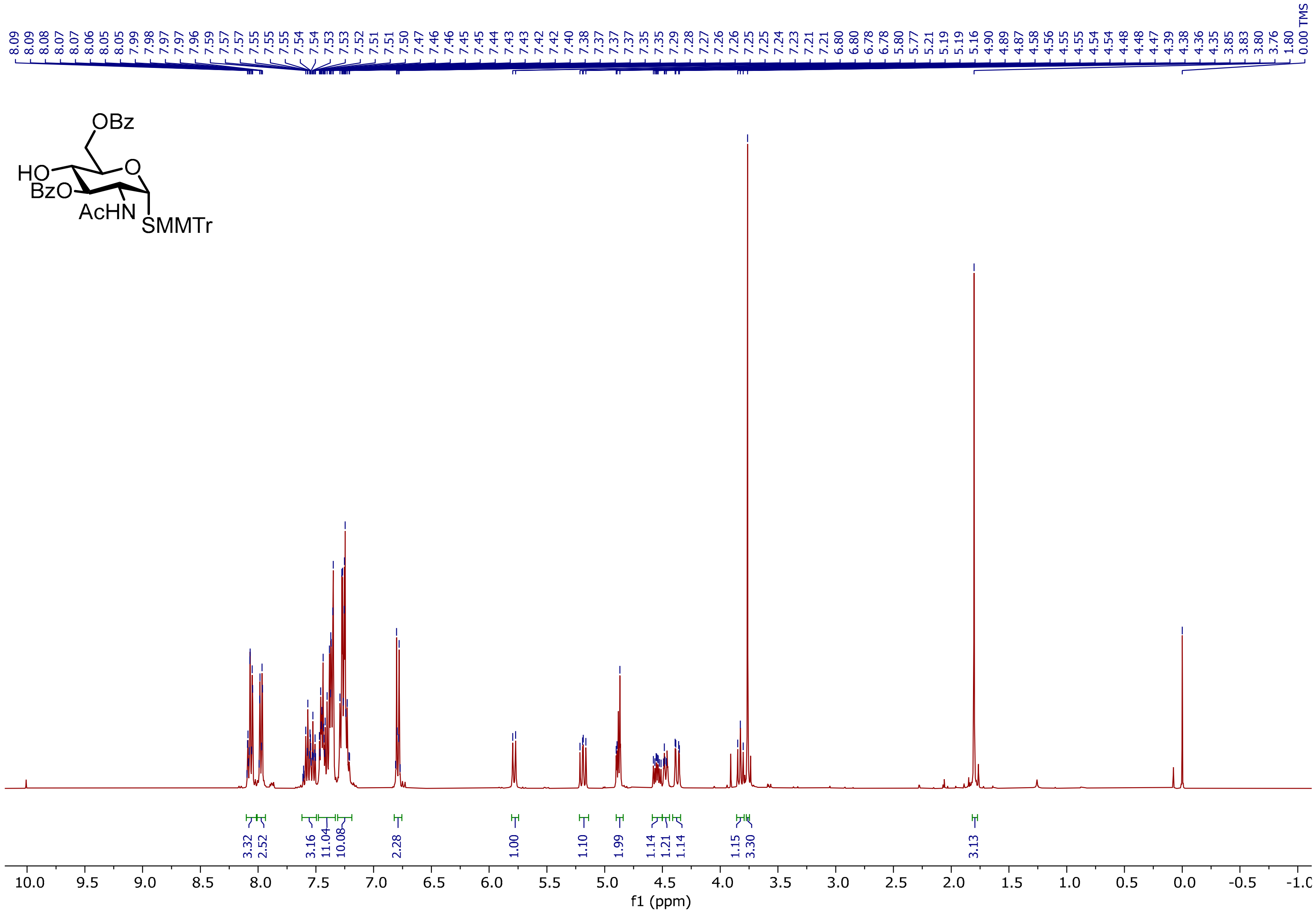

^{1}H NMR (400 MHz, Chloroform-d)

***p*-Monomethoxytrityl 2-acetamido-3,6-di-*O*-benzoyl-2-deoxy-1-thio-α-D-glucopyranoside 30**

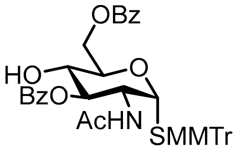

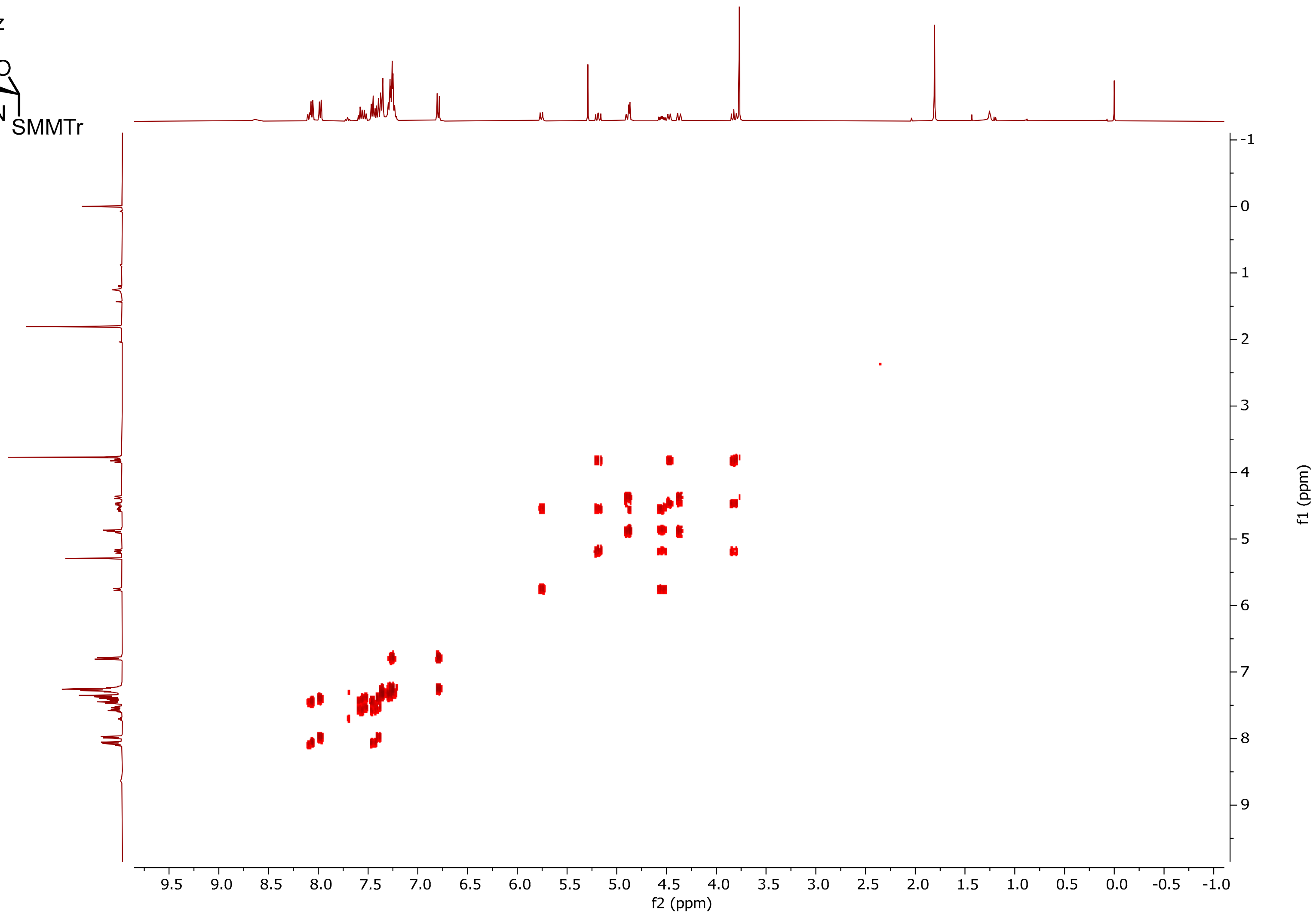

COSY (400 × 400 MHz, Chloroform-*d*)

_p_-Monomethoxytrityl 2-acetamido-3,6-di-_O_-benzoyl-2-deoxy-1-thio-α-D-glucopyranoside 30

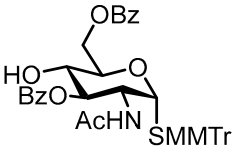

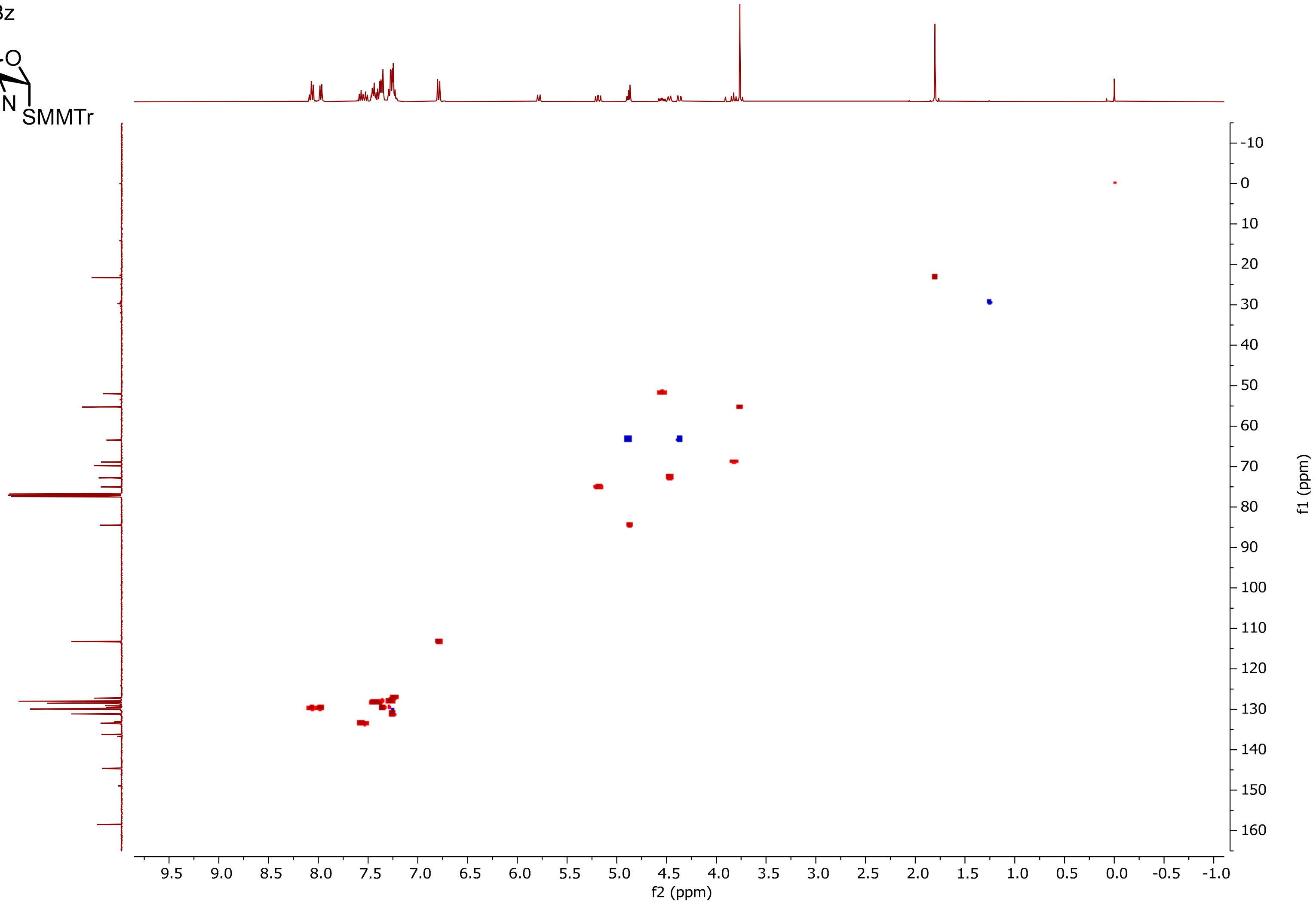

HSQC (400 × 100 MHz, Chloroform-_d_)

_p_-Monomethoxytrityl 2-acetamido-3,6-di-_O_-benzoyl-2-deoxy-1-thio-α-D-glucopyranoside 30

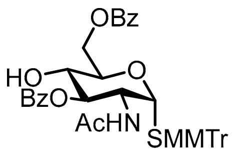

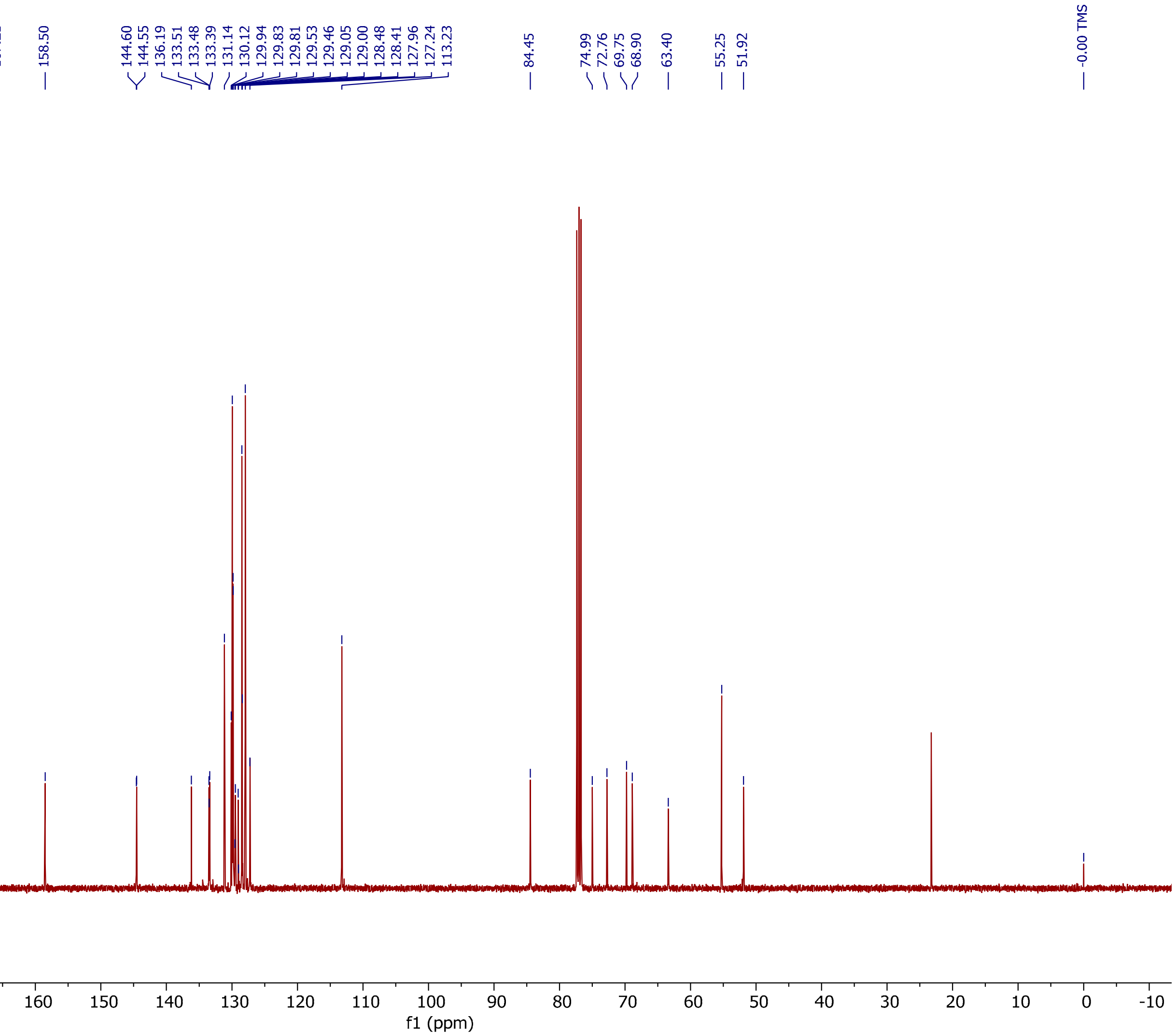

^{13}C NMR (100 MHz, Chloroform-_d_)

p-Monomethoxytrityl 2-acetamido-3,6-di-O-benzoyl-2-deoxy-4-O-levulinoyl-1-thio-α-D-glucopyranoside 32

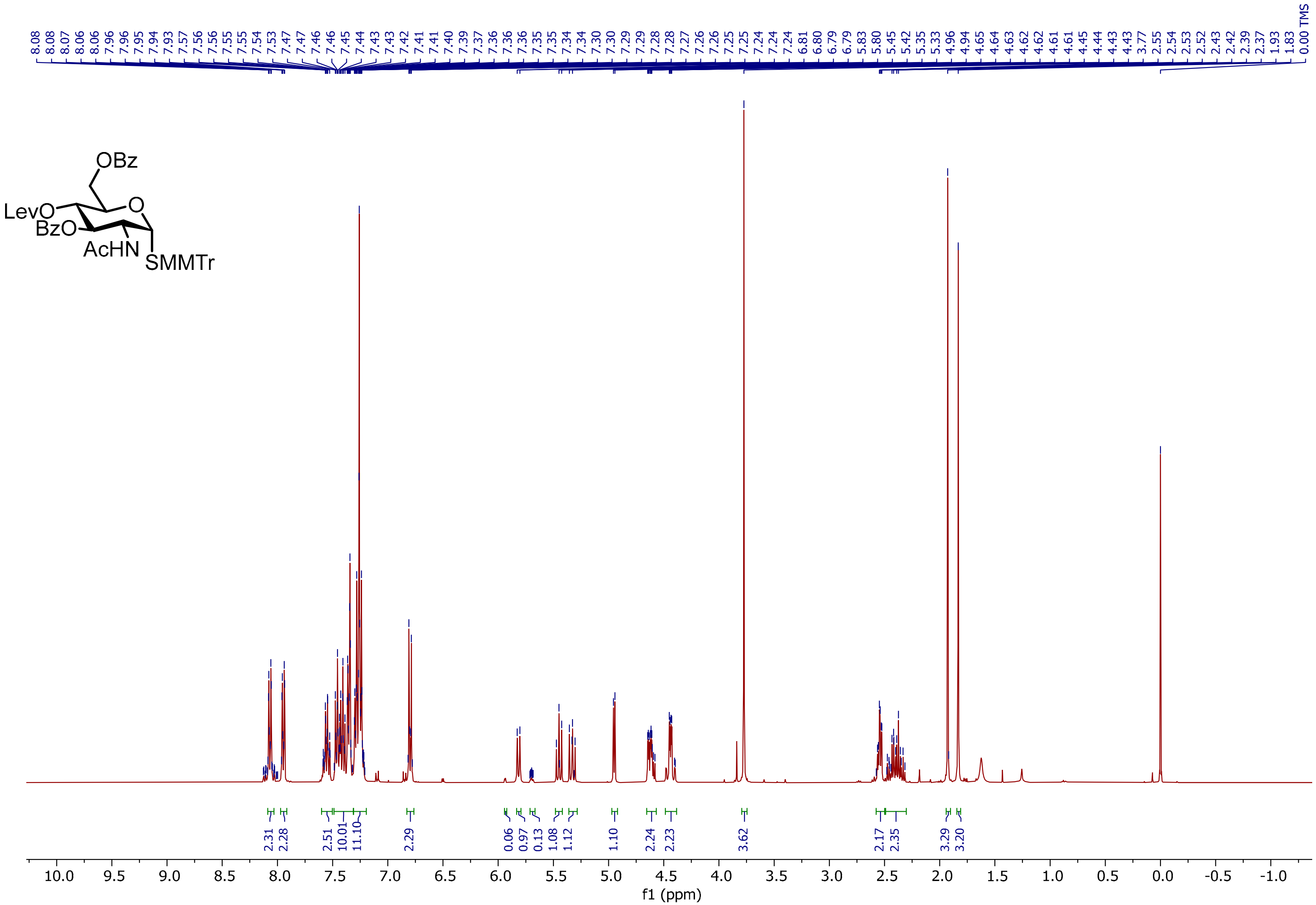

^{1}H NMR (400 MHz, Chloroform-*d*)

p-Monomethoxytrityl 2-acetamido-3,6-di-*O*-benzoyl-2-deoxy-4-*O*-levulinoyl-1-thio-α-D-glucopyranoside 32

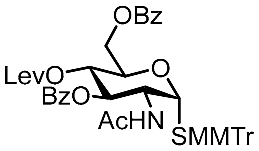

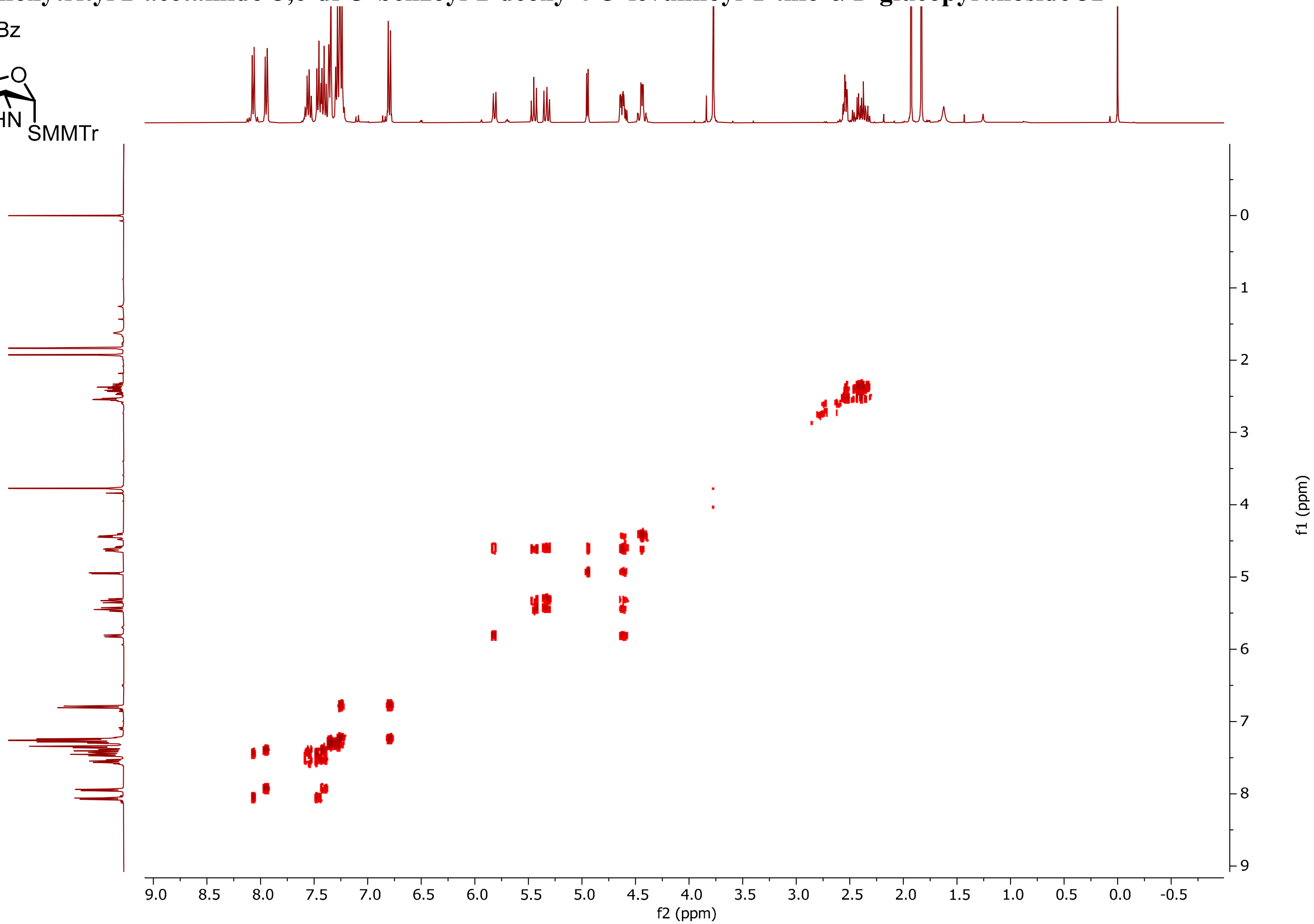

COSY (400 × 400 MHz, Chloroform-*d*)

***p*-Monomethoxytrityl 2-acetamido-3,6-di-*O*-benzoyl-2-deoxy-4-*O*-levulinoyl-1-thio-α-D-glucopyranoside 32**

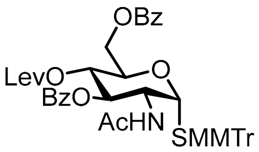

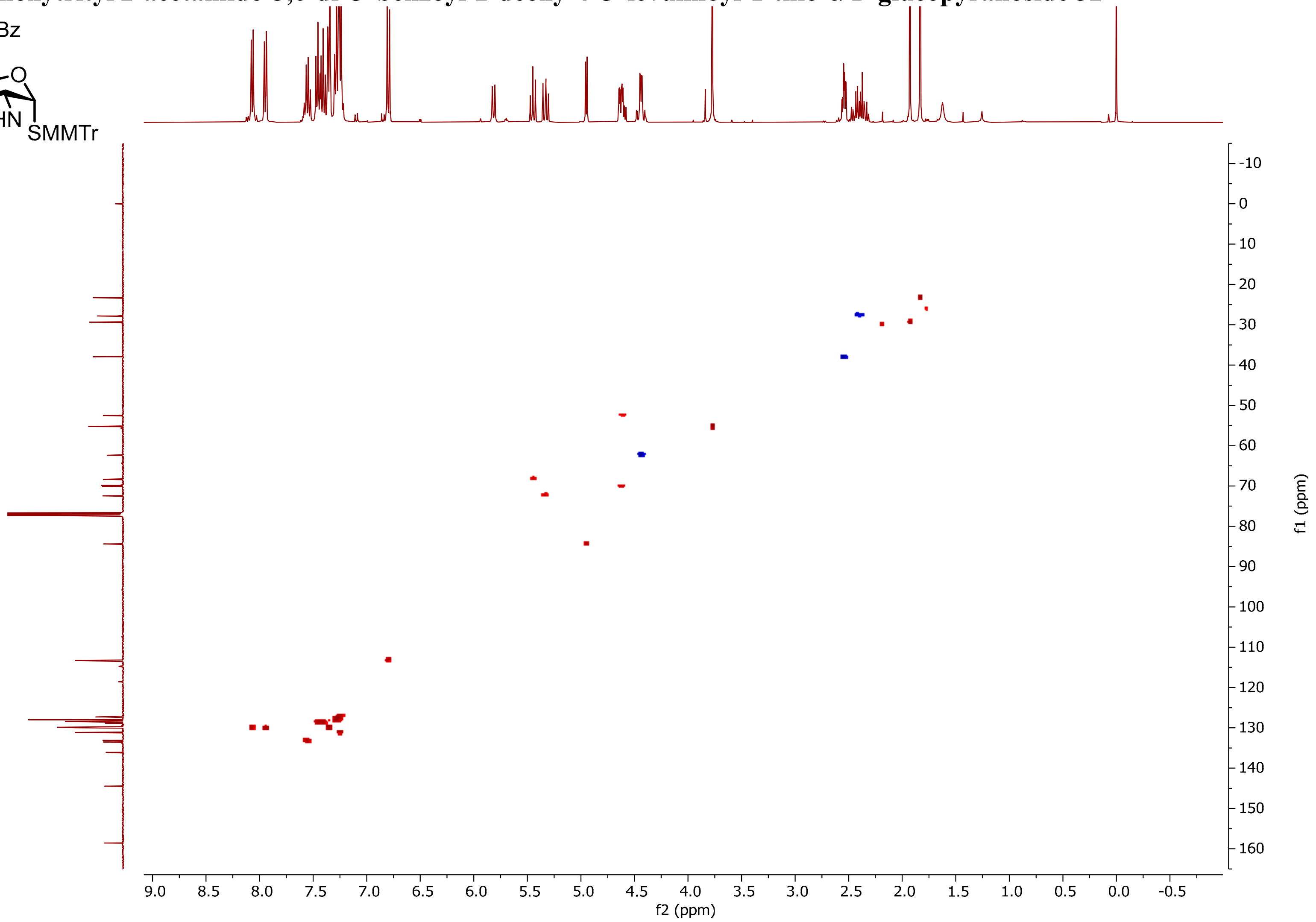

HSQC (400 × 100 MHz, Chloroform-*d*)

p-Monomethoxytrityl 2-acetamido-3,6-di-O-benzoyl-2-deoxy-4-O-levulinoyl-1-thio-α-D-glucopyranoside 32

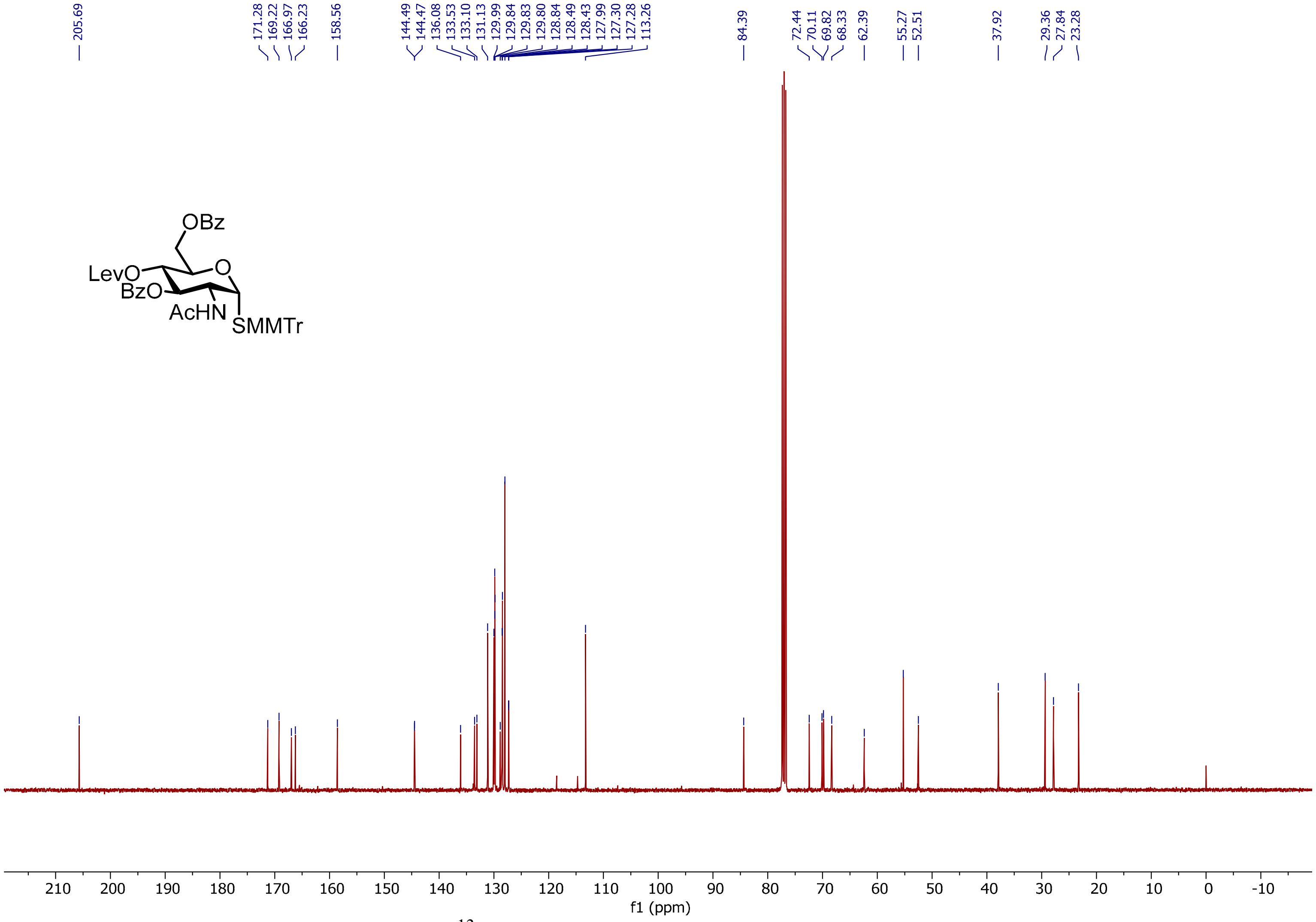

^{13}C NMR (100 MHz, Chloroform-*d*)

2-Acetamide-3,6-di-*O*-benzoyl-2-deoxy-4-*O*-levulinoyl-1-thio-α-D-glucopyranose 34

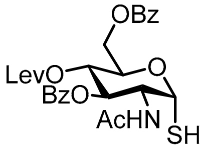

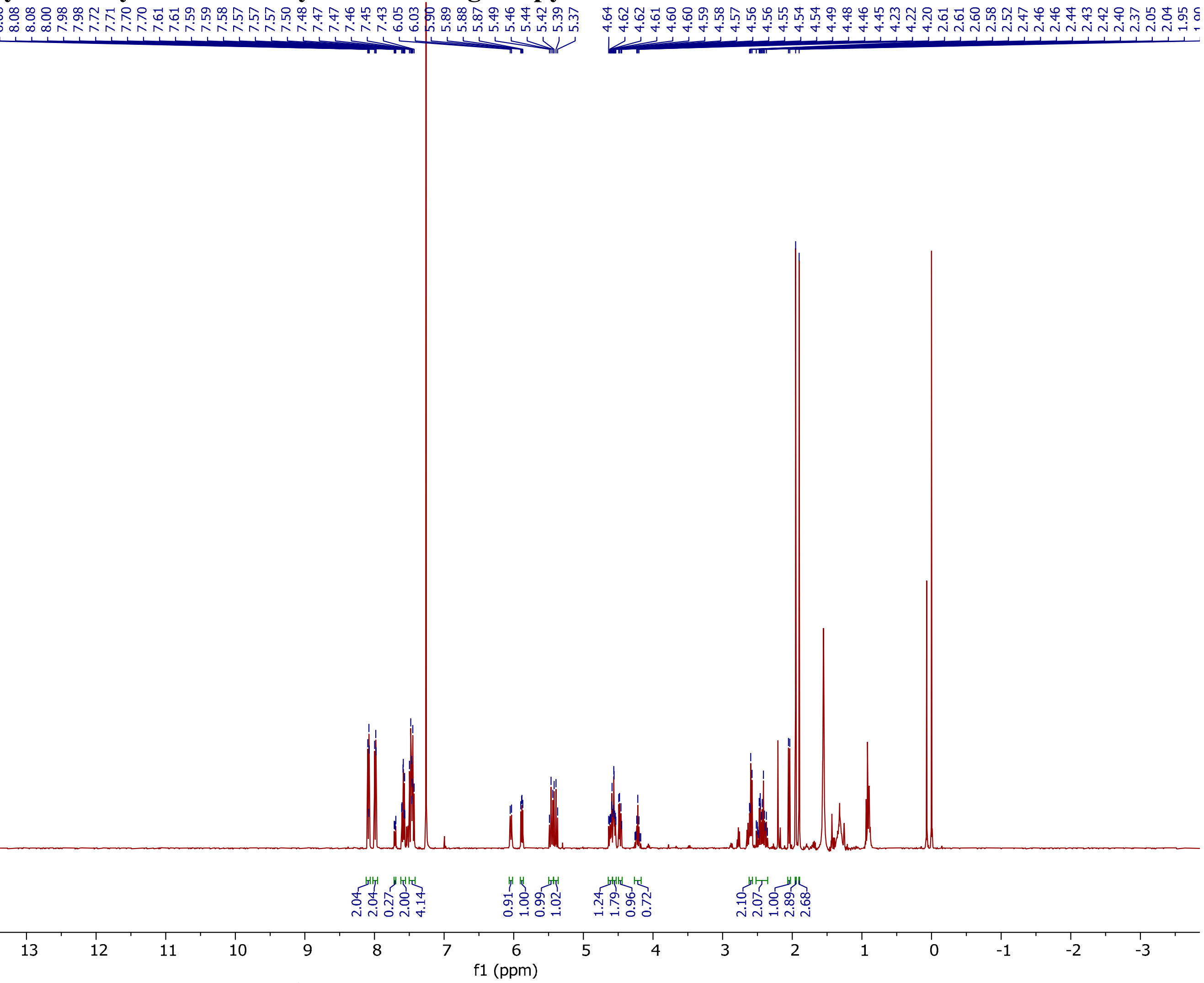

^{1}H NMR (400 MHz, Chloroform-*d*)

2-Acetamide-3,6-di-*O*-benzoyl-2-deoxy-4-*O*-levulinoyl-1-thio-α-D-glucopyranose 34

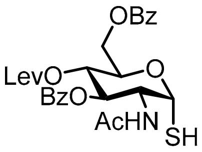

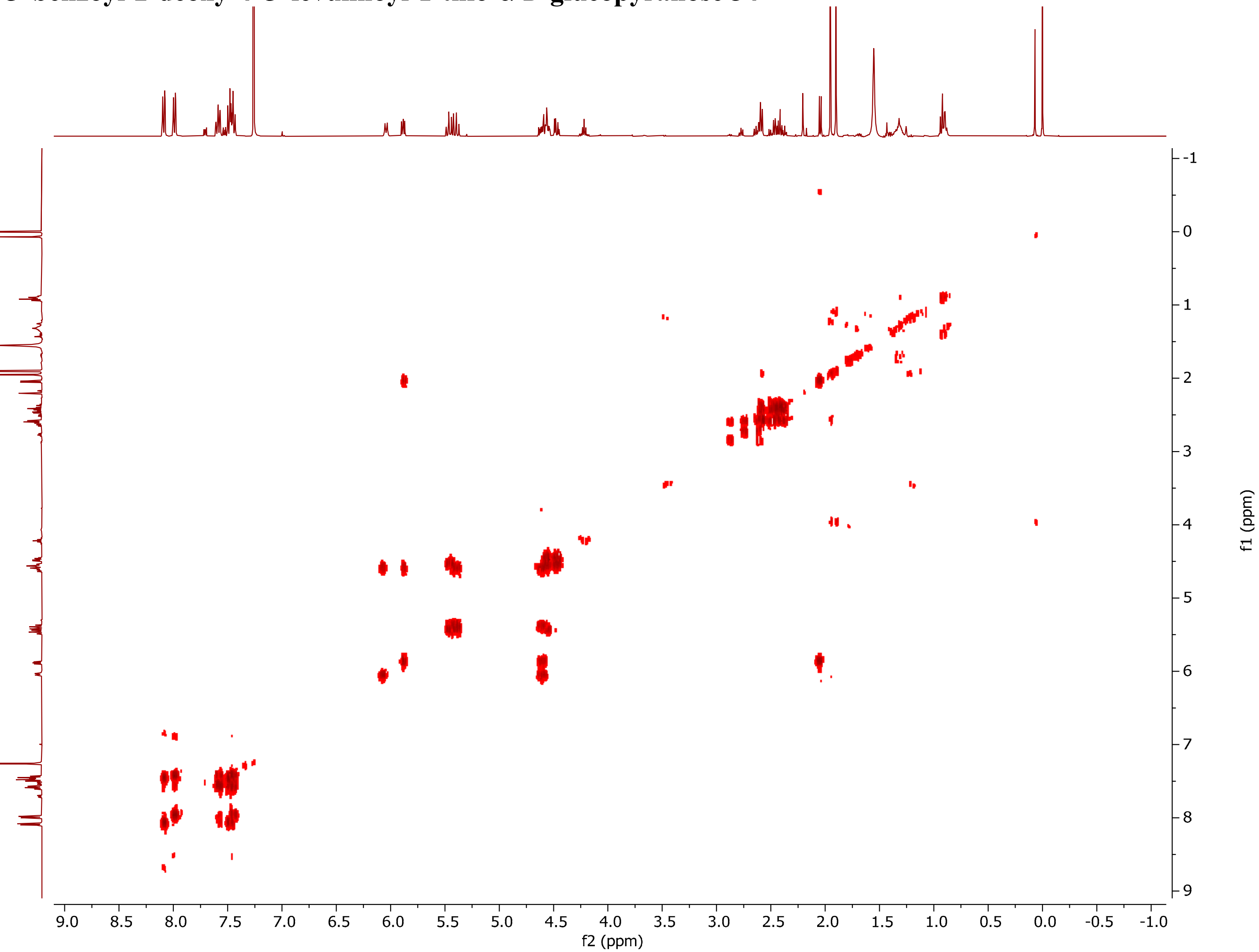

COSY (400 × 400 MHz, Chloroform-*d*)

2-Acetamide-3,6-di-*O*-benzoyl-2-deoxy-4-*O*-levulinoyl-1-thio-α-D-glucopyranose 34

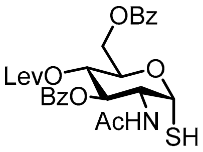

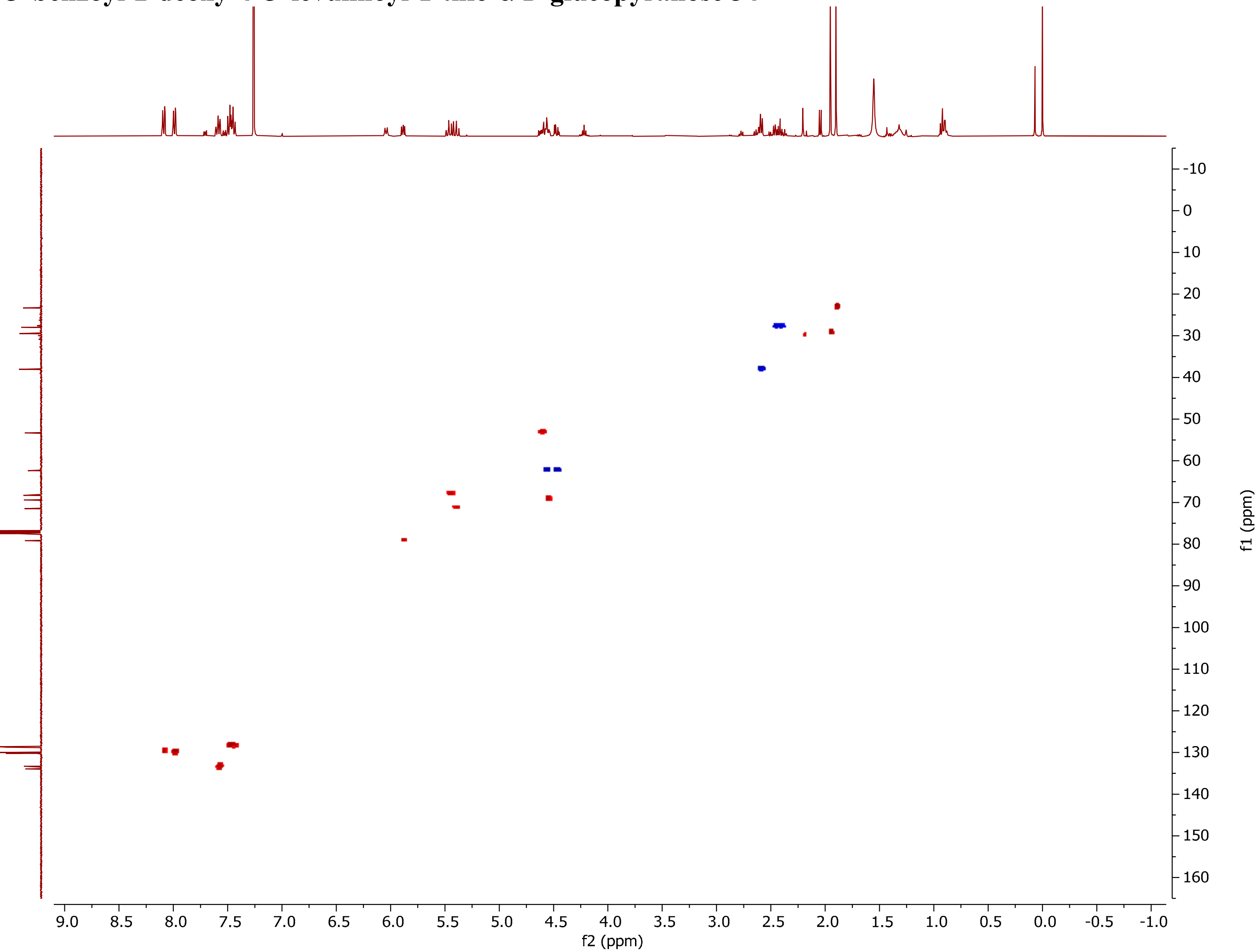

HSQC (400 × 100 MHz, Chloroform-*d*)

2-Acetamide-3,6-di-*O*-benzoyl-2-deoxy-4-*O*-levulinoyl-1-thio-α-D-glucopyranose 34

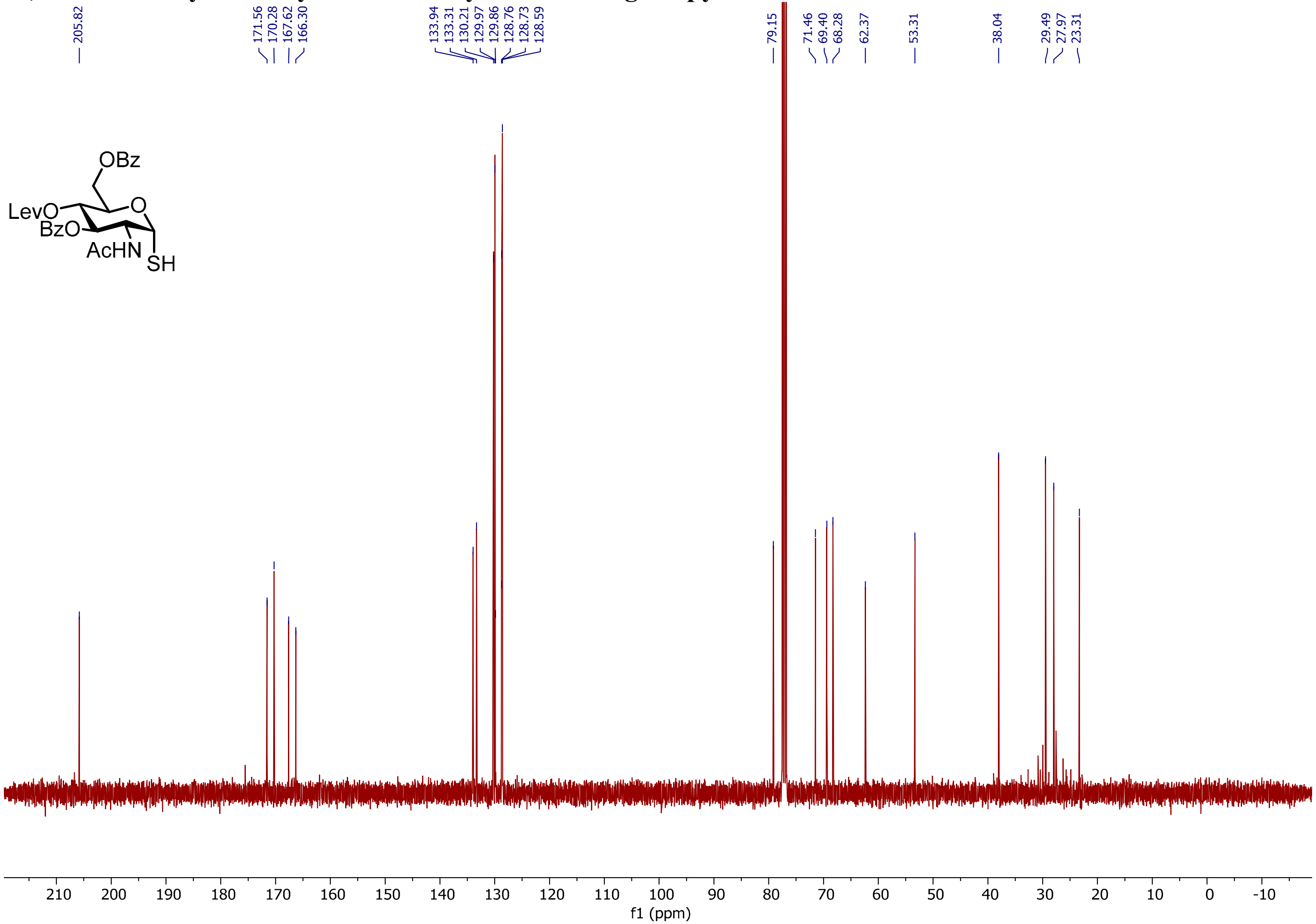

^{13}C NMR (100 MHz, Chloroform-*d*)

_p_-Monomethoxytrityl 2-acetamido-3-_O_-benzoyl-2-deoxy-4-_O_-levulinoyl-6-_O_-(_tert_-butyldiphenylsilyl)-1-thio-α-D-glucopyranoside 33

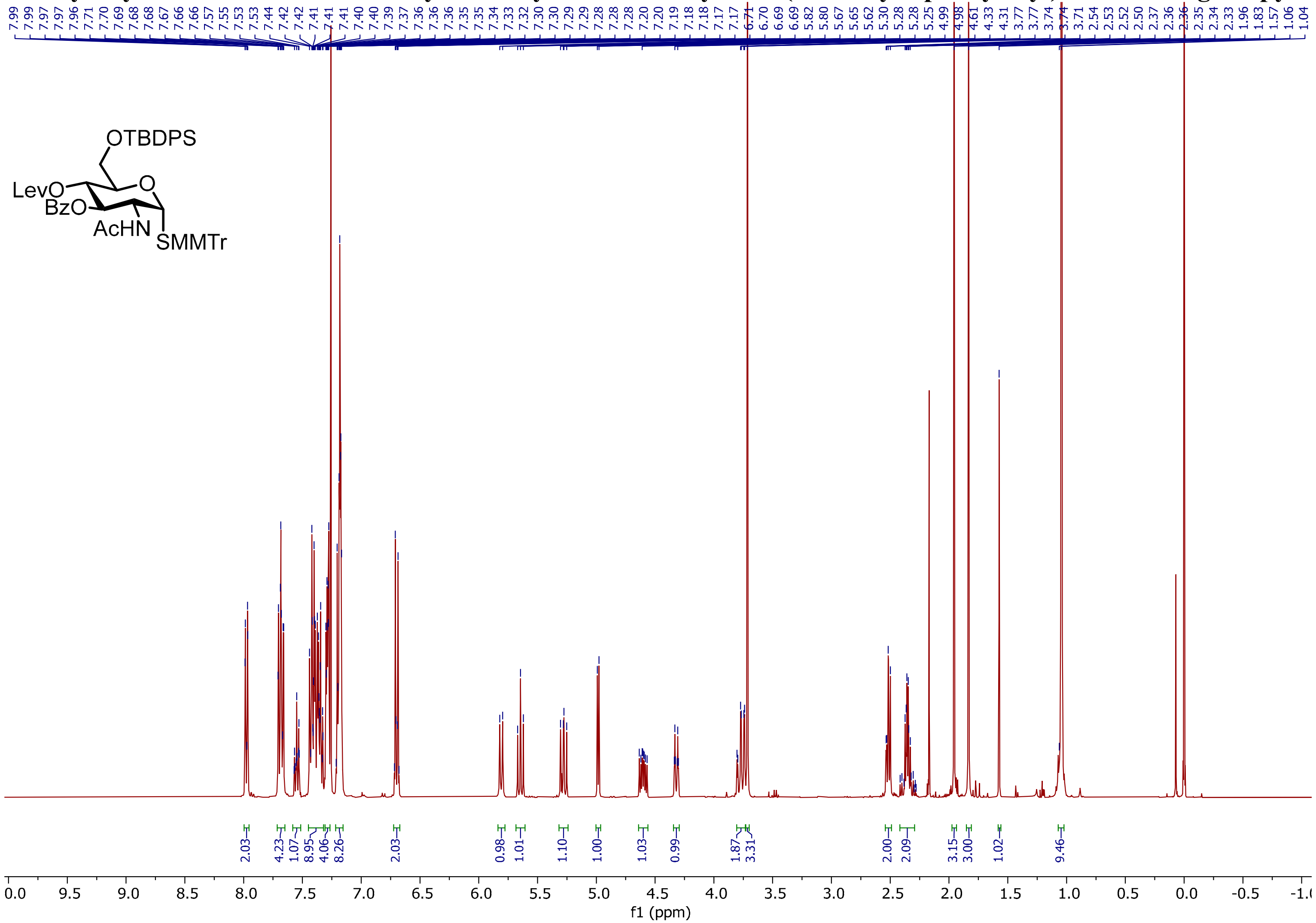

^{1}H NMR (400 MHz, Chloroform-_d_)

_p_-Monomethoxytrityl 2-acetamido-3-_O_-benzoyl-2-deoxy-4-_O_-levulinoyl-6-_O_-(_tert_-butyldiphenylsilyl)-1-thio-α-D-glucopyranoside 33

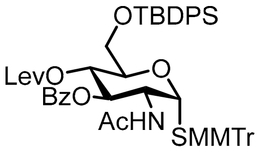

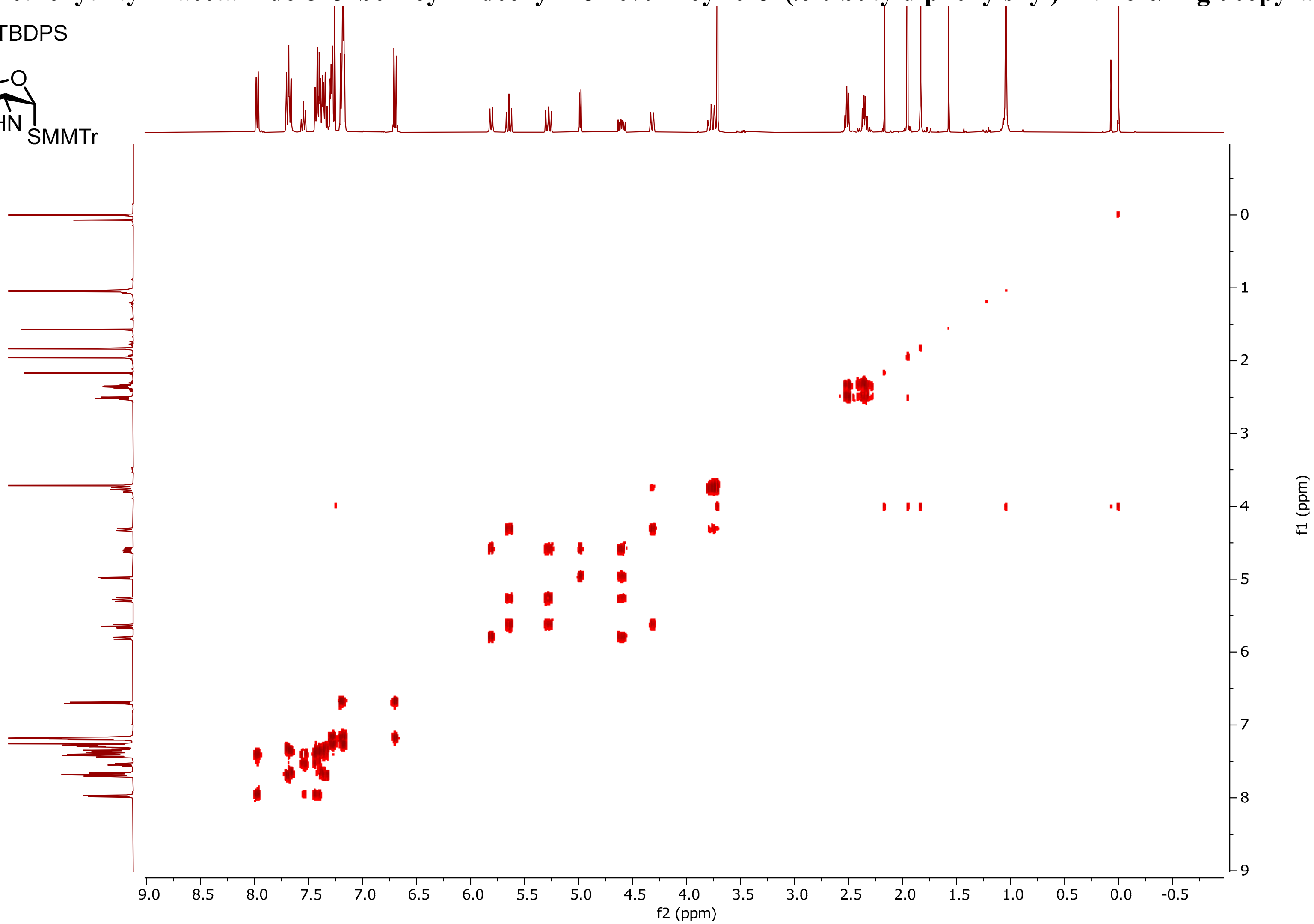

COSY (400 × 400 MHz, Chloroform-_d_)

p-Monomethoxytrityl 2-acetamido-3-O-benzoyl-2-deoxy-4-O-levulinoyl-6-O-(tert-butyldiphenylsilyl)-1-thio-α-D-glucopyranoside 33

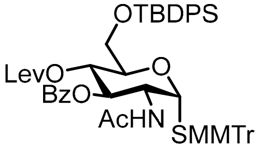

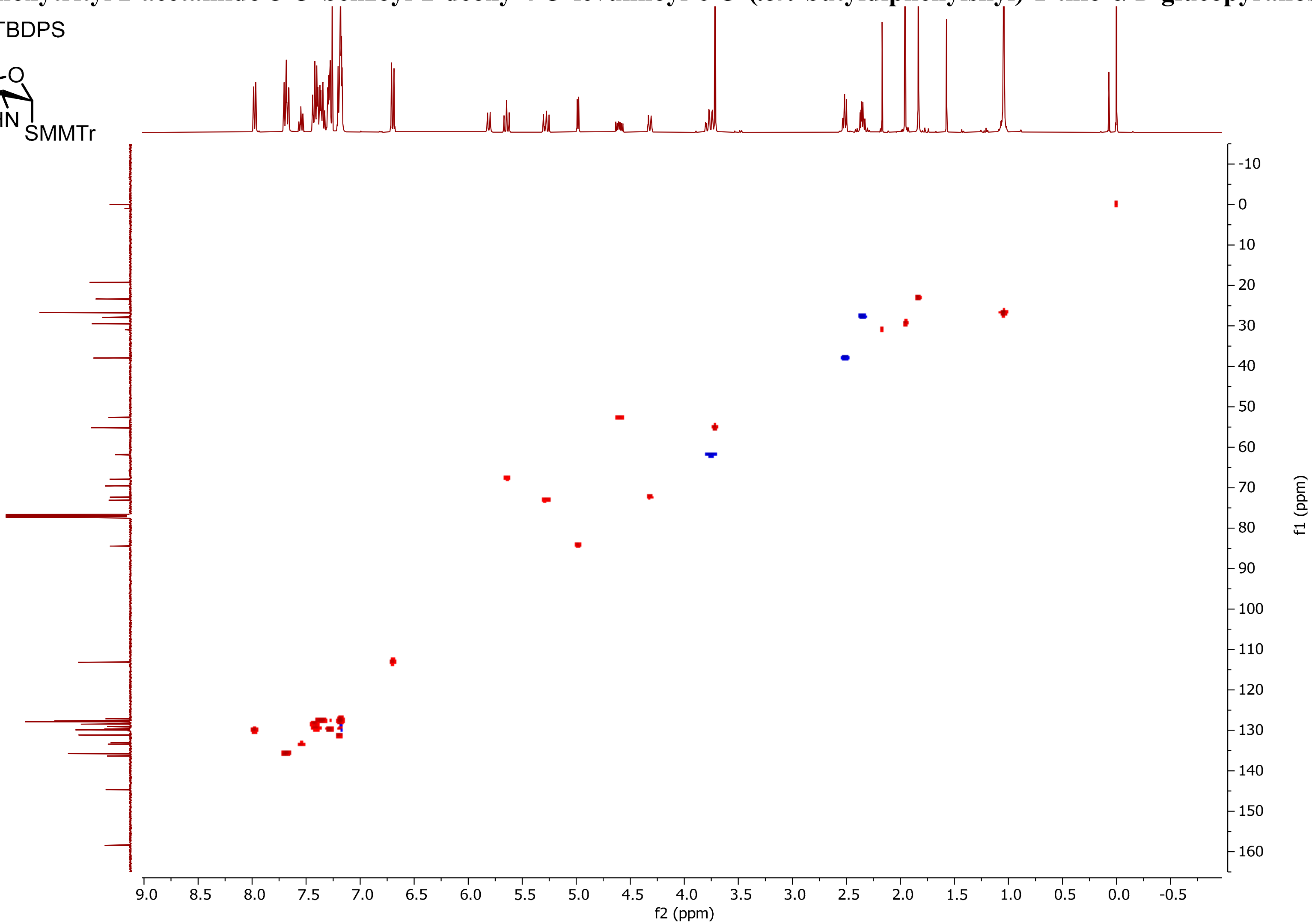

HSQC (400 × 100 MHz, Chloroform-*d*)

***p*-Monomethoxytrityl 2-acetamido-3-*O*-benzoyl-2-deoxy-4-*O*-levulinoyl-6-*O*-(*tert*-butyldiphenylsilyl)-1-thio-α-D-glucopyranoside 33**

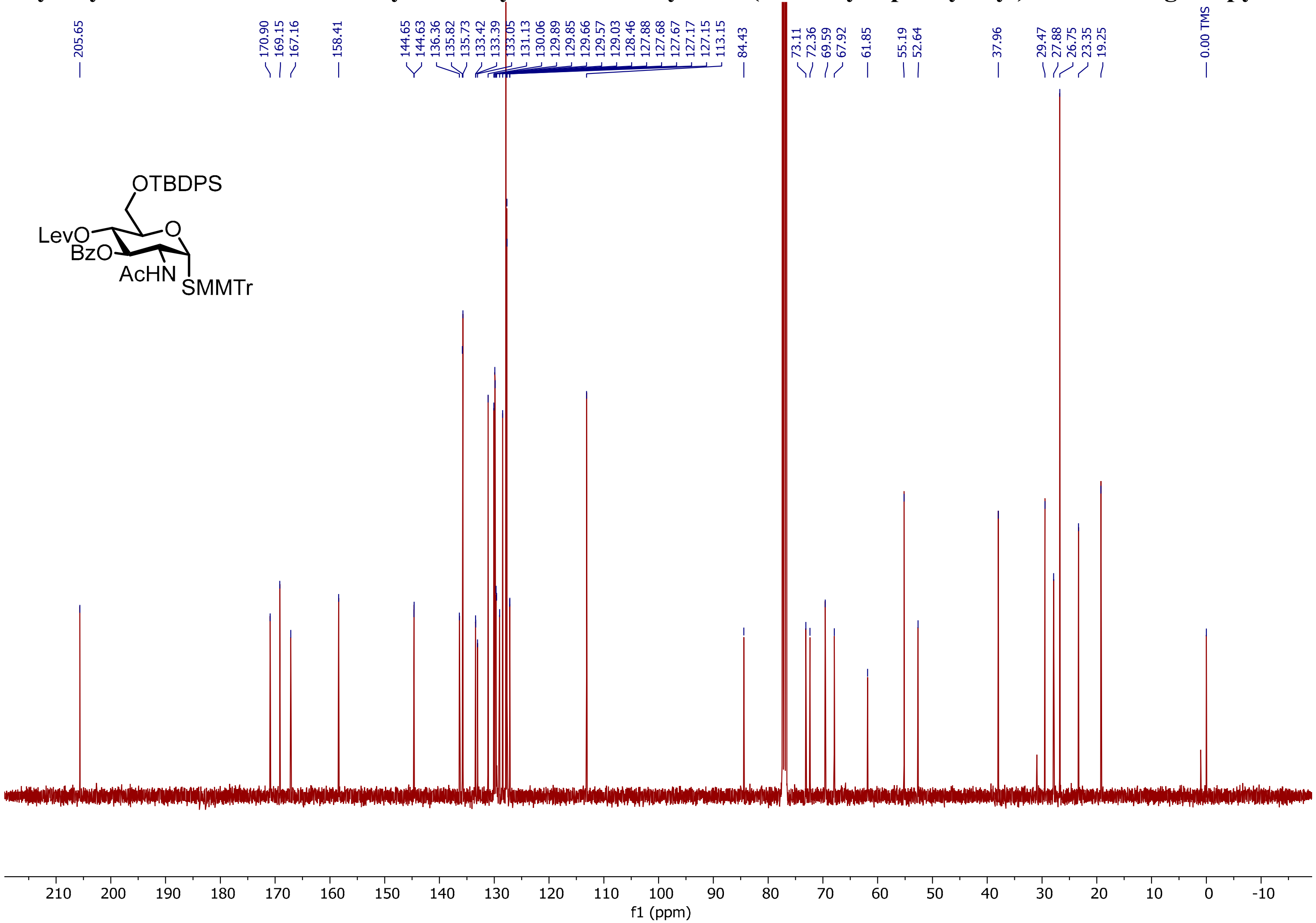

^{13}C NMR (100 MHz, Chloroform-*d*)

2-Acetamido-3-*O*-benzoyl-2-deoxy-4-*O*-levulinoyl-6-*O*-(*tert*-butyldiphenylsilyl)-1-thio-α-D-glucopyranose 35

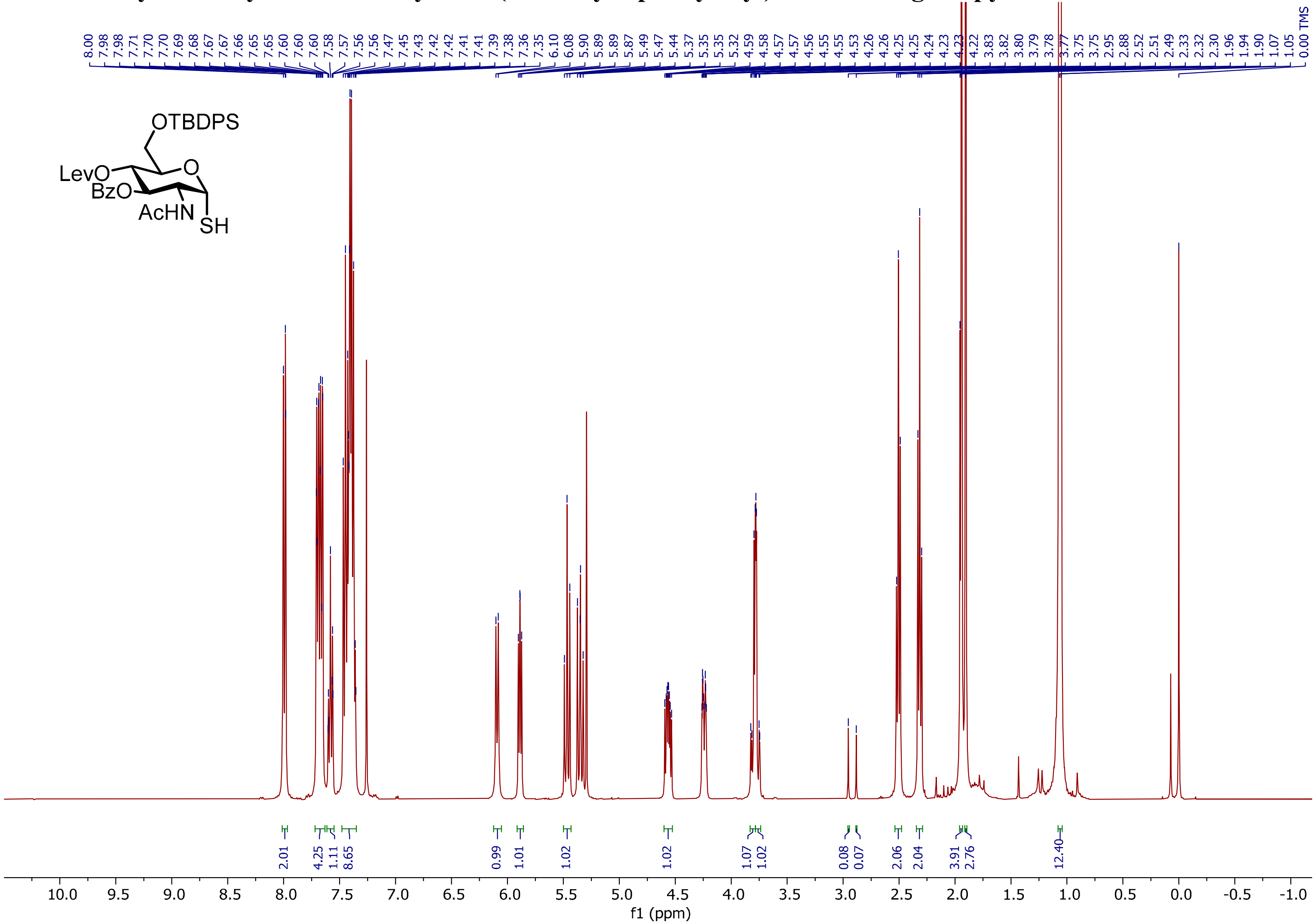

^{1}H NMR (400 MHz, Chloroform-*d*)

2-Acetamido-3-*O*-benzoyl-2-deoxy-4-*O*-levulinoyl-6-*O*-(*tert*-butyldiphenylsilyl)-1-thio-α-D-glucopyranose 35

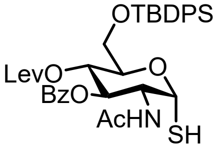

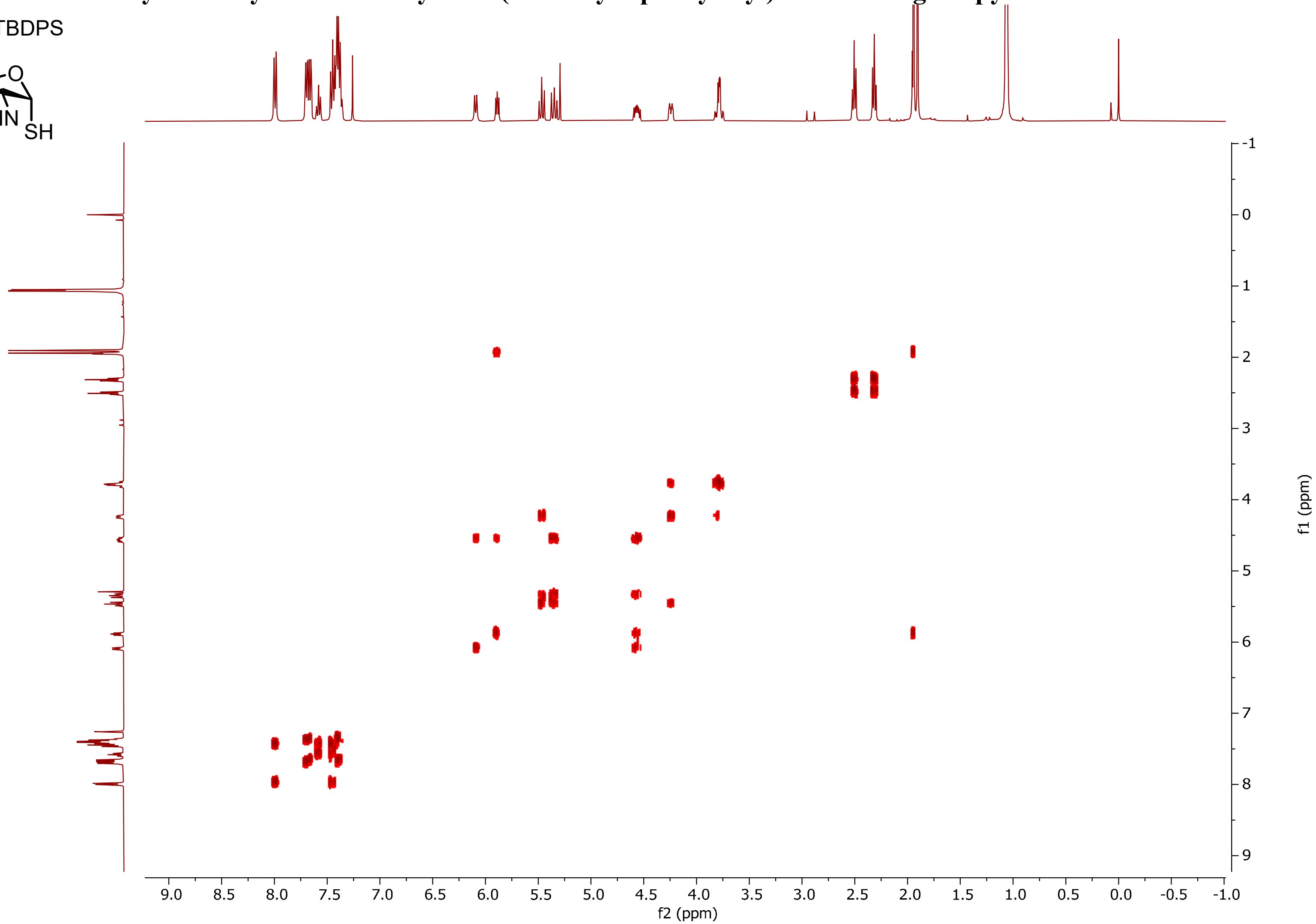

COSY (400 × 400 MHz, Chloroform-*d*)

2-Acetamido-3-*O*-benzoyl-2-deoxy-4-*O*-levulinoyl-6-*O*-(*tert*-butyldiphenylsilyl)-1-thio-α-D-glucopyranose 35

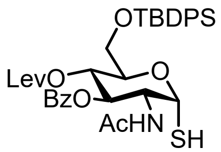

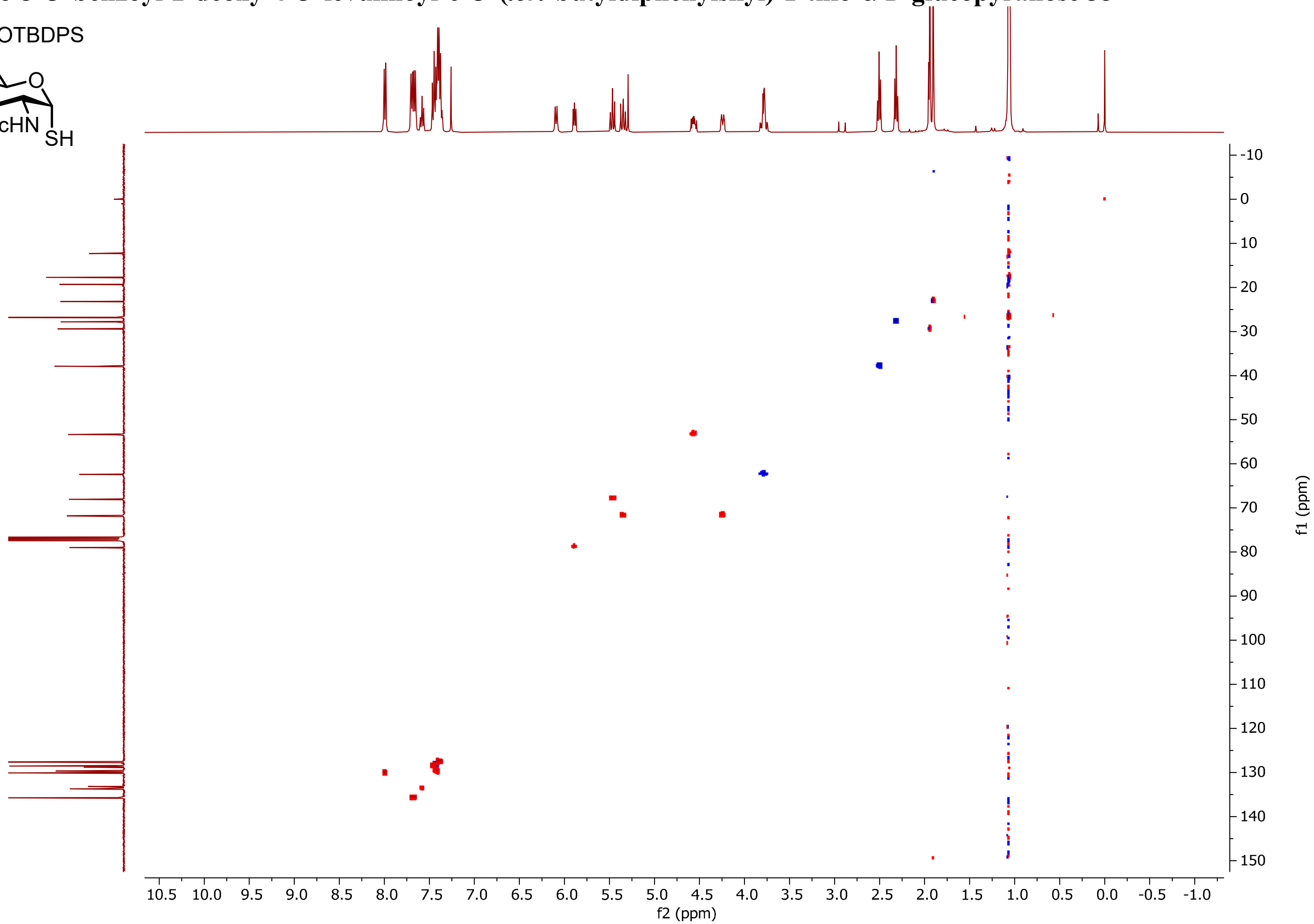

HSQC (400 × 100 MHz, Chloroform-*d*)

2-Acetamido-3-*O*-benzoyl-2-deoxy-4-*O*-levulinoyl-6-*O*-(*tert*-butyldiphenylsilyl)-1-thio-α-D-glucopyranose 35

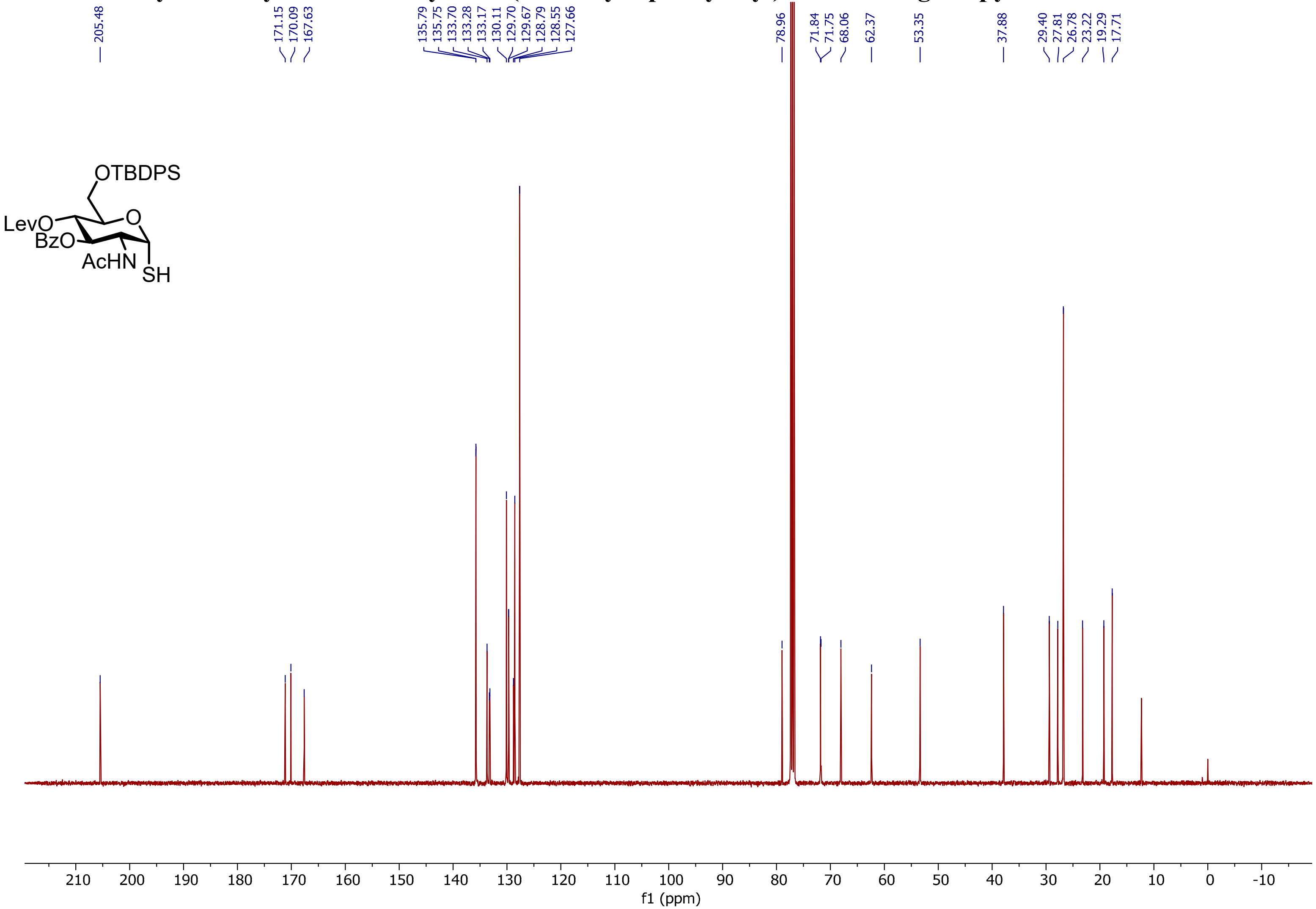

^{13}C NMR (100 MHz, Chloroform-*d*)

2-Azido-3-*O*-benzyl-2-deoxy-4-*O*-levulinoyl-6-*O*-(*tert*-butyldiphenylsilyl)-1-thio-α-D-glucopyranose 36

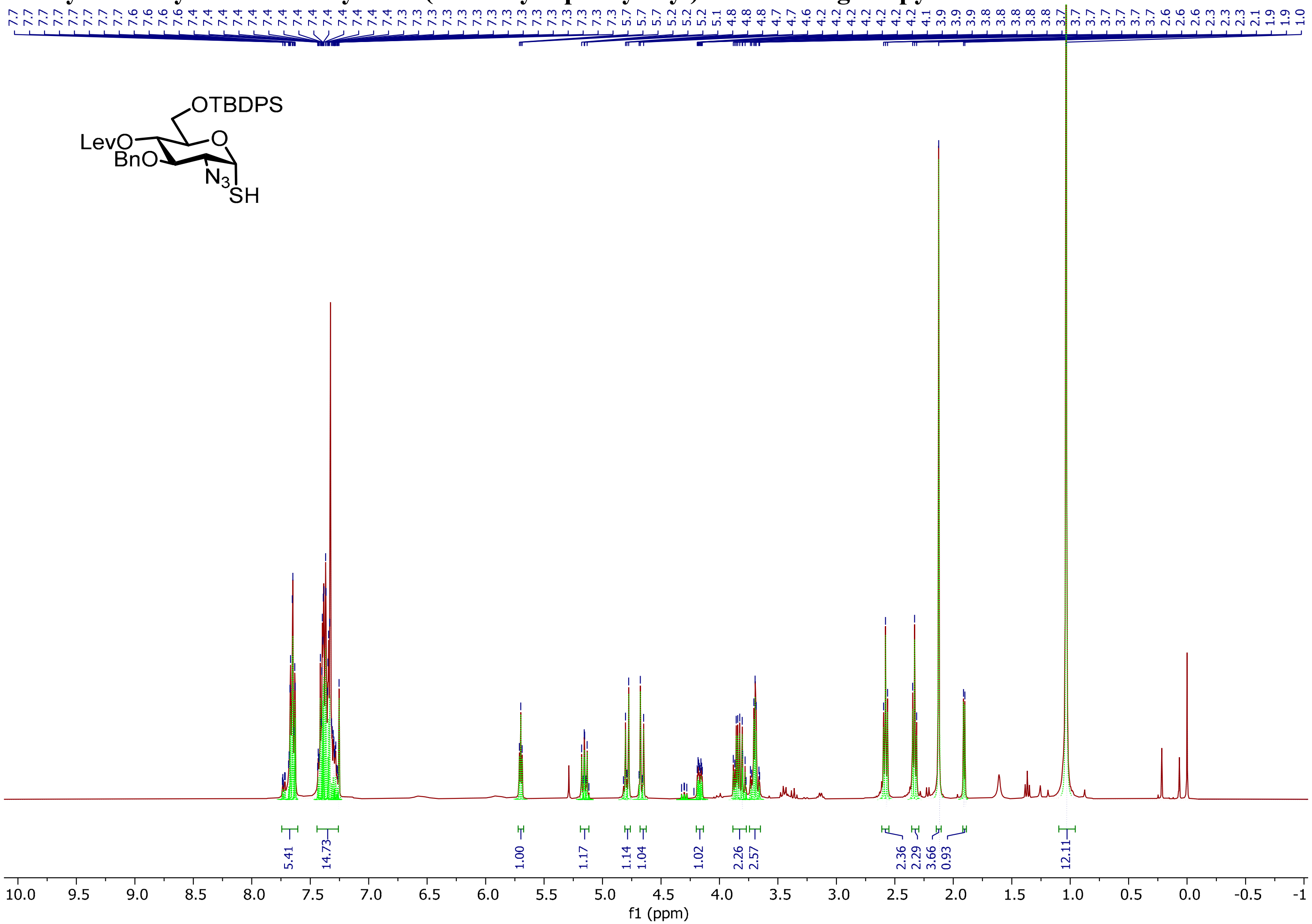

^{1}H NMR (400 MHz, Chloroform-*d*)

2-Azido-3-*O*-benzyl-2-deoxy-4-*O*-levulinoyl-6-*O*-(*tert*-butyldiphenylsilyl)-1-thio-α-D-glucopyranose 36

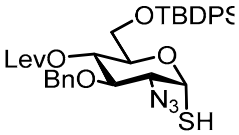

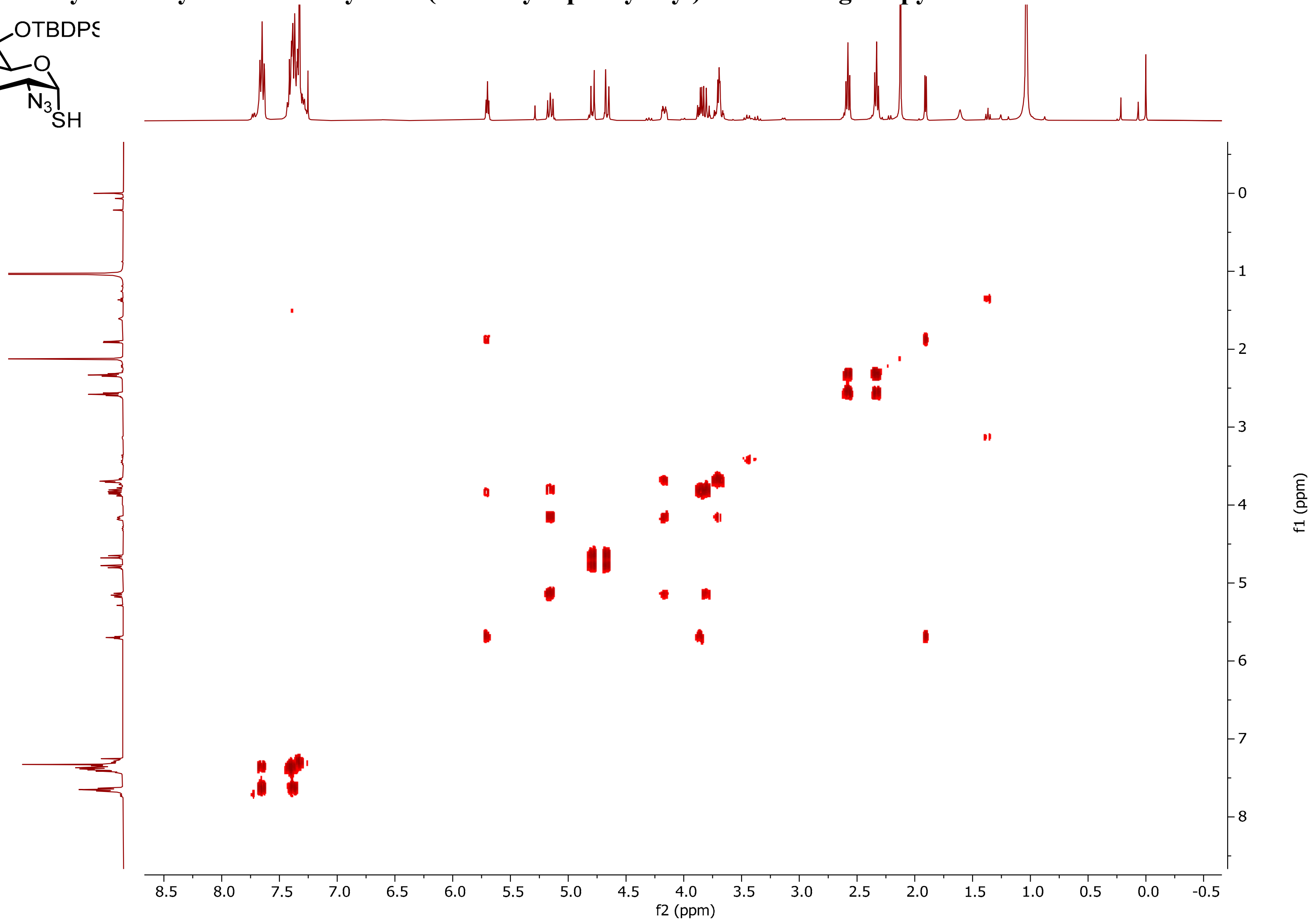

COSY (400 × 400 MHz, Chloroform-*d*)

2-Azido-3-*O*-benzyl-2-deoxy-4-*O*-levulinoyl-6-*O*-(*tert*-butyldiphenylsilyl)-1-thio-α-D-glucopyranose 36

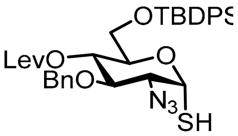

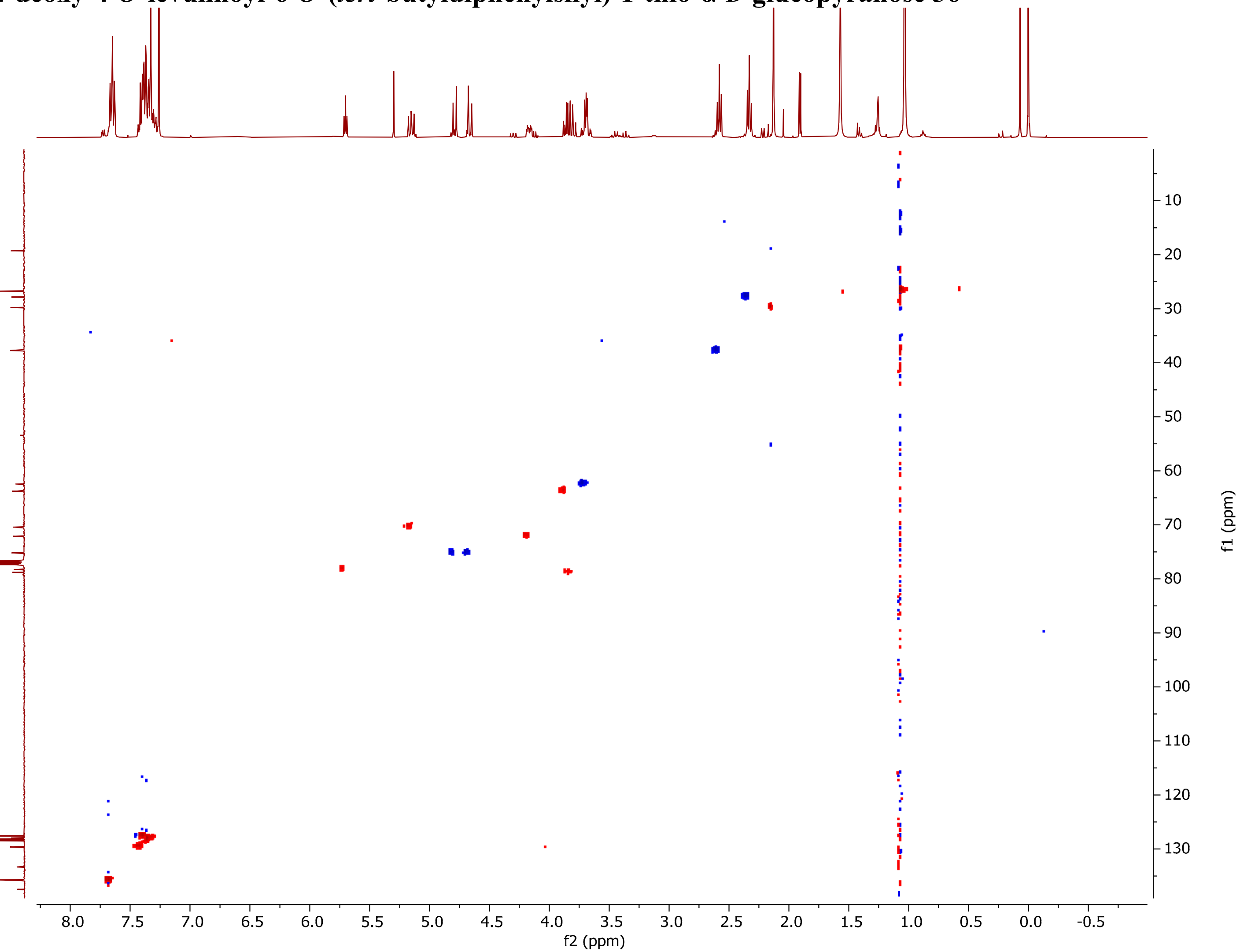

HSQC (400 × 100 MHz, Chloroform-*d*)

2-Azido-3-*O*-benzyl-2-deoxy-4-*O*-levulinoyl-6-*O*-(*tert*-butyldiphenylsilyl)-1-thio-α-D-glucopyranose 36

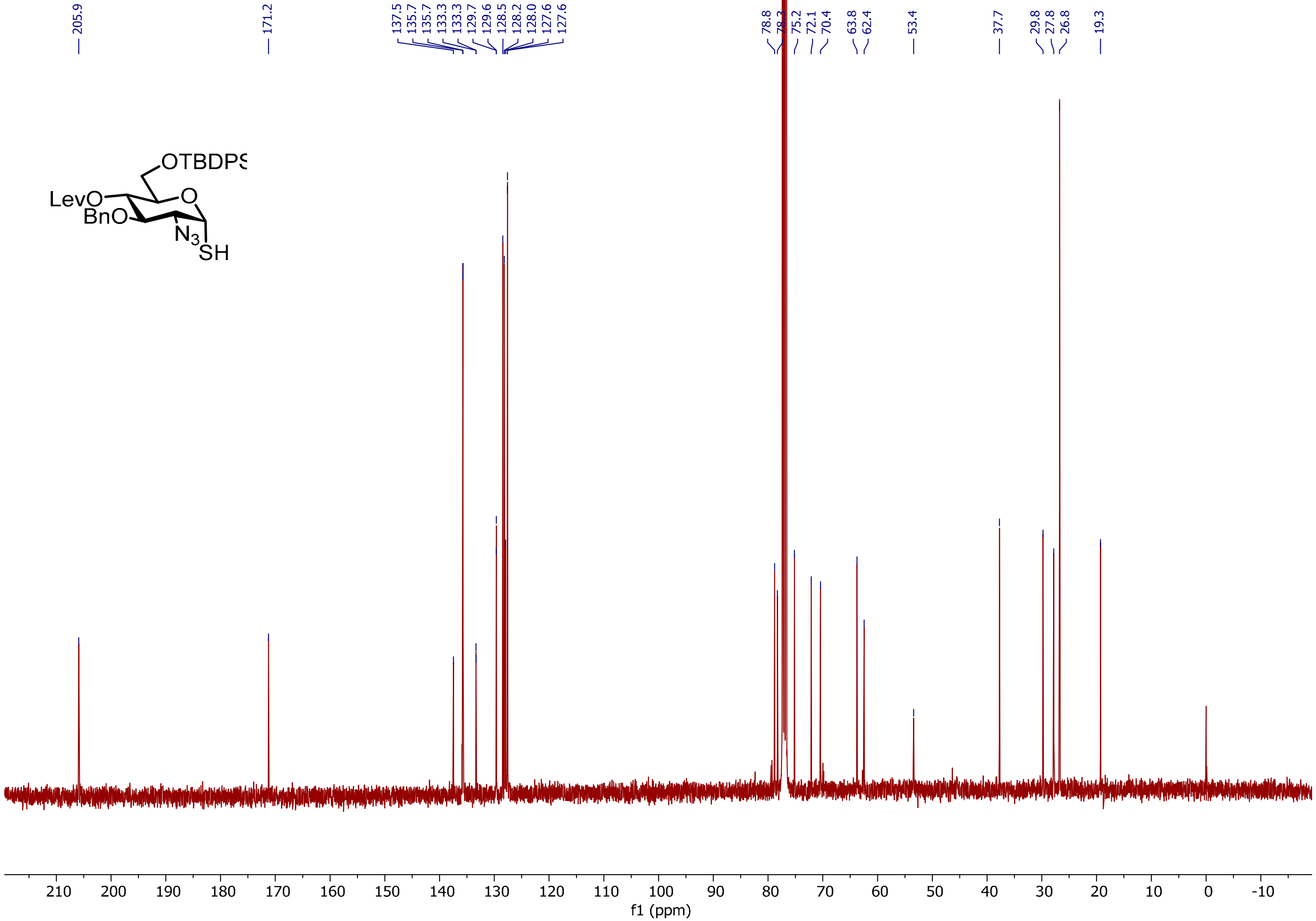

^{13}C NMR (100 MHz, Chloroform-*d*)

1-*S*-Acetyl-azido-3-*O*-benzyl-2-deoxy-4-*O*-levulinoyl-6-*O*-(*tert*-butyldiphenylsilyl)-1-thio-α-D-glucopyranose 40

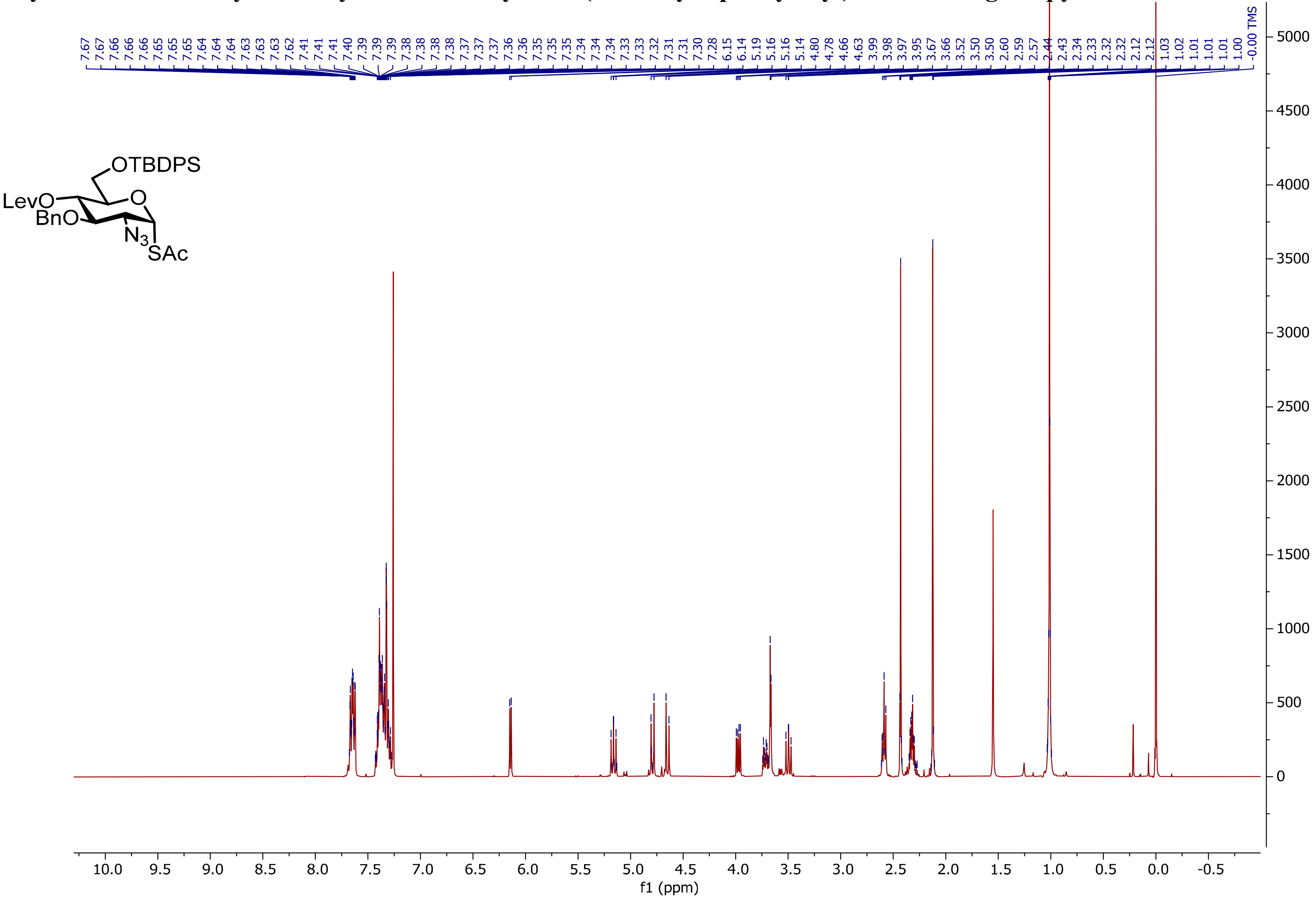

^{1}H NMR (400 MHz, Chloroform-*d*)

1-*S*-Acetyl-azido-3-*O*-benzyl-2-deoxy-4-*O*-levulinoyl-6-*O*-(*tert*-butyldiphenylsilyl)-1-thio-α-D-glucopyranose 40

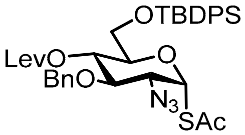

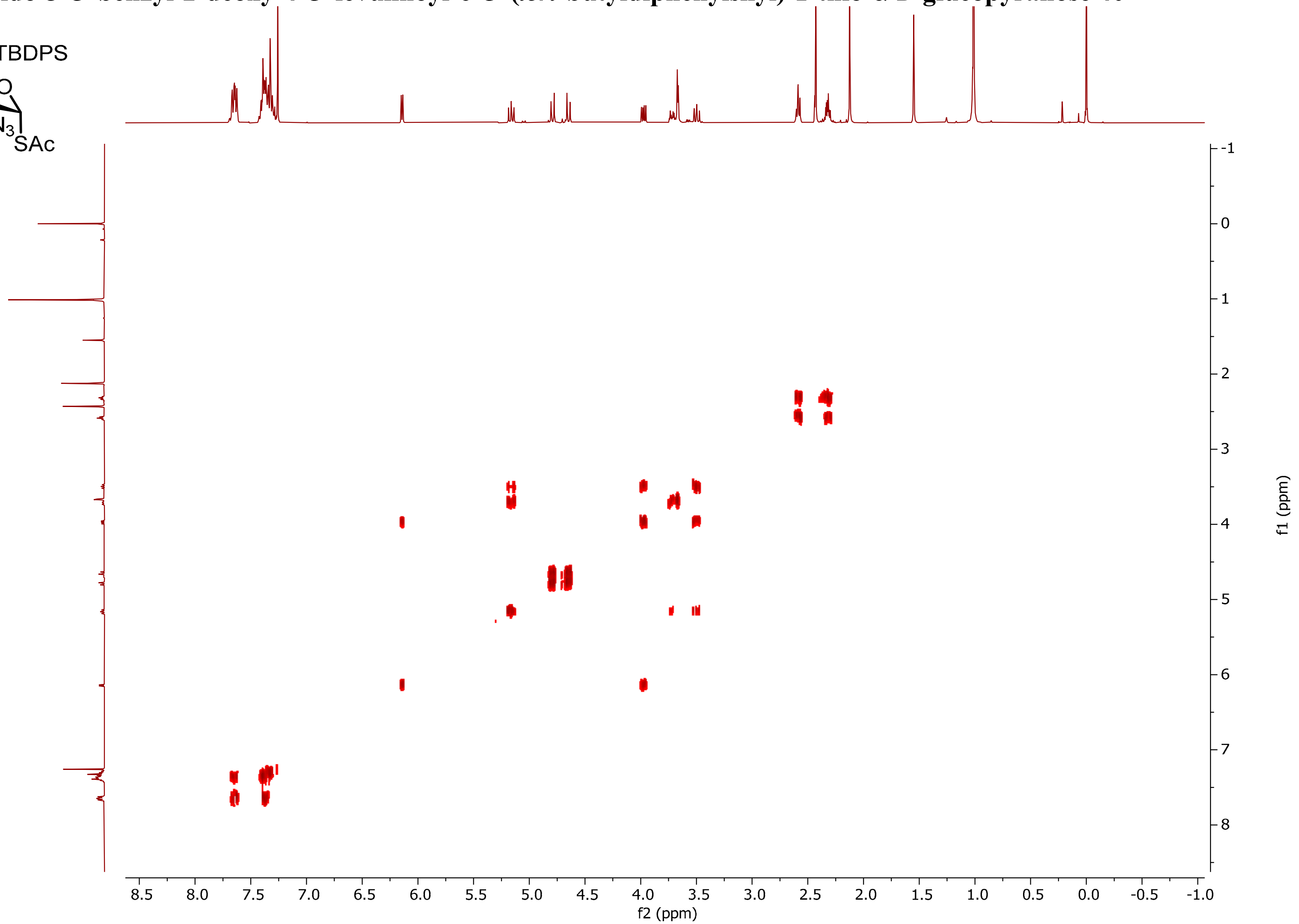

COSY (400 × 400 MHz, Chloroform-*d*)

1-*S*-Acetyl-azido-3-*O*-benzyl-2-deoxy-4-*O*-levulinoyl-6-*O*-(*tert*-butyldiphenylsilyl)-1-thio-α-D-glucopyranose 40

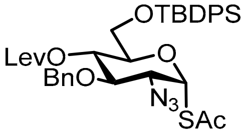

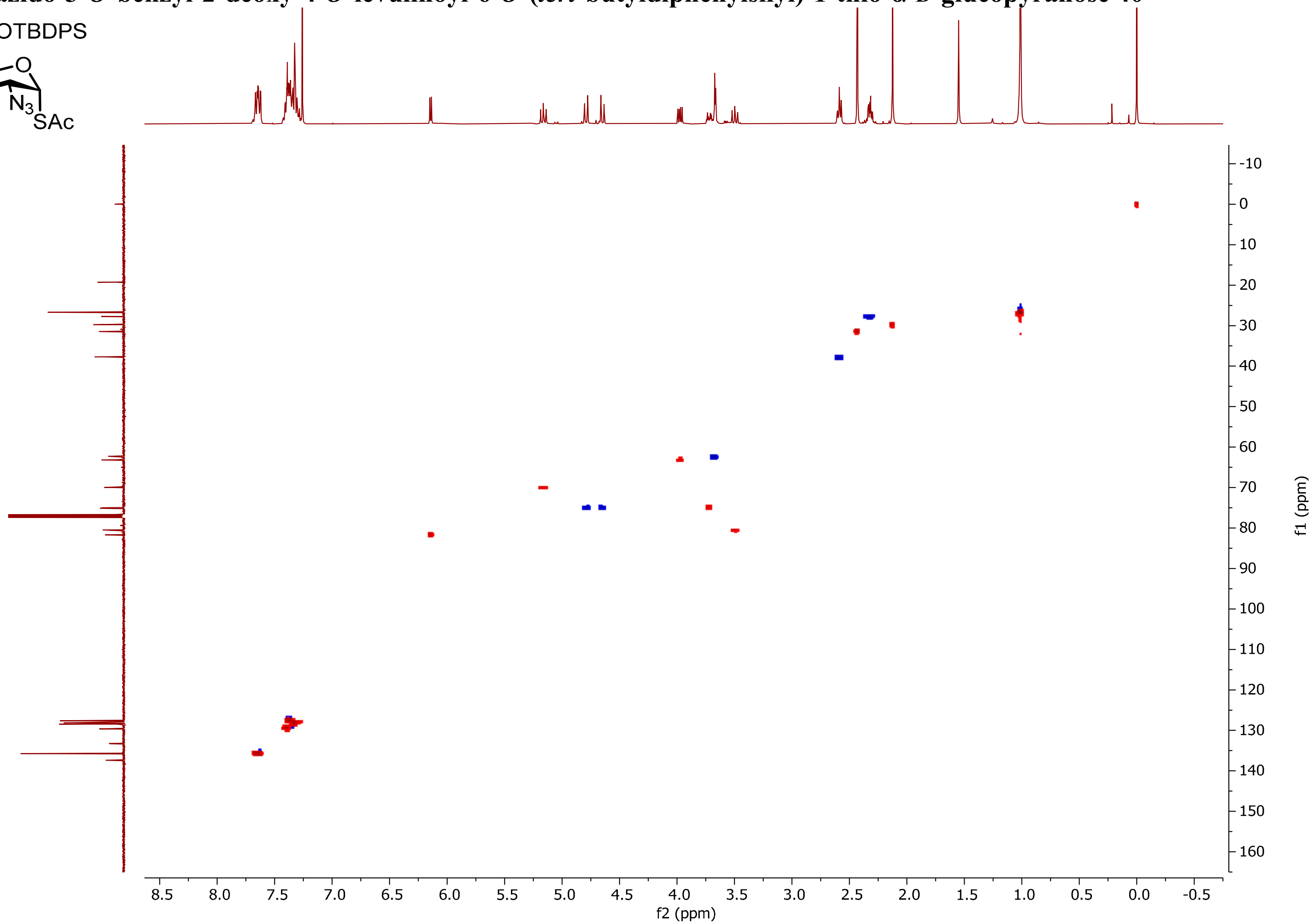

HSQC (400 × 100 MHz, Chloroform-*d*)

1-*S*-Acetyl-azido-3-*O*-benzyl-2-deoxy-4-*O*-levulinoyl-6-*O*-(*tert*-butyldiphenylsilyl)-1-thio-α-D-glucopyranose 40

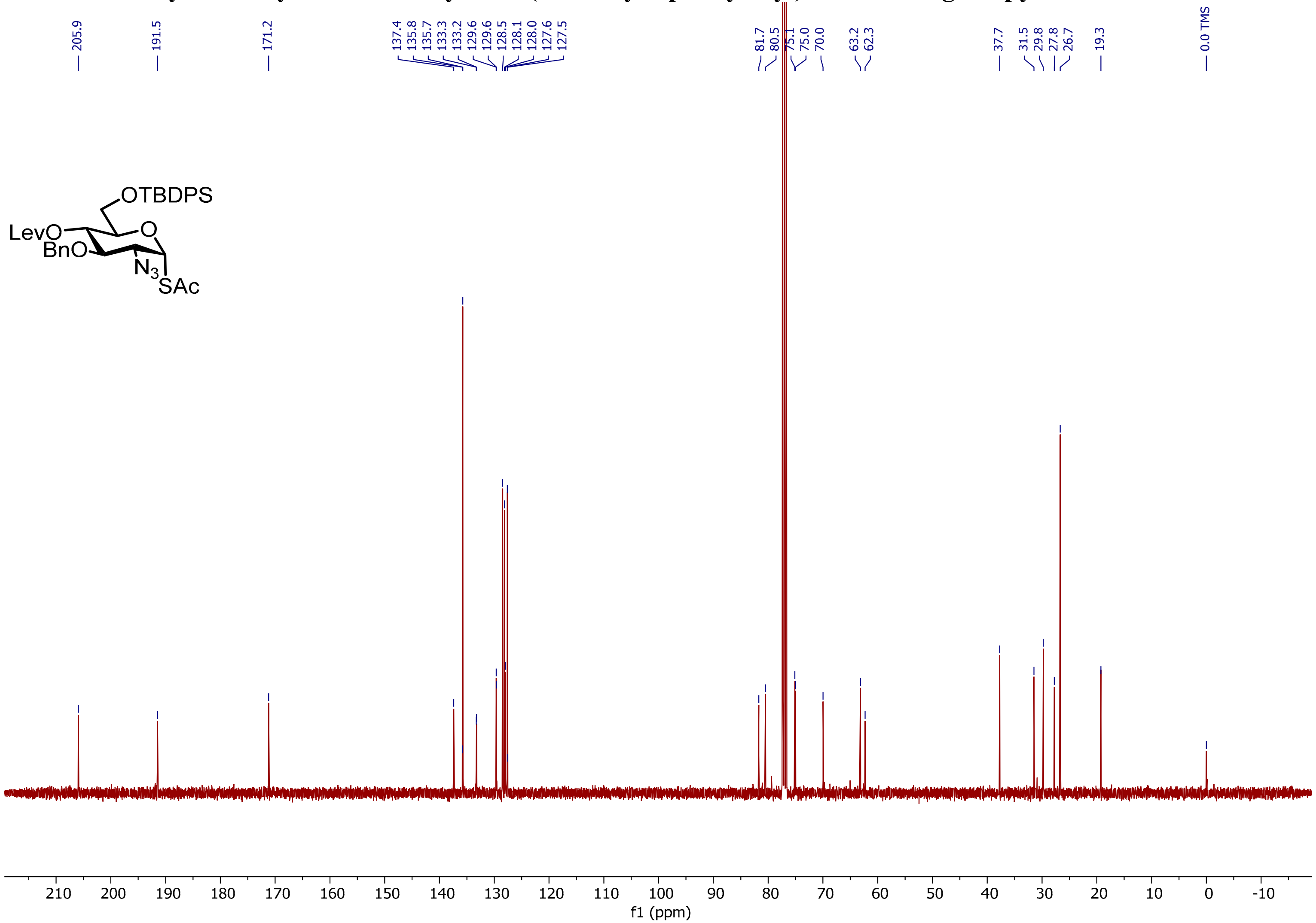

^{13}C NMR (100 MHz, Chloroform-*d*)

2-Acetamido-1-*S*-acetyl-3-*O*-benzoyl-2-deoxy-4-*O*-levulinoyl-6-*O*-(*tert*-butyldiphenylsilyl)-1-thio-α-D-glucopyranose 39

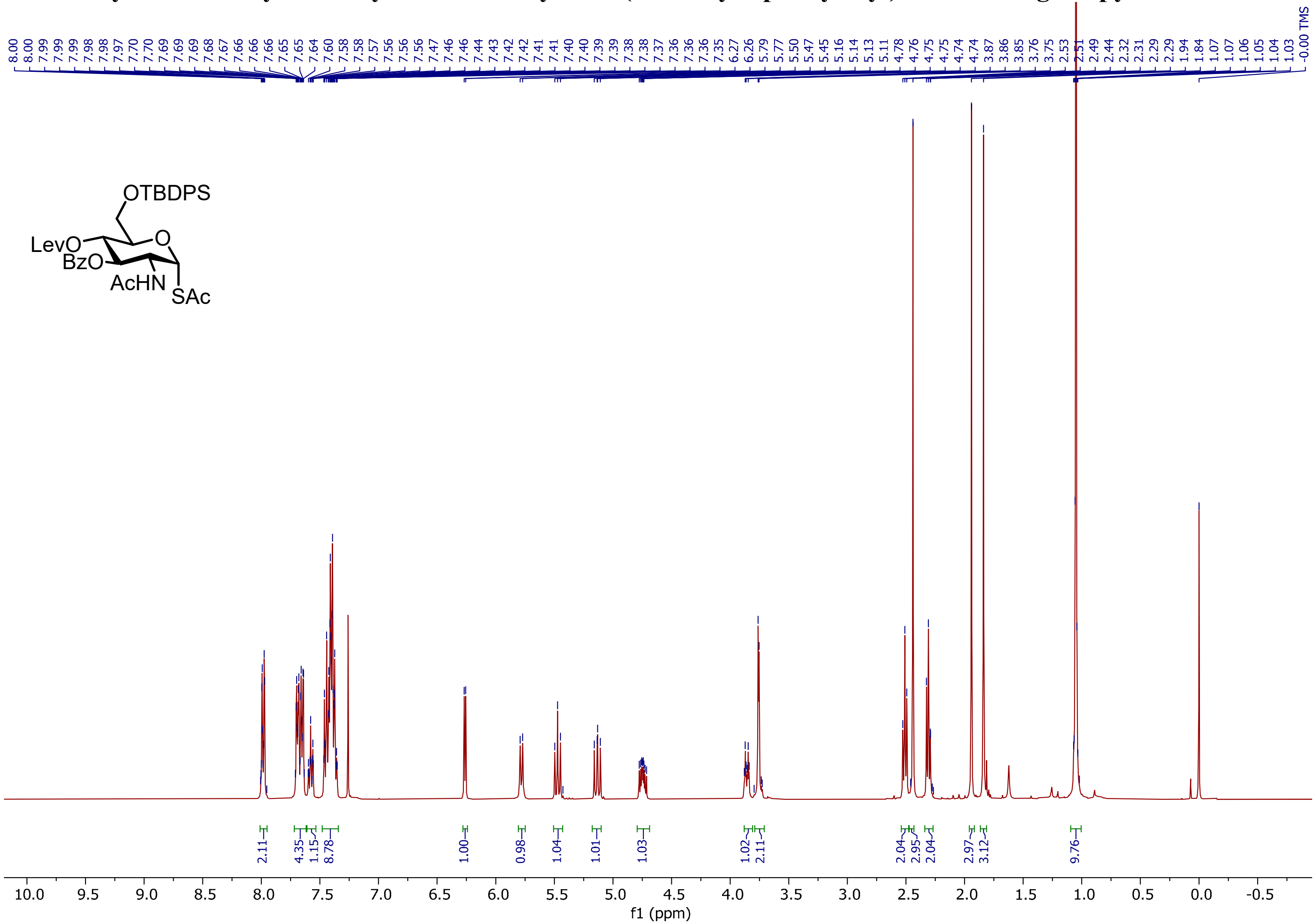

^{1}H NMR (Chloroform-*d*, 400 MHz)

2-Acetamido-1-*S*-acetyl-3-*O*-benzoyl-2-deoxy-4-*O*-levulinoyl-6-*O*-(*tert*-butyldiphenylsilyl)-1-thio-α-D-glucopyranose 39

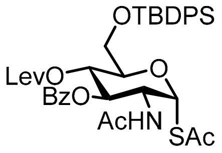

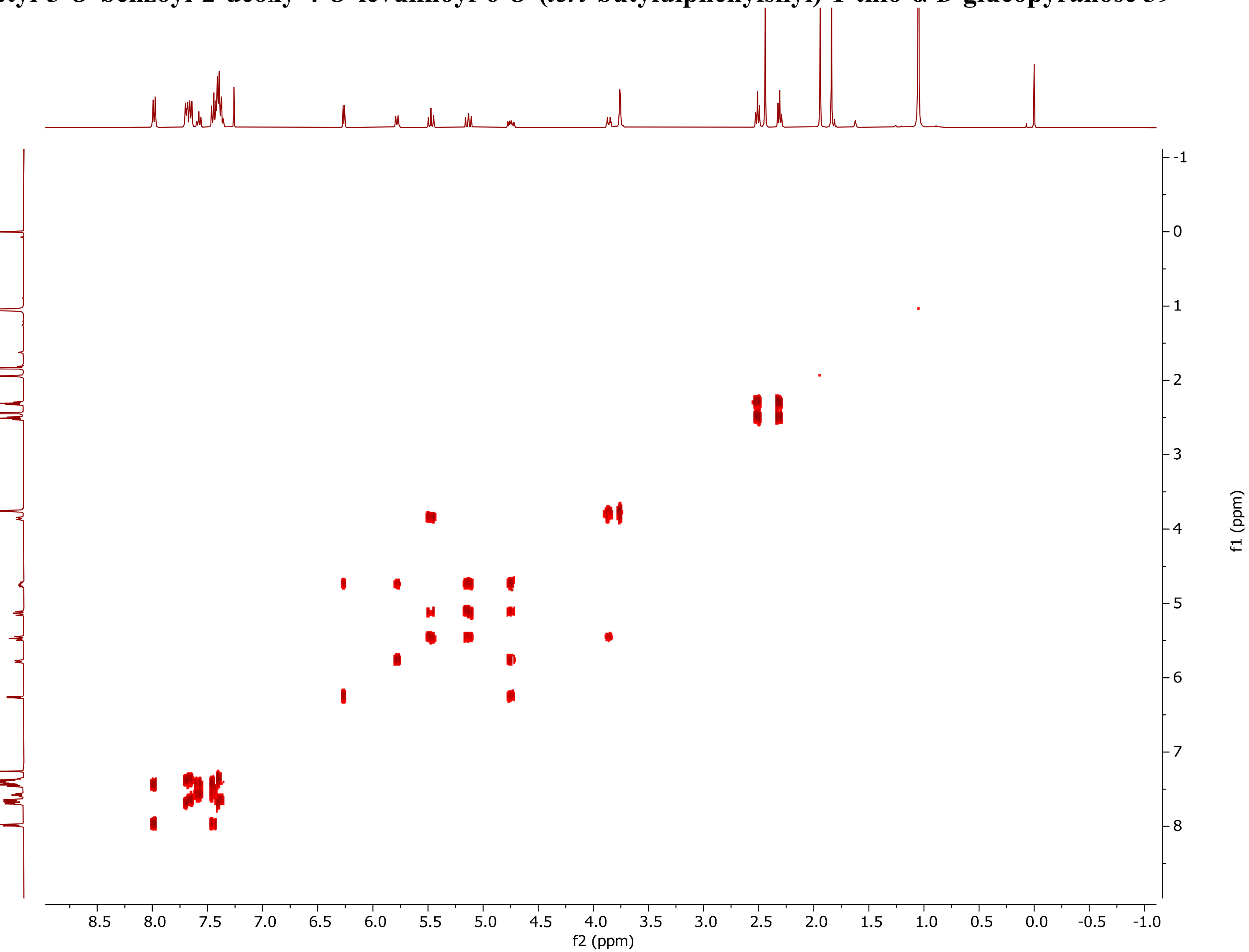

COSY (400 × 400 MHz, Chloroform-*d*)

2-Acetamido-1-*S*-acetyl-3-*O*-benzoyl-2-deoxy-4-*O*-levulinoyl-6-*O*-(*tert*-butyldiphenylsilyl)-1-thio-α-D-glucopyranose 39

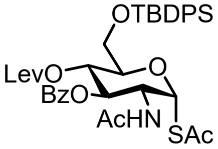

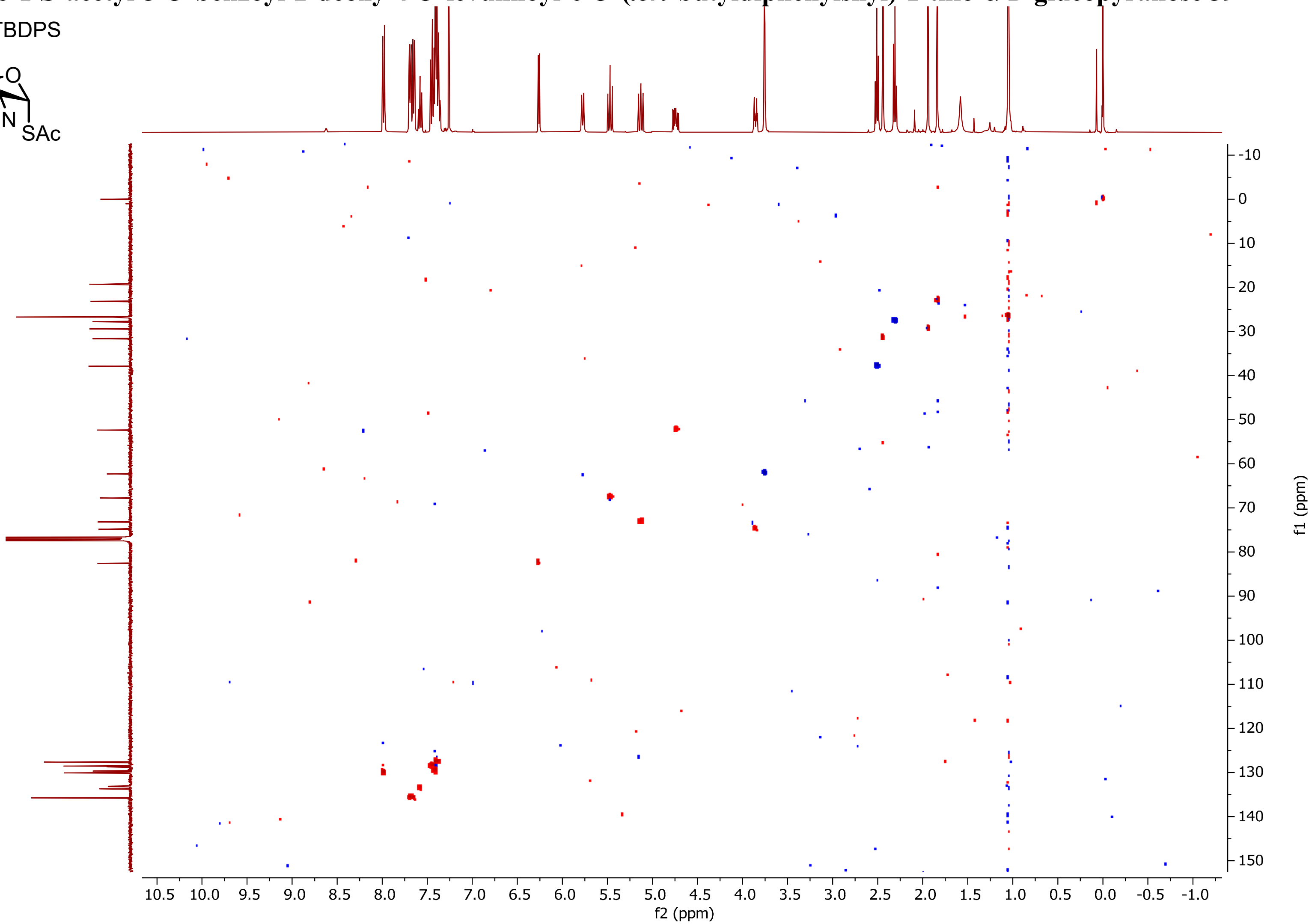

HSQC (400 × 100 MHz, Chloroform-*d*)

2-Acetamido-1-*S*-acetyl-3-*O*-benzoyl-2-deoxy-4-*O*-levulinoyl-6-*O*-(*tert*-butyldiphenylsilyl)-1-thio-α-D-glucopyranose 39

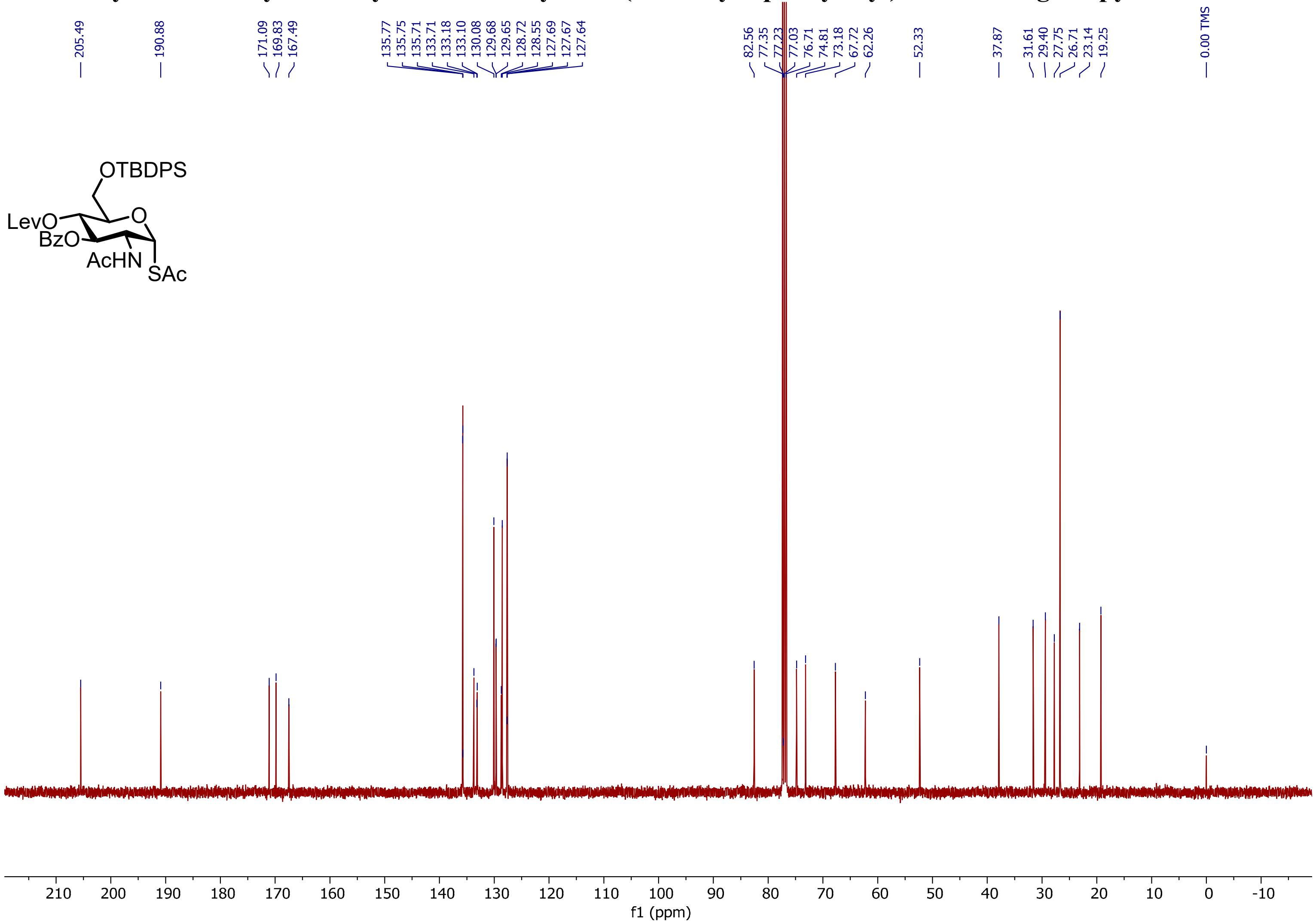

^{13}C NMR (Chloroform-*d*, 100 MHz)

S-linked Disaccharides

_S_-(2-Acetamido-3,6-di-_O_-benzoyl-2-deoxy-4-_O_-levulinoyl-α-D-glucopyranosyl)- (1→4)- _p_-methoxyphenyl 2,3-di-_O_-benzoyl-4-thio-6-O-(triisopropylsilyl)-β-D-glucopyranoside 37

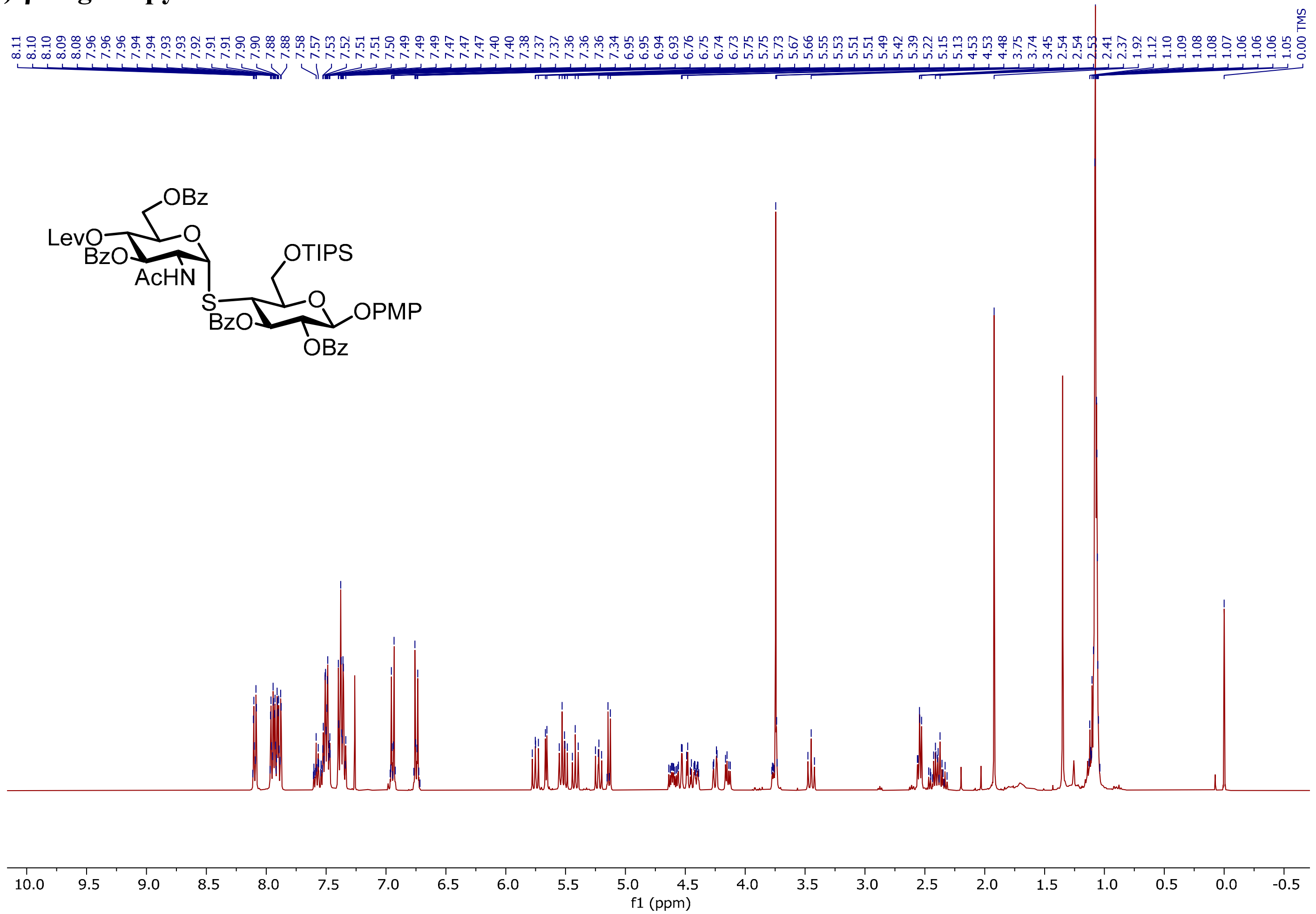

^{1}H NMR (400 MHz, Chloroform-*d*)

***S*-(2-Acetamido-3,6-di-*O*-benzoyl-2-deoxy-4-*O*-levulinoyl-α-D-glucopyranosyl)- (1→4)- *p*-methoxyphenyl 2,3-di-*O*-benzoyl-4-thio-6-O-(triisopropylsilyl)-β-D-glucopyranoside 37**

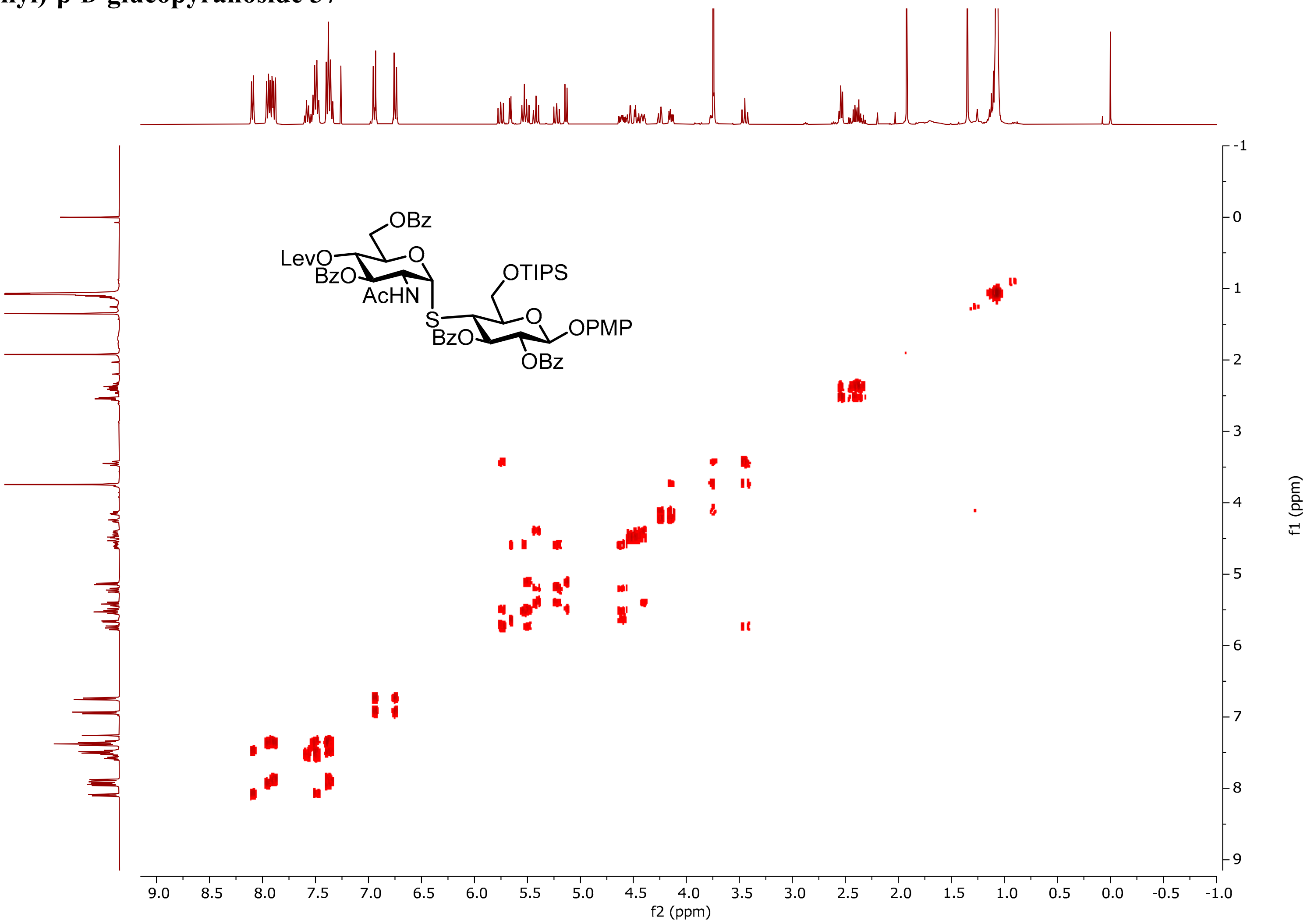

COSY (400 × 400 MHz, Chloroform-*d*)

S-(2-Acetamido-3,6-di-O-benzoyl-2-deoxy-4-O-levulinoyl-α-D-glucopyranosyl)- (1→4)- p-methoxyphenyl 2,3-di-O-benzoyl-4-thio-6-O-(triisopropylsilyl)-β-D-glucopyranoside 37

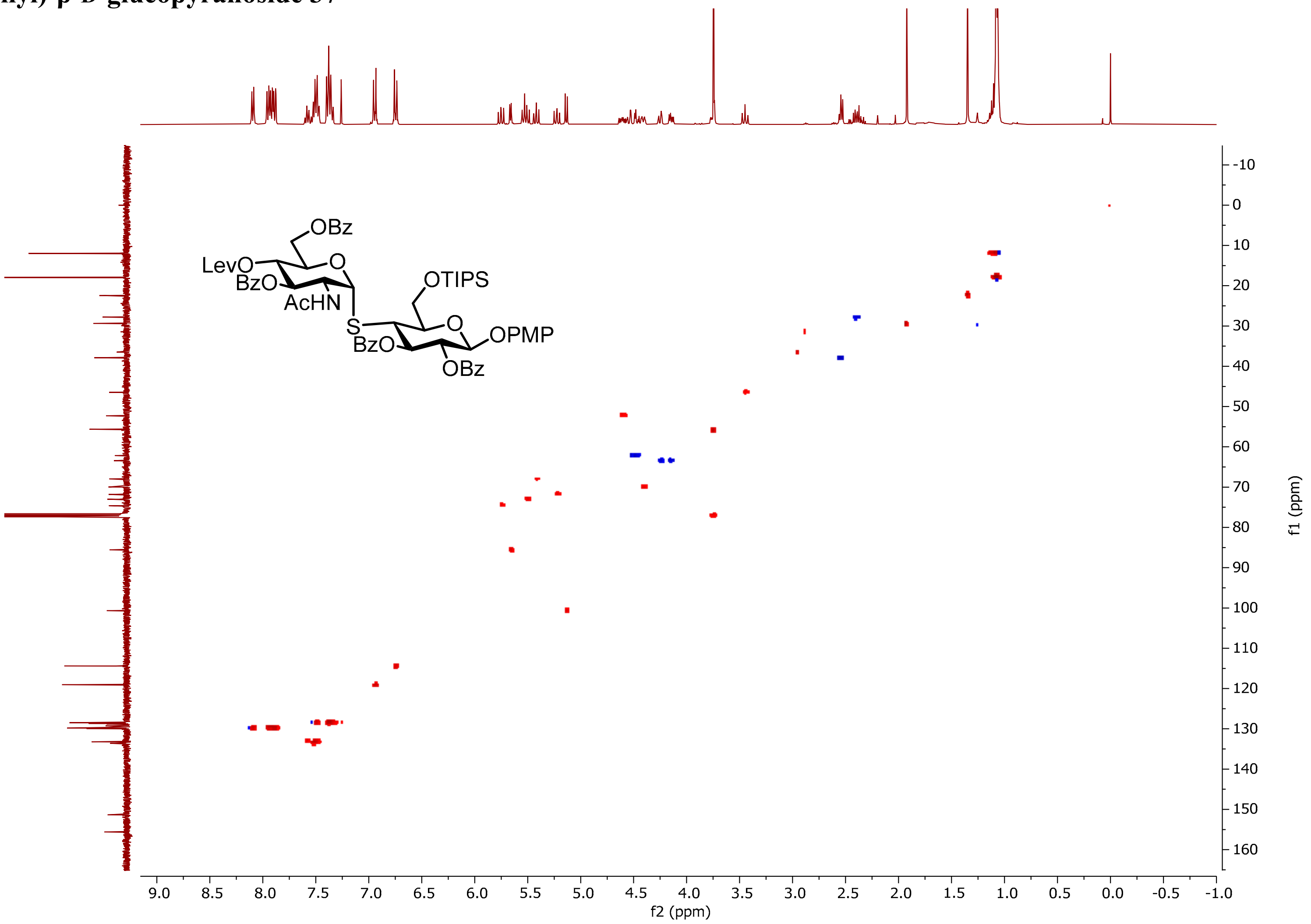

HSQC (400 × 100 MHz, Chloroform-*d*)

S-(2-Acetamido-3,6-di-O-benzoyl-2-deoxy-4-O-levulinoyl-α-D-glucopyranosyl)- (1→4)- p-methoxyphenyl 2,3-di-O-benzoyl-4-thio-6-O-(triisopropylsilyl)-β-D-glucopyranoside 37

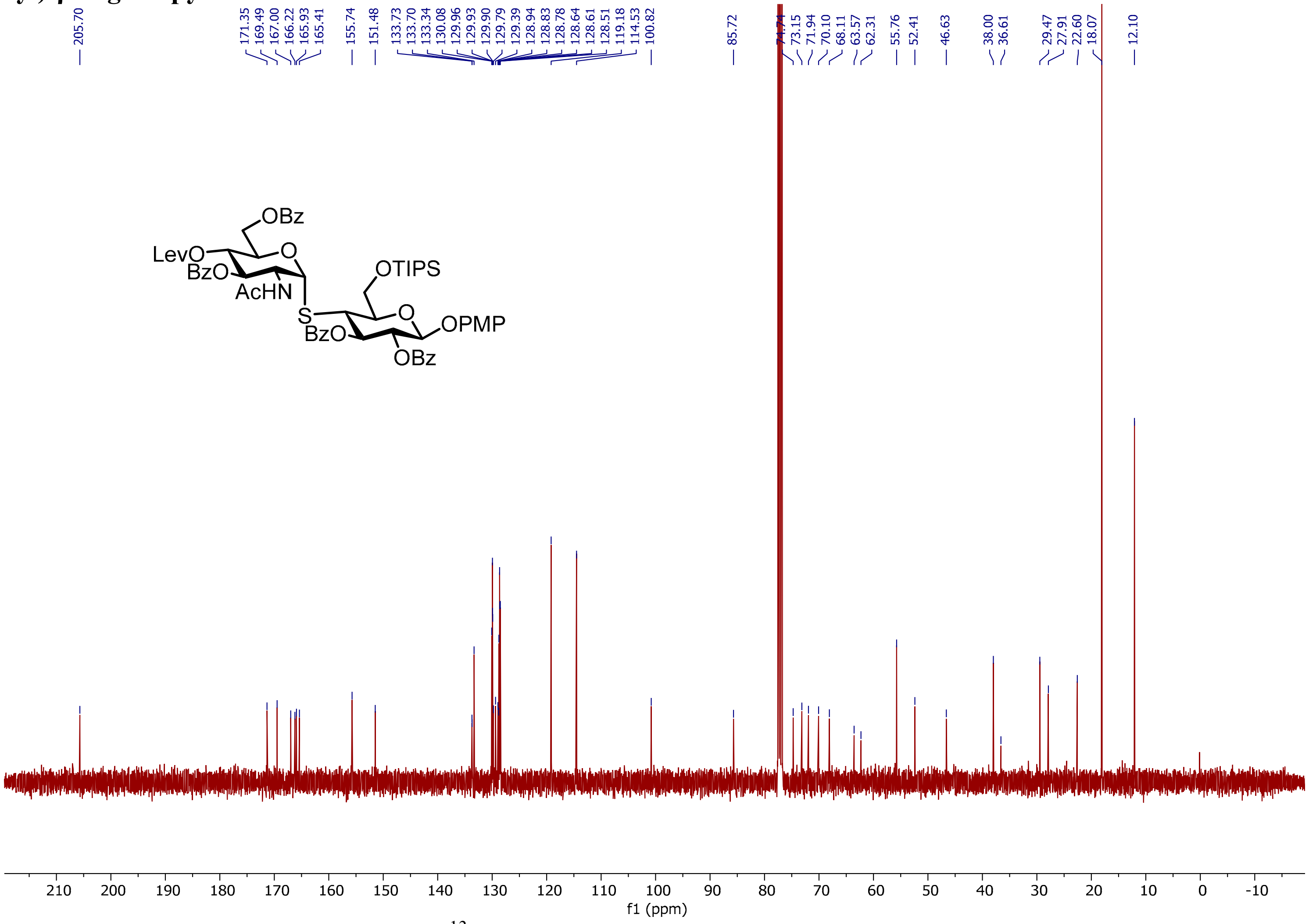

^{13}C NMR (100 MHz, Chloroform-*d*)

***S*-(2-Azido-3-*O*-benzyl-2-deoxy-4-*O*-levulinoyl-6-*O*-(*tert*-butyldiphenylsilyl)-α-D-glucopyranosyl)- (1→4)- *p*-methoxyphenyl 2,3-di-*O*-benzoyl-4-thio-6-*O*-(triisopropylsilyl)-β-D-glucopyranoside 38**

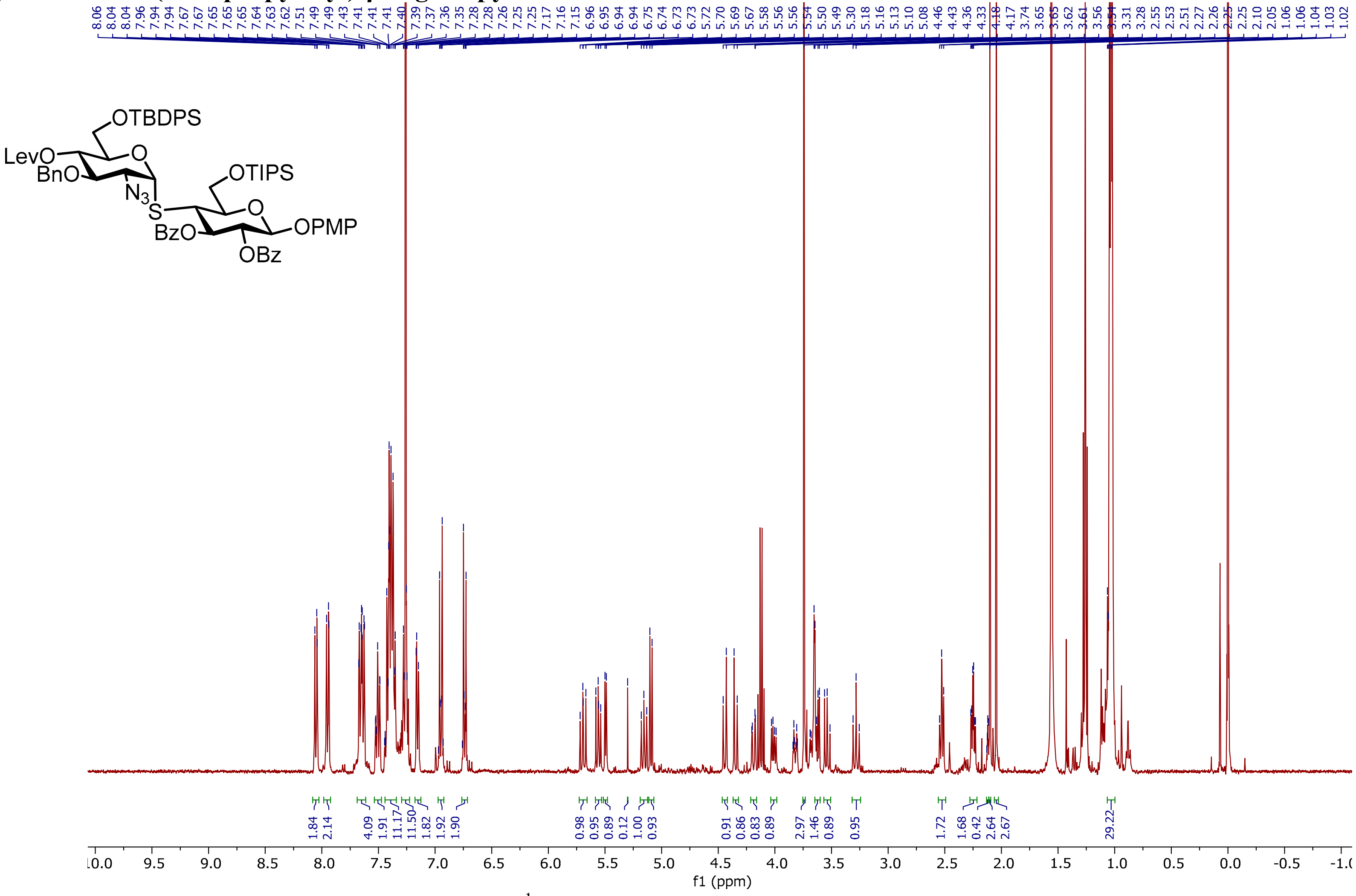

^{1}H NMR (400 MHz, Chloroform-*d*)

S-(2-Azido-3-O-benzyl-2-deoxy-4-O-levulinoyl-6-O-(tert-butyldiphenylsilyl)-α-D-glucopyranosyl)- (1→4)- p-methoxyphenyl 2,3-di-O-benzoyl-4-thio-6-O-(triisopropylsilyl)-β-D-glucopyranoside 38

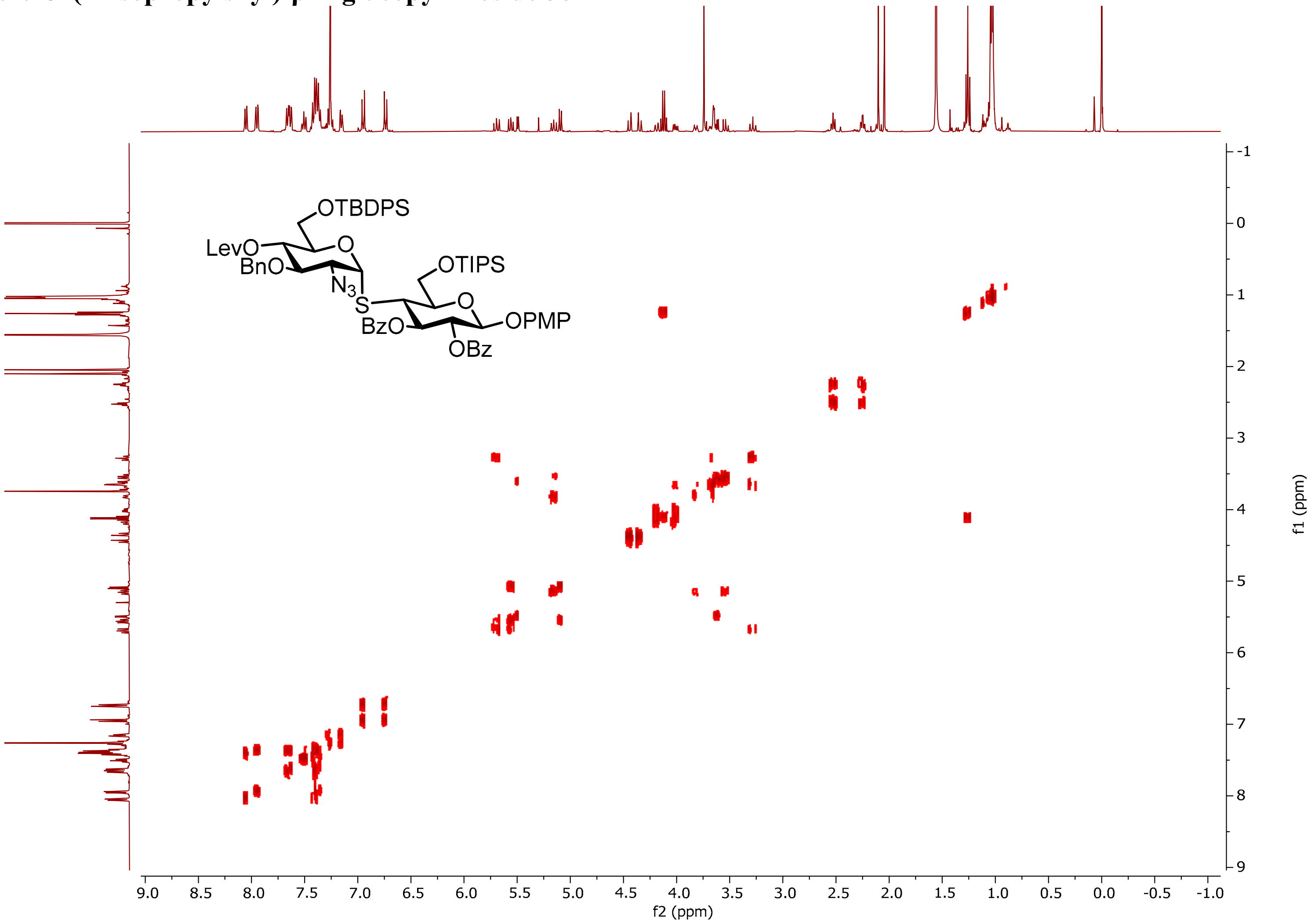

COSY (400 × 400 MHz, Chloroform-*d*)

***S*-(2-Azido-3-*O*-benzyl-2-deoxy-4-*O*-levulinoyl-6-*O*-(*tert*-butyldiphenylsilyl)-α-D-glucopyranosyl)- (1→4)- *p*-methoxyphenyl 2,3-di-*O*-benzoyl-4-thio-6-*O*-(triisopropylsilyl)-β-D-glucopyranoside 38**

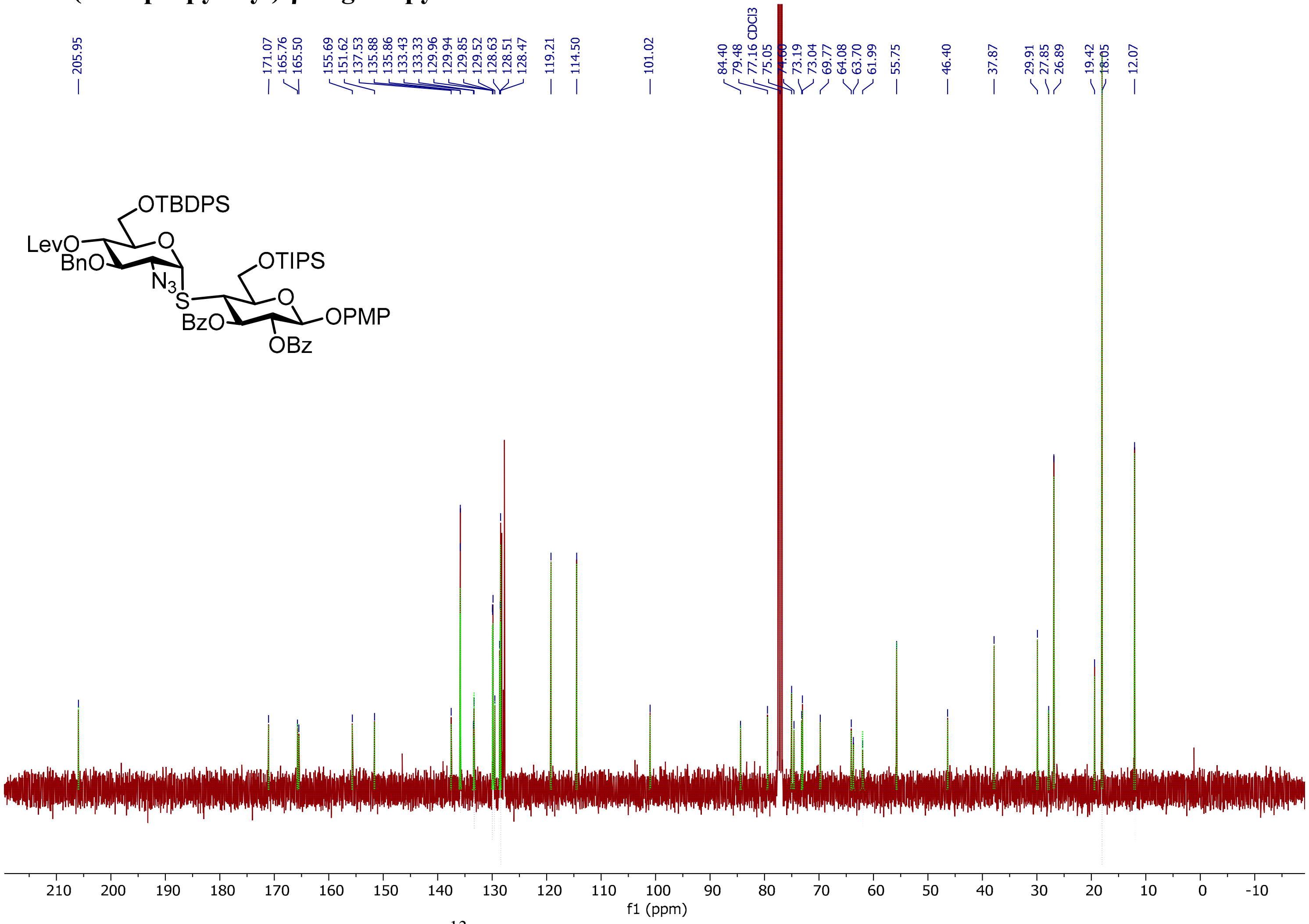

^{13}C NMR (100 MHz, Chloroform-*d*)

***S*-(2-Acetamido-2-deoxy-3-*O*-benzoyl-4-*O*-levulinoyl-6-*O*-(*tert*-butyldiphenylsilyl)-α-D-glucopyranosyl)- (1→4)- *p*-methoxyphenyl 2,3-di-*O*-benzoyl-4-thio-6-O-(triisopropylsilyl)-β-D-glucopyranoside 41**

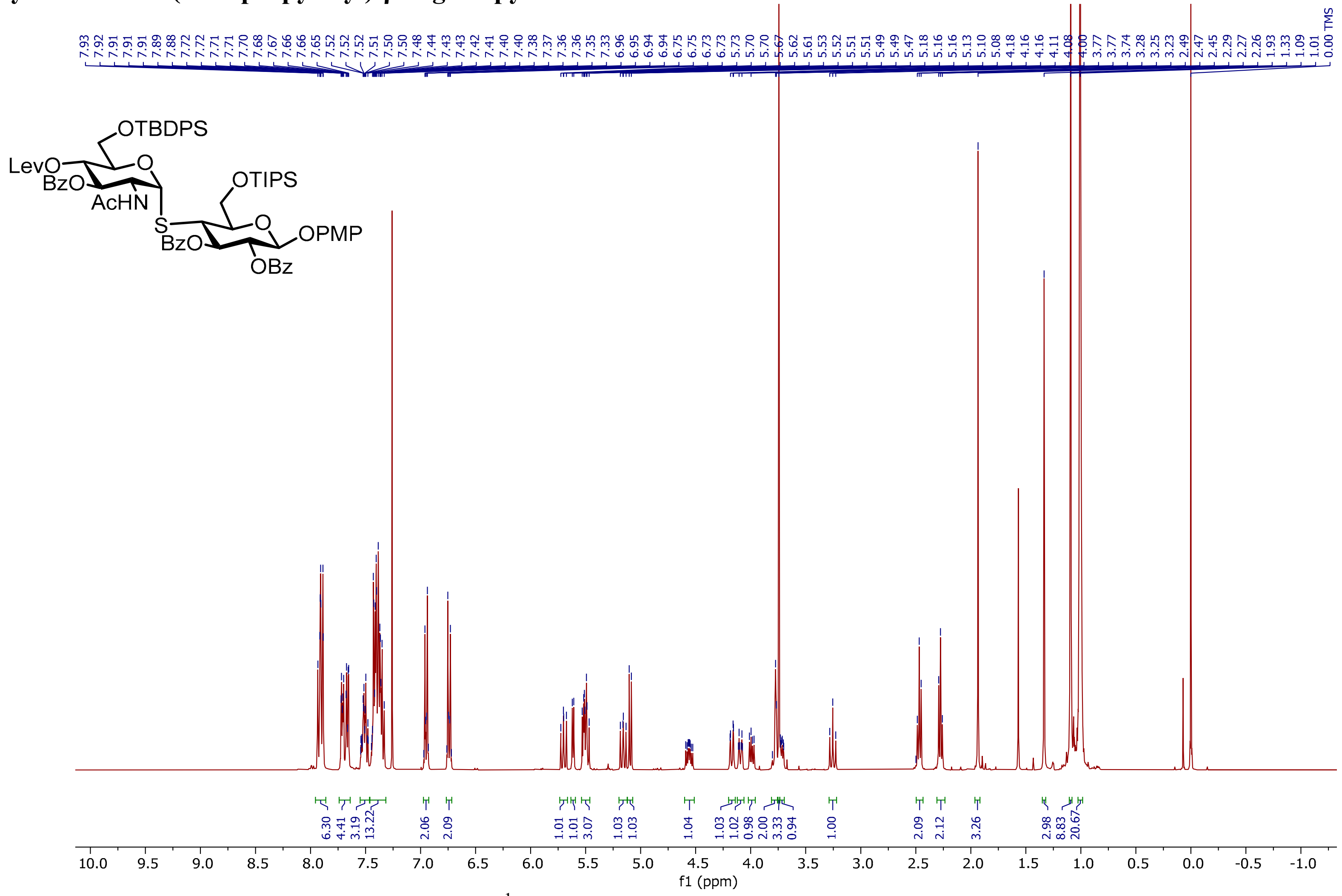

^{1}H NMR (400 MHz, Chloroform-*d*,)

***S*-(2-Acetamido-2-deoxy-3-*O*-benzoyl-4-*O*-levulinoyl-6-*O*-(*tert*-butyldiphenylsilyl)-α-D-glucopyranosyl)- (1→4)- *p*-methoxyphenyl 2,3-di-*O*-benzoyl-4-thio-6-O-(triisopropylsilyl)-β-D-glucopyranoside 41**

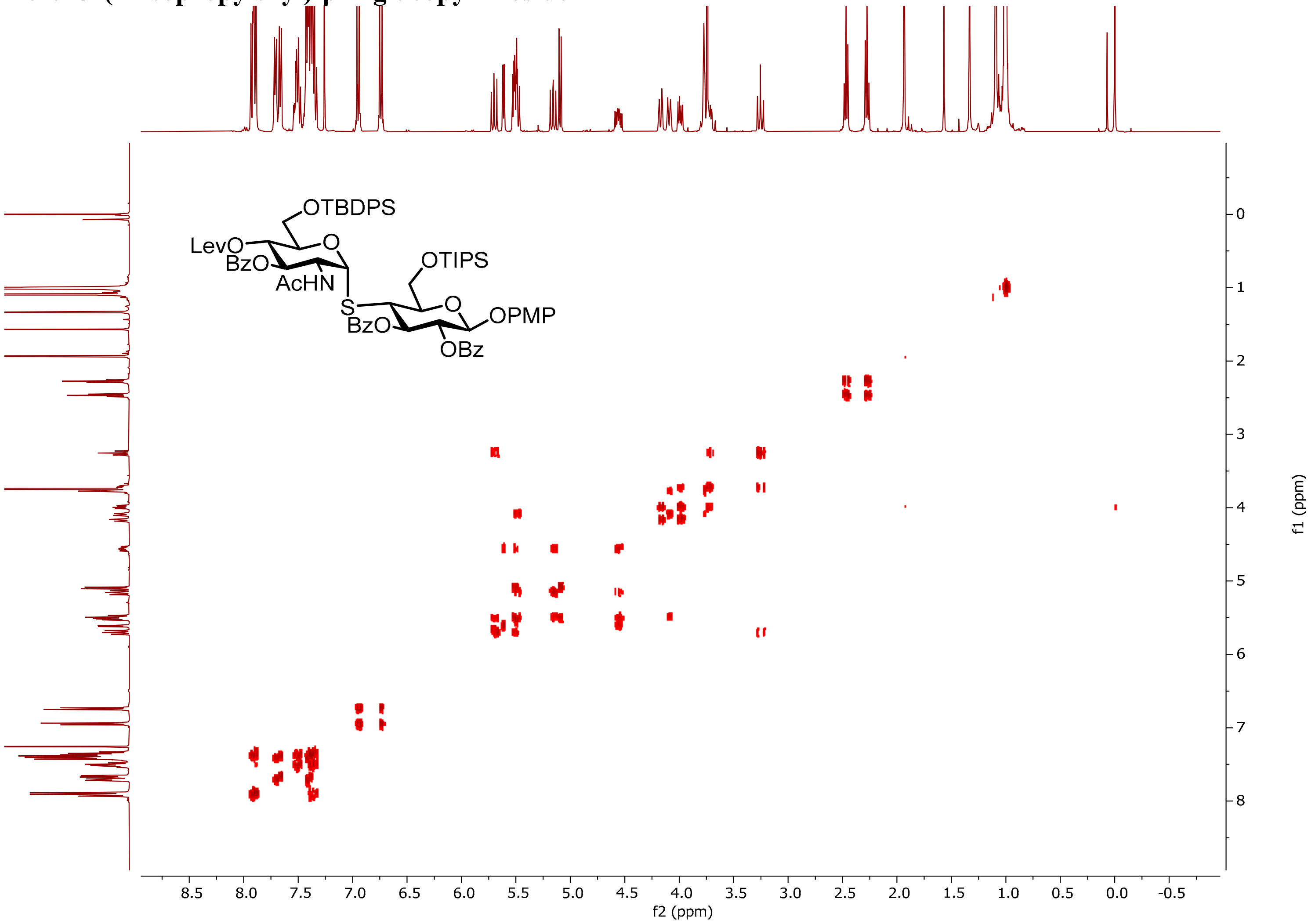

COSY (400 × 400 MHz, Chloroform-*d*)

***S*-(2-Acetamido-2-deoxy-3-*O*-benzoyl-4-*O*-levulinoyl-6-*O*-(*tert*-butyldiphenylsilyl)-α-D-glucopyranosyl)- (1→4)- *p*-methoxyphenyl 2,3-di-*O*-benzoyl-4-thio-6-O-(triisopropylsilyl)-β-D-glucopyranoside 41**

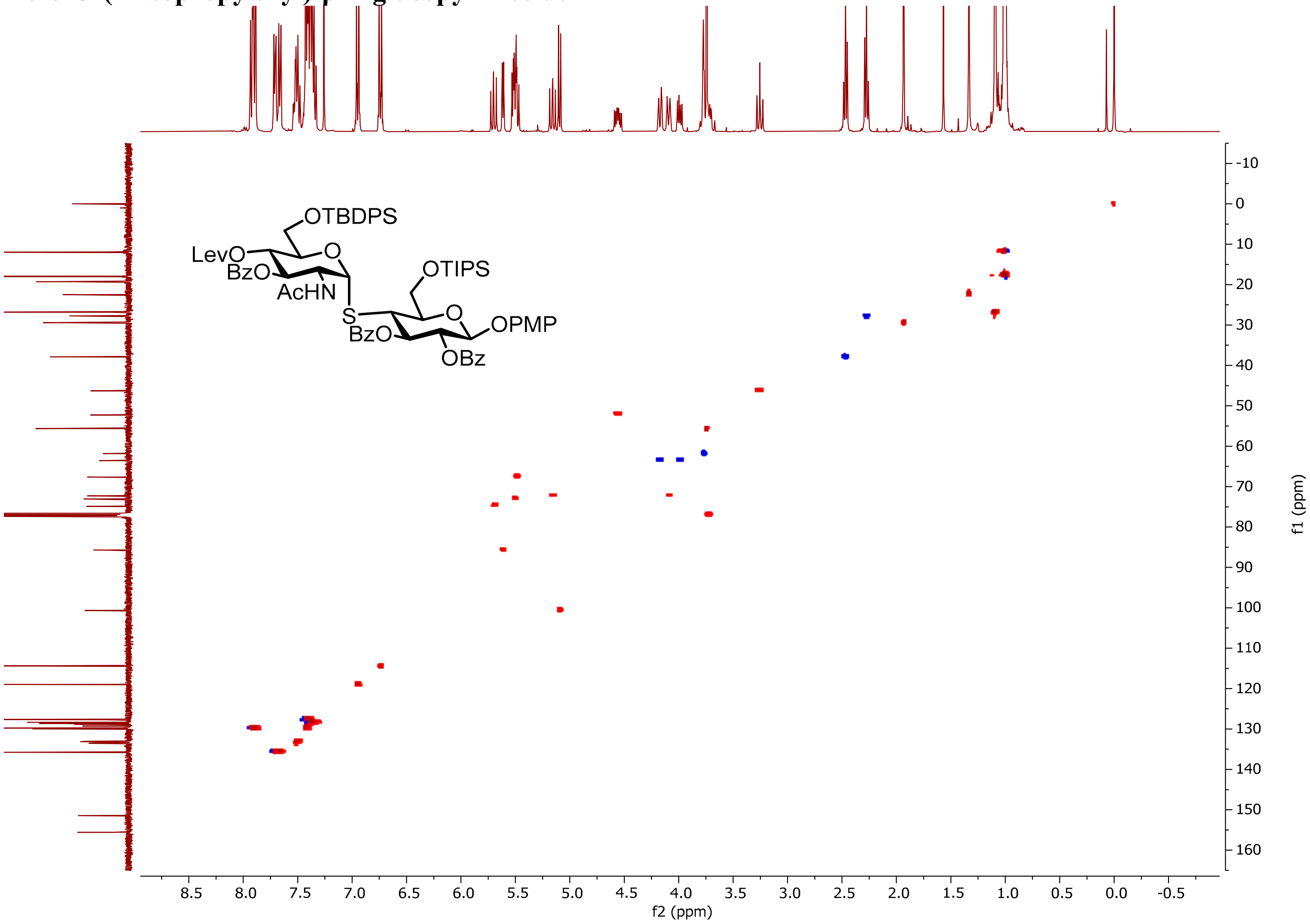

HSQC (400 × 100 MHz, Chloroform-*d*)

***S*-(2-Acetamido-2-deoxy-3-*O*-benzoyl-4-*O*-levulinoyl-6-*O*-(*tert*-butyldiphenylsilyl)-α-D-glucopyranosyl)- (1→4)- *p*-methoxyphenyl 2,3-di-*O*-benzoyl-4-thio-6-O-(triisopropylsilyl)-β-D-glucopyranoside 41**

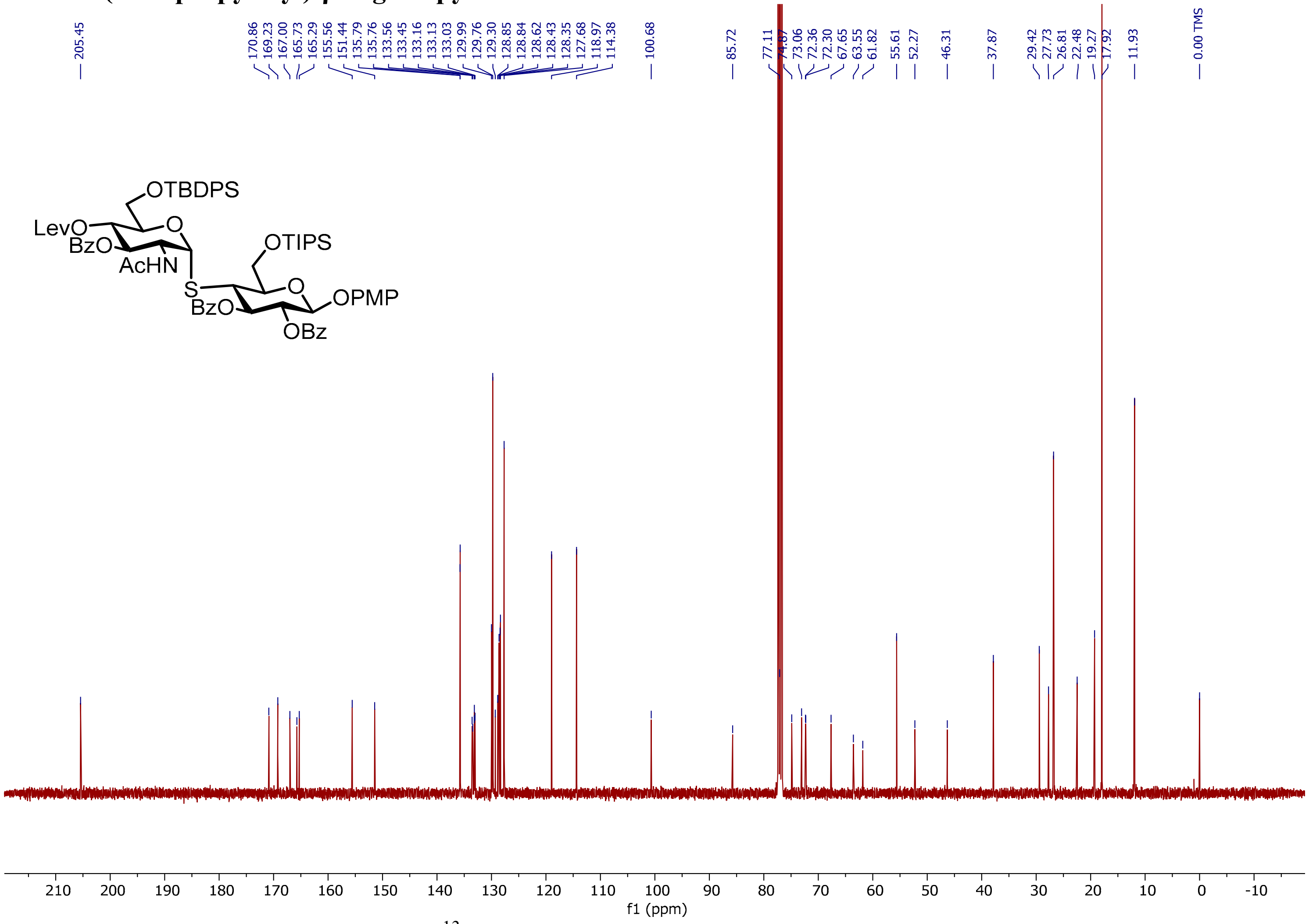

^{13}C NMR (100 MHz, Chloroform-*d*,)

_S_-(2-Azido-3-_O_-benzyl-2-deoxy-4-_O_-levulinoyl-6-_O_-(_tert_-butyldiphenylsilyl)-α-D-glucopyranosyl)- (1→4)- _p_-methoxyphenyl 2,3-di-_O_-benzoyl-4-thio-β-D-glucopyranoside 43

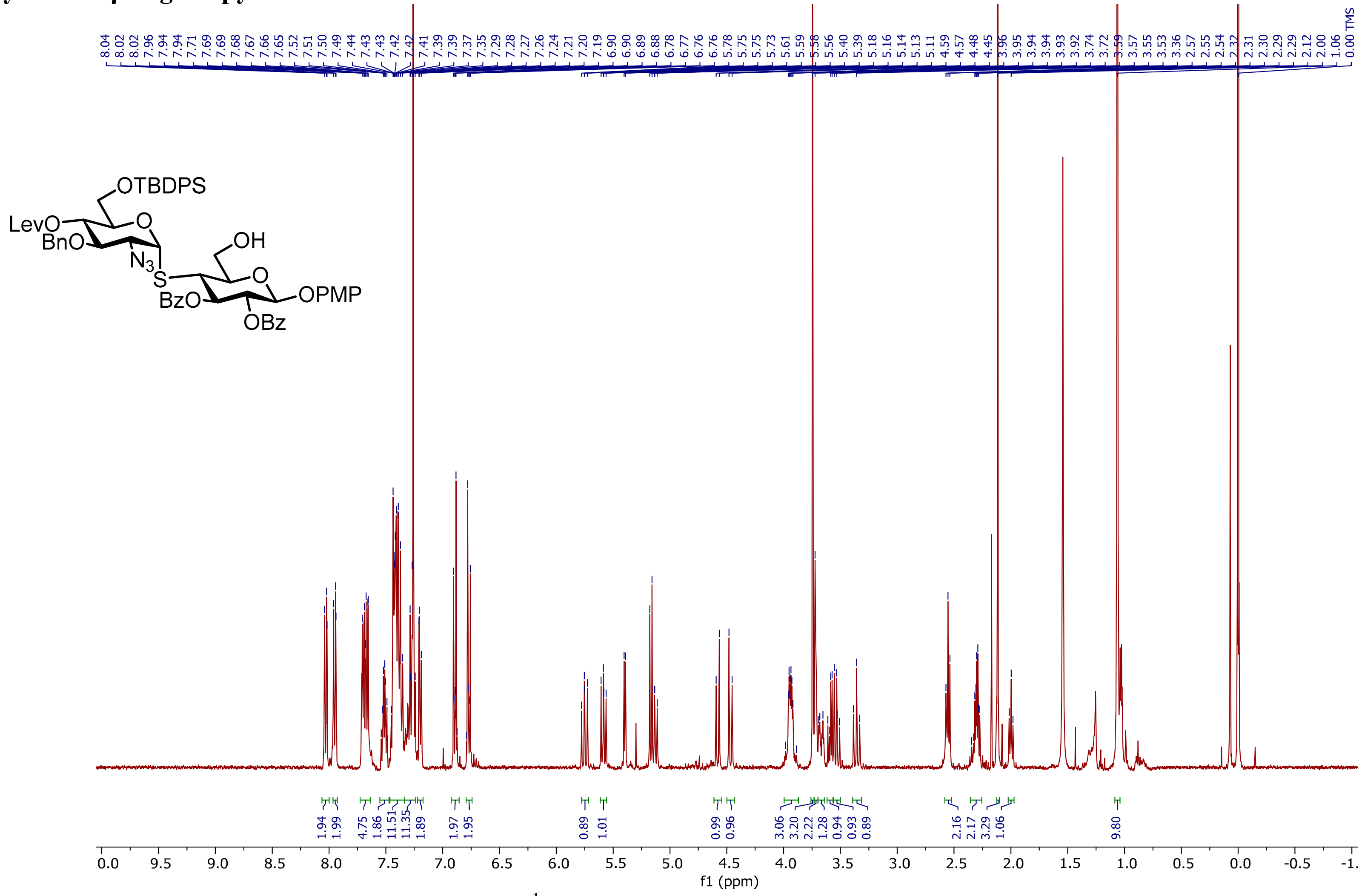

^{1}H NMR (400 MHz, Chloroform-_d_)

S-(2-Azido-3-O-benzyl-2-deoxy-4-O-levulinoyl-6-O-(tert-butyldiphenylsilyl)-α-D-glucopyranosyl)- (1→4)- p-methoxyphenyl 2,3-di-O-benzoyl-4-thio-β-D-glucopyranoside 43

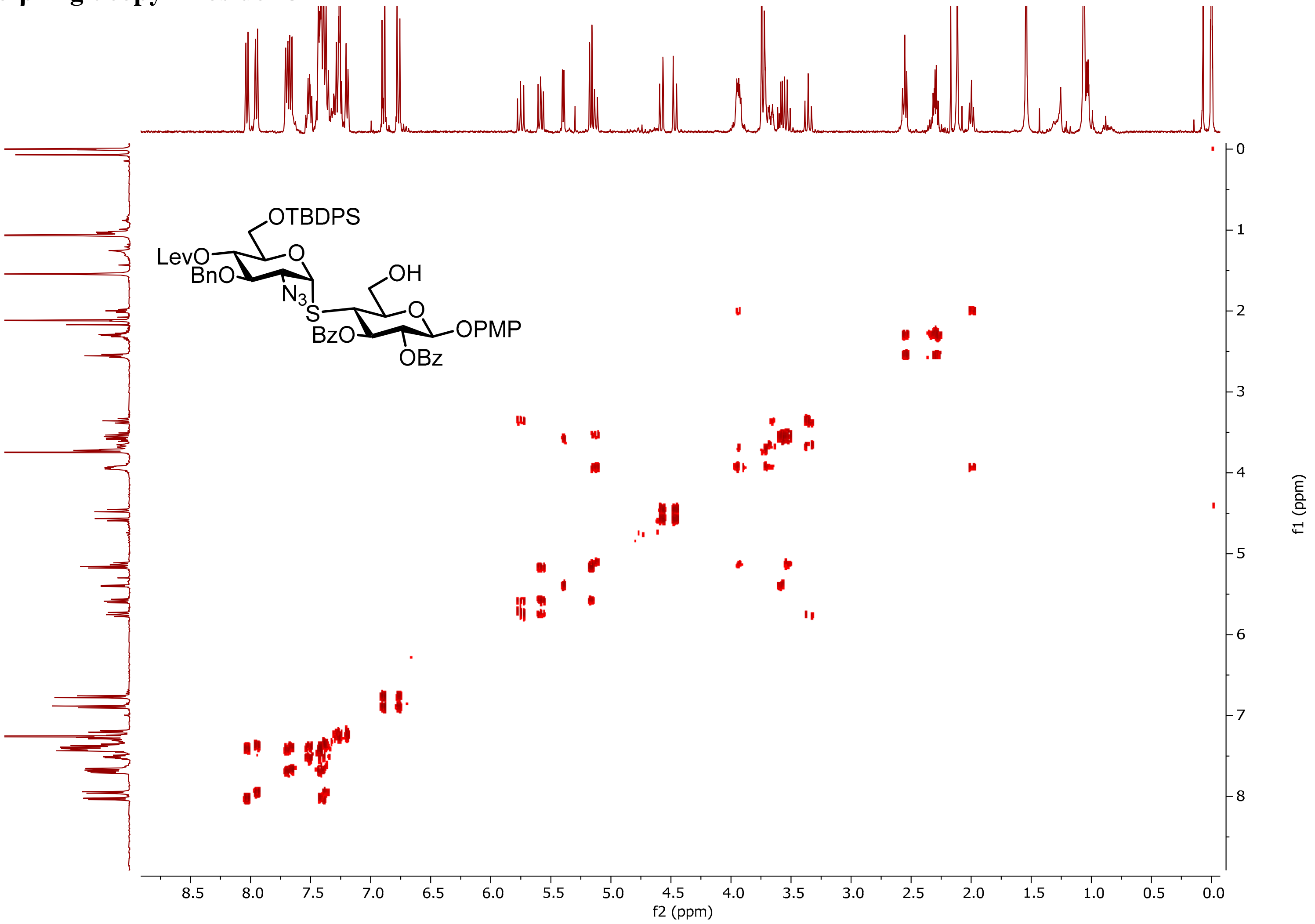

COSY (400 × 400 MHz, Chloroform-d)

S-(2-Azido-3-O-benzyl-2-deoxy-4-O-levulinoyl-6-O-(*tert*-butyldiphenylsilyl)-α-D-glucopyranosyl)- (1→4)- *p*-methoxyphenyl 2,3-di-O-benzoyl-4-thio-β-D-glucopyranoside 43

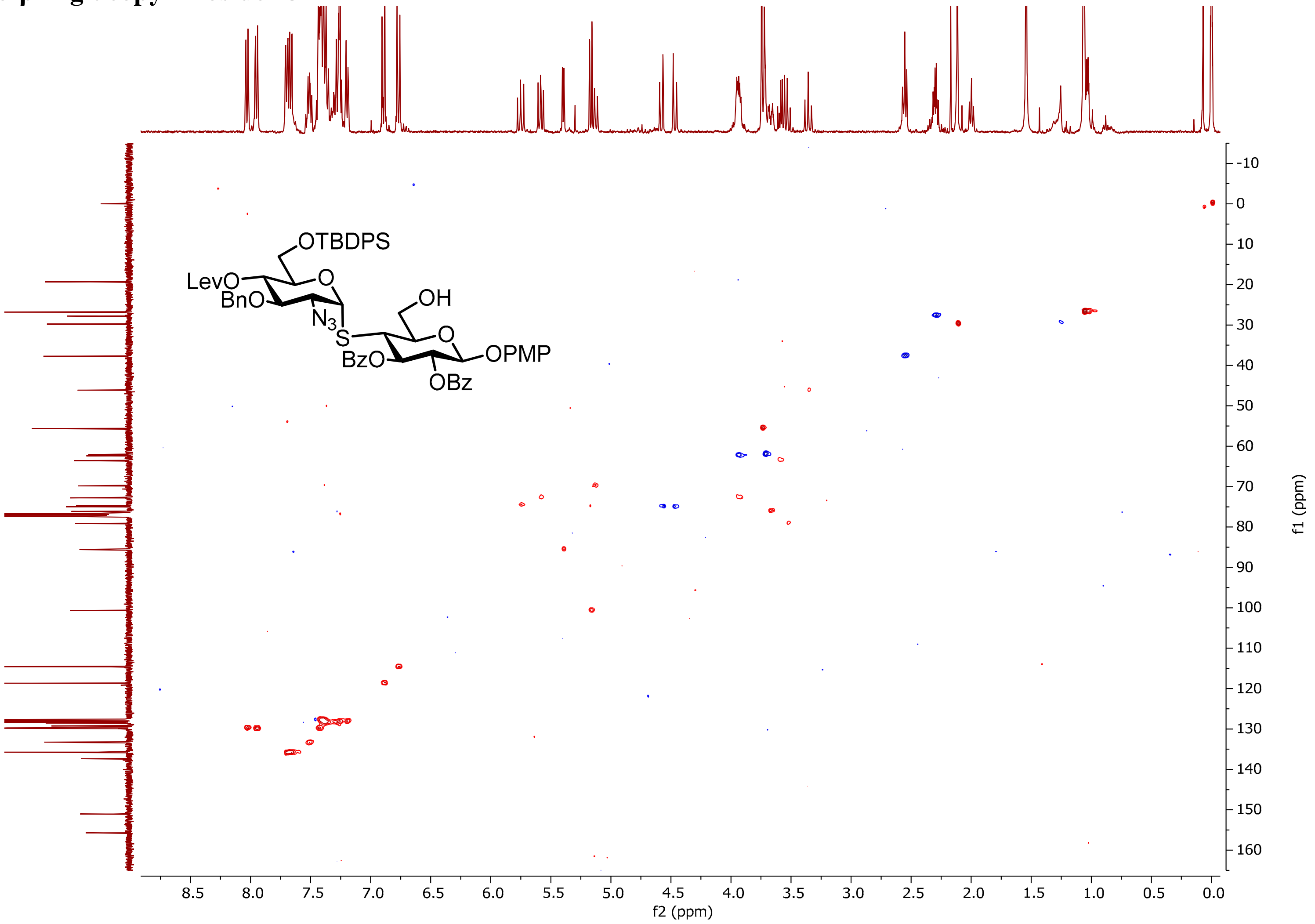

HSQC (400 × 100 MHz, Chloroform-*d*)

S-(2-Azido-3-O-benzyl-2-deoxy-4-O-levulinoyl-6-O-(tert-butyldiphenylsilyl)-α-D-glucopyranosyl)- (1→4)- p-methoxyphenyl 2,3-di-O-benzoyl-4-thio-β-D-glucopyranoside 43

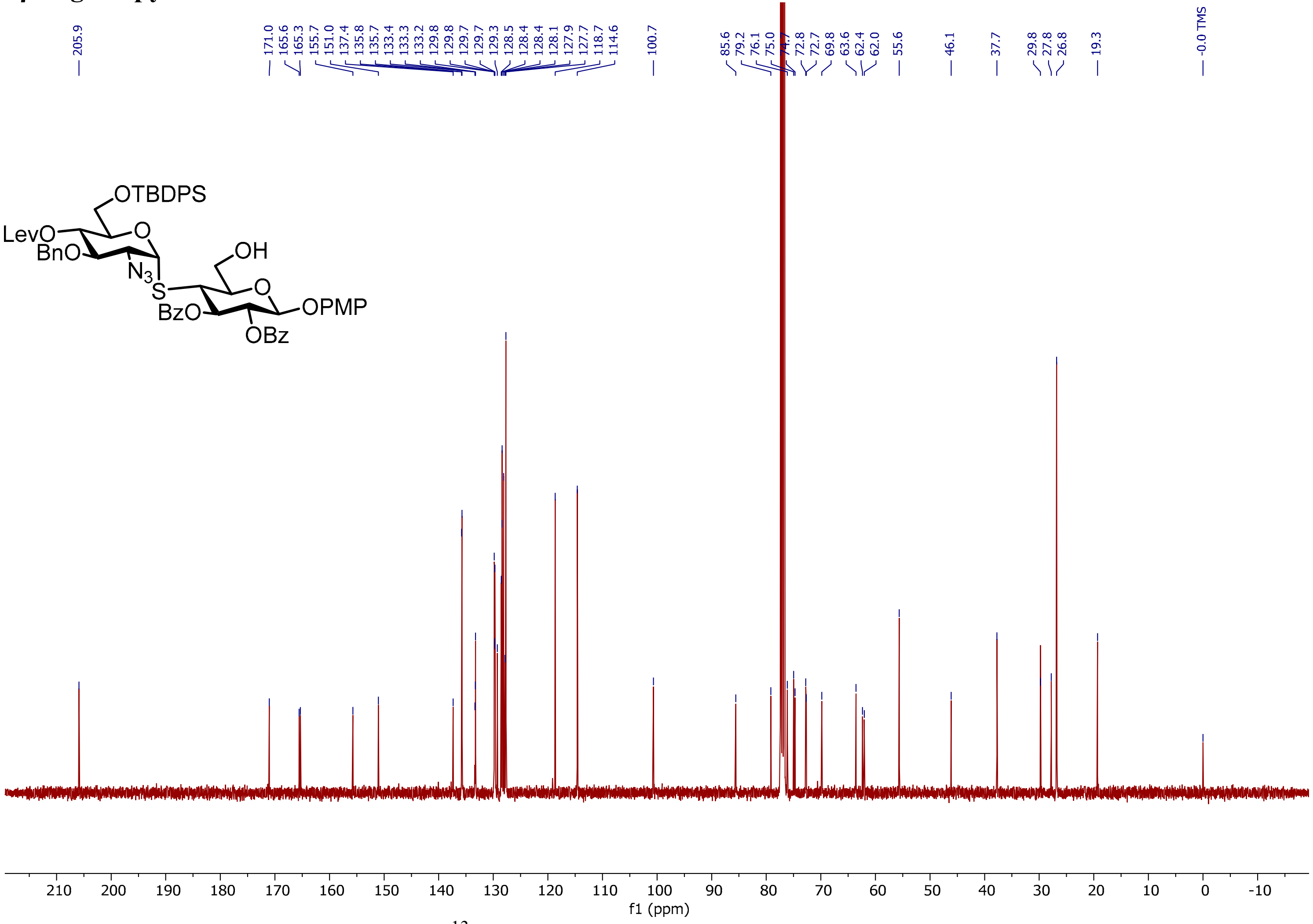

^{13}C NMR (100 MHz, Chloroform-*d*)

***S*-(2-Acetamido-3-*O*-benzoyl-2-deoxy-4-*O*-levulinoyl-6-*O*-(*tert*-butyldiphenylsilyl)-α-D-glucopyranosyl)- (1→4)- *p*-methoxyphenyl 2,3-di-*O*-benzoyl-4-thio-β-D-glucopyranoside 42**

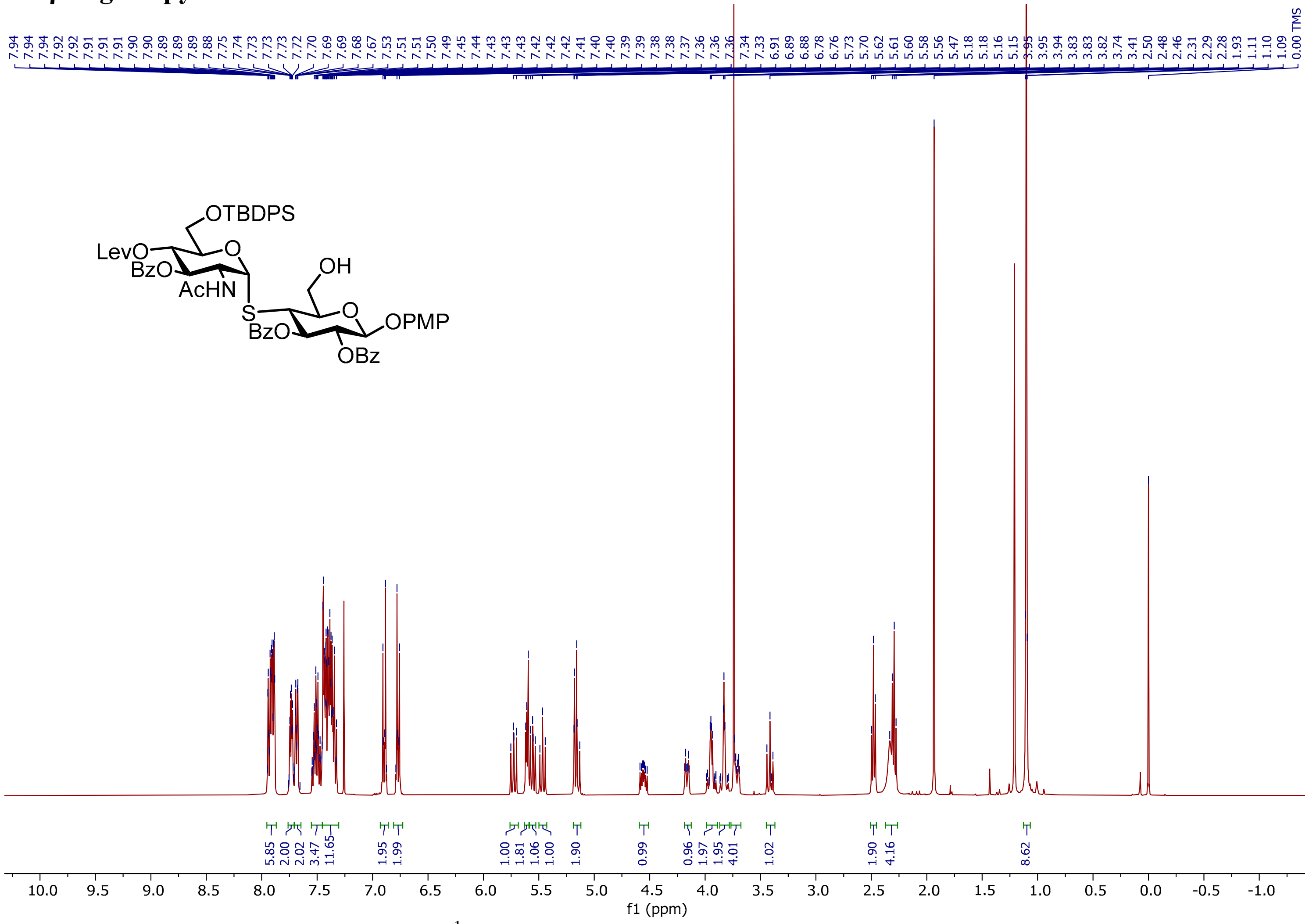

^{1}H NMR (400 MHz, Chloroform-*d*)

***S*-(2-Acetamido-3-*O*-benzoyl-2-deoxy-4-*O*-levulinoyl-6-*O*-(*tert*-butyldiphenylsilyl)-α-D-glucopyranosyl)-(1→4)-*p*-methoxyphenyl 2,3-di-*O*-benzoyl-4-thio-β-D-glucopyranoside 42**

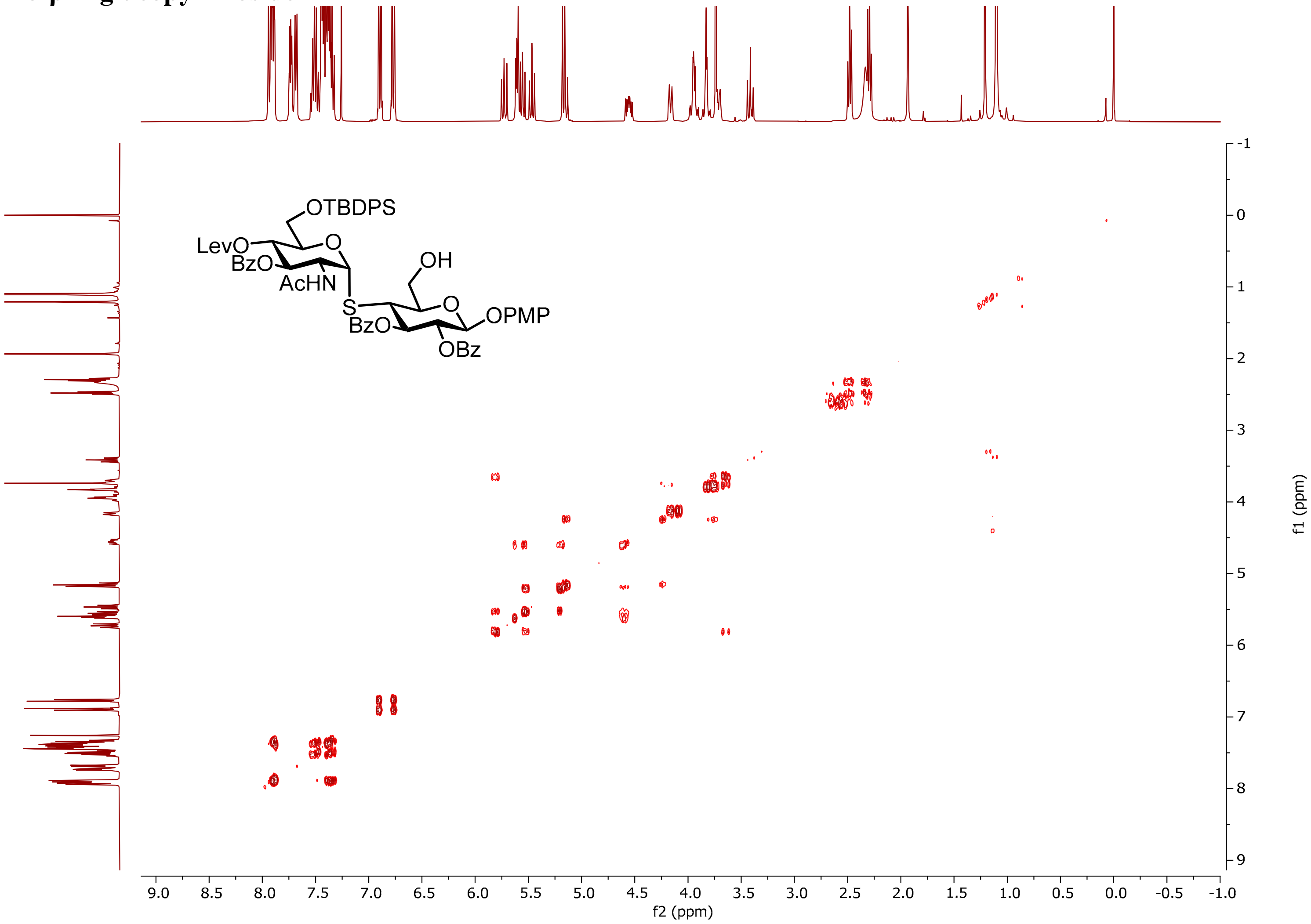

COSY (400 × 400 MHz, Chloroform-*d*)

***S*-(2-Acetamido-3-*O*-benzoyl-2-deoxy-4-*O*-levulinoyl-6-*O*-(*tert*-butyldiphenylsilyl)-α-D-glucopyranosyl)-(1→4)-*p*-methoxyphenyl 2,3-di-*O*-benzoyl-4-thio-β-D-glucopyranoside 42**

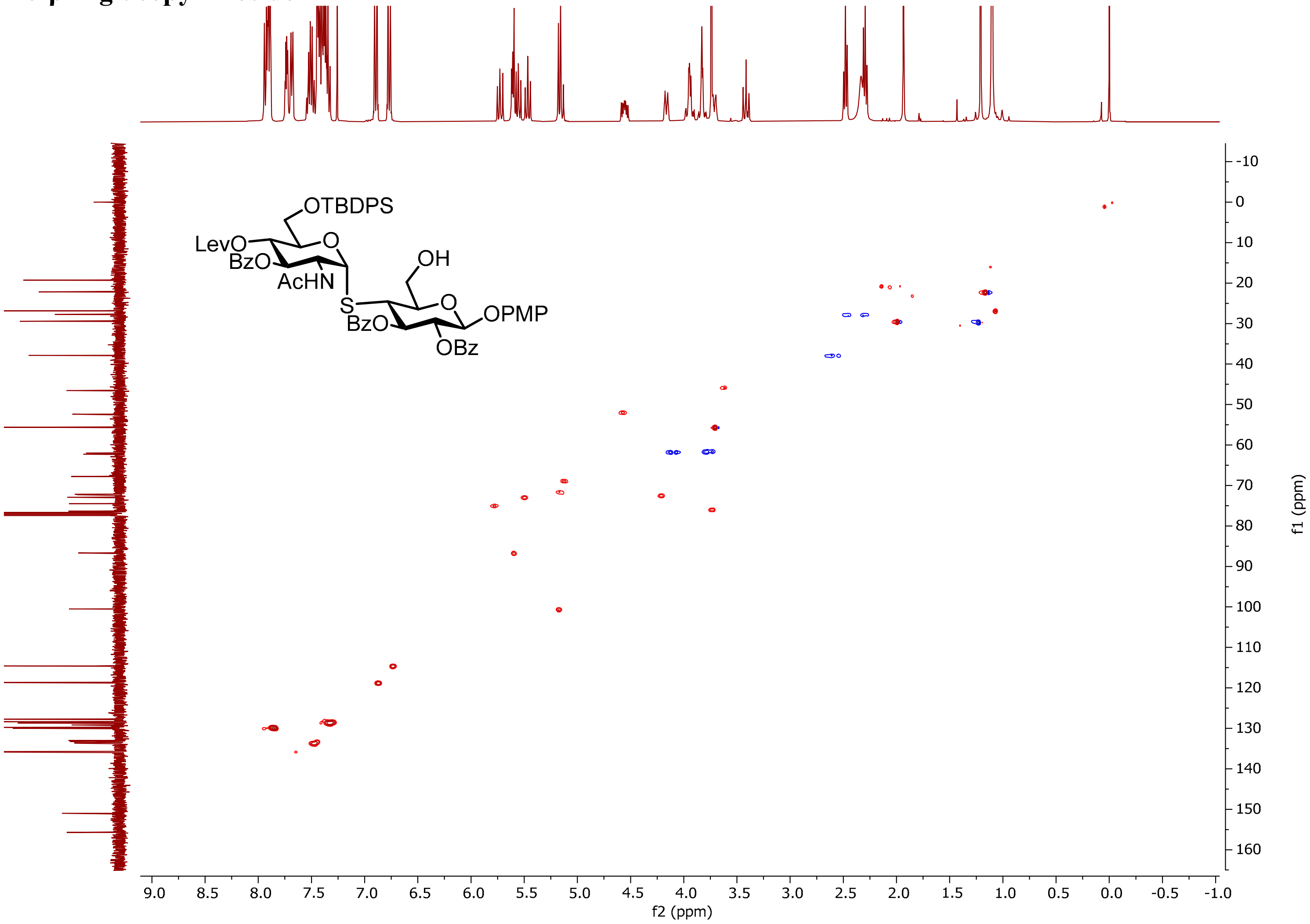

HSQC (400 × 100 MHz, Chloroform-*d*)

_S_-(2-Acetamido-3-_O_-benzoyl-2-deoxy-4-_O_-levulinoyl-6-_O_-(_tert_-butyldiphenylsilyl)-α-D-glucopyranosyl)-(1→4)-_p_-methoxyphenyl 2,3-di-_O_-benzoyl-4-thio-β-D-glucopyranoside 42

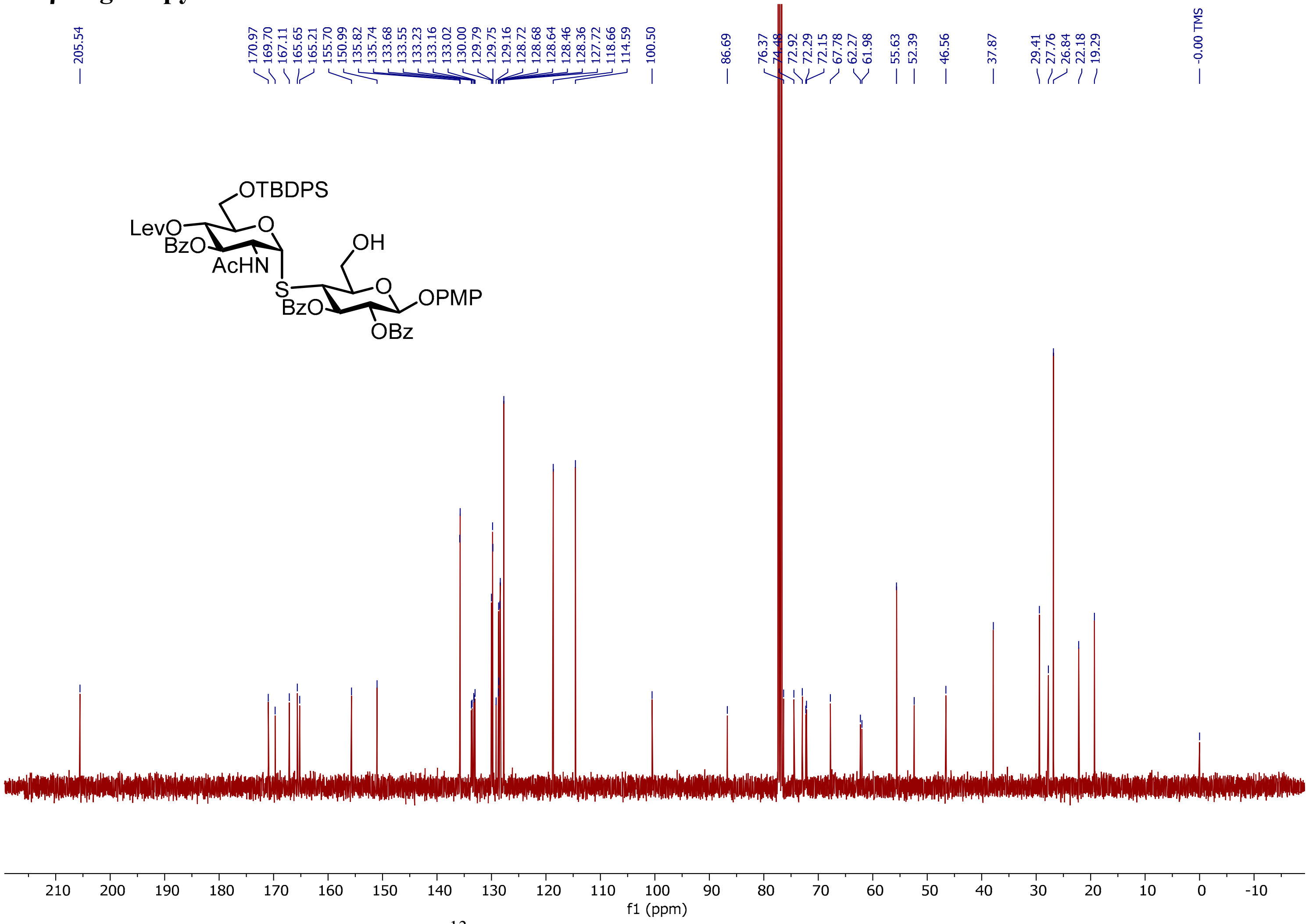

^{13}C NMR (100 MHz, Chloroform-d)

Methyl *S*-(2-azido-3-*O*-benzyl-2-deoxy-4-*O*-levulinoyl-6-*O*-(*tert*-butyldiphenylsilyl)-α-D-glucopyranosyl)-(1→4)-(*p*-methoxyphenyl 2,3-*O*-dibenzoyl-4-thio-β-D-glucopyranosid)uronate 45

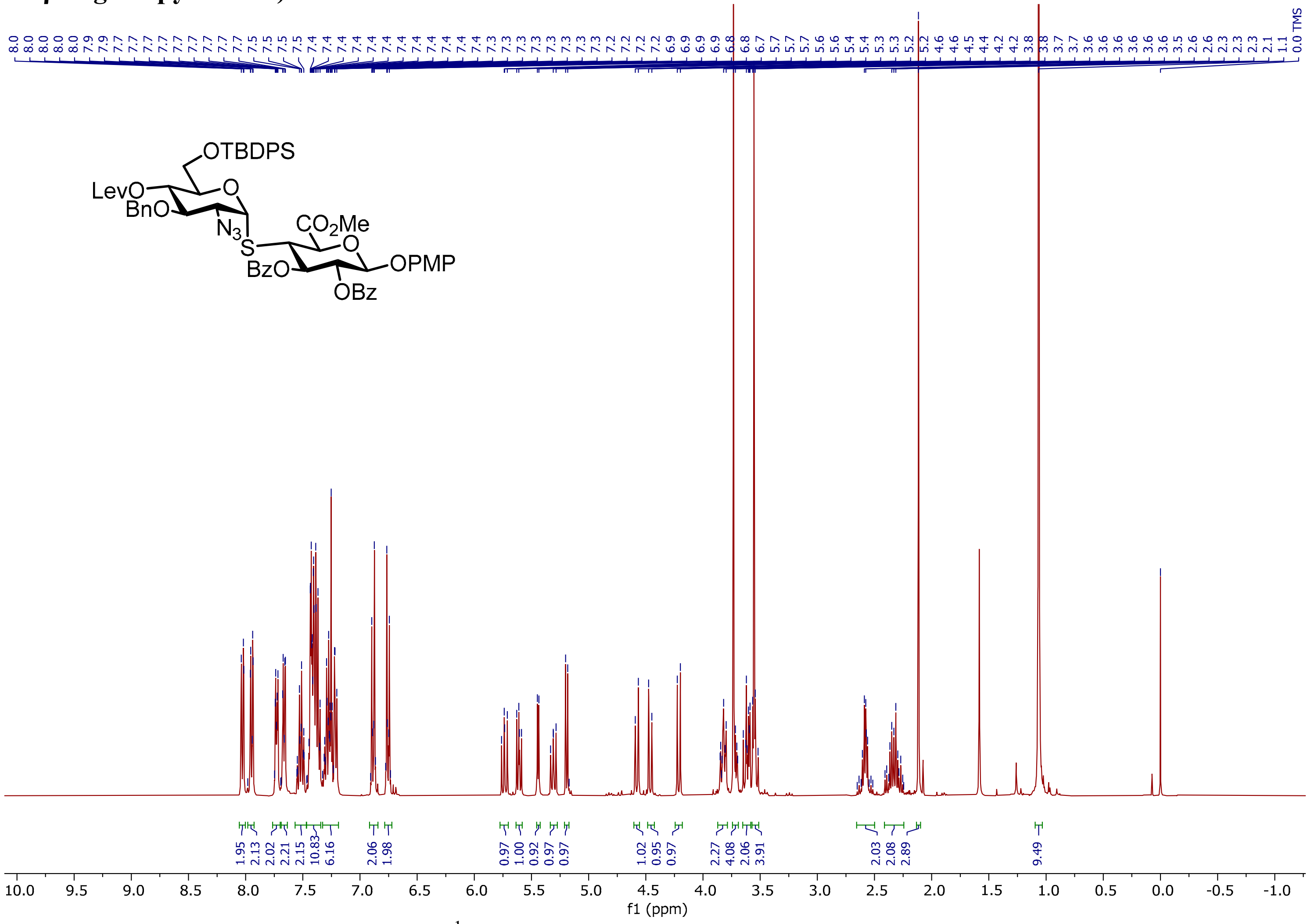

^{1}H NMR (400 MHz, Chloroform-*d*)

Methyl *S*-(2-azido-3-*O*-benzyl-2-deoxy-4-*O*-levulinoyl-6-*O*-(*tert*-butyldiphenylsilyl)-α-D-glucopyranosyl)-(1→4)- (*p*-methoxyphenyl 2,3-*O*-dibenzoyl-4-thio-β-D-glucopyranosid)uronate 45

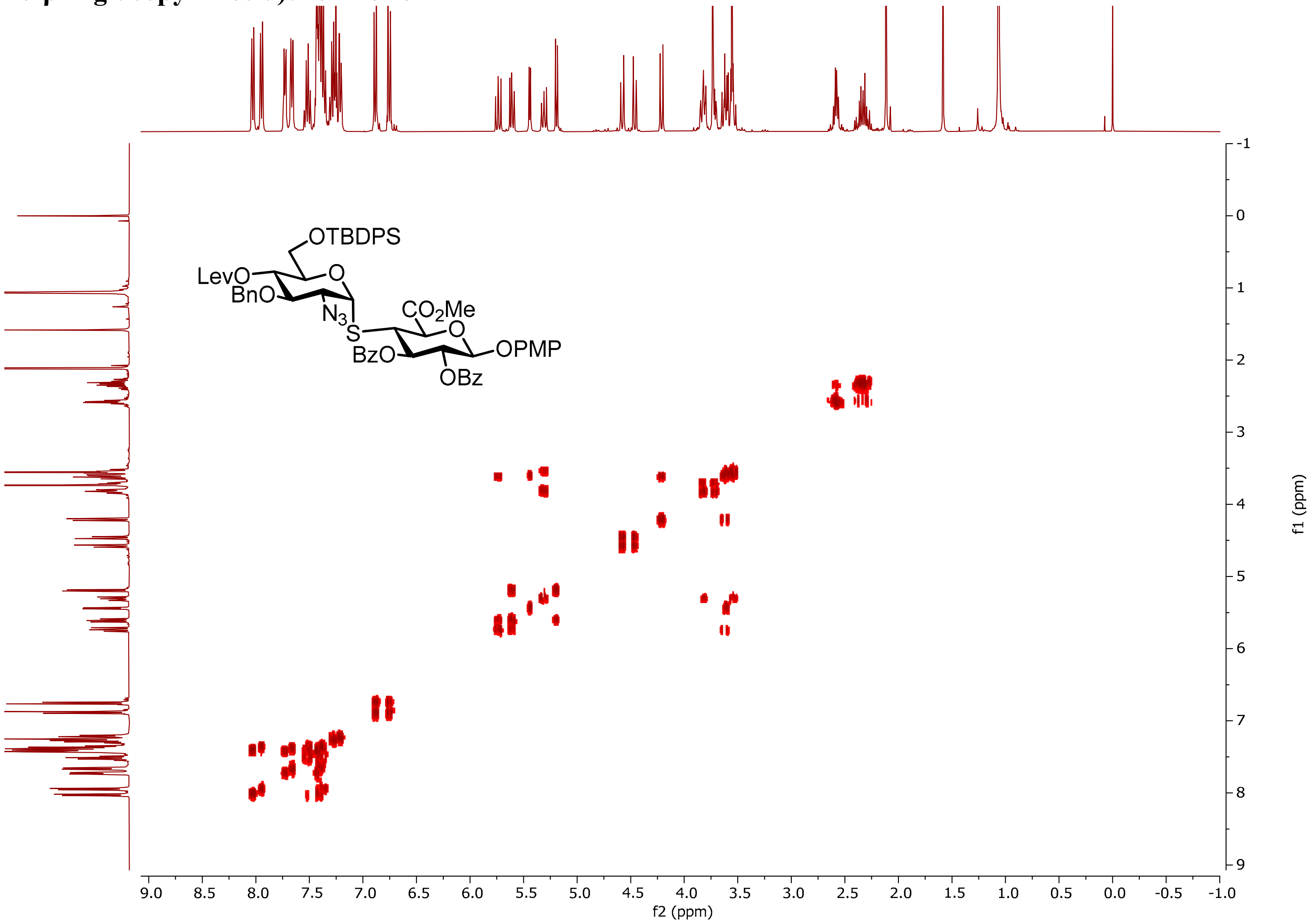

COSY (400 × 400 MHz, Chloroform-*d*)

Methyl *S*-(2-azido-3-*O*-benzyl-2-deoxy-4-*O*-levulinoyl-6-*O*-(*tert*-butyldiphenylsilyl)-α-D-glucopyranosyl)-(1→4)- (*p*-methoxyphenyl 2,3-*O*-dibenzoyl-4-thio-β-D-glucopyranosid)uronate 45

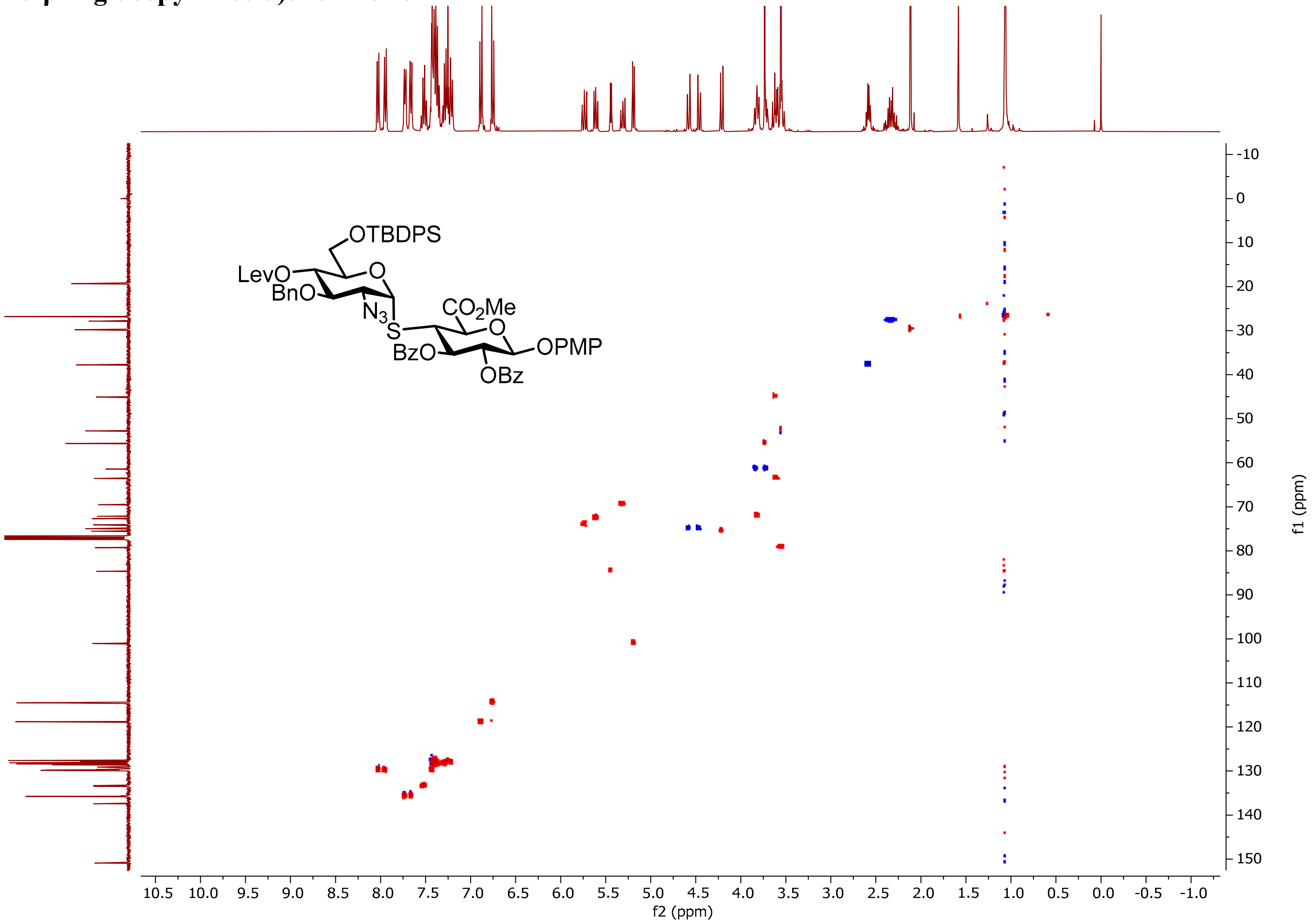

HSQC (400 × 100 MHz, Chloroform-*d*)

Methyl *S*-(2-azido-3-*O*-benzyl-2-deoxy-4-*O*-levulinoyl-6-*O*-(*tert*-butyldiphenylsilyl)-α-D-glucopyranosyl)-(1→4)- (*p*-methoxyphenyl 2,3-*O*-dibenzoyl-4-thio-β-D-glucopyranosid)uronate 45

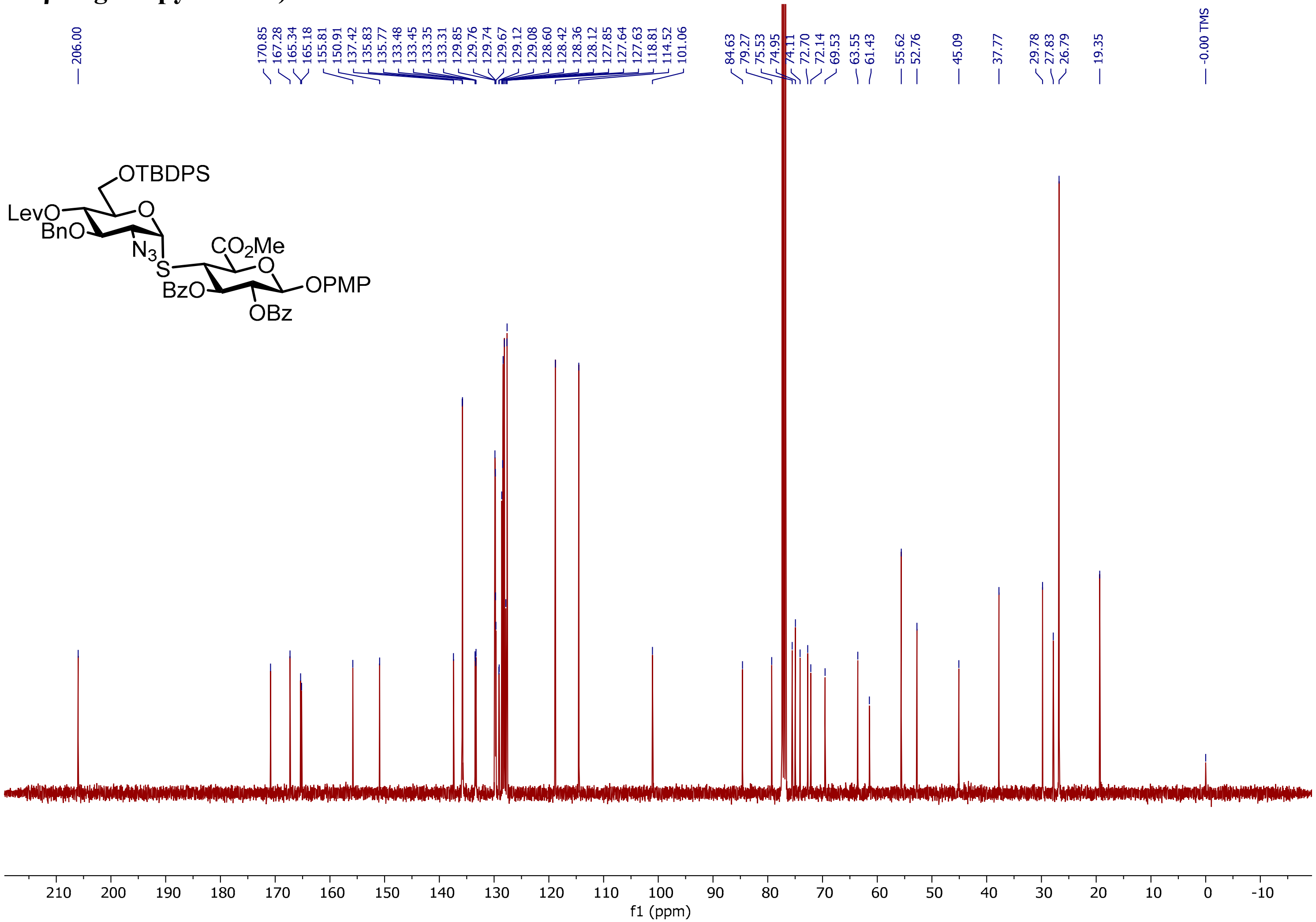

^{13}C NMR (100 MHz, Chloroform-*d*)

Methyl (*S*-(2-acetamido-3-*O*-benzoyl-2-deoxy-4-*O*-levulinoyl-6-*O*-(*tert*-butyldiphenylsilyl)-α-D-glucopyranosyl)- (1→4)-(*p*-methoxyphenyl 2,3-di-*O*-benzoyl-4-thio-β-D-glucopyranosid)uronate 44

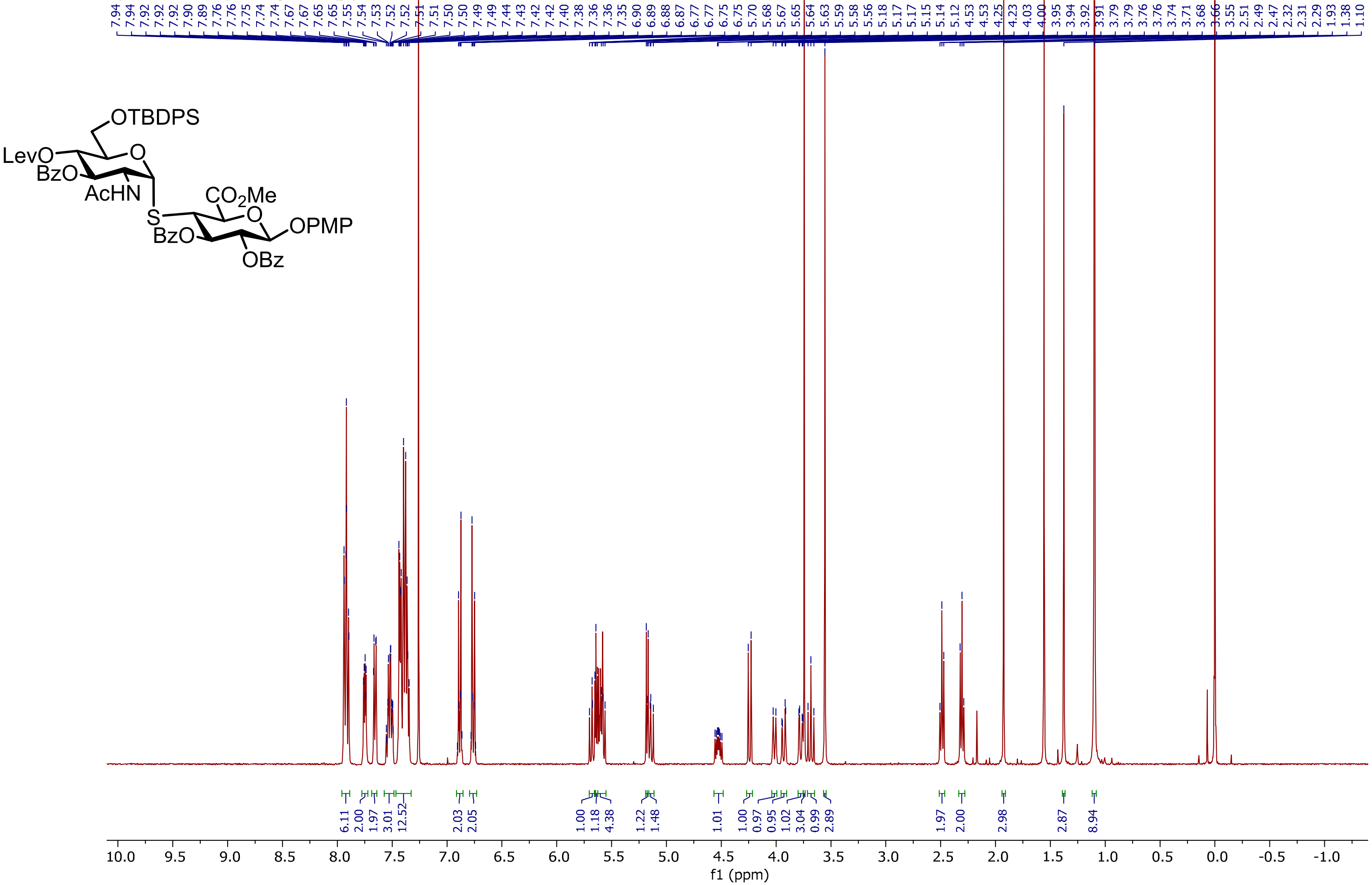

^{1}H NMR (400 MHz, Chloroform-*d*)

Methyl (*S*-(2-acetamido-3-*O*-benzoyl-2-deoxy-4-*O*-levulinoyl-6-*O*-(*tert*-butyldiphenylsilyl)-α-D-glucopyranosyl)- (1→4)-(*p*-methoxyphenyl 2,3-di-*O*-benzoyl-4-thio-β-D-glucopyranosid)uronate 44

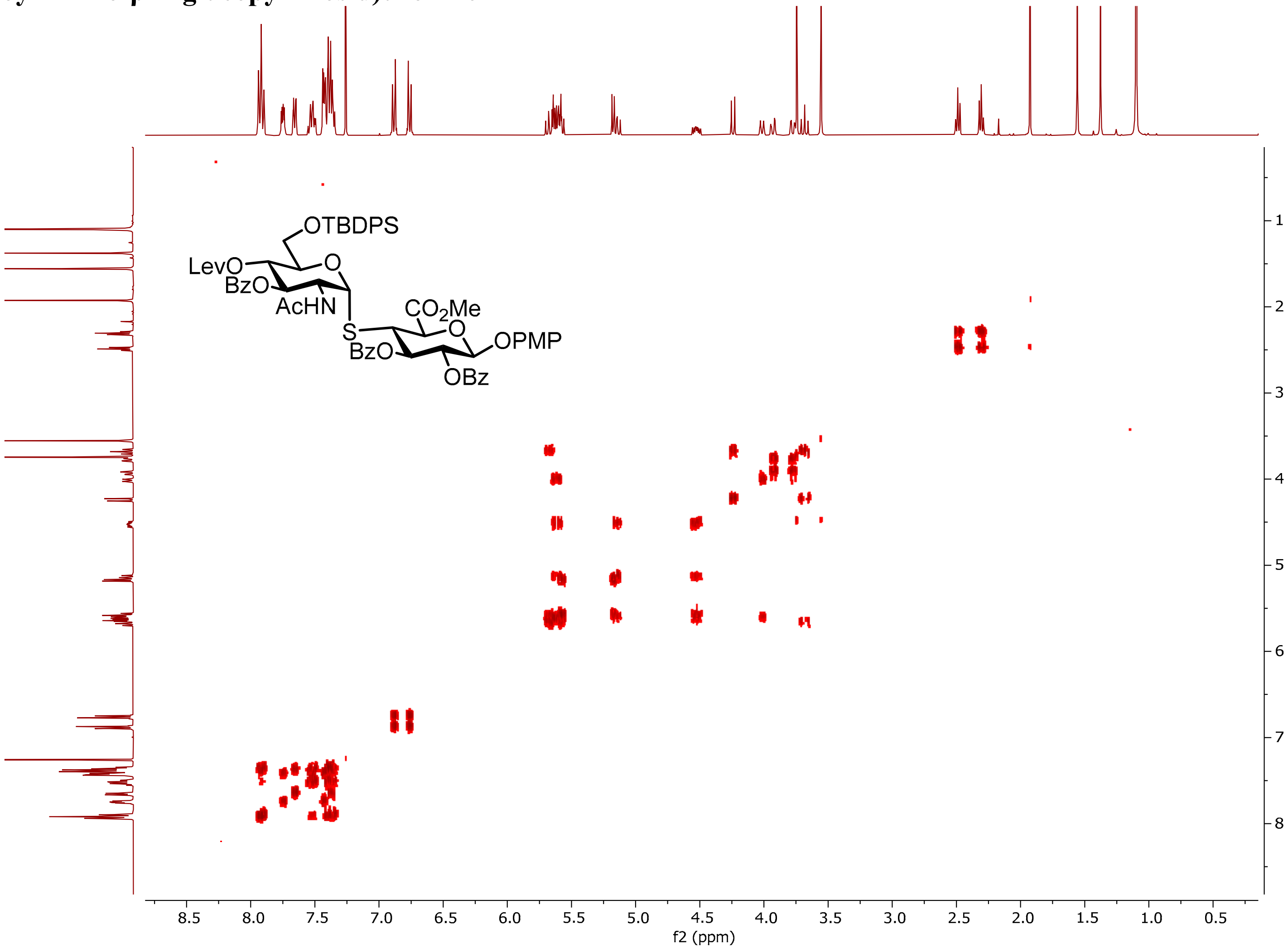

COSY (400 × 400 MHz, Chloroform-*d*)

Methyl (*S*-(2-acetamido-3-*O*-benzoyl-2-deoxy-4-*O*-levulinoyl-6-*O*-(*tert*-butyldiphenylsilyl)-α-D-glucopyranosyl)- (1→4)-(*p*-methoxyphenyl 2,3-di-*O*-benzoyl-4-thio-β-D-glucopyranosid)uronate 44

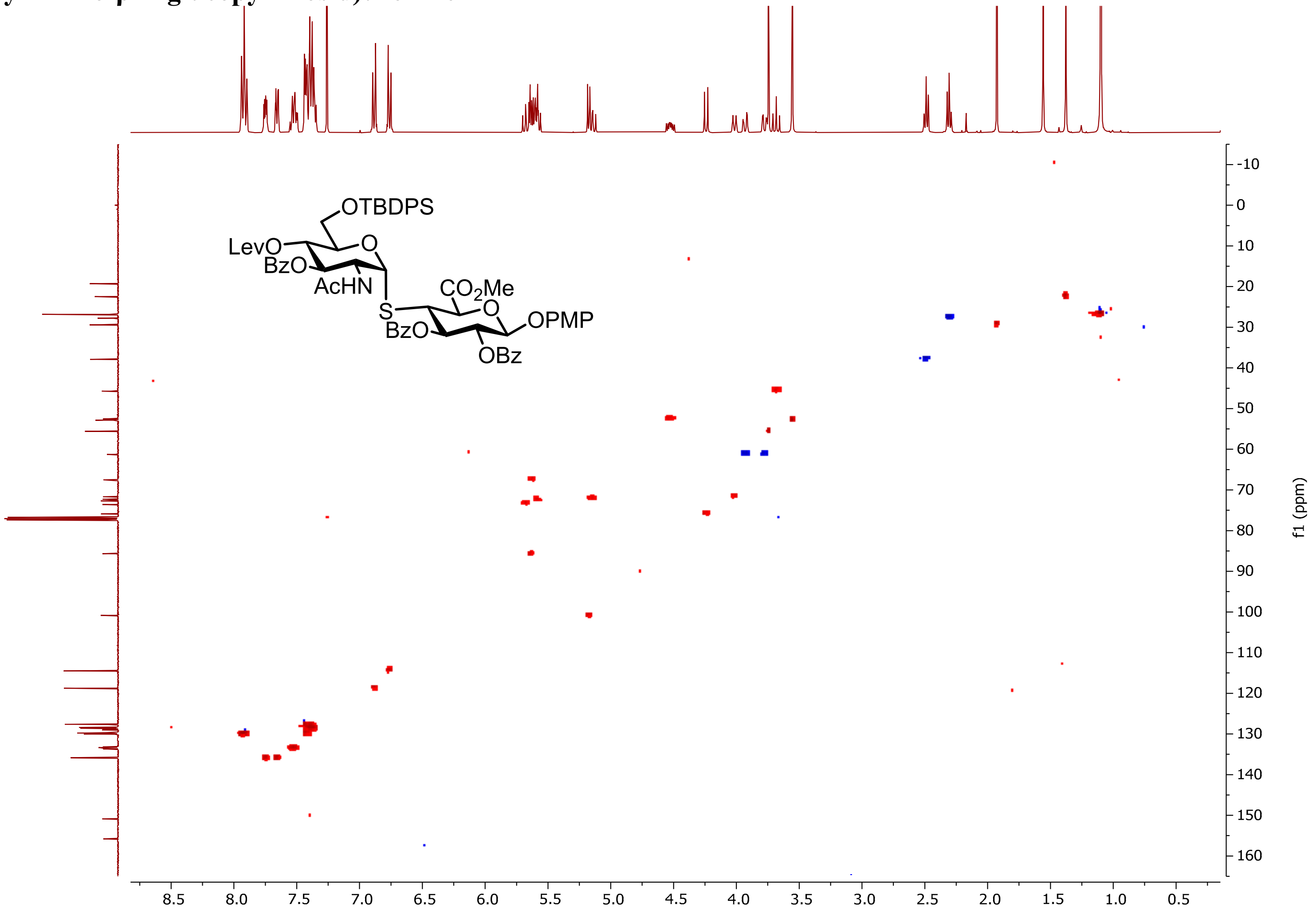

HSQC (400 × 100 MHz, Chloroform-*d*)

Methyl (*S*-(2-acetamido-3-*O*-benzoyl-2-deoxy-4-*O*-levulinoyl-6-*O*-(*tert*-butyldiphenylsilyl)-α-D-glucopyranosyl)- (1→4)-(*p*-methoxyphenyl 2,3-di-*O*-benzoyl-4-thio-β-D-glucopyranosid)uronate 44

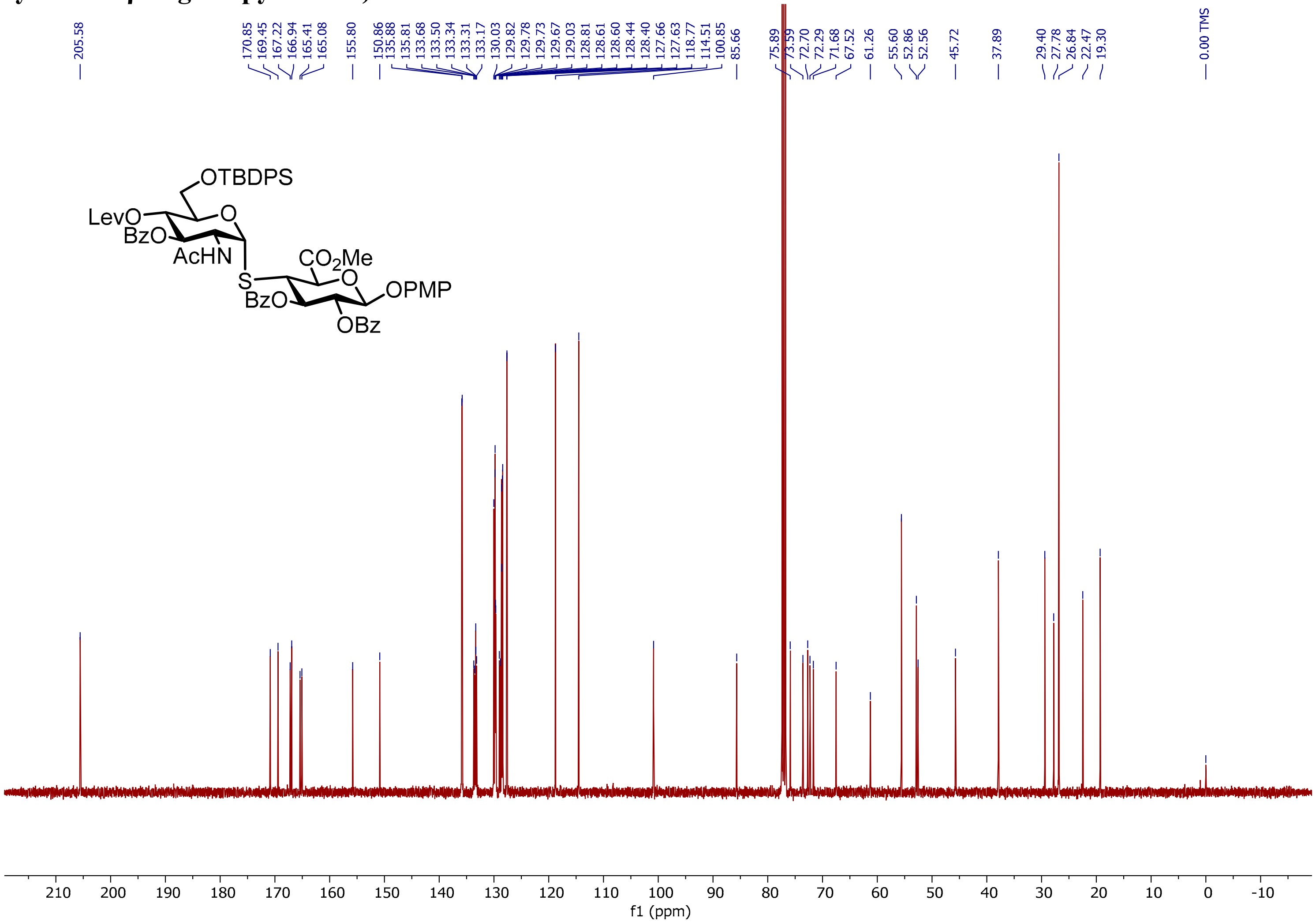

^{13}C NMR (100 MHz, Chloroform-*d*)

Side Products Obtained During NaH Mediated S_N2 Coupling

p-Methoxyphenyl 2,3-di-*O*-benzyl-4-deoxy-6-*O*-(triisopropylsilyl)-α-L-*threo*-hex-4-enopyranosid S13 and ***p*-methoxyphenyl 2,3-di-*O*-benzyl-4-deoxy-6-*O*-(triisopropylsilyl)-β-D-*erythro*-hex-4-enopyranosid S14**

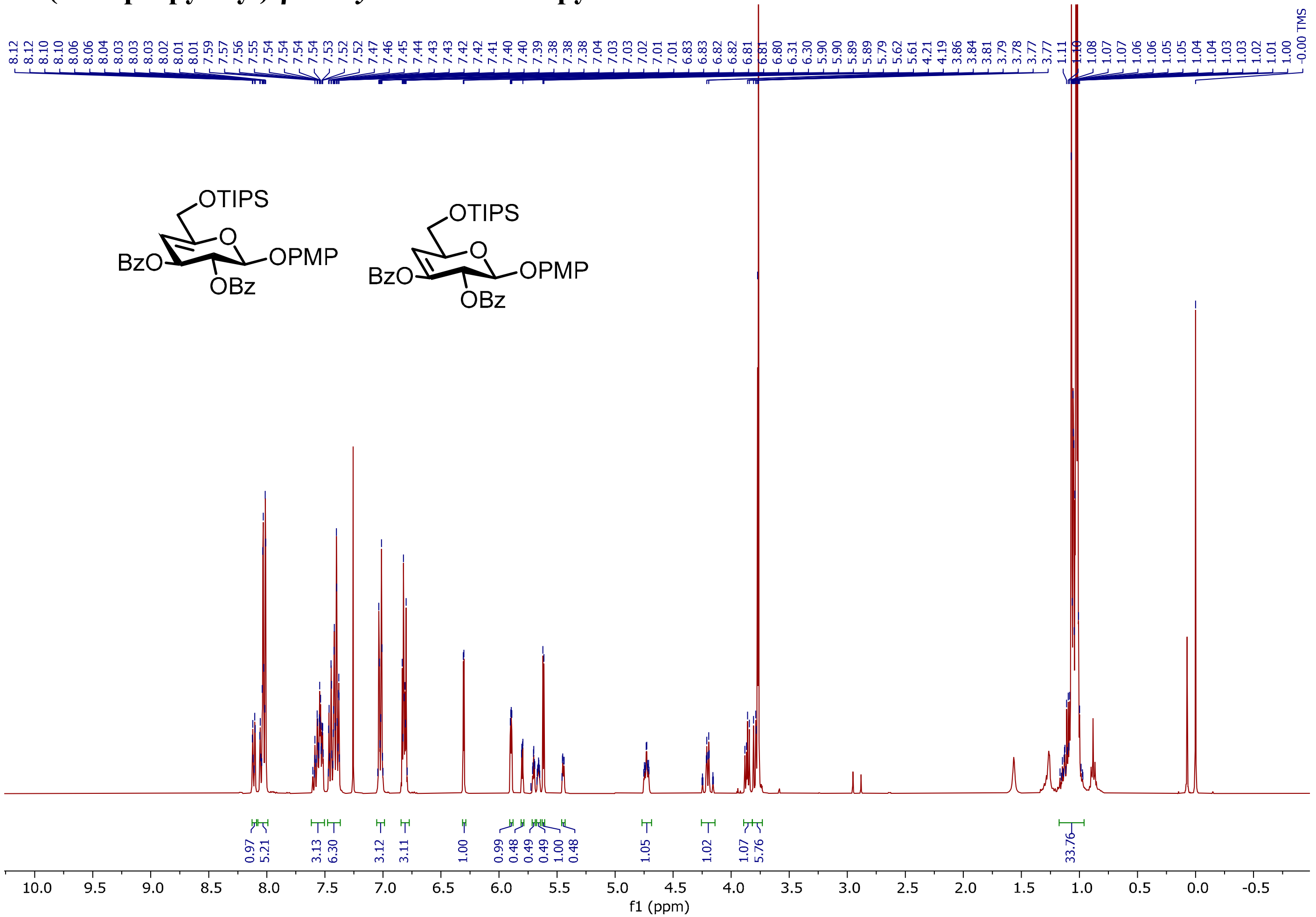

^{1}H NMR (400 MHz, Chloroform-*d*)

***p*-Methoxyphenyl 2,3-di-*O*-benzyl-4-deoxy-6-*O*-(triisopropylsilyl)-α-L-*threo*-hex-4-enopyranosid S13 and *p*-methoxyphenyl 2,3-di-*O*-benzyl-4-deoxy-6-*O*-(triisopropylsilyl)-β-D-*erythro*-hex-4-enopyranosid S14**

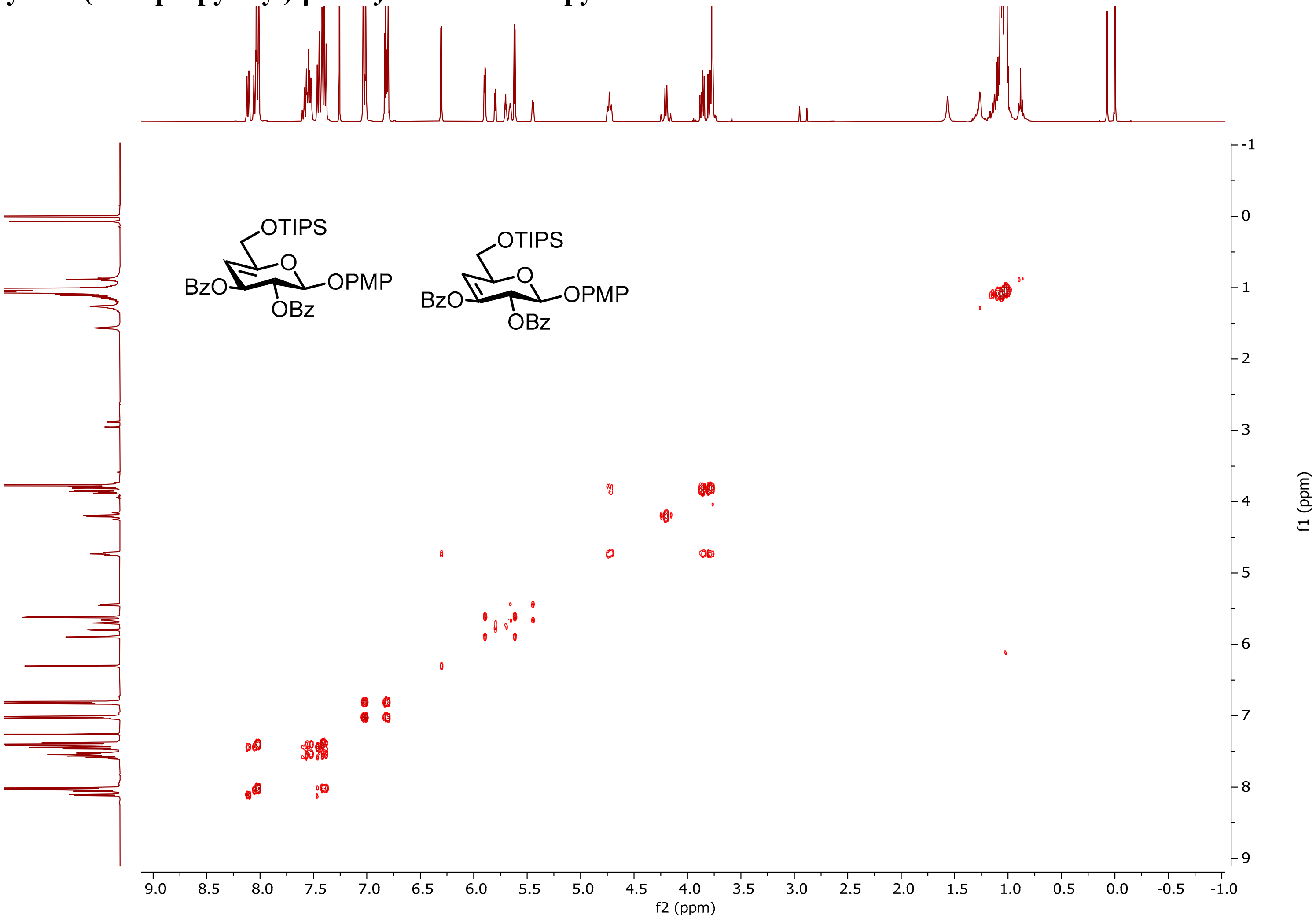

COSY (400 × 400 MHz, Chloroform-*d*)

***p*-Methoxyphenyl 2,3-di-*O*-benzyl-4-deoxy-6-*O*-(triisopropylsilyl)-α-L-*threo*-hex-4-enopyranosid S13 and *p*-methoxyphenyl 2,3-di-*O*-benzyl-4-deoxy-6-*O*-(triisopropylsilyl)-β-D-*erythro*-hex-4-enopyranosid S14**

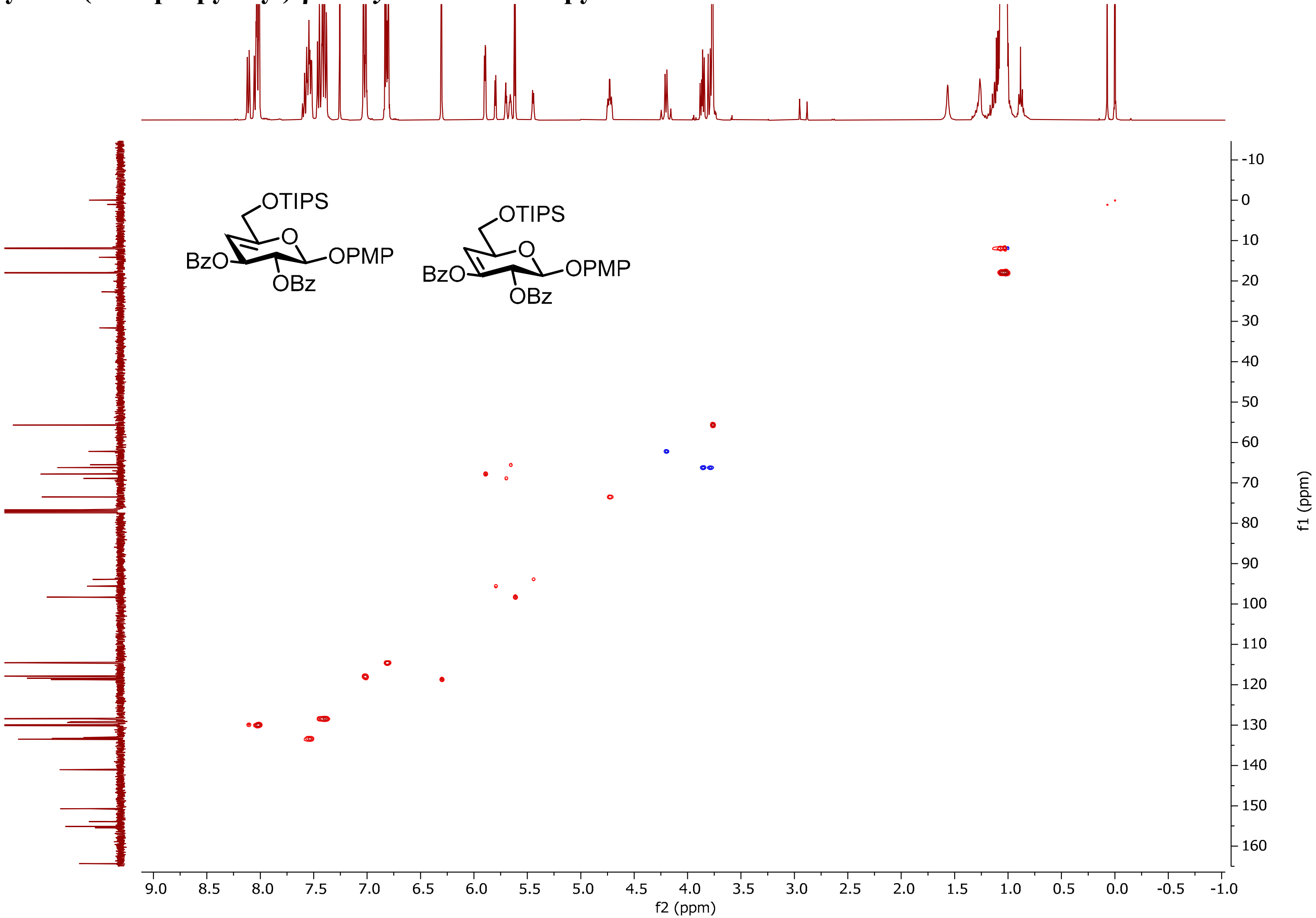

HSQC (400 × 100 MHz, Chloroform-*d*)

***p*-Methoxyphenyl 2,3-di-*O*-benzyl-4-deoxy-6-*O*-(triisopropylsilyl)-α-L-*threo*-hex-4-enopyranosid S13 and *p*-methoxyphenyl 2,3-di-*O*-benzyl-4-deoxy-6-*O*-(triisopropylsilyl)-β-D-*erythro*-hex-4-enopyranosid S14**

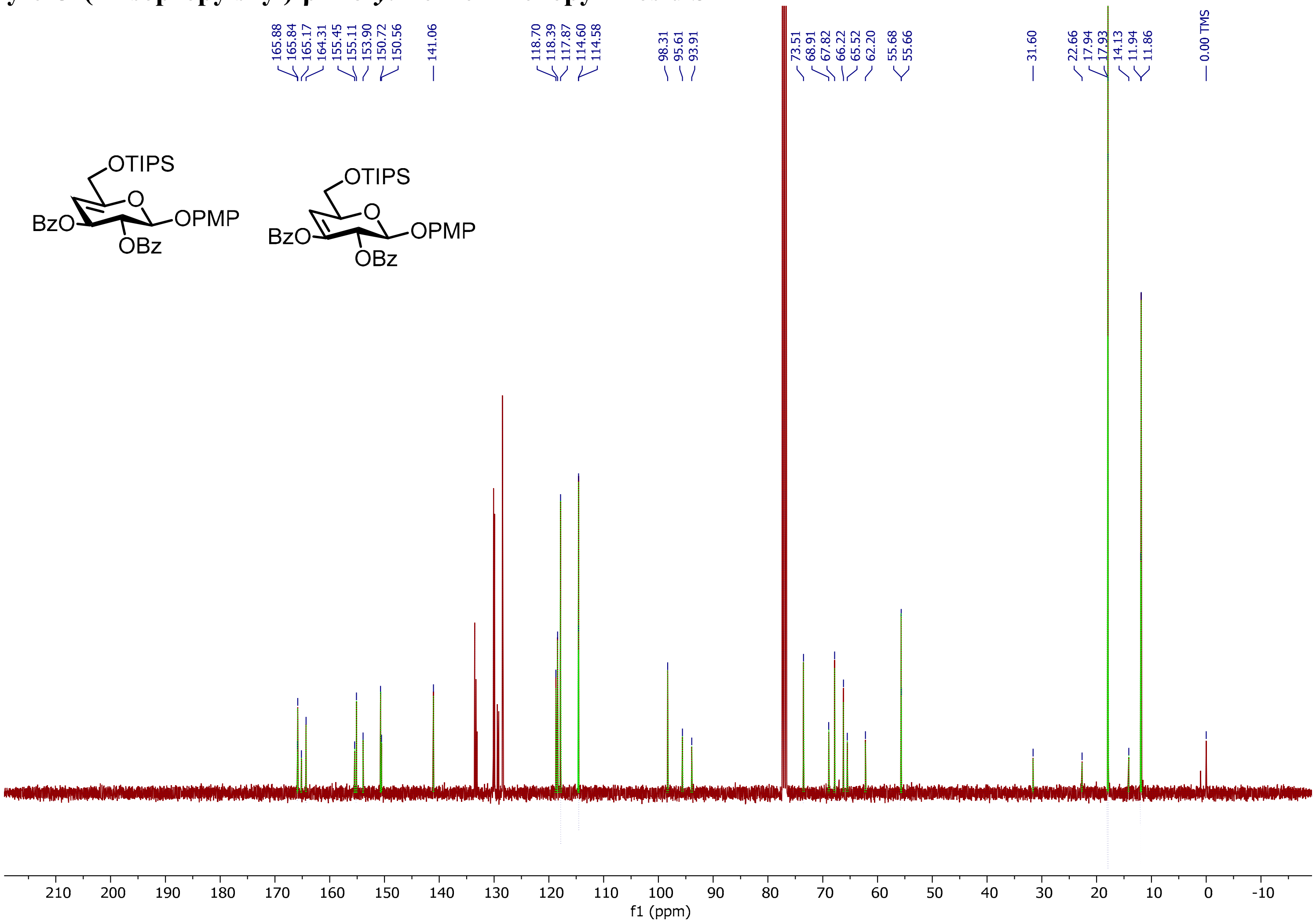

^{13}C NMR (100 MHz, Chloroform-*d*)

***p*-Methoxyphenyl 2,3-di-*O*-benzyl-4-deoxy-6-*O*-(triisopropylsilyl)-α-L-*threo*-hex-4-enopyranosid S13** and ***p*-methoxyphenyl 2,3-di-*O*-benzyl-4-deoxy-6-*O*-(triisopropylsilyl)-β-D-*erythro*-hex-4-enopyranosid S14**

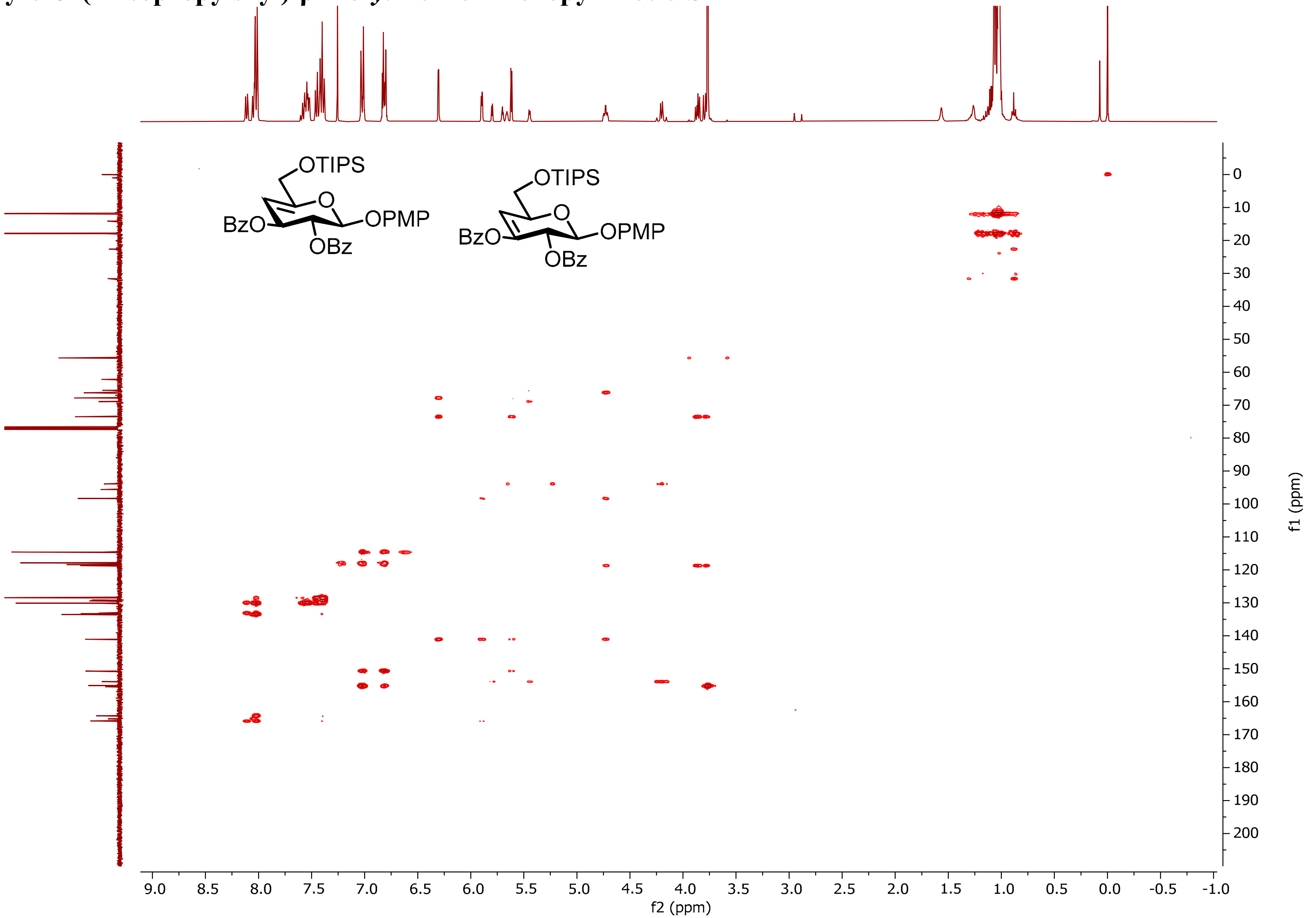

HMBC (400 × 100 MHz, Chloroform-*d*)

***p*-Methoxyphenyl 2,3-di-*O*-benzoyl-4-*O*-levulinyl-6-*O*-(triisopropylsilyl)-β-D-glucopyranoside S15**

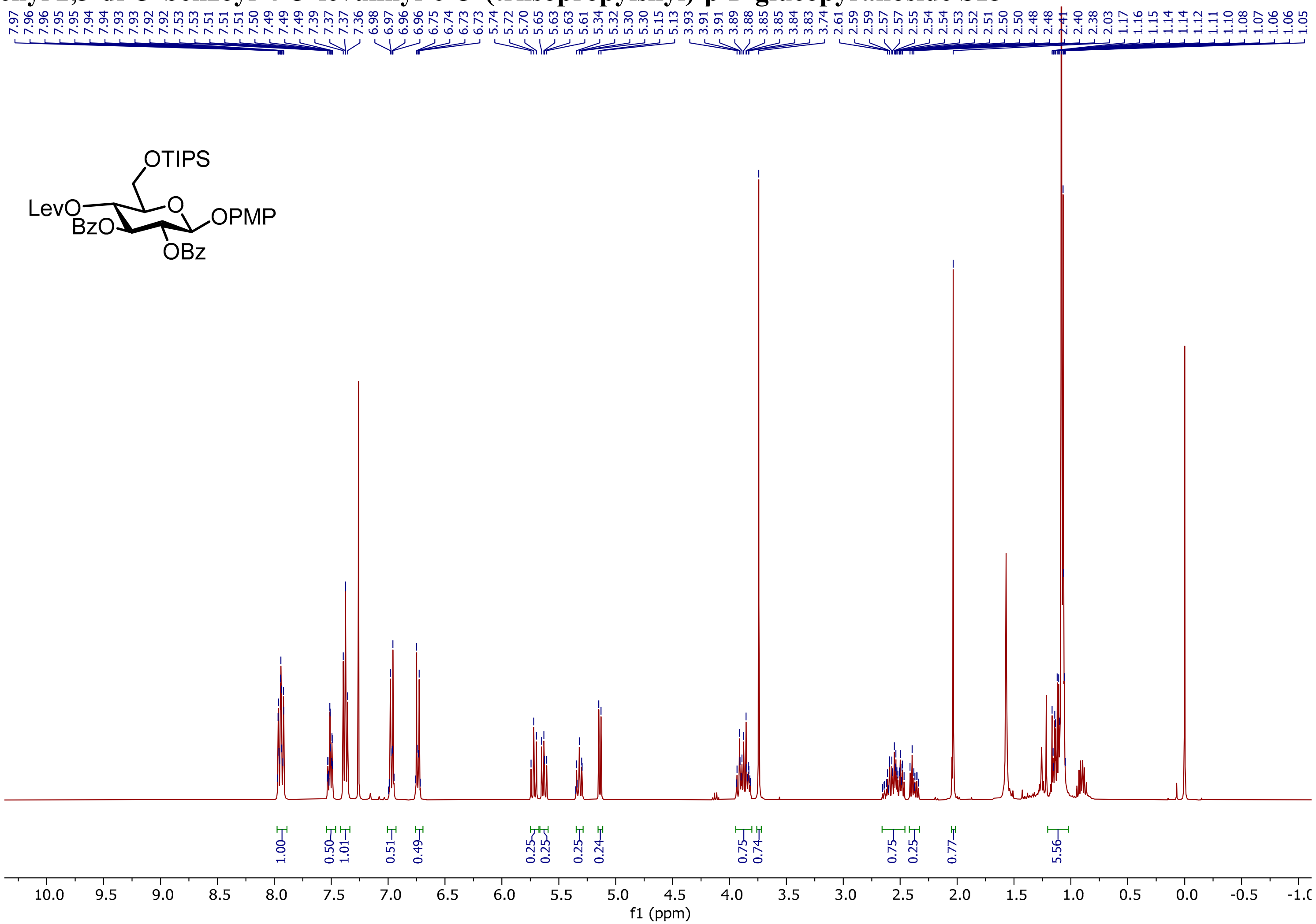

^{1}H NMR (400 MHz, Chloroform-*d*)

_p_-Methoxyphenyl 2,3-di-_O_-benzoyl-4-_O_-levulinyl-6-_O_-(triisopropylsilyl)-β-D-glucopyranoside S15

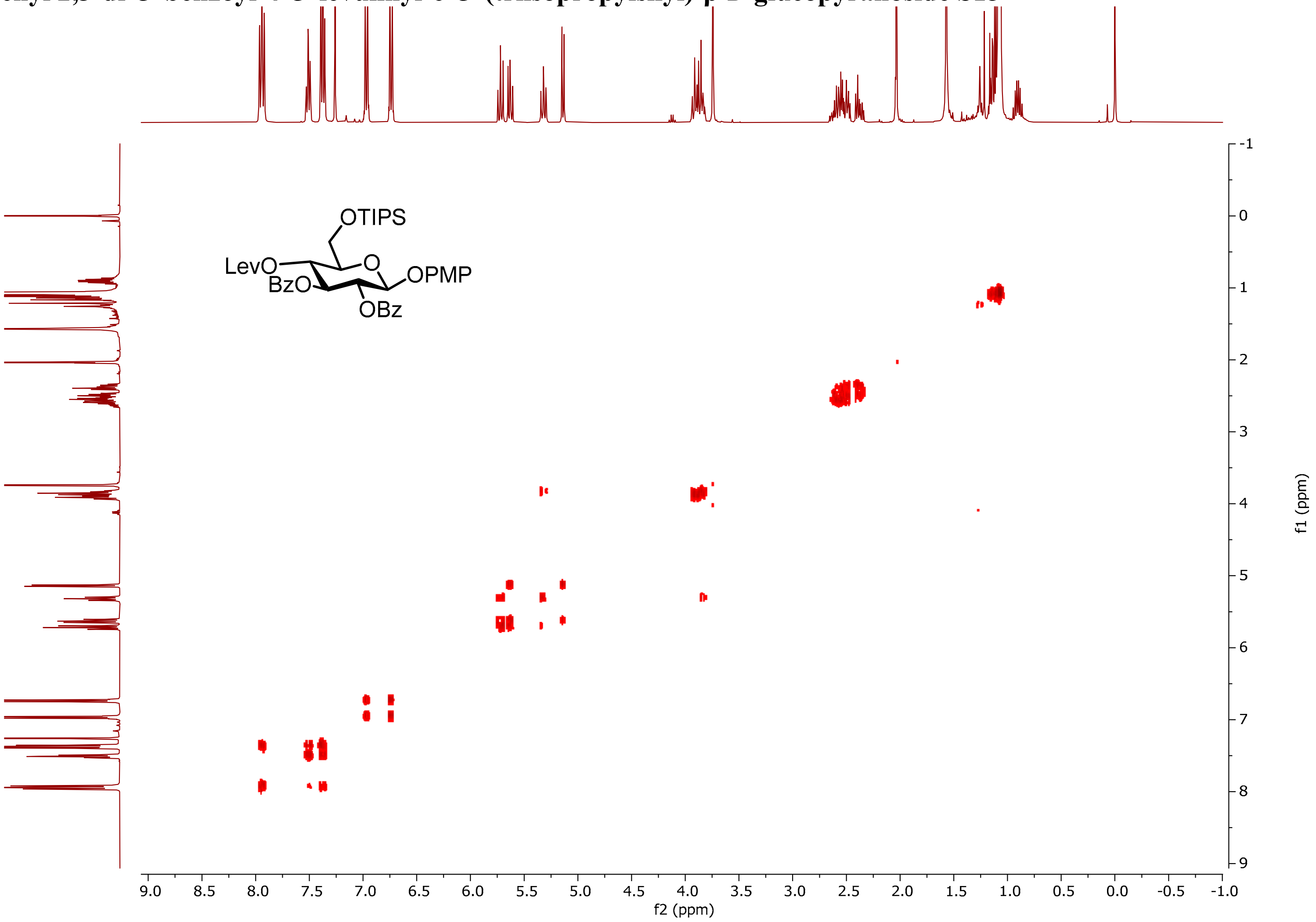

COSY (400 × 400 MHz, Chloroform-_d_)

p-Methoxyphenyl 2,3-di-O-benzoyl-4-O-levulinyl-6-O-(triisopropylsilyl)-β-D-glucopyranoside S15

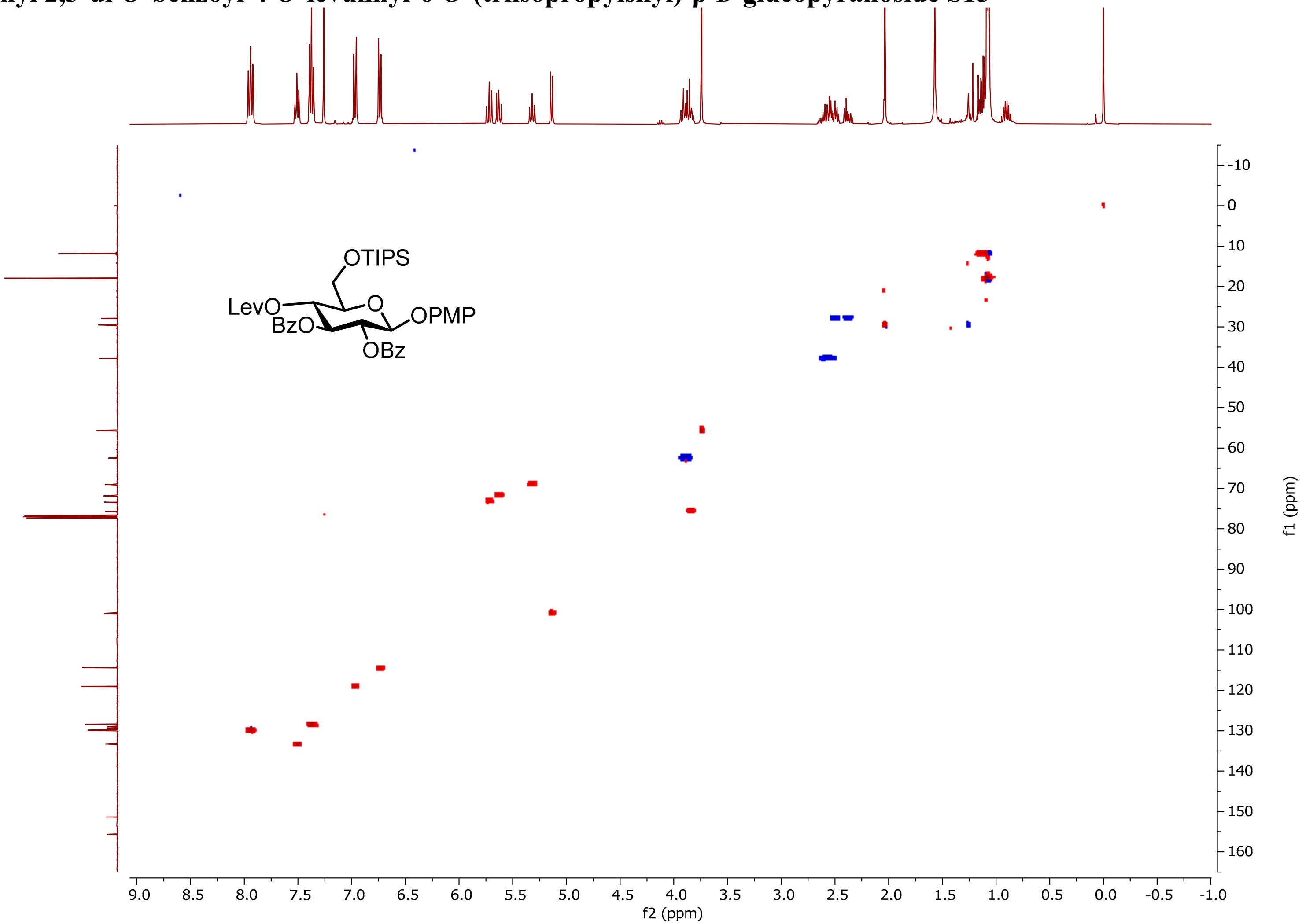

HSQC (400 × 100 MHz, Chloroform-*d*)

_p_-Methoxyphenyl 2,3-di-_O_-benzoyl-4-_O_-levulinyl-6-_O_-(triisopropylsilyl)-β-D-glucopyranoside S15

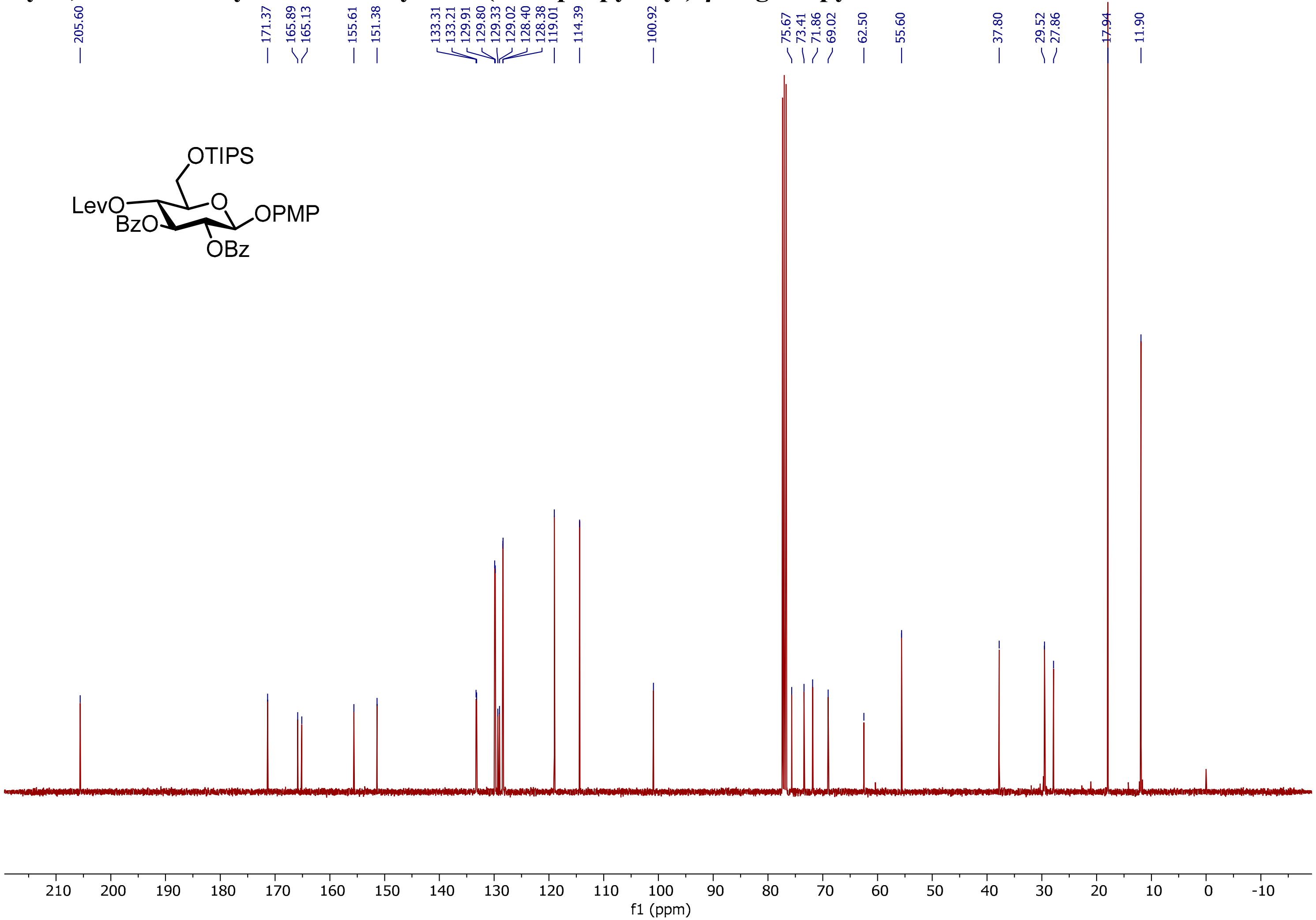

^{13}C NMR (100 MHz, Chloroform-_d_)

***S*-(2-Acetamido-3,4,6-tri-*O*-benzoyl-2-deoxy-α-D-glucopyranosyl)- (1→4)- *p*-methoxyphenyl 2,3-di-*O*-benzoyl-4-thio-β-D-glucopyranoside S17**

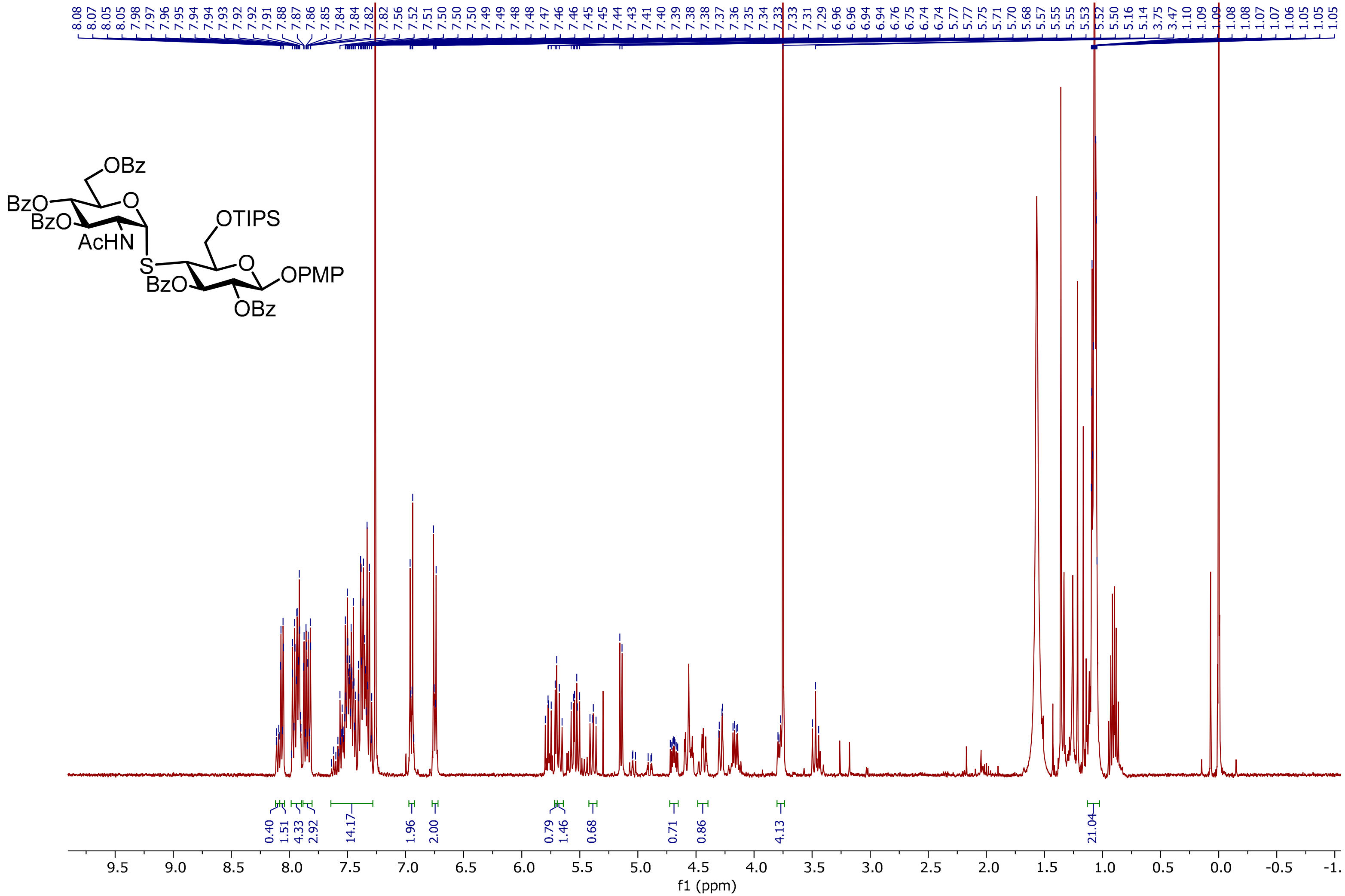

^{1}H NMR (400 MHz, Chloroform-d)

***S*-(2-Acetamido-3,4,6-tri-*O*-benzoyl-2-deoxy-α-D-glucopyranosyl)-(1→4)- *p*-methoxyphenyl 2,3-di-*O*-benzoyl-4-thio-β-D-glucopyranoside S17**

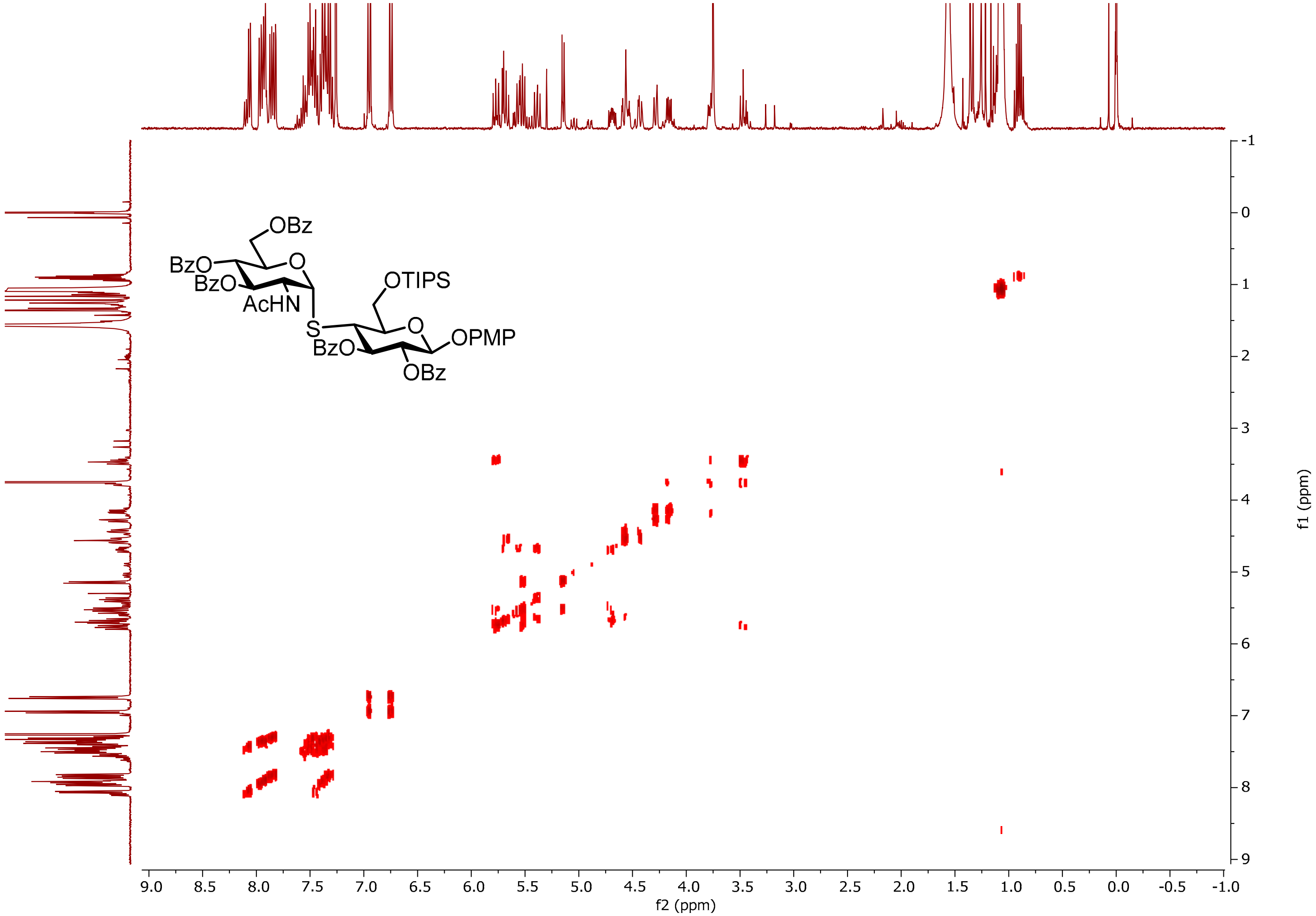

COSY (400 × 400 MHz, Chloroform-*d*)

S-(2-Acetamido-3,4,6-tri-*O*-benzoyl-2-deoxy-α-D-glucopyranosyl)-(1→4)- *p*-methoxyphenyl 2,3-di-*O*-benzoyl-4-thio-β-D-glucopyranoside S17

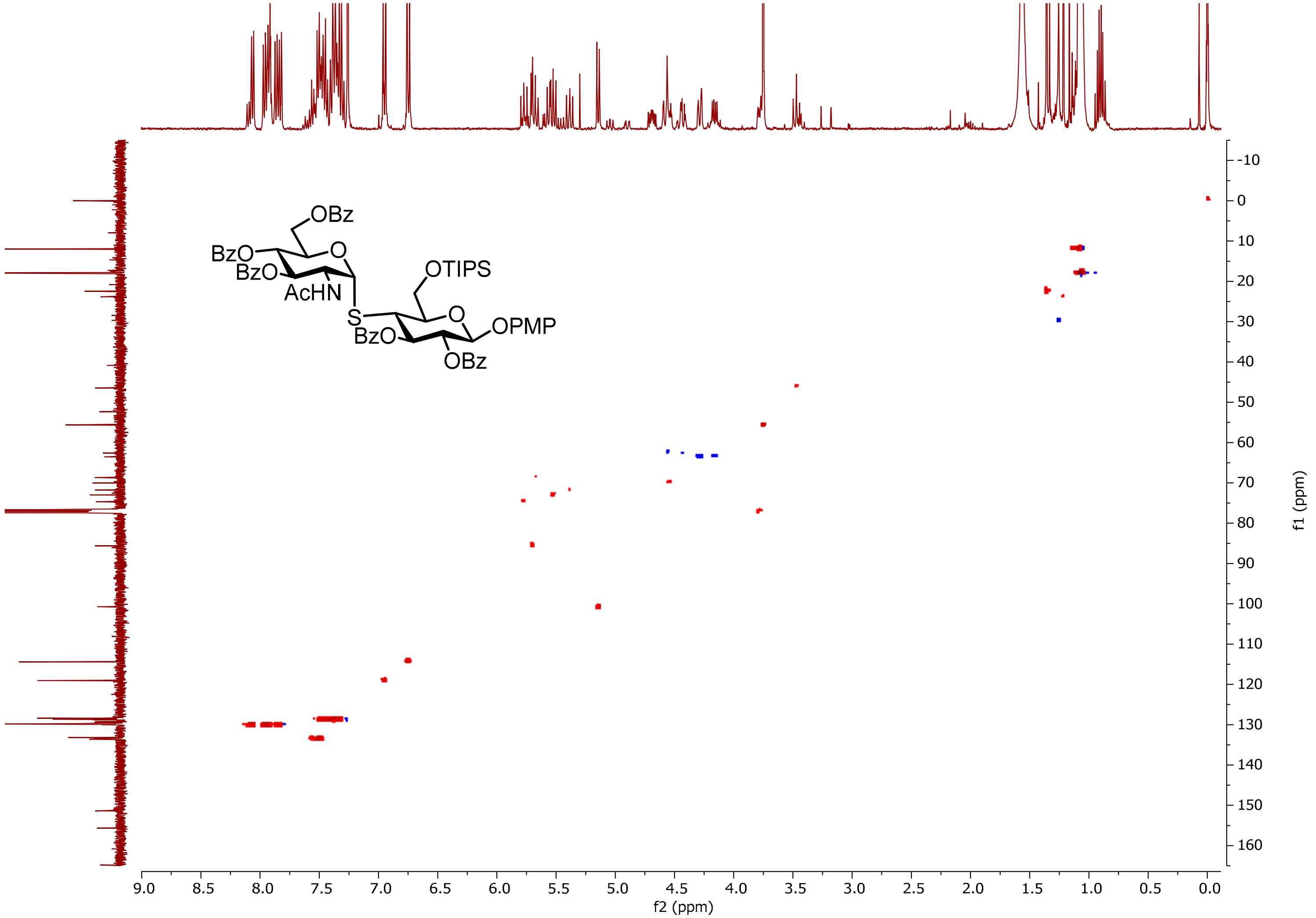

HSQC (400 × 100 MHz, Chloroform-*d*)

***S*-(2-Acetamido-3,4,6-tri-*O*-benzoyl-2-deoxy-α-D-glucopyranosyl)-(1→4)- *p*-methoxyphenyl 2,3-di-*O*-benzoyl-4-thio-β-D-glucopyranoside S17**

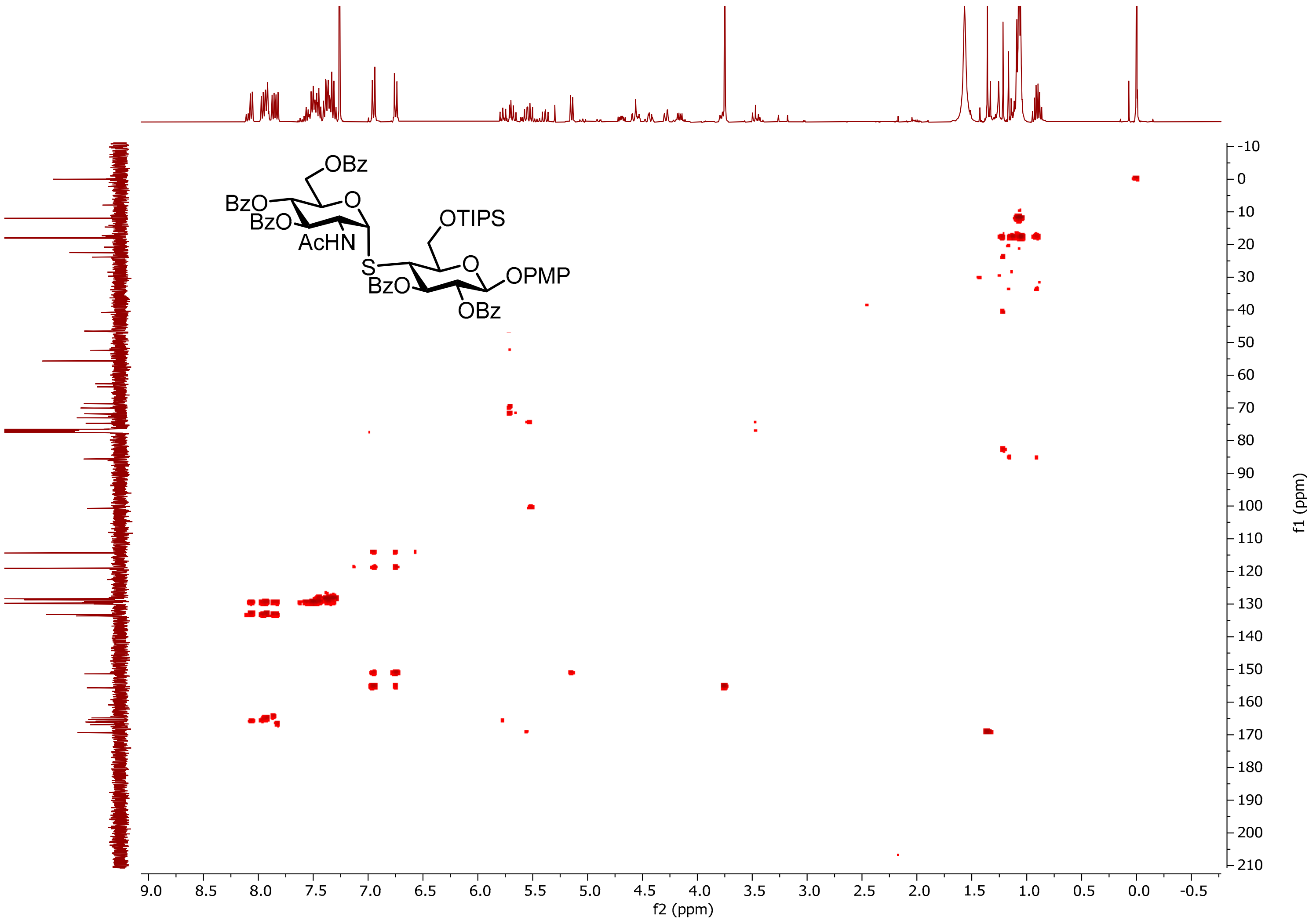

HMBC (400 × 100 MHz, Chloroform-*d*)

_S_-(2-Acetamido-3,4,6-tri-_O_-benzoyl-2-deoxy-α-D-glucopyranosyl)-(1→4)-_p_-methoxyphenyl 2,3-di-_O_-benzoyl-4-thio-β-D-glucopyranoside S17

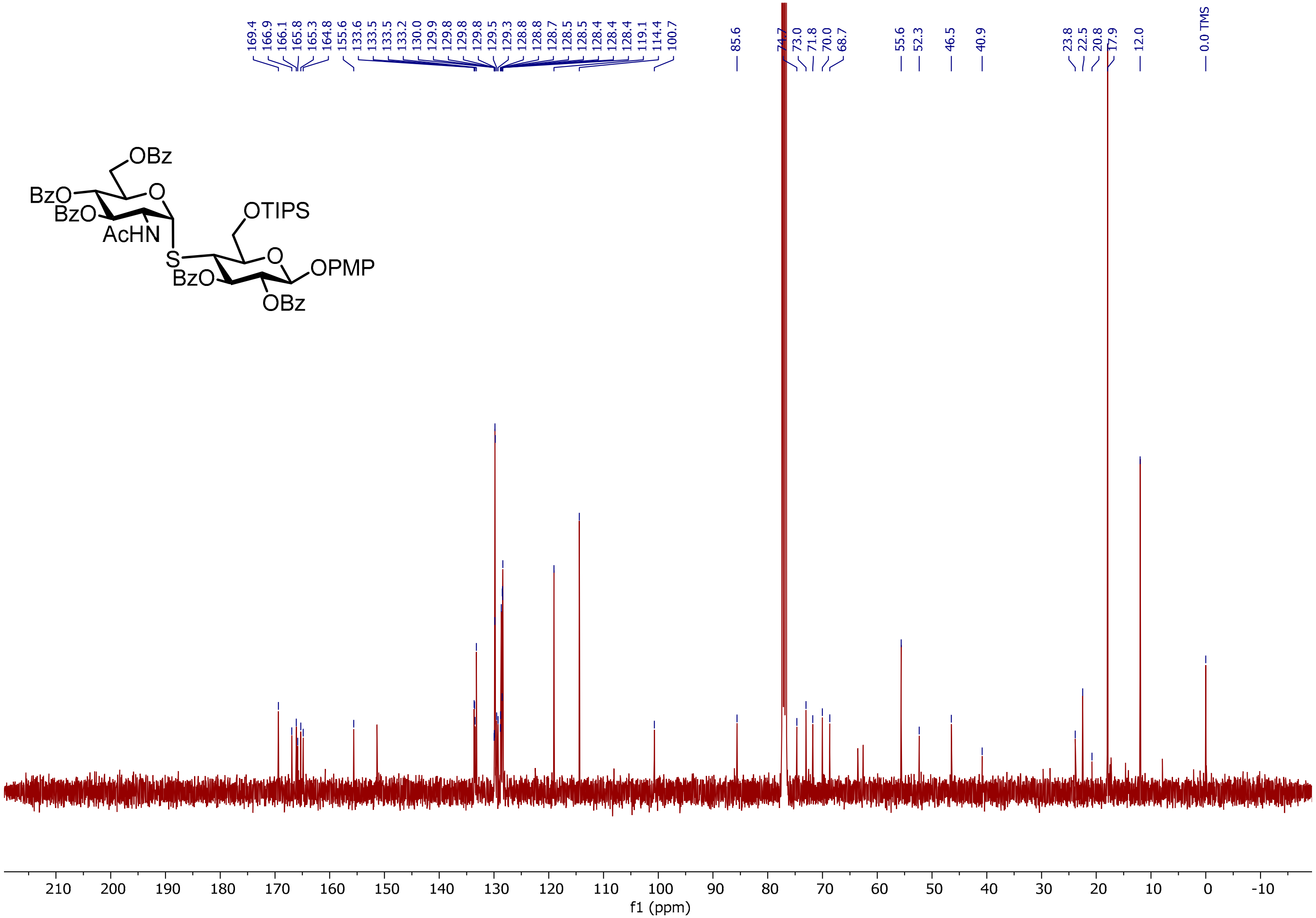

^{13}C NMR (100 MHz, Chloroform-*d*)

_S_-(2-Acetamido-3,6-di-_O_-benzoyl-2-deoxy-α-D-glucopyranosyl)-(1→4)-_p_-methoxyphenyl 2,3-di-_O_-benzoyl-4-thio-6-_O_-(triisopropylsilyl)-β-D-glucopyranoside S16

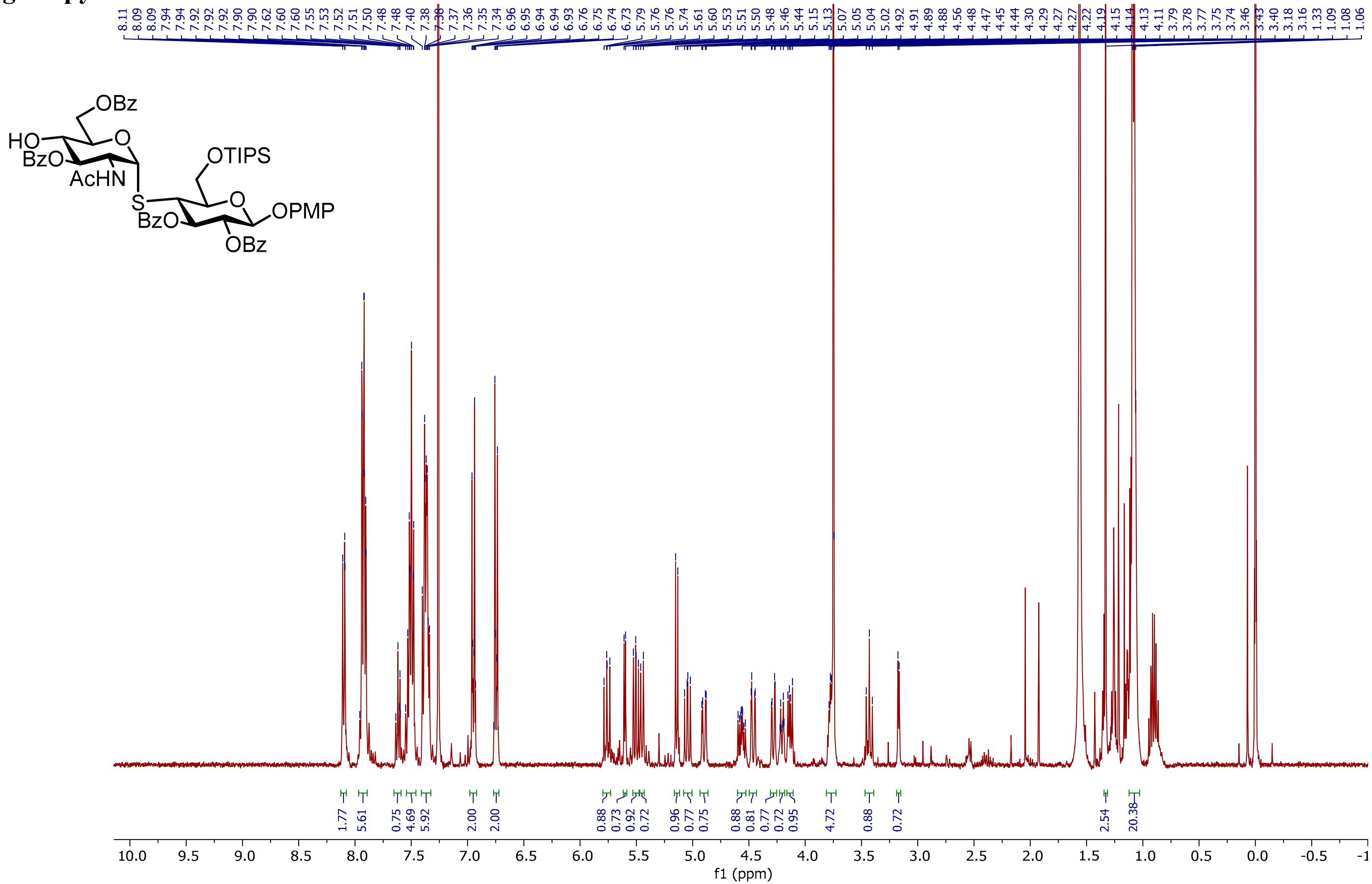

^{1}H NMR (400 MHz, Chloroform-_d_)

S-(2-Acetamido-3,6-di-O-benzoyl-2-deoxy-α-D-glucopyranosyl)-(1→4)-p-methoxyphenyl 2,3-di-O-benzoyl-4-thio-6-O-(triisopropylsilyl)-β-D-glucopyranoside S16

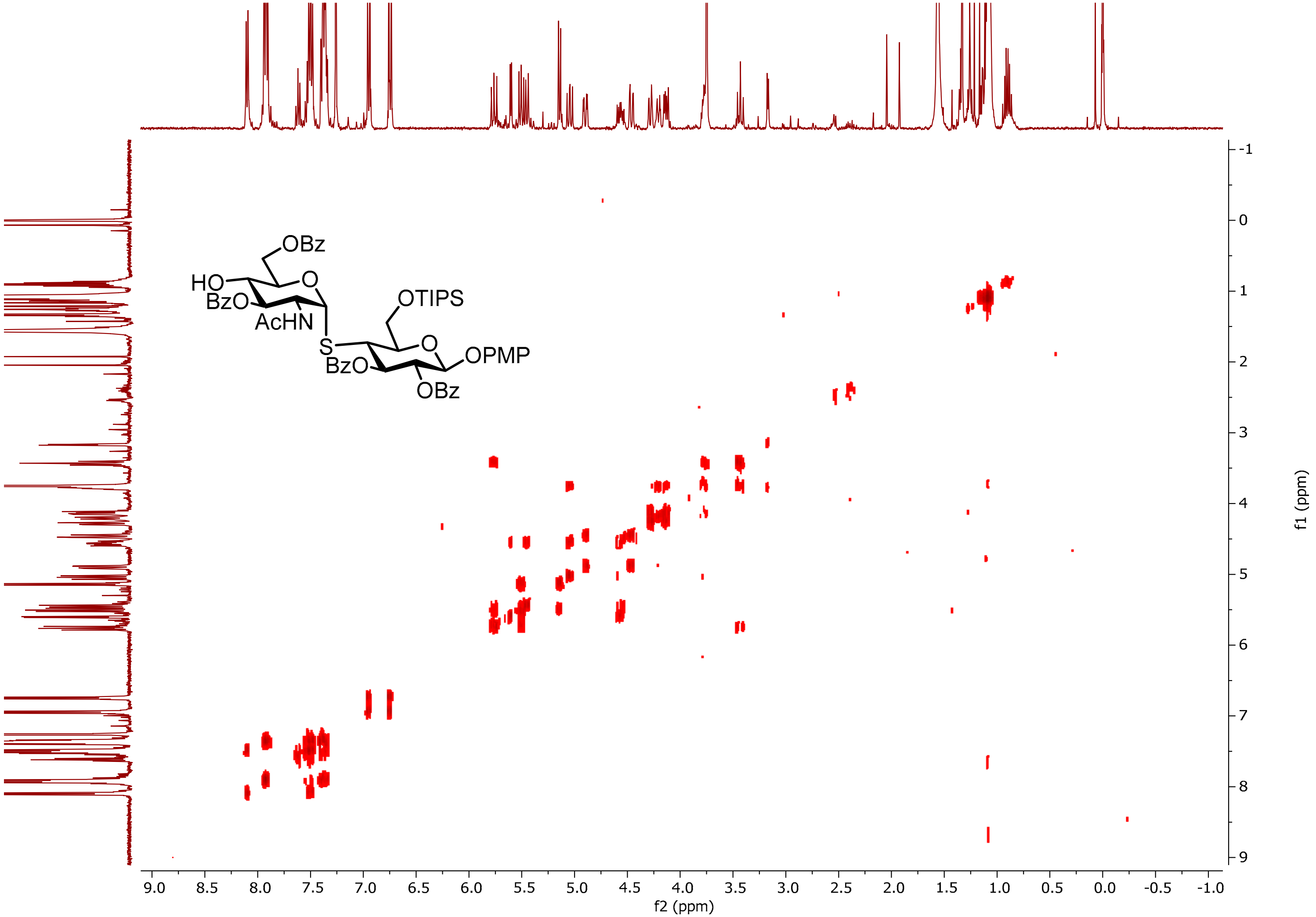

COSY (400 × 400 MHz, Chloroform-d)

_S_-(2-Acetamido-3,6-di-_O_-benzoyl-2-deoxy-α-D-glucopyranosyl)-(1→4)-_p_-methoxyphenyl 2,3-di-_O_-benzoyl-4-thio-6-_O_-(triisopropylsilyl)-β-D-glucopyranoside S16

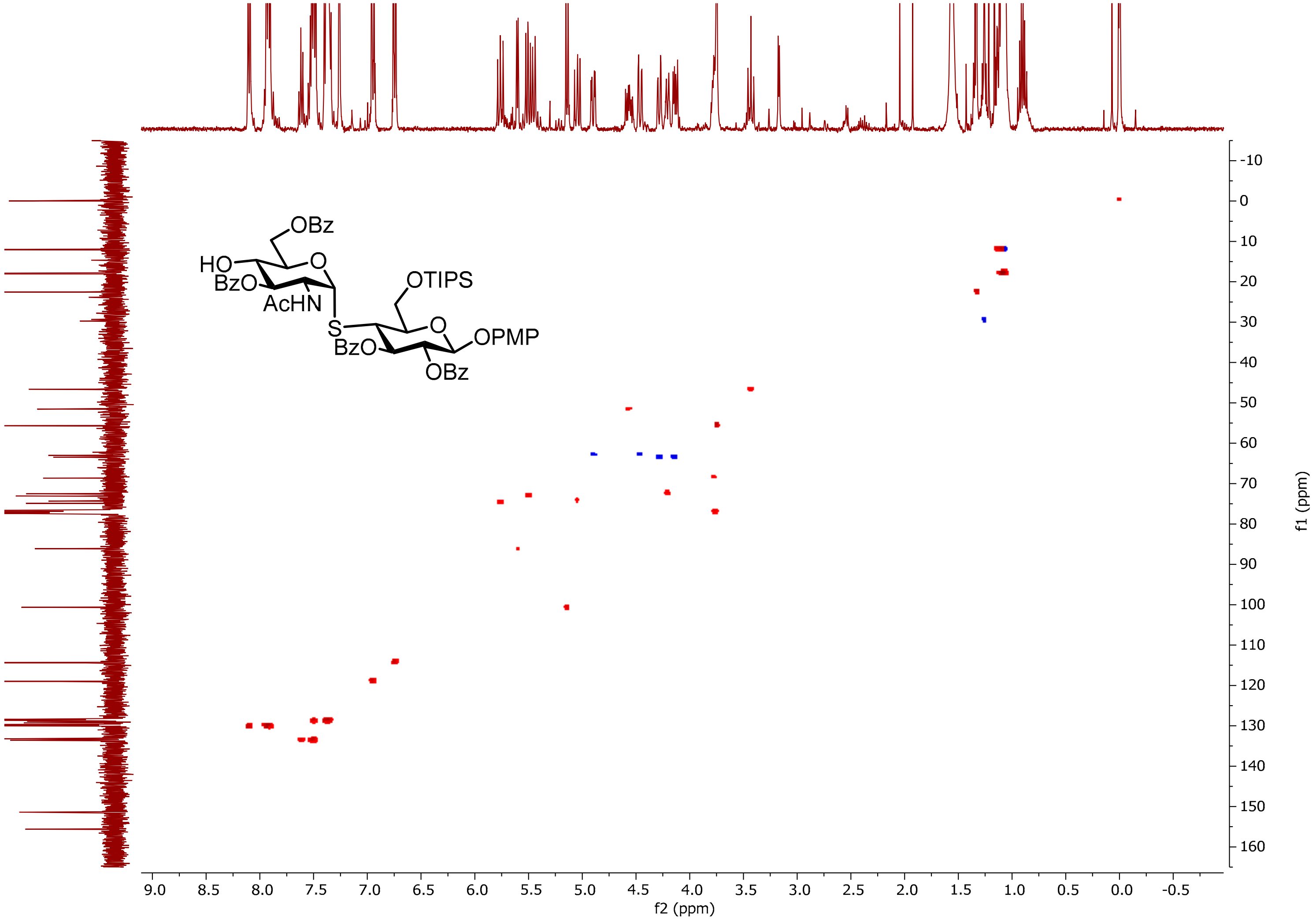

HSQC (400 × 100 MHz, Chloroform-_d_)

S-(2-Acetamido-3,6-di-O-benzoyl-2-deoxy-α-D-glucopyranosyl)-(1→4)-p-methoxyphenyl 2,3-di-O-benzoyl-4-thio-6-O-(triisopropylsilyl)-β-D-glucopyranoside S16

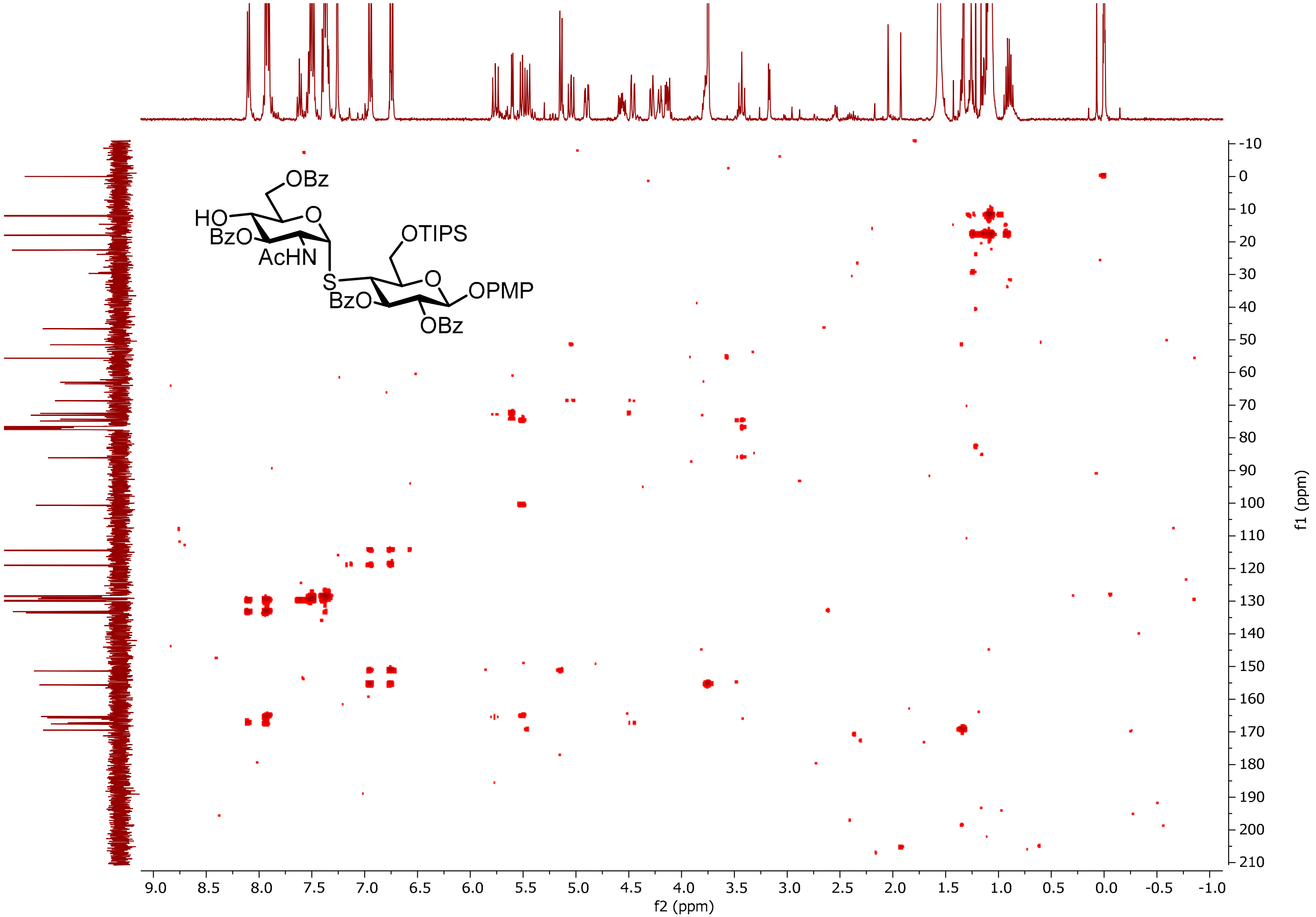

HMBC (400 × 100 MHz, Chloroform-*d*)

_S_-(2-Acetamido-3,6-di-_O_-benzoyl-2-deoxy-α-D-glucopyranosyl)-(1→4)-_p_-methoxyphenyl 2,3-di-_O_-benzoyl-4-thio-6-_O_-(triisopropylsilyl)-β-D-glucopyranoside S16

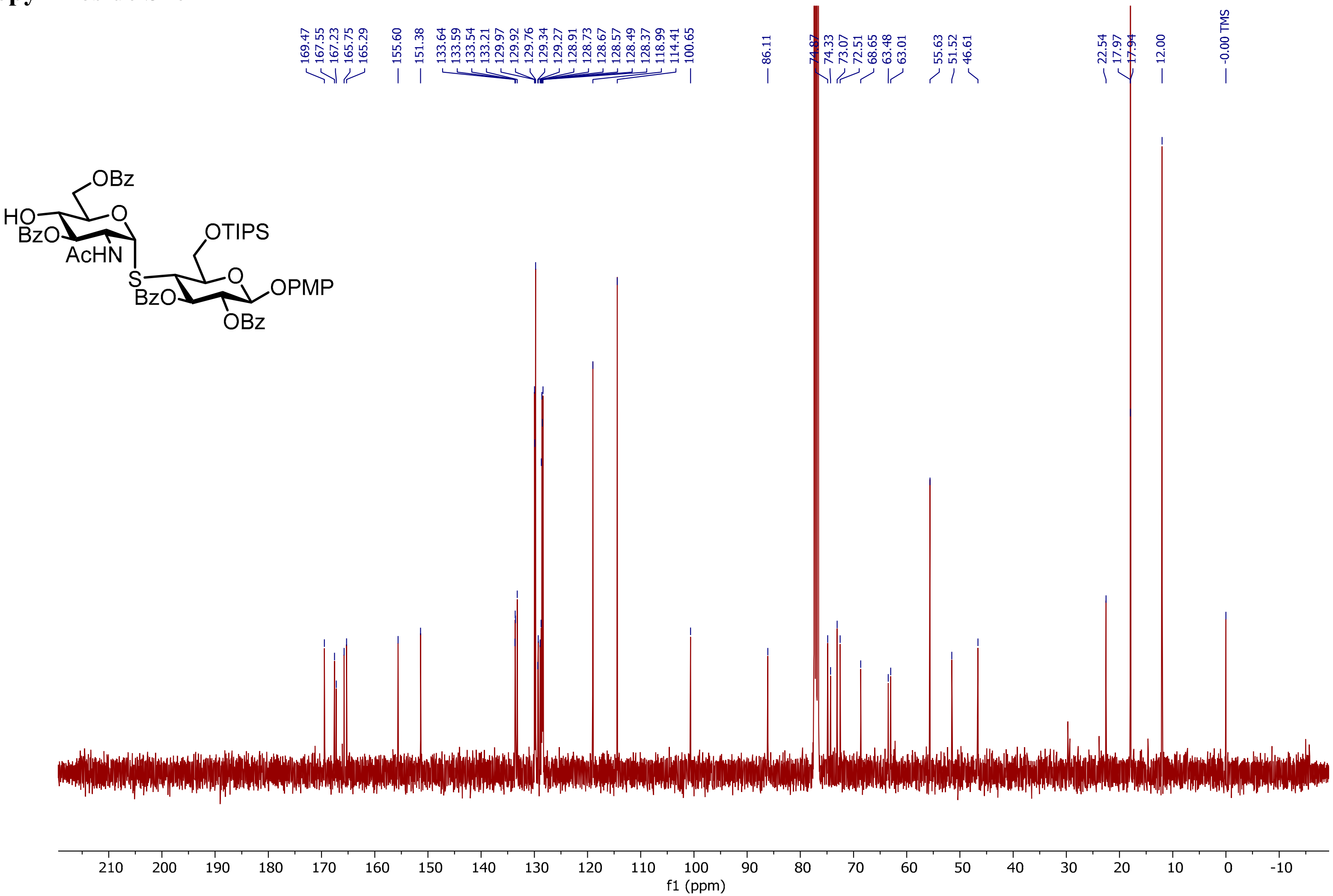

^{13}C NMR (100 MHz, Chloroform-*d*)

_p_-Methoxyphenyl 2,3-di-_O_-benzoyl-6-_O_-(triisopropylsilyl)-β-D-glucopyranoside S18

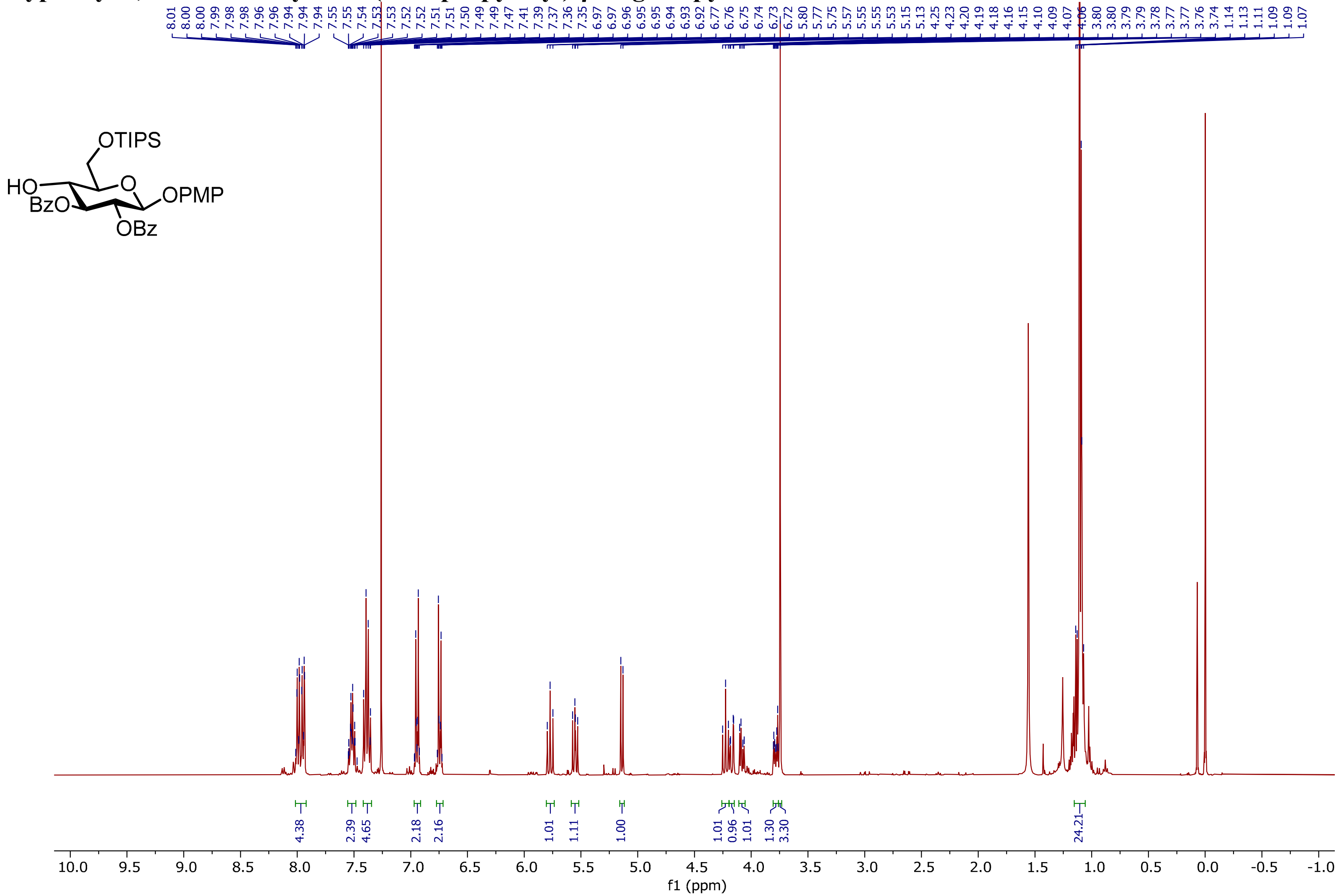

^{1}H NMR (400 MHz, Chloroform-*d*)

p-Methoxyphenyl 2,3-di-O-benzoyl-6-O-(triisopropylsilyl)-β-D-glucopyranoside S18

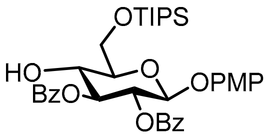

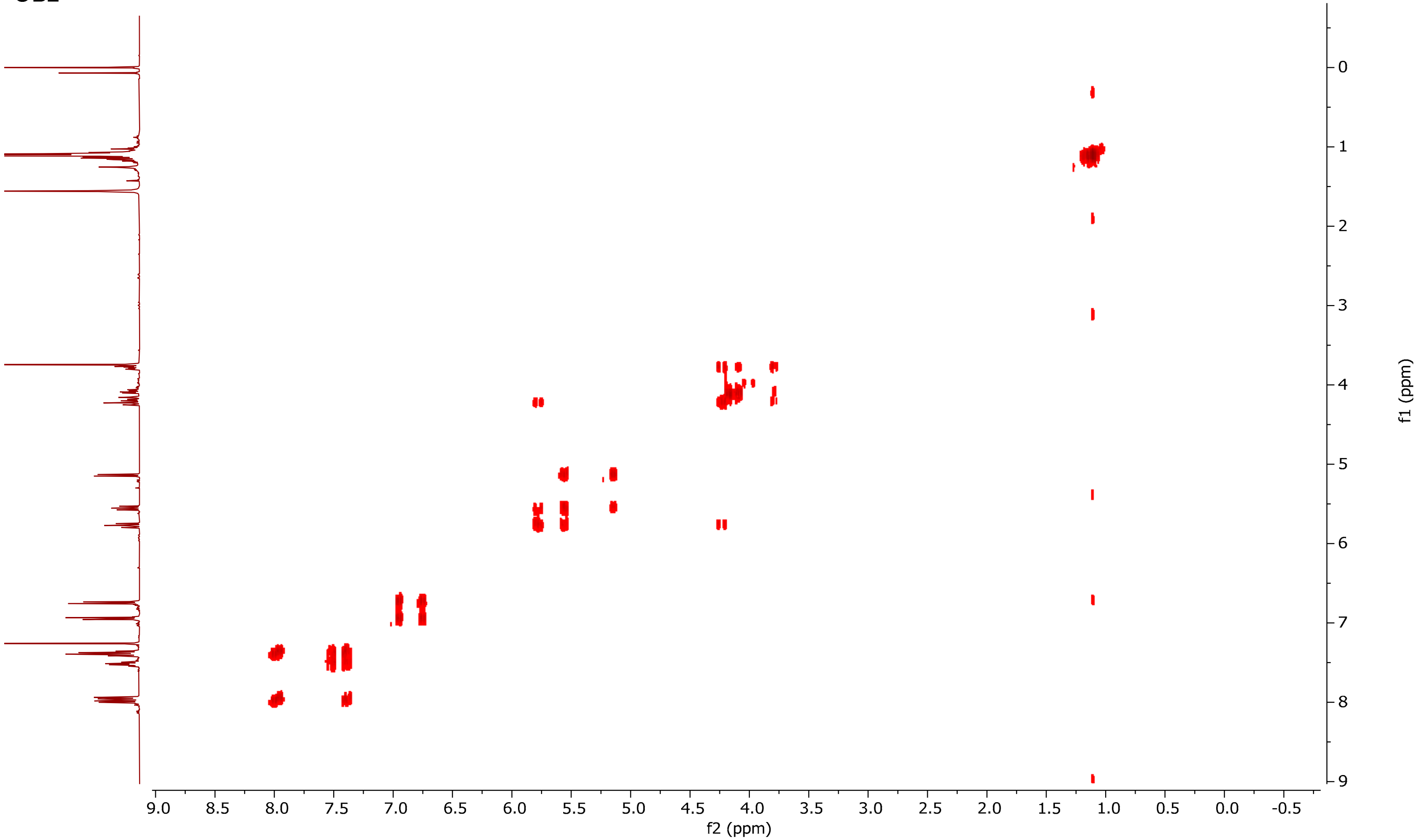

COSY (400 × 400 MHz, Chloroform-*d*)

***p*-Methoxyphenyl 2,3-di-*O*-benzoyl-6-*O*-(triisopropylsilyl)-β-D-glucopyranoside S18**

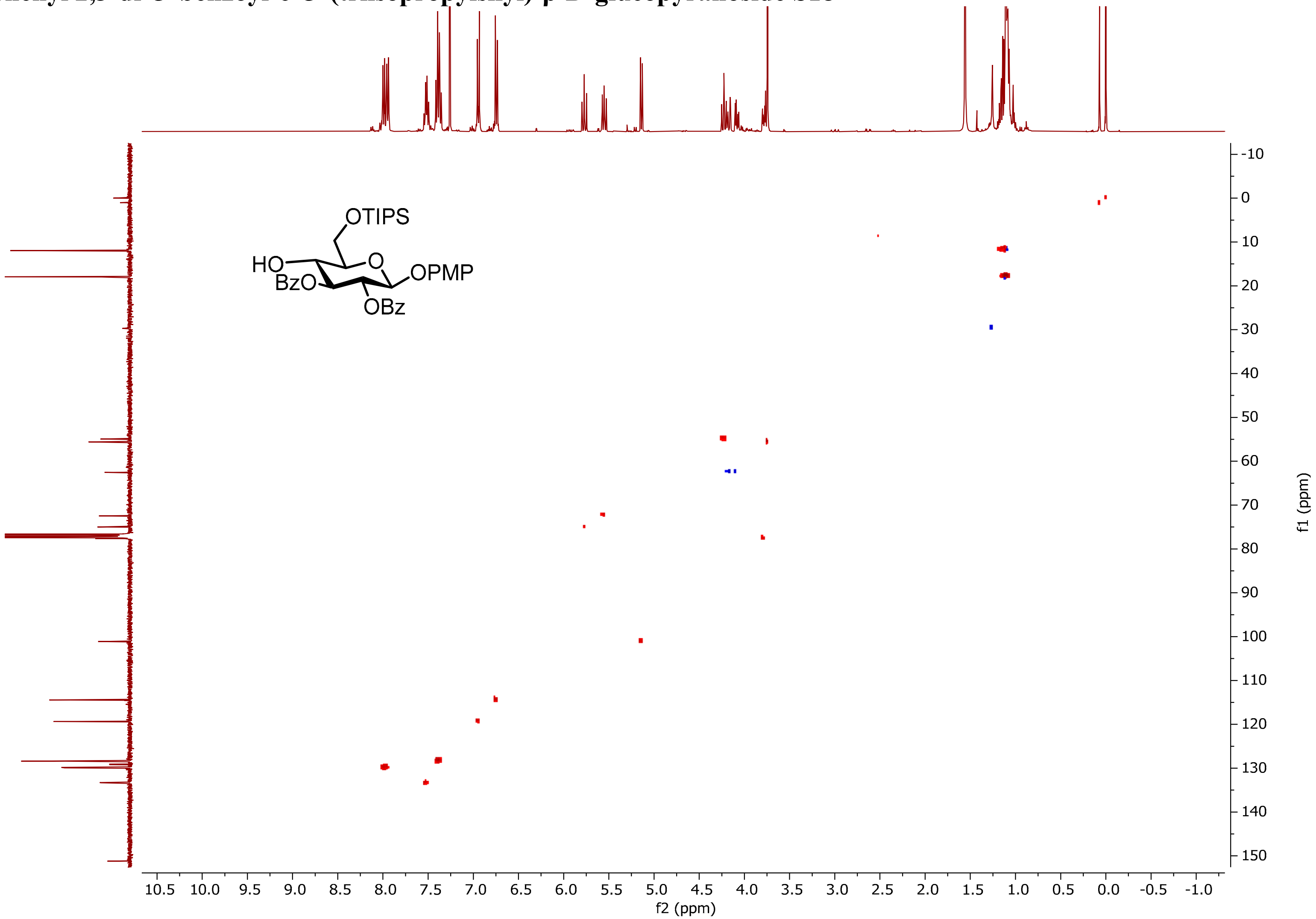

HSQC (400 × 100 MHz, Chloroform-*d*)

p-Methoxyphenyl 2,3-di-O-benzoyl-6-O-(triisopropylsilyl)-β-D-glucopyranoside S18

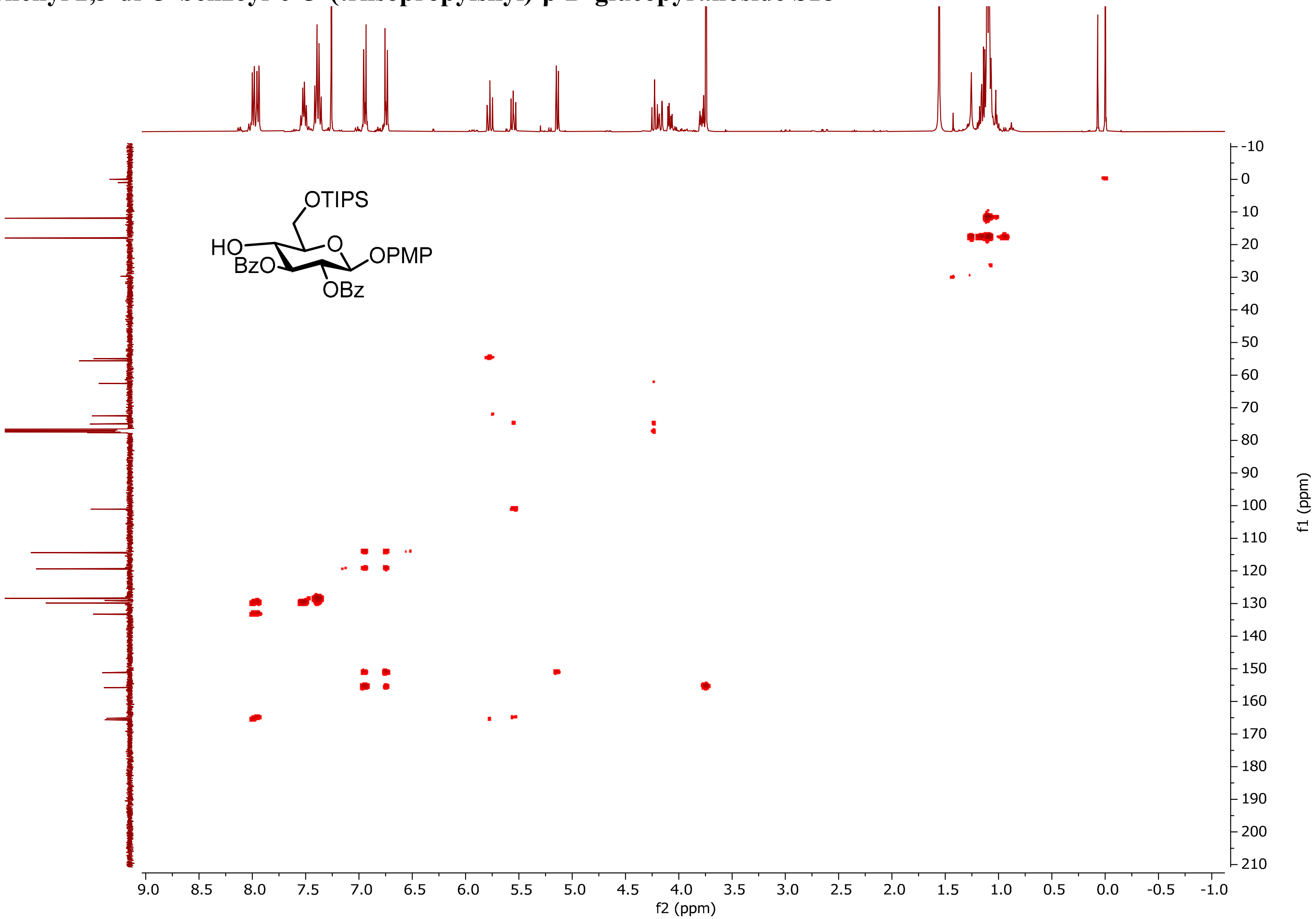

HMBC (400 × 100 MHz, Chloroform-*d*)

_p_-Methoxyphenyl 2,3-di-_O_-benzoyl-6-_O_-(triisopropylsilyl)-β-D-glucopyranoside S18

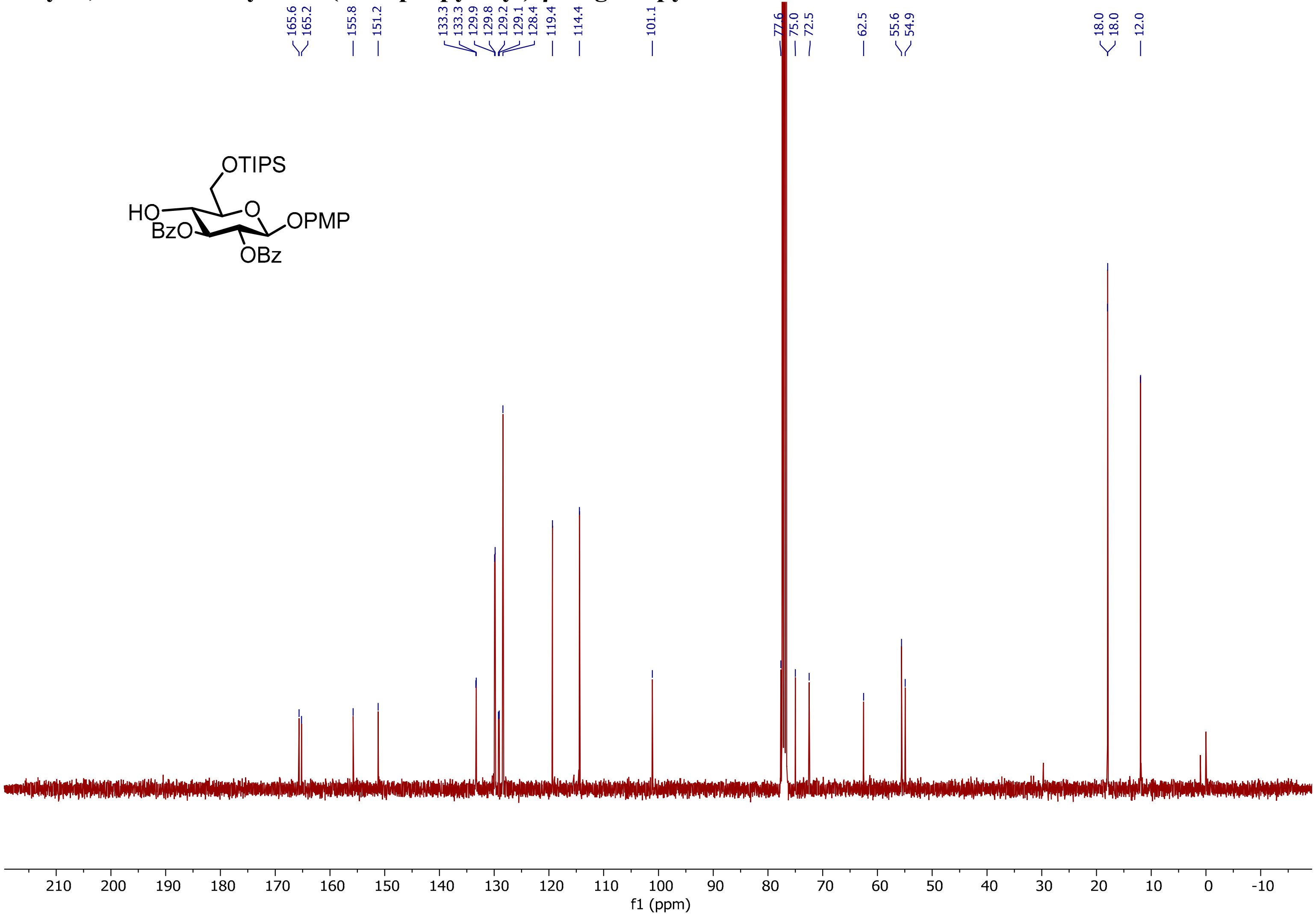

^{13}C NMR (100 MHz, Chloroform-_d_)

References

1. Chatterjee, D.; Paul, A.; Rajkamal; Yadav, S. $Cu(ClO_4)_2{\cdot}6H_2O$ catalyzed solvent free per-*O*-acetylation and sequential one-pot conversions of sugars to thioglycosides. *RSC Adv.* **2015**, *5*, 29669-29674, doi:10.1039/C5RA03461B.

2. Petermichl, M.; Schobert, R. Total Synthesis of the Diglycosidic Tetramic Acid Ancorinoside A. *Chem. Eur. J.* **2017**, *23*, 14743-14746, doi:10.1002/chem.201704379

3. Witczak, Z. J.; Kaplon, P.; Dey, P. M. Thio-sugars VII. Effect of 3-deoxy-4-*S*-(beta-D-gluco- and beta-D-galactopyranosyl)-4-thiodisaccharides and their sulfoxides and sulfones on the viability and growth of selected murine and human tumor cell lines. *Carbohydr. Res.* **2003**, *338*, 11-18. doi:10.1016/s0008-6215(02)00394-4 PubMed.

4. Abdu-Allah, H. H. M.; Tamanaka, T.; Yu, J.; Zhuoyuan, L.; Sadagopan, M.; Adachi, T.; Tsubata, T.; Kelm, S.; Ishida, H.; Kiso, M. Design, Synthesis, and Structure−Affinity Relationships of Novel Series of Sialosides as CD22-Specific Inhibitors. *J. Med. Chem.* **2008**, *51*, 6665-6681.doi:10.1021/jm8000696.

5. Lu, Y.-P.; Li, H.; Cai, M.-,.; Li, Z.-J. Synthesis of a divalent glycoside of an α-galactosyl disaccharide epitope involved in the hyperacute rejection of xenotransplantation. *Carbohydr. Res.* **2001**, *334*, 289-294, doi:10.1016/S0008-6215(01)00194-X.

6. Chang, C.-W.; Lin, M.-H.; Chan, C.-K.; Su, K.-Y.; Wu, C.-H.; Lo, W.-C.; Lam, S.; Cheng, Y.-T.; Liao, P.-H.; Wong, C.-H.; Wang, C.-C. Automated Quantification of Hydroxyl Reactivities: Prediction of Glycosylation Reactions. *Angew. Chem. Int. Ed.* **2021**, *60*, 12413-12423, doi:10.1002/anie.202013909.

7. Geert Volbeda, A.; van Mechelen, J.; Meeuwenoord, N.; Overkleeft, H. S.; van der Marel, G. A.; Codée, J. D. C. Cyanopivaloyl Ester in the Automated Solid-Phase Synthesis of Oligorhamnans. *J. Org. Chem.* **2017**, *82*, 12992-13002,doi:10.1021/acs.joc.7b02511.

8. Pongener, I.; Miller, G. J. d-Glucuronate and d-Glucuronate Glycal Acceptors for the Scalable Synthesis of d-GlcN-α-1,4-d-GlcA Disaccharides and Modular Assembly of Heparan Sulfate. *J. Org. Chem.* **2023**, *88* (15), 11130-11139, doi:10.1021/acs.joc.3c01108.
